# Terpenes as Naturally Occurring Stereochemical Templates: Conformationally Driven Discovery of Reactivity

**DOI:** 10.1021/acs.orglett.5c03431

**Published:** 2025-09-23

**Authors:** Omar Arto, Rubén Miguélez, Hannah Siera, Jan Schulte, Isabel Merino, Gebhard Haberhauer, Pablo Barrio

**Affiliations:** † Department of Organic and Inorganic Chemistry, 16763 Universidad de Oviedo, Julian Clavería 8, 33006 Oviedo, Spain; ‡ Institut für Organische Chemie, Universität Duisburg-Essen, Universitätstraße 7, 45117 Essen, Germany; § Servicios Científico Técnicos, 16763 Universidad de Oviedo, Fernando Bonguera s/n, 30006 Oviedo, Spain

## Abstract

Natural terpene-derived substrates have been used as stereochemical templates in the gold­(I)-catalyzed cycloisomerization of aliphatic 1-bromoalkynes. The conformational restrictions imposed by these substrates have unlocked an unprecedented 5-*exo* cyclization mode in gold catalysis. This reactivity was extended to structurally simplified substrates.

## Graphical abstract

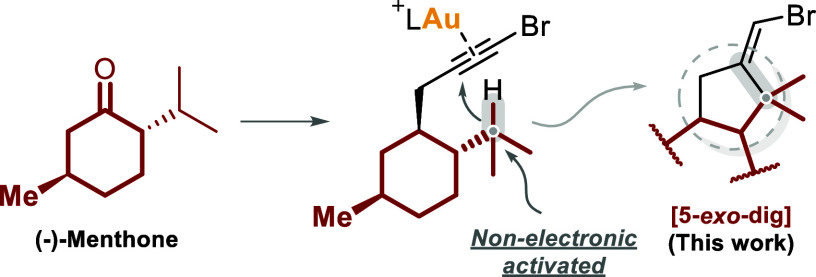

Natural products have served both as a source of inspiration and as a source of feedstock materials for synthetic organic chemists. Hence, on one hand, their intricate molecular architectures assembled with exquisite selectivity by Nature pose a challenge that has been masterfully tackled since Wholer’s milestone synthesis of urea almost a century ago.^1,2^ On the other hand, chemists have also recognized the potential of natural products as starting materials for the synthesis of more complex ones. This has been accomplished using two different approaches, the so-called semisynthesis and chiral pool-based synthesis. In semisynthesis, a very large and complex fragment of the final molecule is obtained from Nature and usually can be produced in significant quantities.^3^ Perhaps the most important example is the semisynthesis of paclitaxel (Taxol) that ensured sufficient supply for the treatment of cancer patients worldwide.^4^ The chiral pool approach relies on Nature’s surgical precision for the installation of stereocenters.^5^ In this way, a commonly not so large fragment of the molecule is borrowed from Nature as the starting material with part or all of the stereochemistry of the final natural product fixed from the onset of the synthesis. Among the several classes of natural products, terpenes play a paramount role in the chiral pool approach (Figure 1A).^6^ They have served as starting materials for not only the synthesis of other natural products but also the preparation of chiral reagents, auxiliaries, and ligands (Figure 1C).^7^ In this work, terpene-derived 1-bromoalkynes have been used as stereochemical templates with fixed conformations for the study of conformational effects in the gold­(I)-catalyzed cycloisomerization of aliphatic 1-bromoalkynes recently reported by our group (Figure 1B).^8,9^

**Figure 1:**
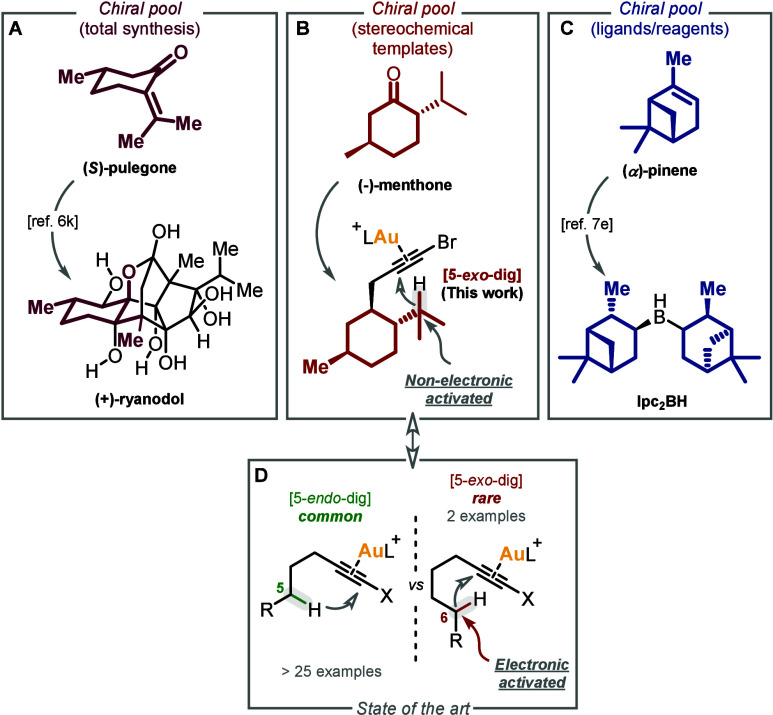
Terpenes as privileged chiral pool substrates (A) for the total synthesis of natural products, (C) for the development of new chiral reagents, and (B) as stereochemical templates. (D) 5-*exo*-dig vs 5-*endo*-dig cyclization modes in gold­(I)-catalyzed C­(sp^3^)–H bond functionalization.

Remarkably, the use of these terpene-derived multisubstituted substrates has unlocked a novel 5-*exo* reactivity mode. The 5-*exo* cyclization mode is often the preferred pathway in some of the best-studied gold-catalyzed reactions, intramolecular nucleophilic additions,^10^ and as the first step in the cycloisomerization of 1,6-enynes^11–13^ and the 1,2-[shift] in propargyl esters.^14,15^ However, it is quite rare for C–H insertions and limited to activated C­(sp^3^)–H bonds (Figure 1D).^16^

In a previous study, we have shown how conformational effects dictate the reactivity and hence the selectivity of the gold­(I)-catalyzed cycloisomerization of aliphatic 1-bromoalkynes.^9^ Aiming to tackle more complex substitution patterns and stereochemical settings, we identified terpenes as ideal starting materials. Specifically, the complementary substitution patterns of carvone and menthone allowed us to envision several interesting derivatives with complete control over the position of the methyl and isopropyl groups, the relative stereochemistry, and, if desired, the absolute stereochemistry, affording enantiopure compounds (Figure 2). Moreover, the relative stereochemistry of the newly created stereocenter, bearing the reactive bromoalkynyl unit, may be chosen at will by means of stereoselective synthesis. In summary, the so-called “menthone” and “carvone” series substrates differ in the relative position of the methyl and isopropyl substituents. The relative stereochemistry of the methyl and isopropyl groups is always *trans*, while the relative stereochemistry of the third stereocenter can be defined by synthesis. Finally, two classes of compounds have been used, differing in the nature of the bromoalkyne unit attached to the cyclohexane ring: bromoethynyl (directly attached to the ring) and bromopropargyl (separated by a methylene tether). After an intensive synthetic effort, eight possible derivatives, **1a**–**d** and **2a**–**d**, were successfully synthesized, in most cases as single diastereoisomers (see the Supporting Information for details). All reactions are carried out without any deviation from the originally reported reaction conditions ([IPrAu­(NCMe)]­[SbF_6_] (2.5 mol %), CH_2_Cl_2_, 80 °C, 2 h^8a,17^ ); in this way, the conformational effect may be studied aside from any other experimental variable.

**Figure 2:**
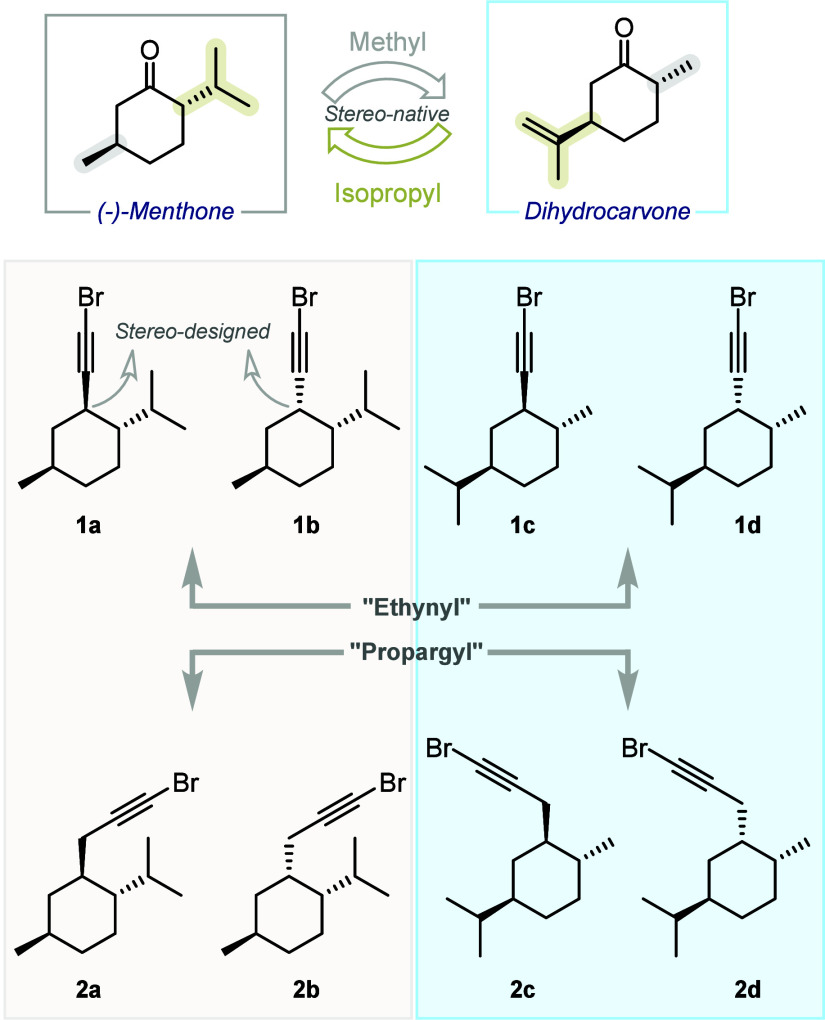
Terpene-derived substrates.

We started our study by comparing the reactivities of both menthone derivatives in bromoethynyl compounds **1a** and **1b**. The 1,4-*trans* relationship of the methyl and isopropyl substituents ensures their equatorial disposition, allowing us to evaluate the influence of placing the bromoethynyl reactive moiety in either the equatorial or the axial position on reactivity. We expected all-equatorial derivative **1a** to be unreactive due to the very unfavorable all-axial reactive conformation required. However, we overlooked the presence of an alternative reactive C­(sp^3^)–H bond in a 1,5 relative disposition to the bromoalkyne at the isopropyl exocyclic position. Hence, fused derivative **3a** was obtained instead of the expected bridged derivative, although in moderate yield (Scheme 1). According to DFT calculations, insertion into the exocyclic C­(sp^3^)–H bond of the ^i^Pr group is favored by more than 10 kcal/mol over insertion into the endocyclic C­(sp^3^)–H bond (Figure S1 and the Supporting Information). Conversely, expected bridged derivative **4b** was obtained as the major product for **1b**. As expected from the result obtained in our previous studies, a single regioisomer arising from the reaction at the tertiary C­(sp^3^)–H bond was observed.^9^ Due to the reaction at the exocyclic isopropyl C­(sp^3^)–H bond, fused derivative **3b** was obtained as the minor product. Then we switched to the carvone series. The reaction of all-equatorial derivative **1c** afforded a complex mixture of up to four different products (see the NMR spectra in the Supporting Information). On the other hand, **1d** behaved like **1b**, affording bridged product **4d** as a single regioisomer. The lower reactivity of the methyl C­(sp^3^)–H bonds prevents a competitive reaction at this position. These results provide evidence of how the conformational preferences of the substrates dictate their reactivity.

**Scheme 1:**
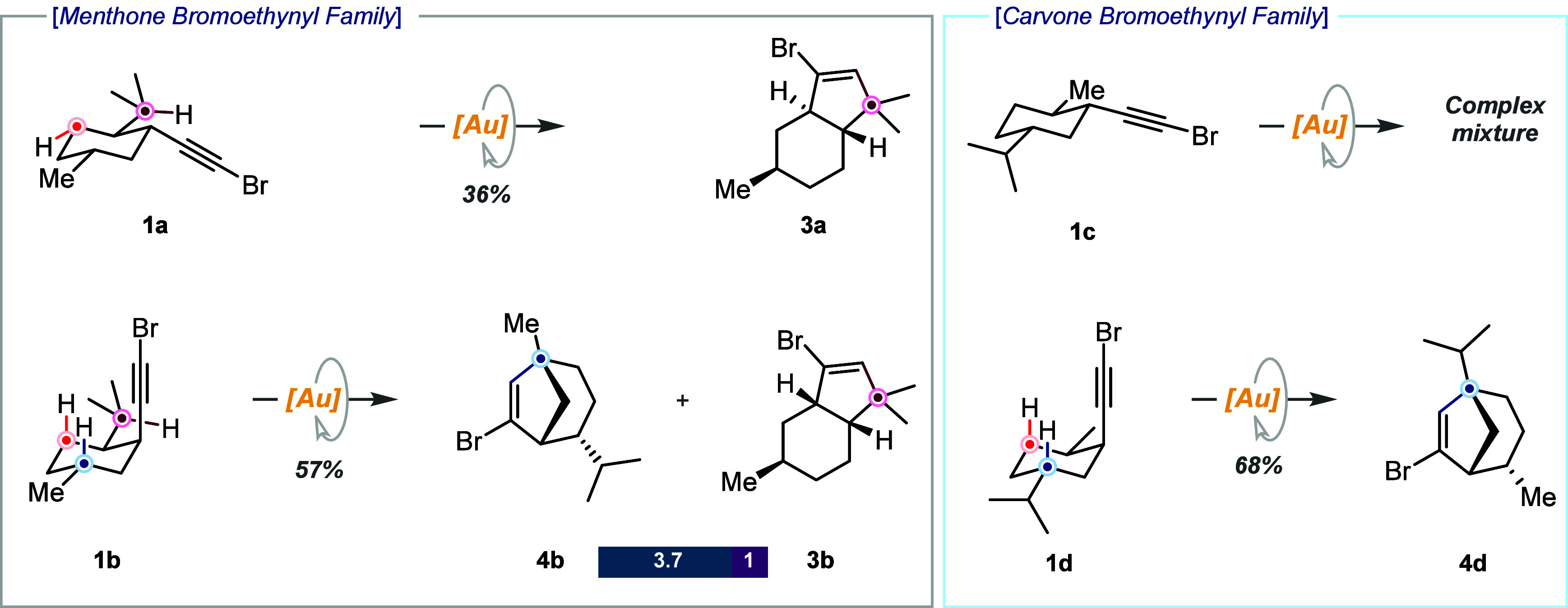
Bromoethynyl Family^a^

a Yields and ratios determined by ^1^H NMR of the crude reaction mixtures using CH_2_Br_2_ as the internal standard.

An even stronger influence was observed in the bromopropargyl series. Akin to the bridged series, we started our study with menthone derivatives **2a** and **2b**. In this case, for all-equatorial conformer **2a**, a CH versus CH_2_ scenario was anticipated, since several reactive C–H bonds in relative position 5 were available. Unexpectedly, a new product **5a** arising from the reaction with the exocyclic isopropyl C­(sp^3^)–H bond was obtained, in a remarkable 88% yield (Scheme 2).[Fn fn1] Several features of this transformation are worth noting. (1) This is the first example of a gold­(I)-catalyzed intramolecular insertion into a non-activated C­(sp^3^)–H bond following a 5-*exo* cyclization mode (see Scheme 3).^16^ (2) The new 5-*exo* reactivity occurred at a C­(sp^3^)–H bond in relative position 6 to the bromoalkyne, in the presence of several C­(sp^3^)–H bonds potentially reactive in the “normal” 5-*endo* reactivity. (3) The reaction shows complete selectivity with 88% yield overwhelming the “normal reactivity”. (4) This reactivity switch may be explained by the shallow activation barrier associated with its transition state (11 kcal/mol) (Figure S2 and computational details in the Supporting Information). (5) The discovery of this new reactivity was driven by conformational bias. The key role of conformational effects in the new 5-*exo* reactivity was brought to light by the outcome of the reaction with diastereoisomer **2b**. Now, in addition to the analogous 5-*exo* product at the isopropyl C–H bond in **5b**, a second 5-*exo* product involving an endocyclic tertiary C–H bond in **6b** was formed in a significant 12% yield. In the carvone series, the lack of a 2-isopropyl group in 1,2-*trans* derivative **2c** restores the “normal” 5-*endo*-fused reactivity, favoring the cyclization at the CH group over the CH_2_ group (2:1) with poor diastereoselectivity for minor regioisomer **7c** (3:1). The regioselectiviy and diasatereoselectivity of this and other complex reaction mixtures were established by integration of the characteristic olefinic signals in the ^1^H NMR spectrum, which were unambiguously assigned by selective 1D TOCSY and 1D NOESY experiments, together with careful analysis of 2D NMR experiments (see the NMR spectra in the Supporting Information). This result mimics almost perfectly that obtained in our previous study with a truncated substrate.^9^

**Scheme 2:**
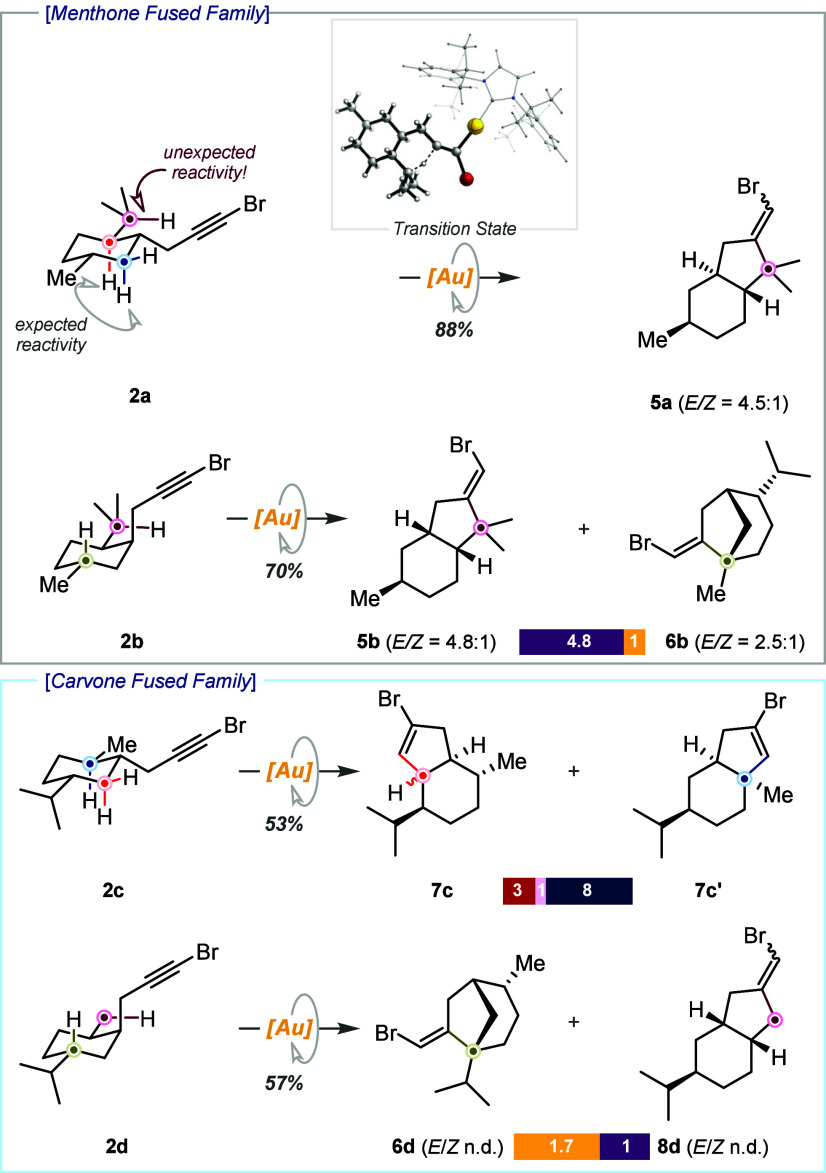
Unlocking New Reactivity in the Bromopropargyl Family^a^

a Yields and ratios determined by ^1^H NMR of the crude reaction mixtures using CH_2_Br_2_ as the internal standard.

**Scheme 3:**
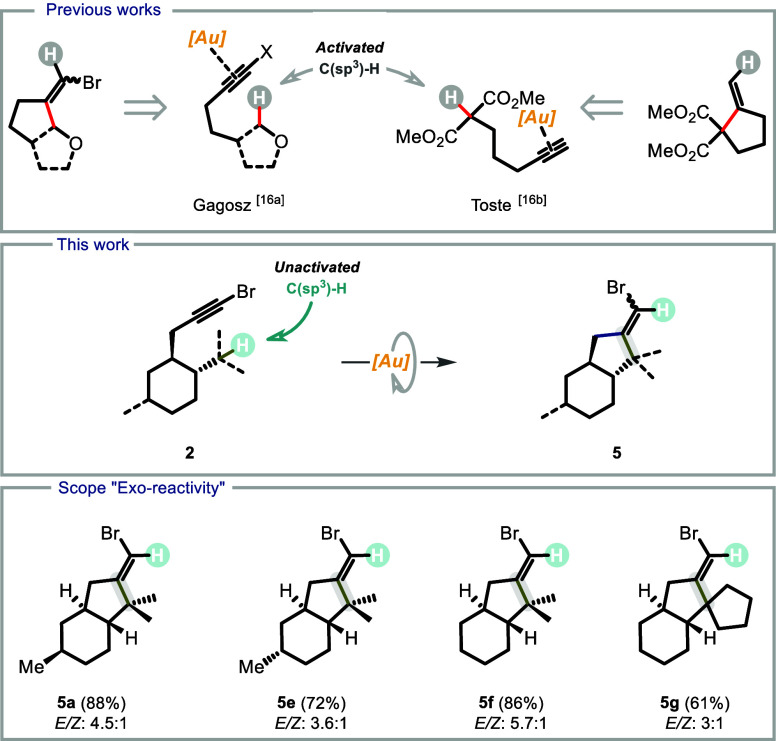
Preliminary Assessment of the Generality of the Novel 5-*exo*-dig Reactivity

Switching the relative stereochemistry of the bromopropargyl unit in **2d** resulted in a new unexpected reaction outcome. In addition to the 5-*exo* product at the endocyclic *cis* C­(sp^3^)–H bond in position 5 of the cyclohexane ring in **6d**, analogous to the result obtained with *cis*-menthone analog **2b**, another 5-*exo* product was obtained (Scheme 2). A close look at the NMR spectrum allowed us to determine that the insertion occurred in the *cis* methyl group, affording **8d**. This is the first example in which we unequivocally observed the activation of a methyl C­(sp^3^)–H bond in this reaction. Again, the outcome of this reaction was dictated by the rigid conformation of substrate **2d**.

The structural features required for the newly reported 5-*exo* cycloisomerization reaction were preliminarily determined (Scheme 3). Taking menthone-derived **5a** as a starting point, corresponding isomenthone derivative **5e** showed that the stereochemistry of the methyl group does not significantly influence the reactivity. Then, truncated analogue **2f** was synthesized. As expected, the distal methyl group in terpene derivatives **5a** and **5e** was not crucial for triggering the 5-*exo* reactivity, and product **5f** was obtained in good yield. Similarly, we established that tertiary C­(sp^3^)–H bonds other than the isopropyl one could be engaged in this reactivity (Scheme 3, **5g**).

In conclusion, the use of naturally occurring terpene-derived substrates has enabled us to study how conformational effects in complex substitution patterns, otherwise hardly accessible by synthesis, dictate the reactivity and hence the selectivity of the gold­(I)-catalyzed cycloisomerization of aliphatic 1-bromoalkynes. In addition, a new conformationally driven reactivity has been discovered, and its generality has been preliminarily established. DFT calculations have shown the very favorable nature of this reaction when an appropriate conformation is adopted. In-depth research on synthetic applications and mechanistic aspects of the new 5-*exo* reaction is ongoing. This study will be disclosed in due time.

## Supplementary Material







## Data Availability

The data underlying this study are available in the published article and its .

## Footnotes

a The structure of new product **5a** was unambiguously determined by X-ray diffraction of 2,4-dinitrotosylhydrazone **9**, formed on the corresponding ketone obtained by ozonolysis of **5a**.

## Notes

Experimental procedures and characterization (PDF)
Computational details (PDF)
NMR spectra (PDF)

## Notes

Dedicated to the memory of our colleague Iván Lavandera.
