# Supplementary material for: Terpenes as Naturally Occurring Stereochemical Templates: Conformationally Driven Discovery of Reactivity
Source: Org Lett. 2025 Sep 23;27(39):11071–6. doi: 10.1021/acs.orglett.5c03431 (PMC12501937; doi:10.1021/acs.orglett.5c03431)
Supplement: Supplementary file 1 [file ol5c03431_si_001.pdf]

# Supporting Information
# Terpenes as Naturally Occurring Stereochemical Templates: Conformationally-Driven Discovery of Reactivity

Omar Arto,[a‡] Rubén Miguélez,[a,‡] Hannah Siera,[b] Jan Schulte,[b] Isabel Merino,[c] Gebhard Haberhauer,[b] Pablo Barrio[a*]

[a] Department of Organic and Inorganic Chemistry

Universidad de Oviedo

Julian Clavería 8 33006 Oviedo (Spain)

[b] Institut für Organische Chemie Universität Duisburg-Essen

Universitätstraße 7, 45117 Essen (Germany)

[c] Servicios Científico Técnicos

Universidad de Oviedo

Fernando Bonguera s/n, 30006 Oviedo (Spain)

Correspondence to: barriopablo@uniovi.es

**This PDF file includes:**

Materials and Methods
X-ray data
References

# Table of Contents

**1. EXPERIMENTAL SECTION** .....S3

1.1 MATERIAL AND METHODS .....S3

*1.1.1 Equipment and Instruments* .....S3

*1.1.2 Methods* .....S4

*1.1.3 Chemicals* .....S4

1.2 EXPERIMENTAL PROCEDURES .....S5

1.3 CHARACTERIZATION .....S9

**2. APPENDIX**

*X-ray* .....S43

**3. LITERATURE REFERENCES** .....S45

# 1. *Experimental section*

## 1.1. *Material and Methods*

### 1.1.2. *Equipment and Instruments*

**Nuclear Magnetic Resonance (NMR) spectroscopy:**

NMR spectra were recorded on a Bruker AV 600 spectrometer operating at 600.15 ($^{1}$H), 150.91 MHz ($^{13}$C), using a 5 mm PATXI $^{1}$H/D-$^{13}$C/$^{15}$N inverse probe with a z-gradient coil, or on a Bruker AV 400 spectrometer operating at 400.54 ($^{1}$H) and 100.72 MHz ($^{13}$C), using a 5 mm PABBI 1H/D-BB inverse probe with a z-gradient coil, or on a Bruker AV 300 spectrometer operating at 300.13 ($^{1}$H), 75.46 MHz ($^{13}$C), using a 5 mm QNP $^{1}$H/$^{13}$C/$^{19}$F/$^{31}$P/D probe with a z-gradient coil and equipped with an automatic sample changer. The NMR samples were prepared in $CDCl_3$ and measured at 298K (unless otherwise stated). Data are reported as follows: chemical shift (δ) in parts per million (ppm) relative to tetramethylsilane (TMS), multiplicity (s: singlet, d: doublet, t: triplet, q: quartet, sep: septet, non: nonet, dd: double doublet, dt: double triplet, m: multiplet), coupling constants (J) in Hertz (Hz) and integration. $^{13}$C multiplicities were assigned by DEPT experiments. The residual solvent signals of deuterated solvents were used as internal references. All the experiments were acquired with the TOPSPIN 2.1 Bruker NMR software and the spectra analysis was conducted via the NMR processing softwares TOPSPIN 2.1 or MestReNova v.14.2.1-27684. Assignment of the NMR peaks in the $^{1}$H and $^{13}$C spectra was accomplished with the aid of additional 2D NMR experiments (gsCOSY, gsHSQCEd, gsHMBC, gsTOCSY, gsNOESY) and their selective 1D versions when needed (sel-1D-gsNOESY, sel-1D-gsTOCSY) which were recorded on the AV600 or AV400 spectrometers. The atom numbering of the signal assignment does not correspond to IUPAC rules.

**Mass Spectrometry (MS):**

High resolution mass spectra (HRMS) were measured on a high-resolution mass spectrometer IMPACT II, BRUKER (Servicios Ciefntifico Técnicos, Universidad de Oviedo) with a quadrupole and a Time-Of-Flight (TOF) tube as analyzer, using conventional Electrospray Ion Source (ESI), in full scan mode (4 eV) and positive ion polarity. The equipment employes $N_2$ at the nebulization step (2.4 Bar), and as drying gas (250 ºC, 6.0 L/min). Alternatively, and due to the low polarity of these compounds, some HRMS spectra were determined on an Agilent equipment using APCI(+) ionization (6545 Q-TOF, AGILENT, MS Spectrometer, Servicios Cientifico Técnicos). In some cases, HRMAS could not be obtained, since the molecules failed to ionize under any of these conditions.

**X-Ray Difraction (XRD):**

Monocrystal X-Ray Diffraction was measured using a Oxford Diffraction Xcalibur Nova diffractometer (Servicios Ciefntifico Técnicos, Universidad de Oviedo) with a Nova type X ray source (microfocus and copper radiation optics), kappa geometry goniometer and Onyx typo CCD detector (165 mm).

### 1.1.2. Methods

All reactions discussed as results of this work were carried out using oven dried glassware under an atmosphere of argon (99.999%) using standard Schlenk techniques or young sealed tubes. Glassware was evacuated and further dried by heating with a heat-gun. Electric heating-stirring plates with oil baths were used for reactions at elevated temperatures. For reactions below room temperature, the reaction vessel was cooled using a JULABO FT902-Cryostat. Reaction temperatures refer to the external bath temperature. Cannulas and syringes were used for the transfer of reagents and solvents, which were flooded with inert gas (3×) before use. Purification by column chromatography was performed using manual air pressure on Geduran© Si60 silica gel (40-63 µm) from Merck KGaA. Silica gel F254 TLC plates from Merck KGaA were used for monitoring reactions, analyzing fractions of column chromatography, and measuring Rf values. To visualize the analytes, TLC plates were treated with appropriate staining solutions followed by subsequent heating.

### 1.1.2. Chemicals

Commercial reagents were purchased with the best quality affordable from Sigma Aldrich, TCI, Alfa Aesar and Acros Organics. Solvents purchased in technical grade quality were distilled under reduced pressure and used for purification procedures. and used without further purification unless otherwise stated.1,2-Dichloroethane was distilled from $CaH_2$ and THF from sodium/benzophenone. Other anhydrous solvents were purchased from commercial sources. TLC was performed on aluminum-backed plates coated with silica gel 60, with F245 indicator, and developed with phosphomolybdic acid or potassium permanganate stains. Solvents used in column chromatography were obtained from commercial suppliers and used without further purification.

## *1.2. Experimental Procedures*

### General Procedure A: *Synthesis of aldehydes (Homologation reaction)*[1]

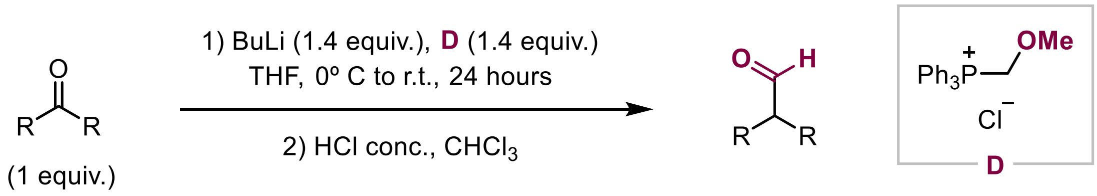


To a round bottom flask with a magnetic stir bar, BuLi (1.4 equiv., 1.6M or 2.5M in hexane) was added dropwise to a solution of methoxymethyltriphenylphosphonium chloride (C) in THF (0.4M) at 0 ºC. The mixture was stirred for 45 minutes, and the corresponding ketone (1 equiv.) was added dropwise at the same temperature. Then, the resulting reaction mixture was stirred for 24 hours at room temperature. After completion of the reaction, the mixture was quenched with HCl 1N at 0 ºC, and it was extracted with diluted with $Et_2O$ three times and washed with brine. The combined organic layers were dried over anhydrous $Na_2SO_4$, filtered, concentrated until almost dryness, and hexane was added to precipitate the solids. The crude product was filtered through a pad of celite. The mixture was concentrated, redissolved in $CHCl_3$, and concentrated aqueous HCl was added at 0 ºC. The mixture was stirred at room temperature; the evolution of the reaction was followed by TLC chromatography (ca. 4 hours). Then, the solvent was removed, diluted with water, and extracted with $Et_2O$ three times. The crude was used without any purification, or the aldehyde was distilled under reduced pressure.

## General Procedure B: *Synthesis of 1,1-dibromoalkenes "Corey-Fuch Reaction"*

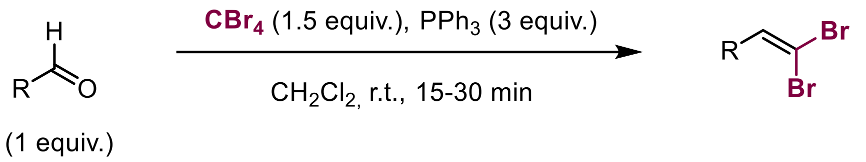


The procedure described in the literature was followed.[2] To a solution of the corresponding aldehyde (1 equiv.) in anhydrous dichloromethane (0.1M), $CBr_4$ (1.5 equiv.) was added followed by $PPh_3$ (3 equiv.). The reaction mixture was stirred for 15-30 minutes and then hexane was added. Solids were filtered through alternate pads of celite /silica gel/ celite. Removal of solvents under vacuum afforded crude 1,1-dibromoalkenes in sufficient purity to be used in the next step without further purification or they were purified by flash column chromatography on silica gel using n-hexane as eluent.

## General Procedure C: *Synthesis of 1-bromoalkynes*

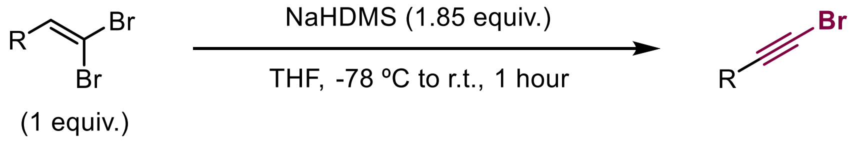


The procedure described in the literature was followed.[2] In a Schlenk flask under argon, NaHMDS (1M in THF, 1.85 equiv.) was added dropwise to a solution of the corresponding dibromoolefin (1 equiv.) in THF (0.4M), at -78 ºC. When the addition was complete, the cooling bath was removed, and the reaction mixture was stirred for one further hour. After this time, the reaction was quenched with $NH_4Cl$ sat. extracted with $Et_2O$ (x3) and the combined organic layers washed with water and brine. The organic layer was dried over $Na_2SO_4$, filtered and the solvent removed in vacuum. The final 1-bromoalkynes were purified by flash column chromatography on silica gel using n-hexane or n-pentane as eluents.

## General Procedure D: *Synthesis of alcohols*

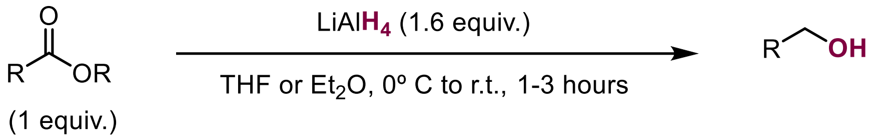


At 0 ºC, a solution of the corresponding carboxylic acid or ester (1 equiv.) in dry THF (1 M) was added dropwise to a suspension of $LiAlH_4$ (1.6 equiv.) in dry THF (0.4 M). After the addition was complete the reaction mixture was stirred for 1-3 hours at room temperature. Then, the reaction was carefully quenched by dropwise addition of water (1mL per gram of LAH), NaOH 10% (1mL per gram of LAH) and water (3mL per gram of LAH) at 0 ºC. Solids were filtered through alternate pads of celite /silica gel/ celite and removing of the solvents by rotary evaporation afforded the crude alcohol that was used in the next step without further purification.

## General Procedure E: *Synthesis of alkyl bromides*[2]

In a Schlenk flask under argon, $PPh_3$ (1.2 equiv.) was added to a solution of the corresponding alcohol (1 equiv.) in anhydrous DCM (0.5M). The mixture was taken to 0 ºC and NBS (1.2 equiv.) was added portion wise. Then, the reaction was taken to room temperature and upon finishing (1-3 hours, monitored by TLC) the solvent was removed under vacuum and the crude reaction mixture purified by means of flash column chromatography on silica gel using n-hexane or n-pentane as eluents affording the corresponding primary bromide.

## General Procedure F: *Synthesis of terminal alkynes*

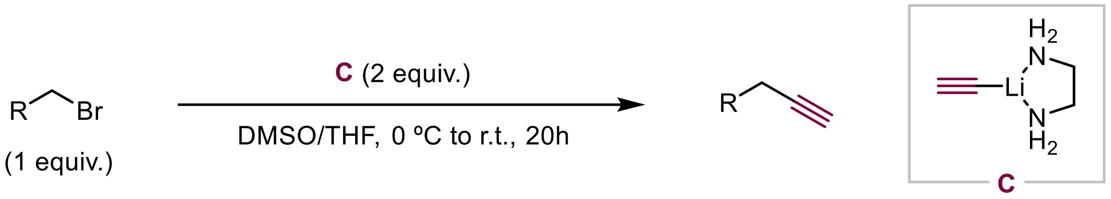


The procedure described in the literature was followed, with a slide modification.[2] Lithium acetylide (C) (2 equiv.) was weighed in a flame dried Schlenk flask under argon. Anhydrous DMSO (1 mL per mmol of C) and THF (0.5 mL per mmol of C) were added, and the reaction mixture was taken to 0 ºC. A solution of the corresponding primary bromide (1 equiv.) in THF (0.5 mL per mmol) was added at once. The reaction mixture was then stirred overnight at room temperature. The day after, the reaction was quenched with saturated $NH_4Cl$ solution, extracted with $Et_2O$ (x3) and washed with brine (x5). The organic layer was dried over $Na_2SO_4$ and filtered by a pad of silica gel using n-hexane as eluent.

## General Procedure G: *Synthesis of 1-bromoalkynes*[2]

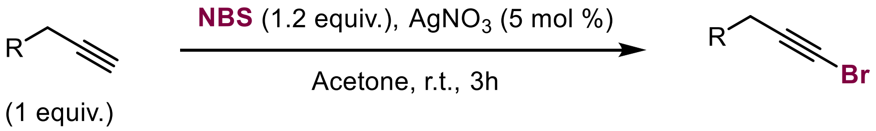


In a Schlenk flask under Ar, NBS (1.2 equiv.) was added to a solution of terminal alkyne (typically 1 mmol, 1 equiv.) in Acetone (0,5M). Then, $AgNO_3$ (5 mol %) was added and the reaction mixture was stirred for 3h in the dark. Acetone was removed in a rotatory evaporator and the crude reaction mixture purified by means of flash column chromatography (typically hexane were used as eluent).

## General Procedure H: *Mesylation of alcohols*[3]

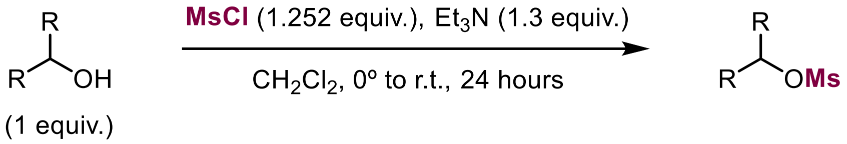


A solution of the corresponding alcohol (1 equiv.) in anhydrous DCM (0.5M), was cooled to 0ºC. At this temperature, MsCl (1.252 equiv.) was added. After stirring for 20 minutes triethylamine (1.3 equiv.) was added dropwise and the reaction was allowed to reach room temperature. The reaction was stirred 24 h and then it was diluted with DCM. The crude reaction mixture was washed with water (two times), saturated aqueous $NaHCO_3$ and

saturated aqueous NaCl. The combined organic layers were dried over $Na_2SO_4$ and concentrated under vacuum. The crude mesylates were used in the following step without further purification.

## General Procedure I: *$S_N2$ reaction. Cyanation*[3]

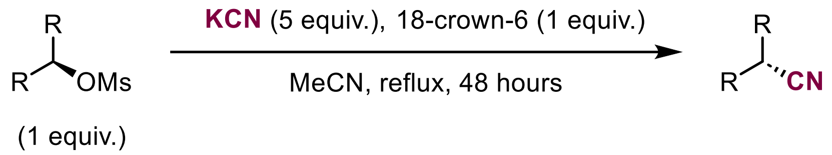


In a round bottom flask with a magnetic stir bar under Ar, the corresponding mesylate (1 equiv.) was dissolved in MeCN (0.3M), followed by the addition of KCN (5 equiv.) and 18-crown-6 (1 equiv.). The resulting reaction mixture was stirred for 48 hours at reflux. After completion of the reaction, the mixture was allowed to reach room temperature and concentrated under vacuum. The resulting mixture was diluted with DCM, washed with water (x3) and brine (x3). The organic layer was dried over $Na_2SO_4$, filtered through alternate pads of celite /silica gel/ celite and the solvent was removed in vacuum. The crude cyanides were obtained in sufficient purity to be used in the next step without further purification or they were purified by flash column chromatography on silica gel using n-hexane/EtOAc mixtures as eluents.

## General Procedure J: *Synthesis of aldehydes (Cyanide reduction)*[4]

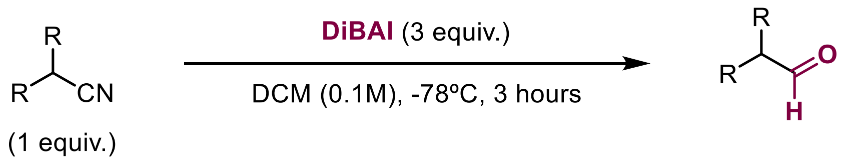


In a Schlenk flask under argon, DiBAl (1.2M in toluene, 2.5 equiv.) was added dropwise to a solution of the corresponding cyanide (1 equiv.) in DCM (0.1M), at -78 ºC. After completion of the reaction an aqueous solution of Rochelle's salt (0.13M) was added dropwise. Upon completion of the addition, the reaction mixture was allowed to warm to room temperature, extracted with DCM (x3) and washed with brine. The combined organic layers were dried over $Na_2SO_4$, filtered and the solvent removed in vacuum. The aldehydes obtained were used in the next step without further purification.

## General Procedure K: *Knoevenagel reaction*[5]

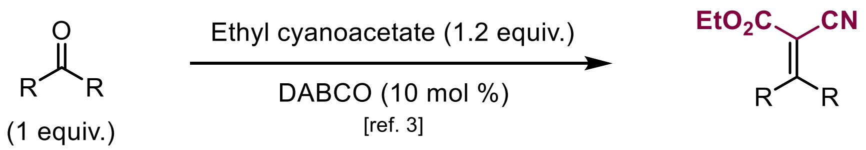


To a round bottom flask with a magnetic stir bar, the corresponding ketone (1 equiv.), ethyl cyanoacetate (1.2 equiv.), and 1,4-diazabicyclo[2.2.2]octane (DABCO, 10 mol%) were added. The resulting reaction mixture was stirred overnight at room temperature. After completion of the reaction the mixture was diluted with EtOAc and washed with water and brine. The combined organic layer was dried over anhydrous $Na_2SO_4$, filtered, and concentrated in vacuo. The crude product was purified by flash column

chromatography using Hexane/EtOAc (10:1 to 5:1) as the eluent to yield the corresponding products.

## General Procedure K': *Knoevenagel reaction*[6]

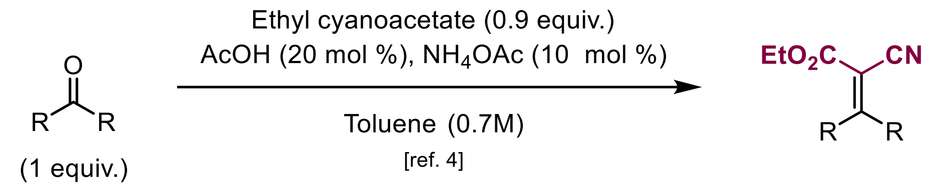


A mixture of ethyl cyanoacetate (0.9 equiv.), the corresponding ketone (1 equiv.), ammonium acetate (10 mol %), and glacial acetic acid (20 mol %) in toluene (0.7M) was refluxed for 4 h using a Dean-Stark water separator. The mixture was washed with water, dried over anhydrous sodium sulfate, and concentrated. The crude product was purified by flash column chromatography using Hexane/EtOAc (10:1 to 5:1) as the eluent to yield the corresponding products.

## General Procedure L: 1,4-addition to α,β-unsaturated compounds[5]

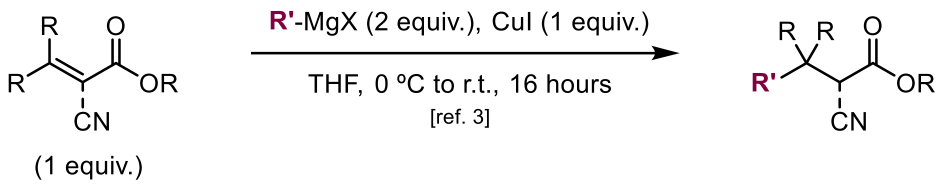


To a suspension of copper iodide (1.0 equiv.) in THF (0.5M) the corresponding alkyl magnesium bromide (2.0 equiv.) in THF was added dropwise at 0 °C. After stirring for 2 h at the same temperature, ethyl 3-methylcrotonate (1.0 equiv.) was added. The reaction mixture was slowly warmed to room temperature and stirred overnight. The reaction was quenched with sat. $NaHCO_3$ (aq.) and filtered through a pad of celite. After removing copper salts from the mixture, the reaction mixture was diluted with EtOAc and washed with water and brine. The combined organic layers were dried over anhydrous $Na_2SO_4$, filtered, and concentrated in vacuo. In most cases, esters synthesized through this procedure were pure enough for the next step.

## General Procedure M: Hydrolysis of cyanoacetate derivatives[5]

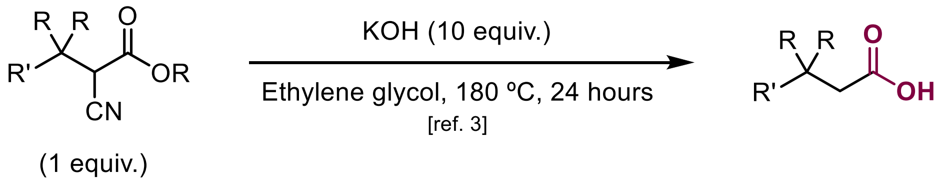


A clean, oven-dried screw cap reaction tube with a magnetic stir–bar was charged with the cyanoacetate derivative (1 equiv.) and KOH (10.0 equiv.), followed by the addition of ethylene glycol (1M). The reaction mixture was sealed tightly and was vigorously stirred for 24 h in a preheated oil bath at 180 °C. After the stipulated time, the reaction mixture was cooled to room temperature and diluted with water. The diluted reaction mixture was acidified with 2N HCl to pH 3 and then extracted with EtOAc three times. The combined organic layers were dried over anhydrous $Na_2SO_4$ and concentrated in vacuo. The crude product was purified through flash column chromatography using Hexane/EtOAc as the eluent to provide corresponding acid.

## General Procedure N: Cycloisomerization of 1-bromoalkynes

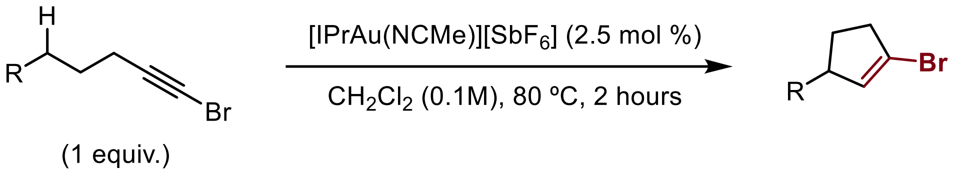


The procedure was followed as described in the literature.[2] [IPrAu(CNMe)][$SbF_6$] (2.5 mol%) was weighted in a flamed pressure Schlenk under Ar and dissolved in the least amount of dry DCM. Then, the 1-bromoalkyne (1 equiv.) was added. Finally, the remaining dry DCM (to achieve a final 0.1M concentration) was added and the reaction mixture was stirred at 80 ºC for 2 hours. After that time, it was cooled down to room temperatureand concentrated on a rotary evaporator under reduced pressure. The crude was purified by flash chromatography to yield the pure compound using hexane or pentane as eluents.

## *Characterization*

### 1.2.1.1. *Menthone family substrates (**1a,b**; **2a,b**)*

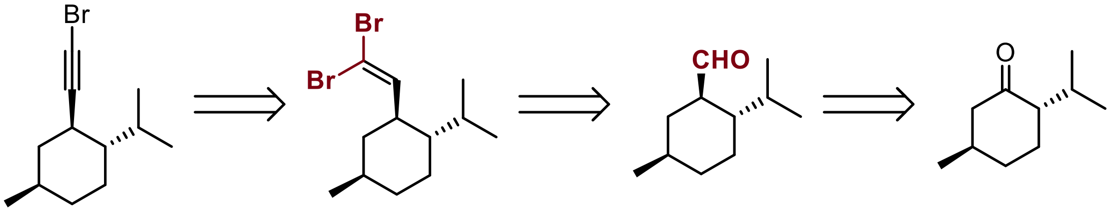


**1a** **1a-CBr$_2$** **1a-CHO** **(L)-Menthone**

***(1R,2S,5R)-2-isopropyl-5-methylcyclohexane-1-carbaldehyde* (*1a-CHO*)**

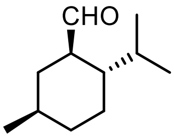


The title compound was synthesized using General Procedure A, starting from 10 mmol (1.5 g) of *(L)-menthone*, the aldehyde ***1a-CHO*** as a slightly green liquid (1.2 g, 7.4 mmol, 74%, dr = 10:1). The crude was purified by distillation (220ºC at 10mmHg). Spectra matches those previously reported. [1]

**$^1$H NMR** (300 MHz, $CDCl_3$) δ 9.48 (d, *J* = 4.8 Hz, 1H), 1.82 – 1.60 (m, 5H), 1.53 (tt, *J* = 11.5, 3.2 Hz, 1H), 1.39 (ttd, *J* = 12.6, 6.4, 3.2 Hz, 1H), 1.13 – 0.94 (m, 3H), 0.94 – 0.81 (m, 9H), 0.79 (d, *J* = 6.9 Hz, 3H).

***(1S,2R,4R)-2-(2,2-dibromovinyl)-1-isopropyl-4-methylcyclohexane* (1a-*CBr$_2$*)**

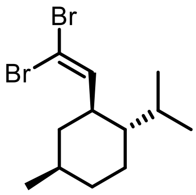


The title compound was synthesized using General Procedure B, starting from 1 mmol (168 mg) of aldehyde ***1a-CHO***, yielding dibromoolefin ***1a-CBr$_2$*** as a colorless liquid (238

mg, 0.7 mmol, 73%, dr = 10:1). The crude was purified by flash column chromatography using n-hexane as eluent.

**$^1$H NMR** (300 MHz, $CDCl_3$) δ 6.14 (d, *J* = 9.7 Hz, 1H), 1.78 – 1.58 (m, 5H), 1.40 (dddd, *J* = 14.8, 11.3, 5.6, 3.2 Hz, 2H), 1.14 – 1.03 (m, 2H), 0.93 – 0.74 (m, 13H).

**$^{13}$C NMR** (75 MHz, $CDCl_3$) δ 143.9 (CH), 87.0 (C), 47.2 (CH), 45.8 (CH), 40.4 ($CH_2$), 34.9 ($CH_2$), 32.3 (CH), 29.3 (CH), 24.3 ($CH_2$), 22.5 ($CH_3$), 21.5 ($CH_3$), 16.5 ($CH_3$).

**HRMS** (GC-Q-TOF) m/z: $[M\text{-}Br]^+$ calcd for $C_{12}H_{20}Br$: 243.0748; found: 243.0744.

### ***(1S,2R,4R)-2-(bromoethynyl)-1-isopropyl-4-methylcyclohexane*** **(*1a*)**

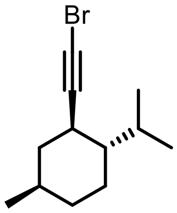


The title compound was synthesized using General Procedure C, starting from 0.7 mmol (238 mg) of dibromoolefin ***1a-CBr₂***, yielding bromoalkyne ***1a*** as a colorless liquid (136 mg, 0.56 mmol, 80%, dr = 10:1). The crude was purified by flash column chromatography using n-hexane as eluent.

**$^1$H NMR** (300 MHz, $CDCl_3$) δ 2.25 – 2.11 (m, 2H), 1.97 (dtd, *J* = 12.8, 3.4, 2.1 Hz, 1H), 1.75 – 1.57 (m, 2H), 1.17 – 1.02 (m, 1H), 0.90 (dd, *J* = 13.6, 6.8 Hz, 9H), 0.77 (d, *J* = 6.9 Hz, 3H).

**$^{13}$C NMR** (75 MHz, $CDCl_3$) δ 83.7 (C), 47.3 (CH), 42.2 ($CH_2$), 38.6 (C), 34.8 ($CH_2$), 34.7 (CH), 32.5 (CH), 28.9 (CH), 24.2 ($CH_2$), 22.3 ($CH_3$), 21.4 ($CH_3$), 15.8 ($CH_3$).

**HRMS** (GC-Q-TOF) m/z: $[M\text{-}CH_3]^+$ calcd for $C_{11}H_{16}Br$: 227.0435; found: 227.0435.

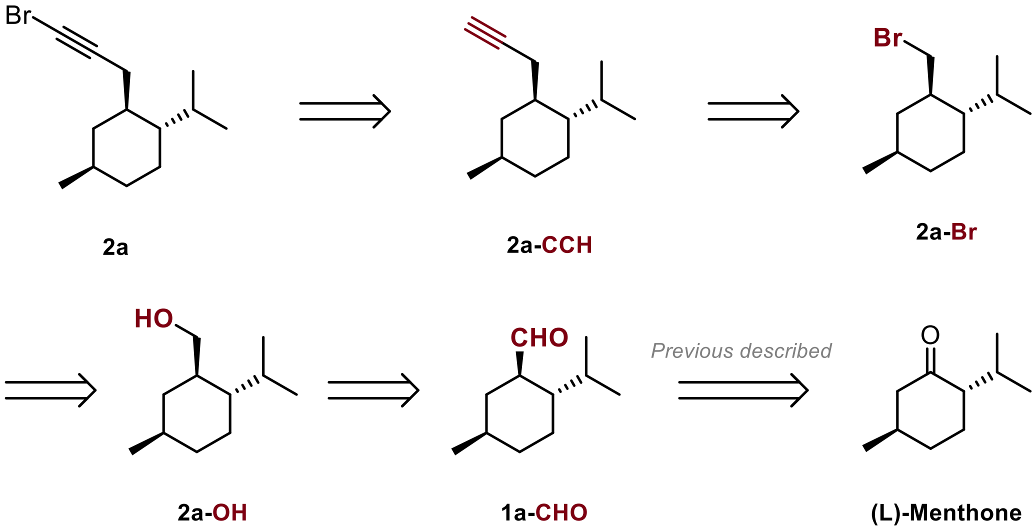


### ***((1R,2S,5R)-2-isopropyl-5-methylcyclohexyl)methanol*** **(*2a-OH*)**

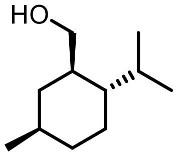


The title compound was synthesized using General Procedure D (using 1 equiv. of $LiAlH_4$), starting from 5 mmol (841 mg) of aldehyde ***1a-CHO***, yielding alcohol ***2a-OH*** as a colorless liquid (852 mg, 5 mmol, 100%). The crude alcohol was used in the next

step without further purification. The $^1$H NMR spectrum matches the one previously reported. [8]
**$^1$H NMR** (300 MHz, $CDCl_3$) δ 3.68 (dd, *J* = 10.7, 3.3 Hz, 1H), 3.53 (dd, *J* = 10.8, 6.3 Hz, 1H), 1.86 – 1.98 (m, 1H), 1.85 – 1.54 (m, 5H), 1.39 (qq, *J* = 11.2, 3.4 Hz, 3H), 1.16 – 0.97 (m, 2H), 0.90 (dd, *J* = 6.7, 2.1 Hz, 6H), 0.76 (d, *J* = 6.9 Hz, 3H).

***(1S,2R,4R)-2-(bromomethyl)-1-isopropyl-4-methylcyclohexane (2a-Br)***

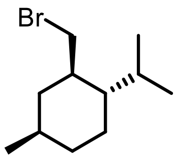


The title compound was synthesized using General Procedure E, starting from 5 mmol (852 mg) of alcohol ***2a-OH***, yielding bromoalkane (***2a-Br***) as a colorless liquid (898 mg, 3.9 mmol, 77%). The crude was purified by flash column chromatography using n-hexane as eluent.
**$^1$H NMR** (300 MHz, $CDCl_3$) δ 3.49 (d, *J* = 3.7 Hz, 2H), 1.94 (dtq, *J* = 10.1, 6.4, 3.1 Hz, 1H), 1.82 – 1.30 (m, 5H), 1.30 – 0.96 (m, 3H), 0.91 (dd, *J* = 6.7, 5.1 Hz, 7H), 0.75 (d, *J* = 6.9 Hz, 3H).
**$^{13}$C NMR** (75 MHz, $CDCl_3$) δ 44.8 (CH), 40.5 ($CH_2$), 40.4 ($CH_2$), 40.3 (CH), 35.1 ($CH_2$), 32.6 (CH), 26.5 (CH), 23.9 ($CH_2$), 22.7 ($CH_3$), 21.5 ($CH_3$), 15.4 ($CH_3$).
**HRMS** (GC-Q-TOF) m/z: $[M]^+$ calcd for $C_{11}H_{21}Br$: 232.0827; found: 232.0825.

***(1S,2S,4R)-1-isopropyl-4-methyl-2-(prop-2-yn-1-yl)cyclohexane* (*2a-CCH*)**

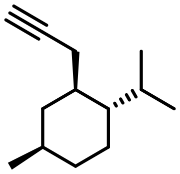

The title compound was synthesized using General Procedure F, starting from 3.9 mmol (898 mg) of bromoalkane ***2a-Br***, yielding alkyne ***2a-CCH*** as a colorless liquid (633 mg, 3.5 mmol, 91%). The crude was purified by flash column chromatography using n-hexane as eluent.
**$^1$H NMR** (300 MHz, $CDCl_3$) δ 2.33 – 2.23 (m, 1H), 2.15 (ddd, *J* = 16.8, 7.0, 2.7 Hz, 1H), 1.95 (t, *J* = 2.8 Hz, 2H), 1.84 – 1.60 (m, 3H), 1.56 (s, 1H), 1.49 – 1.29 (m, 3H), 0.90 (dd, *J* = 6.7, 4.7 Hz, 8H), 0.73 (d, *J* = 6.9 Hz, 3H).
**$^{13}$C NMR** (75 MHz, CDCl3) δ 83.3 (C), 69.4 (CH), 45.7 (CH), 41.5 ($CH_2$), 38.0 (CH), 35.3 ($CH_2$), 32.8 (CH), 26.8 (CH), 24.1 ($CH_2$), 22.7 ($CH_2$), 21.6 ($CH_3$), 15.4 ($CH_3$).
**HRMS** (GC-Q-TOF) m/z: $[M-CH_3]^+$ calcd for $C_{12}H_{19}$: 163.1487; found: 163.1489.

***(1S,2S,4R)-2-(3-bromoprop-2-yn-1-yl)-1-isopropyl-4-methylcyclohexane* (*2a*)**

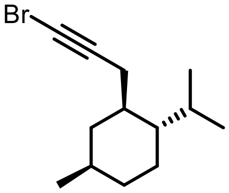


The title compound was synthesized using General Procedure G, starting from 3.5 mmol (633 mg) of alkyne ***2a-CCH***, yielding bromoalkyne ***2a*** as a colorless liquid (738 mg, 2.9 mmol, 82%). The crude was purified by flash column chromatography using n-hexane as eluent.

$^{1}$**H NMR** (300 MHz, $CDCl_3$) δ 2.29 (dd, *J* = 16.8, 3.7 Hz, 1H), 2.15 (dd, *J* = 16.8, 7.1 Hz, 1H), 1.88-1.97 (m, 1H), 1.86 – 1.59 (m, 3H), 1.49 – 1.20 (m, 4H), 0.90 (dd, *J* = 6.6, 3.4 Hz, 8H), 0.72 (d, *J* = 6.9 Hz, 3H).
$^{13}$**C NMR** (75 MHz, CDCl3) δ 79.1 (C), 45.9 (CH), 41.7 ($CH_2$), 38.2 (CH), 35.2 ($CH_2$), 32.8 (CH), 29.9 (C), 26.8 (CH), 24.1 ($CH_2$), 24.0 ($CH_2$), 22.8 ($CH_3$), 21.7 ($CH_3$), 15.4 ($CH_3$).
**HRMS** (GC-Q-TOF) m/z: $[M-CH_3]^+$ calcd for $C_{12}H_{18}Br$: 241.0592; found: 241.0594.

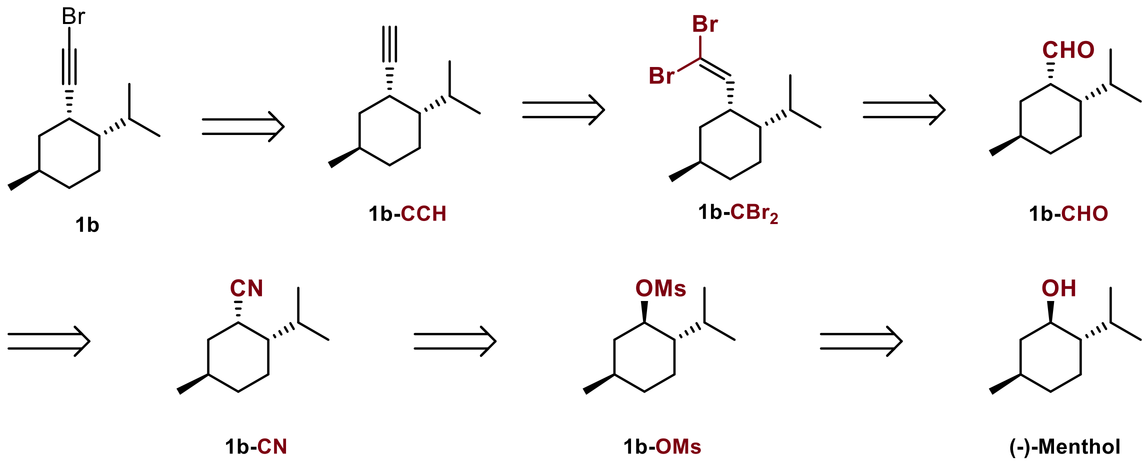


### *(1R,2S,5R)-2-isopropyl-5-methylcyclohexyl methanesulfonate* (*1b-OMs*)

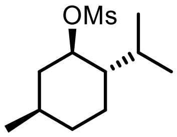


The title compound was synthesized using General Procedure H, starting from 50 mmol (7.81 g) of *(-)-menthol*, yielding mesylate ***1b-OMs*** as a yellow liquid (11.60 g, 49.5 mmol, 99%). The crude mesylate was used in the next step without further purification. The spectral data matches with the literature.[3]
$^{1}$**H NMR** (300 MHz, $CDCl_3$) δ 4.55 (td, *J* = 10.9, 4.6 Hz, 1H), 3.01 (s, 3H), 2.26 (dddd, *J* = 11.9, 5.0, 3.3, 1.9 Hz, 1H), 2.02 – 2.12 (m, 1H), 1.70 (ddq, *J* = 14.2, 8.4, 3.0, 2.5 Hz, 2H), 1.58 (d, *J* = 1.9 Hz, 1H), 1.56 – 1.36 (m, 2H), 1.26 (td, *J* = 12.1, 10.9 Hz, 1H), 1.14 – 0.97 (m, 1H), 0.93 (dd, *J* = 6.7, 2.6 Hz, 7H), 0.83 (d, *J* = 7.0 Hz, 3H).
**HRMS** unstable

### *(1S,2S,5R)-2-isopropyl-5-methylcyclohexane-1-carbonitrile* (*1b-CN*)

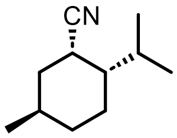


The title compound was synthesized using General Procedure I, starting from 49.5 mmol (11.60 g) of mesylate ***1b-OMs***, yielding cyanide ***1b-CN*** as a colorless liquid (3.88 mg, 23.50 mmol, 47%). The crude was purified by column chromatography using Hexane/EtOAc (10:1 to 5:1) as eluent.
$^{1}$**H NMR** (300 MHz, $CDCl_3$) δ 3.10 – 3.02 (m, 1H), 1.98 (dt, *J* = 13.4, 2.9 Hz, 1H), 1.89 (dtd, *J* = 13.5, 3.4, 1.8 Hz, 1H), 1.75 (dddt, *J* = 21.7, 11.8, 6.5, 3.3 Hz, 2H), 1.64 – 1.54 (m, 1H), 1.37 – 1.20 (m, 1H), 1.14 (ddd, *J* = 13.4, 12.1, 4.2 Hz, 1H), 1.02 (dq, *J* = 10.2, 3.5 Hz, 1H), 0.95 (d, *J* = 6.7 Hz, 7H), 0.92 (d, *J* = 6.4 Hz, 3H).
$^{13}$**C NMR** (75 MHz, $CDCl_3$) δ 121.1 (C), 45.7 (CH), 37.4 ($CH_2$), 34.6 ($CH_2$), 31.3 (CH), 31.2 (CH), 28.6 (CH), 27.0 ($CH_2$), 21.9 ($CH_3$), 20.8 ($CH_3$), 20.7 ($CH_3$).

**HRMS** (GC-Q-TOF) m/z: $[M]^+$ calcd for $C_{11}H_{19}N$: 165.1517; found: 165.1511.

***(1S,2S,5R)-2-isopropyl-5-methylcyclohexane-1-carbaldehyde* (*1b-CHO*)**

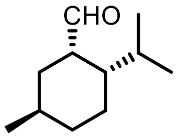


The title compound was synthesized using General Procedure J, starting from 6 mmol (992 mg) of cyanide ***1b-CN***, yielding aldehyde ***1b-CHO*** as a colorless liquid (616 mg, 3.7 mmol, 61%). The crude aldehyde was used in the next step without further purification. Spectral data matches with the literature.[3]

**$^1$H NMR** (300 MHz, $CDCl_3$) δ 9.89 (d, $J$ = 2.0 Hz, 1H), 2.72 (tq, $J$ = 4.4, 2.1 Hz, 1H), 2.05 (dq, $J$ = 13.5, 2.8 Hz, 1H), 1.91 – 1.68 (m, 3H), 1.47 (dddt, $J$ = 12.6, 9.8, 6.6, 3.1 Hz, 1H), 1.38 – 1.21 (m, 2H), 1.18 – 1.01 (m, 2H), 0.92 (dd, $J$ = 6.6, 4.1 Hz, 5H), 0.89 (d, $J$ = 1.8 Hz, 1H), 0.85 (d, $J$ = 6.5 Hz, 3H).

**HRMS** not purified

***(1S,2S,4R)-2-(2,2-dibromovinyl)-1-isopropyl-4-methylcyclohexane* (*1b-CBr₂*)**

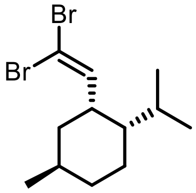


The title compound was synthesized using General Procedure B, starting from 3.7 mmol (616 mg) of aldehyde ***1j-CHO***, yielding dibromoolefin ***1j-CBr$_2$*** as a colorless liquid (759 mg, 2.3 mmol, 64%). The crude was purified by flash column chromatography using n-hexane as eluent.

**$^1$H NMR** (300 MHz, $CDCl_3$) δ 6.59 (d, $J$ = 9.9 Hz, 1H), 2.90 – 2.75 (m, 1H), 1.88 – 1.66 (m, 1H), 1.41 (dddp, $J$ = 15.6, 9.5, 6.4, 3.1 Hz, 1H), 1.31 – 1.19 (m, 2H), 1.16 (dd, $J$ = 4.2, 3.0 Hz, 1H), 1.11 (d, $J$ = 3.9 Hz, 1H), 1.07 (d, $J$ = 3.7 Hz, 2H), 1.00 – 0.93 (m, 1H), 0.86 (td, $J$ = 6.4, 3.9 Hz, 9H).

**$^{13}$C NMR** (75 MHz, $CDCl_3$) δ 139.9 (CH), 87.6 (C), 46.6 (CH), 41.5 (CH), 40.2 ($CH_2$), 35.5 ($CH_2$), 31.1 (CH), 27.8 (CH), 26.1 ($CH_2$), 22.8 ($CH_3$), 21.2 ($CH_3$), 21.1 ($CH_3$).

**HRMS** unstable

***(1S,2S,4R)-2-ethynyl-1-isopropyl-4-methylcyclohexane* (*1b-CCH*)**

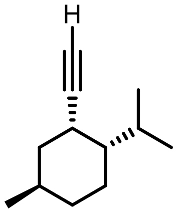


Dibromoolefine ***1b-CBr$_2$*** (144 mg, 0.6 mmol, 1 equiv.) was dissolved in THF (7 mL) in a flame dried Schlenk under Ar. The mixture was cooled to -78ºC and n-BuLi (0.78 mmol, 0.5 mL, 1.6 M solution in THF, 1.3 equiv.) was added dropwise and the reaction mixture was stirred for 2 hours. The reaction was quenched carefully with water extracted with $Et_2O$ (x3) and the combined organic layers washed with water and brine. The organic layer was dried over $Na_2SO_4$, filtered and the solvent removed in vacuum. The crude was filtered through a short pad of silica using n-hexane as eluent to afford alkyne ***1b-CCH*** as a colorless liquid (97 mg, 0.6 mmol, quantitative). The crude alkyne was used in the next step without further purification.

**$^1$H NMR** (300 MHz, $CDCl_3$) δ 3.05 (d, J = 4.3 Hz, 1H), 2.08 – 1.79 (m, 2H), 1.72 (s, 2H), 1.56 – 1.36 (m, 4H), 1.31 – 1.14 (m, 2H), 1.14 – 0.87 (m, 3H), 0.31 (s, 6H).
**HRMS** not purified

***(1S,2S,4R)-2-(bromoethynyl)-1-isopropyl-4-methylcyclohexane* (*1b*)**

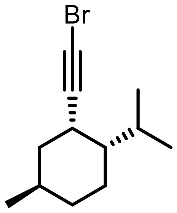


The title compound was synthesized using General Procedure G, starting from 0.6 mmol (97 mg) of alkyne ***1b-CCH***, yielding bromoalkyne ***1b*** as a colorless liquid (71 mg, 0.3 mmol, 49%). The crude was purified by flash column chromatography using n-hexane as eluent.
**$^1$H NMR** (300 MHz, $CDCl_3$) δ 2.91 (dq, *J* = 5.3, 2.0, 1.4 Hz, 1H), 1.83 (dt, *J* = 12.5, 2.8 Hz, 1H), 1.73 (dddd, *J* = 13.9, 6.2, 3.1, 1.6 Hz, 3H), 1.53 (dp, *J* = 9.4, 6.7 Hz, 1H), 1.32 (dd, *J* = 12.6, 3.3 Hz, 2H), 1.05 (ddd, *J* = 12.6, 11.6, 4.1 Hz, 1H), 0.96 – 0.79 (m, 10H).
**$^{13}$C NMR** (75 MHz, $CDCl_3$) δ 82.0 (C), 47.3 (CH), 40.2 ($CH_2$), 39.2 (C), 35.4 ($CH_2$), 32.0 (CH), 31.1 (CH), 27.9 (CH), 26.6 ($CH_2$), 22.3 ($CH_3$), 21.1 ($CH_3$), 20.9 ($CH_3$).
**HRMS** (GC-Q-TOF) m/z: $[M-CH_3]^+$ calcd for $C_{11}H_{16}Br$: 227.0435; found: 227.0436.

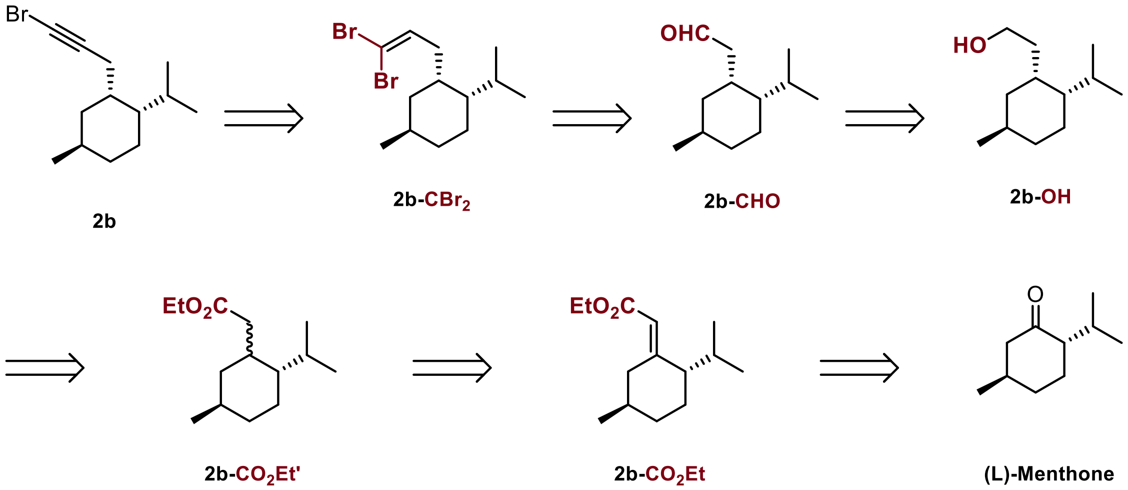


***ethyl (E)-2-((2S,5R)-2-isopropyl-5-methylcyclohexylidene)acetate* (*2b-CO₂Et*)**

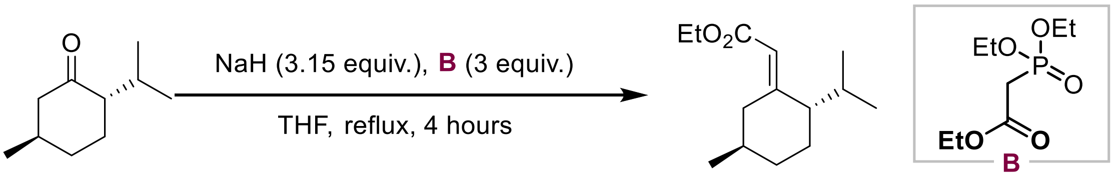


**2b-*CO₂Et***

The procedure was followed as described in the literature, with a slide modification.[9] In a round bottom flask with a magnetic stir bar under Ar, ethyl 2-(diethoxyphosphoryl)acetate B (6.73 g, 3 equiv.) in THF (0.8M) was added dropwise to a suspension of NaH (0,72 g, 3.15 equiv.) in THF (2,46M). The mixture was stirred for 30 minutes. Then *(L)-menthone* (1.54 g, 10 mmol, 1 equiv.) in THF (0.33M) was added dropwise at 0 °C. The reaction mixture was stirred for 4 hours at reflux. After completion of the reaction, water was added to the mixture after it was allowed to reach room temperature. The crude reaction mixture was extracted with $Et_2O$ (x3) and washed with

brine. The combined organic layers were dried over $Na_2SO_4$, filtered and the solvent removed in vacuum. The crude mixture was purified by flash column chromatography on silica gel using Hexane/EtOAc (40:1 to 10:1) as eluents to afford the corresponding conjugated ester ***2b-CO₂Et*** as a colorless liquid (1.00 g, 4.5 mmol, 45%). Spectral data match those previously reported. [10]

**$^1$H NMR** (300 MHz, $CDCl_3$,) δ 5.66 – 5.55 (m, 1H), 4.12 (q, *J* = 7.1 Hz, 2H), 2.37 – 2.23 (m, 1H), 2.07 – 1.90 (m, 2H), 1.90 – 1.73 (m, 2H), 1.70 (dd, *J* = 7.6, 3.9 Hz, 1H), 1.57 – 1.43 (m, 1H), 1.43 (s, 2H), 1.25 (t, *J* = 7.1 Hz, 3H), 1.21 – 1.08 (m, 1H), 1.02 – 0.80 (m, 9H), 0.76 (dd, *J* = 10.5, 6.6 Hz, 1H).

**$^{13}$C NMR** (75 MHz, $CDCl_3$, ***major***) δ 167.2 (C), 164.9 (C), 113.4 (CH), 59.5 ($CH_2$), 52.7 (CH), 36.2 ($CH_2$), 33,8 ($CH_2$), 31.8 ($CH_2$), 27.7 ($CH_2$), 26.9 (CH), 22.0 ($CH_3$), 20.6 ($CH_3$), 19.7 ($CH_3$), 14.4 ($CH_3$).

### *ethyl 2-((2S,5R)-2-isopropyl-5-methylcyclohexyl)acetate* (*2b-$CO_2$Et’*)

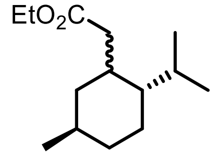


The procedure described in the literature was followed, with a slight modification.[11] A round bottom flask with a magnetic stir bar under Ar was charged with $Cu(OAc)_2$ (5% mol, 0.04 mmol, 7.3 mg), *rac*-BINAP (6% mol, 0.048 mmol, 31 mg), *t*-BuONa (15% mol, 0.12 mmol, 11.5 mg). After evacuating to vacuum and filling back with Ar (x3), the mixture was dissolved in toluene (2.4 mL) and stirred for 20 minutes. Then PMHS (3 equiv., 2.4 mmol, 0.57 mL) was added and a mixture of the conjugated ester (***2b-CO₂Et***) (179 mg, 0.8 mmol, 1 equiv.), *t*-BuOH (3 equiv., 2.4 mmol, 0.23 mL) and toluene (0.8 mL) was subsequently added dropwise. The mixture was stirred for 36 hours. Upon completion of the reaction, $NH_4F$ (sat.) was added to the mixture and stirred for 30 minutes. The crude reaction mixture was extracted with EtOAc (x3) and washed with brine. The combined organic layers were dried over $Na_2SO_4$, filtered and the solvent removed in vacuum. The crude was filtered by a pad of silica and used in the next step without further purification.

**HRMS** not purified

### *2-((1R,2S,5R)-2-isopropyl-5-methylcyclohexyl)ethan-1-ol* (*2b-OH*)

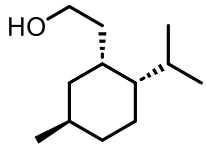


The title compound was synthesized using General Procedure D, starting from crude ester ***2b-CO₂Et’***, yielding alcohol ***2b-OH*** as a colorless liquid (310 mg, 1.68 mmol, 42% over two steps). The crude was purified by column chromatography using Hexane/EtOAc (20:1 to 5:1) as eluent.

**$^1$H NMR** (300 MHz, $CDCl_3$,) δ 3.78 – 3.52 (m, 2H), 1.99 – 1.86 (m, 1H), 1.67 (ddt, *J* = 11.1, 6.1, 2.9 Hz, 3H), 1.53 (tdd, *J* = 9.1, 7.7, 4.7 Hz, 4H), 1.42 – 1.31 (m, 1H), 1.03 (ddd, *J* = 24.7, 12.8, 3.4 Hz, 3H), 0.87 (t, *J* = 6.4 Hz, 7H), 0.82 (d, *J* = 6.4 Hz, 3H).**$^{13}$C NMR** (75 MHz, $CDCl_3$) δ 62.1 ($CH_2$), 48.2 (CH), 38.7 ($CH_2$), 36.0 ($CH_2$), 31.7 (CH), 29.4 (CH), 28.5 ($CH_2$), 26.3 (CH), 25.4 ($CH_2$), 22.9 ($CH_3$), 21.8 ($CH_3$), 20.8 ($CH_3$).

**HRMS** (GC-Q-TOF) m/z: $[M]^+$ calcd for $C_{12}H_{24}O$: 184.1827; found: 184.1822.

***2-((1R,2S,5R)-2-isopropyl-5-methylcyclohexyl)acetaldehyde* (*2b-CHO*)**

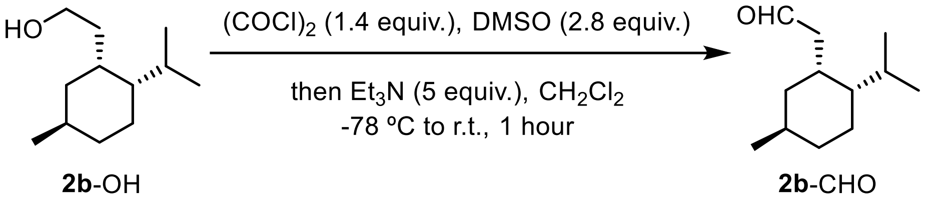


The procedure described in the literature was followed.[2] In a Schlenk flask under Ar, DMSO (2.8 equiv.) was added dropwise to a solution of oxalyl chloride (1.4 equiv.) in dry DCM (0.5M) at -78 °C. Then, alcohol ***2b-OH*** (92 mg, 0.5 mmol, 1 equiv.) dissolved in dry DCM (1M) was added dropwise to the reaction mixture at the same temperature. After stirring for 15 minutes triethylamine (5 equiv.) was added dropwise and the reaction was allowed to reach room temperature. Then, water was added, and the crude reaction mixture was extracted with $Et_2O$ (x3), the combined organic layers were washed with 1N HCl, saturated aqueous $NaHCO_3$ and saturated aqueous NaCl. The combined organic layers were dried over $Na_2SO_4$ and concentrated under vacuum affording aldehyde ***2b-CHO*** as a colorless liquid (61 mg, 0.33 mmol). The crude aldehyde was used in the following step without further purification.

**$^1$H NMR** (300 MHz, $CDCl_3$,) δ 9.74 (d, $J$ = 3.4 Hz, 1H), 2.02 – 1.86 (m, 1H), 1.79 – 1.58 (m, 5H), 1.56 (d, $J$ = 3.0 Hz, 2H), 1.35 (t, $J$ = 7.2 Hz, 2H), 1.25 (ddd, $J$ = 13.0, 9.0, 6.5 Hz, 1H), 1.15 – 0.93 (m, 4H), 0.92 – 0.84 (m, 9H), 0.84 – 0.78 (m, 6H).

**$^{13}$C NMR** (75 MHz, $CDCl_3$) δ 203.6 (CH), 47.3 (CH), 41.3 ($CH_2$), 39.5 ($CH_2$), 35.5 ($CH_2$), 30.1 (CH), 29.6 (CH), 26.1 (CH), 25.4 ($CH_2$), 22.7 ($CH_3$), 21.5 ($CH_3$), 20.7 ($CH_3$).

**HRMS** not purified

***(1S,2R,4R)-2-(3,3-dibromoallyl)-1-isopropyl-4-methylcyclohexane* (*2b-CBr₂*)**

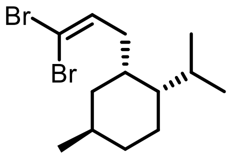


The title compound was synthesized using General Procedure B, starting from 0.33 mmol (61 mg) of aldehyde ***2b-CHO***, yielding dibromoolefin ***2b-CBr$_2$*** as a colorless liquid (44 mg, 0.17 mmol, 34% over two steps). The crude was purified by flash column chromatography using n-hexane as eluent.

**$^1$H NMR** (400 MHz, $CDCl_3$,) δ 6.38 (dd, $J$ = 8.2, 6.4 Hz, 1H), 2.14 (ddd, $J$ = 15.1, 11.8, 8.2 Hz, 1H), 2.06 – 1.95 (m, 2H), 1.77 – 1.68 (m, 2H), 1.67 – 1.61 (m, 1H), 1.61 – 1.49 (m, 1H), 1.38 (dp, $J$ = 9.5, 6.5 Hz, 1H), 1.13 – 0.94 (m, 4H), 0.94 – 0.82 (m, 9H).

**$^{13}$C NMR** (101 MHz, $CDCl_3$) δ 139.2 (CH), 88.4 (C), 47.9 (CH), 38.7 ($CH_2$), 35.8 ($CH_2$), 34.9 (CH), 29.9 ($CH_2$), 29.6 (CH), 26.5 (CH), 25.3 ($CH_2$), 22.8 ($CH_3$), 21.8 ($CH_3$), 20.8 ($CH_3$).

**HRMS** unstable

***(1S,2R,4R)-2-(3-bromoprop-2-yn-1-yl)-1-isopropyl-4-methylcyclohexane* (*2b*)**

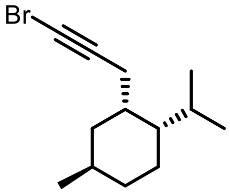


The title compound was synthesized using General Procedure C, starting from 0.17 mmol (44 mg) of dibromoolefin ***2b-CBr$_2$***, yielding bromoalkyne ***2b*** as a colorless liquid (26 mg,

0.1 mmol, 64%). The crude was purified by flash column chromatography using n-hexane as eluent.

**$^1$H NMR** (400 MHz, $CDCl_3$,) δ 2.22 – 2.15 (m, 2H), 2.15 – 2.08 (m, 1H), 2.01 (dd, *J* = 13.4, 2.9 Hz, 1H), 1.76 – 1.65 (m, 2H), 1.48 (dtq, *J* = 12.6, 6.4, 3.1 Hz, 1H), 1.29 (ddd, *J* = 10.0, 7.8, 5.0 Hz, 1H), 1.05 – 0.94 (m, 3H), 0.91 – 0.85 (m, 9H), 0.85 – 0.68 (m, 1H).

**$^{13}$C NMR** (101 MHz, $CDCl_3$) δ 80.5 (C), 47.3 (CH), 38.8 ($CH_2$), 37.7 (C), 35.8 ($CH_2$), 35.1 (CH), 29.6 (CH), 26.1 (CH), 25.0 ($CH_2$), 22.9 ($CH_3$), 21.7 ($CH_3$), 20.8 ($CH_3$), 17.2 ($CH_2$).

**HRMS** (GC-Q-TOF) m/z: $[M]^+$ calcd for $C_{13}H_{21}Br$: 256.0827; found: 256.0823.

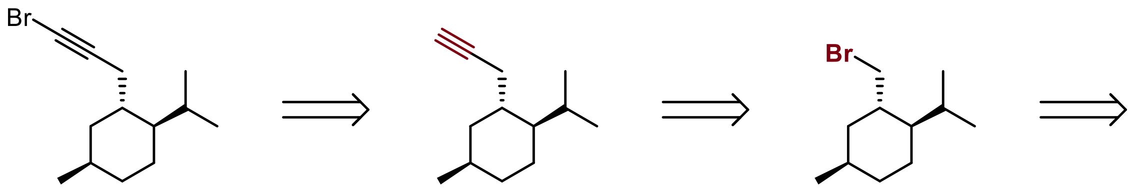


**2e** **2e-CCH** **2e-Br**

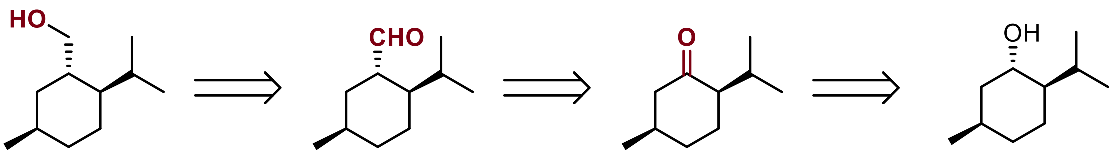


**2e-OH** **2e-CHO** **2e-CO** ***1R*-(+)-Isomenthol**

***(2R,5R)-2-isopropyl-5-methylcyclohexan-1-one (2e-CO)***

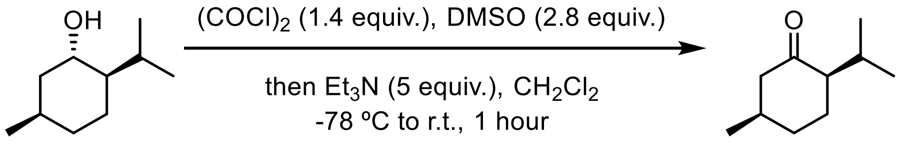


**2e**-CO

The procedure described in the literature was followed.[2] In a Schlenk flask under Ar, DMSO (2.8 equiv.) was added dropwise to a solution of oxalyl chloride (1.4 equiv.) in dry DCM (0.5M) at -78 °C. Then, *(1R)-(+)-isomenthol* (1.56 g, 10 mmol, 1 equiv.) dissolved in dry DCM (1M) was added dropwise to the reaction mixture at the same temperature. After stirring for 15 minutes triethylamine (5 equiv.) was added dropwise and the reaction was allowed to reach room temperature. Then, water was added, and the crude reaction mixture was extracted with $Et_2O$ (x3), the combined organic layers were washed with 1N HCl, saturated aqueous $NaHCO_3$ and saturated aqueous NaCl. The combined organic layers were dried over $Na_2SO_4$ and concentrated under vacuum affording aldehyde ***2e-CO*** as a colorless liquid (61 mg, 0.33 mmol). The crude ketone was used in the following step without further purification.

**$^1$H NMR** (300 MHz, $CDCl_3$) δ = 2.36 – 2.24 (m, 1H), 2.17 – 2.05 (m, 1H), 2.05 – 1.88 (m, 4H), 1.79 – 1.59 (m, 3H), 1.55 – 1.37 (m, 2H), 0.98 (d, *J* = 6.5 Hz, 3H), 0.93 (d, *J* = 6.3 Hz, 4H), 0.84 (d, *J* = 6.4 Hz, 3H).

**HRMS** not purified

***(1S,2R,5R)-2-isopropyl-5-methylcyclohexane-1-carbaldehyde (2e-CHO)***

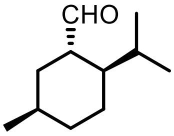


The title compound was synthesized using General Procedure A, starting from 10 mmol (1.54 g) of isomenthone (***2e-CO***), yielding a mixture of isomenthone (***2e-CO***), menthone, and the corresponding aldehyde (***2e-CHO***). This mixture was taken forward in the synthesis and the purification was carried out along the following steps.

**$^1$H NMR** (300 MHz, $CDCl_3$) δ = 2.36 – 2.21 (m, 2H), 2.16 (s, 9H), 1.50 – 1.25 (m, 4H), 1.17 (t, *J* = 7.0 Hz, 1H), 1.08 – 0.90 (m, 5H), 0.90 – 0.68 (m, 11H).

**$^{13}$C NMR** (75 MHz, $CDCl_3$) δ = 206.36, 76.74, 65.94, 57.29, 48.09, 34.51, 29.47, 26.97, 21.55, 21.26, 20.98, 19.98.

**HRMS** not purified

***((1S,2R,5R)-2-isopropyl-5-methylcyclohexyl)methanol (2e-OH)***

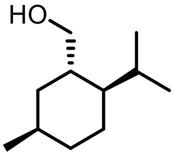


The title compound was synthesized using General Procedure D (using 1 equiv. of $LiAlH_4$), starting from crude aldehyde ***2e-CHO***, yielding alcohol ***2e-OH*** as a colorless liquid (334 mg, 2.0 mmol, 20% yield for three steps from ketone ***2e-CO***). The crude was purified by column chromatography using hexane/EtOAc (10:1) as eluent.
**$^1$H NMR** (300 MHz, $CDCl_3$) δ = 3.61 (t, *J* = 4.8 Hz, 2H), 2.16 (td, *J* = 7.1, 2.6 Hz, 1H), 2.02 – 1.88 (m, 2H), 1.88 – 1.71 (m, 3H), 1.71 – 1.52 (m, 2H), 1.41 (dt, *J* = 8.1, 5.2 Hz, 3H), 1.27 – 1.00 (m, 4H), 0.94 – 0.87 (m, 3H), 0.82 (dt, *J* = 11.9, 3.3 Hz, 6H). **HRMS** not purified

***(1R,2S,4R)-2-(bromomethyl)-1-isopropyl-4-methylcyclohexane (2e-Br)***

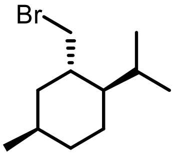


The title compound was synthesized using General Procedure E, starting from 2.0 mmol (334 mg) of alcohol ***2e-OH***, yielding bromoalkane ***2e-Br*** as a colorless liquid (247 mg, 1.1 mmol, 54%). The crude was purified by flash column chromatography using n-hexane as eluent.
**$^1$H NMR** (300 MHz, $CDCl_3$) δ = 3.51 (qd, *J* = 9.9, 5.6 Hz, 2H), 1.94 – 1.83 (m, 2H), 1.81 – 1.69 (m, 2H), 1.60 (ddd, *J* = 12.7, 7.8, 4.4 Hz, 2H), 1.54 (s, 2H), 1.40 (dd, *J* = 10.0, 5.5 Hz, 2H), 1.33 – 1.18 (m, 1H), 1.13 – 1.00 (m, 1H), 0.91 (ddt, *J* = 7.0, 4.9, 2.3 Hz, 6H), 0.84 (d, *J* = 6.8 Hz, 3H).

**$^{13}$C NMR** (75 MHz, $CDCl_3$) δ = 49.4 ($CH_2$), 44.5 (CH), 40.2 (CH), 36.2 ($CH_2$), 35.8 (CH), 31.0 (CH), 27.1 ($CH_2$), 26.8 ($CH_3$), 21.4 ($CH_3$), 20.3 ($CH_2$), 18.0 ($CH_3$).

**HRMS** unstable

***(1R,2R,4R)-1-isopropyl-4-methyl-2-(prop-2-yn-1-yl)cyclohexane (2e-CCH)***

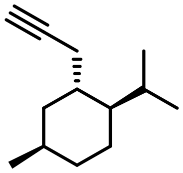

The title compound was synthesized using General Procedure F, starting from 1.1 mmol (247 mg) of bromoalkane ***2e-Br***, yielding alkyne ***2e-CCH*** as a colorless liquid (53 mg, 0.3 mmol, 28%). The crude was purified by flash column chromatography using n-hexane as eluent.
**$^{1}$H NMR** (300 MHz, $CDCl_3$) δ 2.22 (dd, $J$ = 6.3, 2.7 Hz, 2H), 1.94 (t, $J$ = 2.7 Hz, 1H), 1.82 (dp, $J$ = 19.2, 6.6 Hz, 3H), 1.47 (ddd, $J$ = 9.4, 8.0, 3.5 Hz, 2H), 1.43 – 1.24 (m, 4H), 1.12 (dq, $J$ = 12.6, 6.7, 6.0 Hz, 1H), 0.91 (dd, $J$ = 6.9, 2.3 Hz, 6H), 0.81 (d, $J$ = 6.8 Hz, 3H).
**$^{13}$C NMR** (75 MHz, $CDCl_3$) δ = 83.7 (C), 69.3 (CH), 45.4 (CH), 36.6 ($CH_2$), 33.3 (CH), 31.2 ($CH_2$), 27.3 (CH), 26.9 ($CH_2$), 21.6 ($CH_3$), 20.1 ($CH_3$), 19.9 ($CH_2$), 17.8 ($CH_3$).
**HRMS** non-ionizable

***(1R,2R,4R)-2-(3-bromoprop-2-yn-1-yl)-1-isopropyl-4-methylcyclohexane*** **(*2e*)**

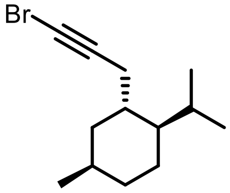


The title compound was synthesized using General Procedure G, starting from 0.3 mmol (53 mg) of alkyne ***2e-CCH***, yielding bromoalkyne ***2e*** as a colorless liquid (54 mg, 0.21 mmol, 71%). The crude was purified by flash column chromatography using n-hexane as eluent.
**$^{1}$H NMR** (300 MHz, $CDCl_3$) δ = 2.27 (d, J = 6.3 Hz, 2H), 1.95 – 1.72 (m, 4H), 1.41 (dddd, J = 27.5, 20.3, 10.9, 5.1 Hz, 7H), 1.19 – 1.03 (m, 1H), 0.94 (dd, J = 6.8, 2.4 Hz, 9H), 0.84 (d, J = 6.8 Hz, 3H).
**$^{13}$C NMR** (75 MHz, $CDCl_3$) δ 79.6 (C), 45.6 (CH), 38.2 (C), 36.7 ($CH_2$), 33.5 (C), 31.2 ($CH_2$), 27.3 (CH), 24.3 ($CH_2$), 21.6 ($CH_3$), 20.1 ($CH_3$), 19.9 ($CH_2$), 17.8 ($CH_3$).
**HRMS** (GC-Q-TOF) m/z: $[M]^+$ calcd for $C_{13}H_{21}Br$: 256.0827; found: 256.0825.

### 1.2.1.2. *Carvone family substrates (**1c,d**; **2c,d**)*

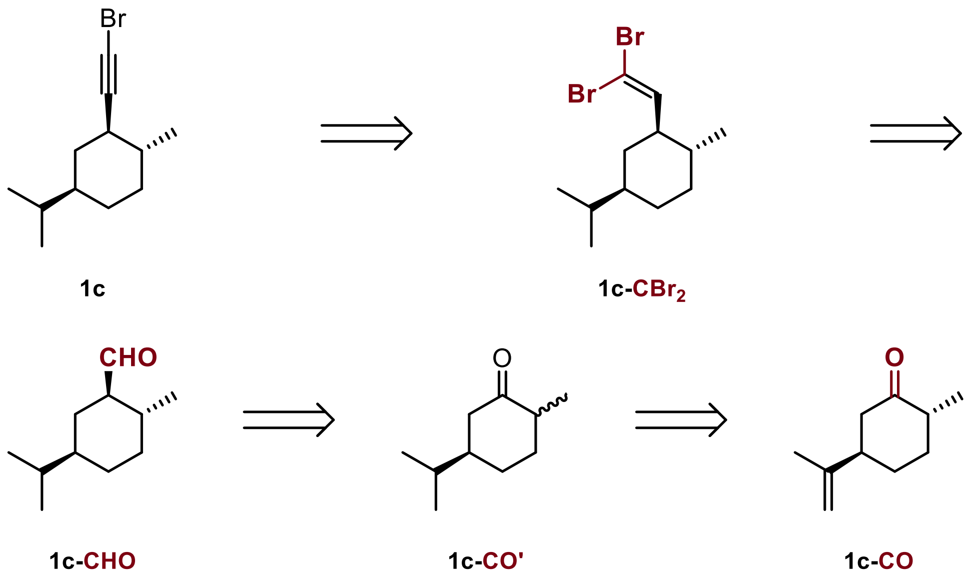


***(2R,5R)-2-methyl-5-(prop-1-en-2-yl)cyclohexan-1-one (1c-C=C)***

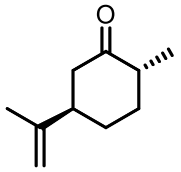


The title compound was obtained after an extensive silica gel chromatography of the commercially available mixture of isomers *(+)-dihydrocarvone*, starting from a 4:1 (trans:cis) mixture, to afford the title compound (***1k-C=C***) diasteromerically pure (dr > 60:1). Spectrla data match those previously reported. [12]

**$^1$H NMR** (300 MHz, $CDCl_3$) δ = 4.85 – 4.66 (m, 2H), 2.49 – 2.34 (m, 2H), 2.34 – 2.22 (m, 2H), 2.19 – 2.06 (m, 1H), 1.99 – 1.88 (m, 1H), 1.79 – 1.72 (m, 3H), 1.68 – 1.55 (m, 1H), 1.37 (qd, *J* = 13.0, 3.5 Hz, 1H), 1.03 (d, *J* = 6.5 Hz, 3H).

***(5R)-5-isopropyl-2-methylcyclohexan-1-one (1c-CO)***

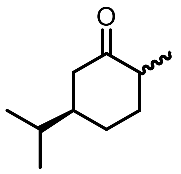


A round bottom flask with a magnetic stir bar and Pd/C (10 w/w%) was evacuated to vacuum and filled with Ar (x3). ***1c-C=C*** (761 mg, 5 mmol, 1 equiv.) diluted in AcOEt (0.6 M) was added and hydrogen was bubbled for 15 minutes. The reaction mixture was maintained on a hydrogen atmosphere and stirred overnight at room temperature. The day after, the solids were filtered through a pad of celite. Removal of solvents under vacuum afforded ketone ***1c-CO*** as a slightly green liquid (454 mg, 3.0 mmol, 59%, dr = 3:1), in sufficient purity to be used in the next step without further purification.

**$^{1}$H NMR** (300 MHz, $CDCl_3$) δ = 2.47 – 2.36 (m, 1H), 2.36 – 2.22 (m, 2H), 2.11 – 1.95 (m, 2H), 1.82 (dt, *J* = 12.2, 2.6 Hz, 1H), 1.25 (dd, *J* = 12.7, 3.3 Hz, 6H), 1.05 (d, *J* = 7.0 Hz, 1H), 0.97 (d, *J* = 6.5 Hz, 3H), 0.86 (ddd, *J* = 6.6, 3.7, 1.8 Hz, 8H).

**$^{13}$C NMR** (75 MHz, Chloroform-d) δ 215.3 (C), 213.8 (C, ***major***), 46.7 (CH, ***major***), 45.4 ($CH_2$, ***major***), 45.0 (CH, ***major***), 44.8 (CH), 44.4 (CH), 43.0 ($CH_2$), 35.2 ($CH_2$, ***major***), 32.8 (CH, ***major***), 31.4 ($CH_2$), 30.7 (CH), 29.0 ($CH_2$, ***major***), 25.0 ($CH_2$), 20.1 ($CH_3$), 20.0 ($CH_3$), 19.7 ($CH_3$, ***major***), 19.4 ($CH_3$, ***major***), 16.0 ($CH_3$), 14.4 ($CH_3$, ***major***).

**HRMS** not purified

***(1R,2R,5R)-5-isopropyl-2-methylcyclohexane-1-carbaldehyde (1c-CHO)***

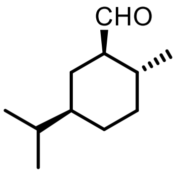


The title compound was synthesized using General Procedure A, starting from 2.1 mmol (343 g) of ketone ***1c-CO'***, yielding aldehyde ***1c-CHO*** as a slightly green liquid (100 mg, 0.6 mmol, 29%). The crude aldehyde was use without further purification.

**$^{1}$H NMR** (300 MHz, $CDCl_3$) δ = 9.54 (d, *J* = 3.8 Hz, 1H), 1.94 – 1.83 (m, 1H), 1.83 – 1.65 (m, 3H), 1.56 (tdd, *J* = 11.9, 6.9, 5.0 Hz, 1H), 1.45 (td, *J* = 6.8, 5.0 Hz, 1H), 1.14 – 1.04 (m, 1H), 1.04 – 0.95 (m, 3H), 0.91 (d, *J* = 6.5 Hz, 3H), 0.85 (d, *J* = 6.8 Hz, 6H).

**$^{13}$C NMR** (75 MHz, $CDCl_3$) δ = 205.5 (CH), 57.7 (CH), 42.7 (CH), 34.6 ($CH_2$), 32.8 (CH), 32.2 (CH), 29.5 ($CH_2$), 29.1 ($CH_2$), 20.4 ($CH_3$), 19.8 ($CH_3$ x2).

**HRMS** not purified

***(1R,2R,4R)-2-(2,2-dibromovinyl)-4-isopropyl-1-methylcyclohexane* (*1c-CBr₂*)**

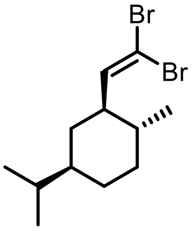


The title compound was synthesized using General Procedure B, starting from 0.6 mmol (100 mg) of aldehyde ***1c-CHO***, yielding dibromoolefin ***1c-CBr$_2$*** as a colorless liquid (133 mg, 0.4 mmol, 69%, dr > 20:1). The crude was purified by flash column chromatography using n-hexane as eluent.

**$^{1}$H NMR** (300 MHz, $CDCl_3$) δ = 6.19 (d, *J* = 9.5 Hz, 1H), 2.00 (dtd, *J* = 11.7, 9.8, 3.6 Hz, 1H), 1.84 – 1.55 (m, 4H), 1.46 (ddd, *J* = 13.8, 12.3, 6.8 Hz, 2H), 1.32 – 0.94 (m, 5H), 0.94 – 0.68 (m, 12H).

**$^{13}$C NMR** (75 MHz, $CDCl_3$) δ = 143.8 (CH), 87.6 (C), 49.8 (CH), 43.3 (CH), 37.1 (CH), 35.0 ($CH_2$), 34.7 ($CH_2$), 32.9 (CH), 29.3 ($CH_2$), 20.4 ($CH_3$), 19.9 ($CH_3$), 19.8 ($CH_3$).

**HRMS** unstable

***(1R,2R,4R)-2-(bromoethynyl)-4-isopropyl-1-methylcyclohexane* (*1c*)**

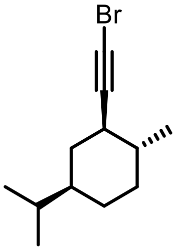

The title compound was synthesized using General Procedure C, starting from 0.38 mmol (124 mg) of dibromoolefin ***1c-CBr₂***, yielding bromoalkyne ***1c*** as a colorless liquid (83 mg, 0.34 mmol, 89%, dr > 20:1). The crude was purified by flash column chromatography using n-hexane as eluent.

**$^{1}$H NMR** (300 MHz, $CDCl_3$) δ 2.00 – 1.90 (m, 1H), 1.85 (dd, *J* = 11.0, 3.6 Hz, 1H), 1.80 – 1.69 (m, 1H), 1.69 – 1.60 (m, 1H), 1.52 – 1.37 (m, 2H), 1.30 (ddt, J = 17.2, 10.6, 3.1 Hz, 1H), 1.22 – 1.06 (m, 1H), 1.03 (d, *J* = 6.5 Hz, 5H), 0.85 (d, *J* = 6.8 Hz, 6H).

**$^{13}$C NMR** (75 MHz, $CDCl_3$) δ 84.2 (C), 43.5 (CH), 38.7 (CH), 38.4 (C), 37.7 (CH), 36.4 ($CH_2$), 34.8 ($CH_2$), 32.7 (CH), 29.3 ($CH_2$), 21.0 ($CH_3$), 19.9 ($CH_3$), 19.8 ($CH_3$).

**HRMS** (GC-Q-TOF) m/z: $[M-CH_3]^+$ calcd for $C_{11}H_{16}Br$: 227.0430; found: 227.0434.

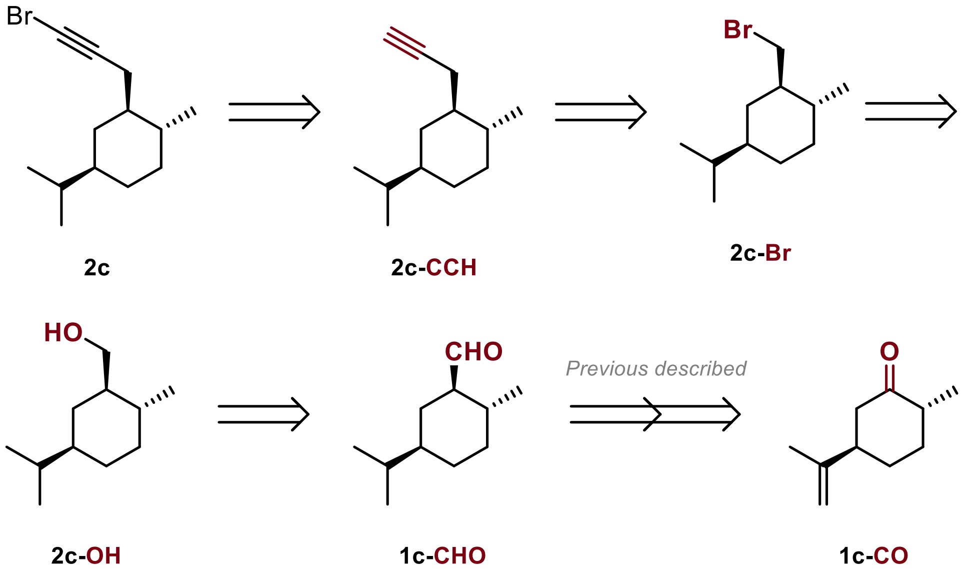


***((1R,2R,5R)-5-isopropyl-2-methylcyclohexyl)methanol (2c-OH)***

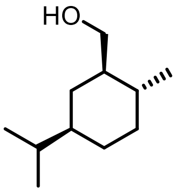

The title compound was synthesized using General Procedure D (using 1 equiv. of $LiAlH_4$), starting from 1.8 mmol (299 mg) of aldehyde ***1c-CHO***, yielding alcohol ***1c-OH*** as a colorless liquid (215 mg, 1.3 mmol, 71%). The crude alcohol was used in the next step without further purification.

**$^{1}$H NMR** (300 MHz, $CDCl_3$) δ = 3.75 – 3.63 (m, 2H), 3.49 (dd, *J* = 10.7, 6.1 Hz, 1H), 1.84 – 1.76 (m, 1H), 1.73 (p, *J* = 2.8 Hz, 1H), 1.64 (dddt, *J* = 16.6, 8.3, 5.5, 2.9 Hz, 3H),

1.34 – 1.46 (m, $J$ = 6.8, 5.2 Hz, 2H), 1.19 – 1.01 (m, 3H), 1.01 – 0.91 (m, 2H), 0.88 (d, $J$ = 6.1 Hz, 3H), 0.83 (d, $J$ = 2.0 Hz, 4H), 0.81 (d, $J$ = 1.9 Hz, 3H).
**$^{13}$C NMR** (75 MHz, $CDCl_3$) δ = 66.2 ($CH_2$), 46.7 (CH), 43.9 (CH), 36.0 ($CH_2$), 33.7 (CH), 33.2 ($CH_2$), 33.1 (CH), 29.7 ($CH_2$), 20.0 ($CH_3$ x2), 19.9 ($CH_2$).
**HRMS** not purified

***(1R,2R,4R)-2-(bromomethyl)-4-isopropyl-1-methylcyclohexane (2c-Br)***

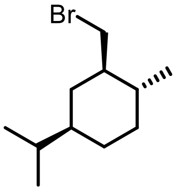


The title compound was synthesized using General Procedure E, starting from 1.3 mmol (215 mg) of alcohol ***2c-OH***, yielding bromoalkane ***2c-Br*** as a colorless liquid (191 mg, 0.82 mmol, 65%). The crude was purified by flash column chromatography using n-hexane as eluent.
**$^{1}$H NMR** (300 MHz, $CDCl_3$) δ = 3.62 – 3.42 (m, 2H), 1.81 – 1.73 (m, 1H), 1.73 – 1.62 (m, 2H), 1.39 – 1.48 (m, 1H), 1.33 – 1.07 (m, 4H), 1.07 – 0.98 (m, 2H), 0.91 (d, $J$ = 6.0 Hz, 3H), 0.88 (d, $J$ = 2.6 Hz, 3H), 0.86 (d, $J$ = 2.6 Hz, 3H).
**$^{13}$C NMR** (75 MHz, $CDCl_3$) δ = 45.6 (CH), 43.9 (CH), 40.6 ($CH_2$), 35.6 ($CH_2$), 35.2 (CH), 34.8 ($CH_2$), 33.0 (CH), 29.6 ($CH_2$), 20.0 ($CH_3$), 19.7 ($CH_3$), 19.6 ($CH_3$).
**HRMS** unstable

***(1R,2S,4R)-4-isopropyl-1-methyl-2-(prop-2-yn-1-yl)cyclohexane (2c-CCH)***

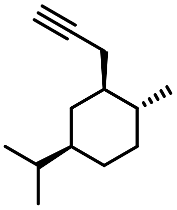

The title compound was synthesized using General Procedure F, starting from 0.78 mmol (183 mg) of bromoalkane ***2c-Br***, yielding alkyne ***2c-CCH*** as a colorless liquid (95 mg, 0.53 mmol, 68%). The crude was purified by flash column chromatography using n-hexane as eluent.
**$^{1}$H NMR** (300 MHz, $CDCl_3$) δ = 2.32 (dt, $J$ = 16.8, 3.0 Hz, 1H), 2.16 (ddd, $J$ = 16.8, 6.9, 2.7 Hz, 1H), 1.94 (t, $J$ = 2.7 Hz, 1H), 1.81 (dq, $J$ = 12.4, 2.8 Hz, 1H), 1.68 (tq, $J$ = 10.8, 2.5, 1.9 Hz, 2H), 1.37 – 1.47 (m, 1H), 1.28 – 1.03 (m, 4H), 0.90 (d, $J$ = 6.1 Hz, 3H), 0.86 (dd, $J$ = 6.8, 3.2 Hz, 8H).
**$^{13}$C NMR** (75 MHz, $CDCl_3$) δ = 83.4 (C), 69.3 (CH), 44.2 (CH), 43.1 (CH), 36.1 (CH), 35.8 ($CH_2$), 35.6 ($CH_2$), 33.0 (CH), 29.6 ($CH_2$), 23.4 ($CH_2$), 20.1 ($CH_3$), 19.9 ($CH_3$), 19.9 ($CH_3$).
**HRMS** non-ionizable

***(1R,2S,4R)-2-(3-bromoprop-2-yn-1-yl)-4-isopropyl-1-methylcyclohexane (2c)***

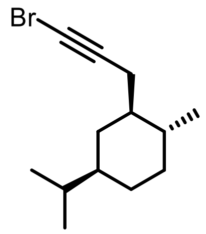


The title compound was synthesized using General Procedure G, starting from 0.53 mmol (95 mg) of alkyne ***2c-CCH***, yielding bromoalkyne ***2c*** as a colorless liquid (91 mg, 0.36 mmol, 67%). The crude was purified by flash column chromatography using n-hexane as eluent.

**$^{1}$H NMR** (300 MHz, $CDCl_3$) δ = 2.34 (dd, $J$ = 16.8, 3.2 Hz, 1H), 2.18 (dd, $J$ = 16.8, 6.7 Hz, 1H), 1.72 (dddd, $J$ = 24.1, 11.5, 5.7, 3.0 Hz, 3H), 1.38 – 1.47 (m, 1H), 1.10 (dtt, $J$ = 17.4, 7.1, 3.3 Hz, 3H), 1.03 – 0.94 (m, 2H), 0.92 – 0.83 (m, 10H).

**$^{13}$C NMR** (75 MHz, $CDCl_3$) δ = 77.6 (C), 44.2 (CH), 43.2 (CH), 38.2 (C), 36.2 (CH), 35.8 ($CH_2$), 35.8 ($CH_2$), 33.0 (CH), 29.6 ($CH_2$), 24.7 ($CH_2$), 20.1 ($CH_3$), 19.9 ($CH_3$), 19.8 ($CH_3$).

**HRMS** (GC-Q-TOF) m/z: $[M-CH_3]^+$ calcd for $C_{12}H_{18}Br$: 241.0586; found: 241.0590.

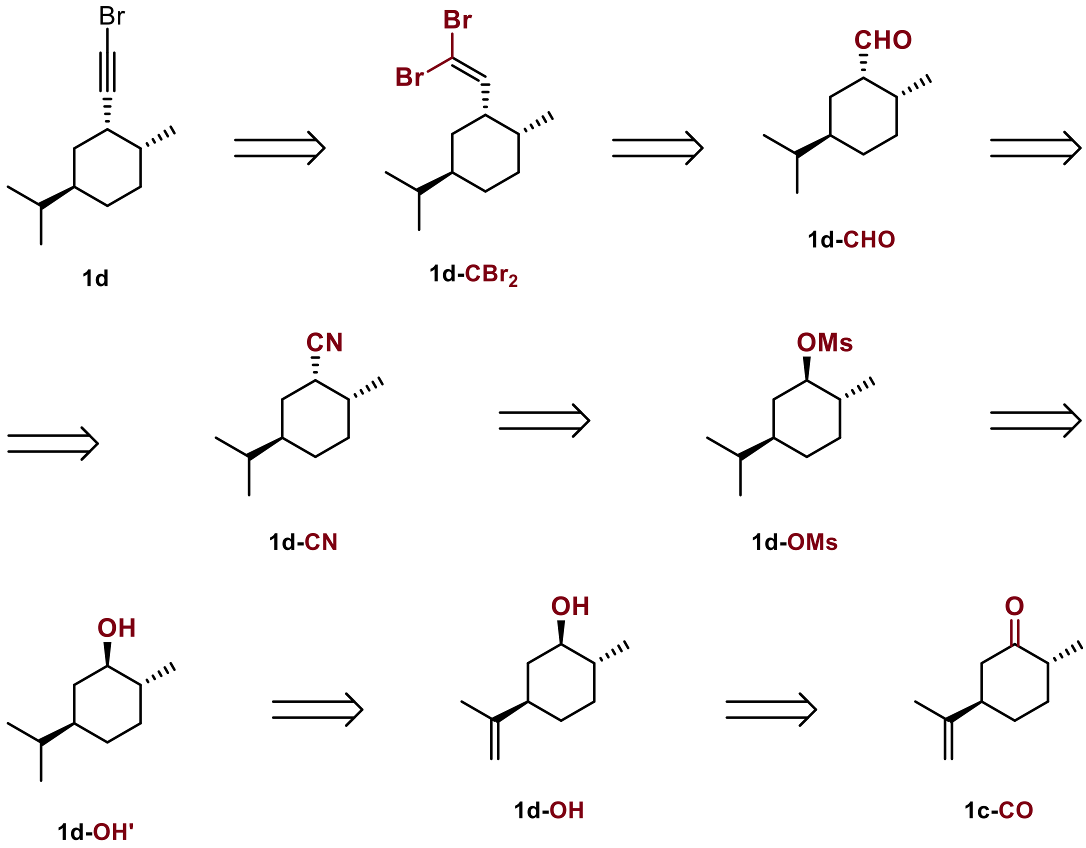


***(1R,2R,5R)-2-methyl-5-(prop-1-en-2-yl)cyclohexan-1-ol (1d-C=C)***

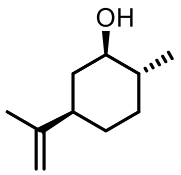

The procedure described in the literature was followed.[13] In a flamed Schlenk, under Argon, a suspension of $LiAlH_4$ (0.5 equiv., 949 mg, 25 mmol) in THF (0.6M, 42 mL) was cooled to -78 ºC. Then a solution of *(2R,5R)-2-methyl-5-(prop-1-en-2-yl)cyclohexan-1-one* (1 equiv., 50 mmol, 7.61 g) in THF (2.4 M, 21 mL) was added over 10 minutes. The reaction was stirred for another 5 minutes and quenched with $NH_4Cl$ (sat.) very carefully. The reaction mixture was extracted with $Et_2O$ (x3) and washed with brine. The combined organic layers were dried over $Na_2SO_4$, filtered and the solvent removed in vacuum. The crude (mixture of diastereoisomers) was purified by column chromatography using hexane/EtOAc (10:1 to 7:1) as eluent to afford the pure olefin (***1d-C=C***) as a slightly green liquid (5.04 g, 32.7 mmol, 65%). Spectral data match those previously reported. [13]

**$^1$H NMR** (300 MHz, $CDCl_3$) δ 4.67 (s, 2H), 3.16 (td, *J* = 10.2, 4.0 Hz, 1H), 2.09 – 1.88 (m, 3H), 1.84 – 1.60 (m, 6H), 1.01 (d, *J* = 6.3 Hz, 6H).

**$^{13}$C NMR** (75 MHz, $CDCl_3$) δ 149.5 (C), 108.7 ($CH_2$), 76.4 (CH), 44.2 (CH), 40.7 ($CH_2$), 40.1 (CH), 33.4 ($CH_2$), 31.2 ($CH_2$), 21.0 ($CH_3$), 18.5 ($CH_3$).

**(1R,2R,5R)-5-isopropyl-2-methylcyclohexan-1-ol (1d-OH)**

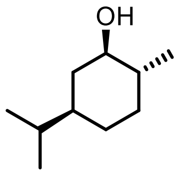


Using a slight modification of the procedure in the literature[14] the corresponding olefin ***1d-C=C*** (2.31 g, 15 mmol, 1 equiv.) was dissolved in MeOH (0.67 M). Then $PtO_2$ was added (10% w/w). The glass container was placed in a high-pressure hydrogenation apparatus. The vessel was slowly pressurized to 5 bar with hydrogen gas, then allowed to vent; and this process was repeated three times. After repressurization to 5 bar, the mixture was stirred overnight at room temperature. After this time, the vessel was slowly depressurized and opened. The mixture was filtered over a pad of celite, dissolved in $Et_2O$ and washed with water to remove MeOH, affording after concentration in vacuum ***1d-OH'*** as a colorless liquid (2.35 g, 15 mmol, quantitative). The crude product was used without any purification.

**$^1$H NMR** (300 MHz, $CDCl_3$) δ 3.18 – 3.00 (m, 1H), 1.94 (ddd, *J* = 10.6, 5.0, 2.8 Hz, 1H), 1.71 (dt, *J* = 9.9, 3.7 Hz, 1H), 1.60 (dq, *J* = 9.4, 4.5, 3.8 Hz, 2H), 1.39 – 1.48 (m, 1H), 1.30 – 1.08 (m, 3H), 0.92 (dd, *J* = 41.5, 6.5 Hz, 14H).

**$^{13}$C NMR** (75 MHz, $CDCl_3$) δ 77.6 (CH), 43.2 (CH), 40.4 (CH), 39.0 ($CH_2$), 33.4 ($CH_2$), 32.7 (CH), 29.2 ($CH_2$), 20.0 ($CH_3$), 19.9 ($CH_3$), 18.5 ($CH_3$).

**HRMS** (GC-Q-TOF) m/z: $[M+H]^+$ calcd for $C_{10}H_{21}O$: 157.1587; found: 157.1585.

***(1R,2R,5R)-5-isopropyl-2-methylcyclohexyl methanesulfonate* (1d-OMs)**

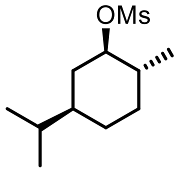


The title compound was synthesized using General Procedure H, starting from 25 mmol (3.9 g) of alcohol ***1d-OH'***, yielding mesylate ***1d-OMs*** as a yellow liquid (5.6 g, 23.8 mmol, 95%). The crude mesylate was used in the next step without further purification.

**$^{1}$H NMR** (300 MHz, $CDCl_3$) δ 4.22 (td, *J* = 10.4, 5.0 Hz, 1H), 2.98 (d, *J* = 1.0 Hz, 3H), 2.17 (ddt, *J* = 10.6, 4.3, 1.9 Hz, 1H), 1.91 – 1.75 (m, 1H), 1.69 – 1.41 (m, 3H), 1.38 – 1.14 (m, 2H), 1.00 (dd, *J* = 6.5, 1.2 Hz, 5H), 0.84 (dt, *J* = 6.8, 1.5 Hz, 6H).
**$^{13}$C NMR** (75 MHz, $CDCl_3$) δ 88.4 (CH), 42.9 (CH), 38.8 ($CH_3$), 37.8 (CH), 36.9 ($CH_2$), 33.1 ($CH_2$), 32.4 (CH), 28.2 ($CH_2$), 19.8 ($CH_3$), 19.6 ($CH_3$), 18.6 ($CH_3$).
**HRMS** not purified

***(1S,2R,5R)-5-isopropyl-2-methylcyclohexane-1-carbonitrile* (*1d-CN*)**

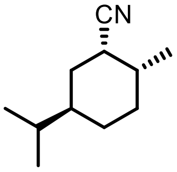


The title compound was synthesized using General Procedure I, starting from 23.8 mmol (5.6 g) of mesylate ***1d-OMs***, yielding cyanide ***1d-CN*** as a colorless liquid (2.1 g, 12.7 mmol, 53%). The crude was purified by column chromatography using hexane/EtOAc (10:1 to 5:1) as eluent.
**$^{1}$H NMR** (300 MHz, $CDCl_3$) δ 2.92 – 2.82 (m, 1H), 1.97 (dq, *J* = 12.9, 2.6 Hz, 1H), 1.75 (dt, *J* = 12.9, 3.0 Hz, 1H), 1.70 – 1.60 (m, 1H), 1.60 – 1.50 (m, 1H), 1.50 – 1.36 (m, 2H), 1.36 – 1.23 (m, 2H), 1.06 (d, *J* = 6.6 Hz, 3H), 1.03 – 0.93 (m, 1H), 0.86 (dd, *J* = 6.5, 1.9 Hz, 6H).
**$^{13}$C NMR** (75 MHz, $CDCl_3$) δ 120.9 (C), 39.2 (CH), 35.2 (CH), 34.0 (CH), 32.4 ($CH_2$), 32.3 (CH), 30.9 ($CH_2$), 28.8 ($CH_2$), 20.3 ($CH_3$), 19.8 ($CH_3$), 19.5 ($CH_3$).
**HRMS** (GC-Q-TOF) m/z: $[M]^+$ calcd for $C_{11}H_{19}N$: 165.1517; found: 165.1521.

***(1S,2R,5R)-5-isopropyl-2-methylcyclohexane-1-carbaldehyde* (*1d-CHO*)**

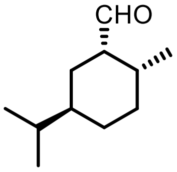


The title compound was synthesized using General Procedure J, starting from 9 mmol (1.49 g) of nitrile ***1d-CN***, yielding aldehyde ***1d-CHO*** as a colorless liquid (1.25 g, 7.46 mmol, 83%). The crude aldehyde was used in the next step without further purification.
**$^{1}$H NMR** (300 MHz, $CDCl_3$) δ 9.88 (d, *J* = 1.8 Hz, 1H), 2.61 – 2.39 (m, 5H), 2.05 (tddd, *J* = 12.2, 9.5, 7.4, 3.5 Hz, 4H), 1.85 (ddd, *J* = 10.1, 5.0, 2.7 Hz, 6H), 1.82 – 1.50 (m), 1.45 – 1.19 (m), 1.11 (d, *J* = 7.1 Hz, 16H), 0.97 – 0.68 (m).
**HRMS** not purified

***(1R,2S,4R)-2-(2,2-dibromovinyl)-4-isopropyl-1-methylcyclohexane* (*1d-CBr$_2$*)**

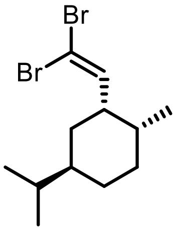

The title compound was synthesized using General Procedure B, starting from 7.72 mmol (1.3 g) of aldehyde ***1d-CHO***, yielding dibromoolefin (***1d-CBr***$_2$) as a colorless liquid (401 mg, 1.24 mmol, 16%, dr > 20:1). The crude was purified by flash column chromatography using n-hexane as eluent.

**$^1$H NMR** (300 MHz, $CDCl_3$) δ 6.56 (d, *J* = 10.0 Hz, 1H), 2.75 – 2.62 (m, 1H), 1.72 (dt, *J* = 12.5, 2.7 Hz, 3H), 1.58 (ddt, *J* = 17.5, 9.5, 3.6 Hz, 2H), 1.46 – 1.34 (m, 1H), 1.24 (dd, *J* = 12.5, 4.0 Hz, 2H), 1.20 – 1.08 (m, 2H), 1.02 (td, *J* = 11.8, 3.3 Hz, 1H), 0.84 (dd, *J* = 6.7, 2.9 Hz, 11H).

**$^{13}$C NMR** (75 MHz, $CDCl_3$) δ 139.4 (CH), 88.1 (C), 44.6 (CH), 38.5 (CH), 35.2 (CH), 34.5 ($CH_2$), 32.9 (CH), 30.5 ($CH_2$), 29.4 ($CH_2$), 20.1 ($CH_3$), 19.9 ($CH_3$), 19.6 ($CH_3$).

**HRMS** unstable

***(1R,2S,4R)-2-(bromoethynyl)-4-isopropyl-1-methylcyclohexane* (*1d*)**

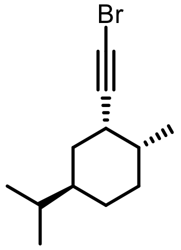


The title compound was synthesized using General Procedure C (stirring the reaction 1 hour 30 minutes at room temperature instead of 1 hour), starting from 1.24 mmol (401 mg) of dibromoolefin ***1l-CBr***$_2$, yielding bromoalkyne ***1l*** as a colorless liquid (228 mg, 0.94 mmol, 76%, dr > 20:1). The crude was purified by flash column chromatography using n-hexane as eluent.

**$^1$H NMR** (300 MHz, $CDCl_3$) δ 2.78 – 2.65 (m, 1H), 1.83 (dq, *J* = 12.7, 2.7 Hz, 1H), 1.68 (dt, *J* = 12.8, 3.0 Hz, 1H), 1.53 – 1.36 (m, 4H), 1.32 (dd, *J* = 12.3, 3.4 Hz, 1H), 1.17 (td, *J* = 12.3, 4.1 Hz, 1H), 0.97 (d, *J* = 6.2 Hz, 3H), 0.86 (dd, *J* = 6.5, 3.3 Hz, 7H).

**$^{13}$C NMR** (75 MHz, $CDCl_3$) δ 81.8 (C), 39.5 (C), 38.5 (CH), 35.8 (CH), 35.4 (CH), 34.9 ($CH_2$), 32.6 (CH), 30.5 ($CH_2$), 29.3 ($CH_2$), 20.5 ($CH_3$), 20.0 ($CH_3$), 19.7 ($CH_3$).

**HRMS** (GC-Q-TOF) m/z: $[M-CH_3]^+$ calcd for $C_{11}H_{16}Br$: 227.0430; found: 227.0435.

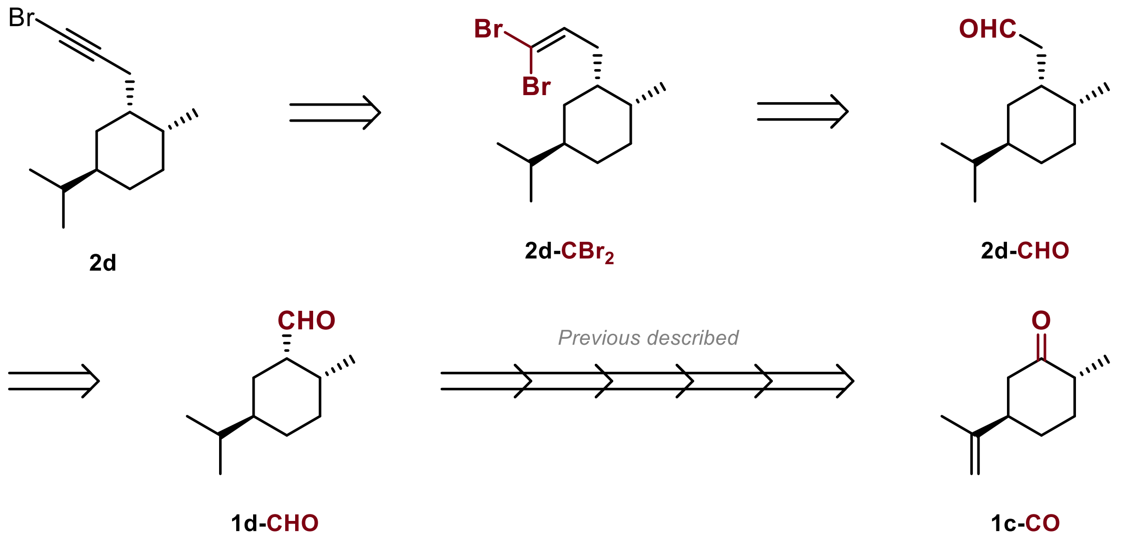


***2-((1R,2R,5R)-5-isopropyl-2-methylcyclohexyl)acetaldehyde* (*2d-CHO*)**

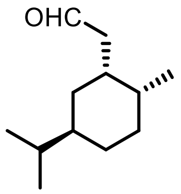


The procedure described in the literature was followed, with a slight modification.[7] In a flamed Schlenk containing *(methoxymethyl)triphenylphosphonium chloride* (4 equiv., 8.06 g) in THF (0.4M) was added $^{t}$BuOK (4 equiv., 2.64 g) portion wise at 0 ºC. This mixture was stirred at room temperature for 15 minutes. Then, it was taken to 0 ºC again, and aldehyde ***1d-CHO*** (1 equiv., 989 mg, 5.9 mmol) dissolved in THF (0.3M) was added. The reaction was stirred at room temperature for 30 minutes. Then, it was quenched with brine and extracted with $Et_2O$ three times. The crude was filtered by a pad of celite using HxH:$Et_2O$ (10:1) as eluent. The intermediate was dissolved in $CHCl_3$ (0.05M) and treated with p-TsOH (10 mol%) overnight at room temperature. Then, the solvent was removed, diluted with water and extracted with $Et_2O$ three times. The crude product was purified by flash column chromatography using hexane/ $Et_2O$ (40:1 to 20:1) as the eluent to yield aldehyde ***2d-CHO*** as a colorless liquid (44 mg, 0.24 mmol, 4%).

**$^{1}$H NMR** (300 MHz, $CDCl_3$) δ 9.74 (dd, *J* = 2.7, 1.6 Hz, 1H), 2.40 – 2.29 (m, 2H), 1.72 – 1.61 (m, 2H), 1.56 (dq, *J* = 12.8, 2.2 Hz, 1H), 1.48 (q, *J* = 3.8 Hz, 1H), 1.40 – 1.29 (m, 1H), 1.22 (tdd, *J* = 12.2, 3.2, 1.4 Hz, 1H), 1.16 – 1.06 (m, 2H), 1.05 – 0.92 (m, 2H), 0.85 – 0.76 (m, 9H).

**$^{13}$C NMR** (75 MHz, $CDCl_3$) δ 203.6 (CH), 41.9 ($CH_2$), 37.4 (CH), 34.9 (CH), 34.5 ($CH_2$), 33.7 (CH), 32.5 (CH), 29.6 ($CH_2$), 29.5 ($CH_2$), 20.0 ($CH_3$), 19.8 ($CH_3$), 19.7 ($CH_2$).

**HRMS** (GC-Q-TOF) m/z: $[M]^+$ calcd for $C_{12}H_{22}O$: 182.1671; found: 182.1673.

***(1R,2R,4R)-2-(3,3-dibromoallyl)-4-isopropyl-1-methylcyclohexane* (*2d-CBr₂*)**

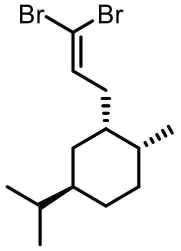


The title compound was synthesized using General Procedure B, starting from 0.24 mmol (44 mg) of aldehyde ***2d-CHO***, yielding dibromoolefin ***2d-$CBr_2$*** as a colorless liquid (57 mg, 0.17 mmol, 70%, dr = 3:1). The crude was purified by flash column chromatography using n-hexane as eluent.

**$^{1}$H NMR** (300 MHz, $CDCl_3$) δ 6.36 (dd, *J* = 8.0, 6.8 Hz, 1H), 2.08 (d, *J* = 7.6 Hz, 2H), 1.81 (dq, *J* = 9.3, 5.3, 4.6 Hz, 1H), 1.65 (ddt, *J* = 12.8, 10.3, 2.8 Hz, 5H), 1.51 – 1.42 (m, 2H), 1.41 – 1.33 (m, 1H), 1.30 – 1.16 (m, 3H), 1.11 (dt, *J* = 12.5, 3.3 Hz, 2H), 1.07 – 0.95 (m, 3H), 0.87 (dd, *J* = 11.0, 6.9 Hz, 15H).

**$^{13}$C NMR** (75 MHz, $CDCl_3$) δ 139.3 (CH), 88.2 (C), 38.6 (CH), 37.4 (CH), 35.4 (CH), 33.3 ($CH_2$), 32.6 (CH), 30.3 ($CH_2$), 29.8 ($CH_2$), 29.6 ($CH_2$), 20.0 ($CH_3$), 19.6 ($CH_3$).

**HRMS** unstable

***(1R,2R,4R)-2-(3-bromoprop-2-yn-1-yl)-4-isopropyl-1-methylcyclohexane* (*2d*)**

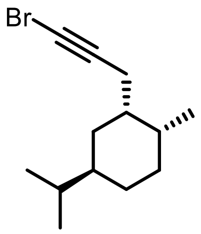

The title compound was synthesized using General Procedure R, starting from 0.15 mmol (50 mg) of dibromoolefin ***2d-CBr₂***, yielding bromoalkyne ***2d*** as a colorless liquid (29 mg, 0.11 mmol, 75%, dr = 4:1). The crude was purified by flash column chromatography using n-hexane as eluent.

**$^{1}$H NMR** (300 MHz, $CDCl_3$) δ 2.23 (d, *J* = 5.4 Hz, 1H), 2.18 (d, *J* = 10.2 Hz, 1H), 1.96 (dddd, *J* = 19.5, 9.5, 4.9, 2.7 Hz, 2H), 1.67 (dtt, *J* = 11.8, 5.2, 2.7 Hz, 3H), 1.52 – 1.37 (m, 3H), 1.14 (dt, *J* = 9.7, 2.4 Hz, 3H), 1.11 – 0.98 (m, 3H), 0.91 – 0.89 (m, 7H), 0.89 – 0.86 (m, 6H).
**$^{13}$C NMR** (75 MHz, $CDCl_3$) δ 80.4 (C), 38.4 (CH), 37.8 (C), 37.2 (CH), 34.8 (CH), 33.2 ($CH_2$), 32.4 (CH), 29.5 ($CH_2$), 29.3 ($CH_2$), 20.0 ($CH_3$), 20.0 ($CH_3$), 19.3 ($CH_3$), 17.6 ($CH_2$).
**HRMS** (GC-Q-TOF) m/z: $[M-CH_3]^+$ calcd for $C_{12}H_{18}Br$: 241.0586; found: 241.0590.

### 1.2.2. *Products from bridged family* ***(3a,b; 4b,d)***

***(3aR,6S,7aR)-3-bromo-1,1,6-trimethyl-3a,4,5,6,7,7a-hexahydro-1H-indene (3a)***

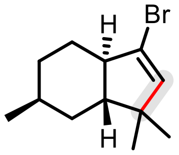

The title compound was synthesized using General Procedure N, starting from bromoalkyne ***1a*** (49 mg, 0.2 mmol), yielding a mixture of starting material and bromocyclopentene ***3a*** in 36% *yield by NMR analysis of the crude reaction mixture using $CH_2Br_2$ as internal standard*. The crude was purified by flash column chromatography using n-hexane as eluent, to afford the title compound as the major product as a pale-yellow oil (19 mg, 0.12 mmol, 39%,).

**$^{1}$H NMR** (300 MHz, $CDCl_3$) δ 5.75 (d, *J* = 2.7 Hz, 1H), 2.35 (ddt, *J* = 12.1, 10.4, 3.0 Hz, 1H), 1.99 (dtd, *J* = 12.2, 3.5, 1.5 Hz, 1H), 1.79 (dq, *J* = 13.0, 1.6 Hz, 1H), 1.67 – 1.52 (m, 2H), 1.44 (dt, *J* = 7.3, 4.3 Hz, 1H), 1.34 – 1.11 (m, 3H), 1.05 (s, 2H), 0.99 – 0.86 (m, 5H), 0.85 (s, 2H).
**$^{13}$C NMR** (75 MHz, $CDCl_3$) δ 141.9 (CH), 125.4 (C), 58.0 (CH), 52.2 (CH), 45.1 (C), 38.1 ($CH_2$), 35.4 ($CH_2$), 33.2 (CH), 26.8 ($CH_3$), 24.6 ($CH_2$), 22.5 ($CH_3$), 19.33 ($CH_3$).
**HRMS** (GC-Q-TOF) m/z: $[M]^+$ calcd for $C_{12}H_{19}Br$: 242.0670; found: 242.0674.

***(3aS,5R,7aR)-3-bromo-1,1,5-trimethyl-3a,4,5,6,7,7a-hexahydro-1H-indene (3b) and (1S,4S,5S)-6-bromo-4-isopropyl-1-methylbicyclo[3.2.1]oct-6-ene (4b)***

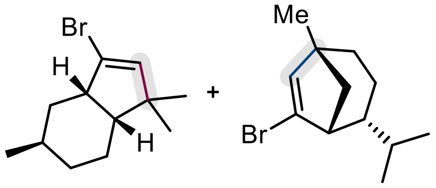


***3b***
***minor***

***4b***
***major***

The title compounds were synthesized using General Procedure N, starting from 0.29 mmol (70 mg) of bromoalkyne ***1b***, yielding a mixture of starting material and bromocyclopentenes (***3b*** and ***4b***) in *57% yield as an inseparable mixture* [3.7(***4b***):1(***3b***)] *by analysis of the 1H NMR of the crude reaction mixture using $CH_2Br_2$ as internal standard*. The crude was purified by column chromatography using n-hexane as eluent to afford the named compounds as a colorless liquid (65 mg, 0.27 mmol).

**$^1$H NMR** (400 MHz, $CDCl_3$) δ = 5.72 (d, *J* = 1.1 Hz, ***minor***), 5.62 (d, *J* = 3.0 Hz, 1H, ***major***), 3.14 (tt, *J* = 5.8, 2.9 Hz, 1H), 2.96 – 2.82 (m, 1H), 2.08 – 1.96 (m, 2H), 1.79 – 1.66 (m, 3H), 1.55 (qd, *J* = 7.4, 5.7, 3.6 Hz, 3H), 1.38 – 1.17 (m, 5H), 1.07 – 0.96 (m, 9H), 0.94 – 0.82 (m, 10H).

**$^{13}$C NMR** (101 MHz, $CDCl_3$, ***major***) δ = 140.0 (CH), 126.1 (C), 47.9 (CH), 46.8 (C), 46.6 (CH), 35.0 ($CH_2$), 33.3 ($CH_2$), 28.4 (CH), 25.4 ($CH_3$), 25.1 ($CH_2$), 22.6 ($CH_3$), 22.5 ($CH_3$).

**$^{13}$C NMR** (101 MHz, $CDCl_3$, ***minor***) δ = 138.1 ($CH_2$), 120.8 (C), 52.5 ($CH_2$), 49.1 (CH), 45.7 (C), 44.1 (CH), 33.2 ($CH_2$), 31.4 (CH), 24.8 ($CH_3$), 21.6 ($CH_3$), 21.4 ($CH_3$), 17.2 ($CH_2$).

**HRMS** (GC-Q-TOF) m/z: $[M]^+$ calcd for $C_{12}H_{19}Br$: found: 242.0667.

***Catalytic reaction for (1R,2R,4R)-2-(bromoethynyl)-4-isopropyl-1-methylcyclohexane (1c)***

The title compound was reacted using General Procedure N, starting from 0.34 mmol (83 mg) of bromoalkyne ***1c***, yielding a complex mixture of starting material and at least six products.

***(1S,4R,5S)-6-bromo-1-isopropyl-4-methylbicyclo[3.2.1]oct-6-ene (4d)***

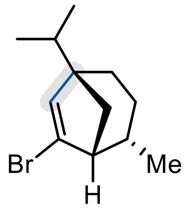


The title compound was synthesized using General Procedure N, starting from 0.2 mmol (49 mg) of bromoalkyne ***2d***, yielding bromocyclopentene ***4d*** in *68% yield by $^1$H NMR analysis of the crude reaction mixture using $CH_2Br_2$ as internal standard*. The crude was purified by column chromatography using n-hexane as eluent to afford an inseparable mixture of the title bromocyclopentene and starting material as a colorless liquid (33 mg, 0.14 mmol).

**$^1$H NMR** (400 MHz, $CDCl_3$) δ = 5.82 (s, 1H), 2.51 (d, *J* = 5.7 Hz, 1H), 2.01 (ddd, *J* = 9.1, 5.7, 2.4 Hz, 1H), 1.75 – 1.52 (m, 4H), 1.36 – 1.14 (m, 3H), 1.01 (d, *J* = 6.5 Hz, 3H), 0.84 (dd, *J* = 6.9, 4.5 Hz, 6H).

**$^{13}$C NMR** (101 MHz, $CDCl_3$) δ = 136.1 (CH), 119.9 (C), 53.3 (CH), 53.0 (C), 47.2 ($CH_2$), 33.6 (CH), 32.3 (CH), 29.0 ($CH_2$), 27.4 ($CH_2$), 20.8 ($CH_3$), 18.0 ($CH_3$), 17.9 ($CH_3$).

**HRMS** (GC-Q-TOF) m/z: $[M]^+$ calcd for $C_{12}H_{19}Br$: 242.0670; found: 242.0672.

### 1.2.2. *Products from fused family* ***(5a,b,e-g; 7b,d; 8c,c'; 9d)***

***(3aS,5R,7aR)-2-(bromomethylene)-1,1,5-trimethyloctahydro-1H-indene*** **(*5a*)**

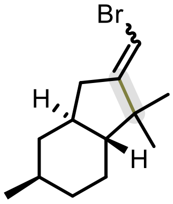


The title compound was synthesized using General Procedure N, starting from bromoalkyne ***2a*** (103 mg, 0.4 mmol), yielding bromoalkene ***5a*** in *88% yield by NMR analysis of the crude reaction mixture using $CH_2Br_2$ as internal standard*. The crude was purified by column chromatography using n-hexane as eluent to afford the named compound as a colorless liquid (99 mg, 0.38 mmol, 95%), as a mixture of isomers E/Z (4.5:1) (ratio by analysis of the NMR crude). In the same way, the reaction was carried out on a 1 mmol scale of starting material (**2a**) (1 equiv., 257 mg), using the same catalyst loading (2.5 mol%, 22 mg) and solvent dilution (0.1M, 10 mL), affording **5a** as a colorless liquid (226mg, 0.88mmol, 88%) as a 4.5:1 E/Z mixture.
**$^1$H NMR** (300 MHz, $CDCl_3$) δ 5.91 – 5.87 (m, 1H), 2.58 (ddd, *J* = 17.2, 7.2, 2.2 Hz, 1H), 1.98 – 1.72 (m, 4H), 1.67 – 1.19 (m, 9H), 1.09 (s, 4H), 1.05 (s, 1H), 0.96 – 0.81 (m, 9H).
**$^{13}$C NMR** (75 MHz, CDCl3, ***major***) δ 158.6 (C), 98.7 (CH), 57.0 (CH), 45.7 (C), 41.0 ($CH_2$), 40.3 (CH), 38.9 ($CH_2$), 35.2 ($CH_2$), 33.1 (CH), 26.9 ($CH_3$), 25.5 ($CH_2$), 24.1 ($CH_3$), 22.6 ($CH_3$).
**HRMS** (GC-Q-TOF) m/z: $[M]^+$ calcd for $C_{13}H_{21}Br$: 256.0827; found: 256.0826.

***(3aS,5R,7aR)-1,1,5-trimethyloctahydro-2H-inden-2-one*** **(*5a-CO*)**

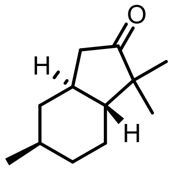


The procedure described in the literature was followed.[18] In a flamed Schlenk flask, under Ar, bromoalkene ***5a*** (99 mg, 0.38 mmol, 1 equiv.) was dissolved in DCM (0.01M) and cooled to -78 °C. A steam of oxygen was bubbled in the reaction mixture for 10 minutes. Then ozone was bubbled until the color changed to blue and the resulting blue mixture was allowed to warm to room temperature. A steam of argon was bubbled to remove the ozone from the solution for 15 minutes. After removing the blue color of the reaction mixture, $PPh_3$ (2 equiv., 200 mg, 0.76 mmol) was added and the resulting mixture was stirred overnight. After completion of the reaction the solvent was removed under vacuum and purified by column chromatography using hexane/EtOAc (100:1 to 10:1) as eluents to afford the pure title ketone ***5a-CO*** as a colorless liquid (40 mg, 0.22 mmol, 58%).
**$^1$H NMR** (300 MHz, $CDCl_3$) δ 2.43 – 2.28 (m, 1H), 2.00 – 1.57 (m, 5H), 1.57 – 1.34 (m, 1H), 1.25 – 1.09 (m, 2H), 1.04 – 0.72 (m, 12H).
**$^{13}$C NMR** (75 MHz, CDCl3) δ 223.6 (C), 54.0 (CH), 47.8 (C), 44.1 ($CH_2$), 40.7(CH), 38.1 ($CH_2$), 35.2 ($CH_2$), 33.0 (CH), 25.3 ($CH_3$), 23.0 ($CH_2$), 22.6 ($CH_3$), 18.5 ($CH_3$).
**HRMS** (GC-Q-TOF) m/z: $[M+H]^+$ calcd for $C_{12}H_{21}O$: 181.1587; found: 181.1588.

***1-(2,4-dinitrophenyl)-2-((3aS,5R,7aR,E)-1,1,5-trimethyloctahydro-2H-inden-2-ylidene)hydrazine* (*9*)**

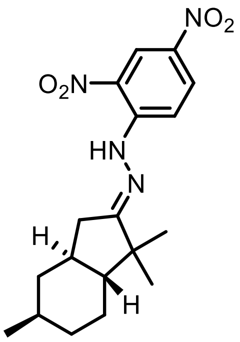


The procedure was followed as described in the literature.[19] In a round bottom flask, to a solution of ketone ***5a-CO*** (40 mg, 0.22 mmol, 1 equiv.) in EtOH/$H_2O$ (3.7 mL/1.2 mL) was added *(2,4-dinitrophenyl)hydrazine* (1.2 equiv., 52 mg, 0.26 mmol) and $H_2SO_4$ (conc.). The reaction mixture was stirred for 3 hours at room temperature. Then the mixture was quenched with water, extracted with EtOAc (x3) and washed with $NaHCO_3$ (sat.) and brine. The combined organic layers were dried over $Na_2SO_4$, filtered and the solvent removed in vacuum. The crude was purified by column chromatography using hexane/EtOAc (20:1) as eluent to afford the pure hydrazone ***6a*** as a yellow solid (38 mg, 0.11 mmol, 47%). The solid was re-dissolved in MeOH, then subjected to liquid–liquid crystallization from a 1:1 MeOH/$Et_2O$ mixture at 0 °C, affording the desired compound (***9***) after 3 days.
**$^1$H NMR** (300 MHz, $CDCl_3$) δ 10.75 (s, 1H), 9.09 (d, *J* = 2.6 Hz, 1H), 8.26 (dd, *J* = 9.6, 2.6 Hz, 1H), 7.94 (d, *J* = 9.6 Hz, 1H), 2.71 (dd, *J* = 17.1, 7.2 Hz, 1H), 2.12 – 1.31 (m, 7H), 1.23 (s, 5H), 1.07 – 0.91 (m, 7H).
**$^{13}$C NMR** (75 MHz, $CDCl_3$) δ 173.2 (C), 145.3 (C), 137.5 (C), 130.0 (CH), 128.8 (C), 123.7 (CH), 116.5 (CH), 55.3 (CH), 45.1 (C), 40.6 ($CH_2$), 39.6 (CH), 35.0 ($CH_2$), 33.7 ($CH_2$), 33.0 (CH), 25.4 ($CH_3$), 25.1 ($CH_2$), 22.5 ($CH_3$), 21.9 ($CH_3$).
**HRMS** (GC-Q-TOF) m/z: $[M+H]^+$ calcd for $C_{18}H_{25}N_4O_4$: 361.1870; found: 361.1873.

***(3aR,5R,7aR)-2-(bromomethylene)-1,1,5-trimethyloctahydro-1H-indene (5b) and (1S,4S,5R)-7-(bromomethylene)-4-isopropyl-1-methylbicyclo[3.2.1]octane (6b)***

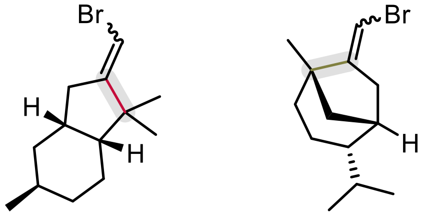


**5b** *major* **6b** *minor*

The title compounds were synthesized using General Procedure N, starting from bromoalkyne ***2b*** (26 mg, 0.1 mmol), yielding a mixture of bromoolefins in *70% yield as an inseparable mixture of regio-and diastereoisomers [4.8(**5b**):1(**6b**), **5b** E/Z = 4.8:1, **6b** E/Z = 2.5:1]by NMR analysis of the crude reaction mixture using $CH_2Br_2$ as internal standard*. The crude was purified by flash column chromatography using n-hexane as eluent to afford the title compound as a colorless liquid (20 g, 0.08 mmol, 77%). The proportions were inferred by analysis of the 1-D and 2-D NMR spectra, see Upporting Information NMR Spectra.

**$^{1}$H NMR** (600 MHz, $CDCl_3$,) δ 5.89 (t, *J* = 1.8 Hz, 1H, ***6b-Z***), 5.87 (bt, 1H, ***5b-Z***), 5.83 (t, *J* = 2.7 Hz, 1H, ***5b-E***), 5.74 (t, *J* = 2.6 Hz, 1H, ***6b-E***), *, 2.45 – 2.54 (m, 1H **5b-E**, 1H ***5b-Z***), 2.45-2.36 (m, 1H ***5b-E***, 1H ***5b-Z***), 2.36 – 2.16 (m, 1H ***5b-E***, 2H ***5b-Z***, 4H ***6b-E***), 1.75 – 1.58 (m), 1.58 – 1.30 (m), 1.24 (s, 3H ***5b-Z***), 1.16 (dt, *J* = 13.1, 5.4 Hz, 1H ***5b-E***), 1.08 (s, 3H ***6b-E***), 1.07 (s, 3H ***5b-E***), 1.03 (s, 3H ***5b-E***), 1.20 – 0.83 (m), 0.83 – 0.73 (m).
*impurities: 3.53 (dt, *J* = 9.0, 4.6 Hz), 3.35 (q, *J* = 9.0 Hz)
***5b-Z*****:** **$^{13}$C NMR** (150 MHz, $CDCl_3$) δ 157.6 (C), 98.3 (CH), 50.3 (CH), 48.4 (C), 35.8 ($CH_2$), 35.5 ($CH_2$), 34.1 ($CH_2$), 34.0 (CH), 30.2 ($CH_3$), 26.5 (CH), 24.9 ($CH_2$), 22.8 ($CH_3$), 22.6 ($CH_3$).
***5b-E*****:** **$^{13}$C NMR** (150 MHz, $CDCl_3$) δ 154.0 (C), 95.7 (CH), 51.6 (CH), 47.4 (C), 38.0 ($CH_2$), 37.5 ($CH_2$), 34.5 ($CH_2$), 34.3 (CH), 26.6 ($CH_3$), 26.1 (CH), 25.4 ($CH_2$), 22.6 ($CH_3$), 21.9 ($CH_3$).
***6b-Z*****:** **$^{13}$C NMR** (150 MHz, $CDCl_3$) δ 155.6 (C), 96.0 (CH), 48.3 ($CH_2$), 47.9 (CH), 46.9 (CH), 46.0 (C), 35.1 ($CH_2$), 29.2 (CH), 25.0 ($CH_2$), 24.5 ($CH_2$), 23.7 ($CH_3$), 21.6 ($CH_3$), 20.7 ($CH_3$).
***6b-E*** **(asignable signals): $^{13}$C NMR** (150 MHz, $CDCl_3$) δ 151.4 (C), 94.0 (CH), 49.7 ($CH_2$), 45.7 (C).
**HRMS** mixture of isomers

### *(3aS,6R,7aS)-2-bromo-6-isopropyl-3a-methyl-3a,4,5,6,7,7a-hexahydro-1H-indene (7c') and (4S,7R,7aS)-2-bromo-4-isopropyl-7-methyl-3a,4,5,6,7,7a-hexahydro-1H-indene (7c)*

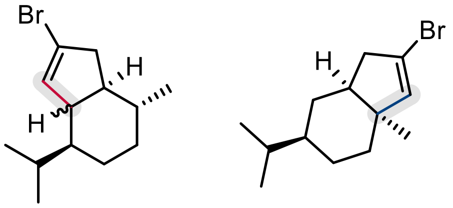


**7c** ***minor*** **7c'** ***major***

The title compounds were synthesized using General Procedure N, starting from bromoalkyne **2c** (54 mg, 0.2 mmol), yielding a mixture of bromocyclopentenes ***7c'*** and ***7c*** in *53% yield as an inseparable mixture of regio- and diastereoisomers [8(**7c'**):3(**7c-cis**):1(**7c-trans**)] by NMR analysis of the crude reaction mixture using $CH_2Br_2$ as internal standard*. The crude mixture was purified by column chromatography using n-hexane as eluent to afford a mixture of the title compounds as a colorless liquid (22.8 mg, 0.09 mmol, 44%).
**$^{1}$H NMR** (300 MHz, $CDCl_3$) δ = 6.09 (d, *J* = 2.3 Hz, ***minor***), 5.86 (s, ***minorm***), 5.64 – 5.55 (m, 1H, ***major***), 2.99 (ddd, *J* = 15.7, 6.0, 2.6 Hz, 1H), 2.50 (dd, *J* = 14.8, 6.3 Hz, 1H), 2.38 – 2.19 (m, 1H), 1.98 (d, *J* = 15.7 Hz, 1H), 1.92 – 1.71 (m, 3H), 1.56 (d, *J* = 5.2 Hz, 4H), 1.48 – 1.33 (m, 2H), 1.33 – 1.17 (m, 2H), 0.99 (s, 4H), 0.97 – 0.87 (m, 6H), 0.87 – 0.77 (m, 10H).
**$^{13}$C NMR** (75 MHz, $CDCl_3$) δ = 140.0 (CH, ***major***), 134.0 (CH), 119.9 (C, ***major***), 56.5 (CH), 54.1 ($CH_2$), 48.2 (C, ***major***), 47.0 ($CH_2$, ***major***), 46.5 (CH), 45.6 (CH, ***major***), 43.7 ($CH_2$), 42.1 (CH ***major***), 35.8 ($CH_2$, ***major***), 34.3 ($CH_2$, ***major***), 32.8 (CH, ***major***), 29.7 (CH), 27.9 ($CH_3$, ***major***), 26.3 ($CH_2$, ***major***), 26.2 ($CH_2$), 20.8 ($CH_3$), 20.3 ($CH_3$), 19.9 ($CH_3$, ***major***), 19.8 ($CH_3$, ***major***), 16.8 ($CH_3$).
**HRMS** (GC-Q-TOF) m/z: $[M]^+$ calcd for $C_{13}H_{21}Br$: 256.0827; found: 256.0828.

### *(1S,4R,5R,E)-7-(bromomethylene)-1-isopropyl-4-methylbicyclo[3.2.1]octane (6d) and (3aR,5R,7aS,Z)-2-(bromomethylene)-5-isopropyloctahydro-1H-indene (8d)*

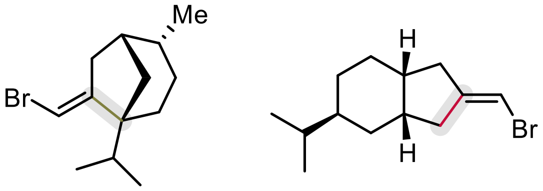


**6d** *major* **8d** *minor*

The title compound was synthesized using General Procedure N, starting from bromoalkyne ***2d*** (28 mg, 0.11 mmol) of, yielding a mixture of bromoolefins ***6d*** and ***8d*** in *57% combined yield [1.7(**6d**): 1(**8d**)] by NMR analysis of the crude reaction mixture using $CH_2Br_2$ as Internal standard*. In the $^1$H NMR spectrum of the crude reaction signals corresponding bromocyclopentenes ***7c'*** and ***7c*** from the catalytic reaction of the minor diastereoisomer of substrate ***2d*** are also found. The crude was purified by column chromatography using n-hexane as eluent, affording the mixture of ***6d*** and ***8d*** as a colorless liquid (18 mg, 0.07).

**$^1$H NMR** (400 MHz, $CDCl_3$) δ = 5.95 (d, *J* = 2.6 Hz), 5.66 (t, *J* = 2.7 Hz, 1H), 2.42 (dt, *J* = 17.6, 2.5 Hz, 2H), 2.11 (ddd, *J* = 17.6, 6.6, 3.2 Hz, 1H), 1.98 (dd, *J* = 10.3, 4.7 Hz, 1H), 1.90 – 1.69 (m, 4H), 1.69 – 1.57 (m, 4H), 1.55 (s, 3H), 1.33 (dd, *J* = 11.1, 2.9 Hz, 3H), 1.16 (ddq, *J* = 11.7, 4.8, 2.7 Hz, 2H), 1.06 (ddd, *J* = 18.2, 11.8, 5.4 Hz, 1H), 0.99 (s, 1H), 0.94 – 0.89 (m, 5H), 0.89 – 0.85 (m, 5H), 0.85 – 0.81 (m, 8H).

**$^{13}$C NMR** (101 MHz, $CDCl_3$, ***major***) δ = 154.1 (C), 96.2 (CH), 53.1 (C), 39.1 ($CH_2$), 39.0 ($CH_2$), 38.6 (CH), 35.9 ($CH_2$), 35.5 (CH), 32.2 (CH), 28.8 ($CH_2$), 20.3 ($CH_3$), 18.5 ($CH_3$), 18.2 ($CH_3$).

**$^{13}$C NMR** (101 MHz, $CDCl_3$, ***minor***) δ = 150.9 (CH), 94.2 (C), 39.5 ($CH_2$), 39.4 (CH), 38.5 ($CH_2$), 35.6 (CH), 34.0 ($CH_2$), 31.7 (CH), 30.6 (CH), 29.5 ($CH_2$), 22.8 ($CH_2$), 18.2 ($CH_3$), 18.0 ($CH_3$).

**HRMS** (GC-Q-TOF) m/z: $[M]^+$ calcd for $C_{13}H_{21}Br$: 256.0827; found: 256.0827.

## 4. *Exo reactivity substrates* ***(2e-g)***

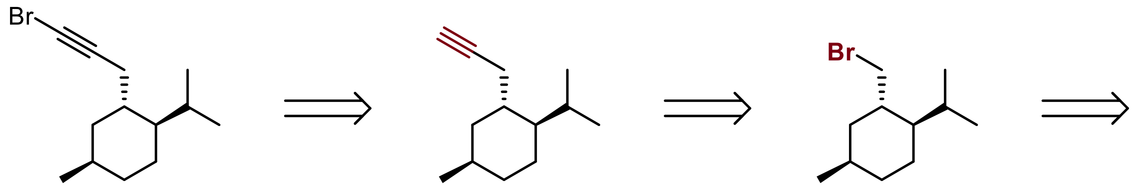


**2e** **2e-CCH** **2e-Br**

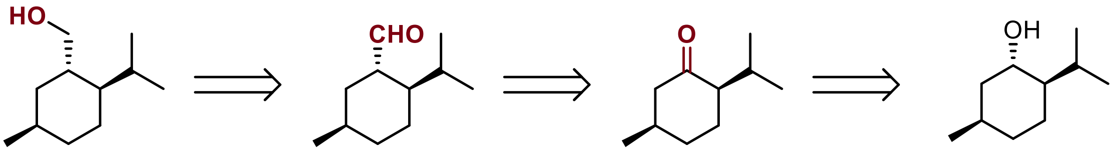


**2e-OH** **2e-CHO** **2e-CO** ***1R*-(+)-Isomenthol**

### *(2R,5R)-2-isopropyl-5-methylcyclohexan-1-one (2e-CO)*

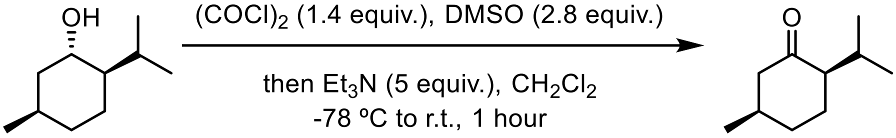


**2e**-CO

The procedure described in the literature was followed.[6] In a Schlenk flask under Ar, DMSO (2.8 equiv.) was added dropwise to a solution of oxalyl chloride (1.4 equiv.) in dry DCM (0.5M) at -78 ºC. Then, *(1R)-(+)-isomenthol* (1.56 g, 10 mmol, 1 equiv.) dissolved in dry DCM (1M) was added dropwise to the reaction mixture at the same temperature. After stirring for 15 minutes triethylamine (5 equiv.) was added dropwise and the reaction was allowed to reach room temperature. Then, water was added, and the crude reaction mixture was extracted with $Et_2O$ (x3), the combined organic layers were washed with 1N HCl, saturated aqueous $NaHCO_3$ and saturated aqueous NaCl. The combined organic layers were dried over $Na_2SO_4$ and concentrated under vacuum affording aldehyde ***2e-CO*** as a colorless liquid (61 mg, 0.33 mmol). The crude ketone was used in the following step without further purification.

**$^1$H NMR** (300 MHz, $CDCl_3$) δ = 2.36 – 2.24 (m, 1H), 2.17 – 2.05 (m, 1H), 2.05 – 1.88 (m, 4H), 1.79 – 1.59 (m, 3H), 1.55 – 1.37 (m, 2H), 0.98 (d, *J* = 6.5 Hz, 3H), 0.93 (d, *J* = 6.3 Hz, 4H), 0.84 (d, *J* = 6.4 Hz, 3H).

**HRMS** not purified

***(1S,2R,5R)-2-isopropyl-5-methylcyclohexane-1-carbaldehyde (2e-CHO)***

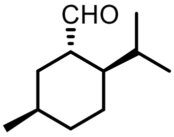


The title compound was synthesized using General Procedure A, starting from 10 mmol (1.54 g) of isomenthone (***2e-CO***), yielding a mixture of isomenthone (***2e-CO***), menthone, and the corresponding aldehyde (***2e-CHO***). This mixture was taken forward in the synthesis and the purification was carried out along the following steps.

**$^1$H NMR** (300 MHz, $CDCl_3$) δ = 2.36 – 2.21 (m, 2H), 2.16 (s, 9H), 1.50 – 1.25 (m, 4H), 1.17 (t, *J* = 7.0 Hz, 1H), 1.08 – 0.90 (m, 5H), 0.90 – 0.68 (m, 11H).

**$^{13}$C NMR** (75 MHz, $CDCl_3$) δ = 206.36, 76.74, 65.94, 57.29, 48.09, 34.51, 29.47, 26.97, 21.55, 21.26, 20.98, 19.98.

**HRMS** not purified

***((1S,2R,5R)-2-isopropyl-5-methylcyclohexyl)methanol (2e-OH)***

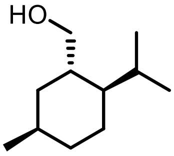


The title compound was synthesized using General Procedure D (using 1 equiv. of $LiAlH_4$), starting from crude aldehyde ***2e-CHO***, yielding alcohol ***2e-OH*** as a colorless liquid (334 mg, 2.0 mmol, 20% yield for three steps from ketone ***2e-CO***). The crude was purified by column chromatography using hexane/EtOAc (10:1) as eluent.

**$^1$H NMR** (300 MHz, $CDCl_3$) δ = 3.61 (t, *J* = 4.8 Hz, 2H), 2.16 (td, *J* = 7.1, 2.6 Hz, 1H), 2.02 – 1.88 (m, 2H), 1.88 – 1.71 (m, 3H), 1.71 – 1.52 (m, 5H), 1.41 (dt, *J* = 8.1, 5.2 Hz, 9H), 1.27 – 1.00 (m, 4H), 0.94 – 0.87 (m, 16H), 0.82 (dt, *J* = 11.9, 3.3 Hz, 9H).

***(1R,2S,4R)-2-(bromomethyl)-1-isopropyl-4-methylcyclohexane (2e-Br)***

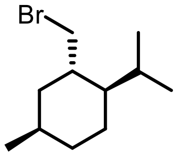

The title compound was synthesized using General Procedure E, starting from 2.0 mmol (334 mg) of alcohol ***2e-OH***, yielding bromoalkane ***2e-Br*** as a colorless liquid (247 mg, 1.1 mmol, 54%). The crude was purified by flash column chromatography using n-hexane as eluent.

**$^{1}$H NMR** (300 MHz, $CDCl_3$) δ = 3.51 (qd, *J* = 9.9, 5.6 Hz, 2H), 1.94 – 1.83 (m, 2H), 1.81 – 1.69 (m, 2H), 1.60 (ddd, *J* = 12.7, 7.8, 4.4 Hz, 2H), 1.54 (s, 2H), 1.40 (dd, *J* = 10.0, 5.5 Hz, 5H), 1.33 – 1.18 (m, 2H), 1.13 – 1.00 (m, 1H), 0.91 (ddt, *J* = 7.0, 4.9, 2.3 Hz, 10H), 0.84 (d, *J* = 6.8 Hz, 3H).

**$^{13}$C NMR** (75 MHz, $CDCl_3$) δ = 49.4 ($CH_2$), 44.5 (CH), 40.2 (CH), 36.2 ($CH_2$), 35.8 (CH), 31.0 (CH), 27.1 ($CH_2$), 26.8 ($CH_3$), 21.4 ($CH_3$), 20.3 ($CH_2$), 18.0 ($CH_3$).

**HRMS** unstable

***(1R,2R,4R)-1-isopropyl-4-methyl-2-(prop-2-yn-1-yl)cyclohexane (2e-CCH)***

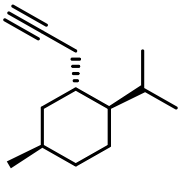

The title compound was synthesized using General Procedure F, starting from 1.1 mmol (247 mg) of bromoalkane ***2e-Br***, yielding alkyne ***2e-CCH*** as a colorless liquid (53 mg, 0.3 mmol, 28%). The crude was purified by flash column chromatography using n-hexane as eluent.

**$^{1}$H NMR** (300 MHz, $CDCl_3$) δ = 2.22 (dd, *J* = 6.3, 2.7 Hz, 2H), 1.94 (t, *J* = 2.7 Hz, 1H), 1.78 –1.84 (m, *J* = 19.2, 6.6 Hz, 3H), 1.47 (ddd, *J* = 9.4, 8.0, 3.5 Hz, 2H), 1.43 – 1.24 (m, 4H), 1.07 – 1.18 (m, 1H), 0.91 (dd, *J* = 6.9, 2.3 Hz, 7H), 0.81 (d, *J* = 6.8 Hz, 3H).

**$^{13}$C NMR** (75 MHz, $CDCl_3$) δ = 83.7 (C), 69.3 (CH), 45.4 (CH), 36.6 ($CH_2$), 33.3 (CH), 31.2 ($CH_2$), 27.3 (CH), 26.9 (CH), 23.0 ($CH_2$), 21.6 ($CH_3$), 20.1 ($CH_3$), 19.9 ($CH_2$), 17.8 ($CH_3$).

**HRMS** non-ionizable

***(1R,2R,4R)-2-(3-bromoprop-2-yn-1-yl)-1-isopropyl-4-methylcyclohexane* (*2e*)**

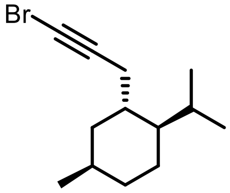

The title compound was synthesized using General Procedure G, starting from 0.3 mmol (53 mg) of alkyne ***2e-CCH***, yielding bromoalkyne ***2e*** as a colorless liquid (54 mg, 0.21 mmol, 71%). The crude was purified by flash column chromatography using n-hexane as eluent.

**$^{1}$H NMR** (300 MHz, $CDCl_3$) δ = 2.27 (d, J = 6.3 Hz, 2H), 1.95 – 1.72 (m, 2H), 1.41 (dddd, J = 27.5, 20.3, 10.9, 5.1 Hz, 7H), 1.19 – 1.03 (m, 1H), 0.94 (dd, J = 6.8, 2.4 Hz, 6H), 0.84 (d, J = 6.8 Hz, 3H).

**$^{13}$C NMR** (75 MHz, $CDCl_3$) δ 79.6 (C), 45.6 (CH), 38.2 (C), 36.7 ($CH_2$), 33.5 (C), 31.2 ($CH_2$), 27.3 (C), 27.0 (CH), 24.3 ($CH_2$), 21.6 ($CH_3$), 20.1 ($CH_3$), 19.9 ($CH_2$), 17.8 ($CH_3$).
**HRMS** (GC-Q-TOF) m/z: $[M]^+$ calcd for $C_{13}H_{21}Br$: 256.0827; found: 256.0825.

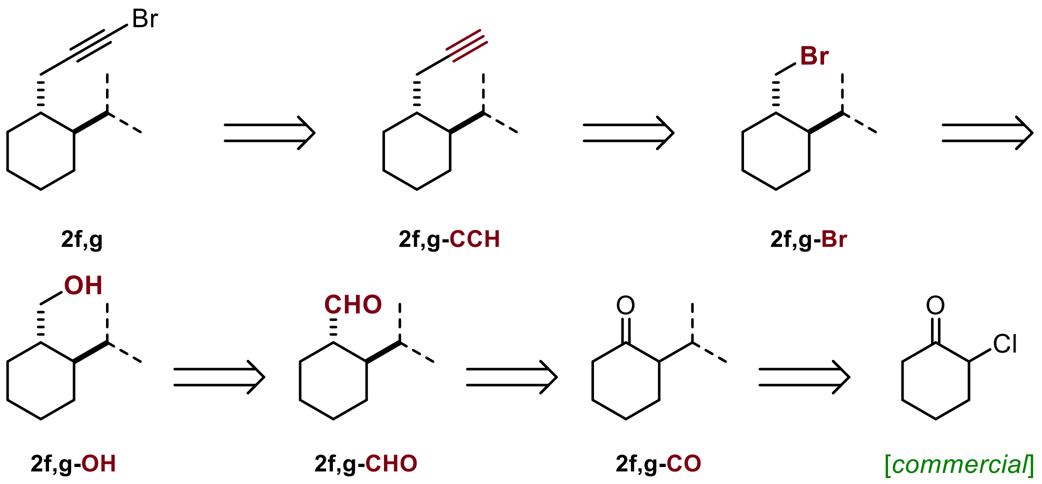


***(S)-2-isopropylcyclohexan-1-one*** **(2*f*-*CO*)**

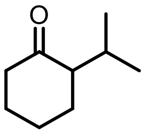


The title compound was synthesized using the procedure described in reference,[23] starting from 10 mmol (1.3 g) of *2-chlorocyclohexanone* yielding ketone ***2f-CO*** as a colorless liquid (567 mg, 4.0 mmol, 40%). The crude was purified by column chromatography using Hexane/$Et_2O$ (20:1 to 10:1) as eluent. The $^1$H NMR spectrum matches the one previously reported.[23]
**$^1$H NMR** (300 MHz, $CDCl_3$) δ 2.43 – 2.19 (m, 1H), 2.13 (dd, $J$ = 13.0, 6.5 Hz, 1H), 1.96 (dtt, $J$ = 12.5, 5.4, 2.9 Hz, 1H), 1.91 – 1.68 (m, 1H), 1.68 – 1.47 (m, 1H), 0.88 (dd, $J$ = 6.6, 2.7 Hz, 3H).

***(1R,2S)-2-isopropylcyclohexane-1-carbaldehyde*** **(2*f*-*CHO*)**

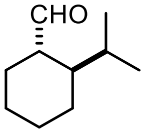


The title compound was synthesized using General Procedure A, starting from 4 mmol (567 mg) of ketone ***2f-CO***, yielding aldehyde ***2f-CHO*** as a colorless liquid (617 mg, 4.0 mmol, quantitative, dr = 20:1). The crude aldehyde was used in the next step without further purification. The $^1$H NMR spectrum matches the one previously reported.[24]
**$^1$H NMR** (300 MHz, $CDCl_3$) δ 9.51 (d, $J$ = 4.5 Hz, 1H), 2.44 – 2.03 (m, 3H), 2.03 – 1.92 (m, 1H), 1.81 – 1.52 (m, 8H), 1.21 (t, $J$ = 7.0 Hz, 3H), 0.96 – 0.84 (m, 6H), 0.81 (d, $J$ = 6.8 Hz, 3H).
**HRMS** (GC-Q-TOF) m/z: $[M+H]^+$ calcd for $C_{10}H_{19}O$: 155.1430; found: 155.1437.

***((1R,2S)-2-isopropylcyclohexyl)methanol*** **(2*f*-*OH*)**

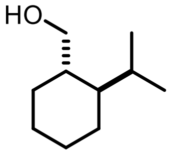

The title compound was synthesized using General Procedure D (using 1 equiv. of $LiAlH_4$), starting from 4 mmol (567 mg) of aldehyde ***2f-CHO***, yielding alcohol ***2f-OH*** as a colorless liquid (348 mg, 2.23 mmol, 56%). The crude alcohol was used in the next step without further purification.

**$^1$H NMR** (300 MHz, $CDCl_3$) δ 3.71 – 3.62 (m, 1H), 3.57 – 3.47 (m, 1H), 1.92 (td, *J* = 6.9, 3.3 Hz, 1H), 1.86 – 1.67 (m, 5H), 1.63 (dt, *J* = 12.3, 2.7 Hz, 1H), 1.35 (td, *J* = 6.7, 3.4 Hz, 1H), 1.14 (dddd, *J* = 19.3, 9.0, 5.0, 2.3 Hz, 4H), 1.06 – 0.93 (m, 1H), 0.88 (d, *J* = 7.0 Hz, 4H), 0.76 (d, *J* = 7.0 Hz, 3H).

**$^{13}$C NMR** (75 MHz, $CDCl_3$) δ 65.6 ($CH_2$), 44.3 (CH), 41.8 (CH), 30.1 ($CH_2$), 27.0 (CH), 26.5 ($CH_2$), 26.3 ($CH_2$), 24.4 ($CH_2$), 21.5 ($CH_3$), 15.9 ($CH_3$).

**HRMS** not purified

***(1R,2S)-1-(bromomethyl)-2-isopropylcyclohexane (2f-Br)***

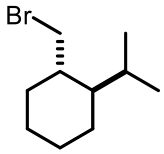


The title compound was synthesized using General Procedure E, starting from 2.2 mmol (348 mg) of alcohol ***2f-OH***, yielding bromoalkane ***2f-Br*** as a colorless liquid (399 mg, 1.8 mmol, 82%). The crude was purified by flash column chromatography using n-hexane as eluent.

**$^1$H NMR** (300 MHz, $CDCl_3$) δ 3.49 (d, *J* = 4.1 Hz, 2H), 1.94 (ddq, *J* = 10.2, 6.9, 3.4 Hz, 1H), 1.84 – 1.70 (m, 3H), 1.70 – 1.60 (m, 1H), 1.54 – 1.44 (m, 1H), 1.39 – 1.18 (m, 4H), 1.06 – 0.95 (m, 1H), 0.92 (d, *J* = 6.9 Hz, 3H), 0.77 (d, *J* = 7.0 Hz, 3H).

**$^{13}$C NMR** (75 MHz, $CDCl_3$) δ 45.2 (CH), 40.6 (CH), 40.3 ($CH_2$), 31.8 ($CH_2$), 26.7 (CH), 26.4 ($CH_2$), 26.2 ($CH_2$), 24.1 ($CH_2$), 21.3 ($CH_3$), 15.6 ($CH_3$).

**HRMS** (GC-Q-TOF) m/z: $[M]^+$ calcd for $C_{10}H_{19}Br$: 218.0668; found: 218.0670.

***(1S,2S)-1-isopropyl-2-(prop-2-yn-1-yl)cyclohexane* (*2f-CCH*)**

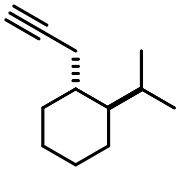

The title compound was synthesized using General Procedure F, starting from 1.8 mmol (399 mg) of bromoalkane ***2f-Br***, yielding alkyne ***2f-CCH*** as a colorless liquid (164 mg, 1.1 mmol, 62%). The crude was purified by flash column chromatography using n-hexane as eluent.

**$^1$H NMR** (300 MHz, $CDCl_3$) δ 2.27 (ddd, *J* = 16.9, 3.8, 2.7 Hz, 1H), 2.16 (ddd, *J* = 16.9, 6.8, 2.7 Hz, 1H), 1.93 (t, *J* = 2.7 Hz, 2H), 1.85 (dtd, *J* = 9.5, 3.3, 2.0 Hz, 1H), 1.72 (dddd, *J* = 9.1, 4.5, 3.2, 1.6 Hz, 2H), 1.68 – 1.58 (m, 1H), 1.44 – 1.32 (m, 1H), 1.29 – 1.14 (m, 4H), 1.03 – 0.93 (m, 1H), 0.90 (d, *J* = 6.9 Hz, 3H), 0.75 (d, *J* = 6.9 Hz, 3H).

**$^{13}$C NMR** (75 MHz, $CDCl_3$) δ 83.3 (C), 69.4 (CH), 46.2 (CH), 38.3 (CH), 32.6 ($CH_2$), 26.9 (CH), 26.6 ($CH_2$), 24.3 ($CH_2$), 22.8 ($CH_2$), 21.5 ($CH_3$), 15.6 ($CH_3$).

**HRMS** (GC-Q-TOF) m/z: $[M-CH_3]^+$ calcd for $C_{11}H_{17}$: 149.1330; found: 149.1337.

***(1S,2S)-1-(3-bromoprop-2-yn-1-yl)-2-isopropylcyclohexane* (*2f*)**

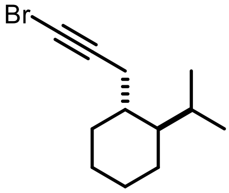


The title compound was synthesized using General Procedure G, starting from 1.1 mmol (164 mg) of alkyne ***2f*-*CCH***, yielding bromoalkyne ***2f*** as a colorless liquid (170 mg, 0.7 mmol, 59%). The crude was purified by flash column chromatography using n-hexane as eluent.

**$^1$H NMR** (300 MHz, $CDCl_3$) δ 2.29 (dd, *J* = 16.8, 3.8 Hz, 1H), 2.16 (dd, *J* = 16.8, 7.0 Hz, 1H), 1.92 (ddt, *J* = 10.1, 6.9, 3.2 Hz, 1H), 1.83 (ddt, *J* = 9.9, 5.6, 3.1 Hz, 1H), 1.78 – 1.68 (m, 2H), 1.68 – 1.57 (m, 1H), 1.38 (dtq, *J* = 10.5, 6.9, 3.6 Hz, 1H), 1.21 (d, *J* = 8.1 Hz, 4H), 0.97 (dd, *J* = 12.0, 2.9 Hz, 1H), 0.90 (d, *J* = 6.9 Hz, 3H), 0.74 (d, *J* = 6.9 Hz, 3H).

**$^{13}$C NMR** (75 MHz, $CDCl_3$) δ 79.2 (C), 46.4 (CH), 38.5 (CH), 38.2 (C), 32.8 ($CH_2$), 26.9 (CH), 26.5 ($CH_2$), 24.3 ($CH_2$), 24.1 ($CH_2$), 21.5 ($CH_3$), 15.6 ($CH_3$).

**HRMS** (GC-Q-TOF) m/z: $[M\text{-}CH_3]^+$ calcd for $C_{11}H_{16}Br$: 227.0435; found: 227.0432.

***(S)-2-cyclopentylcyclohexan-1-one* (*2g*-*CO*)**

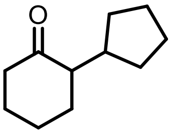


The title compound was synthesized using the procedure described in reference,[23] starting from 10 mmol (1.3 g) of the *2-chlorocyclohexanone* yielding ketone ***2g*-*CO*** as a colorless liquid (299 mg, 1.8 mmol, 18%). The crude was purified by column chromatography using Hexane/DCM (4:1 to DCM) as eluent. The $^1$H NMR spectra matches the one previously reported. [23]

**$^1$H NMR** (300 MHz, $CDCl_3$) δ 2.33 (dt, *J* = 18.0, 5.5 Hz, 3H), 2.21 – 1.90 (m, 4H), 1.80 (dddd, *J* = 25.3, 12.6, 6.0, 3.6 Hz, 4H), 1.68 – 1.40 (m, 7H), 1.07 (dtd, *J* = 21.5, 8.6, 4.8 Hz, 2H).

***(1R,2S)-2-cyclopentylcyclohexane-1-carbaldehyde* (*2g*-*CHO*)**

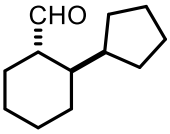


The title compound was synthesized using General Procedure A, starting from 1.8 mmol (299 mg) of ketone ***2g*-*CO***, yielding aldehyde ***2g*-*CHO*** as a colorless liquid. The crude aldehyde was used in the next step without further purification.

**$^1$H NMR** (300 MHz, $CDCl_3$) δ 9.55 (dd, *J* = 3.9, 1.6 Hz, 1H), 2.13 (ddd, *J* = 8.5, 5.2, 2.9 Hz, 2H), 2.05 – 1.33 (m, 22H), 1.33 – 1.22 (m, 3H), 1.22 – 0.98 (m, 5H), 0.94 – 0.76 (m, 2H).

**HRMS** not purified

***((1R,2S)-2-cyclopentylcyclohexyl)methanol* (*2g*-*OH*)**

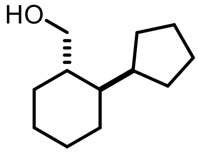


The title compound was synthesized using General Procedure D, starting from 1.8 mmol (299 mg) of aldehyde ***2g*-*CHO***, yielding alcohol ***2g*-*OH*** as a colorless liquid (154 mg,

0.84 mmol, 47%). The crude was purified by flash column chromatography using hexane/EtOAc (10:1 to 5:1) as eluent.
**$^{1}$H NMR** (300 MHz, $CDCl_3$) δ 3.66 (ddd, *J* = 16.5, 10.8, 4.2 Hz, 2H), 2.10 (h, *J* = 8.5 Hz, 1H), 1.98 – 1.70 (m, 2H), 1.70 – 1.39 (m, 10H), 1.25 (d, *J* = 8.1 Hz, 7H).**$^{13}$C NMR** (75 MHz, $CDCl_3$) δ 65.8 ($CH_2$), 42.7 (CH), 41.1 (CH), 40.4 (CH), 30.4 ($CH_2$), 28.6 ($CH_2$), 27.3 ($CH_2$), 25.8 ($CH_2$), 25.7 ($CH_2$), 25.6 ($CH_2$), 25.4 ($CH_2$), 25.3 ($CH_2$).
**HRMS** (GC-Q-TOF) m/z: $[M-OH]^+$ calcd for $C_{12}H_{21}$: 165.1638; found: 165.1640.

***(1R,2S)-1-(bromomethyl)-2-cyclopentylcyclohexane (2g-Br)***

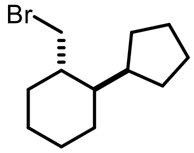


The title compound was synthesized using General Procedure E, starting from 0.84 mmol (154 mg) of alcohol ***2g-OH***, yielding bromoalkane ***2g-Br*** as a colorless liquid (151 mg, 0.61 mmol, 73%). The crude was purified by flash column chromatography using n-hexane as eluent.
**$^{1}$H NMR** (300 MHz, $CDCl_3$) δ 3.55 (dd, *J* = 4.5, 3.0 Hz, 2H), 2.21 – 2.01 (m, 1H), 1.79 – 1.61 (m, 4H), 1.56 (s, 8H), 1.40 – 1.00 (m, 6H).
**$^{13}$C NMR** (75 MHz, $CDCl_3$) δ 42.3 (CH), 41.8 (CH), 40.4 ($CH_2$), 40.0 (CH), 30.5 ($CH_2$), 30.1 ($CH_2$), 26.8 ($CH_2$), 25.8 ($CH_2$), 25.7 ($CH_2$), 25.4 ($CH_2$), 25.2 ($CH_2$), 25.2 ($CH_2$).
**HRMS** (GC-Q-TOF) m/z: $[M]^+$ calcd for $C_{12}H_{21}Br$: 244.0827; found: 244.0826.

***(1S,2S)-1-cyclopentyl-2-(prop-2-yn-1-yl)cyclohexane* (*2g-CCH*)**

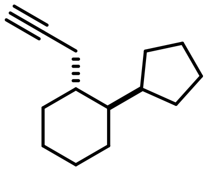

The title compound was synthesized using General Procedure F, starting from 0.61 mmol (151 mg) of bromoalkane ***2g-Br***, yielding alkyne ***2g-CCH*** as a colorless liquid (87 mg, 0.46 mmol, 75%). The crude was purified by flash column chromatography using n-hexane as eluent.
**$^{1}$H NMR** (300 MHz, $CDCl_3$) δ 2.34 – 2.18 (m, 2H), 2.17 – 2.01 (m, 1H), 1.93 (t, J = 2.7 Hz, 1H), 1.88 – 1.74 (m, 1H), 1.74 – 1.60 (m, 3H), 1.54 (dt, J = 6.6, 2.7 Hz, 6H), 1.44 – 1.13 (m, 7H), 1.13 – 0.97 (m, 1H)
**$^{13}$C NMR** (75 MHz, $CDCl_3$) δ 83.5 (C), 69.3 (CH), 43.1 (CH), 40.3 (CH), 39.3 (CH), 31.4 ($CH_2$), 30.1 ($CH_2$), 26.7 ($CH_2$), 25.8 ($CH_2$), 25.7 ($CH_2$), 25.7 ($CH_2$), 25.6 ($CH_2$), 25.5 ($CH_2$), 23.1 ($CH_2$).
**HRMS** (GC-Q-TOF) m/z: $[M]^+$ calcd for $C_{14}H_{22}$: 190.1722; found: 190.1724.

***(1S,2S)-1-(3-bromoprop-2-yn-1-yl)-2-cyclopentylcyclohexane* (*2g*)**

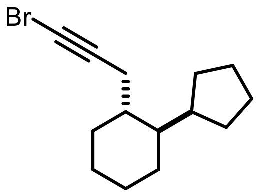

The title compound was synthesized using General Procedure G, starting from 0.46 mmol (87 mg) of alkyne ***2g-CCH***, yielding bromoalkyne ***2g*** as a colorless liquid (99 mg, 0.37 mmol, 80%). The crude was purified by flash column chromatography using n-hexane as eluent.

**$^{1}$H NMR** (300 MHz, $CDCl_3$) δ 2.39 – 2.18 (m, 2H), 2.05 – 2.16 (m, 1H), 1.79 (ddt, $J$ = 9.0, 7.3, 4.5 Hz, 1H), 1.73 – 1.60 (m, 4H), 1.60 – 1.45 (m, 5H), 1.45 – 1.37 (m, 1H), 1.25 (d, $J$ = 6.5 Hz, 6H), 1.12 – 0.97 (m, 1H).

**$^{13}$C NMR** (75 MHz, $CDCl_3$) δ 79.5 (C), 43.3 (CH), 40.4 (CH), 39.5 (CH), 38.2 (C), 31.61 ($CH_2$), 30.2 ($CH_2$), 26.7 ($CH_2$), 25.8 ($CH_2$), 25.7 ($CH_2$), 25.7 ($CH_2$), 25.6 ($CH_2$), 25.5 ($CH_2$), 24.4 ($CH_2$).

**HRMS** (GC-Q-TOF) m/z: $[M]^+$ calcd for $C_{14}H_{21}Br$: 268.0827; found: 268.0824.

5. *5-exo reactivity products* **(5e-g)**

***(3aR,5R,7aS,E)-2-(bromomethylene)-1,1,5-trimethyloctahydro-1H-indene*** **(*5e*)**

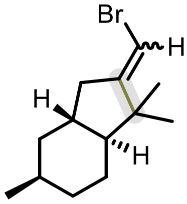


The title compound was synthesized using General Procedure N, starting from bromoalkyne ***2e*** (52 mg, 0.2 mmol) of, yielding bromoalkene ***5e*** in *72% yield as a mixture of isomers E/Z (3.6:1) by NMR analysis of the crude reaction mixture using $CH_2Br_2$ as internal standard*. The crude was purified by column chromatography using n-hexane as eluent to afford the named compound as a colorless liquid (46 mg, 0.18 mmol, 88%).

**$^{1}$H NMR** (300 MHz, $CDCl_3$) δ = 5.90 (d, $J$ = 2.9 Hz, 1H), 2.67 – 2.45 (m, 1H), 2.05 (dq, $J$ = 7.2, 2.5 Hz, 1H), 1.91 – 1.65 (m, 4H), 1.65 – 1.16 (m, 8H), 1.16 – 0.77 (m, 14H).

**$^{13}$C NMR** (75 MHz, $CDCl_3$) δ = 158.4 (C), 98.7 (CH), 57.8 (CH), 46.0 (C), 39.3 ($CH_2$), 38.1 ($CH_2$), 34.0 (CH), 31.9 ($CH_2$), 28.2 (CH), 26.9 ($CH_3$), 24.2 ($CH_3$), 20.7 ($CH_2$), 18.7 ($CH_3$).

**HRMS** (GC-Q-TOF) m/z: $[M]^+$ calcd for $C_{13}H_{21}Br$: 256.0827; found: 256.0826.

***(3aS,7aR,Z)-2-(bromomethylene)-1,1-dimethyloctahydro-1H-indene (5f)***

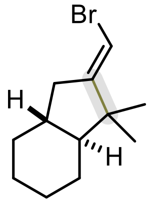


The title compound was synthesized using General Procedure N, starting from bromoalkyne ***2f*** (60 mg, 0.23 mmol) of, yielding bromoalkene ***5f*** in *86% yield as a mixture of isomers E/Z (5.7:1) by NMR analysis of the crude reaction mixture using $CH_2Br_2$ as internal standard*. The crude was purified by column chromatography using n-hexane as eluent to afford the named compound as a colorless liquid (52 mg, 0.20 mmol, 88%).

**$^{1}$H NMR** (300 MHz, $CDCl_3$) δ = 5.90 (dd, $J$ = 3.2, 2.1 Hz, 1H), 2.60 (ddd, $J$ = 17.3, 7.3, 2.2 Hz, 1H), 2.04 – 1.39 (m, 7H), 1.30 – 1.15 (m, 2H), 1.07 (d, $J$ = 9.1 Hz, 7H), 0.87 (s, 3H).**$^{13}$C NMR** (75 MHz, $CDCl_3$, ***major***) δ = 158.2 (C), 98.5 (CH), 57.1 (CH), 45.9 (C),

40.2 (CH), 39.0 ($CH_2$), 32.3 ($CH_2$), 26.7 ($CH_3$), 26.4 ($CH_2$), 26.2 ($CH_2$), 26.0 ($CH_2$), 24.0 ($CH_3$).
**$^{13}$C NMR** (75 MHz, $CDCl_3$, ***minor***) δ = 154.6 (C), 95.8 (CH), 58.3 (CH), 45.0 (C), 42.3 ($CH_2$), 41.2 (CH), 32.2 ($CH_2$), 26.4 ($CH_2$), 25.8 ($CH_2$), 25.0 ($CH_3$), 19.4 ($CH_3$).
**HRMS** (GC-Q-TOF) m/z: $[M]^+$ calcd for $C_{12}H_{19}Br$: 242.0670; found: 242.0666.

### *(3a'S,7a'R,E)-2'-(bromomethylene)octahydrospiro[cyclopentane-1,1'-indene]* (**5g**)

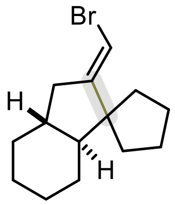


The title compound was synthesized using General Procedure N, starting from bromoalkyne ***2g*** (54 mg, 0.2 mmol) of, yielding bromoalkene ***5g*** in *61% yield as a mixture of isomers E/Z (3:1) by NMR analysis of the crude reaction mixture using $CH_2Br_2$ as internal standard*. The crude was purified by column chromatography using n-hexane as eluent to afford the product as a colorless liquid (32 mg, 0.12 mmol, 60%).
**$^{1}$H NMR** (300 MHz, $CDCl_3$) δ = 5.89 (t, *J* = 2.8 Hz, 1H), 2.56 (ddd, *J* = 17.5, 7.6, 2.3 Hz, 1H), 1.92 (t, *J* = 3.0 Hz, 1H), 1.82 (d, *J* = 3.2 Hz, 2H), 1.73 (d, *J* = 3.2 Hz, 2H), 1.65 (m, 7H), 1.54 (dd, *J* = 10.6, 3.3 Hz, 1H), 1.41 (ddd, *J* = 11.0, 7.6, 3.5 Hz, 1H), 1.26 – 1.14 (m, 4H), 1.01 (d, *J* = 11.6 Hz, 1H).
**$^{13}$C NMR** (75 MHz, $CDCl_3$, ***major***) δ = 159.4 (C), 97.6 (CH), 57.7 (C), 55.9 (CH), 41.2 (CH), 39.3 ($CH_2$), 36.4 ($CH_2$), 34.3 ($CH_2$), 32.3 ($CH_2$), 26.4 ($CH_2$), 26.2 ($CH_2$), 26.0 ($CH_2$), 25.8 ($CH_2$), 25.8 ($CH_2$).
**HRMS** (GC-Q-TOF) m/z: $[M]^+$ calcd for $C_{14}H_{21}Br$: 268.0827; found: 268.0824.

## *2. Appendix*

*X-Ray data*

## 10k (X-ray data)

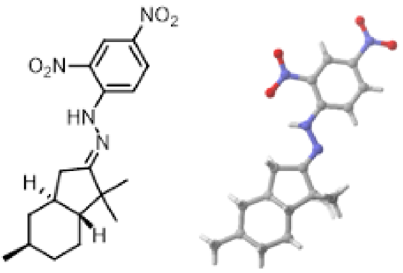


| | |
|---|---|
| Crystal data | |
| Chemical formula | $C_{18}H_{24}N_4O_4$ |
| $M_r$ | 360.41 |
| Crystal system, space group | Monoclinic, $P2_1$ |
| Temperature (K) | 297 |
| *a*, *b*, *c* (Å) | 7.718 (2), 7.074 (2), 17.706 (8) |
| $\beta$ (°) | 99.78 (4) |
| $V$ (Å$^3$) | 952.6 (6) |
| $Z$ | 2 |
| Radiation type | Cu $K\alpha$ |
| $\mu$ (mm$^{-1}$) | 0.75 |
| Crystal size (mm) | 0.52 × 0.38 × 0.05 |
| | |
| Data collection | |
| Diffractometer | Xcalibur, Onyx, Nova |
| Absorption correction | Multi-scan<br>*CrysAlis PRO* 1.171.38.46 (Rigaku Oxford Diffraction, 2015) Empirical absorption correction using spherical harmonics, implemented in SCALE3 ABSPACK scaling algorithm. |
| $T_{min}$, $T_{max}$ | 0.384, 1.000 |
| No. of measured, independent and observed [$I > 2\sigma(I)$] reflections | 3114, 2000, 1132 |
| $R_{int}$ | 0.054 |
| $(\sin \theta/\lambda)_{max}$ (Å$^{-1}$) | 0.581 |
| | |
| Refinement | |
| $R[F^2 > 2\sigma(F^2)]$, $wR(F^2)$, $S$ | 0.100, 0.342, 1.16 |
| No. of reflections | 2000 |
| No. of parameters | 238 |
| No. of restraints | 1 |
| H-atom treatment | H-atom parameters constrained |
| $\Delta\rho_{max}$, $\Delta\rho_{min}$ (e Å$^{-3}$) | 0.36, −0.21 |
| Absolute structure | Flack x determined using 141 quotients [(I+)-(I-)]/[(I+)+(I-)] (Parsons, Flack and Wagner, Acta Cryst. B69 (2013) 249-259). |
| Absolute structure parameter | −2.0 (10) |

Computer programs: *CrysAlis PRO* 1.171.38.43 (Rigaku OD, 2015), *SHELXT* (Sheldrick, 2015), *SHELXL2019*/2 (Sheldrick, 2019), *PLATON* (Spek, 2009), *enCIFer* (Allen,2004).

### Table 2

Hydrogen-bond geometry (Å, º)

| *D*—H···*A* | *D*—H | H···*A* | *D*···*A* | *D*—H···*A* |
|---|---|---|---|---|
| N2—H2···O1 | 0.86 | 1.98 | 2.597 (6) | 127 |
| C17—H17···O2$^{i}$ | 0.93 | 2.50 | 3.105 (9) | 123 |
| C18—H18···O2$^{i}$ | 0.93 | 2.49 | 3.108 (8) | 124 |

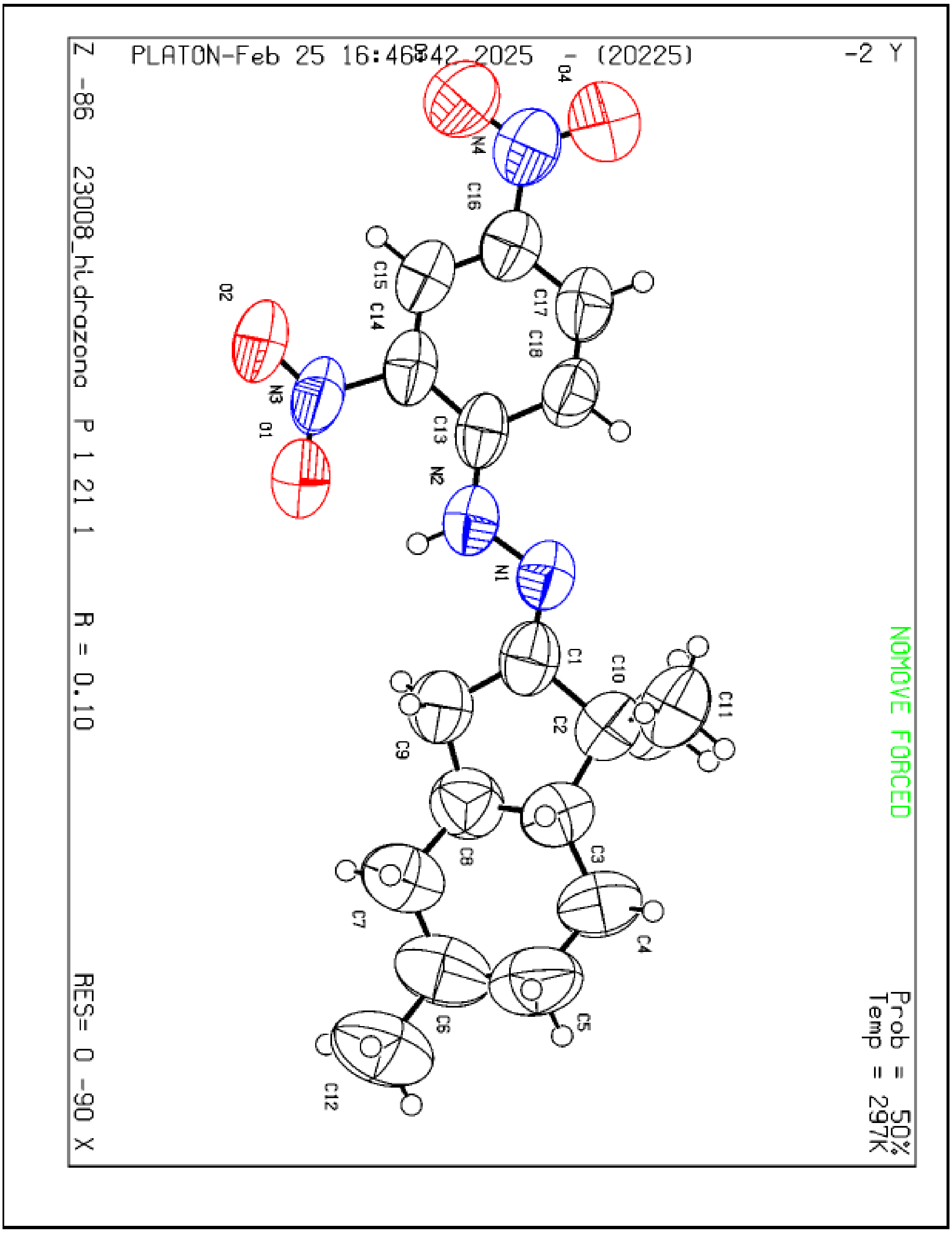


**Figure S1.** ORTEP diagram for compound **9** (50% probability ellipsoid contours).

# *3. References*


[1] Trân, K., Van Den Hauwe, R., X. Sainsily, X., Couvineau, P., Côté, J., L. Simard, L., Echevarria, M., Murza, A., Serre, A., Théroux, L., Saibi, S., Haroune, L., Longpré, J. M., Lesur, O., Auger-Messier, M., Spino, C., Bouvier, M., Sarret, P., Ballet, S., Marsault, É., *J. Med. Chem.*, **2021**, *64*, 5345–5364.

[2] Miguélez, R., Semleit, N., Rodríguez-Arias, C., Mykhailiuk, P., González, J. M., Haberhauer, G., Barrio, P., *Angew. Chem.*, **2023**, *135*, e202305296; *Angew. Chem. Int. Ed.,* **2023**, *62*, e202305296.

[3] Dillner, D. K., Org. Prep. Proced. Int., 2009, 41, 2, 147–152.

[4] Lv, N., Han, J.-C., Zhang, P., Huang, Y.-R., Xu, Z.-X., Xu, K., Wang, X.-F., Li, X., Chung, L. W., *Chem.*, **2024**, *10*, 1, 190–198.

[5] Das, J., Ali, W., Ghosh, A., Pal, T., Mandal, A., Teja, C., Dutta, S., Pothikumar, R., Ge, H., Zhang, X., Maiti, D., *Nature Chem.*, **2023**, *15*, 11, 1626–1635.

[6] Menichincheri, M., Bargiotti, A., Berthelsen, J., Bertrand, J. A., Bossi, R., Ciavolella, A., Cirla, A., Cristiani, C., Croci, V., D'Alessio, R., Fasolini, M., Fiorentini, F., Forte, B., Isacchi, A., Martina, K., Molinari, A., Montagnoli, A., Orsini, P., Orzi, F., Vanotti, E., et al., *J. Med. Chem.*, **2009**, *52*, 2, 293–307.

[7] Chen, C. M., Shiao, H. Y., Uang, B. J., Hsieh, H. P., *Angew. Chem. Int. Ed.*, **2018**, *57*, 47, 15572–15576.

[8] Kuhwald, C., Kirschning, A., *Org. Lett.*, **2021**, *23*, 4300–4304.

[9] Moebius, H.-J., WO 2004/037234 A2, 2004.

[10] Engel, D. A., Lopez, S. S., Dudley. G. B., *Tetrahedron*, **2008**, *64*, 29, 6988–6996.

[11] a) Lonardi, G., Parolin, R., Licini, G., Orlandi, M., *Angew. Chem. Int. Ed.*, **2023**, *62*, e202216649.; b) Deng, J., Hu, X., Huang, J., Yu, S., Wang, D., Duan, Z., Zheng, Z., *J. Org. Chem.*, **2008**, *73*, 6022–6024.

[12] Wei, Y., Rao, B., Cong, X., Zeng, X., *J. Am. Chem. Soc.*, **2015**, *137*, 29, 9250–9253.

[13] Harrowven, D. C.; Pascoe, D. D.; Demurtas, D.; Bourne, H. O. *Angew. Chem. Int. Ed.* **2005**, *44*, 8, 1221–1222.

[14] Schoenauer, S., Schieberle, P., *J. Agric. Food Chem.*, **2016**, *64*, 3849–3861.

[15] Malosh, C. F., Ready, J. M., *J. Am. Chem. Soc.*, **2004**, *126*, 10240–10241.

[16] Capacci, A. G., Malinowski, J. T., McAlpine, N. J., Kuhne, J., Macmillan, D. W. C., *Nat. Chem.*, **2017**, *9*, 1073–1077.

[17] Patel, N. C., Schwarz, J. B., Islam, K., Miller, W., Tran, T. P., Wei, Y., *Synth. Commun.*, **2011**, *41*, 2209–2215.

[18] Miguélez, R., Arto, O., Rodríguez-Arias, C., del Blanco, Á., Barrio, P., González, J. M., *Eur. J. Org. Chem.*, **2024**, *27*, e202301274.

[19] Chai, Z., Zeng, T. T., Li, Q., Lu, L. Q., Xiao, W. J., Xu, D., *J. Am. Chem. Soc.*, **2016**, *138*, 32, 10128–10131.
