# Supplementary material for: Terpenes as Naturally Occurring Stereochemical Templates: Conformationally Driven Discovery of Reactivity
Source: Org Lett. 2025 Sep 23;27(39):11071–6. doi: 10.1021/acs.orglett.5c03431 (PMC12501937; doi:10.1021/acs.orglett.5c03431)
Supplement: Supplementary file 2 [file ol5c03431_si_002.pdf]

Supporting Information

Terpenes as Naturally Occurring Stereochemical Templates: Conformationally-Driven Discovery of Reactivity

Omar Arto,[a‡] Rubén Miguélez,[a,‡] Hannah Siera,[b] Jan Schulte,[b] Isabel Merino,[c] Gebhard Haberhauer,[b] Pablo Barrio[a*]

[a] Department of Organic and Inorganic Chemistry

Universidad de Oviedo

Julian Clavería 8 33006 Oviedo (Spain)

[b] Institut für Organische Chemie

Universität

Duisburg-Essen

Universitätstraße 7, 45117 Essen (Germany)

[c] Servicios Científico Técnicos

Universidad de Oviedo

Correspondence to: barriopablo@uniovi.es

This PDF file includes:

Figures S1-S2
Computational Details
Cartesian coordinates and Absolute Energies
References

1. **Figures** S3
2. **Computational Details** S4
3. **Cartesian Coordinates and Absolute Energies for All Calculated Compounds** S5
4. **References** S36

1. Figures

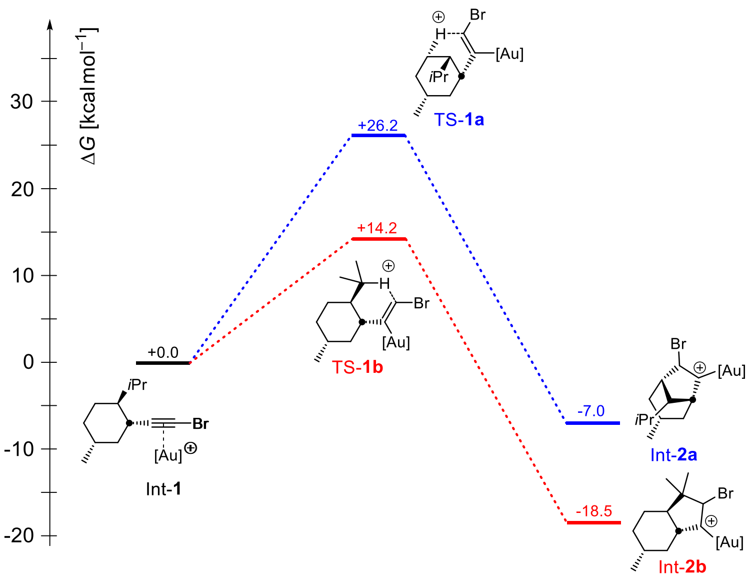

Figure S1. Free energy (Δ*G*) profile of the gold(I)-catalyzed reaction of alkyne **1a** calculated by means of PBE0-D3BJ(SMD)/6-311++G(d,p),def2-TZVP//PBE0-D3BJ/6-31G*,def2-TZVP. $[Au]^+ = IPrAu^+$.

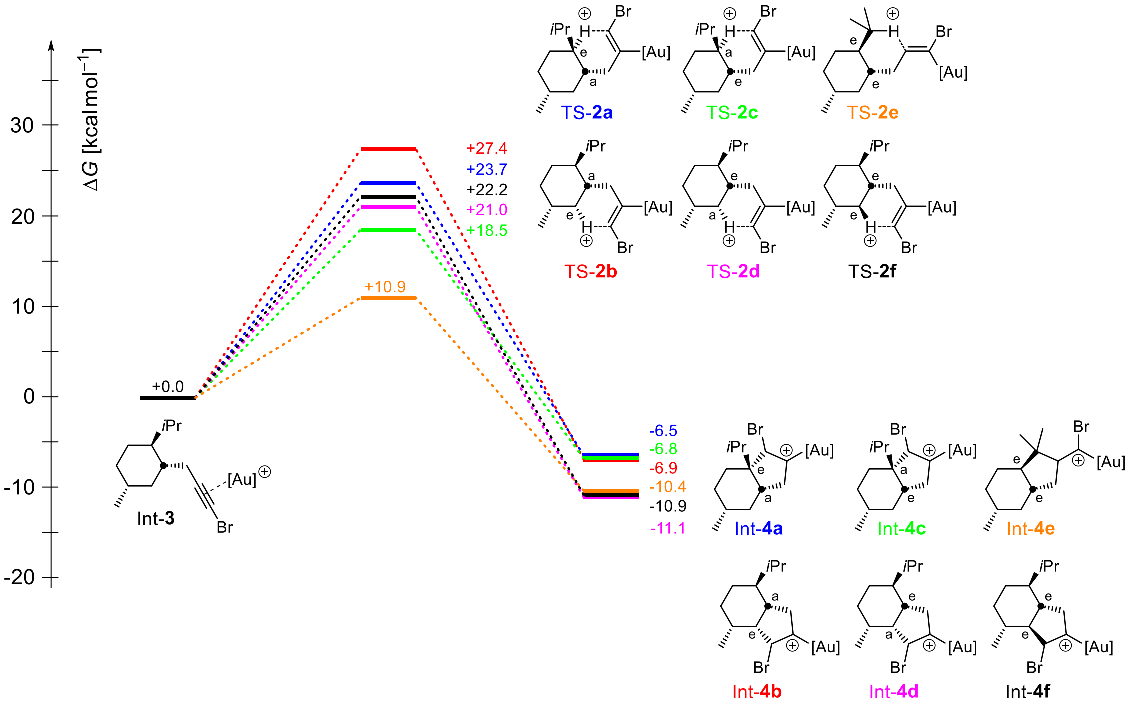

Figure S2. Free energy (Δ*G*) profile of the gold(I)-catalyzed reaction of alkyne **2a** calculated by means of PBE0-D3BJ(SMD)/6-311++G(d,p),def2-TZVP//PBE0-D3BJ/6-31G*,def2-TZVP. $[Au]^+ = IPrAu^+$.

2. Computational Details

All calculations were performed by using the program package Gaussian 16[1]. The geometrical parameters of the stationary points for the gold(I)-catalyzed cyclization were optimized by means of the density functional PBE0[2] and the dispersion correction D3BJ[3]. For all structures C_1 symmetry was applied. For the optimization process two different types of basis sets were used: For the light atoms C, H, N and Br the 6-31G(d)[4-5] basis set was employed. For Au the def2-TZVP[6-7] basis set was used. Frequency calculations were carried out at each of the structures to verify the nature of the stationary point. It turned out that all transition states have exactly one imaginary frequency. All other structures have none.

Furthermore, the energies of the stationary points were calculated using the density functional PBE0, the additional dispersion correction D3BJ and the basis set 6-311++G(d,p)[8-11] for C, H, N and Br. For Au the def2-TZVP basis set was employed. To take solvent effects into account, the solvent model SMD[12] (dichloromethane as solvent) was used for the single point calculations.

3. Cartesian Coordinates and Absolute Energies for All Calculated Compounds

Table S1. Absolute energies [au] calculated by means of different methods.

Compound	E^{a}	G^{a}	E^{b}
**1a**	-3039.189690	-3038.945932	-3042.067056
Int-**1**	-4333.595022	-4332.817305	-4336.816574
TS-**1**a	-4333.547987	-4332.770143	-4336.775011
Int-**2**a	-4333.611394	-4332.827636	-4336.833719
TS-**1**b	-4333.570417	-4332.795233	-4336.791443
Int-**2**b	-4333.623925	-4332.844577	-4336.847756

[a] PBE0-D3BJ/6-31G*,def2-TZVP

[b] PBE0-D3BJ(SMD)/6-311++G(d,p),def2-TZVP//PBE0-D3BJ/6-31G*,def2-TZVP

Table S2. Absolute energies [au] calculated by means of different methods.

Compound	E^{a}	G^{a}	E^{b}
**2a**	-3078.460889	-3078.188931	-3081.349767
Int-**3**	-4372.861763	-4372.056725	-4376.095955
TS-**2**a	-4372.826154	-4372.022310	-4376.057074
Int-**4**a	-4372.879532	-4372.069543	-4376.111255
TS-**2**b	-4372.814843	-4372.010268	-4376.051779
Int-**4**b	-4372.877439	-4372.067889	-4376.111469
TS-**2**c	-4372.832571	-4372.029967	-4376.063985
Int-**4**c	-4372.879026	-4372.069520	-4376.111313
TS-**2**d	-4372.822650	-4372.020495	-4376.059587
Int-**4**d	-4372.881327	-4372.074757	-4376.115173
TS-**2**e	-4372.843207	-4372.040057	-4376.076670
Int-**4**e	-4372.892033	-4372.079176	-4376.120302
TS-**2**f	-4372.822559	-4372.019580	-4376.058595
Int-**4**f	-4372.882029	-4372.074826	-4376.115519

[a] PBE0-D3BJ/6-31G*,def2-TZVP

[b] PBE0-D3BJ(SMD)/6-311++G(d,p),def2-TZVP//PBE0-D3BJ/6-31G*,def2-TZVP

Cartesian coordinates of the optimized geometry for **1a** at PBE0-D3BJ/6-31G*,def2-TZVP level of theory: (number of imaginary frequencies = 0):

C	-0.70089800	-0.22382800	0.57552000
C	-1.42705900	0.91785100	-0.18531300
C	-1.21865900	-1.62654000	0.18870700
H	-0.89258800	-0.07534800	1.65092200
C	-2.94564300	0.68357700	-0.13782900
H	-1.09756200	0.86819800	-1.23532100
C	-2.72743000	-1.78135600	0.31482700
H	-0.92660500	-1.82595300	-0.85240000
H	-0.69772300	-2.36994900	0.80406200
C	-3.39269700	-0.71396000	-0.54295900
H	-3.44396100	1.41538300	-0.78046800
H	-3.29440700	0.88491400	0.88733500
H	-3.00885500	-1.59992800	1.36481000
H	-4.48558400	-0.78825000	-0.46899300
H	-3.13693000	-0.89665600	-1.59793400
C	0.74441700	-0.25373000	0.36142100
C	1.92797700	-0.37405800	0.14710800
Br	3.67998600	-0.51402100	-0.16685100
C	-3.17044700	-3.18693700	-0.06915300
H	-2.69311000	-3.94429700	0.56327500
H	-4.25628800	-3.30022100	0.02867400
H	-2.90444200	-3.40623500	-1.11095900
C	-1.11066200	2.33257800	0.35720500
H	-1.51655800	2.37385500	1.38118100
C	0.37584100	2.67896900	0.43874100
H	0.91776500	2.05546400	1.15225700
H	0.86468100	2.56573000	-0.53617900

H	0.49290400	3.72296200	0.75234900
C	-1.81535300	3.41428000	-0.46738400
H	-2.90573300	3.35484300	-0.41334900
H	-1.52695100	4.40928500	-0.11114800
H	-1.52388100	3.34588600	-1.52365800

Cartesian coordinates of the optimized geometry for **3a** at PBE0-D3BJ/6-31G*,def2-TZVP level of theory: (number of imaginary frequencies = 0):

C	-1.48147600	-0.78652300	-0.48247800
C	-2.64187900	-0.56015000	-0.23223100
Br	-4.35677900	-0.22567600	0.13602100
C	-0.07801500	-1.05610500	-0.76268100
H	0.09733100	-0.98403000	-1.84646900
H	0.13352600	-2.09567200	-0.48502500
C	0.88543700	-0.10207500	-0.03330800
C	0.55662000	1.35375800	-0.36883500
C	2.35957800	-0.40899300	-0.35774000
H	0.73130000	-0.23835700	1.04750700
C	1.45422100	2.34815300	0.36224700
H	0.66790100	1.50297500	-1.45534800
H	-0.49587100	1.55429700	-0.13389600
C	3.25955700	0.57928300	0.39344500
H	2.49257700	-0.20989900	-1.43577200
C	2.91797700	2.02978600	0.07029300
H	1.29049800	2.21379900	1.44374500
H	3.15494700	0.41838400	1.47608100
H	3.57246100	2.70718700	0.63453500
H	3.11539300	2.22113100	-0.99594500
C	2.76378300	-1.88299300	-0.13911500
H	2.13033800	-2.48838000	-0.80261900
C	2.53645600	-2.37653200	1.28885000
H	2.81702600	-3.43218000	1.37777900
H	1.48919700	-2.28688300	1.59790000
H	3.14542700	-1.81534900	2.00709100
C	4.20573500	-2.14889900	-0.57054900
H	4.40529400	-1.74628600	-1.57099900
H	4.40608100	-3.22599300	-0.59623800
H	4.92736100	-1.70120100	0.12197600
H	4.31098100	0.38995300	0.14997300
C	1.10403800	3.78525600	-0.00075400
H	1.73419600	4.49842000	0.54338400
H	0.05737900	4.01194000	0.23273600
H	1.25173800	3.96100200	-1.07399900

Cartesian coordinates of the optimized geometry for Int-**1** at PBE0-D3BJ/6-31G*,def2-TZVP level of theory: (number of imaginary frequencies = 0):

Au	0.19800700	-0.36034900	-0.23355700
C	-1.64339700	-1.51765400	-0.56302300
C	1.39545400	1.12048600	0.40854100
C	3.18365900	2.28752800	1.09096100
C	2.09636400	3.09316200	1.21382100
N	2.72764100	1.08154300	0.59609700
N	1.00954500	2.35493400	0.78721700
C	-0.35955900	2.78509300	0.74917700
C	-0.82351100	3.41584600	-0.41361600
C	-1.17359600	2.50198100	1.85558300
C	-2.17114000	3.77955500	-0.44292000
C	-2.51574700	2.87728100	1.76803900
C	-3.00847200	3.51192800	0.63370800
H	-2.56996500	4.27762200	-1.32172800
H	-3.18032200	2.67907000	2.60423900
H	-4.05351400	3.80487900	0.58912700
C	3.51936400	-0.08254800	0.31268300
C	4.06407900	-0.21475400	-0.97200900
C	3.65834800	-1.04847900	1.31897300
C	4.79075400	-1.37700500	-1.23472500
C	4.39594600	-2.19037600	1.00212400
C	4.95696600	-2.35227000	-0.25875200
H	5.22901600	-1.52111900	-2.21795100
H	4.52970500	-2.96371100	1.75307500
H	5.52797000	-3.24840800	-0.48374100
C	-0.64781000	1.82135600	3.10378700
H	0.42892100	1.65850100	2.97942000
C	-1.29537800	0.44872800	3.30143200
H	-2.38153800	0.53252800	3.42277700
H	-1.09798100	-0.20755400	2.44604900
H	-0.89592400	-0.03788400	4.19770800
C	-0.83682100	2.70646600	4.33806800
H	-0.39520100	2.22829700	5.21871900
H	-0.36145400	3.68363800	4.20470600
H	-1.89774500	2.87734000	4.55057500
C	3.02714900	-0.89800800	2.68831500
H	2.52767000	0.07688700	2.73258700
C	4.08274100	-0.92113600	3.79592200
H	3.61301700	-0.75992100	4.77205000
H	4.60257700	-1.88457900	3.83348000
H	4.83618800	-0.14102400	3.64556900
C	1.95812800	-1.97008600	2.91673000
H	1.18589900	-1.93342900	2.13932100
H	2.39410900	-2.97504900	2.90709800
H	1.47273200	-1.82485700	3.88805700
C	3.84861300	0.82182300	-2.05612500
H	3.35976300	1.69406100	-1.60645300
C	0.06754500	3.66743000	-1.61306200
H	1.09693800	3.41253600	-1.33579400
C	-0.33125300	2.75735400	-2.77856100
H	-0.26987300	1.69982200	-2.49713700
H	-1.35795600	2.95944000	-3.10495100
H	0.33163900	2.92018700	-3.63528700
C	0.05757100	5.13935100	-2.02953900
H	0.76192100	5.30310700	-2.85175800
H	-0.93212300	5.45401400	-2.37780000
H	0.34367800	5.79251300	-1.19875600
C	2.91029500	0.27738000	-3.13726800
H	3.34564600	-0.59755200	-3.63273200
H	1.94610200	-0.02438400	-2.71145800
H	2.72233800	1.03929000	-3.90167100

C	5.17047800	1.30101700	-2.65846400
H	5.69454900	0.49141200	-3.17760300
H	4.98637600	2.09493400	-3.38989800
H	5.84165900	1.69427900	-1.88804800
C	-0.69653300	-2.16554400	-1.03697900
Br	0.32790800	-3.45697200	-1.75189100
H	1.99287700	4.10975500	1.55967100
H	4.22765400	2.45513400	1.30484000
C	-2.93315300	-0.90167400	-0.21985000
C	-3.24862900	0.09590400	-1.35685700
C	-4.02521000	-1.98341100	0.00640600
H	-2.82159200	-0.33190200	0.71371000
C	-4.62533500	0.73115800	-1.23136700
H	-3.18650900	-0.43327200	-2.31808800
H	-2.46998600	0.86896700	-1.36531600
C	-5.40800300	-1.31080200	0.00226100
H	-3.97421900	-2.68498600	-0.84075400
C	-5.66759500	-0.37698500	-1.17095700
H	-4.65889000	1.28784200	-0.28172600
H	-6.17732900	-2.08765000	0.02364200
H	-5.51635200	-0.74232200	0.93853500
H	-6.67392700	0.05015600	-1.08289700
H	-5.64861500	-0.94095900	-2.11539300
C	-3.84564500	-2.77720800	1.32330800
H	-4.08108700	-2.07736300	2.14105900
C	-4.83751700	-3.94066600	1.39700800
H	-5.88066500	-3.61623400	1.36675600
H	-4.67556900	-4.63932200	0.56651000
H	-4.69954400	-4.49910000	2.32856500
C	-2.44102500	-3.31914600	1.58849000
H	-1.69464100	-2.52901000	1.71856600
H	-2.44675800	-3.90636100	2.51308300
H	-2.10226200	-3.98157100	0.78275800
C	-4.87450500	1.70466700	-2.37495200
H	-4.11429100	2.49406900	-2.39790700
H	-4.84865100	1.18717000	-3.34171000
H	-5.85583700	2.18187800	-2.27952600

Cartesian coordinates of the optimized geometry for Int-**2a** at PBE0-D3BJ/6-31G*,def2-TZVP level of theory: (number of imaginary frequencies = 0):

Au	0.03805900	-0.24237800	0.14943700
C	1.94020600	-0.79876100	0.14496600
C	-1.88629400	0.44038600	0.19415800
C	-3.62928600	1.86115600	0.26264800
C	-4.12468100	0.59800500	0.32593700
N	-2.25657400	1.73856000	0.18278100
N	-3.03861700	-0.25336200	0.28210400
C	-3.09567000	-1.68662100	0.32910800
C	-3.18976500	-2.38489000	-0.88199900
C	-3.02317700	-2.31154800	1.58167700
C	-3.21208800	-3.77876800	-0.80952100
C	-3.04854300	-3.70688000	1.59631800
C	-3.14269400	-4.43235300	0.41464700
H	-3.28233900	-4.35889300	-1.72513900
H	-2.99338300	-4.23171600	2.54573300
H	-3.16258600	-5.51783100	0.44841000
C	-1.31350700	2.81633100	0.09148500
C	-0.99503800	3.31057100	-1.18132100
C	-0.72867100	3.28865000	1.27485700
C	-0.04318300	4.32955000	-1.24616000
C	0.22162600	4.30450700	1.15209700
C	0.55869000	4.82112900	-0.09343800
H	0.23335100	4.74049300	-2.21253800
H	0.69516800	4.70267500	2.04517500

H	1.29448400	5.61701100	-0.16666200
C	-2.89411800	-1.52948900	2.87336800
H	-2.99742100	-0.46313300	2.64117900
C	-1.50718200	-1.73054200	3.49009900
H	-1.34146700	-2.78091500	3.75485000
H	-0.71852600	-1.43108500	2.78945600
H	-1.40244300	-1.13227000	4.40214600
C	-4.00076400	-1.88854300	3.86664700
H	-3.92170600	-1.26514200	4.76366900
H	-4.99331000	-1.73601600	3.43041900
H	-3.93277000	-2.93433900	4.18515100
C	-1.09334300	2.74033100	2.63993800
H	-1.87594000	1.98454000	2.50874000
C	-1.66114500	3.83657100	3.54434500
H	-1.97368500	3.41284500	4.50467700
H	-0.91584500	4.61249000	3.75054500
H	-2.52916700	4.32121300	3.08571200
C	0.10338900	2.04565100	3.29385300
H	0.46828600	1.22441600	2.66551100
H	0.93092600	2.74569100	3.45649000
H	-0.18006400	1.62986900	4.26689300
C	-1.61508100	2.74949700	-2.44545900
H	-2.45290100	2.10368800	-2.15818800
C	-3.23832200	-1.68291700	-2.22378000
H	-3.28340000	-0.60233200	-2.04522000
C	-1.96357900	-1.96011400	-3.02434600
H	-1.06974000	-1.65081500	-2.47192300
H	-1.86356400	-3.02735700	-3.25219200
H	-1.98354000	-1.41476700	-3.97454900
C	-4.48949800	-2.06784500	-3.01561000
H	-4.53101600	-1.50577000	-3.95466500
H	-4.49279100	-3.13359400	-3.26874800
H	-5.40256000	-1.85619900	-2.44937600
C	-0.60453400	1.87959700	-3.19916200
H	0.27131500	2.46594200	-3.50002600
H	-0.25353500	1.04568800	-2.58143000
H	-1.05985100	1.46306700	-4.10453400
C	-2.17837300	3.85006300	-3.34583800
H	-1.38716300	4.49687400	-3.74003800
H	-2.69368600	3.40569100	-4.20373200
H	-2.89225800	4.48237600	-2.80797900
C	2.93512900	-0.53619800	1.19628200
C	4.15330900	-1.43743700	0.89250500
H	3.87423400	-2.47301400	1.14692700
C	4.17828000	-1.30743000	-0.64079800
H	2.44167500	-2.56940700	-0.60657400
C	2.67914800	-1.52712900	-0.90458700
Br	2.05151000	-1.40018400	-2.72546000
H	-5.13497100	0.22644800	0.39685600
H	-4.11634900	2.82375500	0.26431200
H	4.76384900	-2.08982900	-1.12984800
C	4.67459600	0.07053900	-1.07676600
C	3.34348500	0.96700900	0.94410600
C	3.91889600	1.26999500	-0.45165200
H	4.65272200	2.07328800	-0.31175500
H	5.73794000	0.12527800	-0.81957700
H	4.62405000	0.14171500	-2.16920400
H	2.48210800	1.61167600	1.14644800
H	2.51936700	-0.58978100	2.20654000
C	5.44876900	-1.13329500	1.64798800
H	5.78312100	-0.11701300	1.40166900
C	5.22203100	-1.20601200	3.15676200
H	4.49503800	-0.46344000	3.50585300
H	4.85581300	-2.19779000	3.44978300
H	6.15742400	-1.02640700	3.69618700
C	6.55052500	-2.10075200	1.22070400

H	7.47239100	-1.90458800	1.77744900
H	6.25813700	-3.13948800	1.41827300
H	6.78425200	-2.01386200	0.15405600
H	4.08299900	1.16330000	1.72765300
C	2.84024800	1.83146100	-1.37635300
H	2.02680200	1.12056000	-1.56119300
H	2.38848800	2.73010700	-0.94399000
H	3.26214800	2.09315400	-2.35253300

Cartesian coordinates of the optimized geometry for Int-**2b** at PBE0-D3BJ/6-31G*,def2-TZVP level of theory: (number of imaginary frequencies = 0):

Au	-0.04765400	-0.09599400	-0.16596200
C	-1.73515400	-1.10013000	-0.34886400
C	1.64172600	0.99532400	0.12777100
C	3.76928000	1.60615500	0.50524900
C	3.00808400	2.73445100	0.52451800
N	2.91022800	0.55670100	0.26099900
N	1.71006200	2.33410100	0.29189700
C	0.54963300	3.17785900	0.23059400
C	0.17869200	3.70498100	-1.01407400
C	-0.18884900	3.37679800	1.40534100
C	-0.98504000	4.47475300	-1.05754900
C	-1.34336500	4.15614300	1.30630100
C	-1.73646600	4.70106100	0.08944900
H	-1.30760800	4.90033900	-2.00331300
H	-1.94062600	4.33904700	2.19517500
H	-2.63516700	5.30905500	0.03477900
C	3.26204000	-0.83329300	0.16993600
C	3.58194900	-1.35356600	-1.09115100
C	3.20474100	-1.60779900	1.33617400
C	3.86356800	-2.71855700	-1.16116700
C	3.49595200	-2.96699800	1.20950000
C	3.82198800	-3.51644800	-0.02426600
H	4.11214600	-3.16339700	-2.12023900
H	3.46195000	-3.60334800	2.08914900
H	4.04455500	-4.57681600	-0.10114300
C	0.20892900	2.76532800	2.73333700
H	1.14916800	2.21975100	2.59364900
C	-0.84002800	1.75321400	3.20172300
H	-1.80930400	2.23529600	3.37269400
H	-0.98080900	0.96142600	2.45619200
H	-0.52757700	1.28494400	4.14139600
C	0.45546300	3.84125900	3.79306700
H	0.79214500	3.38277100	4.72892400
H	1.21994100	4.55402300	3.46704200
H	-0.45723900	4.40687200	4.01027400
C	2.81298100	-1.03001800	2.68092900
H	2.68955300	0.05345600	2.56802900
C	3.90165200	-1.25774100	3.73144300
H	3.62041000	-0.78455400	4.67831800
H	4.05327700	-2.32468700	3.92761800
H	4.86052400	-0.83775000	3.41042200
C	1.46770100	-1.59834300	3.14075100
H	0.68480000	-1.40948600	2.39707600
H	1.52625200	-2.68189200	3.29207700
H	1.16324200	-1.14134500	4.08896800
C	3.57866800	-0.50241700	-2.34459600
H	3.45279500	0.54571400	-2.04869900
C	0.96356000	3.42693300	-2.27973800
H	1.88035900	2.89242200	-2.00564000
C	0.16352000	2.51425400	-3.21356600
H	-0.10050700	1.57410600	-2.71511400
H	-0.76569500	2.99765100	-3.53599200
H	0.74735800	2.27509400	-4.10912800

C	1.38107400	4.71792200	-2.98604900
H	1.99818800	4.48731000	-3.86080200
H	0.51225600	5.28536900	-3.33682400
H	1.95924500	5.36818200	-2.32150300
C	2.38925900	-0.87350800	-3.23503400
H	2.46252100	-1.91239700	-3.57565800
H	1.44261500	-0.76537400	-2.69246400
H	2.35510000	-0.22840800	-4.12000300
C	4.89913900	-0.60426900	-3.10960100
H	5.07012400	-1.61830900	-3.48663300
H	4.88876900	0.06954700	-3.97289200
H	5.75018700	-0.33506200	-2.47558600
C	-3.07733800	-0.57473100	-0.12174900
C	-4.08349900	-1.64222100	-0.56240700
H	-4.17000900	-1.56483000	-1.65909500
C	-3.35872100	-2.97287500	-0.25158400
H	-1.89281800	-2.49766500	-1.76195600
C	-1.92038700	-2.53111100	-0.65317700
Br	-0.44407500	-3.67987600	-0.15574000
H	3.26803300	3.77005000	0.67925400
H	4.82898600	1.45602600	0.64107000
H	-3.01316500	-0.66119300	0.99750000
C	-5.45122900	-1.34872200	0.03479400
H	-5.40923400	-1.45178300	1.12770300
H	-6.19672700	-2.06664200	-0.32637900
C	-5.86969200	0.08028200	-0.32838900
C	-3.46310000	0.85968100	-0.43314500
C	-4.86178700	1.14438800	0.12643300
H	-5.99515000	0.15495100	-1.41901100
H	-6.85003300	0.30015500	0.11004500
H	-3.46402200	0.99378700	-1.52418800
H	-2.72639500	1.56213400	-0.02403400
H	-4.79003500	1.08749500	1.22509900
C	-3.83985100	-4.12973500	-1.11662100
H	-4.87120400	-4.39440500	-0.86157600
H	-3.81037300	-3.87329500	-2.18215900
H	-3.21780000	-5.01739100	-0.96035700
C	-3.43249900	-3.36558700	1.22288100
H	-2.81395400	-4.24827200	1.40858300
H	-3.08886700	-2.57766900	1.90281600
H	-4.46324400	-3.61537800	1.49158900
C	-5.31744800	2.54733100	-0.25166300
H	-4.59581900	3.29978600	0.08617300
H	-5.41792300	2.64452400	-1.33968800
H	-6.28882100	2.78335800	0.19524400

Cartesian coordinates of the optimized geometry for Int-**3** at PBE0-D3BJ/6-31G*,def2-TZVP level of theory: (number of imaginary frequencies = 0):

Au	-0.46390100	-0.41272300	0.06513800
C	1.56521700	-1.24352000	0.21210300
C	-1.89172400	1.00146600	0.02363800
C	-3.85122400	2.08537800	-0.06888700
C	-2.86725100	3.02073700	-0.01635200
N	-3.22733100	0.85314700	-0.04248100
N	-1.67183000	2.33101700	0.03916100
C	-0.35595800	2.90175900	0.10664800
C	0.33009000	3.13117900	-1.09468100
C	0.19443000	3.15022300	1.37213600
C	1.62027700	3.65620700	-0.99842400
C	1.48894100	3.67186700	1.41054200
C	2.19164500	3.92896900	0.23918900
H	2.18374900	3.85442800	-1.90538800
H	1.95111600	3.88125300	2.37093200
H	3.19438000	4.34390900	0.29092400

C	-3.86943900	-0.43056700	-0.08525600
C	-4.07542700	-1.02483300	-1.33791800
C	-4.20893400	-1.04055600	1.13013200
C	-4.65713200	-2.29359800	-1.34802100
C	-4.78732200	-2.30893200	1.06125200
C	-5.00958800	-2.92791100	-0.16293300
H	-4.83404000	-2.79143700	-2.29702700
H	-5.06416300	-2.81926700	1.97914400
H	-5.46185800	-3.91494400	-0.19356400
C	-0.54674900	2.84315800	2.65741400
H	-1.55871700	2.50966400	2.40052400
C	0.13464200	1.69713100	3.41058600
H	1.15438500	1.96869400	3.70625300
H	0.19030100	0.79409600	2.79122900
H	-0.42365300	1.45073000	4.32015500
C	-0.68419000	4.08492200	3.54044700
H	-1.27245100	3.85069600	4.43386200
H	-1.18238000	4.90109900	3.00718900
H	0.29249300	4.45106300	3.87494600
C	-3.93717700	-0.38916800	2.47101700
H	-3.57908200	0.63142400	2.29172900
C	-5.20657100	-0.28390700	3.31829400
H	-4.99448800	0.24637300	4.25269300
H	-5.59787300	-1.27220000	3.58248200
H	-5.99679800	0.25821100	2.78880600
C	-2.83052100	-1.13843000	3.21860100
H	-1.90803600	-1.17659000	2.62733100
H	-3.13031800	-2.16911000	3.43825900
H	-2.60686700	-0.64285600	4.16971500
C	-3.66620500	-0.35538200	-2.63440100
H	-3.31407300	0.65688400	-2.40349200
C	-0.26604100	2.81114800	-2.45020800
H	-1.28534400	2.43752400	-2.29900700
C	0.52935500	1.70244000	-3.14489100
H	0.56387100	0.79453200	-2.53104900
H	1.56085200	2.01531300	-3.34188100
H	0.06836600	1.44533100	-4.10462000
C	-0.36059800	4.06081000	-3.32820600
H	-0.84043700	3.81867300	-4.28230500
H	0.63091300	4.47009900	-3.55041500
H	-0.94550400	4.84787300	-2.84113800
C	-2.50326000	-1.10694200	-3.28815000
H	-2.79247700	-2.12984100	-3.55353100
H	-1.64005700	-1.16705200	-2.61484300
H	-2.18586300	-0.59853400	-4.20520500
C	-4.84816000	-0.21761600	-3.59609800
H	-5.22704400	-1.19596600	-3.91068400
H	-4.54143200	0.32262300	-4.49796500
H	-5.67691900	0.32916900	-3.13468400
C	2.67857600	-0.31866300	0.43581900
H	2.46150700	0.62157100	-0.08225000
C	4.01812400	-0.91311500	-0.04114300
H	3.93647100	-1.08318600	-1.12450500
C	4.29327100	-2.25921500	0.63115900
H	3.46661500	-2.95658400	0.43905800
C	0.78714100	-2.18647300	-0.00155800
Br	0.08455200	-3.81604100	-0.28339900
H	-2.90238300	4.09892300	-0.01245200
H	-4.92484000	2.17627300	-0.12192800
H	2.72851400	-0.08178200	1.50791900
C	5.17490900	0.07242900	0.21195900
C	6.48200700	-0.56023200	-0.27978400
C	5.60352500	-2.88998700	0.16400800
C	6.75156200	-1.90817400	0.38012900
H	5.51690600	-3.07387100	-0.91889700
H	7.68654100	-2.33436800	-0.00364800

H	6.89620500	-1.76265000	1.46148600
H	7.31908100	0.11698800	-0.08037600
H	6.43854600	-0.69272100	-1.37015100
H	4.33269500	-2.11396200	1.72249900
H	5.26215800	0.18226900	1.30683500
C	4.92678000	1.48936000	-0.34797600
H	4.04163300	1.88658500	0.16870600
C	6.07316600	2.44188600	-0.01206800
H	6.97204300	2.22266900	-0.59805600
H	5.78926600	3.47705500	-0.23473300
H	6.34200200	2.38888900	1.04948600
C	4.63295200	1.51667600	-1.84698900
H	5.47053400	1.11482800	-2.42782900
H	3.73751100	0.94154500	-2.11091400
H	4.47117900	2.54695600	-2.18411700
C	5.85454100	-4.22131000	0.85940600
H	5.94605600	-4.08319000	1.94398000
H	5.03642400	-4.92832500	0.67973300
H	6.78171600	-4.68361900	0.50388300

Cartesian coordinates of the optimized geometry for Int-**4a** at PBE0-D3BJ/6-31G*,def2-TZVP level of theory: (number of imaginary frequencies = 0):

Au	-0.25484200	-0.23629800	-0.48059700
C	1.56451500	-0.79384300	-0.99690800
C	-2.02988700	0.37023100	0.31123900
C	-3.54898800	1.62746100	1.39026400
C	-4.12181800	0.42105300	1.13087500
N	-2.27027400	1.57154700	0.88114900
N	-3.17288900	-0.32993100	0.47168600
C	-3.33787500	-1.68300200	0.02157100
C	-3.87039200	-1.89409800	-1.25752700
C	-2.92472700	-2.71843300	0.87079500
C	-3.98879700	-3.21758400	-1.68415700
C	-3.06691700	-4.02303200	0.39460700
C	-3.59323500	-4.27017200	-0.86720800
H	-4.39346900	-3.42670500	-2.66984800
H	-2.76180900	-4.85575900	1.02202700
H	-3.69687400	-5.29299100	-1.21778500
C	-1.28884600	2.61768600	0.96218200
C	-1.19359100	3.52304200	-0.10287700
C	-0.45414700	2.65488500	2.08744000
C	-0.21613400	4.51477000	-0.00739400
C	0.50432600	3.66841200	2.13334400
C	0.62039400	4.59069800	1.09985900
H	-0.10730000	5.23498300	-0.81296200
H	1.17221600	3.73221600	2.98747400
H	1.37127200	5.37366700	1.15653900
C	-2.33178900	-2.46365200	2.24228600
H	-2.35235400	-1.38429700	2.43203100
C	-0.86688200	-2.90638000	2.29542000
H	-0.76926600	-3.98360500	2.11980400
H	-0.27455600	-2.38349600	1.53496300
H	-0.43473500	-2.68462800	3.27746300
C	-3.15520500	-3.13733900	3.34201500
H	-2.74301400	-2.89592200	4.32761700
H	-4.19862500	-2.80659900	3.31727400
H	-3.14823500	-4.22775000	3.23763600
C	-0.53644300	1.62786300	3.19873400
H	-1.40507100	0.98582500	3.01164600
C	-0.74092400	2.28379700	4.56539300
H	-0.85788600	1.51801700	5.33958000
H	0.11514300	2.90720800	4.84522700
H	-1.63322100	2.91825400	4.57427700
C	0.70441400	0.73028400	3.19183300

H	0.82004800	0.22854300	2.22430000
H	1.61504700	1.31162400	3.37433900
H	0.62757800	-0.03510500	3.97224900
C	-2.07139800	3.42514200	-1.33350700
H	-2.82278000	2.64622900	-1.15908300
C	-4.25260700	-0.74617900	-2.17022700
H	-4.28076600	0.17224100	-1.57217700
C	-3.18652100	-0.55535200	-3.25373800
H	-2.19908600	-0.37638900	-2.81201600
H	-3.11363300	-1.44453000	-3.89025200
H	-3.43586300	0.29926800	-3.89246200
C	-5.64031500	-0.92907300	-2.78561800
H	-5.91282100	-0.04215200	-3.36714500
H	-5.67363300	-1.78697600	-3.46567000
H	-6.40349200	-1.08087700	-2.01546500
C	-1.24061000	2.99714700	-2.54644500
H	-0.47449800	3.74390300	-2.78320300
H	-0.73051200	2.04513300	-2.35839400
H	-1.88092400	2.87756300	-3.42729400
C	-2.82110500	4.73063500	-1.60429300
H	-2.13254200	5.55041700	-1.83585600
H	-3.49065600	4.61108700	-2.46264600
H	-3.42259400	5.03275800	-0.74069900
C	2.08370400	-2.16468700	-1.15175600
H	1.54261500	-2.69673600	-1.94859500
C	3.60971900	-2.08771600	-1.31194000
H	3.81861900	-2.04446200	-2.38878900
C	3.98317700	-0.70705700	-0.69669300
H	2.76942700	0.25937000	-2.20881700
C	2.72819300	0.09721200	-1.11255400
Br	2.47026900	1.86290000	-0.35150900
H	-5.10441200	0.03285400	1.34944800
H	-3.92790100	2.50885600	1.88371900
H	1.77008600	-2.70288600	-0.24216100
C	4.40164900	-3.26395700	-0.73743900
H	3.96561200	-4.20208600	-1.10549100
H	5.41224100	-3.22086700	-1.16290800
C	4.01032400	-0.83065400	0.83596900
C	4.53797000	-3.30657500	0.79268200
C	4.95518800	-1.92707700	1.31507400
H	5.97802700	-1.70503500	0.98459600
H	4.98078900	-1.93453000	2.41133900
H	4.27052600	0.13419200	1.27996700
H	2.99417600	-1.04478900	1.20010900
H	5.35698900	-4.00632600	1.00330300
C	5.31234800	-0.16599300	-1.29060500
H	5.99632100	-1.02607700	-1.30524700
C	5.98736300	0.92761300	-0.46109500
H	5.33858500	1.80119200	-0.34391300
H	6.90103900	1.25708600	-0.96656200
H	6.27674600	0.58076200	0.53502500
C	5.16756800	0.31868800	-2.73375400
H	4.61471500	1.26616800	-2.77940200
H	4.67039400	-0.40629500	-3.38887100
H	6.15547200	0.50845100	-3.16452600
C	3.31288100	-3.84802500	1.53434500
H	2.93576100	-4.76539300	1.06809100
H	2.48440100	-3.13152800	1.58712100
H	3.57642500	-4.08875100	2.57006000

Cartesian coordinates of the optimized geometry for Int-**4b** at PBE0-D3BJ/6-31G*,def2-TZVP level of theory: (number of imaginary frequencies = 0):

Au	-0.19418100	0.23389000	-0.25862400
C	1.75948700	0.21922900	-0.53603000

C	-2.19532600	0.12834400	0.11075900
C	-4.23974100	-0.72234100	0.49973600
C	-4.33902600	0.63295300	0.56004600
N	-2.91966300	-1.00678400	0.22706500
N	-3.07675500	1.12971400	0.31744800
C	-2.71406000	2.51823100	0.29168100
C	-2.85621800	3.21608900	-0.91569800
C	-2.20635100	3.09197400	1.46487300
C	-2.46775900	4.55636600	-0.92215700
C	-1.83472700	4.43638000	1.40279700
C	-1.96466800	5.16036700	0.22428400
H	-2.55794900	5.13426200	-1.83700100
H	-1.43924400	4.92155700	2.29067500
H	-1.67138000	6.20589400	0.19822600
C	-2.35453800	-2.32090900	0.09339600
C	-2.23782400	-2.86611300	-1.19199600
C	-1.91911600	-2.97412600	1.25481000
C	-1.65698000	-4.13124400	-1.29437500
C	-1.34195700	-4.23471400	1.09482900
C	-1.21461400	-4.80869400	-0.16504800
H	-1.54782900	-4.58981300	-2.27291100
H	-0.98703900	-4.77392600	1.96797900
H	-0.76728400	-5.79338800	-0.26680200
C	-2.04210700	2.30973000	2.75248800
H	-2.42350500	1.29490200	2.59123500
C	-0.56382400	2.18509400	3.13067800
H	-0.11813000	3.16781500	3.32132100
H	0.00769500	1.70388600	2.32785700
H	-0.45067700	1.58248300	4.03847900
C	-2.85617500	2.92929000	3.89042800
H	-2.76656100	2.32096200	4.79679500
H	-3.91711300	2.99949300	3.62907300
H	-2.50448500	3.93821300	4.13205200
C	-2.01527600	-2.33642700	2.62649300
H	-2.67673000	-1.46502100	2.55440600
C	-2.62270500	-3.28076000	3.66444200
H	-2.75843400	-2.75574400	4.61580300
H	-1.97540100	-4.14284800	3.85776000
H	-3.59765100	-3.65890900	3.34027800
C	-0.64035100	-1.83129900	3.07310600
H	-0.23971200	-1.10115000	2.36120500
H	0.07697100	-2.65687900	3.14335100
H	-0.70658600	-1.35198500	4.05634300
C	-2.69513000	-2.13116600	-2.43539000
H	-3.15313400	-1.18397000	-2.12776600
C	-3.35274300	2.54420600	-2.18039200
H	-3.80094900	1.58253700	-1.90427900
C	-2.17743100	2.24926000	-3.11785600
H	-1.42565500	1.62120000	-2.62523200
H	-1.68698400	3.17764100	-3.43214100
H	-2.52397800	1.72765000	-4.01703200
C	-4.43378600	3.36069000	-2.88915800
H	-4.82587200	2.80124800	-3.74493800
H	-4.04179900	4.30886700	-3.27243600
H	-5.26832700	3.58869800	-2.21823300
C	-1.50297900	-1.79140000	-3.33331800
H	-1.00376700	-2.69863300	-3.69122000
H	-0.76149200	-1.19326300	-2.79056400
H	-1.83294700	-1.22006200	-4.20783300
C	-3.75679800	-2.92706800	-3.19742800
H	-3.35633700	-3.87666700	-3.56868500
H	-4.11107500	-2.35630500	-4.06244900
H	-4.61845000	-3.15410600	-2.56109400
C	2.73005800	1.33537700	-0.49747000
H	2.41004600	2.17421200	-1.13056200
C	4.12613400	0.75648500	-0.78446200

H	4.22768300	0.72440300	-1.87734300
C	3.99188800	-0.70824100	-0.30904800
H	2.58870400	-0.95883200	-1.90611800
C	2.56208800	-0.98780900	-0.79641600
Br	1.75669000	-2.69280800	-0.37533300
H	-5.17982100	1.28199700	0.74917600
H	-4.97616400	-1.50073300	0.62533400
H	4.69579100	-1.34916100	-0.84906400
H	2.64958400	1.73667400	0.52895900
C	5.32629400	1.52365100	-0.21052600
H	5.13860300	2.59940700	-0.35405600
C	4.20032100	-0.94104100	1.19979500
C	5.43992300	1.26372500	1.29513600
C	5.49054600	-0.22503900	1.62460200
H	6.34930200	-0.68661900	1.11968600
H	5.64746200	-0.37100700	2.69961600
H	4.34380100	-2.02205600	1.32094100
H	6.32014700	1.77330400	1.69669300
H	4.58596100	1.72674800	1.80809100
C	6.61012500	1.18914900	-1.01119600
H	6.65709700	0.09792200	-1.14761100
C	6.57231300	1.83693300	-2.39645600
H	6.59011600	2.93034000	-2.30709800
H	5.68396000	1.56617800	-2.97773400
H	7.44551200	1.53942800	-2.98603000
C	7.88843500	1.62265300	-0.29645300
H	8.75611700	1.46560500	-0.94559400
H	8.06701200	1.06203400	0.62617200
H	7.85555200	2.69023400	-0.04436300
C	3.03712600	-0.55702300	2.11788300
H	2.08009100	-0.96770100	1.77464800
H	2.91843000	0.52295300	2.24686400
H	3.21273800	-0.97406500	3.11521200

Cartesian coordinates of the optimized geometry for Int-**4c** at PBE0-D3BJ/6-31G*,def2-TZVP level of theory: (number of imaginary frequencies = 0):

Au	0.22362600	-0.41374700	-0.12142200
C	-1.64458400	-1.04284100	-0.06236600
C	2.09430000	0.38574200	-0.01536600
C	3.73671800	1.91437100	0.12897400
C	4.31089300	0.68370300	0.20819800
N	2.38191400	1.70642700	-0.00709000
N	3.28638000	-0.23330800	0.11914300
C	3.43392400	-1.66003200	0.17179500
C	3.61707800	-2.35147600	-1.03307200
C	3.35332200	-2.28705500	1.42239900
C	3.72324100	-3.74108500	-0.95601900
C	3.46661100	-3.67815400	1.44202300
C	3.64988400	-4.39694200	0.26696400
H	3.86337800	-4.31682300	-1.86617900
H	3.40965500	-4.20472300	2.39040000
H	3.73719100	-5.47895900	0.30461800
C	1.38669500	2.73685900	-0.11669000
C	1.04313600	3.19126100	-1.39712500
C	0.79186700	3.21343300	1.05922800
C	0.06506600	4.18352800	-1.47763900
C	-0.17785400	4.20806700	0.92194800
C	-0.53467300	4.69168300	-0.33126200
H	-0.23258300	4.56104000	-2.45144300
H	-0.66339700	4.60481000	1.80884500
H	-1.28925300	5.46858100	-0.41572300
C	3.12421700	-1.51497700	2.70631100
H	3.15923400	-0.44405700	2.47514600
C	1.73437700	-1.81291600	3.27570200

H	1.63315600	-2.87253900	3.53629200
H	0.95155800	-1.56649400	2.54839200
H	1.55724100	-1.22398200	4.18247600
C	4.21850400	-1.79441300	3.73818200
H	4.06427500	-1.17821300	4.63034700
H	5.21217400	-1.57156500	3.33610600
H	4.21420900	-2.84248900	4.05646800
C	1.13646400	2.66539900	2.42916100
H	1.96120800	1.95185500	2.31769200
C	1.61081300	3.76795400	3.37757200
H	1.90696700	3.33853300	4.34056900
H	0.81898900	4.49948700	3.57184700
H	2.46973000	4.30722700	2.96490100
C	-0.05324900	1.89967800	3.01570100
H	-0.36036300	1.08395800	2.35100200
H	-0.91886200	2.55683800	3.15565600
H	0.20975400	1.47215600	3.98964800
C	1.65746000	2.61344900	-2.65560400
H	2.47469200	1.94371200	-2.36344700
C	3.65819100	-1.64576200	-2.37344100
H	3.70608700	-0.56574400	-2.19155200
C	2.37389200	-1.92076900	-3.16096600
H	1.48861600	-1.60538000	-2.59602000
H	2.26939600	-2.98925200	-3.38095700
H	2.38361600	-1.37878300	-4.11308600
C	4.89822900	-2.02561700	-3.18419100
H	4.93383500	-1.44566900	-4.11244000
H	4.89238300	-3.08556300	-3.46016700
H	5.81806800	-1.83047500	-2.62338500
C	0.62440100	1.77327400	-3.41198600
H	-0.21999700	2.38962800	-3.74028900
H	0.22610400	0.97326800	-2.77706300
H	1.07652600	1.31673400	-4.29940200
C	2.25587700	3.69886600	-3.55168300
H	1.48605000	4.37968400	-3.93066700
H	2.74754100	3.24443000	-4.41832500
H	2.99719300	4.29829700	-3.01320600
C	-2.17652400	-2.36214100	0.34019000
H	-1.68972300	-3.18635200	-0.19892400
H	-3.04303900	-0.43404900	-1.34892000
C	-2.82238100	-0.19526000	-0.28924800
Br	-2.49050900	1.71161200	-0.32005100
H	5.34001100	0.37881700	0.31719600
H	4.16115800	2.90587100	0.15547300
H	-1.82488800	-2.47881900	1.38312500
C	-3.98496000	-0.78439600	0.55838900
C	-5.36284600	-0.32945000	0.08017000
C	-3.70703400	-2.28792400	0.25592000
C	-5.75058300	-0.83450500	-1.30395000
H	-6.10496200	-0.69026600	0.80508300
H	-5.41213200	0.76451400	0.10923800
C	-4.20115800	-2.76487900	-1.11559500
H	-4.18199700	-2.91463700	1.01358100
C	-5.63947600	-2.35240000	-1.41188100
H	-6.77417000	-0.51181800	-1.52819800
H	-5.11823100	-0.36994400	-2.07816000
H	-4.09972100	-3.85703000	-1.15442200
H	-3.55777600	-2.38275600	-1.92473800
H	-6.28684700	-2.79386100	-0.63757400
C	-3.75776100	-0.45196500	2.07535000
H	-2.67017400	-0.38094000	2.23592100
C	-4.28794200	-1.53406600	3.01756800
H	-5.33969300	-1.76748900	2.81248400
H	-4.23195800	-1.17955600	4.05168800
H	-3.71867700	-2.46841400	2.96777200
C	-4.35042300	0.89272500	2.50933900

H	-5.44455600	0.85366300	2.53651000
H	-4.05231600	1.71812300	1.86099900
H	-4.00881500	1.12456900	3.52360000
C	-6.09292800	-2.86312900	-2.77259200
H	-7.13313300	-2.58489100	-2.97215700
H	-6.01904400	-3.95438700	-2.83499500
H	-5.47508800	-2.43918200	-3.57432800

Cartesian coordinates of the optimized geometry for Int-**4d** at PBE0-D3BJ/6-31G*,def2-TZVP level of theory: (number of imaginary frequencies = 0):

Au	-0.15542600	0.06370800	-0.39027100
C	1.73039600	0.03698400	-0.96064900
C	-2.03847100	0.02984300	0.38175600
C	-3.87503300	-0.75437400	1.41379200
C	-4.05914000	0.57617900	1.19694300
N	-2.63113300	-1.06372300	0.90939300
N	-2.92293100	1.03238100	0.56334100
C	-2.67513400	2.38583900	0.15403600
C	-1.99011700	3.23096900	1.03727800
C	-3.09978100	2.77816900	-1.12271400
C	-1.73555100	4.53221300	0.60044100
C	-2.81798600	4.08923000	-1.50930900
C	-2.14555600	4.95715300	-0.65714100
H	-1.20873000	5.22083100	1.25495600
H	-3.12633700	4.43398200	-2.49179300
H	-1.93885500	5.97439700	-0.97698100
C	-2.00578300	-2.35710900	0.91698400
C	-1.17322500	-2.68602000	1.99594100
C	-2.21561400	-3.19976500	-0.18263800
C	-0.54161700	-3.92970500	1.95436200
C	-1.56016100	-4.43223500	-0.17255400
C	-0.73401200	-4.79446900	0.88396200
H	0.11167400	-4.22346800	2.77103400
H	-1.69613700	-5.11450100	-1.00672800
H	-0.23336600	-5.75830500	0.87221700
C	-3.78507000	1.81942800	-2.07547100
H	-4.07203900	0.92310900	-1.51320000
C	-2.81110600	1.37971700	-3.17302700
H	-2.49047200	2.23625900	-3.77687400
H	-1.91506100	0.91547300	-2.74456000
H	-3.28688100	0.65344400	-3.84128700
C	-5.06327600	2.40917200	-2.67258500
H	-5.56982400	1.66023400	-3.29035300
H	-5.75760200	2.73426400	-1.89100500
H	-4.85162400	3.27158400	-3.31372500
C	-3.07400300	-2.79673000	-1.36417600
H	-3.55692000	-1.84103600	-1.12959500
C	-4.18347300	-3.81459500	-1.63434200
H	-4.82197600	-3.46937000	-2.45438200
H	-3.77476300	-4.78895600	-1.92317100
H	-4.81246600	-3.96452000	-0.75072400
C	-2.20439500	-2.57725300	-2.60528900
H	-1.43039500	-1.82395900	-2.41848600
H	-1.70205400	-3.50418800	-2.90397100
H	-2.81758400	-2.23960000	-3.44800600
C	-0.92902400	-1.74207000	3.15612500
H	-1.55668400	-0.85430400	3.01679600
C	-1.51697900	2.77483600	2.40275400
H	-1.85360400	1.74327500	2.55772400
C	0.01247800	2.77030900	2.47508500
H	0.44016600	2.12994300	1.69436300
H	0.41902100	3.77947100	2.34316900
H	0.34885500	2.39698800	3.44873100
C	-2.12349600	3.62683600	3.51960300

H	-1.81128500	3.24853100	4.49888900
H	-1.79917800	4.67073200	3.44867300
H	-3.21762600	3.61412700	3.48095800
C	0.52793700	-1.27079000	3.17538900
H	1.21544400	-2.11370100	3.30982200
H	0.78561800	-0.76643600	2.23616900
H	0.69330300	-0.56610800	3.99780500
C	-1.32416100	-2.37736900	4.49073300
H	-0.70625800	-3.25303700	4.71719700
H	-1.19331200	-1.65937000	5.30735200
H	-2.37024600	-2.70032600	4.48354300
C	2.48387500	0.97853300	-1.80097200
H	2.03304300	1.97123500	-1.87173500
H	2.69596900	-1.10509400	0.57289200
C	2.70771900	-1.00527700	-0.51752500
Br	1.92926900	-2.67949800	-1.17487500
H	-4.88302600	1.23240600	1.43046800
H	-4.50654300	-1.49906900	1.87266700
H	2.39409000	0.53269000	-2.81483900
C	4.08106200	-0.64991300	-1.10366400
C	5.29030300	-1.08337600	-0.27076800
C	3.94307200	0.87511400	-1.32219100
H	4.14647500	-1.12543000	-2.09130400
C	5.41966800	-0.23836200	0.99412900
H	6.17291800	-0.86738700	-0.89093700
C	4.15170400	1.68663500	-0.02873900
H	4.64427700	1.21263500	-2.09295300
C	5.44013100	1.24896700	0.66732300
H	6.33444900	-0.52561200	1.52575700
H	4.58907800	-0.45655900	1.68429100
H	3.31830200	1.42739000	0.65192100
H	5.58800200	1.83368800	1.58174300
H	6.30065700	1.46043700	0.01783600
C	4.05922900	3.21044400	-0.25710600
H	3.17055000	3.39604400	-0.87788100
C	3.83486400	3.95586800	1.05738600
H	3.68294300	5.02467600	0.87289300
H	2.95200300	3.57698100	1.58628100
H	4.69576100	3.86264600	1.72883200
C	5.26245000	3.78562900	-1.00276600
H	6.17107800	3.73689400	-0.39281500
H	5.46250700	3.26422900	-1.94569400
H	5.08881800	4.83942600	-1.24414200
C	5.28411700	-2.57739200	0.03123300
H	4.44925000	-2.85369600	0.68717400
H	5.19901000	-3.17576500	-0.88216800
H	6.20921800	-2.86686900	0.54028800

Cartesian coordinates of the optimized geometry for Int-**4e** at PBE0-D3BJ/6-31G*,def2-TZVP level of theory: (number of imaginary frequencies = 0):

Au	-0.44161800	-0.45312100	-0.36113300
C	2.50179100	-1.22670400	-1.81237000
C	-1.25505800	1.28088400	0.32145200
C	-2.76500300	2.73562100	1.13475000
C	-1.60848300	3.41811100	0.92335500
N	-2.52425200	1.43289200	0.75763100
N	-0.69734300	2.50686200	0.43104600
C	0.64865500	2.81838500	0.04084000
C	0.90060200	3.07498900	-1.31328000
C	1.63748700	2.85791300	1.03634000
C	2.21864500	3.37288200	-1.66793200
C	2.93493900	3.16702500	0.62758700
C	3.22185100	3.42285700	-0.70937200
H	2.45648600	3.58290500	-2.70680700

H	3.73154400	3.21744200	1.36274900
H	4.23789000	3.67110100	-1.00326100
C	-3.47357300	0.35651600	0.80108800
C	-4.29112200	0.15103300	-0.31866400
C	-3.50329300	-0.45458800	1.94326600
C	-5.17906600	-0.92435200	-0.26547900
C	-4.41196500	-1.51417900	1.94428000
C	-5.24054500	-1.74632100	0.85339900
H	-5.82860700	-1.12219500	-1.11313100
H	-4.46879600	-2.16703500	2.81061800
H	-5.94055000	-2.57654000	0.87468400
C	1.32362200	2.54083600	2.48606200
H	0.25827800	2.74029800	2.65368600
C	1.55719900	1.05284000	2.76600900
H	2.60826800	0.78826500	2.59907300
H	0.94208900	0.42625200	2.11117000
H	1.30606400	0.81057100	3.80471500
C	2.10690900	3.40804100	3.47032900
H	1.74687800	3.23178500	4.48905200
H	1.99409400	4.47392400	3.24780300
H	3.17632700	3.17082100	3.46179600
C	-2.58418300	-0.22994800	3.12677200
H	-1.98257000	0.66536900	2.93121600
C	-3.37557400	0.02137200	4.41186300
H	-2.69401700	0.22928800	5.24364200
H	-3.97876000	-0.85037900	4.68777000
H	-4.05290600	0.87475900	4.30300100
C	-1.61517200	-1.40372000	3.28979900
H	-1.03607400	-1.56624200	2.37347200
H	-2.15060300	-2.33241400	3.51645700
H	-0.91424800	-1.21142400	4.10958400
C	-4.20141900	1.01675300	-1.55929400
H	-3.54707700	1.86817900	-1.33834400
C	-0.18963500	3.06500600	-2.36624300
H	-1.14364600	2.84408500	-1.87438200
C	0.05002200	1.96778800	-3.40502800
H	0.07867400	0.97903800	-2.93263600
H	0.99690900	2.11790600	-3.93563900
H	-0.75394600	1.96436900	-4.14887800
C	-0.32519500	4.43792300	-3.02959900
H	-1.15519800	4.43341500	-3.74409600
H	0.58333200	4.70976500	-3.57787500
H	-0.51545400	5.22170700	-2.28906000
C	-3.56281600	0.23592700	-2.71164100
H	-4.17399600	-0.63123100	-2.98584900
H	-2.56605000	-0.12955400	-2.43770200
H	-3.46332400	0.87255300	-3.59786200
C	-5.56402300	1.58390600	-1.96074600
H	-6.26083200	0.79165500	-2.25473600
H	-5.45480800	2.25873200	-2.81629700
H	-6.02183300	2.14434100	-1.13910600
C	3.35190700	-0.81630100	-0.62287600
C	3.74849300	-2.16045000	-0.02549300
H	4.40719700	-2.65080600	-0.76184300
C	2.45185700	-2.99554700	0.03402800
H	1.77889200	-3.29772200	-2.04775400
C	1.70708700	-2.46603700	-1.34033100
H	-1.35076500	4.45512200	1.07267900
H	-3.72520000	3.05163800	1.51163900
H	2.71256400	-0.28437500	0.10070000
C	1.67710800	-2.74572700	1.32977000
H	0.70919400	-3.25747800	1.33494200
H	1.50545100	-1.68430800	1.52700500
H	2.25477500	-3.15078600	2.16663200
C	2.69738200	-4.49038600	-0.10913800
H	3.26389300	-4.85499300	0.75601100

H	1.75623600	-5.05119100	-0.14485900
H	3.27219800	-4.72021400	-1.01257600
C	0.32324400	-2.16684500	-1.02215200
Br	-0.84552900	-3.59798300	-1.22469300
H	1.85117700	-0.42569500	-2.17866600
H	3.14175300	-1.53271100	-2.64909600
C	4.56705400	0.04692700	-0.90951900
H	5.19196300	-0.44450700	-1.67151500
H	4.26181700	1.01581200	-1.32152000
C	5.39187600	0.24884000	0.36537400
C	4.54567700	-1.98415100	1.26134900
H	3.92054500	-1.50220400	2.02480700
H	4.86121500	-2.95259900	1.66943900
C	5.76602700	-1.10203000	0.98381500
H	6.32429900	-0.94147100	1.91426100
H	6.44830100	-1.62799800	0.29954400
H	4.75071300	0.78040600	1.08785600
C	6.62437000	1.10338800	0.10142400
H	7.19506000	1.27810700	1.02010800
H	7.29251900	0.61362600	-0.61747300
H	6.34910900	2.07965300	-0.31488600

Cartesian coordinates of the optimized geometry for Int-**4f** at PBE0-D3BJ/6-31G*,def2-TZVP level of theory: (number of imaginary frequencies = 0):

Au	-0.20355500	0.20798600	-0.08856800
C	1.76749500	0.20269900	-0.17127000
C	-2.23386300	0.08999000	0.04926300
C	-4.29315200	-0.78835600	0.25592600
C	-4.42603700	0.56409500	0.19401000
N	-2.94476400	-1.05373800	0.16425100
N	-3.15361700	1.07792200	0.06920000
C	-2.81965200	2.47032400	-0.03019100
C	-2.75884300	3.04634200	-1.30622800
C	-2.54025000	3.16964600	1.15146000
C	-2.39687900	4.39248300	-1.37692100
C	-2.18389000	4.51315700	1.02317900
C	-2.11323300	5.11783700	-0.22594500
H	-2.33497400	4.87817200	-2.34637200
H	-1.95824700	5.09248700	1.91387500
H	-1.83555500	6.16508700	-0.30330900
C	-2.34292900	-2.35770000	0.20344000
C	-2.12994700	-3.02726900	-1.00911800
C	-1.96955200	-2.87639300	1.45042300
C	-1.52388200	-4.28248800	-0.94307900
C	-1.36520900	-4.13482400	1.45919500
C	-1.14848200	-4.83252100	0.27675000
H	-1.33866800	-4.83404400	-1.86013600
H	-1.05989800	-4.57278900	2.40511400
H	-0.68099700	-5.81265100	0.30594200
C	-2.58543400	2.51151300	2.51575400
H	-2.98010600	1.49601000	2.39509200
C	-1.17593900	2.38676400	3.10064200
H	-0.72411500	3.37317600	3.25492900
H	-0.52183200	1.81624100	2.43049500
H	-1.20601700	1.87413400	4.06843600
C	-3.52027000	3.25324600	3.47286800
H	-3.58282600	2.72186800	4.42844600
H	-4.53105300	3.33521000	3.06019100
H	-3.16149100	4.26674400	3.68238400
C	-2.16684100	-2.11132000	2.74321900
H	-2.72676000	-1.19575700	2.51991000
C	-2.98721600	-2.91163700	3.75641700
H	-3.16498700	-2.31370000	4.65657900
H	-2.46644700	-3.82463000	4.06474000

H	-3.95788700	-3.20412400	3.34274400
C	-0.81623300	-1.68973900	3.32830400
H	-0.24714400	-1.08732700	2.61080700
H	-0.20680500	-2.56299800	3.58624000
H	-0.96146600	-1.09774500	4.23873200
C	-2.49459900	-2.42023600	-2.34844500
H	-3.03588100	-1.48443900	-2.16680900
C	-3.03083600	2.25196800	-2.56785800
H	-3.43033400	1.27306400	-2.27815000
C	-1.72924900	2.00555000	-3.33611800
H	-0.99669500	1.47938500	-2.71255300
H	-1.27897700	2.95078100	-3.65978700
H	-1.91898000	1.39847100	-4.22826300
C	-4.07920900	2.92639800	-3.45417200
H	-4.30496300	2.29402700	-4.31928500
H	-3.72653400	3.89054600	-3.83590200
H	-5.01102400	3.10338300	-2.90729500
C	-1.22989400	-2.07114000	-3.13798500
H	-0.64135600	-2.96899600	-3.35730600
H	-0.59194900	-1.38242300	-2.57167800
H	-1.49064600	-1.59537800	-4.08980500
C	-3.41948000	-3.33383000	-3.15453900
H	-2.92642200	-4.27595100	-3.41738300
H	-3.71436300	-2.84463700	-4.08895100
H	-4.32813000	-3.57705600	-2.59419400
C	2.71419400	1.27485000	0.17713000
H	2.37678000	2.27532400	-0.11353000
C	4.10102100	0.80403900	-0.26528200
H	4.17831300	0.97493800	-1.35046800
C	4.01732200	-0.71194900	-0.04137600
H	2.56601500	-1.01513400	-1.62937700
C	2.58897500	-0.98800000	-0.52411600
Br	1.73470800	-2.63447400	0.05837000
H	-5.29646600	1.20071900	0.22556900
H	-5.02354800	-1.57605500	0.35585800
H	2.65646900	1.26776400	1.28973000
C	5.33309900	1.40458600	0.40242100
C	6.55578600	0.69989800	-0.19963700
C	5.19620000	-1.42907300	-0.69040800
C	6.47237100	-0.82292800	-0.08735000
H	5.17795900	-1.19252800	-1.76677000
H	7.34585900	-1.27634300	-0.57050300
H	6.52286500	-1.11363900	0.97266700
H	7.47023600	1.04546600	0.29386700
H	6.64826900	0.97866600	-1.25847200
H	4.03984600	-0.90709100	1.04288100
H	5.28604100	1.13784600	1.47188300
C	5.37481200	2.94349800	0.33248500
H	4.46145700	3.29964700	0.83461100
C	6.55925900	3.50573300	1.11585100
H	7.51104700	3.28238900	0.62128700
H	6.48413800	4.59503500	1.19859200
H	6.60215400	3.09434400	2.13110100
C	5.36140800	3.49850200	-1.09087700
H	6.25329400	3.19450800	-1.65006900
H	4.48208100	3.17603700	-1.66096000
H	5.35076500	4.59330900	-1.06952700
C	5.18069400	-2.94322600	-0.52240000
H	5.13385300	-3.22046300	0.53746500
H	4.32687000	-3.40693300	-1.02471200
H	6.09416200	-3.37849300	-0.94136300

Cartesian coordinates of the optimized geometry for TS-**1a** at PBE0-D3BJ/6-31G*,def2-TZVP level of theory: (number of imaginary frequencies = 1):

Au	-0.01160700	-0.27978000	0.10134500
C	1.88628400	-1.01862200	0.03919600
C	-1.81896300	0.63274400	0.18193900
C	-3.35687500	2.28123200	0.16527900
C	-4.00719800	1.11433300	0.40646800
N	-2.01991800	1.96156800	0.03187000
N	-3.04652200	0.12133200	0.41184500
C	-3.29708000	-1.27332000	0.63468300
C	-3.57063500	-2.08297900	-0.47553900
C	-3.22956700	-1.75758100	1.94780800
C	-3.78673000	-3.44046300	-0.23376400
C	-3.45377200	-3.12248200	2.13379400
C	-3.73031200	-3.95453600	1.05586000
H	-3.99804700	-4.10387600	-1.06740100
H	-3.40790200	-3.53924300	3.13582600
H	-3.90187500	-5.01411900	1.22225700
C	-0.95550600	2.88337000	-0.23846200
C	-0.63238600	3.14881500	-1.57623200
C	-0.26179200	3.43599000	0.84724500
C	0.43419900	4.01812100	-1.81340600
C	0.80093100	4.29321300	0.55484000
C	1.14396300	4.58383400	-0.76050000
H	0.71396800	4.25163800	-2.83671900
H	1.36192800	4.74381700	1.36881200
H	1.96874300	5.26018400	-0.96697600
C	-2.89239500	-0.86440000	3.12455300
H	-2.87067500	0.17330800	2.77202800
C	-1.49743200	-1.19319300	3.66374500
H	-1.45498000	-2.21972700	4.04515600
H	-0.74034100	-1.09446400	2.87692400
H	-1.23434600	-0.51642900	4.48469400
C	-3.94943400	-0.94931900	4.22674500
H	-3.71234100	-0.24768800	5.03373900
H	-4.94599700	-0.70616000	3.84383600
H	-3.99528800	-1.95193000	4.66556300
C	-0.61653800	3.11721800	2.28568500
H	-1.51402900	2.48862700	2.28555100
C	-0.94340900	4.38459100	3.07784500
H	-1.25642900	4.12573100	4.09495400
H	-0.07334300	5.04556700	3.15770900
H	-1.75172700	4.95235100	2.60560700
C	0.50039000	2.31327900	2.95646700
H	0.68525700	1.37939600	2.41284200
H	1.43612800	2.88382300	2.98725900
H	0.22527700	2.06117700	3.98656000
C	-1.36809800	2.49978400	-2.73092700
H	-2.23308500	1.96235400	-2.32622100
C	-3.59386100	-1.53788800	-1.88904800
H	-3.52782700	-0.44501600	-1.83613600
C	-2.37292600	-2.03172300	-2.66982600
H	-1.44215300	-1.74911900	-2.16581000
H	-2.38391900	-3.12342000	-2.76691700
H	-2.36328400	-1.60227000	-3.67812800
C	-4.89609000	-1.88148100	-2.61364600
H	-4.90926000	-1.41954100	-3.60666100
H	-5.00794100	-2.96221300	-2.75288900
H	-5.76929600	-1.52362300	-2.05827500
C	-0.47367400	1.46808400	-3.42428400
H	0.41442800	1.94345000	-3.85671200
H	-0.13631500	0.70039300	-2.71876400
H	-1.01958600	0.97268000	-4.23486200
C	-1.89738800	3.53566300	-3.72401200
H	-1.08257700	4.07883400	-4.21514800
H	-2.48171700	3.04220700	-4.50782400

H	-2.54055900	4.27147300	-3.23022400
C	2.91705300	-0.48536400	1.00304600
C	4.12895800	-1.44677000	1.04884000
H	3.80350500	-2.42775700	1.42070300
C	4.58381800	-1.58196600	-0.35191300
H	3.38790700	-2.30924000	-0.79359700
C	2.32245200	-1.84575200	-0.90017100
Br	1.65276700	-2.38337600	-2.54312000
H	-5.05041700	0.89586400	0.57280100
H	-3.71283000	3.29560400	0.07553000
H	5.13016500	-2.48392500	-0.64272200
C	4.92053100	-0.35509900	-1.11792100
C	3.37434000	0.90512900	0.53030000
C	4.08312600	0.91954600	-0.84193800
H	4.79628000	1.75156700	-0.81062900
H	5.97550300	-0.17909700	-0.83602400
H	4.95905900	-0.58069700	-2.19024800
H	2.50822500	1.57451900	0.48936700
H	2.48260100	-0.39391000	2.00261500
C	5.29100800	-0.97761600	1.97579200
H	5.69353000	-0.03605900	1.58185800
C	4.77729200	-0.73028700	3.39054100
H	4.06348800	0.09762800	3.43962000
H	4.29044900	-1.62603200	3.79428600
H	5.61486800	-0.48149800	4.05030200
C	6.41552800	-2.01179100	1.98539600
H	7.20586200	-1.70571100	2.67778600
H	6.04795600	-2.99084600	2.31546300
H	6.87772600	-2.13876300	0.99962300
H	4.03891400	1.31636700	1.29894200
C	3.13607000	1.22422300	-2.00547300
H	2.40760000	0.42989800	-2.18120800
H	2.57634800	2.14310200	-1.80424700
H	3.70426900	1.37209600	-2.93094700

Cartesian coordinates of the optimized geometry for TS-**1b** at PBE0-D3BJ/6-31G*,def2-TZVP level of theory: (number of imaginary frequencies = 1):

Au	0.03445400	-0.10332600	-0.09966700
C	1.85512600	-1.05762900	-0.22512700
C	-1.72725500	0.85420700	0.05739700
C	-3.92724800	1.24969400	0.29840500
C	-3.30146400	2.45197400	0.20420500
N	-2.94213200	0.28604500	0.20579800
N	-1.95405200	2.18377600	0.05767100
C	-0.90219000	3.14965100	-0.08264400
C	-0.25805000	3.60357700	1.07637300
C	-0.53536700	3.54583500	-1.37628100
C	0.79371700	4.50591800	0.90730800
C	0.52311500	4.44887100	-1.48901600
C	1.17850500	4.92648600	-0.36035900
H	1.31954100	4.88191400	1.78027100
H	0.83795000	4.78107600	-2.47408800
H	1.99589100	5.63389300	-0.46942400
C	-3.12636400	-1.13645300	0.25894100
C	-3.03475900	-1.77077300	1.50540700
C	-3.32310200	-1.82519500	-0.94565800
C	-3.16351600	-3.16073400	1.52290800
C	-3.44506400	-3.21390400	-0.87191500
C	-3.36888400	-3.87478100	0.34836500
H	-3.10063600	-3.68947300	2.46976600
H	-3.59996600	-3.78413200	-1.78339700
H	-3.47343500	-4.95565900	0.38464100
C	-1.21673000	3.00253600	-2.61551100
H	-2.06575900	2.38636000	-2.29821300

C	-0.26186200	2.09712700	-3.39851000
H	0.60867600	2.65821100	-3.75700900
H	0.10041400	1.27313500	-2.77259400
H	-0.76841700	1.66850200	-4.27030900
C	-1.76985800	4.12372200	-3.49698400
H	-2.30873600	3.70171700	-4.35194100
H	-2.46050500	4.76565100	-2.94041100
H	-0.96908600	4.75781200	-3.89282400
C	-3.36256300	-1.12187100	-2.28678400
H	-3.32547400	-0.04102900	-2.10889300
C	-4.65857200	-1.41685600	-3.04402600
H	-4.68625000	-0.85153300	-3.98162300
H	-4.74432800	-2.47923600	-3.29713000
H	-5.53825500	-1.14204300	-2.45281900
C	-2.13246700	-1.49065600	-3.12072600
H	-1.20706500	-1.24396000	-2.58733300
H	-2.11583200	-2.56278500	-3.34713500
H	-2.13694300	-0.94606400	-4.07140100
C	-2.77156400	-1.00941500	2.78845300
H	-2.76224200	0.06173000	2.55660300
C	-0.64215800	3.12404600	2.46126600
H	-1.53746500	2.49810300	2.37187200
C	0.46771000	2.25183000	3.05448700
H	0.67341200	1.38782200	2.41226600
H	1.39893000	2.81940500	3.16564000
H	0.17725300	1.88264200	4.04431400
C	-0.98986600	4.29026600	3.38818900
H	-1.32397600	3.91391500	4.36089800
H	-0.12342800	4.93680300	3.56504600
H	-1.78904100	4.90978000	2.96824900
C	-1.39413500	-1.36658900	3.35361200
H	-1.33959300	-2.42747900	3.62282800
H	-0.60356700	-1.16291500	2.62199400
H	-1.18698800	-0.77992500	4.25543700
C	-3.87589700	-1.24498500	3.82066300
H	-3.91827400	-2.29456700	4.13163100
H	-3.69234000	-0.64232500	4.71655500
H	-4.85930900	-0.97474200	3.42217500
C	3.17146400	-0.35716800	-0.39382400
C	4.30877800	-1.25415100	0.14508600
H	4.20349200	-1.26499600	1.24033500
C	4.20505700	-2.68624500	-0.32270500
H	3.02253800	-2.89452600	-0.14346500
C	1.73012600	-2.33970100	-0.12217800
Br	0.62074900	-3.77018400	-0.04529900
H	-3.68207600	3.46109800	0.22701700
H	-4.96749100	0.99163400	0.42106900
H	3.30923700	-0.21886500	-1.47840100
C	5.65493400	-0.59366700	-0.18831300
H	5.76803600	-0.51716300	-1.27780500
H	6.47690600	-1.22174500	0.17640900
C	5.73372500	0.79548700	0.44019600
C	3.23557900	1.02397900	0.25688700
C	4.58227500	1.70126300	0.00120600
H	5.71473200	0.69778900	1.53572100
H	6.69329300	1.26163000	0.18614600
H	3.07069600	0.92096500	1.33945900
H	2.42194600	1.65254200	-0.12574100
H	4.67269000	1.85509100	-1.08613000
C	4.85502400	-3.70749800	0.58305900
H	5.94615200	-3.59730500	0.52099900
H	4.56238400	-3.56742500	1.62778200
H	4.61185800	-4.72955800	0.27779200
C	4.42650100	-2.93724200	-1.79716700
H	4.11168200	-3.94750200	-2.07533600
H	3.89009000	-2.22089100	-2.42595200

H	5.49675800	-2.85503600	-2.02361800
C	4.64318400	3.06125200	0.68413200
H	3.82392900	3.70615400	0.34782200
H	4.55909100	2.95329400	1.77287800
H	5.58877600	3.57140600	0.47133300

Cartesian coordinates of the optimized geometry for TS-**2a** at PBE0-D3BJ/6-31G*,def2-TZVP level of theory: (number of imaginary frequencies = 1):

Au	-0.23716000	-0.23812900	-0.09271800
C	1.80627900	-0.52960500	-0.23446400
C	-2.20683200	0.14828600	0.06803500
C	-4.11691400	1.30754600	0.33670300
C	-4.44757400	0.00009100	0.17895000
N	-2.73934900	1.37436100	0.26567300
N	-3.26177200	-0.68974800	0.01578400
C	-3.13759600	-2.10491000	-0.18329500
C	-3.15000900	-2.59207400	-1.49723200
C	-2.97735900	-2.92081900	0.94456900
C	-2.99458800	-3.96896800	-1.66461300
C	-2.82642000	-4.29025800	0.72043900
C	-2.83575900	-4.80868600	-0.56874100
H	-2.99576000	-4.38820100	-2.66657200
H	-2.69811900	-4.95842300	1.56717300
H	-2.71825200	-5.87774700	-0.72107800
C	-1.94914800	2.56667500	0.38288000
C	-1.63142300	3.27110200	-0.78666700
C	-1.50815200	2.94847300	1.65764100
C	-0.85325300	4.42180600	-0.64553600
C	-0.73212500	4.10602300	1.74290800
C	-0.41401700	4.83961700	0.60553100
H	-0.59069500	4.99830500	-1.52799000
H	-0.37484500	4.43731600	2.71377200
H	0.17970000	5.74533000	0.69518100
C	-2.93165100	-2.36037600	2.35166300
H	-3.17878700	-1.29347700	2.30432600
C	-1.51894300	-2.47717900	2.93077000
H	-1.21193100	-3.52614100	3.01132200
H	-0.78978800	-1.95913700	2.29662400
H	-1.47746900	-2.03557000	3.93272100
C	-3.96325600	-3.02711500	3.26331900
H	-3.95202200	-2.55871800	4.25326900
H	-4.97487300	-2.93785300	2.85407900
H	-3.75101500	-4.09285400	3.40126200
C	-1.81367600	2.13675400	2.89966600
H	-2.49446200	1.32479700	2.62003800
C	-2.51614600	2.97602900	3.96827400
H	-2.77709600	2.35114300	4.82895500
H	-1.87405000	3.78579700	4.33167400
H	-3.43608100	3.42668500	3.58158900
C	-0.53558000	1.49355300	3.44523900
H	-0.05929900	0.86025900	2.68767700
H	0.18977100	2.25493800	3.75379100
H	-0.76294100	0.87176800	4.31815500
C	-2.07215100	2.80819100	-2.16006100
H	-2.71149000	1.92678900	-2.03643400
C	-3.28247100	-1.67754700	-2.69820900
H	-3.53318100	-0.67302200	-2.33803200
C	-1.94808100	-1.57668400	-3.44295300
H	-1.15323600	-1.21458200	-2.78028800
H	-1.64252400	-2.55347600	-3.83495500
H	-2.03178300	-0.88470100	-4.28844800
C	-4.40879500	-2.11949900	-3.63379100
H	-4.52420900	-1.40013300	-4.45147700
H	-4.20128900	-3.09646700	-4.08354400

H	-5.36410300	-2.19103400	-3.10364700
C	-0.86295000	2.38073700	-2.99641800
H	-0.18330900	3.22265500	-3.17040200
H	-0.29684800	1.58758200	-2.49430500
H	-1.18782200	2.00408400	-3.97250300
C	-2.89787300	3.87722200	-2.87820800
H	-2.30684200	4.77891300	-3.07268400
H	-3.24995700	3.49873000	-3.84370300
H	-3.77144300	4.17133000	-2.28716100
C	2.51798800	-1.83345800	-0.37394800
H	1.97219200	-2.49887300	-1.05033000
C	3.95670700	-1.63930200	-0.90846700
H	3.84936100	-1.45561400	-1.98295600
C	4.66441000	-0.44970300	-0.29222900
H	3.81326200	0.38033200	-0.22152300
C	2.37006100	0.62425800	-0.13312700
Br	2.23981800	2.40758100	0.13532800
H	-5.40241900	-0.50169000	0.16674900
H	-4.72259300	2.18678000	0.49099800
H	2.50441700	-2.32398600	0.60505000
C	4.80908700	-2.91817800	-0.75042500
H	4.21700100	-3.78134500	-1.07977800
H	5.63950000	-2.83162300	-1.46349700
C	5.07918900	-0.72256300	1.14332500
C	5.40642000	-3.18271000	0.64021000
C	6.05179500	-1.90587000	1.18974900
H	6.94829000	-1.66154900	0.60466100
H	6.38681300	-2.06413000	2.22152900
H	5.52621500	0.16513600	1.59818300
H	4.18606900	-0.95666600	1.73574500
H	6.20757600	-3.91749600	0.48408200
C	5.69951500	0.22980000	-1.20305400
H	6.36973100	-0.57295200	-1.55087000
C	6.54474500	1.27017300	-0.47247900
H	5.91221000	2.04594700	-0.02247800
H	7.21964300	1.76517700	-1.17764700
H	7.16152100	0.83239100	0.31766300
C	5.05152600	0.86667700	-2.43364800
H	4.40718000	1.70453100	-2.13936500
H	4.44555500	0.16511000	-3.01421800
H	5.82196000	1.26303500	-3.10187700
C	4.44125800	-3.80947300	1.64819300
H	3.89035800	-4.64657400	1.20530000
H	3.70782200	-3.09745600	2.04223100
H	4.99679900	-4.19698200	2.50896400

Cartesian coordinates of the optimized geometry for TS-**2b** at PBE0-D3BJ/6-31G*,def2-TZVP level of theory: (number of imaginary frequencies = 1):

Au	-0.20656500	0.17031100	-0.24427700
C	1.78475500	-0.12101900	-0.55267000
C	-2.18483400	0.29671700	0.15856900
C	-4.29165100	-0.34894200	0.62949600
C	-4.26422600	1.00840900	0.64932800
N	-3.00910400	-0.76184800	0.32802400
N	-2.96570000	1.37946100	0.35909200
C	-2.48152600	2.72704700	0.28314100
C	-2.53652000	3.38239400	-0.95423800
C	-1.94992600	3.30669700	1.44276300
C	-2.03028400	4.68198200	-1.00769200
C	-1.45829800	4.60859100	1.33451400
C	-1.49858300	5.28904300	0.12366700
H	-2.05065400	5.22435600	-1.94842200
H	-1.03768100	5.09470100	2.21024400
H	-1.11229000	6.30238700	0.06091100

C	-2.57685600	-2.12563200	0.21413200
C	-2.63835000	-2.73979200	-1.04402800
C	-2.08829400	-2.76602600	1.36129900
C	-2.20441100	-4.06401400	-1.12878300
C	-1.66897900	-4.09018200	1.22248000
C	-1.73182500	-4.73534700	-0.00722600
H	-2.23728400	-4.57485800	-2.08679600
H	-1.28725800	-4.62185800	2.08950700
H	-1.40883900	-5.76929100	-0.09250900
C	-1.87186700	2.56132300	2.75988100
H	-2.40099300	1.60824200	2.64640000
C	-0.41688300	2.23595700	3.10924800
H	0.17006400	3.15193800	3.24319300
H	0.05226300	1.64371900	2.31460800
H	-0.36585100	1.66260100	4.04180400
C	-2.55649100	3.33169600	3.89017100
H	-2.54093400	2.74296700	4.81368400
H	-3.59980600	3.55587600	3.64513900
H	-2.05014300	4.28106400	4.09599400
C	-1.97266800	-2.06253900	2.69807300
H	-2.42081900	-1.06713900	2.60174000
C	-2.73388100	-2.80392500	3.79822700
H	-2.68090500	-2.24518300	4.73879200
H	-2.31141200	-3.79812200	3.98067000
H	-3.78919200	-2.93256400	3.53614500
C	-0.50089200	-1.86493900	3.07207600
H	0.02548500	-1.29606400	2.29698600
H	0.00922100	-2.82797400	3.19036900
H	-0.41631700	-1.31919800	4.01859000
C	-3.11100600	-2.00659500	-2.28236200
H	-3.48513100	-1.02297900	-1.97654200
C	-3.07432600	2.70895500	-2.20055600
H	-3.56321900	1.77490300	-1.90040400
C	-1.92526000	2.34163600	-3.14420400
H	-1.19806800	1.69042700	-2.64544500
H	-1.39713300	3.23944300	-3.48543500
H	-2.30682100	1.81747100	-4.02763400
C	-4.12458200	3.56430900	-2.91044800
H	-4.54436300	3.01632800	-3.76069300
H	-3.69481900	4.49337700	-3.30039300
H	-4.94537600	3.83144900	-2.23678700
C	-1.93993400	-1.77230700	-3.24066200
H	-1.52297000	-2.72219700	-3.59408100
H	-1.13530600	-1.21386400	-2.74883400
H	-2.27016800	-1.20199800	-4.11597100
C	-4.26145300	-2.73966600	-2.97405200
H	-3.94717100	-3.71916000	-3.35080100
H	-4.62031900	-2.15787400	-3.82975000
H	-5.10278600	-2.89807500	-2.29130100
C	2.81577700	0.95515000	-0.42485100
H	2.55818500	1.82629400	-1.03769000
C	4.22468500	0.44480600	-0.79285400
H	4.30205200	0.46806100	-1.88446400
C	4.41666100	-0.97398400	-0.37273900
H	3.30580700	-1.52414800	-1.15967500
C	2.21942800	-1.35693700	-0.78531100
Br	1.39433100	-3.01565600	-0.63078000
H	-5.03538600	1.73858600	0.83865500
H	-5.09189900	-1.05240600	0.79814100
H	5.03442300	-1.56209500	-1.06131800
H	2.79896500	1.31042100	0.61408000
C	5.36028200	1.33226000	-0.19184700
H	5.00617800	2.37204600	-0.24831800
C	4.61801000	-1.38050300	1.05476800
C	5.55831200	0.97579000	1.28401800
C	5.83653500	-0.50701300	1.48821100

H	6.72960300	-0.80740100	0.92627200
H	6.03851700	-0.73192400	2.54133600
H	4.93404800	-2.42968900	1.06551200
H	6.37487700	1.57075900	1.70250800
H	4.66741100	1.26511700	1.85470900
C	6.64198700	1.24492600	-1.05514100
H	6.86340000	0.18385400	-1.24933500
C	6.44240300	1.94017800	-2.40280000
H	6.27327500	3.01408200	-2.25605500
H	5.59644500	1.54576000	-2.97583400
H	7.33400000	1.82859700	-3.02774000
C	7.85682000	1.85378500	-0.35675000
H	8.71107000	1.87950700	-1.04071200
H	8.16552400	1.28685900	0.52705000
H	7.65443500	2.88620000	-0.04502800
C	3.45410800	-1.23418600	2.03276000
H	2.63440000	-1.90625300	1.76855100
H	3.05475200	-0.21926400	2.08110100
H	3.80184100	-1.50431600	3.03499200

Cartesian coordinates of the optimized geometry for TS-**2c** at PBE0-D3BJ/6-31G*,def2-TZVP level of theory: (number of imaginary frequencies = 1):

Au	-0.25296400	-0.29947100	-0.15226800
C	1.74594500	-0.76058700	-0.39854100
C	-2.16781100	0.26092100	0.12153700
C	-3.94468300	1.58572300	0.51244200
C	-4.40259200	0.31621900	0.36302600
N	-2.57320900	1.52760900	0.36097500
N	-3.29663800	-0.47649200	0.12418500
C	-3.31432900	-1.89522900	-0.08744400
C	-3.15983200	-2.73231100	1.02564700
C	-3.45246000	-2.36755700	-1.39954700
C	-3.14811300	-4.10775600	0.78806100
C	-3.43353000	-3.75130100	-1.58078900
C	-3.28414900	-4.61169900	-0.49969100
H	-3.02942600	-4.79229700	1.62308100
H	-3.53482600	-4.15991300	-2.58198100
H	-3.27384600	-5.68559200	-0.66259100
C	-1.67157300	2.64085600	0.44787500
C	-1.13135100	2.96157100	1.70109300
C	-1.35311800	3.33217500	-0.72929700
C	-0.25112600	4.04396300	1.75730300
C	-0.46799700	4.40626400	-0.61769200
C	0.07164100	4.76398500	0.61257100
H	0.18456400	4.32694100	2.71129100
H	-0.20063600	4.97038400	-1.50667700
H	0.74801200	5.61181900	0.68009000
C	-3.57424700	-1.43345900	-2.58653000
H	-3.72038700	-0.41525100	-2.20754300
C	-2.27839800	-1.43639100	-3.40283100
H	-2.07756900	-2.43142200	-3.81604500
H	-1.42140700	-1.14888600	-2.78239200
H	-2.34950800	-0.73131300	-4.23864400
C	-4.78255000	-1.77079300	-3.46127300
H	-4.88261200	-1.03494100	-4.26620700
H	-5.71013100	-1.76979300	-2.87959200
H	-4.68139600	-2.75607700	-3.92898500
C	-1.90315600	2.92997700	-2.08216200
H	-2.61637300	2.11146600	-1.93293300
C	-2.65782600	4.08025400	-2.75112800
H	-3.09110900	3.74756100	-3.70032300
H	-1.99439700	4.92438500	-2.96902400
H	-3.46962500	4.44864500	-2.11535500
C	-0.78148500	2.40028500	-2.97981500

H	-0.26838500	1.55307900	-2.50988600
H	-0.03544700	3.17723100	-3.18170500
H	-1.18700000	2.06533400	-3.94082100
C	-1.44199300	2.16065600	2.94886900
H	-2.20694700	1.41718200	2.69778500
C	-2.97990500	-2.19141900	2.42965300
H	-3.12339200	-1.10512200	2.39998900
C	-1.55562800	-2.45046800	2.92852700
H	-0.81534000	-1.99956900	2.25717300
H	-1.34810300	-3.52489700	2.98824400
H	-1.41635800	-2.02428700	3.92832900
C	-4.01983500	-2.76241000	3.39535500
H	-3.90689200	-2.30770900	4.38532500
H	-3.90680400	-3.84529200	3.51564700
H	-5.03895400	-2.56962600	3.04455300
C	-0.19944900	1.39757500	3.41669500
H	0.60725200	2.08642900	3.69208800
H	0.17599600	0.73507800	2.62810500
H	-0.43440600	0.78601800	4.29480800
C	-2.00808100	3.04208500	4.06350300
H	-1.27725600	3.78540500	4.40034000
H	-2.27797600	2.42932500	4.93011500
H	-2.90300800	3.57921200	3.73260500
C	2.33342500	-2.10842000	-0.68094400
H	1.79610900	-2.87800600	-0.11752900
H	3.83123100	-0.03513400	-0.41837500
C	2.43092100	0.32349700	-0.25719800
Br	2.45994400	2.10865300	0.03836600
H	-5.39858700	-0.09617900	0.40292900
H	-4.45713100	2.51417500	0.71015300
H	2.17275200	-2.33812900	-1.74160400
C	4.55683200	-0.91009000	-0.81438200
C	5.87911900	-0.58412300	-0.13863600
C	3.83018800	-2.13529800	-0.29345300
C	5.85890500	-0.74916700	1.37361300
H	6.63215200	-1.26457500	-0.56446100
H	6.19516300	0.42710800	-0.41452300
C	3.95064500	-2.32261900	1.22312400
H	4.26887700	-3.00823200	-0.79686400
C	5.35892300	-2.13289900	1.77450100
H	6.86481100	-0.57228300	1.77187400
H	5.20588900	0.01373800	1.82455800
H	3.57078800	-3.31932500	1.48085000
H	3.28534100	-1.60112700	1.72167200
H	6.01993200	-2.88130700	1.30918000
C	4.54301400	-0.74060400	-2.33948600
H	3.50387500	-0.87456900	-2.66653200
C	5.38970600	-1.80615900	-3.04197300
H	6.44901600	-1.71649800	-2.77860400
H	5.31146200	-1.67963900	-4.12652100
H	5.06400300	-2.82431100	-2.80572000
C	4.97642100	0.65795900	-2.77644300
H	6.04442500	0.82641300	-2.60301900
H	4.41770300	1.44094900	-2.25085600
H	4.79692700	0.78661800	-3.84838800
C	5.38916900	-2.33212300	3.28376500
H	6.40350200	-2.21095100	3.67867000
H	5.03768600	-3.33191200	3.56134400
H	4.74495200	-1.59929000	3.78572100

Cartesian coordinates of the optimized geometry for TS-**2d** at PBE0-D3BJ/6-31G*,def2-TZVP level of theory: (number of imaginary frequencies = 1):

Au	-0.20477300	0.15958700	-0.25242200
C	1.78876700	0.18955000	-0.65615100

C	-2.16927500	-0.01206200	0.20061900
C	-4.11838500	-0.94391100	0.84149800
C	-4.34777900	0.37583400	0.61989400
N	-2.77990700	-1.15765300	0.57910500
N	-3.14186600	0.92394100	0.22798800
C	-2.92416300	2.30043800	-0.10970100
C	-2.49657900	3.17264700	0.89998900
C	-3.12143500	2.69445400	-1.44011000
C	-2.26536100	4.50056600	0.53664300
C	-2.87523700	4.03286300	-1.74969200
C	-2.45346600	4.92645800	-0.77238300
H	-1.93281500	5.20920900	1.28986000
H	-3.01319400	4.37911200	-2.76982000
H	-2.27004400	5.96474500	-1.03386000
C	-2.09735400	-2.41508900	0.68935700
C	-1.50776200	-2.74566000	1.91718700
C	-2.02748300	-3.23505700	-0.44489300
C	-0.83923100	-3.96856100	1.99442400
C	-1.34717200	-4.44710000	-0.31407600
C	-0.76443100	-4.81366500	0.89339500
H	-0.37015100	-4.26113900	2.92945900
H	-1.27311200	-5.11137500	-1.17038700
H	-0.24726600	-5.76561200	0.97701500
C	-3.53451000	1.71335500	-2.51874800
H	-3.84305900	0.78057400	-2.03273900
C	-2.34193200	1.38555600	-3.42228200
H	-1.98682100	2.28341400	-3.94118900
H	-1.50736000	0.97811200	-2.83982500
H	-2.62596400	0.64657600	-4.18004000
C	-4.72658300	2.21735200	-3.33304000
H	-5.04526600	1.45117000	-4.04778000
H	-5.57801500	2.45905900	-2.68848000
H	-4.47517700	3.11549700	-3.90751000
C	-2.62138100	-2.82923500	-1.77794700
H	-3.16633800	-1.88872000	-1.63857000
C	-3.62059400	-3.86606800	-2.29391600
H	-4.07510000	-3.52153100	-3.22896800
H	-3.13517600	-4.82682000	-2.49770800
H	-4.42213400	-4.04367500	-1.56932000
C	-1.51080600	-2.56612400	-2.79858200
H	-0.81958600	-1.79679000	-2.43554900
H	-0.93060000	-3.47505700	-2.99428000
H	-1.93743000	-2.22656300	-3.74898100
C	-1.54565500	-1.81557200	3.11229100
H	-2.18227700	-0.95978400	2.86059000
C	-2.25857500	2.71212600	2.32376700
H	-2.58674200	1.66969600	2.40625100
C	-0.76486300	2.74769700	2.65853200
H	-0.19250500	2.12557000	1.96035700
H	-0.37262500	3.76974100	2.60332200
H	-0.59088500	2.37399200	3.67383800
C	-3.07405000	3.52887900	3.32769000
H	-2.93081600	3.13667000	4.34028600
H	-2.76777100	4.58066500	3.33516700
H	-4.14353100	3.49399000	3.09554400
C	-0.14580000	-1.27131400	3.41046400
H	0.54287200	-2.07996700	3.68052300
H	0.26815900	-0.75337500	2.53768800
H	-0.18094400	-0.56434600	4.24697600
C	-2.15476900	-2.49349800	4.34070200
H	-1.54429100	-3.33779500	4.67935200
H	-2.22316900	-1.78238500	5.17090800
H	-3.16074800	-2.87158800	4.13070300
C	2.52263100	1.37826900	-1.20430600
H	2.18553600	2.30697400	-0.73707300
H	3.65810500	-0.81595300	-0.37699100

C	2.50957000	-0.91021400	-0.45292500
Br	2.00806600	-2.67602800	-0.15542600
H	-5.24326500	0.97175400	0.70161200
H	-4.77176100	-1.74206600	1.15730100
H	2.24573300	1.45902700	-2.26530800
C	4.39225300	-0.17201500	-1.52922400
C	5.68906600	-0.83648200	-1.19735400
C	4.05916700	1.21010700	-1.09520400
H	3.92717600	-0.50050500	-2.46138400
C	6.18242800	-0.44005600	0.19122900
H	6.37049100	-0.37969700	-1.94365200
C	4.64866100	1.57510700	0.28338100
H	4.50233100	1.86301400	-1.86619400
C	6.08694100	1.06473600	0.40736500
H	7.21331500	-0.78804400	0.31690500
H	5.58244600	-0.96985400	0.94689200
H	4.04399500	1.04086900	1.03309600
H	6.48356600	1.31774600	1.39562900
H	6.72867200	1.57388900	-0.32481900
C	4.51115800	3.08038900	0.59734500
H	3.47011900	3.36088800	0.38691700
C	4.74156000	3.35761700	2.08200800
H	4.52797300	4.40640800	2.31260800
H	4.09235900	2.73736600	2.71071400
H	5.77993200	3.16815700	2.37643300
C	5.40805900	3.97112700	-0.26064800
H	6.46771800	3.82334200	-0.02507100
H	5.27542100	3.79986500	-1.33594200
H	5.17969800	5.02546400	-0.07449200
C	5.67037700	-2.34252900	-1.43972400
H	5.00084000	-2.84371200	-0.73245600
H	5.33680700	-2.58668100	-2.45370700
H	6.67378400	-2.75539800	-1.30353300

Cartesian coordinates of the optimized geometry for TS-**2e** at PBE0-D3BJ/6-31G*,def2-TZVP level of theory: (number of imaginary frequencies = 1):

Au	-0.58489600	-0.46890900	-0.13876900
C	2.83261600	-0.02187900	-0.47446200
C	-1.94251200	1.00037200	0.08910800
C	-3.81511100	2.20128800	0.40994500
C	-2.78651100	3.07838400	0.28150100
N	-3.27353000	0.93617100	0.28947700
N	-1.64872900	2.31986100	0.08610800
C	-0.32050100	2.82617100	-0.10491900
C	0.10673100	3.09274100	-1.41355800
C	0.49834100	2.99231700	1.02140900
C	1.40998800	3.56782700	-1.57562100
C	1.79108800	3.47532700	0.80376600
C	2.24092100	3.76555800	-0.47964300
H	1.77547200	3.78891400	-2.57434500
H	2.45079400	3.63070100	1.65316200
H	3.24785900	4.14631500	-0.62613200
C	-4.00157000	-0.29803200	0.36992700
C	-4.49848700	-0.85223500	-0.81707600
C	-4.14508100	-0.89981600	1.62729600
C	-5.17234900	-2.07014100	-0.71401100
C	-4.82702700	-2.11655200	1.67317500
C	-5.33548600	-2.69473700	0.51637200
H	-5.57040200	-2.53709600	-1.61018100
H	-4.95730600	-2.61982900	2.62676500
H	-5.86235300	-3.64278300	0.57414000
C	0.03935800	2.64328400	2.42230900
H	-1.00473400	2.31550400	2.36986200
C	0.85511900	1.47403000	2.98143400

H	1.91643800	1.73534600	3.06823300
H	0.77082800	0.59055000	2.33737500
H	0.49609900	1.20021600	3.97937500
C	0.09586300	3.85766900	3.35114600
H	-0.28995500	3.59546500	4.34191000
H	-0.50231000	4.68720600	2.96016700
H	1.12253300	4.21776300	3.48024300
C	-3.55778200	-0.29547900	2.88676800
H	-3.19251000	0.71004000	2.64671000
C	-4.60470200	-0.14629600	3.99178600
H	-4.16710300	0.35497500	4.86172800
H	-4.97880100	-1.11932700	4.32784800
H	-5.46279600	0.44325400	3.65273300
C	-2.35733800	-1.11740500	3.36500500
H	-1.58979700	-1.18659200	2.58532200
H	-2.65940700	-2.13710700	3.62894900
H	-1.90703700	-0.65824700	4.25239300
C	-4.29138500	-0.19667800	-2.16733500
H	-3.85391100	0.79523400	-2.00366600
C	-0.77454500	2.84750600	-2.62100800
H	-1.76578500	2.54385300	-2.26640600
C	-0.21902600	1.69601400	-3.46379700
H	-0.12772600	0.77990500	-2.86880800
H	0.77084500	1.94147700	-3.86559800
H	-0.88194600	1.48795900	-4.31062500
C	-0.95455500	4.11576000	-3.45721200
H	-1.63985000	3.92515100	-4.28979400
H	-0.00441500	4.45597100	-3.88345300
H	-1.36473700	4.93511400	-2.85777800
C	-3.29714300	-1.00337700	-3.00738700
H	-3.68138000	-2.00845500	-3.21437100
H	-2.33766200	-1.11187400	-2.48831200
H	-3.11498000	-0.50740600	-3.96738900
C	-5.61221700	0.00597700	-2.91165600
H	-6.08660300	-0.94969700	-3.15943500
H	-5.43802000	0.53951100	-3.85215700
H	-6.32256300	0.58734400	-2.31473300
C	4.19082200	-0.15658200	0.22622000
C	4.97140100	-1.32303400	-0.39548200
H	5.12825600	-1.07344000	-1.45717700
C	4.17388700	-2.61720900	-0.34411100
H	3.06052000	-2.26178000	-0.60266300
C	2.05368800	-1.26603600	-0.44009600
H	-2.75566900	4.15634400	0.30896900
H	-4.87040700	2.35323400	0.57406900
H	4.00859400	-0.37852500	1.28792500
C	4.06447100	-3.27768900	1.01527900
H	3.29563700	-4.05430300	1.00823200
H	3.82660800	-2.56435400	1.80943300
H	5.01945900	-3.75660700	1.26206600
C	4.47491100	-3.59140800	-1.46452500
H	5.50278200	-3.95989600	-1.34792000
H	3.80381600	-4.45404200	-1.43548400
H	4.39878700	-3.11576600	-2.44674400
C	0.91016200	-1.86391000	-0.33660100
Br	0.56843200	-3.69386800	-0.37174000
H	2.23723000	0.75527400	0.02085600
H	2.97970600	0.29942100	-1.51534200
C	4.98038300	1.14483200	0.13152300
H	5.08821300	1.42218600	-0.92903200
H	4.41374100	1.95428000	0.60871500
C	6.36923600	1.03284700	0.76071900
C	6.35291800	-1.44714000	0.26072700
H	6.23090300	-1.70078400	1.32190600
H	6.91367100	-2.26874300	-0.20123200
C	7.12982000	-0.13985100	0.14227200

H	8.10863700	-0.24696600	0.62478400
H	7.32633900	0.07787600	-0.91810100
H	6.23572600	0.81769400	1.83285100
C	7.13958000	2.33966300	0.62483000
H	8.12328600	2.27095000	1.10120400
H	7.29758300	2.59129500	-0.43126300
H	6.59938800	3.17174600	1.09067700

Cartesian coordinates of the optimized geometry for TS-**2f** at PBE0-D3BJ/6-31G*,def2-TZVP level of theory: (number of imaginary frequencies = 1):

Au	-0.21398900	0.13850200	-0.05316100
C	1.80282300	-0.09870400	-0.15488500
C	-2.23287100	0.22465500	0.06762000
C	-4.37486900	-0.46333000	0.20166200
C	-4.37885400	0.89434600	0.20880700
N	-3.05246100	-0.85006200	0.11496100
N	-3.05845800	1.29146100	0.12634100
C	-2.59962300	2.64964600	0.09825000
C	-2.46155500	3.27636400	-1.14740600
C	-2.28605800	3.26946900	1.31509600
C	-1.98610400	4.58868500	-1.14916200
C	-1.81453600	4.58194000	1.25684700
C	-1.66718100	5.23479600	0.03905500
H	-1.86245800	5.11007300	-2.09399400
H	-1.55857100	5.09824300	2.17763100
H	-1.30099000	6.25726400	0.01575400
C	-2.58134300	-2.20497600	0.07626500
C	-2.42698700	-2.81920700	-1.17396100
C	-2.27210800	-2.83624000	1.28866100
C	-1.95923600	-4.13440700	-1.18435600
C	-1.80741900	-4.15090600	1.22246700
C	-1.65840800	-4.79616200	0.00034200
H	-1.82783300	-4.64541900	-2.13385700
H	-1.55884600	-4.67491200	2.14095700
H	-1.30432000	-5.82300300	-0.02915100
C	-2.40974100	2.55201900	2.64400100
H	-2.91102100	1.59305000	2.46978100
C	-1.02355700	2.24470000	3.21751000
H	-0.46911300	3.16819100	3.42067900
H	-0.43465600	1.64190300	2.51617600
H	-1.11214400	1.68969600	4.15826300
C	-3.26277300	3.33828800	3.64066000
H	-3.38919800	2.76466800	4.56509700
H	-4.25601300	3.55387800	3.23341400
H	-2.79615700	4.29279600	3.90750200
C	-2.38942300	-2.13047100	2.62390000
H	-2.84321000	-1.14761900	2.45345500
C	-3.29596100	-2.89203400	3.59222600
H	-3.40814000	-2.33150700	4.52649100
H	-2.88127800	-3.87410000	3.84477200
H	-4.29238800	-3.04957700	3.16637800
C	-1.00147900	-1.89367800	3.22562800
H	-0.37113400	-1.31588900	2.53974200
H	-0.49344500	-2.84263500	3.43187900
H	-1.08260700	-1.34172800	4.16877900
C	-2.71013200	-2.09638300	-2.47469400
H	-3.13136400	-1.11317400	-2.23626300
C	-2.76947400	2.56497500	-2.44944600
H	-3.25350100	1.61064100	-2.21212500
C	-1.47492800	2.24577400	-3.20260600
H	-0.80330100	1.63485800	-2.58830200
H	-0.94316500	3.16455900	-3.47553600
H	-1.69334700	1.69529100	-4.12459400
C	-3.73920000	3.36261500	-3.32261700

H	-3.99491500	2.79138000	-4.22141600
H	-3.30141300	4.31163500	-3.65078300
H	-4.66640000	3.58942400	-2.78618300
C	-1.40983000	-1.86058700	-3.24796300
H	-0.94020400	-2.80970100	-3.53022600
H	-0.69075100	-1.29565800	-2.64386200
H	-1.60801000	-1.29605400	-4.16589100
C	-3.73942800	-2.84186300	-3.32586500
H	-3.36783600	-3.82375300	-3.63893400
H	-3.96610600	-2.27079700	-4.23255600
H	-4.67424600	-2.99770100	-2.77728300
C	2.79894600	1.01471300	0.02861600
H	2.52004000	1.89562800	-0.55744900
C	4.21772900	0.53369200	-0.28855100
H	4.36300000	0.50790300	-1.37776100
C	4.37961400	-0.84881800	0.22631400
H	3.40295100	-1.45485700	-0.67488400
C	2.30353400	-1.32419800	-0.32961400
Br	1.51865600	-3.00635200	-0.19149200
H	-5.18507900	1.60899800	0.26316000
H	-5.17705400	-1.18301600	0.24892600
H	2.74078100	1.32053700	1.08500500
C	5.37476500	1.36536400	0.32611500
C	6.69693700	0.68483000	-0.05032000
C	5.57454200	-1.62328800	-0.21437100
C	6.73963900	-0.77849900	0.37888100
H	5.65907300	-1.57710300	-1.30870600
H	7.67565400	-1.25290700	0.06090500
H	6.70554400	-0.85299800	1.47450800
H	7.53261200	1.21409000	0.41885700
H	6.85216200	0.75202400	-1.13512300
H	4.09887500	-1.00706300	1.27035000
H	5.26945600	1.30919100	1.42161900
C	5.28061200	2.85664300	-0.05212000
H	4.31118200	3.20972600	0.32723800
C	6.36087200	3.67024800	0.65941700
H	7.35785700	3.45968500	0.25674900
H	6.17806300	4.74150600	0.52717800
H	6.37873000	3.46491500	1.73608800
C	5.31285900	3.11455500	-1.55677200
H	6.25952400	2.79032600	-2.00360500
H	4.49716500	2.61161300	-2.08880800
H	5.21383100	4.18673100	-1.75477600
C	5.59705000	-3.06701300	0.26399300
H	5.53435000	-3.11932900	1.35684800
H	4.76069900	-3.63903300	-0.15114900
H	6.52620900	-3.55535600	-0.04391500

4. Supporting Information References

[1] M. J. Frisch, G. W. Trucks, H. B. Schlegel, G. E. Scuseria, M. A. Robb, J. R. Cheeseman, G. Scalmani, V. Barone, G. A. Petersson, H. Nakatsuji, X. Li, M. Caricato, A. V. Marenich, J. Bloino, B. G. Janesko, R. Gomperts, B. Mennucci, H. P. Hratchian, J. V. Ortiz, A. F. Izmaylov, J. L. Sonnenberg, D. Williams-Young, F. Ding, F. Lipparini, F. Egidi, J. Goings, B. Peng, A. Petrone, T. Henderson, D. Ranasinghe, V. G. Zakrzewski, J. Gao, N. Rega, G. Zheng, W. Liang, M. Hada, M. Ehara, K. Toyota, R. Fukuda, J. Hasegawa, M. Ishida, T. Nakajima, Y. Honda, O. Kitao, H. Nakai, T. Vreven, K. Throssell, J. A. Montgomery, Jr.; , J. E. Peralta, F. Ogliaro, M. J. Bearpark, J. J. Heyd, E. N. Brothers, K. N. Kudin, V. N. Staroverov, T. A. Keith, R. Kobayashi, J. Normand, K. Raghavachari, A. P. Rendell, J. C. Burant, S. S. Iyengar, J. Tomasi, M. Cossi, J. M. Millam, M. Klene, C. Adamo, R. Cammi, J. W. Ochterski, R. L. Martin, K. Morokuma, O. Farkas, J. B. Foresman, D. J. Fox, Gaussian 16, Revision A.03, Wallingford CT, **2016**.

[2] C. Adamo, V. Barone, *J. Chem. Phys.* **1999**, *110*, 6158-6170.

[3] S. Grimme, S. Ehrlich, L. Goerigk, *J. Comp. Chem.* **2011**, *32*, 1456-1465.

[4] R. Ditchfield, W. J. Hehre, J. A. Pople, *J. Chem. Phys.* **1971**, *54*, 724-728.

[5] W. J. Hehre, R. Ditchfield, J. A. Pople, *J. Chem. Phys.* **1972**, *56*, 2257-2261.

[6] F. Weigend, R. Ahlrichs, *Phys. Chem. Chem. Phys.* **2005**, *7*, 3297-3305.

[7] D. Andrae, U. Häußermann, M. Dolg, H. Stoll, H. Preuß, *Theor. Chim. Acta* **1990**, *77*, 123-141.

[8] R. Krishnan, J. S. Binkley, R. Seeger, J. A. Pople, *J. Chem. Phys.* **1980**, *72*, 650-654.

[9] A. D. McLean, G. S. Chandler, *J. Chem. Phys.* **1980**, *72*, 5639-5648.

[10] L. A. Curtiss, M. P. McGrath, J. P. Blaudeau, N. E. Davis, R. C. Binning Jr, L. Radom, *J. Chem. Phys.* **1995**, *103*, 6104-6113.

[11] T. Clark, J. Chandrasekhar, G. W. Spitznagel, P. V. R. Schleyer, *J. Comput. Chem.* **1983**, *4*, 294-301.

[12] A. V. Marenich, C. J. Cramer, D. G. Truhlar, *J. Phys. Chem. B* **2009**, *113*, 6378-6396.
