# Supplementary material for: Terpenes as Naturally Occurring Stereochemical Templates: Conformationally Driven Discovery of Reactivity
Source: Org Lett. 2025 Sep 23;27(39):11071–6. doi: 10.1021/acs.orglett.5c03431 (PMC12501937; doi:10.1021/acs.orglett.5c03431)

Supporting Information

Terpenes as Naturally Occurring Stereochemical Templates: Conformationally-Driven Discovery of Reactivity

Omar Arto,[a‡] Rubén Miguélez,[a,‡] Hannah Siera,[b] Jan Schulte,[b] Isabel Merino,[c] Gebhard Haberhauer,[b] Pablo Barrio[a*]

[a] Department of Organic and Inorganic Chemistry

Universidad de Oviedo

Julian Clavería 8 33006 Oviedo (Spain)

[b] Institut für Organische Chemie

Universität

Duisburg-Essen

Universitätstraße 7, 45117 Essen (Germany)

[c] Servicios Científico Técnicos

Universidad de Oviedo

Fernando Bonguera s/n, 30006 Oviedo (Spain)

Correspondence to: barriopablo@uniovi.es

This PDF file includes:

^{1}H NMN Spectra

^{13}C NMR Spectra

2D NMR Spectra

INDEX

Synthesis of starting materials

- Bridged family (1a-d)…………………………………………….4-41
- Fused family (2a-d)………………………………………………..42-80
- Exo family (2e-g)…………………………………………………….81-113

Crude catalytic reactions and characterization of products

- Bridged family……………………………………………………..115-132
- Fused family…………………………………………………………..133-178
- Exo family……………………………………………………………….179-188

SYNTHESIS OF STARTING MATERIALS

1a,b

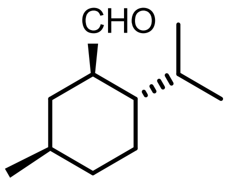

1a-CHO

^{1}H NMR(300 MHz, $CDCl_3$)

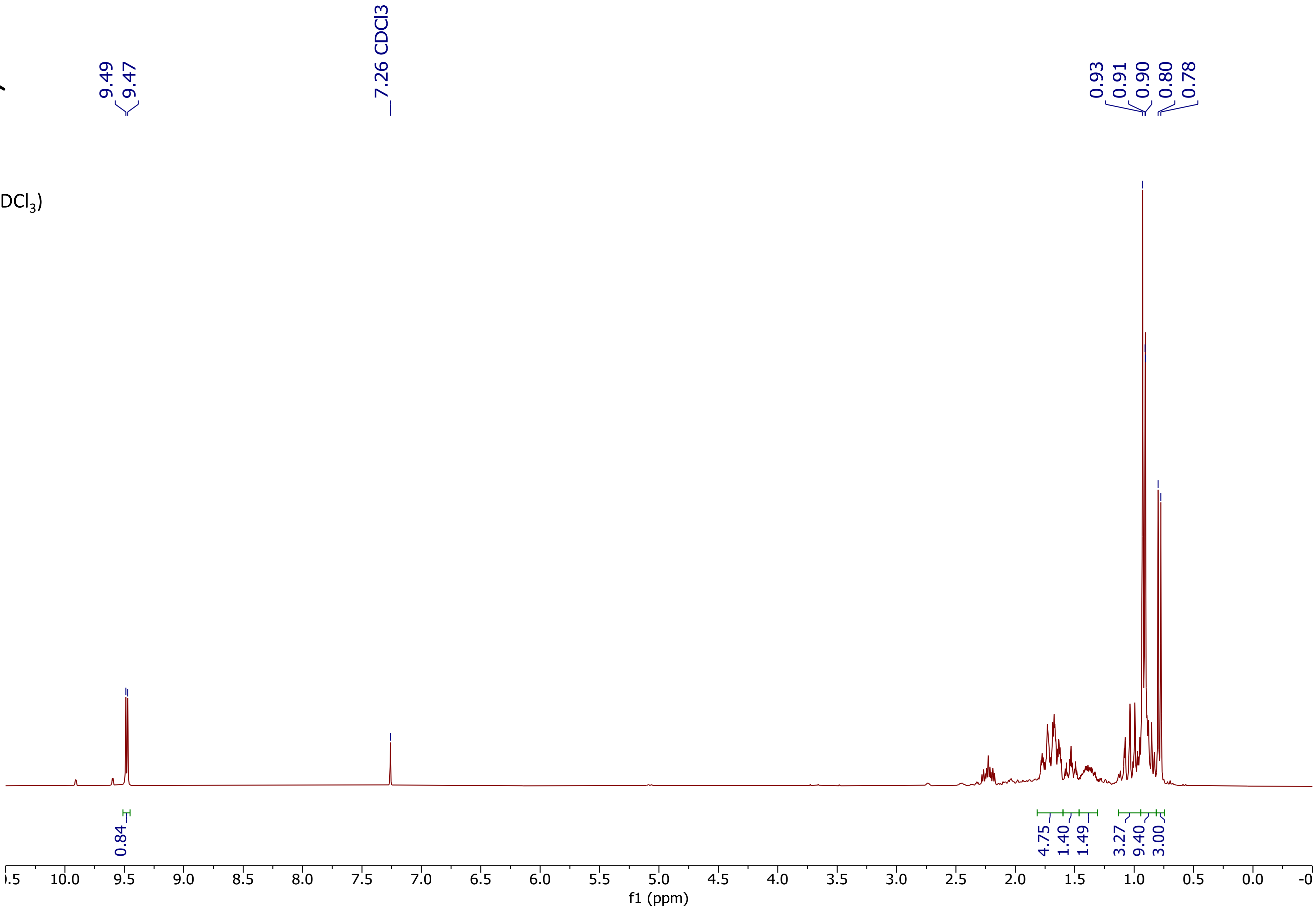

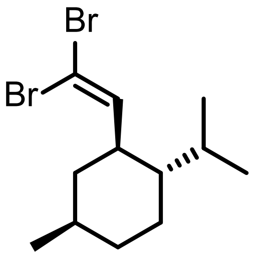

1a-CBr$_2$

^{1}H NMR(300 MHz, $CDCl_3$)

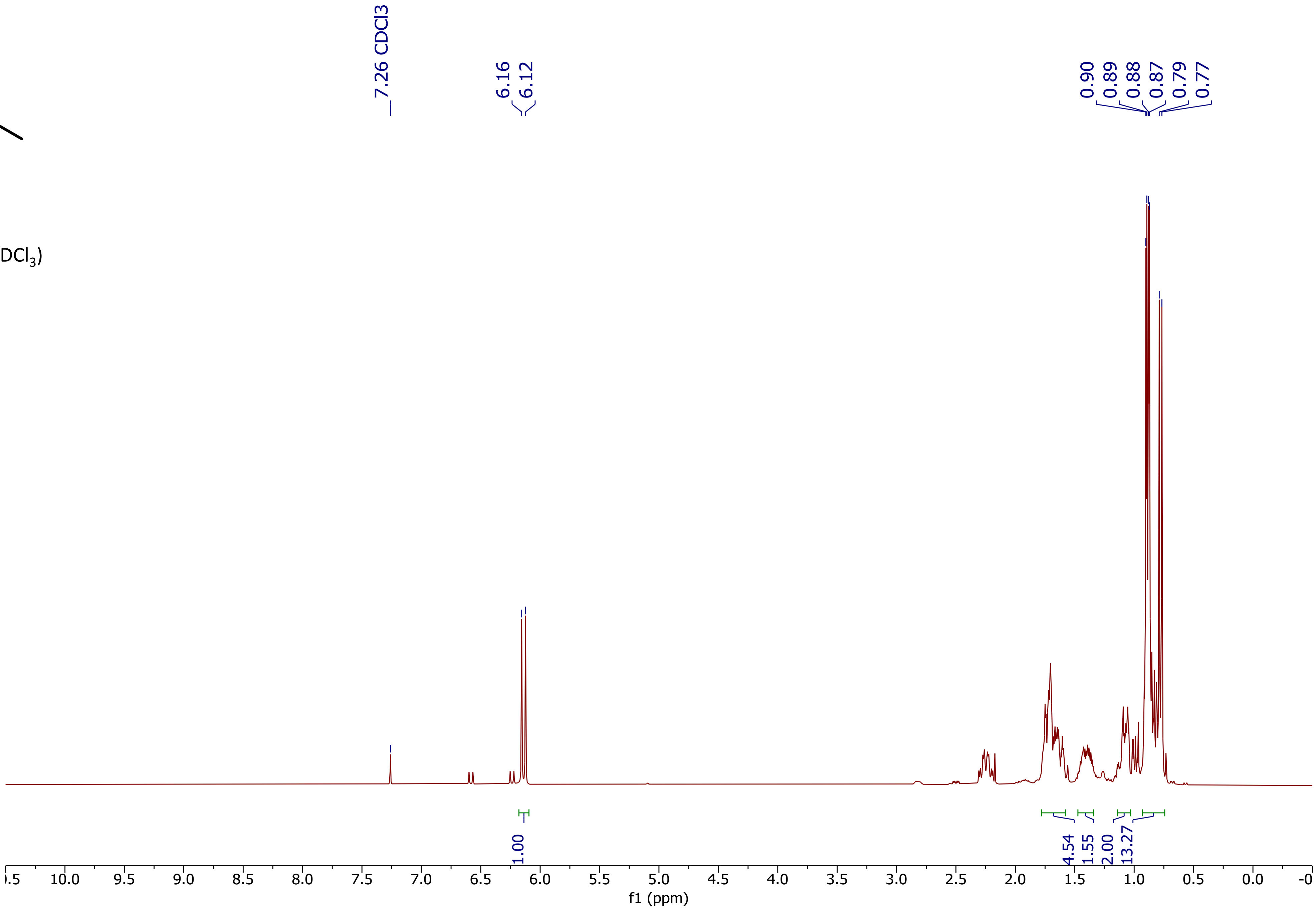

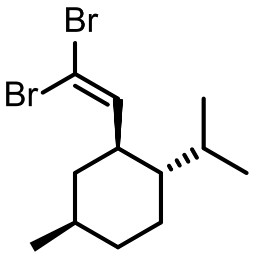

1a-CBr$_2$

^{13}C NMR (75 MHz, $CDCl_3$)

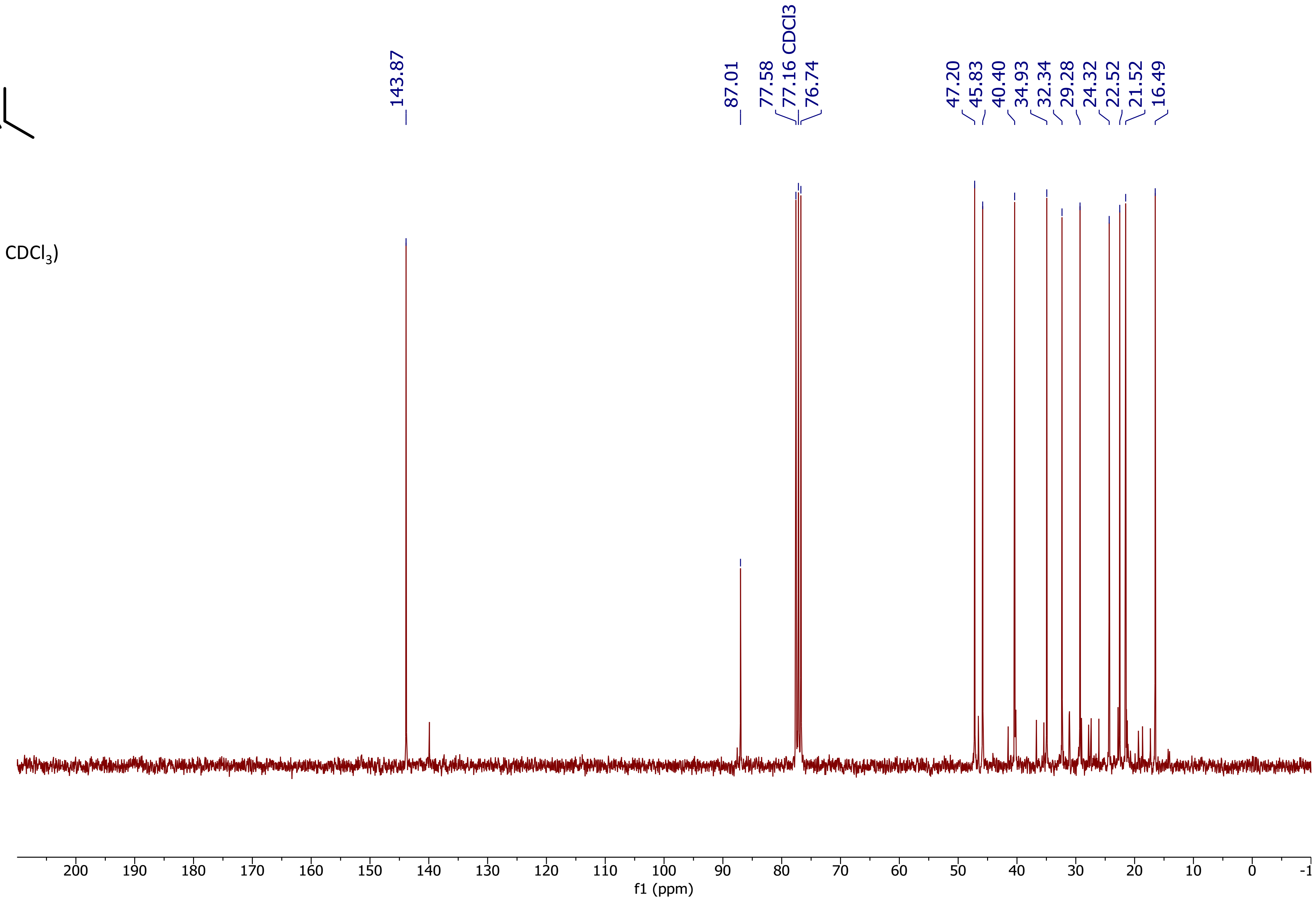

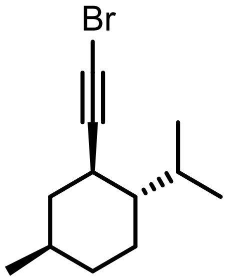

1a

^{1}H NMR(300 MHz, $CDCl_3$)

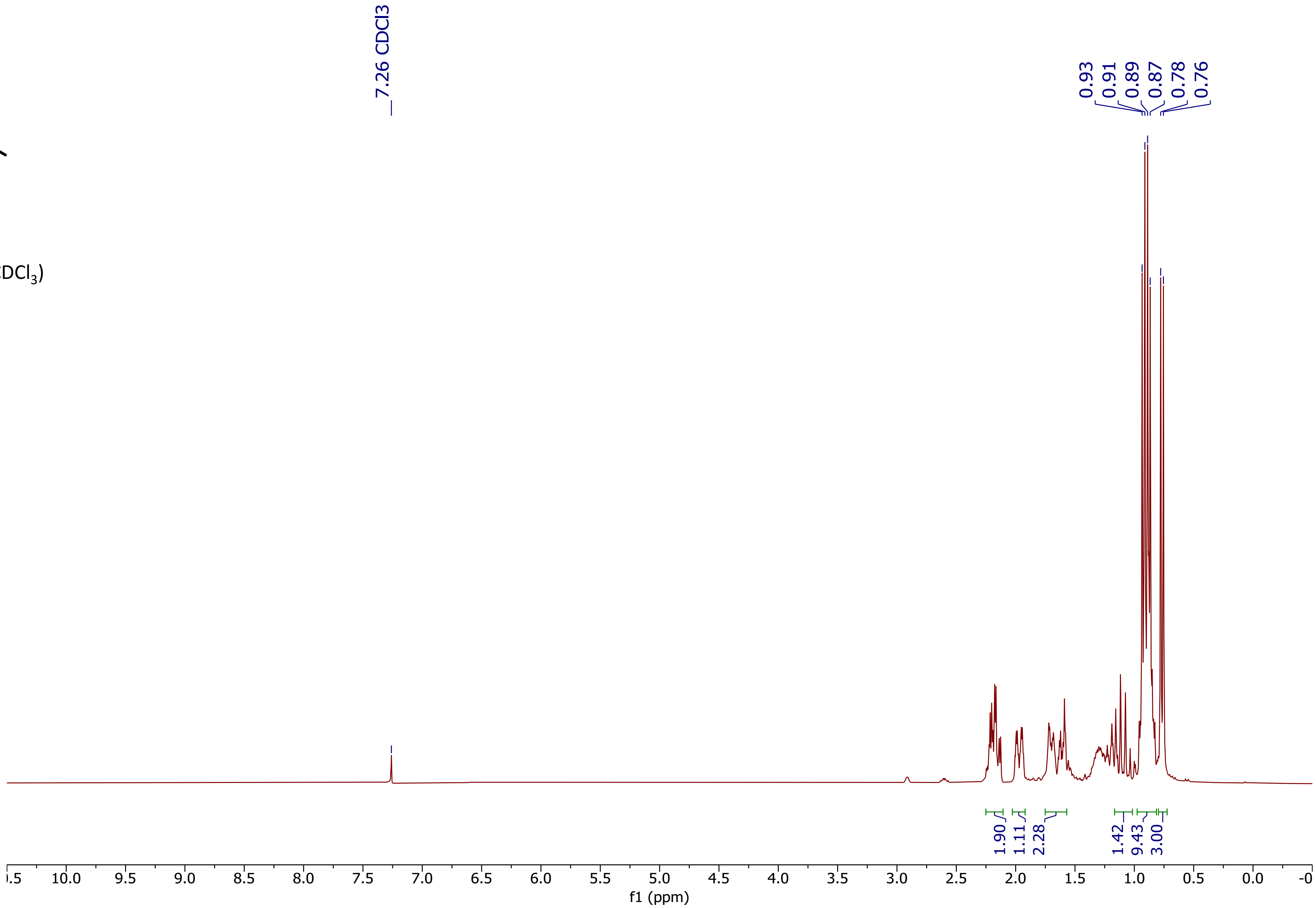

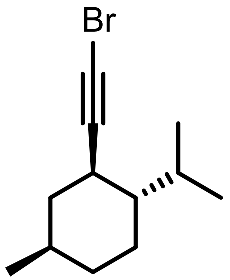

1a

^{13}**C NMR** (75 MHz, $CDCl_3$)

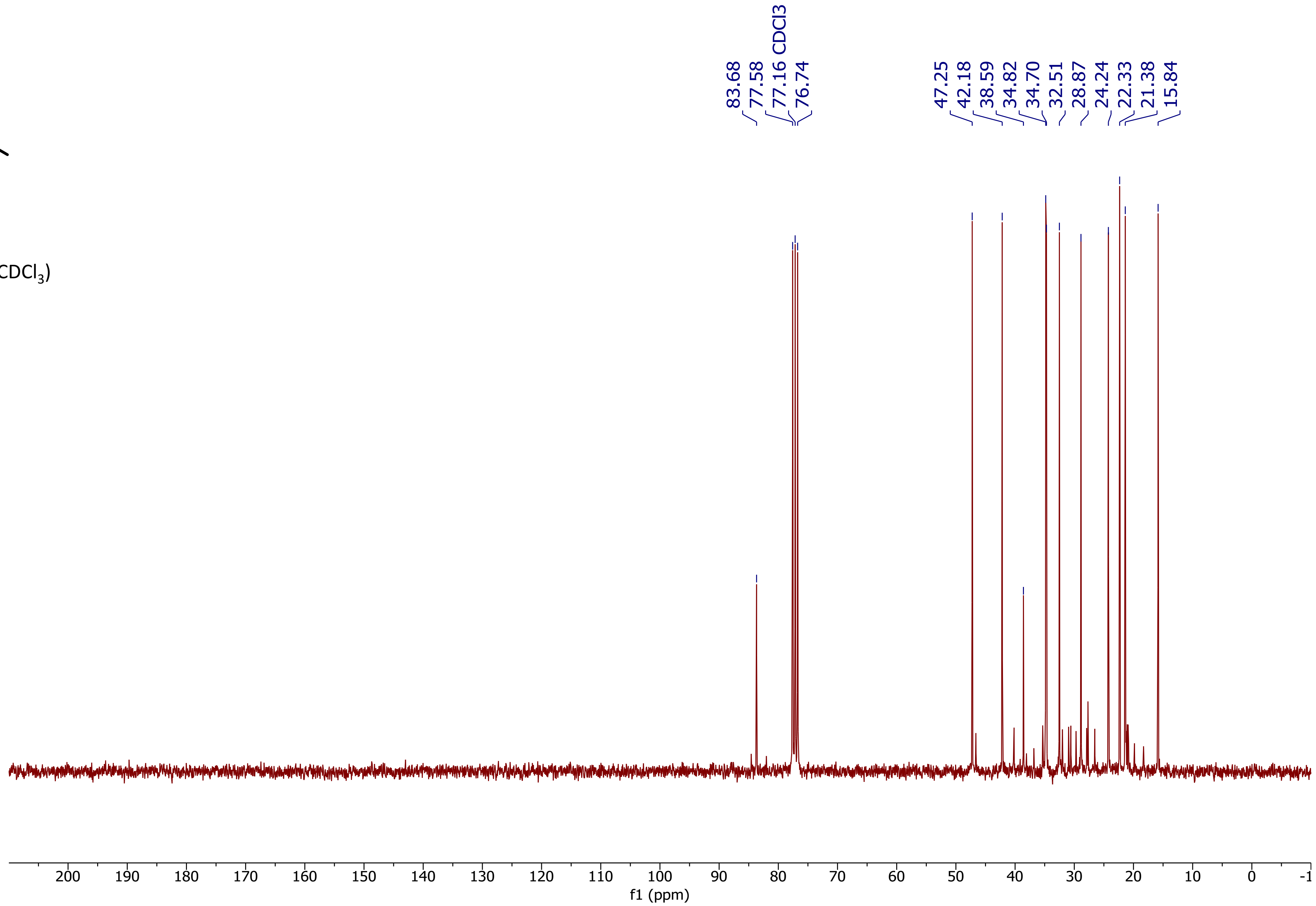

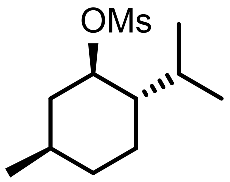

1b-OMs

-crude-

^{1}H NMR(300 MHz, $CDCl_3$)

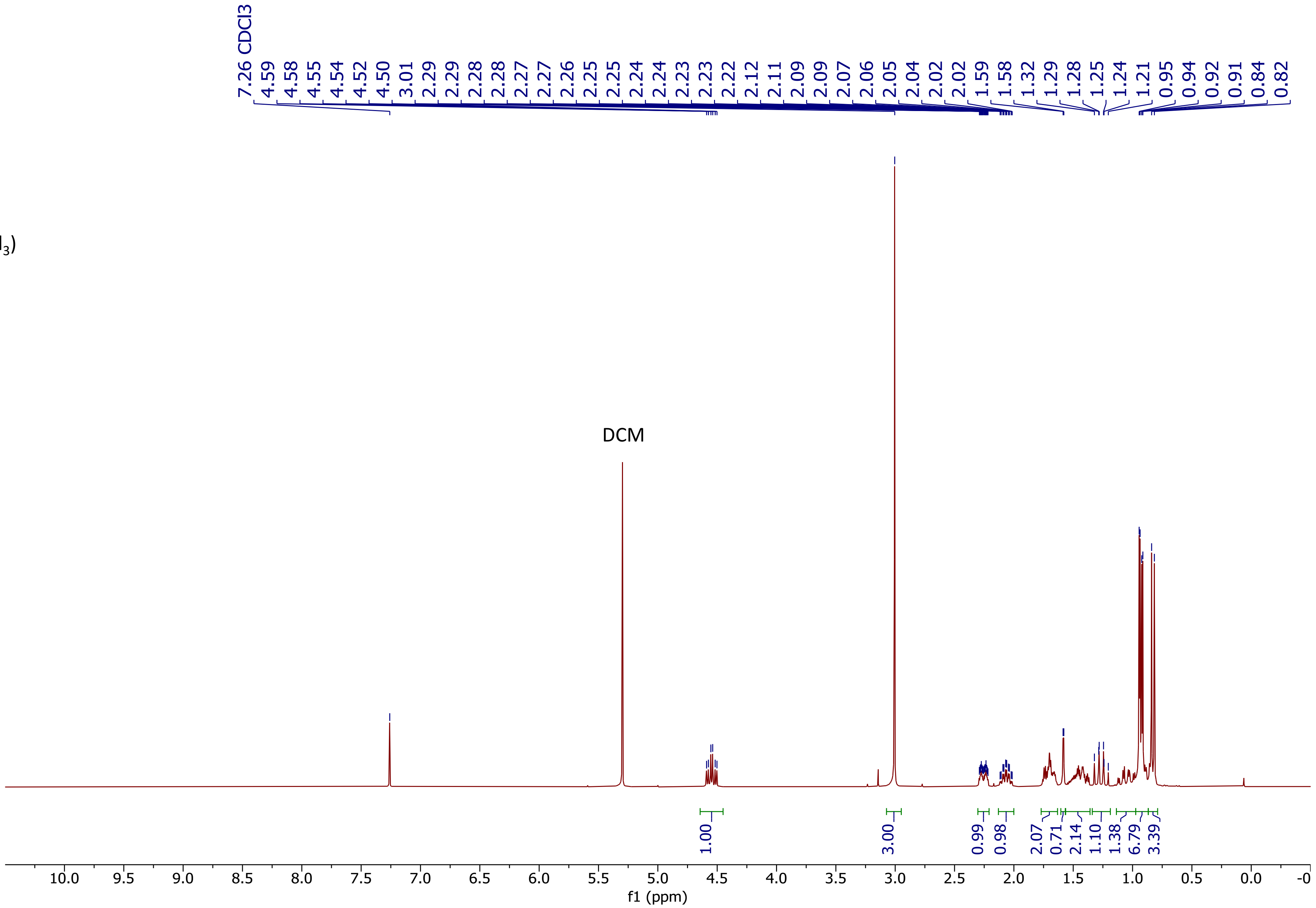

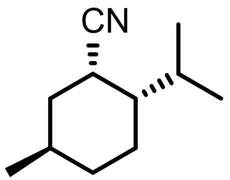

1b-CN

^{1}H NMR(300 MHz, $CDCl_3$)

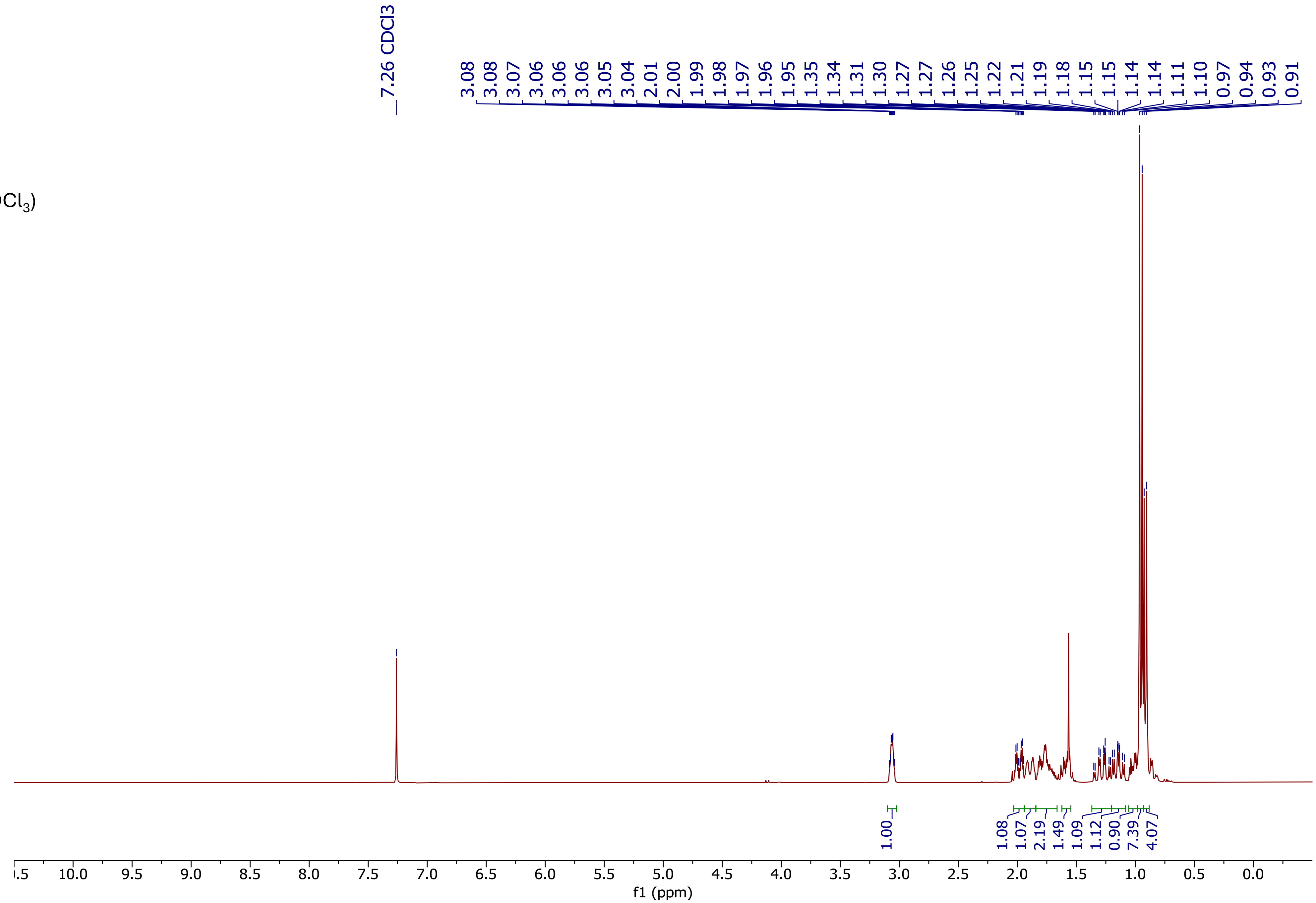

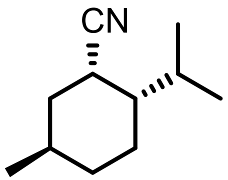

1b-CN

^{13}C NMR (75 MHz, $CDCl_3$)

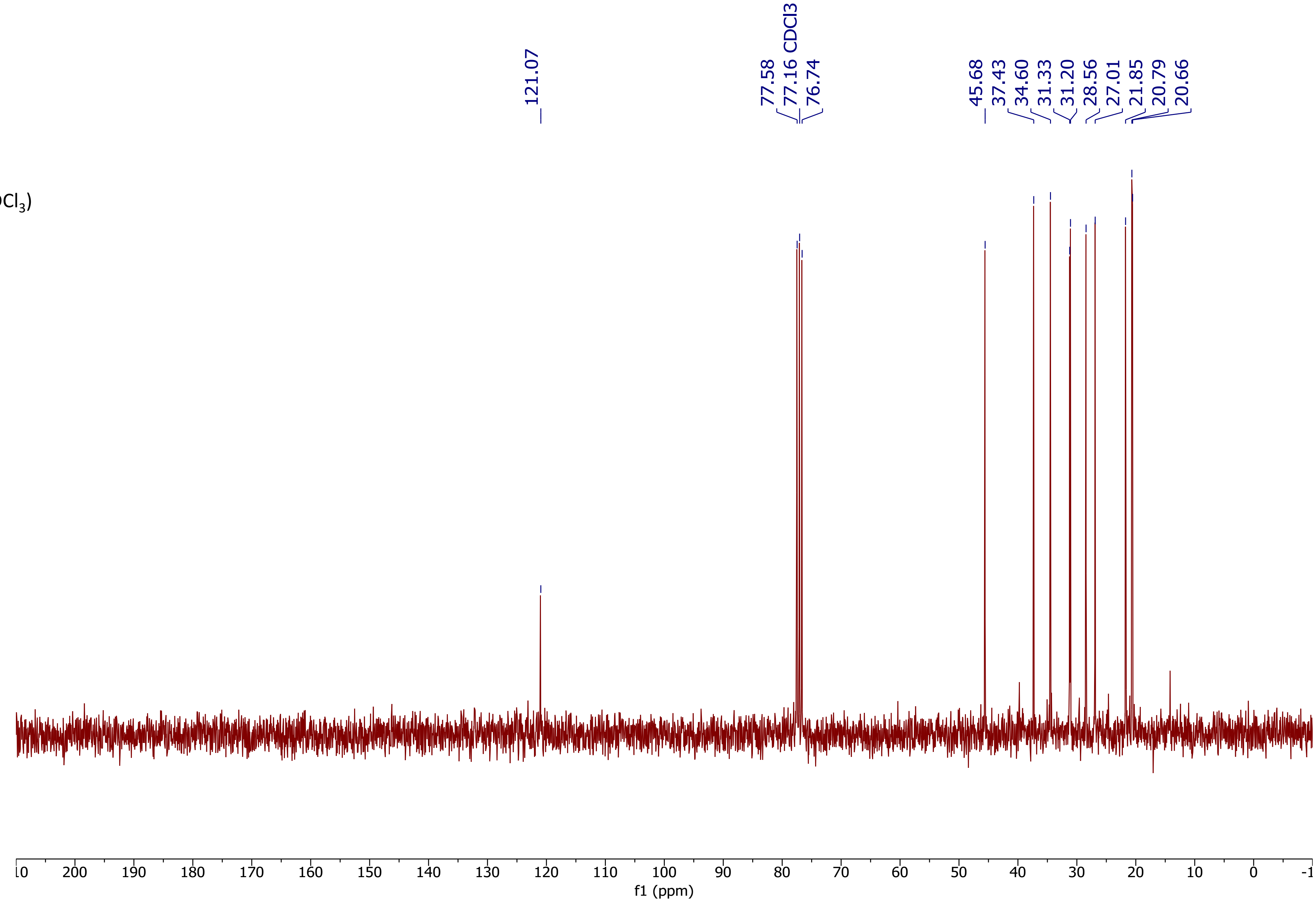

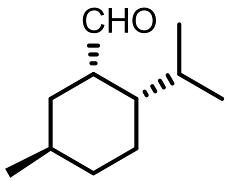

1b-CHO

-crude-

^{1}H NMR(300 MHz, $CDCl_3$)

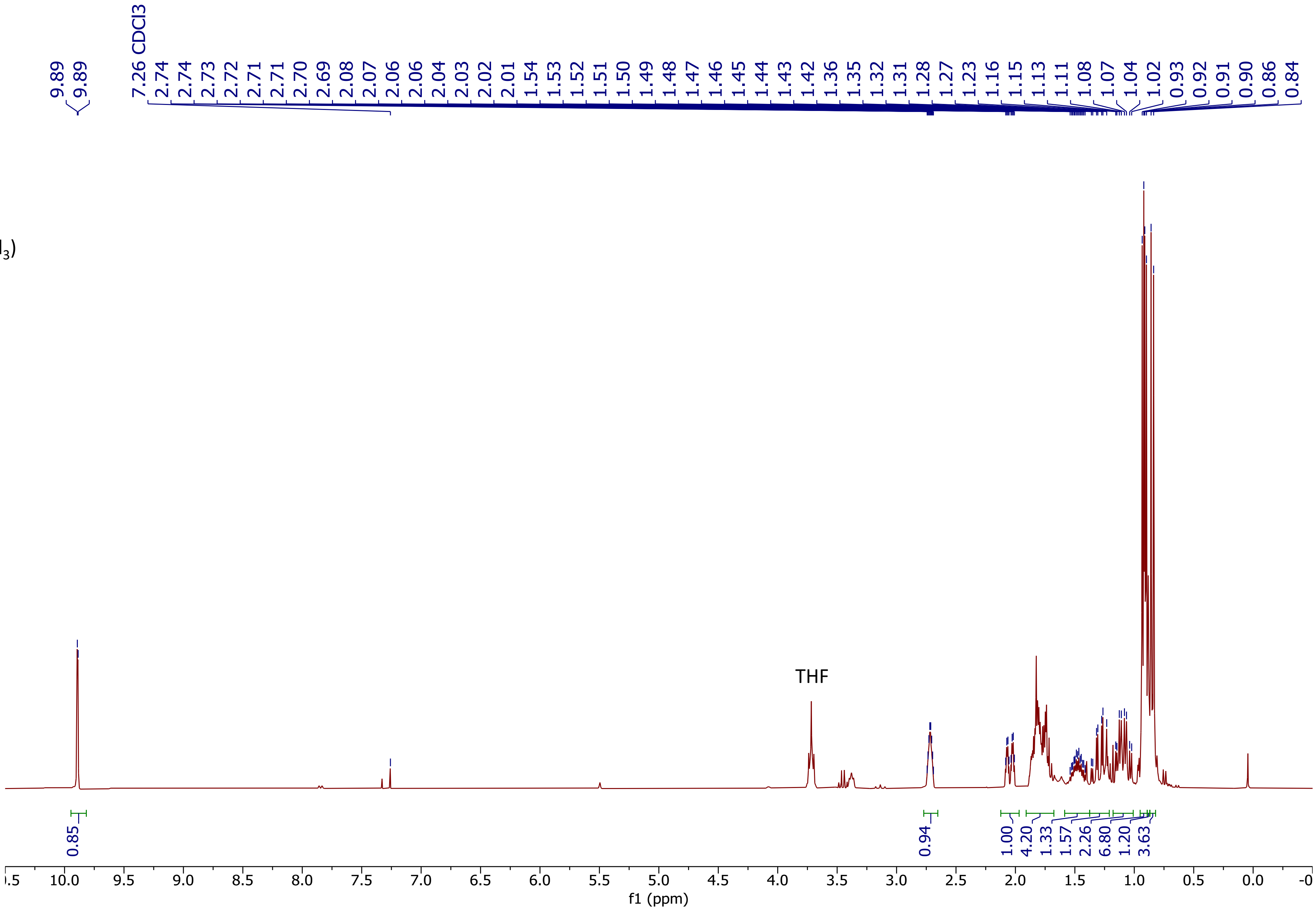

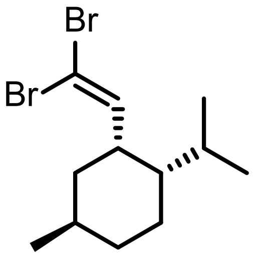

1b-CBr$_2$

^{1}H NMR(300 MHz, $CDCl_3$)

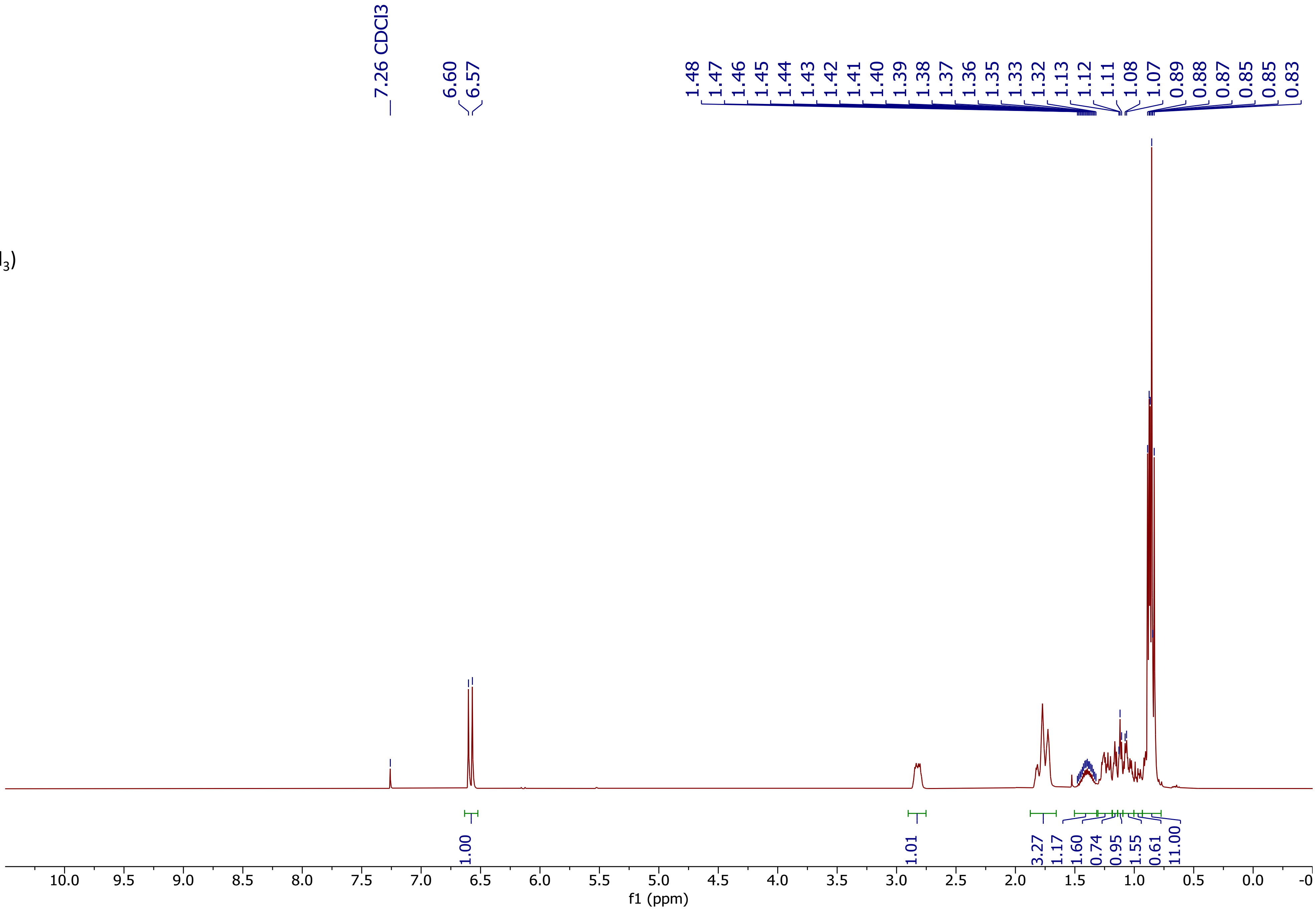

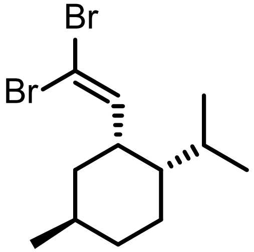

1b-CBr$_2$

^{13}C NMR (75 MHz, $CDCl_3$)

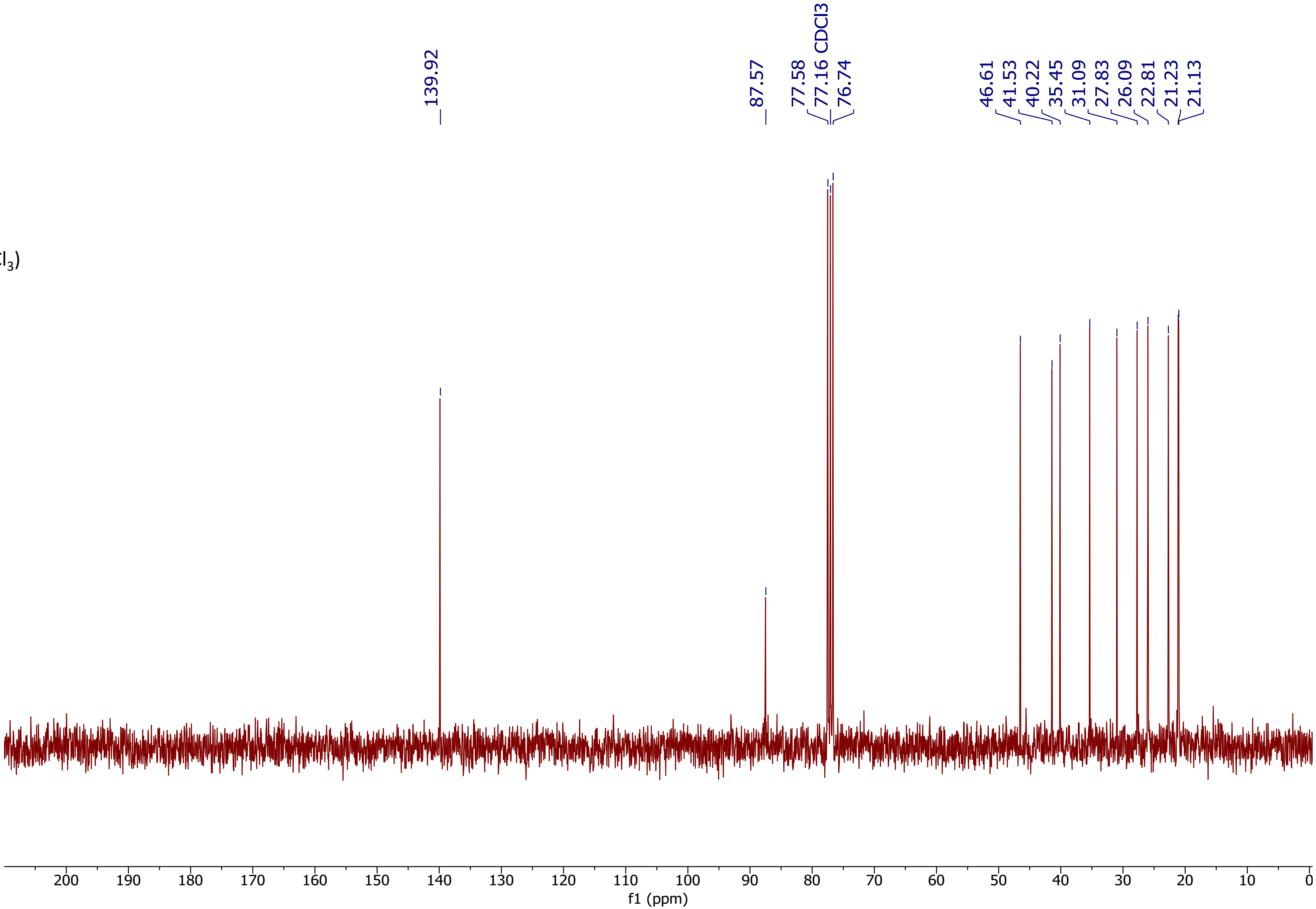

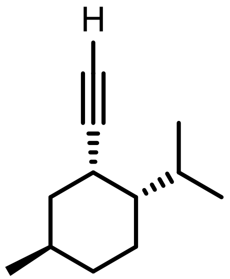

1b-CCH

^{1}H NMR(300 MHz, $CDCl_3$)

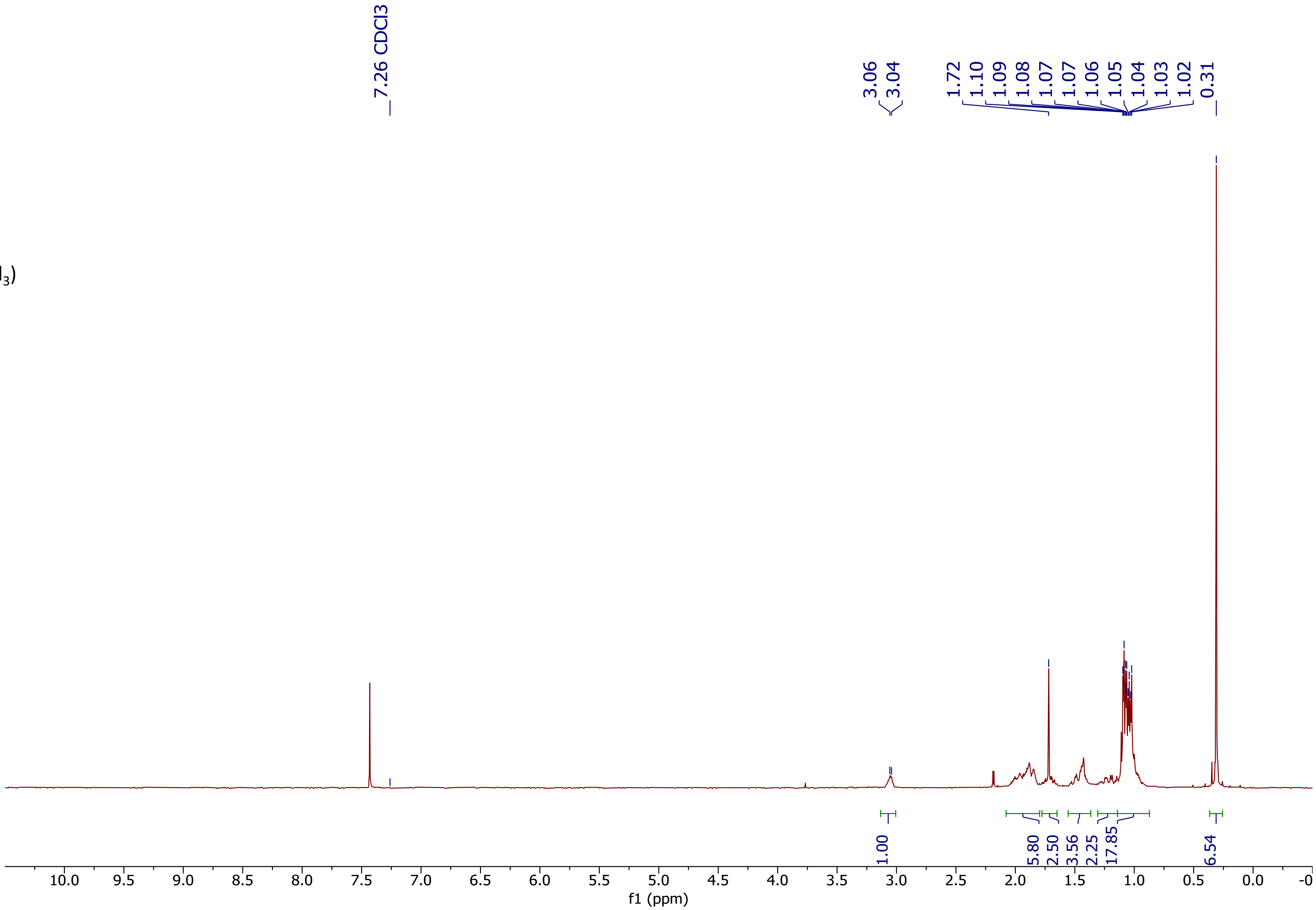

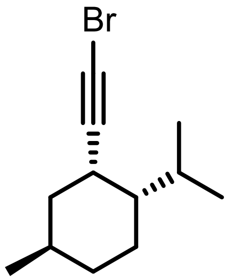

1b

^{1}H NMR(300 MHz, $CDCl_3$)

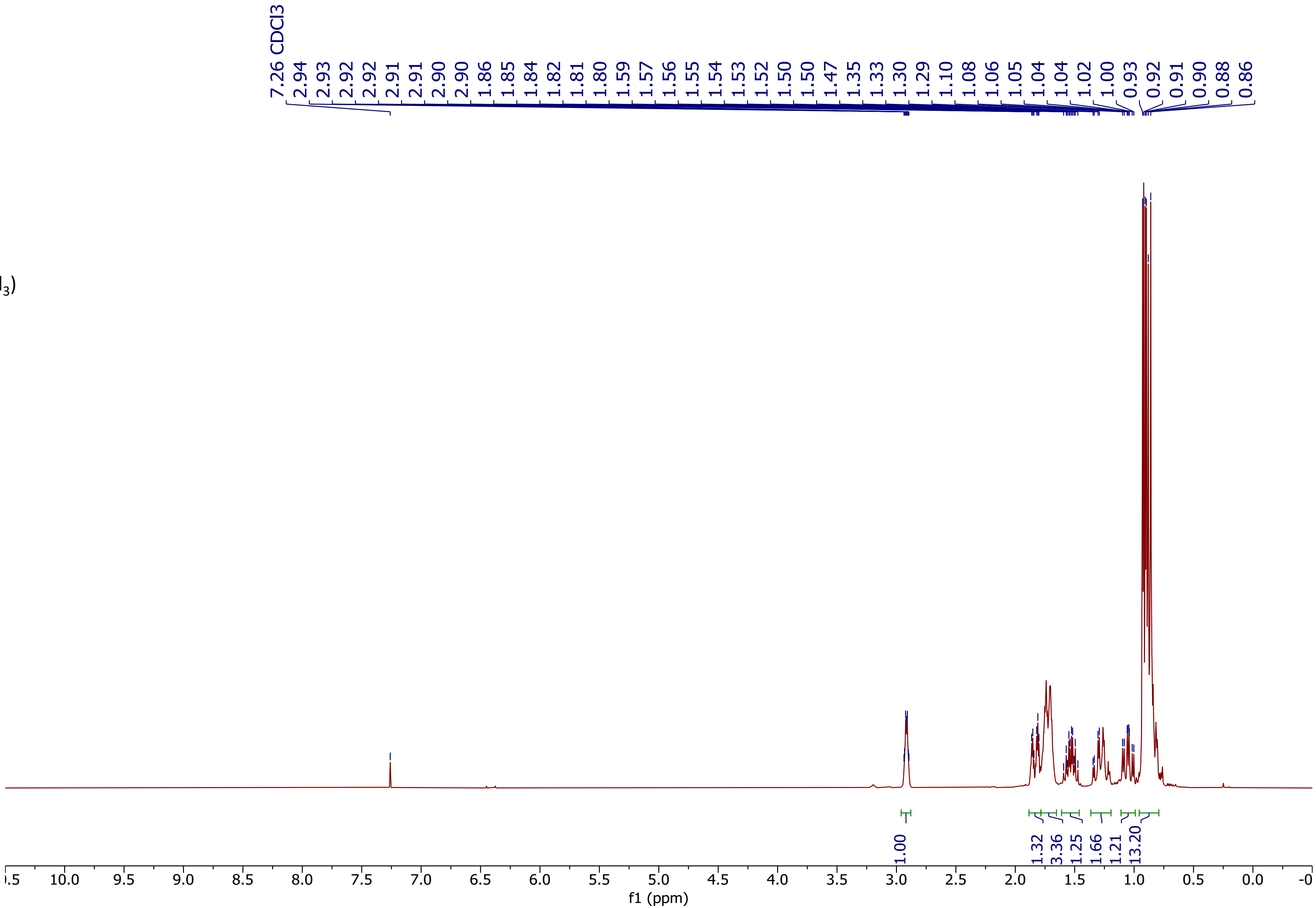

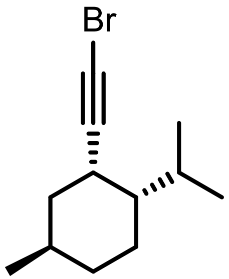

1b

^{13}**C NMR** (75 MHz, $CDCl_3$)

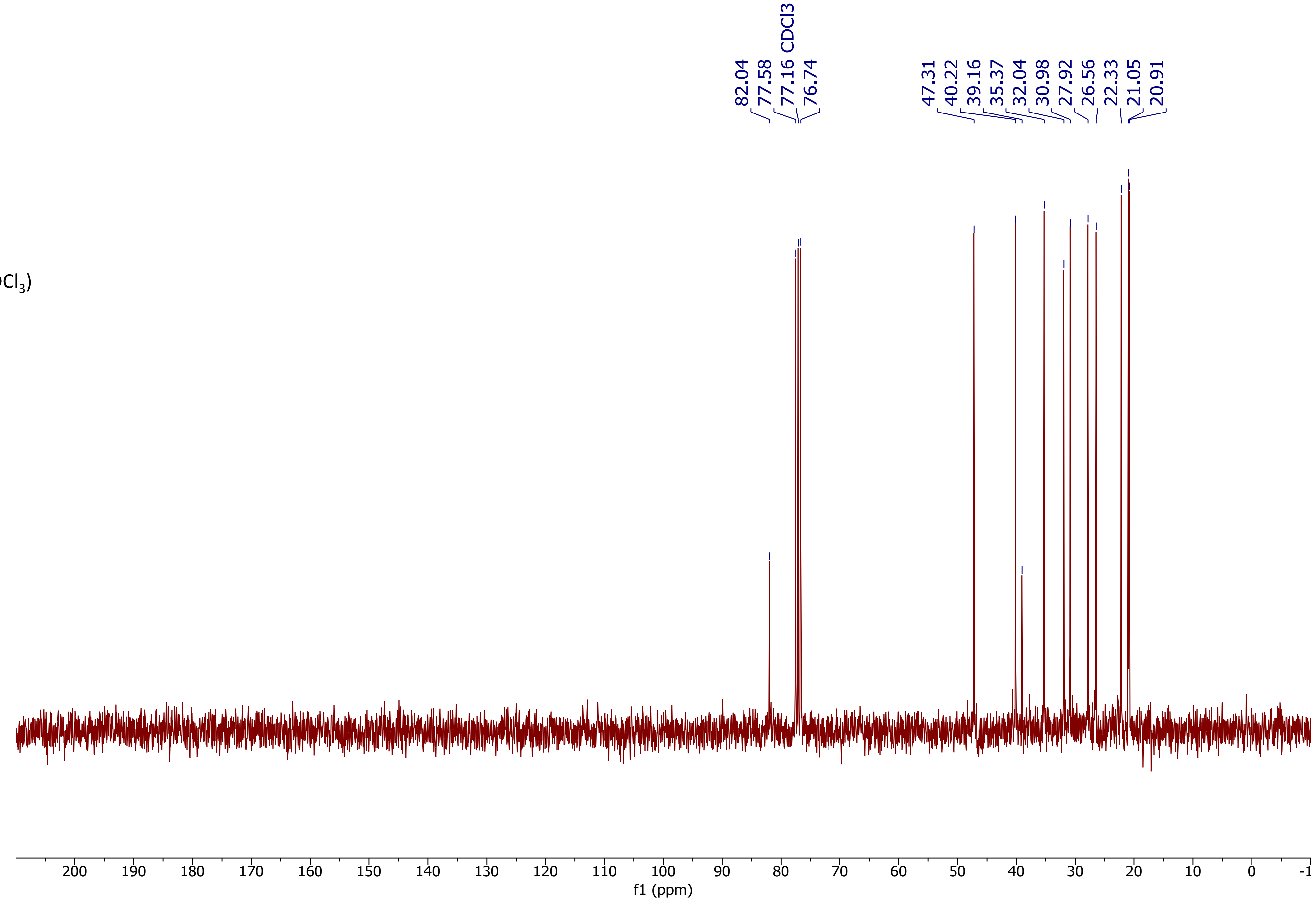

1c,d

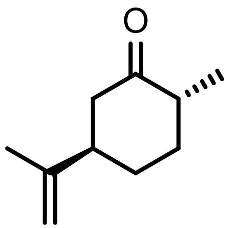

1c-C=C

^{1}H NMR(300 MHz, $CDCl_3$)

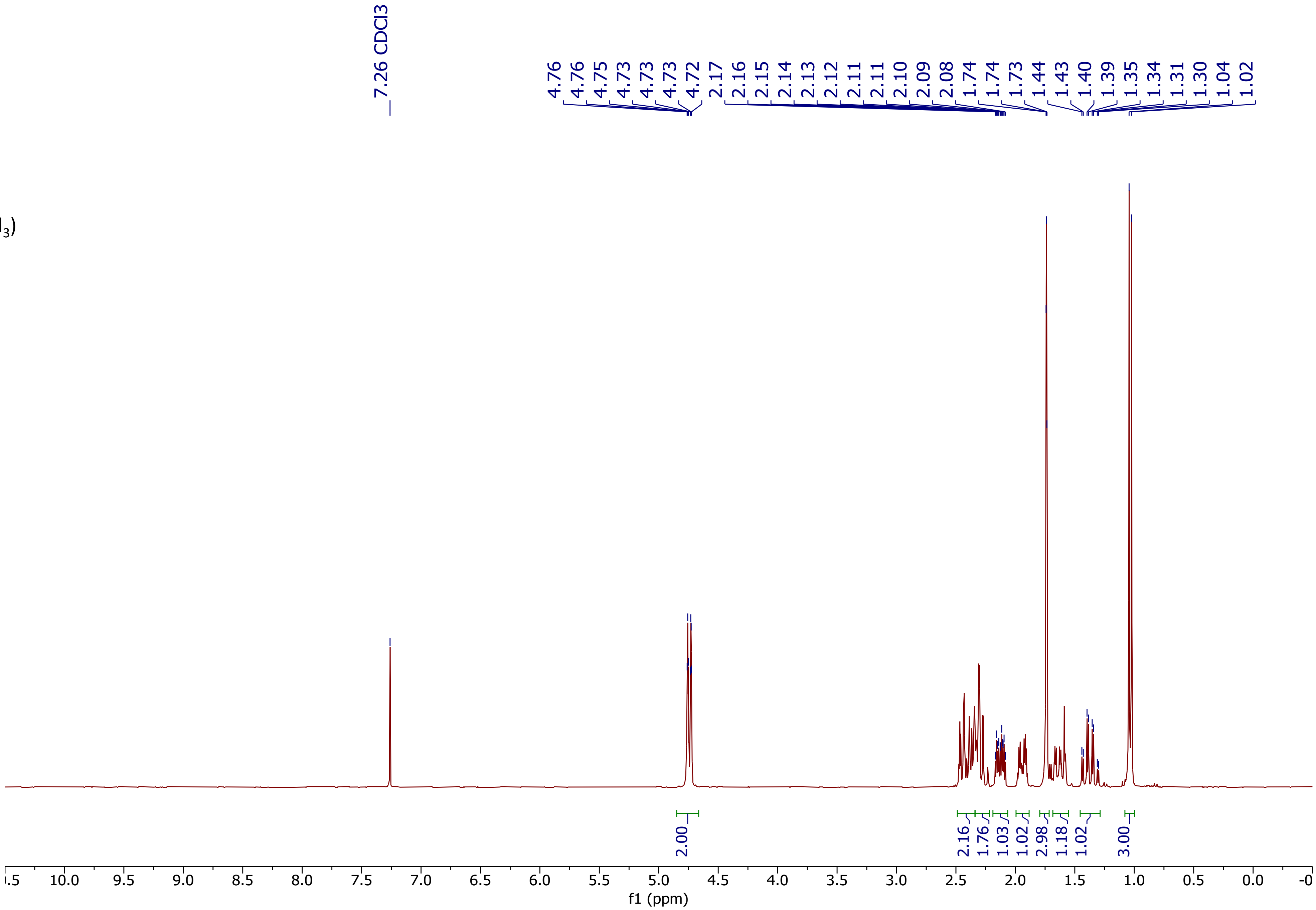

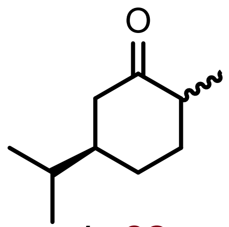

1c-CO

¹H NMR(300 MHz, $CDCl_3$)

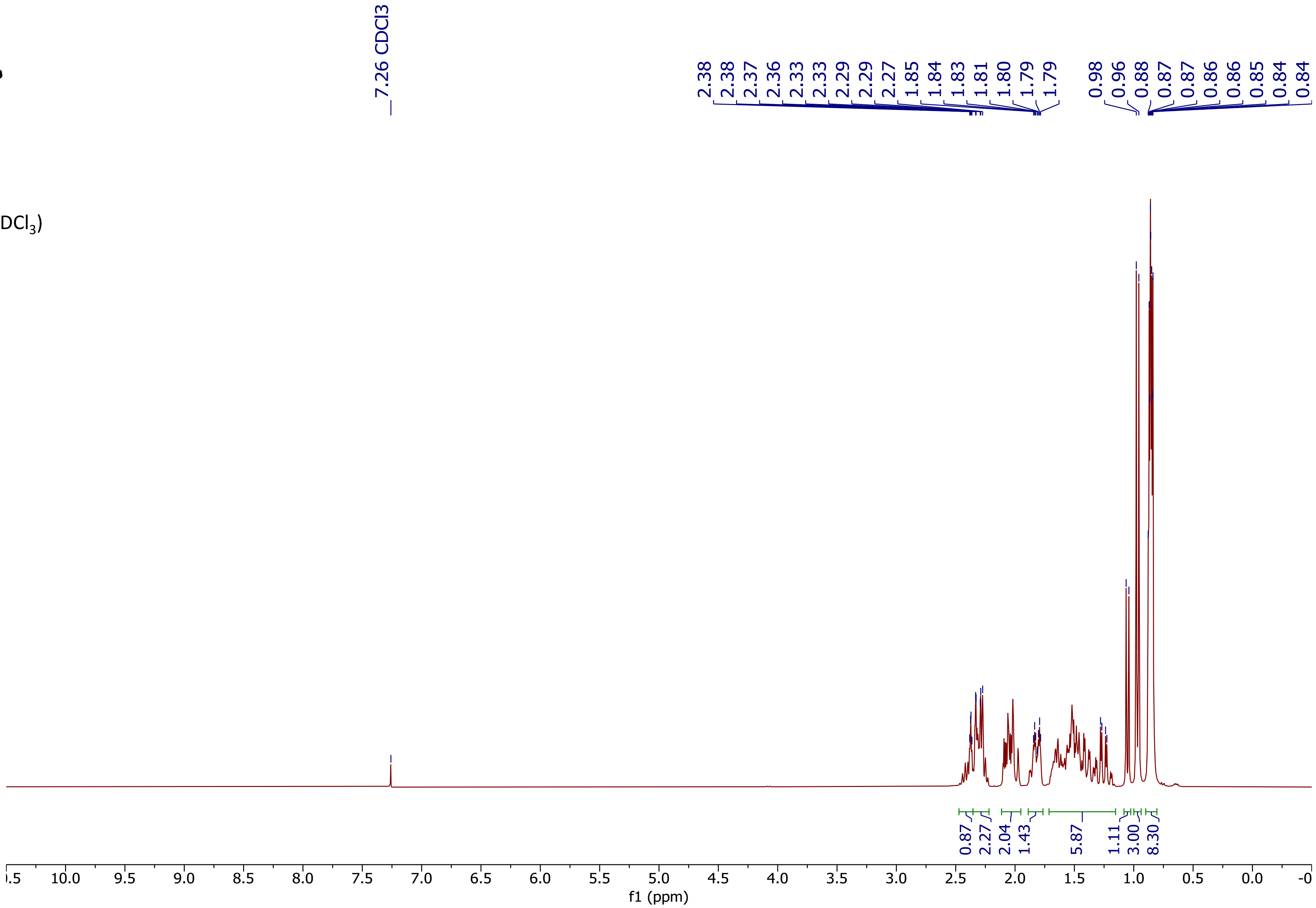

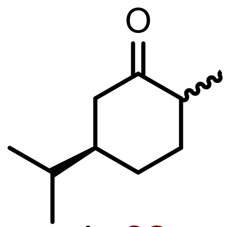

1c-CO

^{13}**C NMR** (75 MHz, $CDCl_3$)

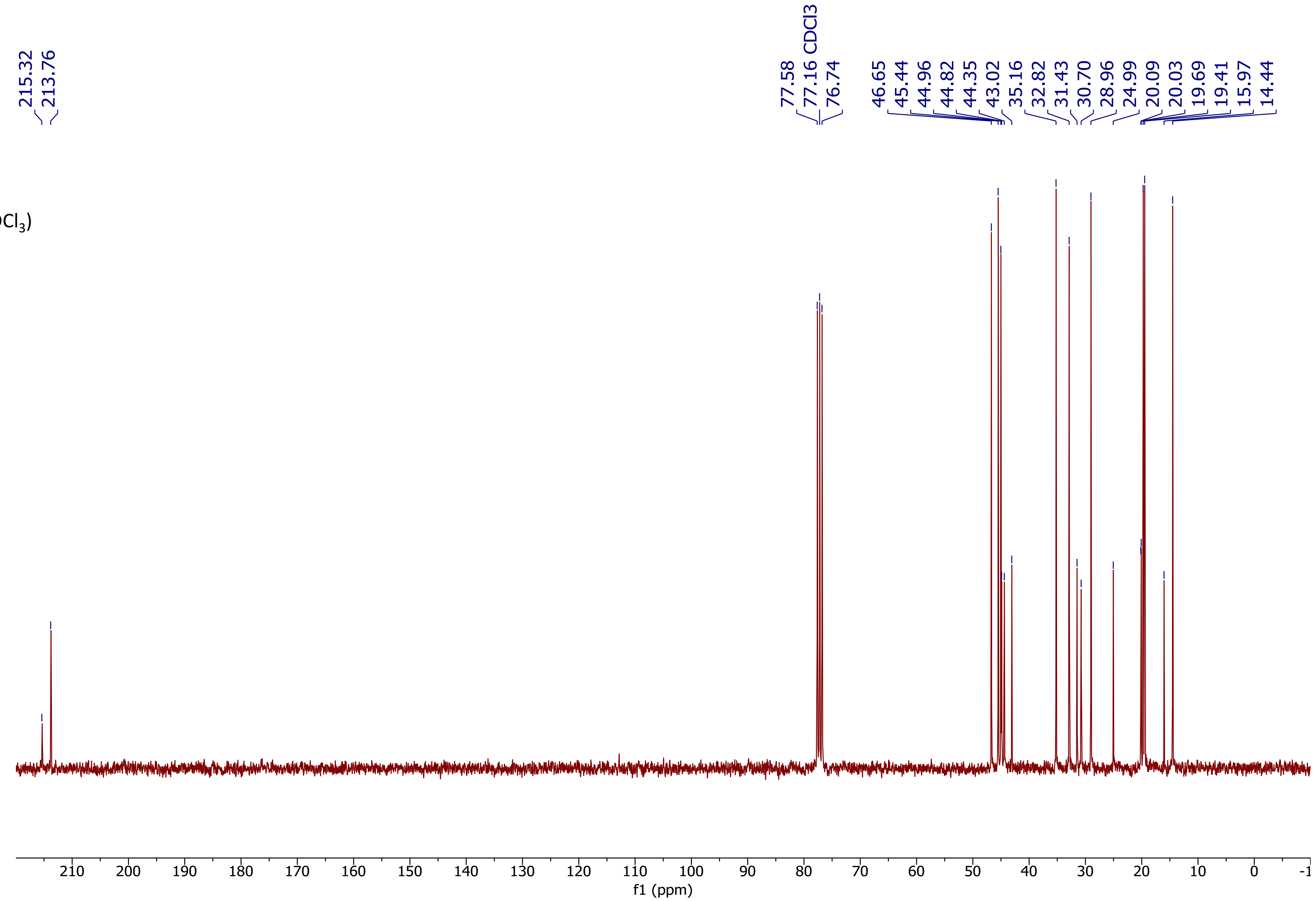

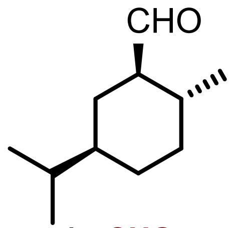

1c-CHO

^{1}H NMR(300 MHz, $CDCl_3$)

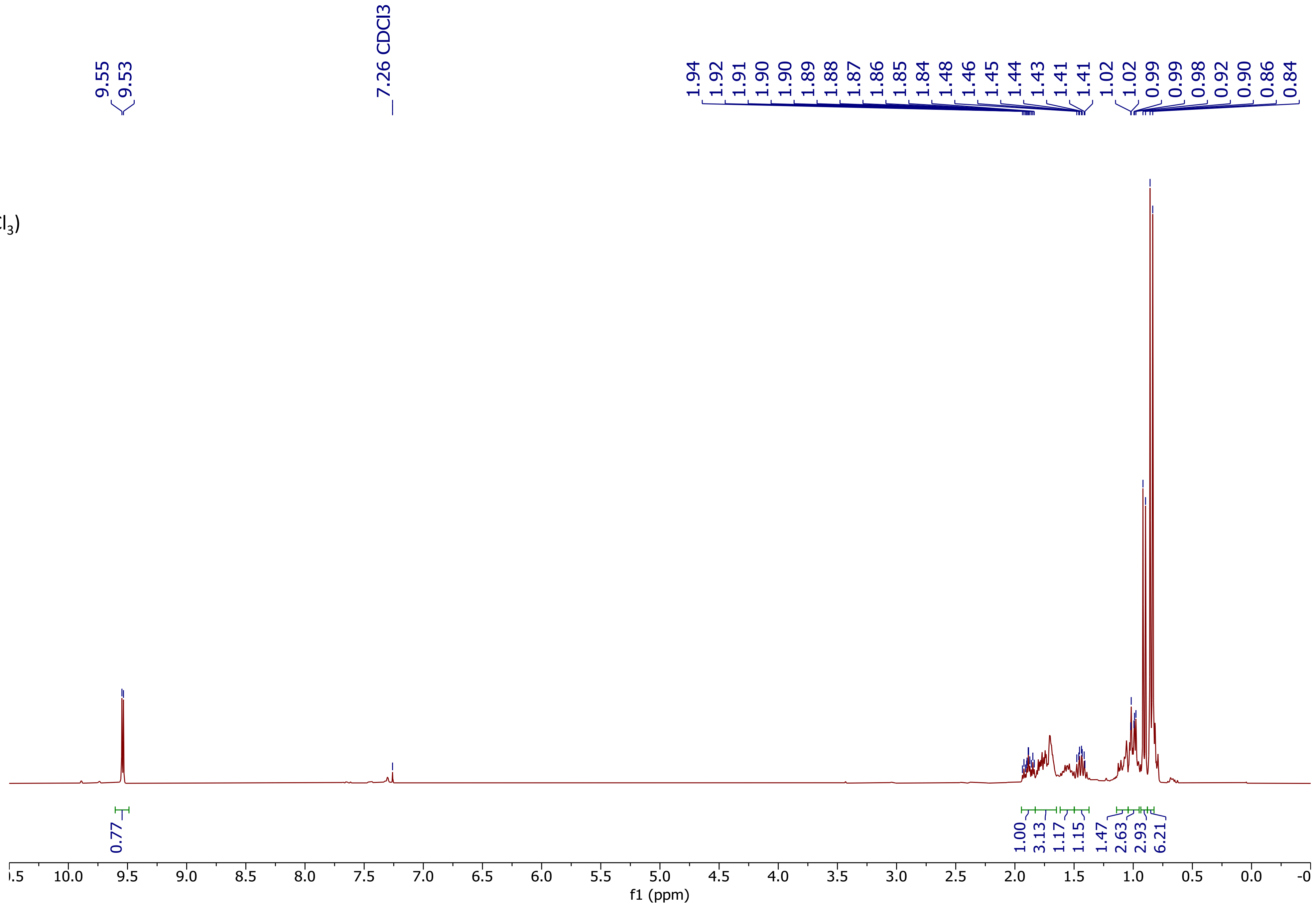

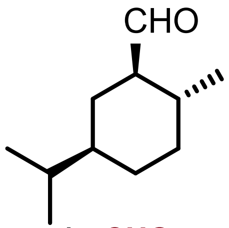

1c-CHO

^{13}C NMR (75 MHz, $CDCl_3$)

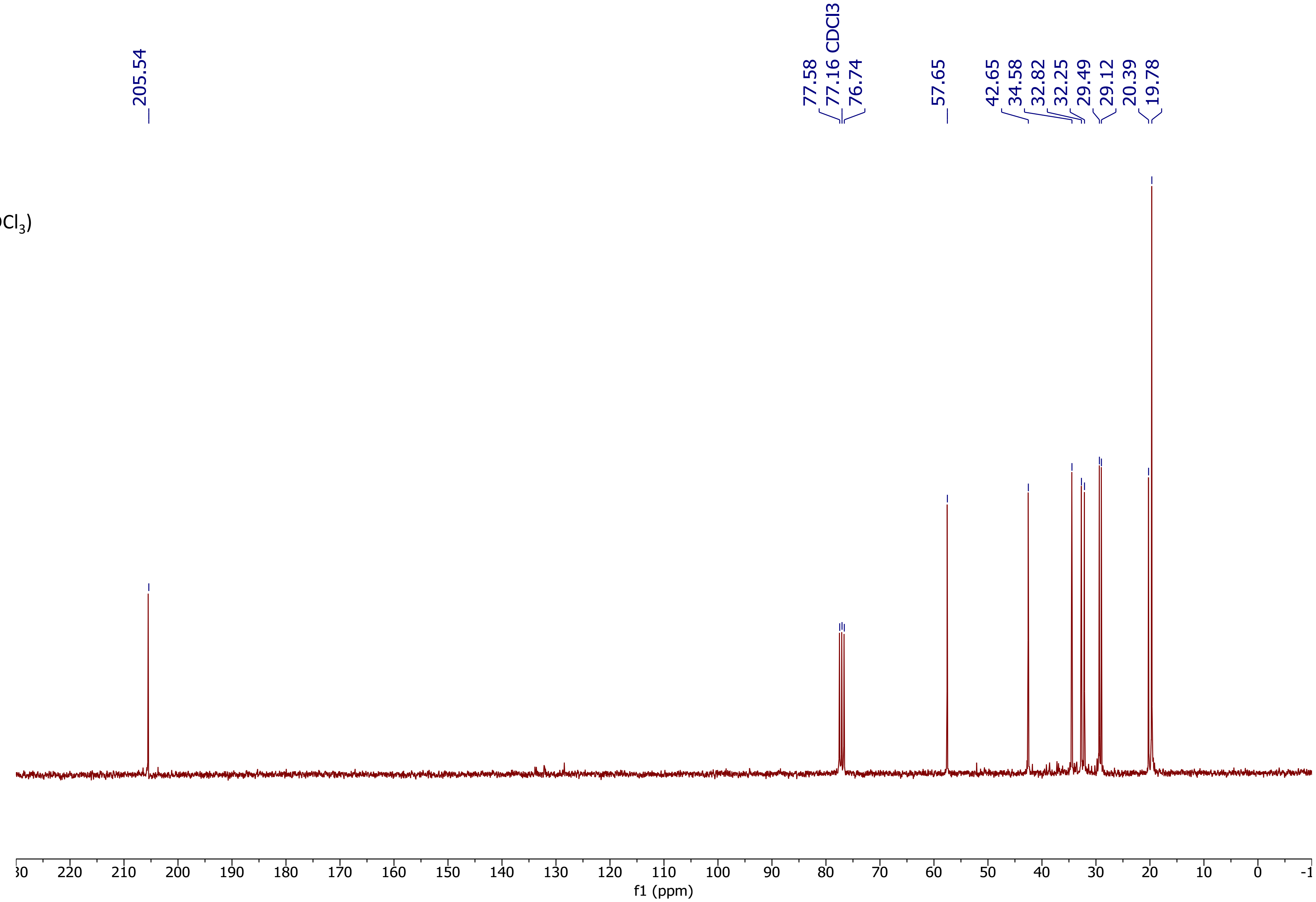

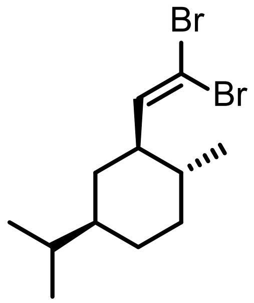

1c-CBr$_2$

^{1}H NMR(300 MHz, $CDCl_3$)

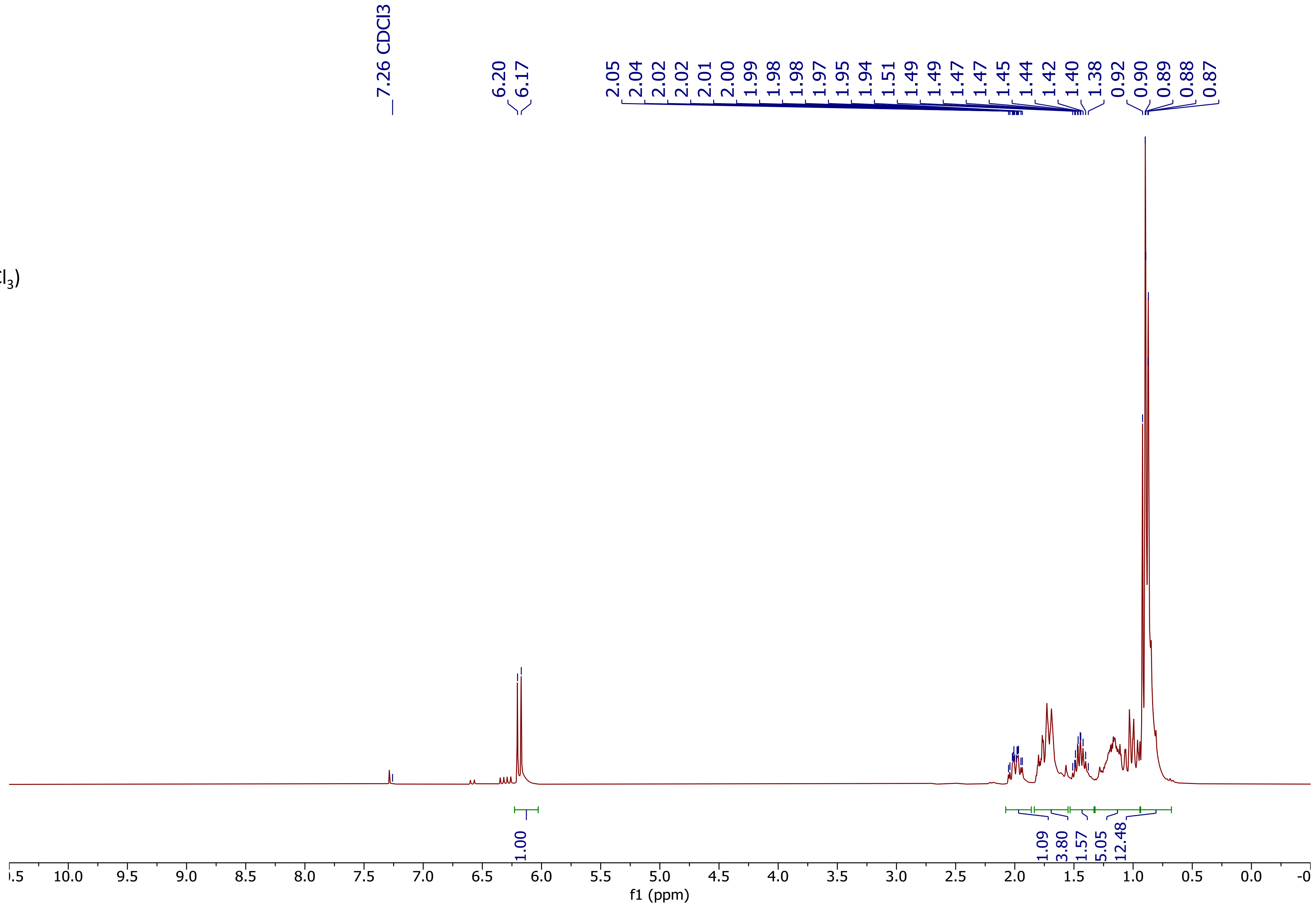

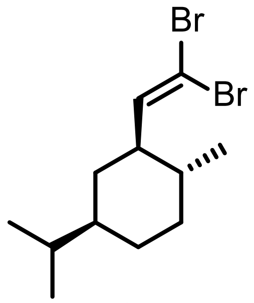

1c-CBr₂

^{13}**C NMR** (75 MHz, $CDCl_3$)

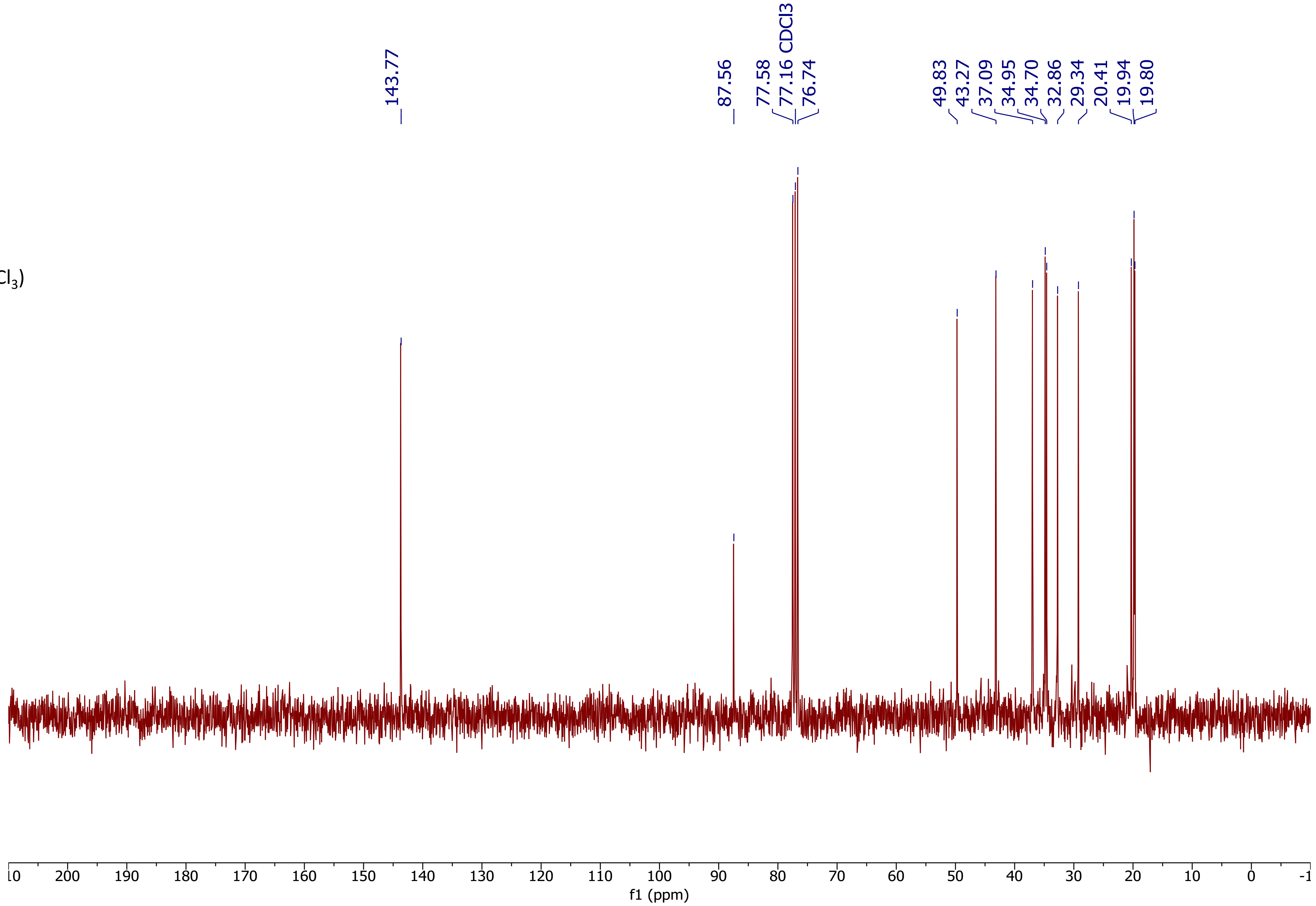

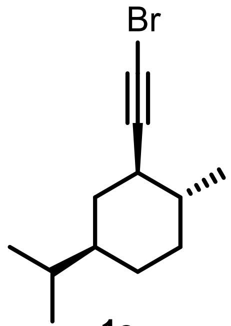

1c

¹H NMR(300 MHz, $CDCl_3$)

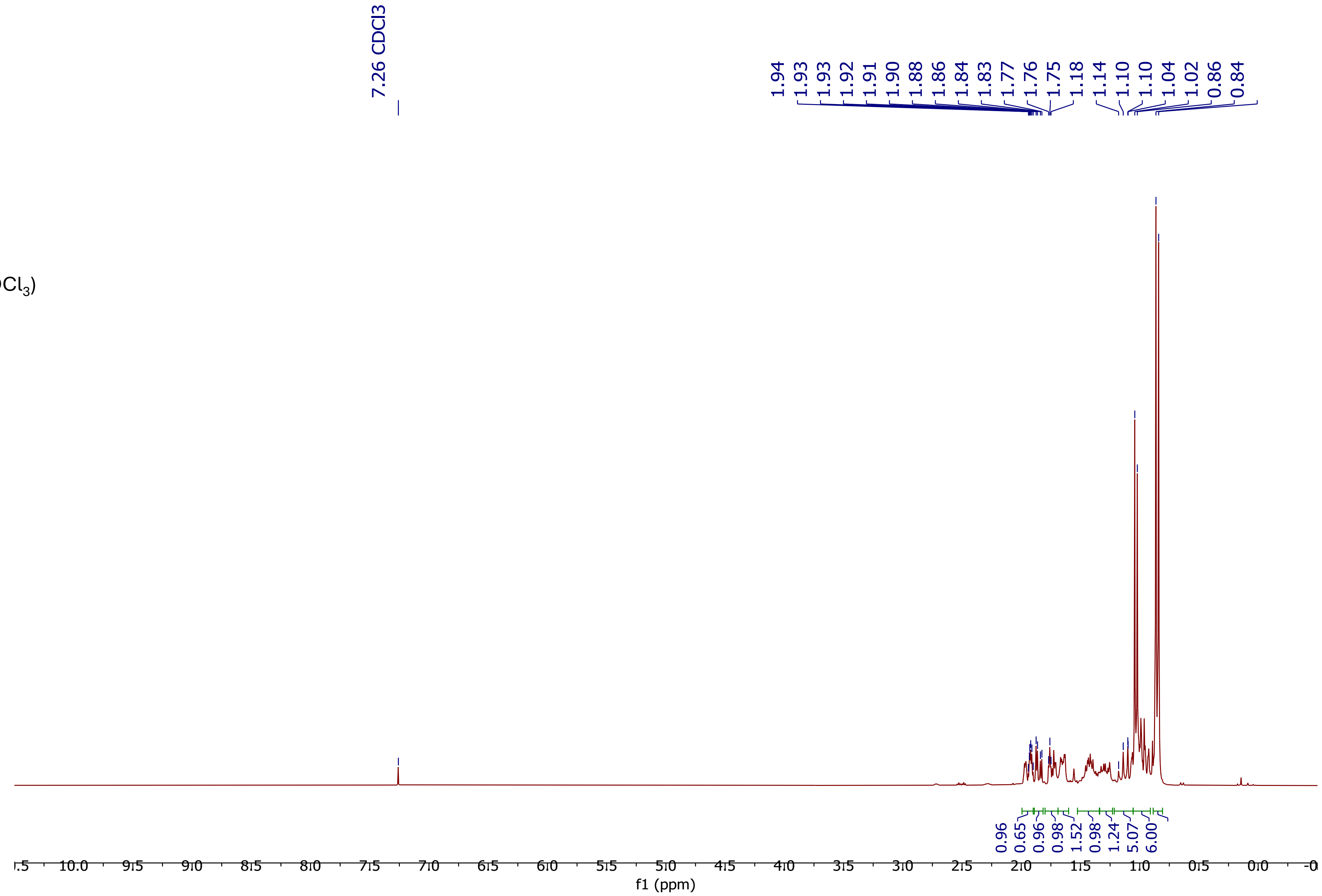

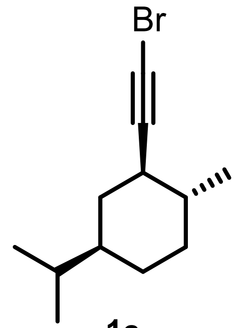

1c

^{13}**C NMR** (75 MHz, $CDCl_3$)

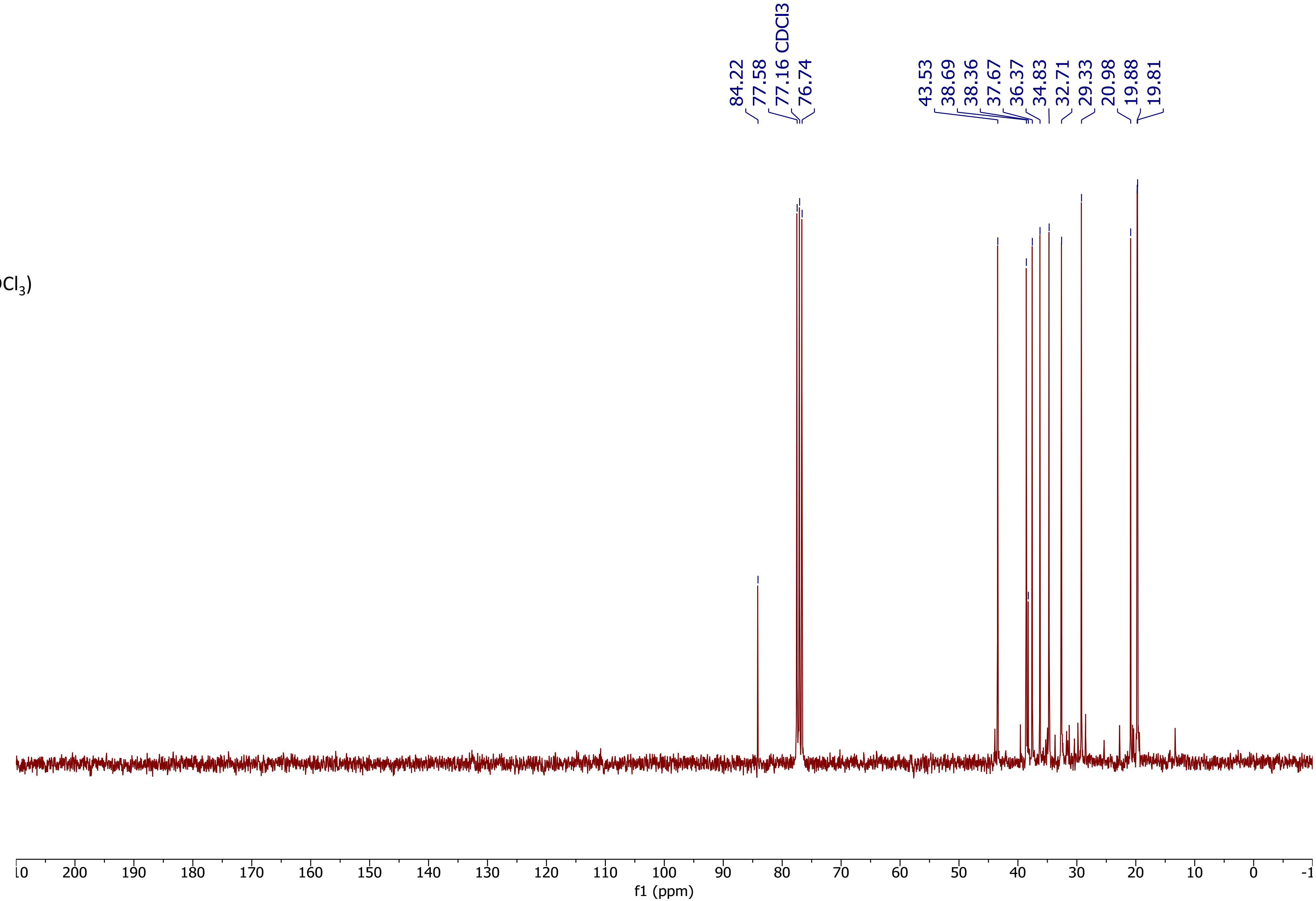

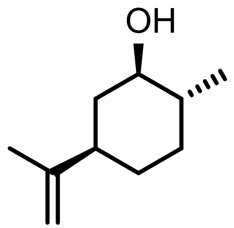

1d-C=C

¹H NMR(300 MHz, $CDCl_3$)

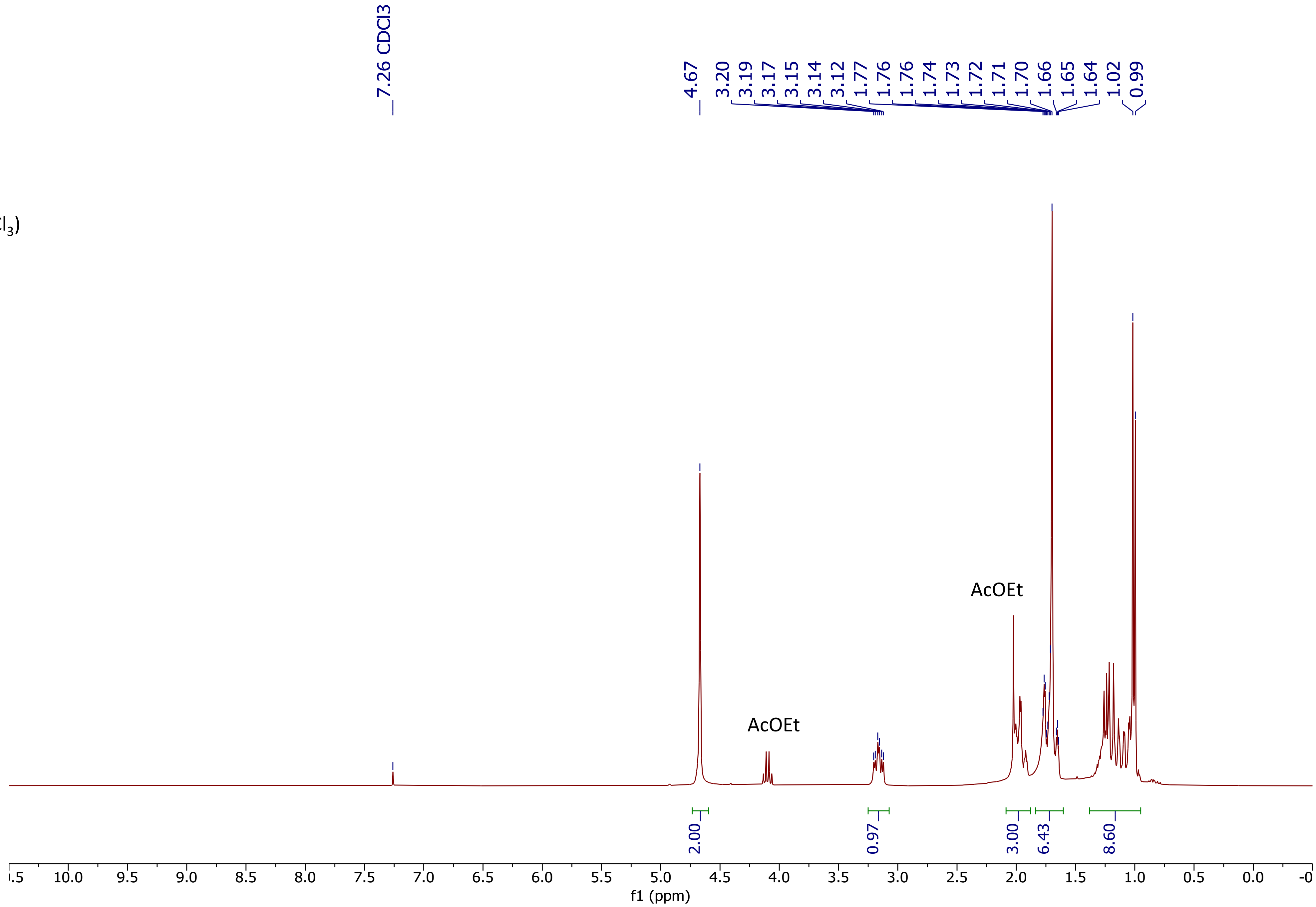

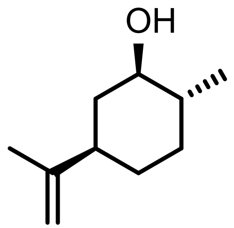

1d-C=C

^{13}**C NMR** (75 MHz, $CDCl_3$)

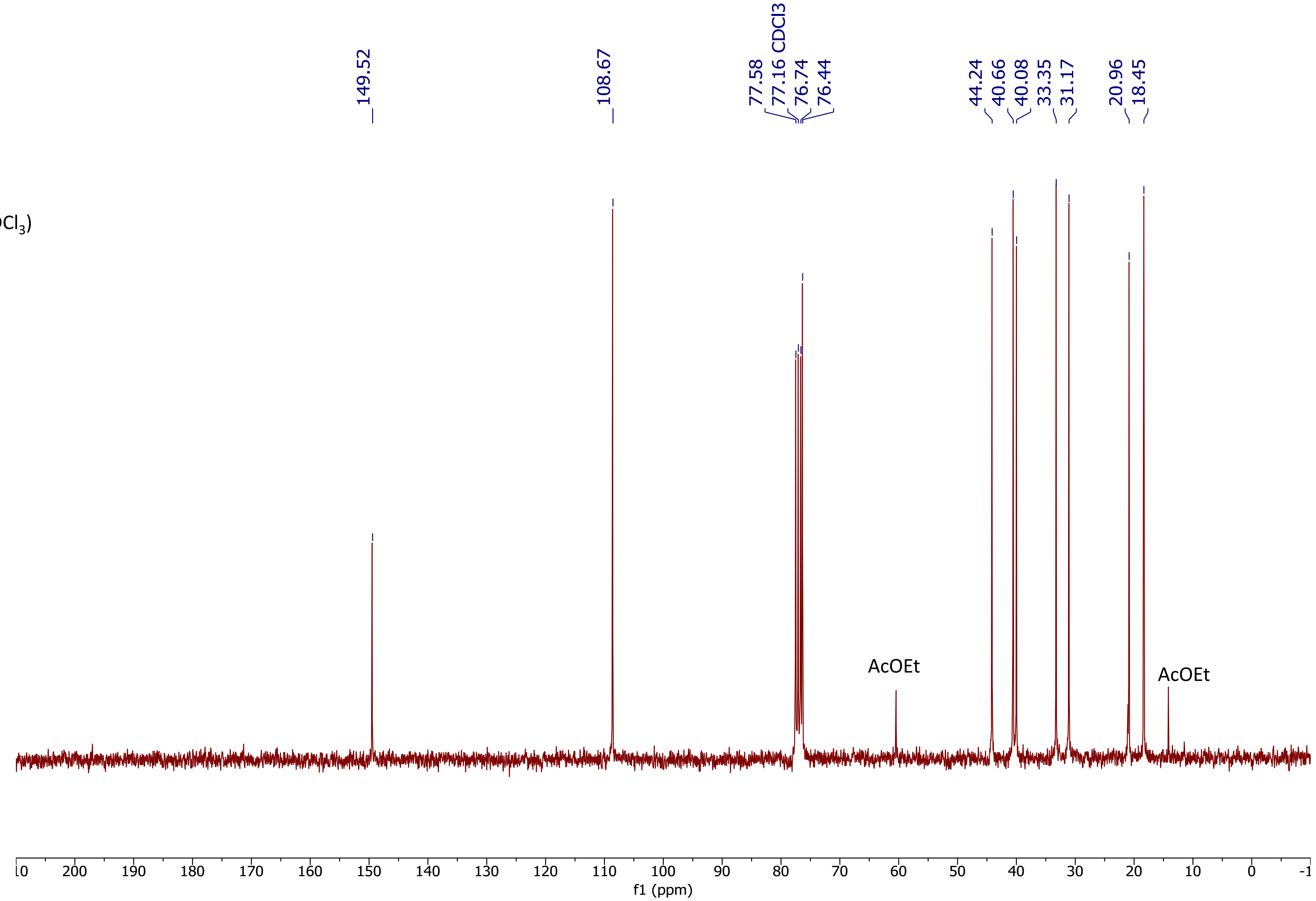

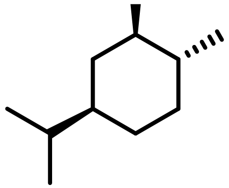

1d-OH

-crude-

^{1}H NMR(300 MHz, $CDCl_3$)

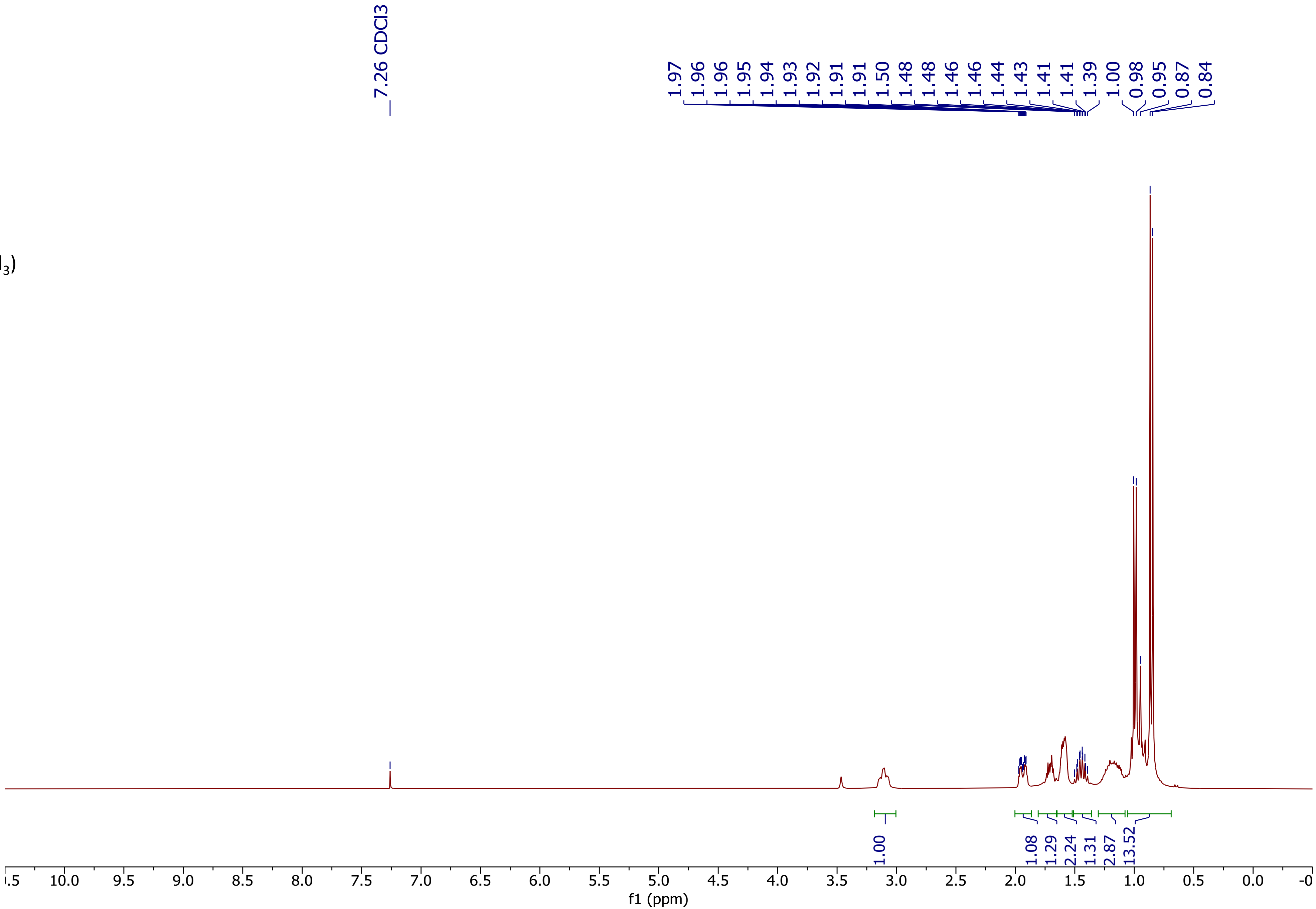

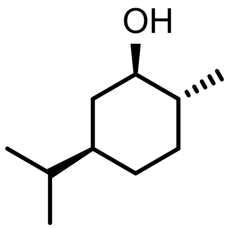

1d-OH

-crude-

^{13}C NMR (75 MHz, $CDCl_3$)

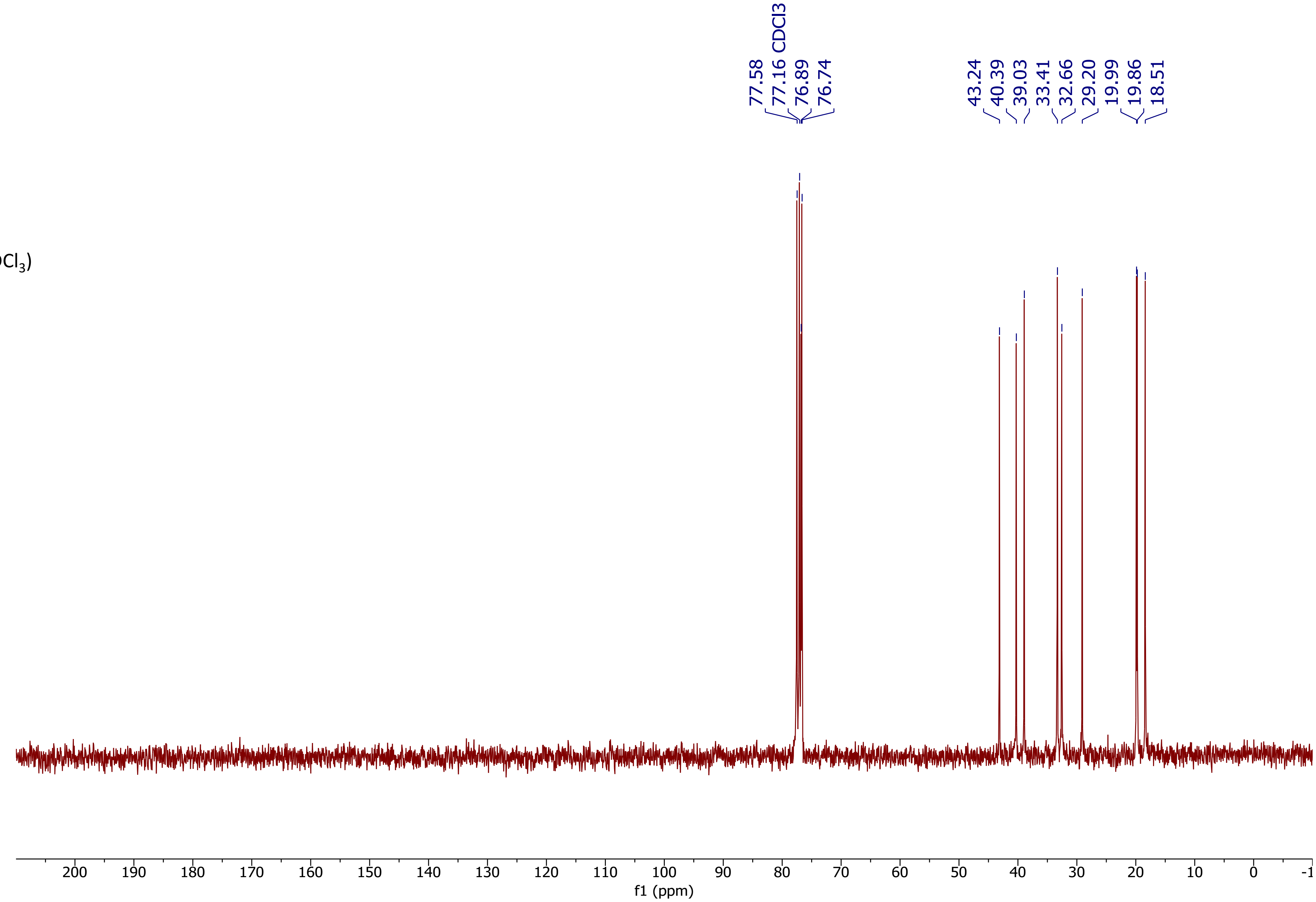

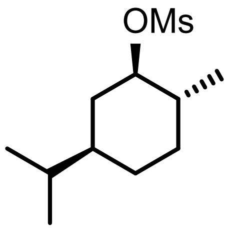

1d-OMs

-crude-

^{1}H NMR(300 MHz, $CDCl_3$)

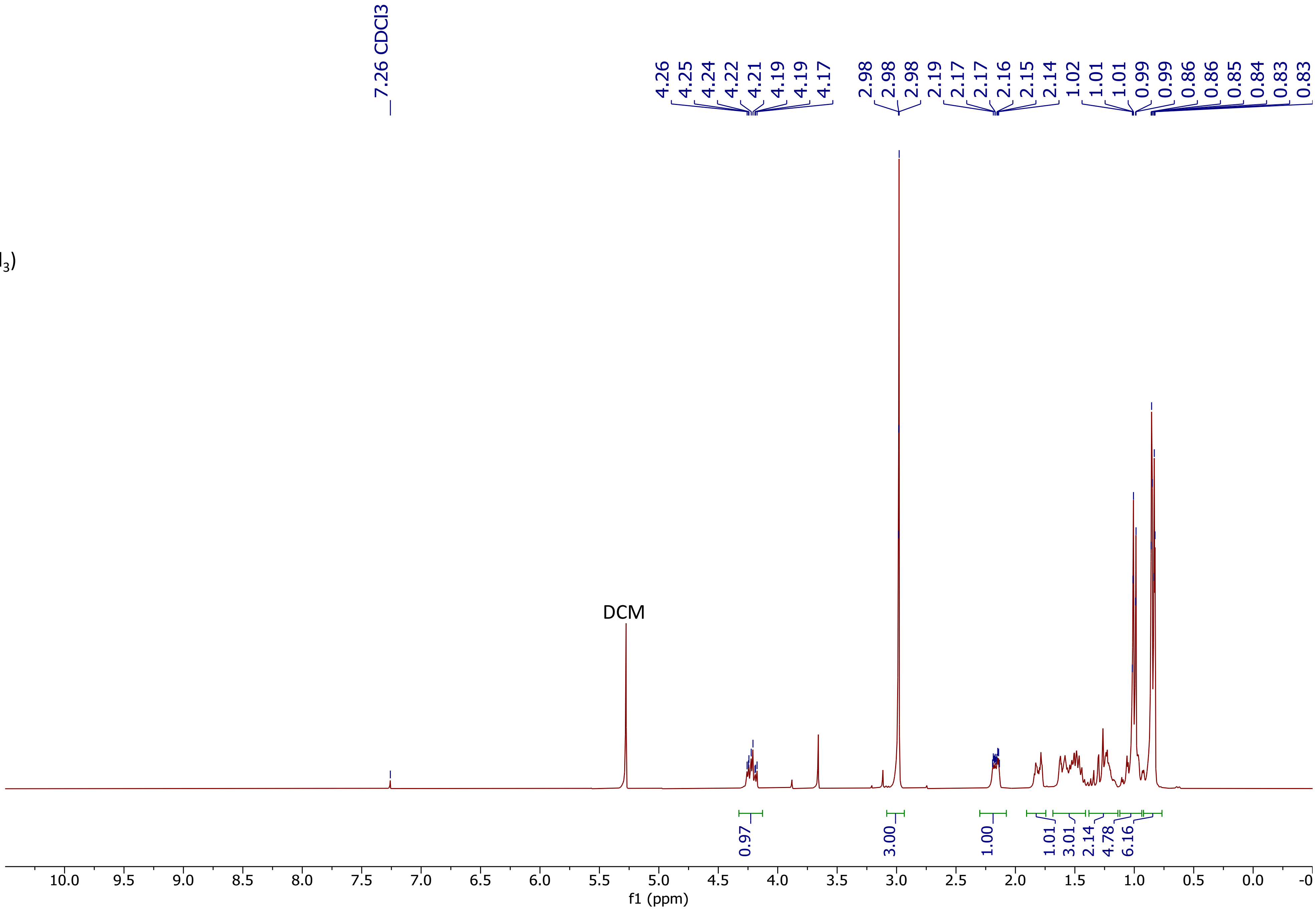

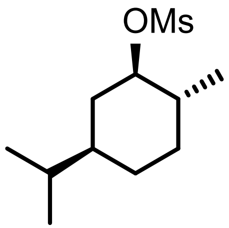

1d-OMs

-crude-

^{13}C NMR (75 MHz, $CDCl_3$)

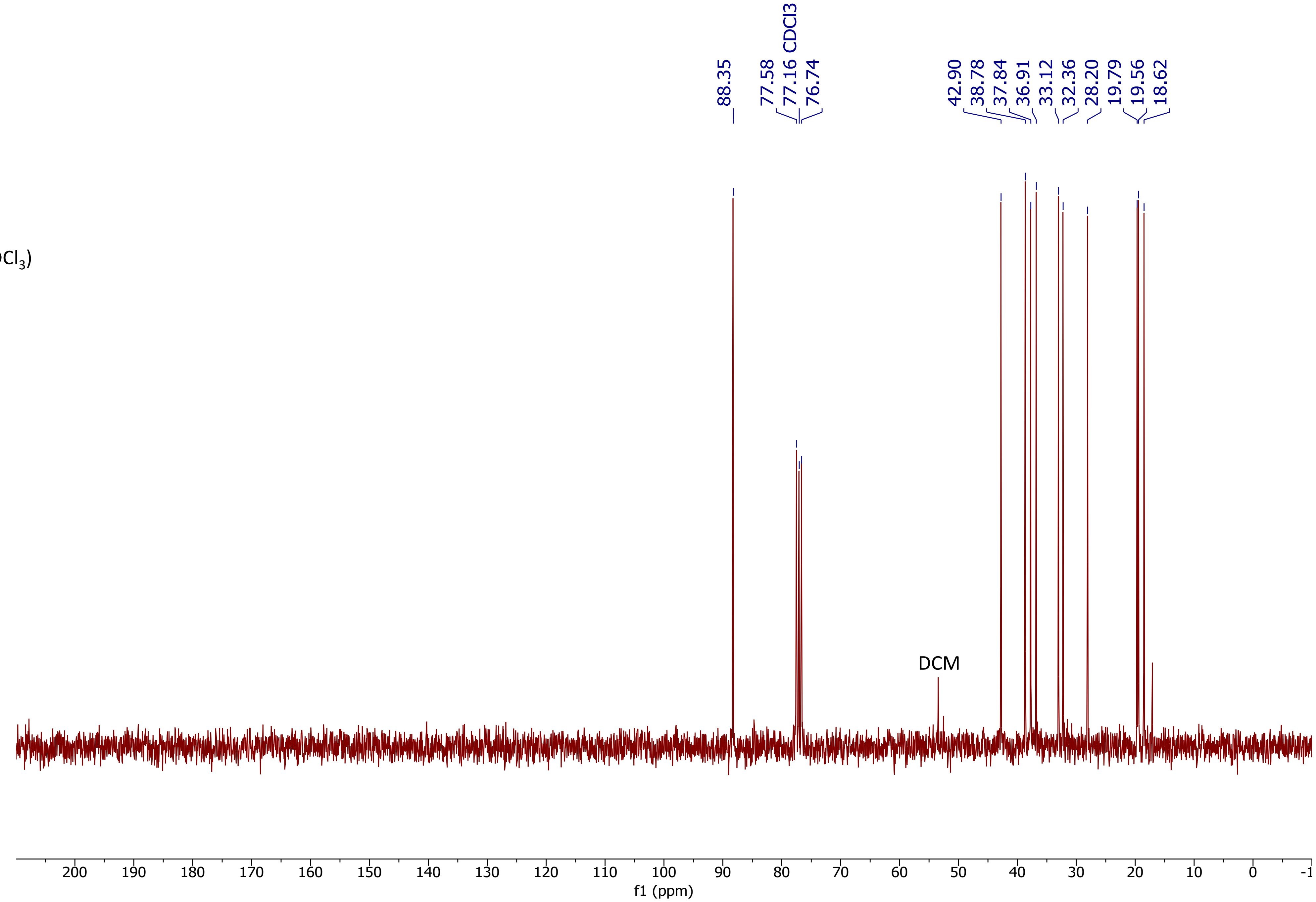

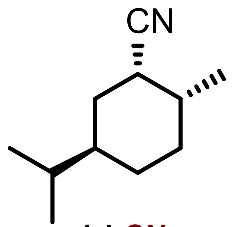

1d-CN

^{1}H NMR(300 MHz, $CDCl_3$)

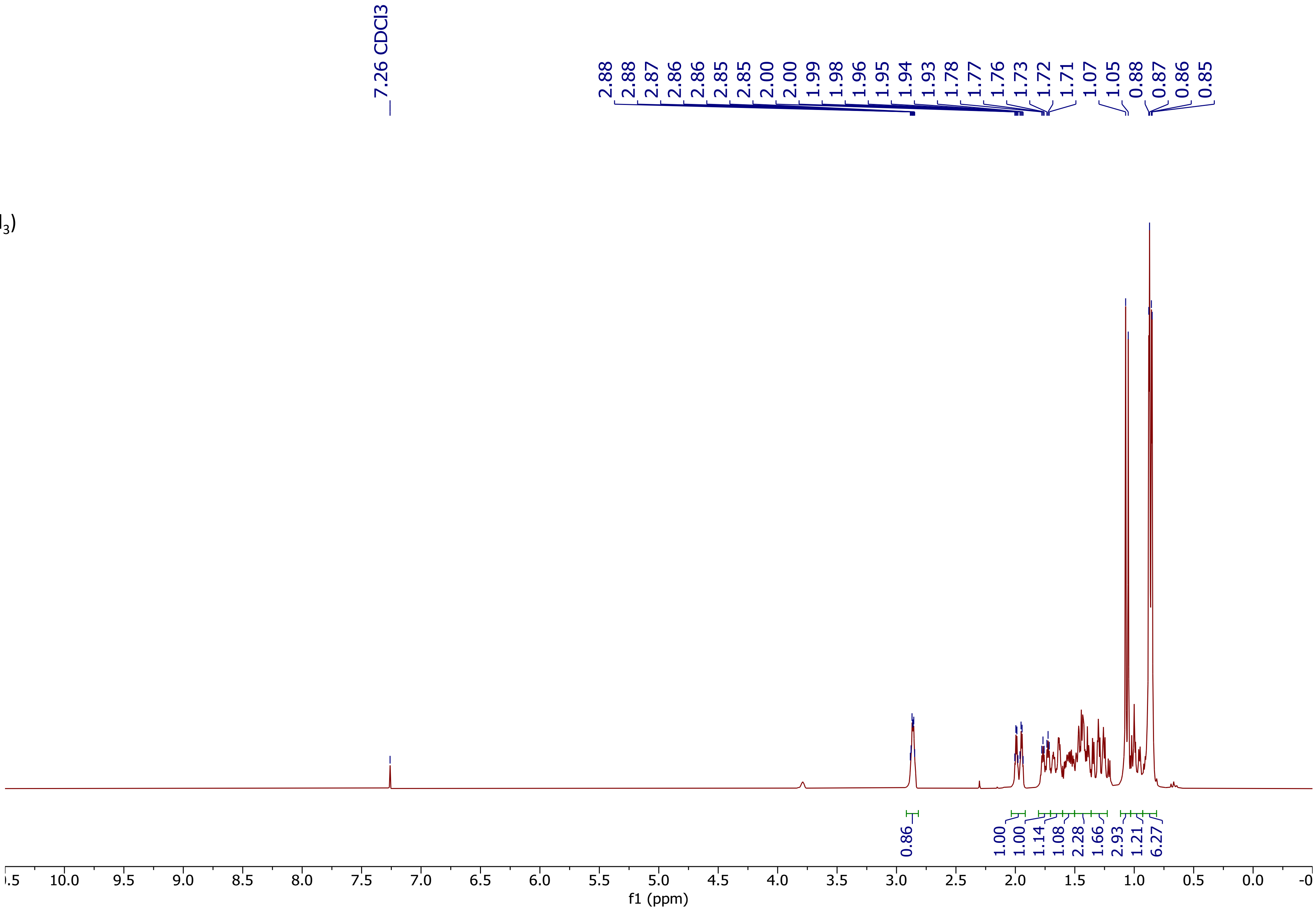

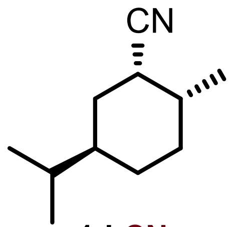

1d-CN

^{13}C NMR (75 MHz, $CDCl_3$)

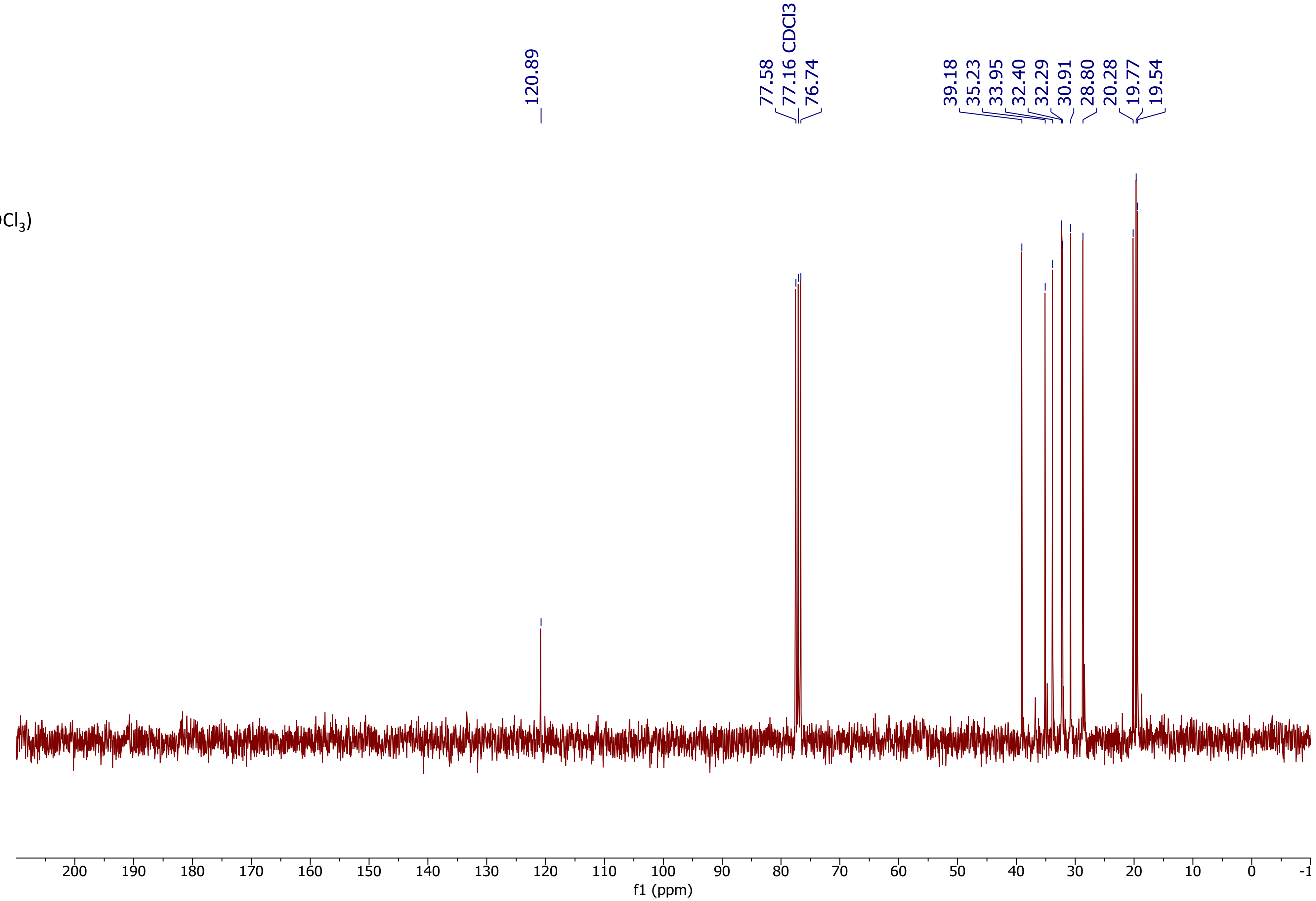

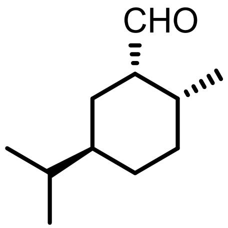

1d-CHO

-crude-

^{1}H NMR(300 MHz, $CDCl_3$)

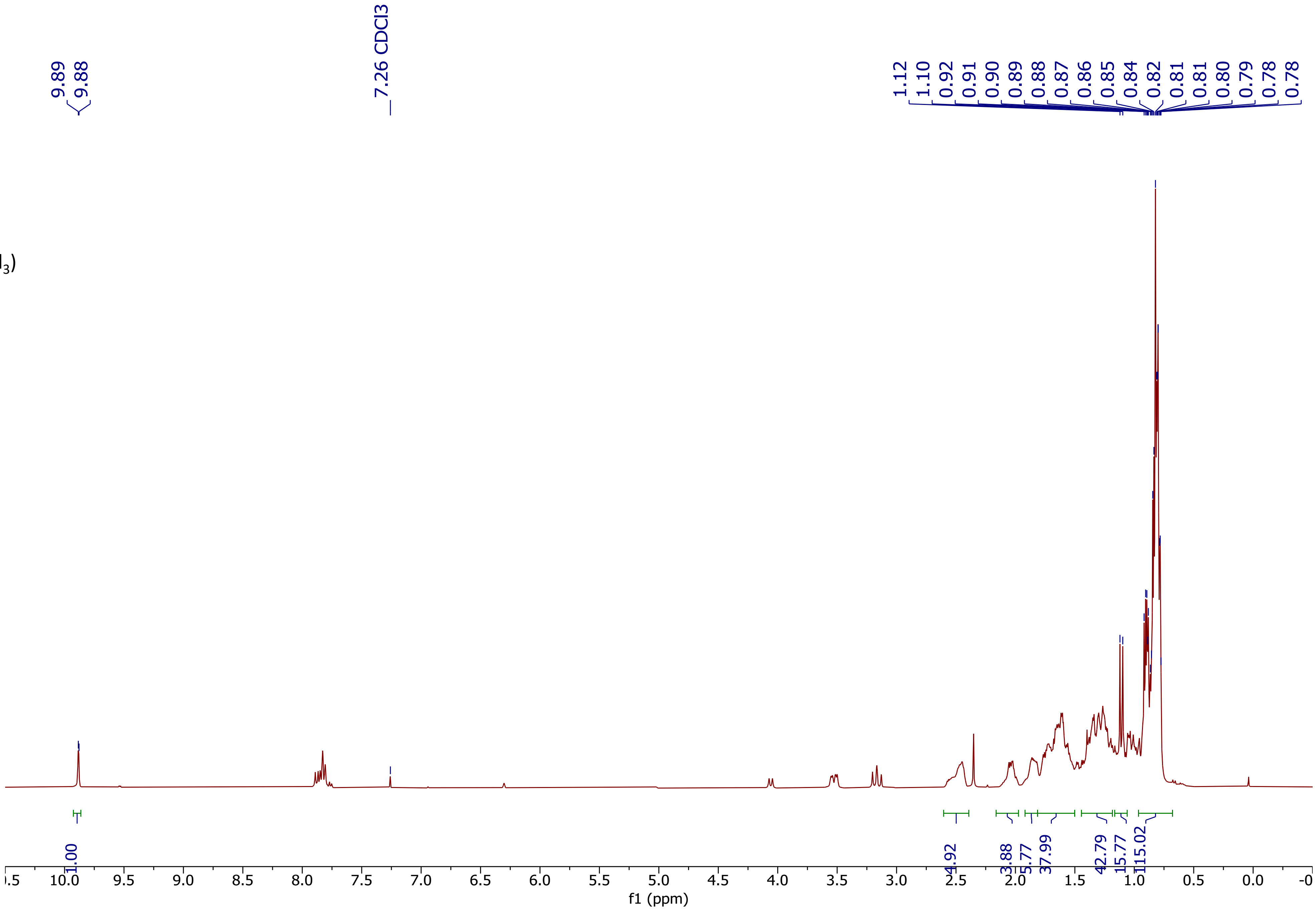

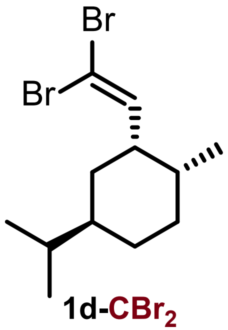

1d-CBr₂

^{1}**H NMR**(300 MHz, $CDCl_3$)

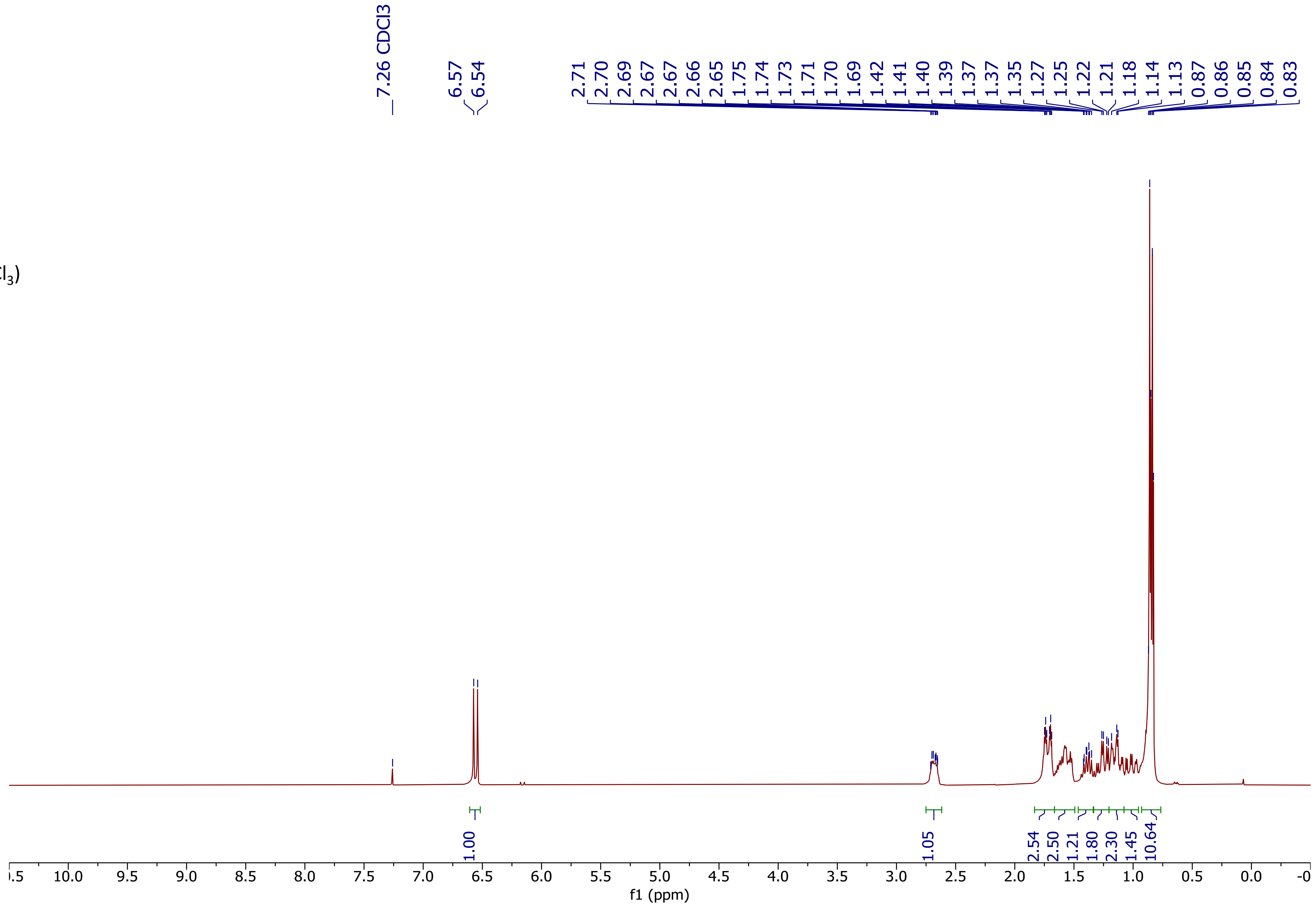

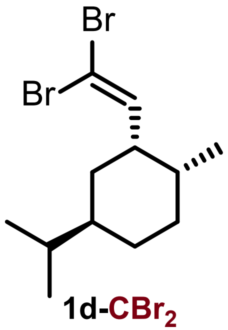

1d-CBr_2

^{13}C NMR (75 MHz, $CDCl_3$)

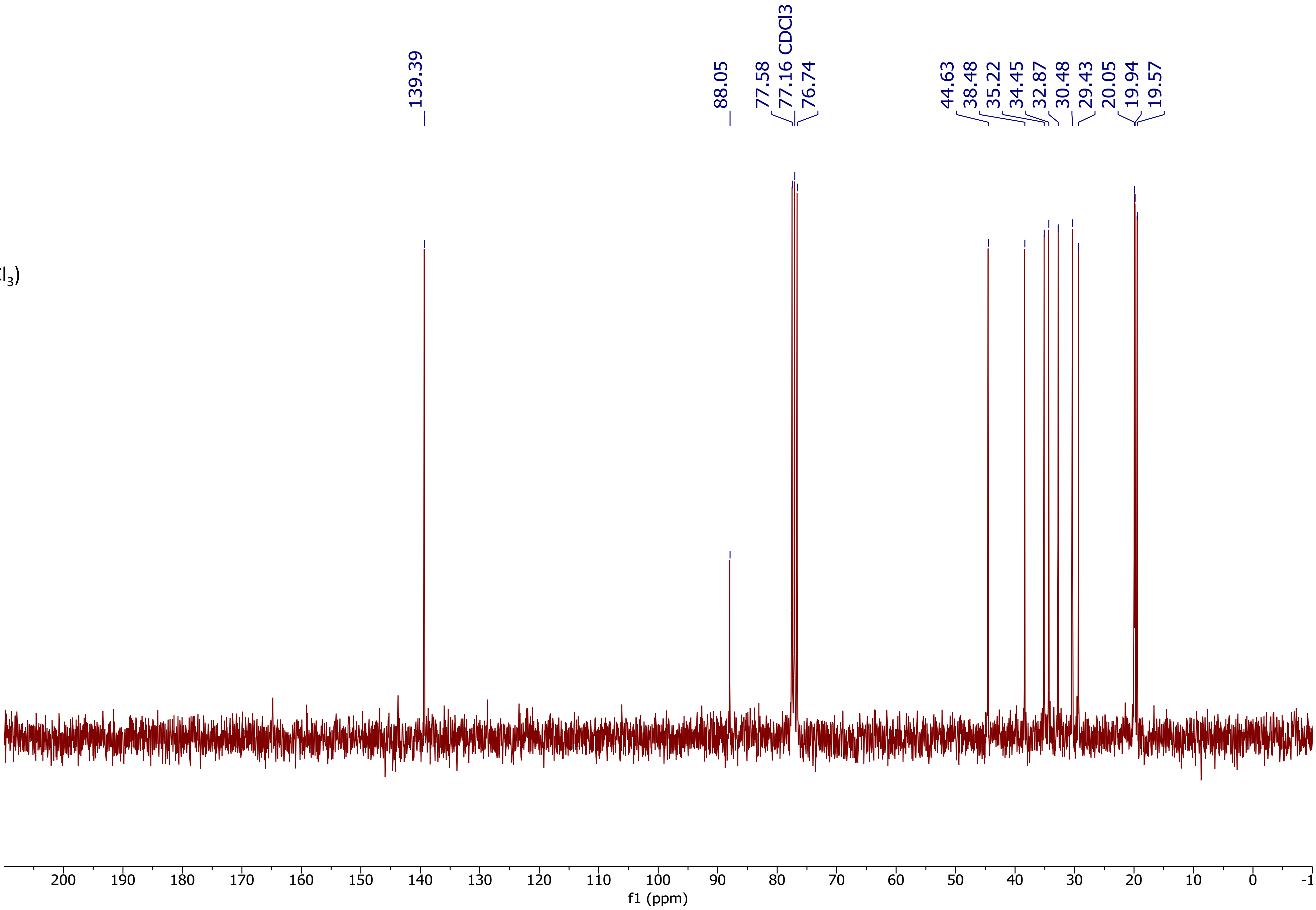

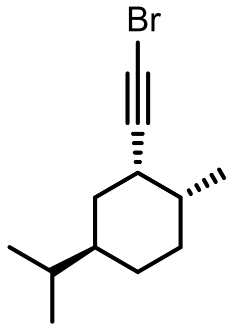

1d

^{1}H NMR(300 MHz, $CDCl_3$)

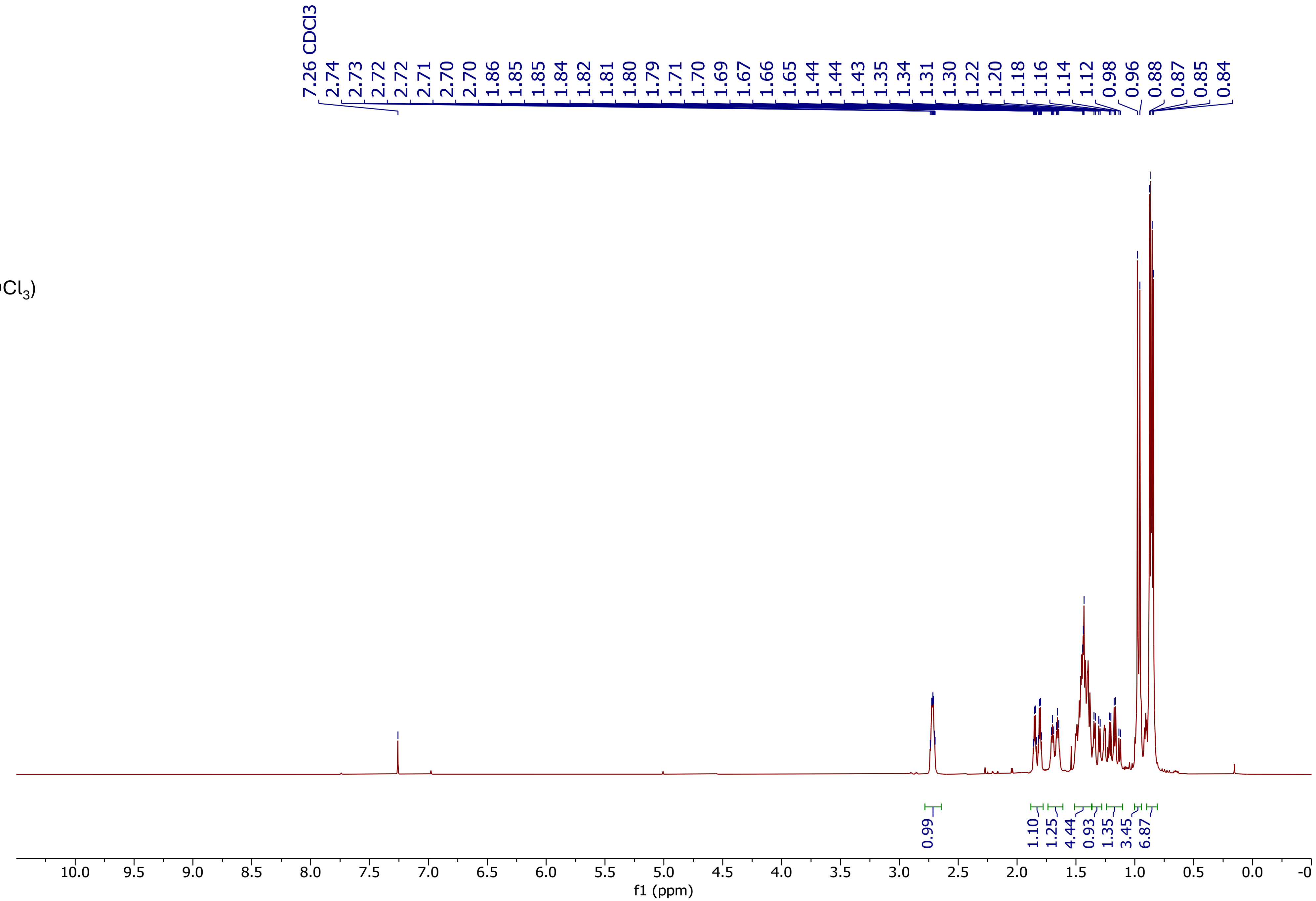

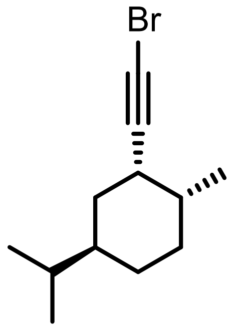

1d

^{13}C NMR (75 MHz, $CDCl_3$)

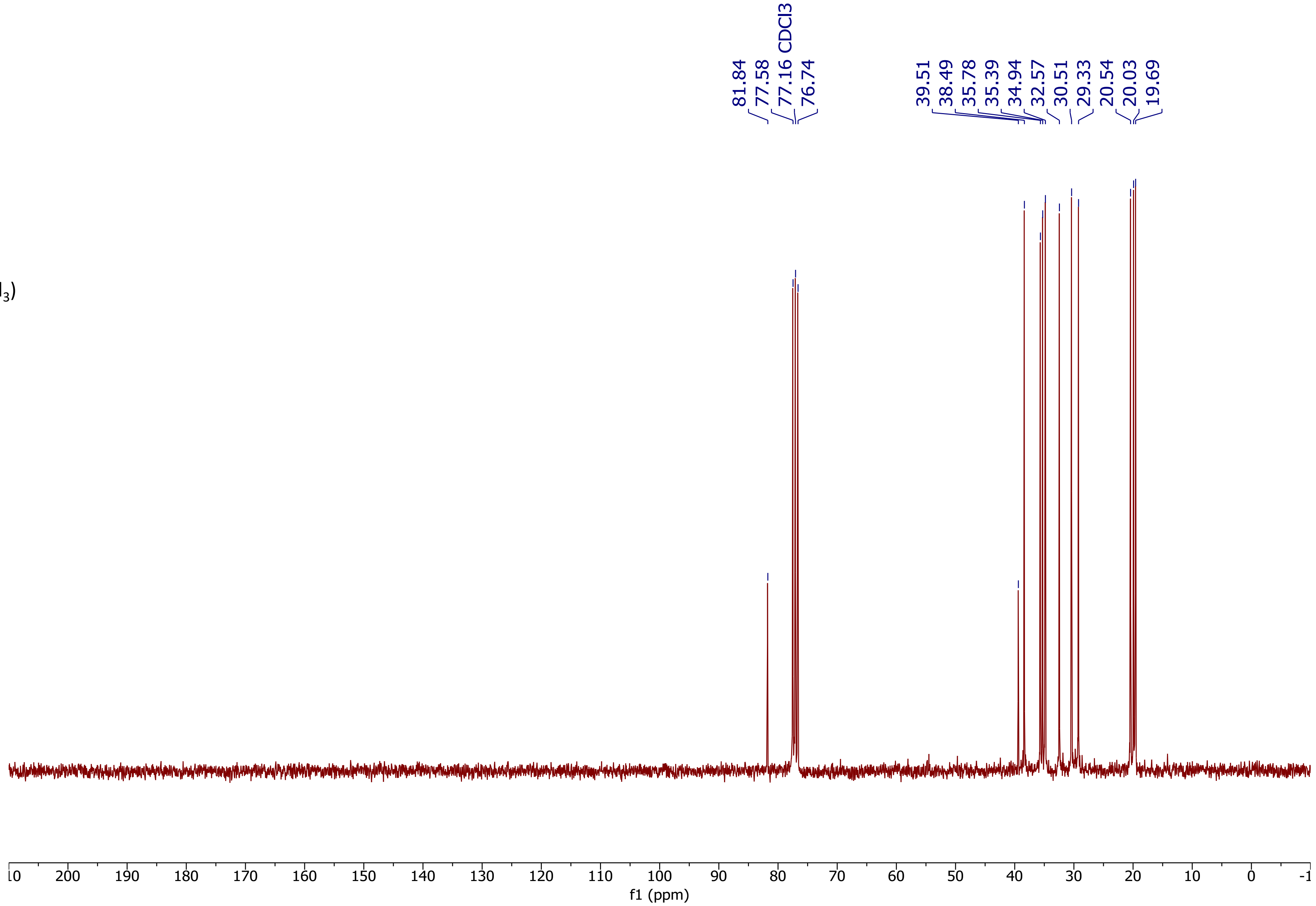

SUBSTRATES FOR THE FUSED FAMILY: 2a-d

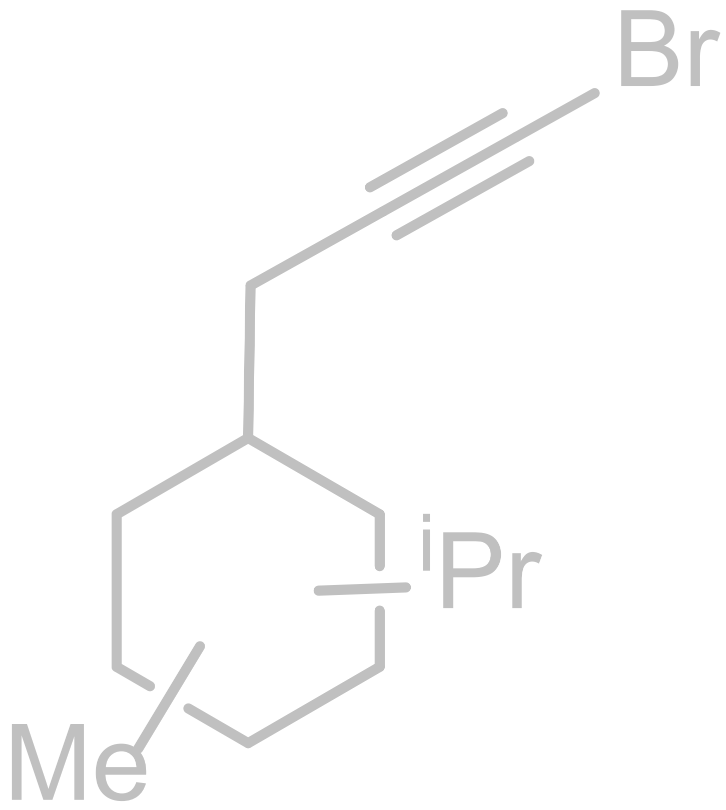

2a-d

2a,b

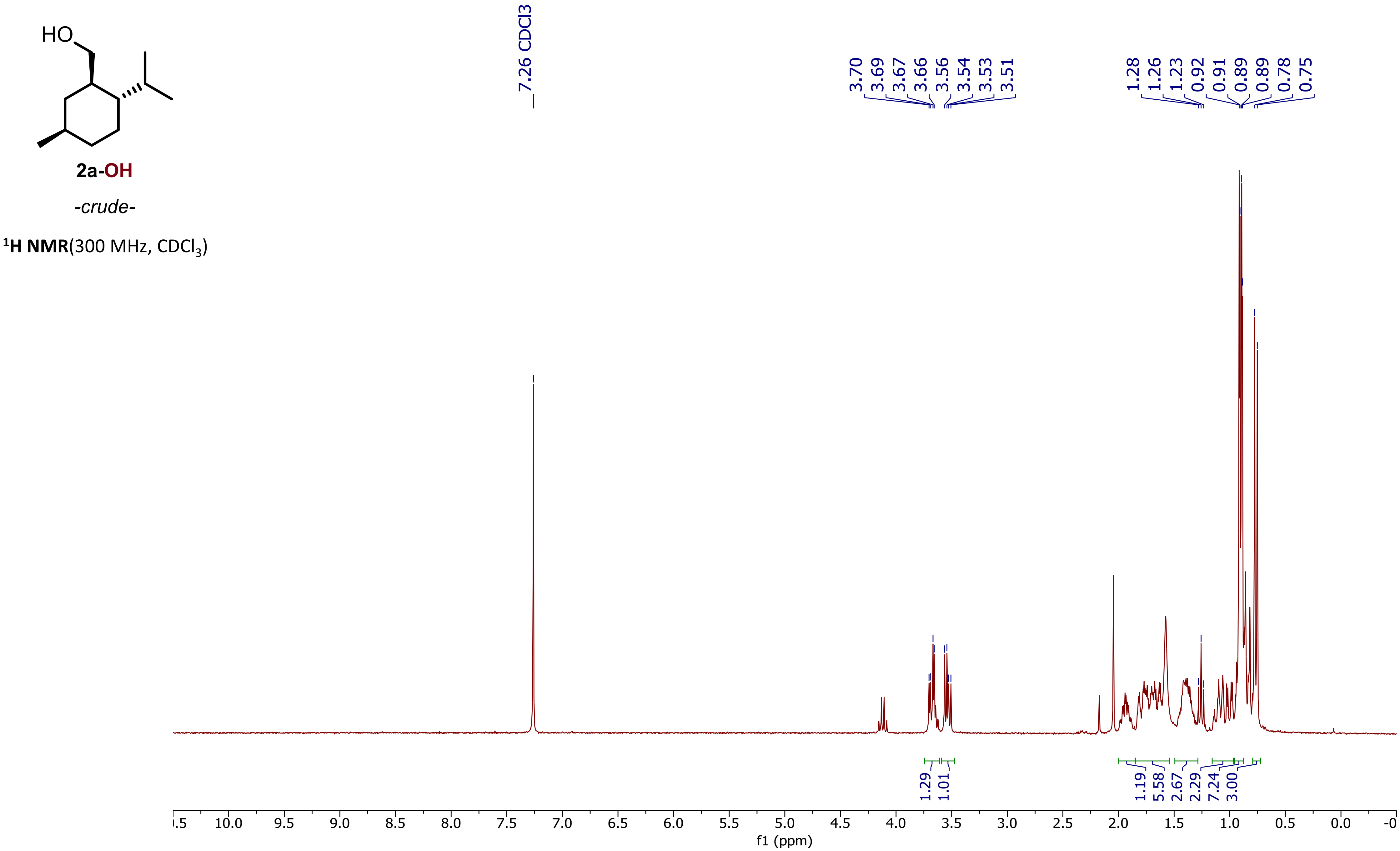
HO
2a-OH
-crude-
¹H NMR(300 MHz, CDCl3)
7.26 CDCl3
3.70
3.69
3.67
3.66
3.56
3.54
3.53
3.51
1.28
1.26
1.23
0.92
0.91
0.89
0.89
0.78
0.75
1.29
1.01
1.19
5.58
2.67
2.29
7.24
3.00
f1 (ppm)

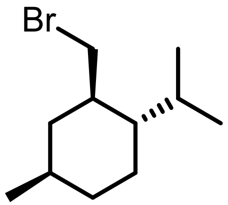

2a-Br

^{1}H NMR(300 MHz, $CDCl_3$)

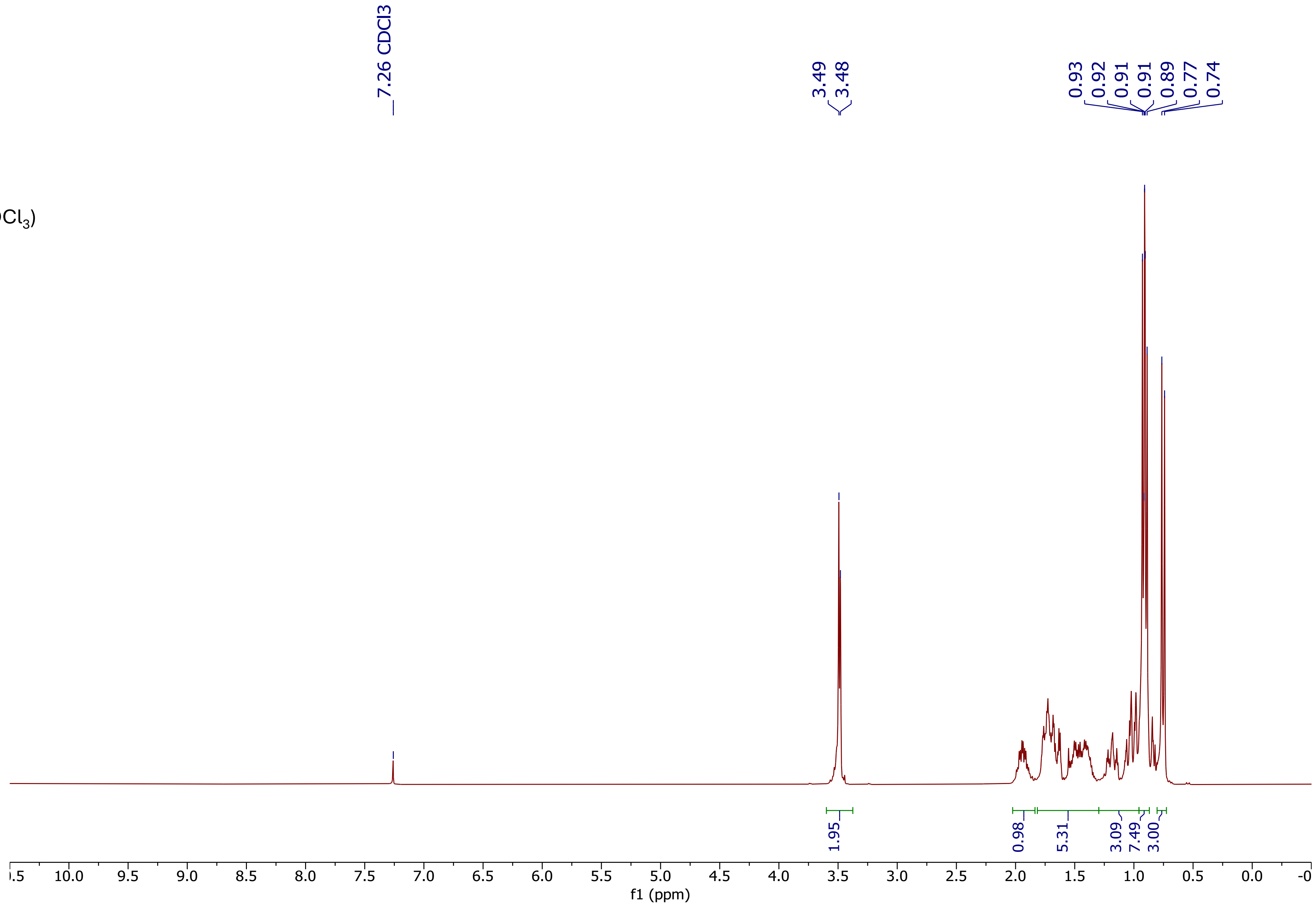

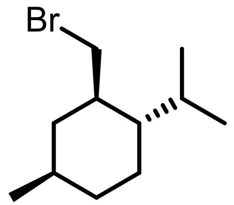

2a-Br

^{13}C NMR (75 MHz, $CDCl_3$)

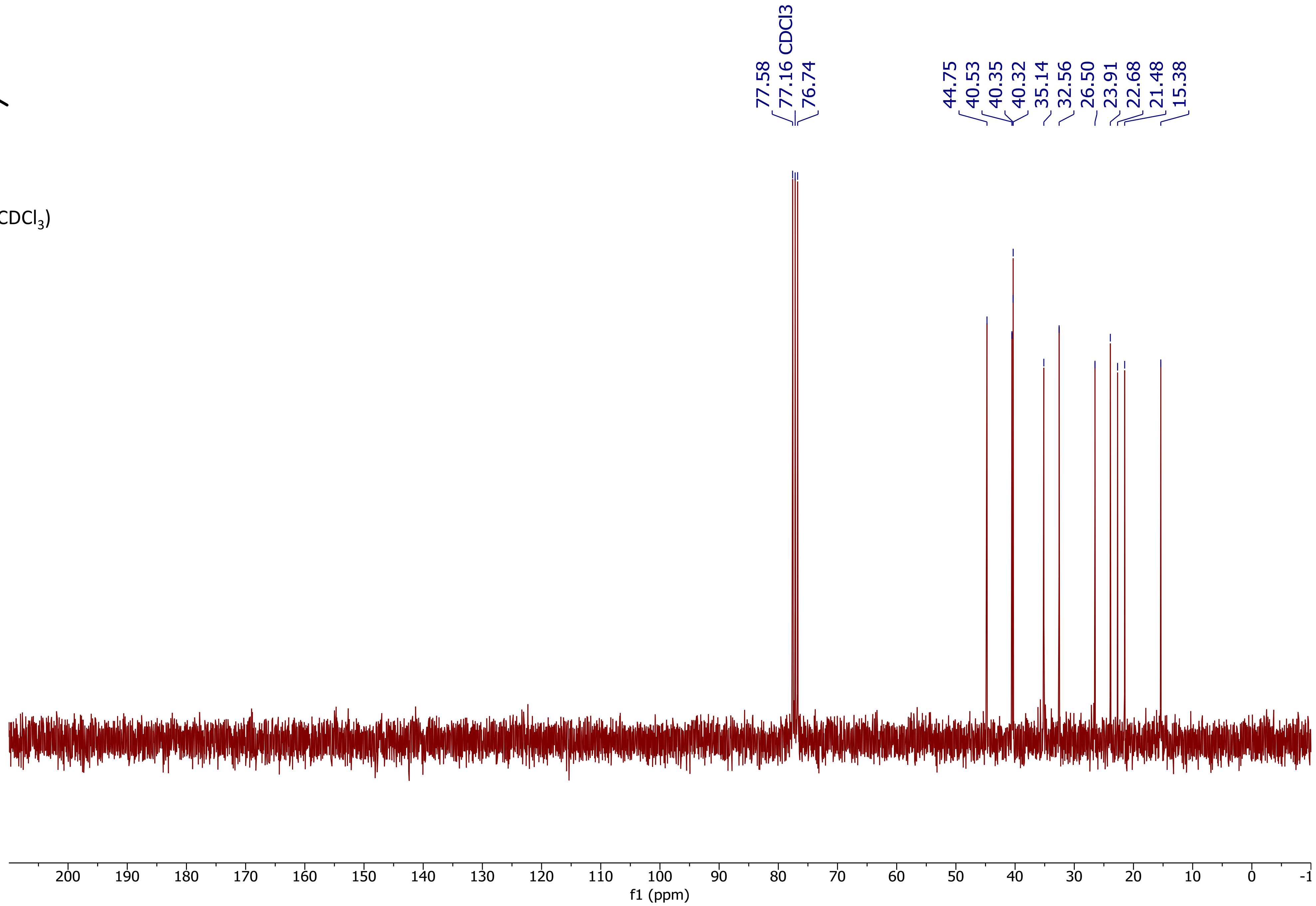

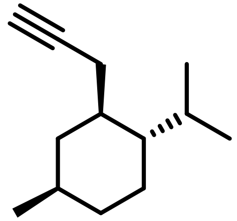

2a-CCH

^{1}H NMR(300 MHz, $CDCl_3$)

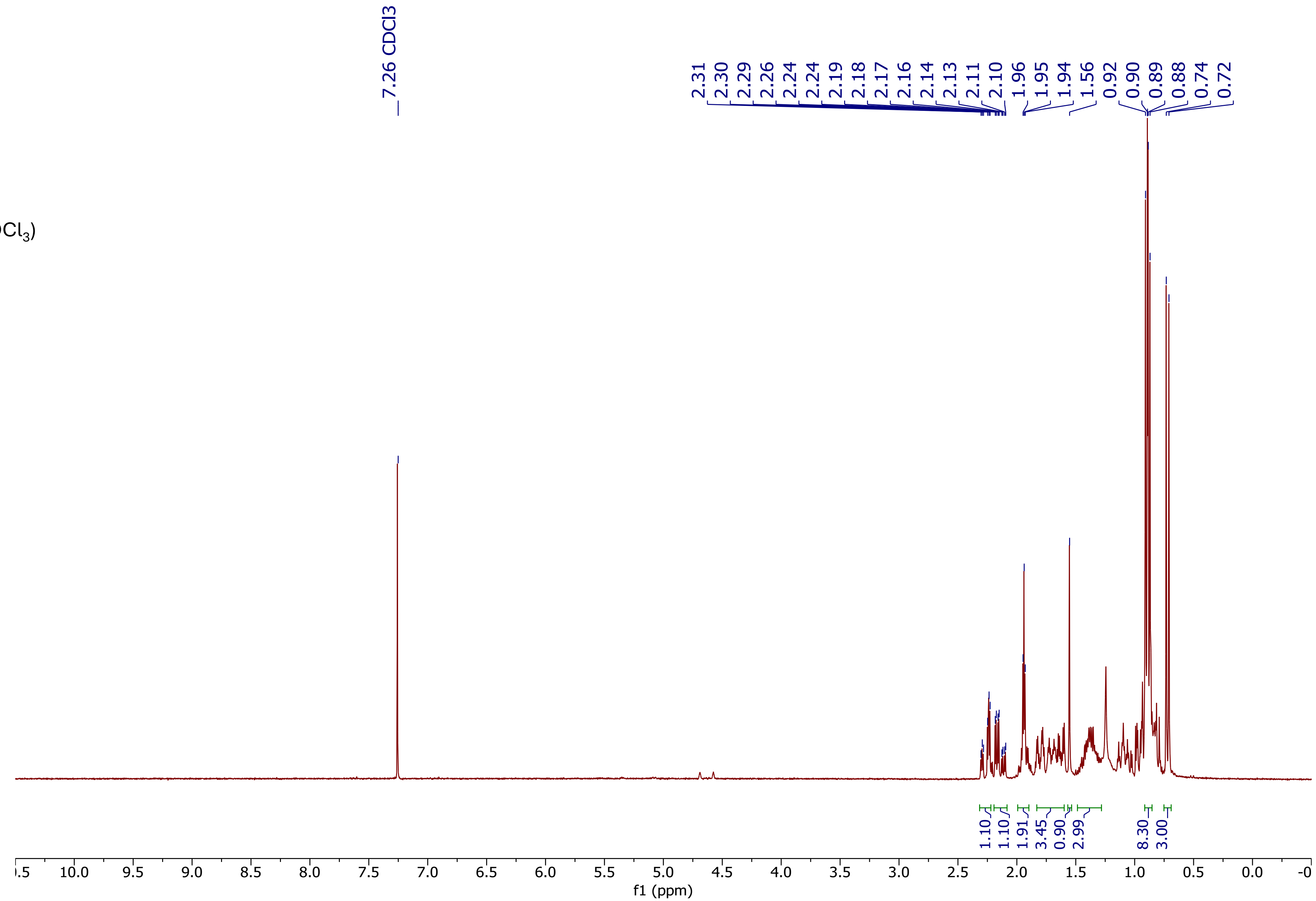

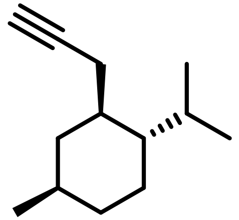

2a-CCH

^{13}C NMR (75 MHz, $CDCl_3$)

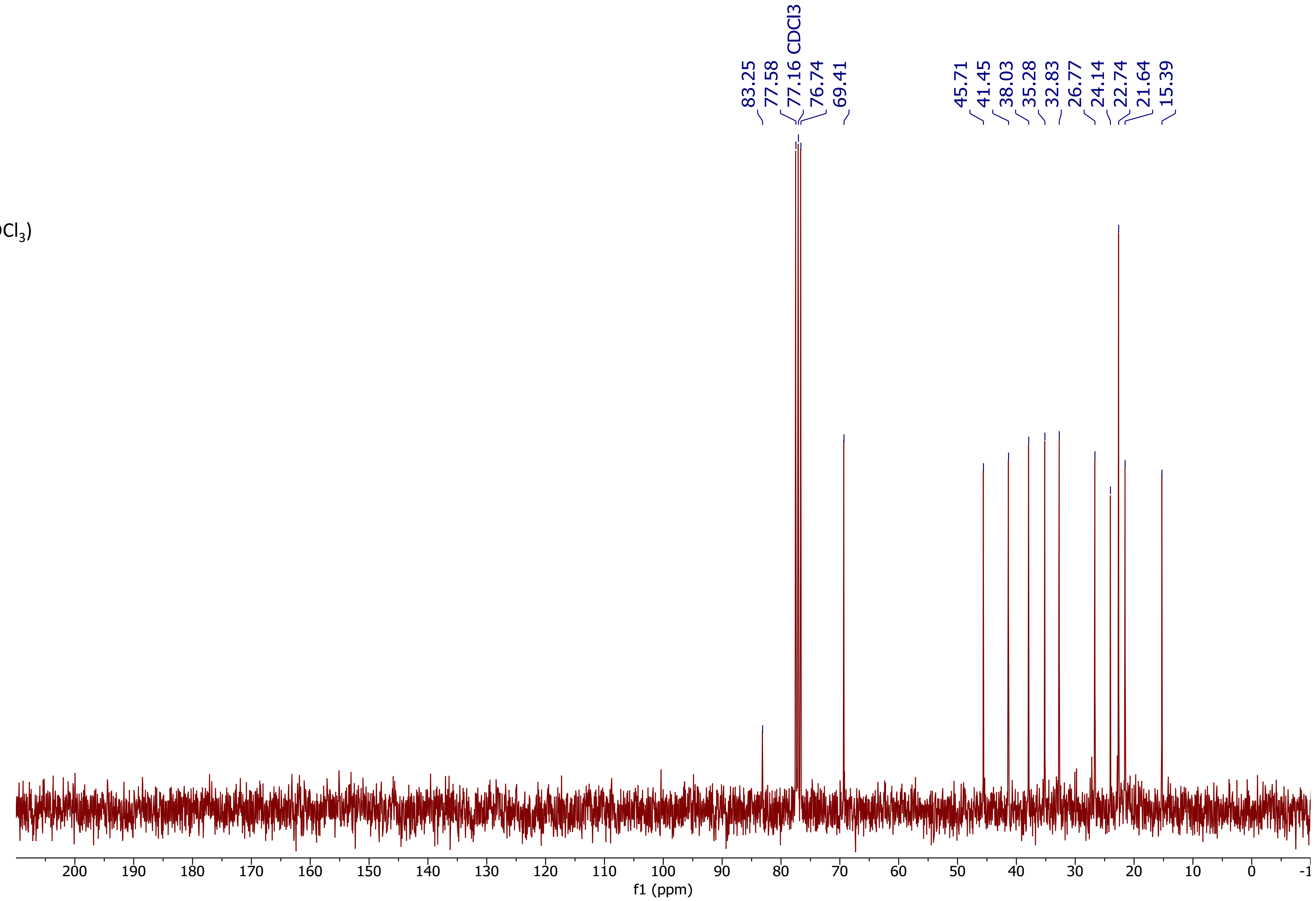

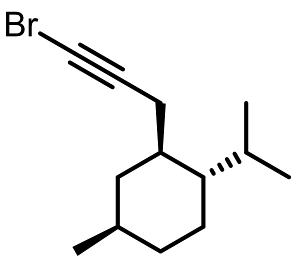

2a

^{1}H NMR(300 MHz, $CDCl_3$)

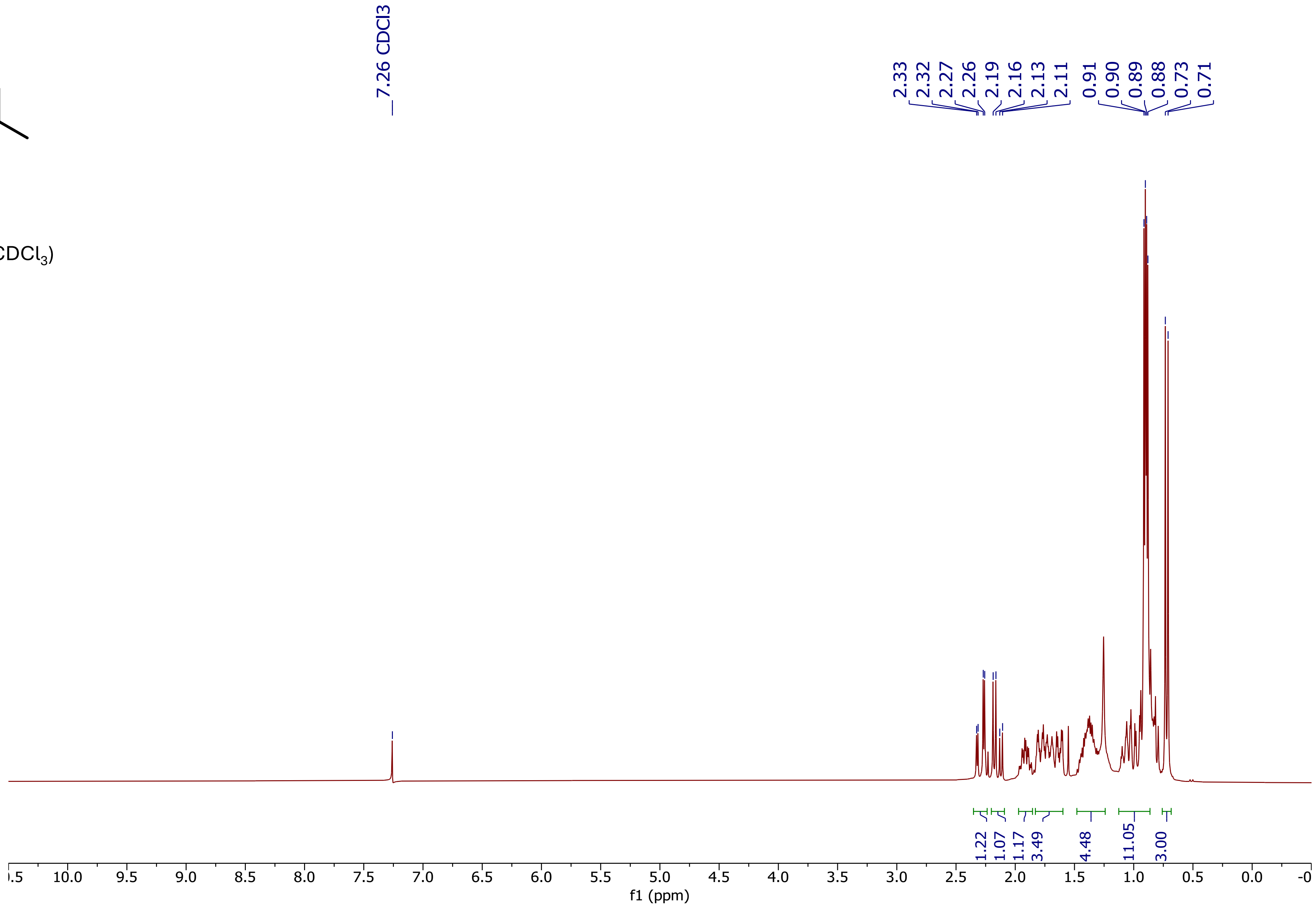

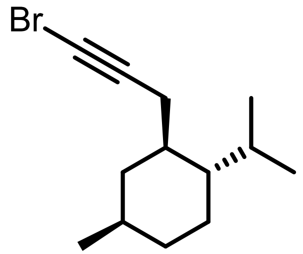

2a

[13]**C NMR** (75 MHz, $CDCl_3$)

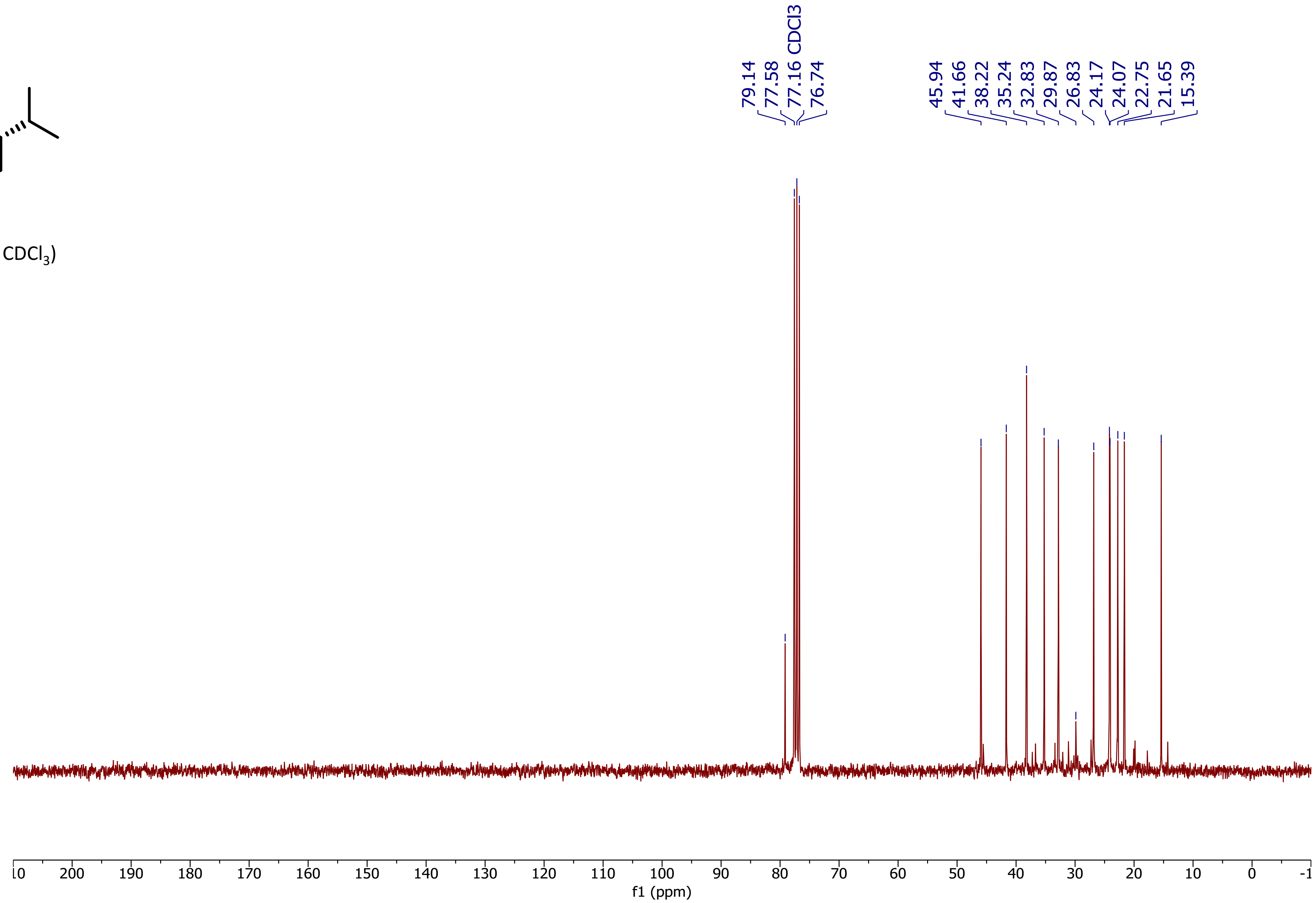

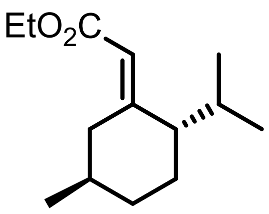

2b-CO_2Et

^{1}H NMR(300 MHz, $CDCl_3$)

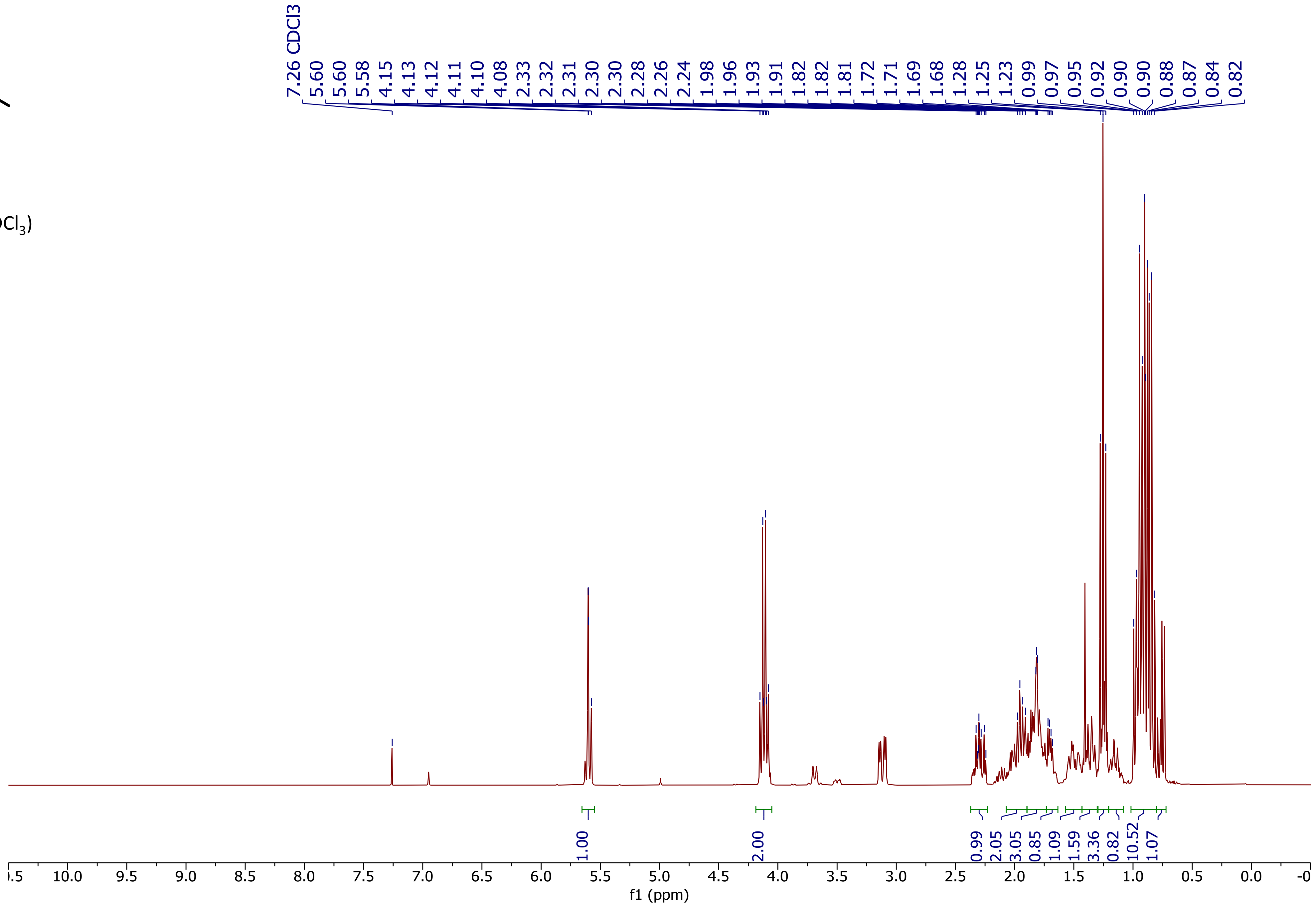

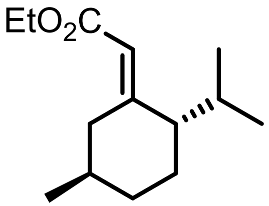

2b-CO_2Et

^{13}C NMR (75 MHz, $CDCl_3$)

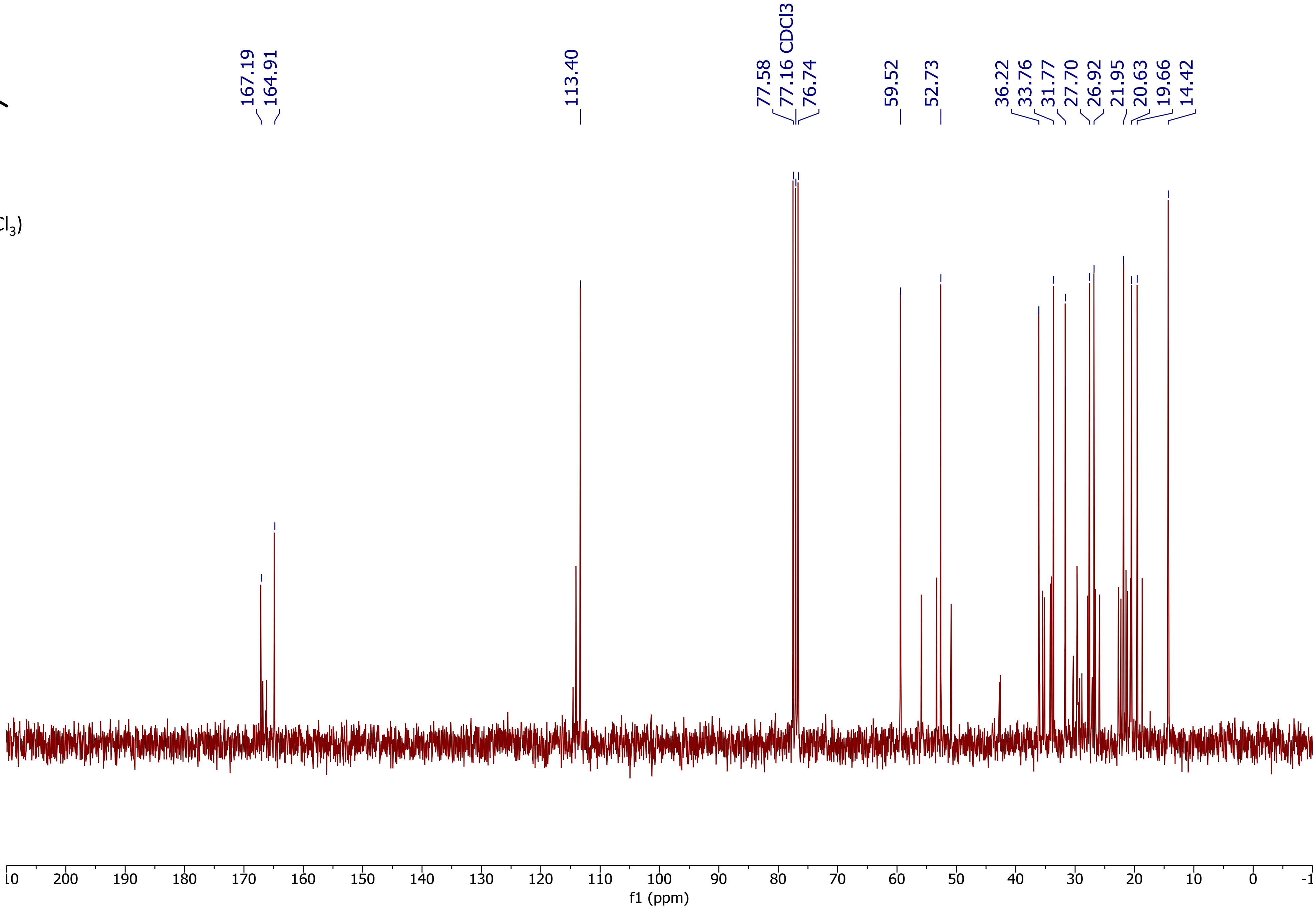

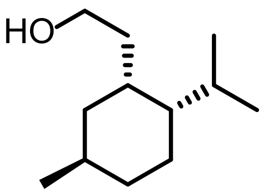

2b-OH

^{1}H NMR(300 MHz, $CDCl_3$)

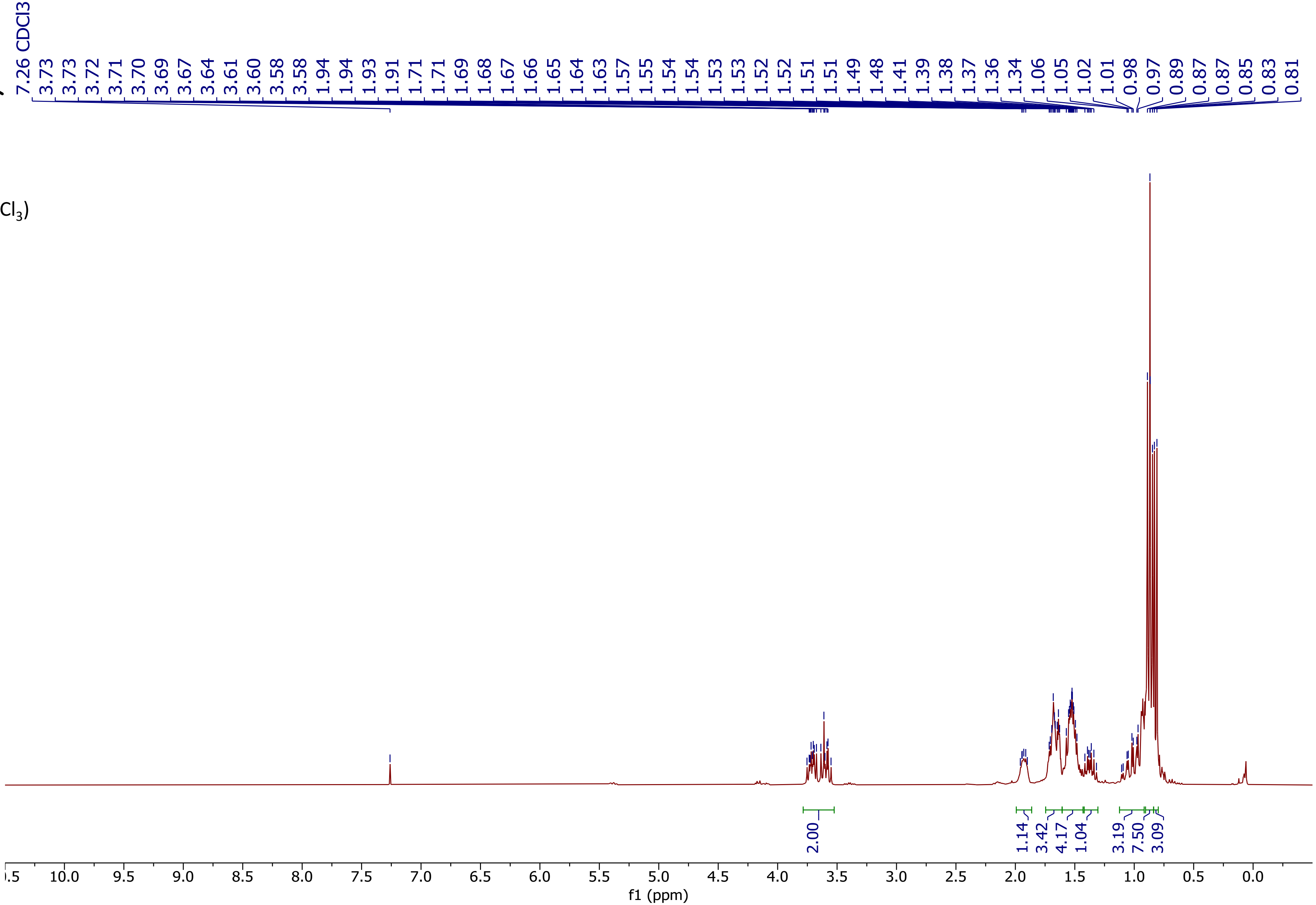

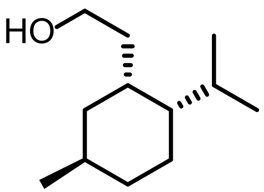

2b-OH

^{13}C NMR (75 MHz, $CDCl_3$)

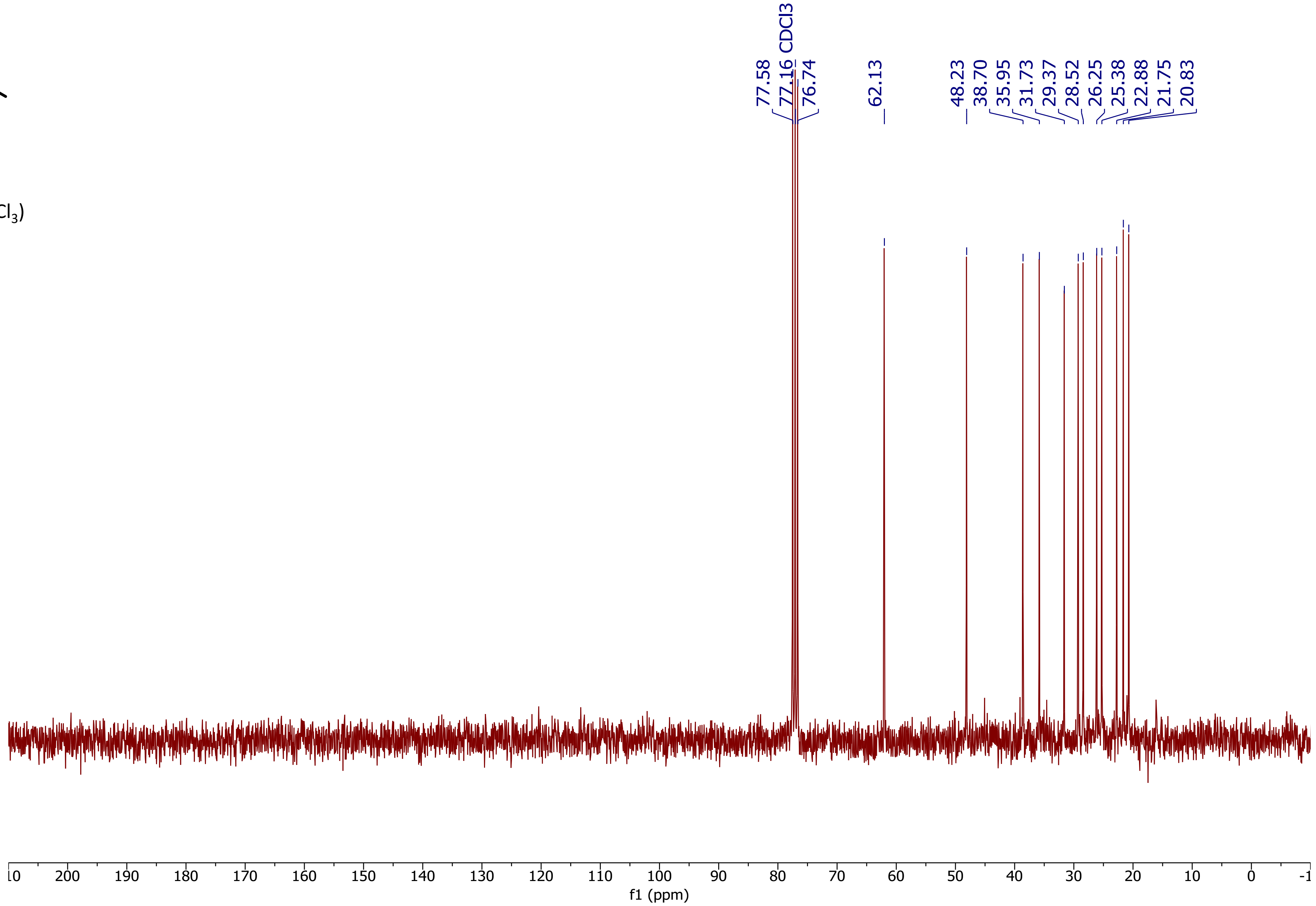

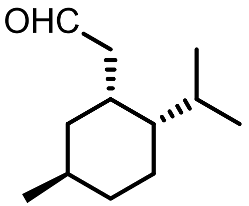

2b-CHO

-crude-

^{1}H NMR(300 MHz, $CDCl_3$)

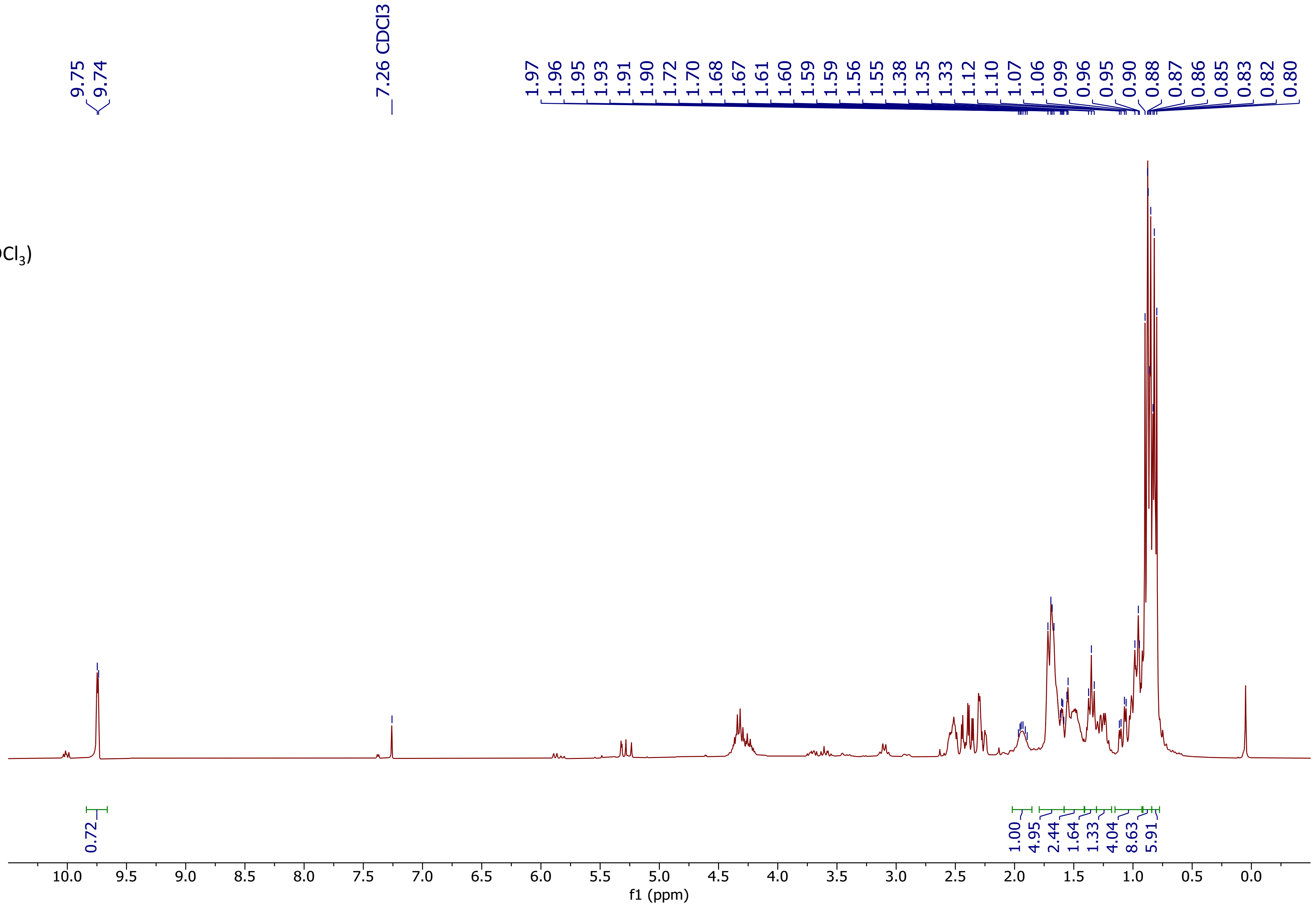

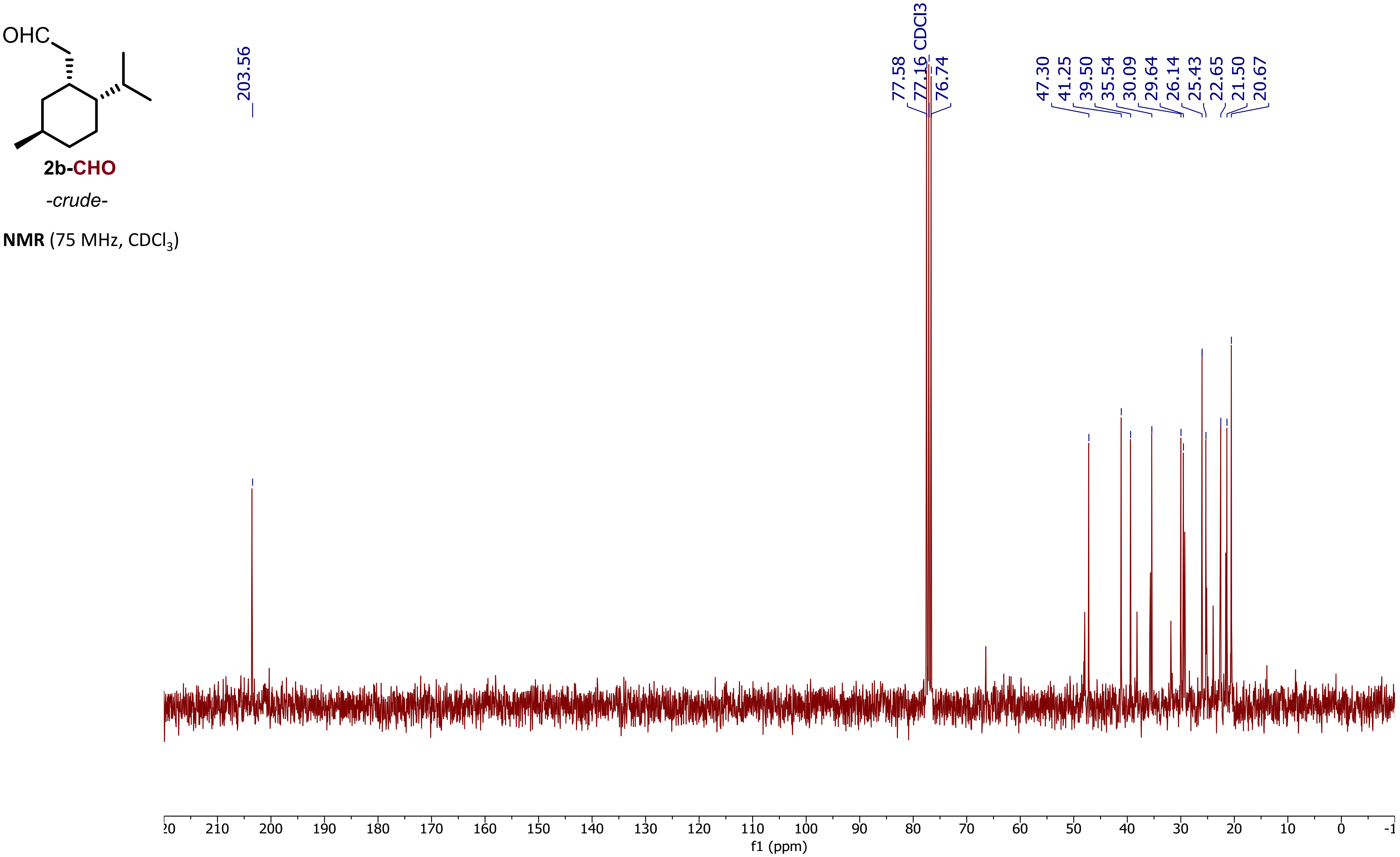

2b-CHO

-crude-

^{13}C NMR (75 MHz, $CDCl_3$)

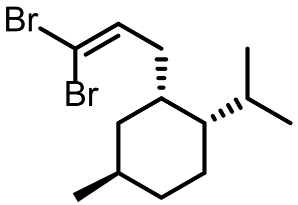

2b-CBr$_2$

^{1}H NMR(400 MHz, CDCl$_3$)

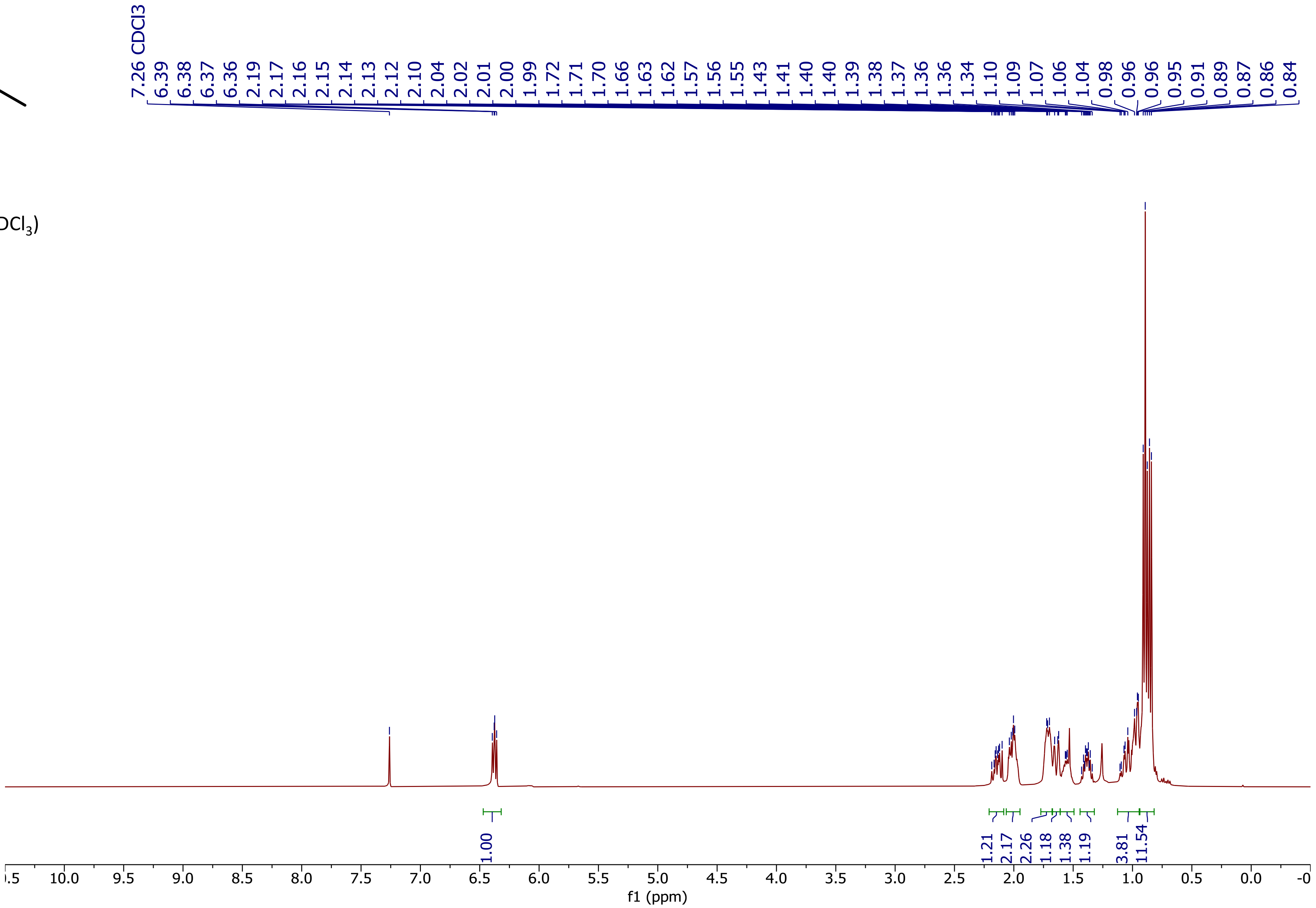

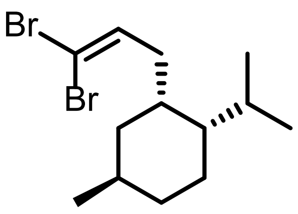

2b-CBr_2

^{13}C NMR (101 MHz, $CDCl_3$)

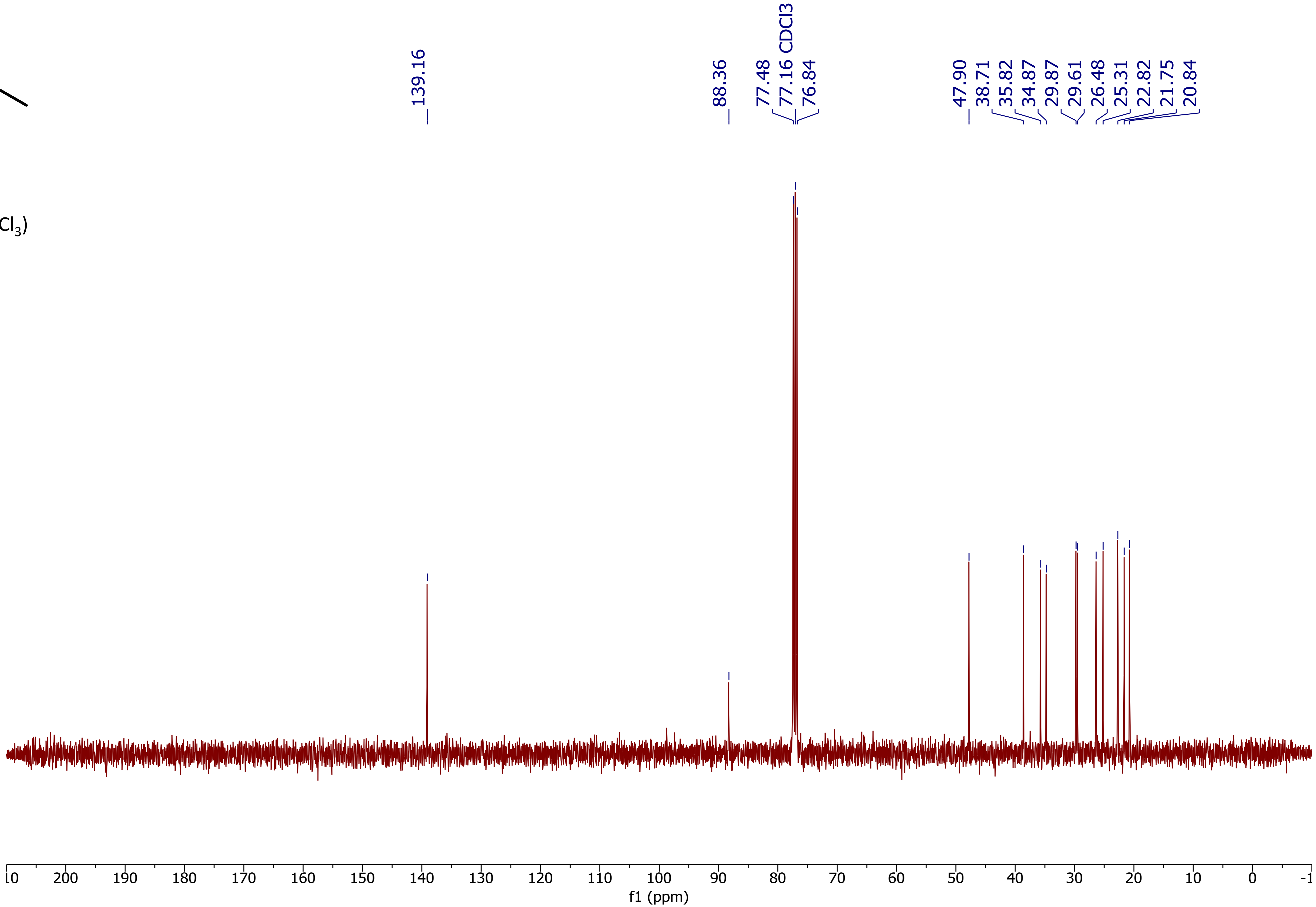

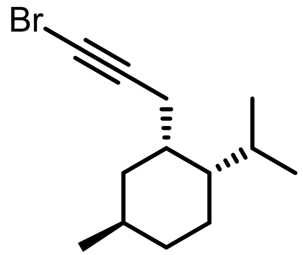

2b

^{1}H NMR(400 MHz, $CDCl_3$)

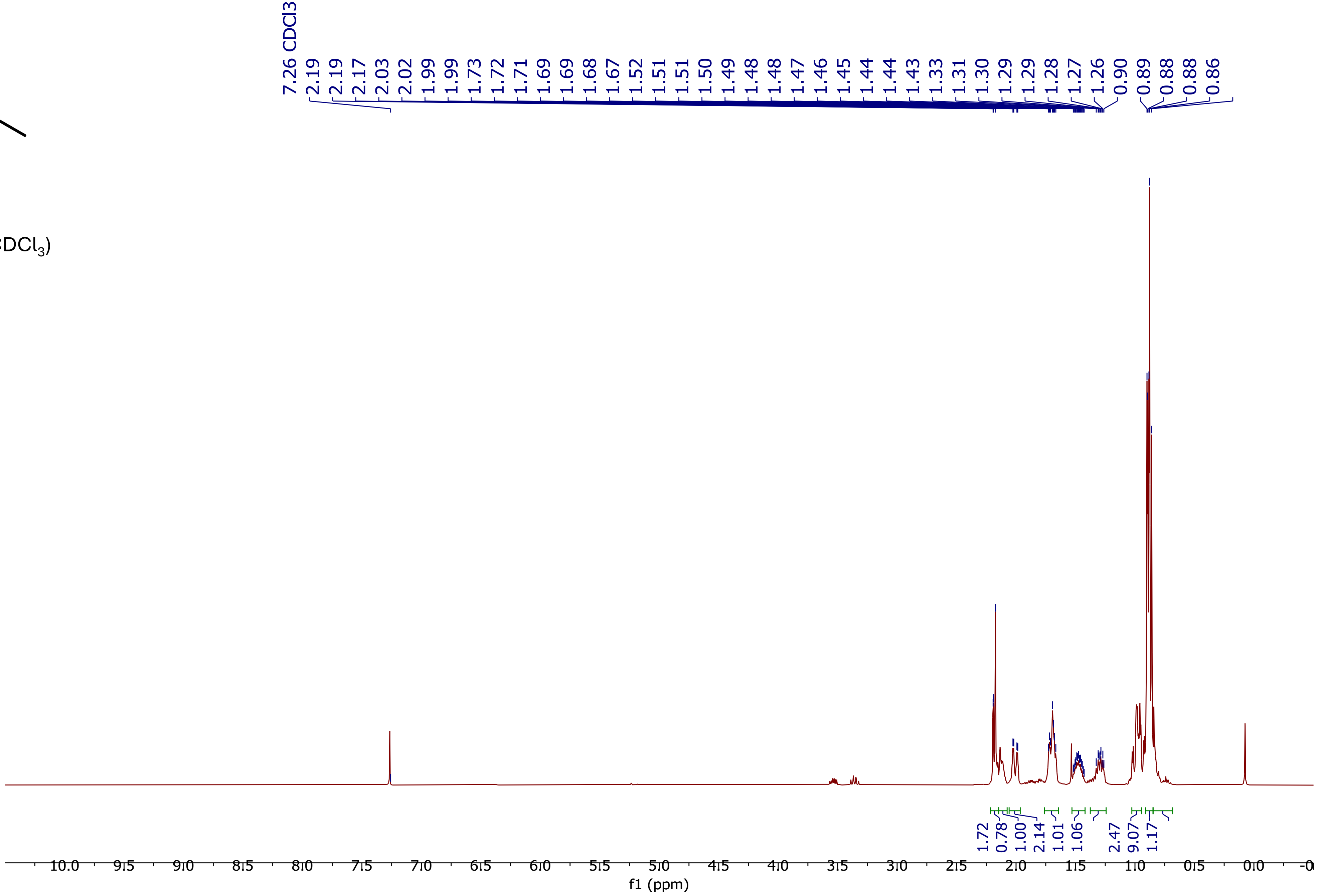

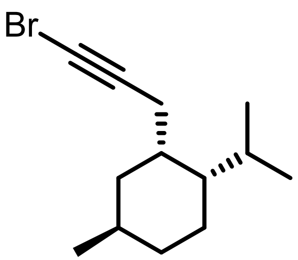

2b

^{13}C NMR (101 MHz, $CDCl_3$)

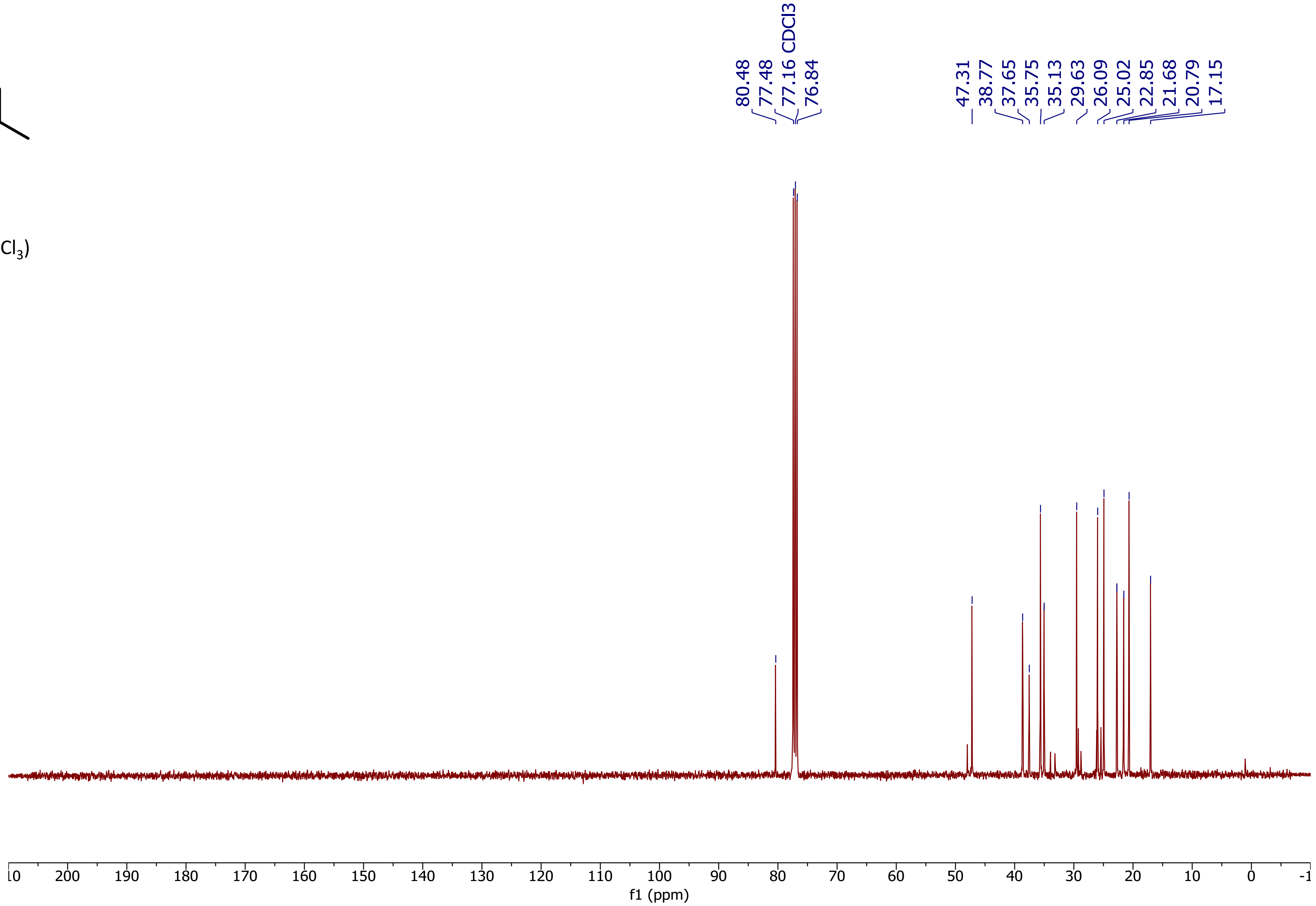

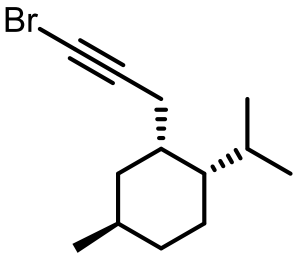

2b

COSY NMR([400, 400] MHz, $CDCl_3$)

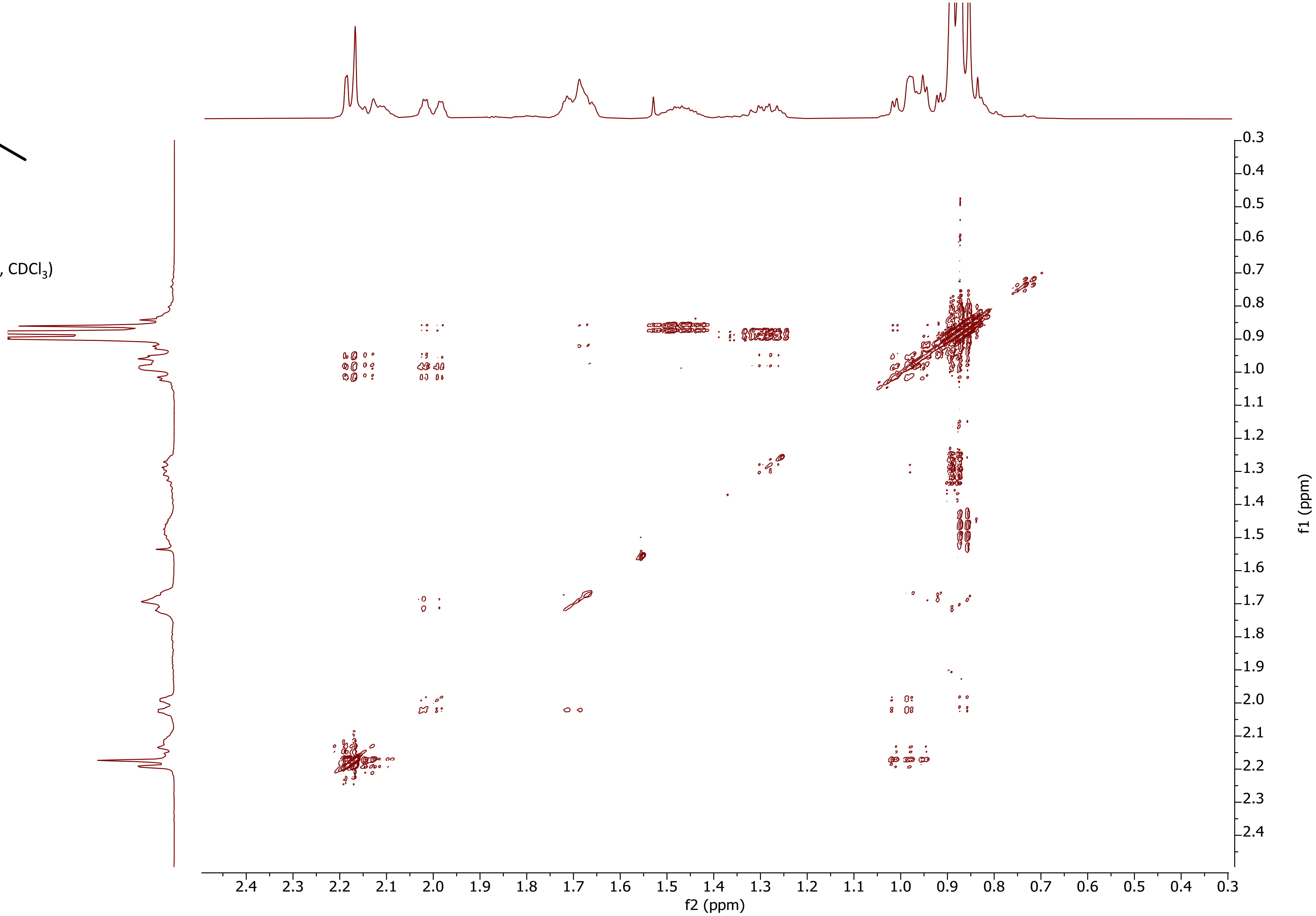

HSQC NMR([400, 101] MHz, $CDCl_3$)

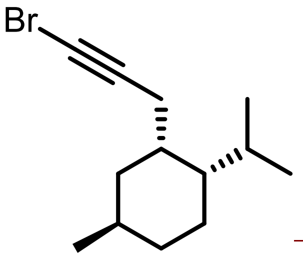

2b

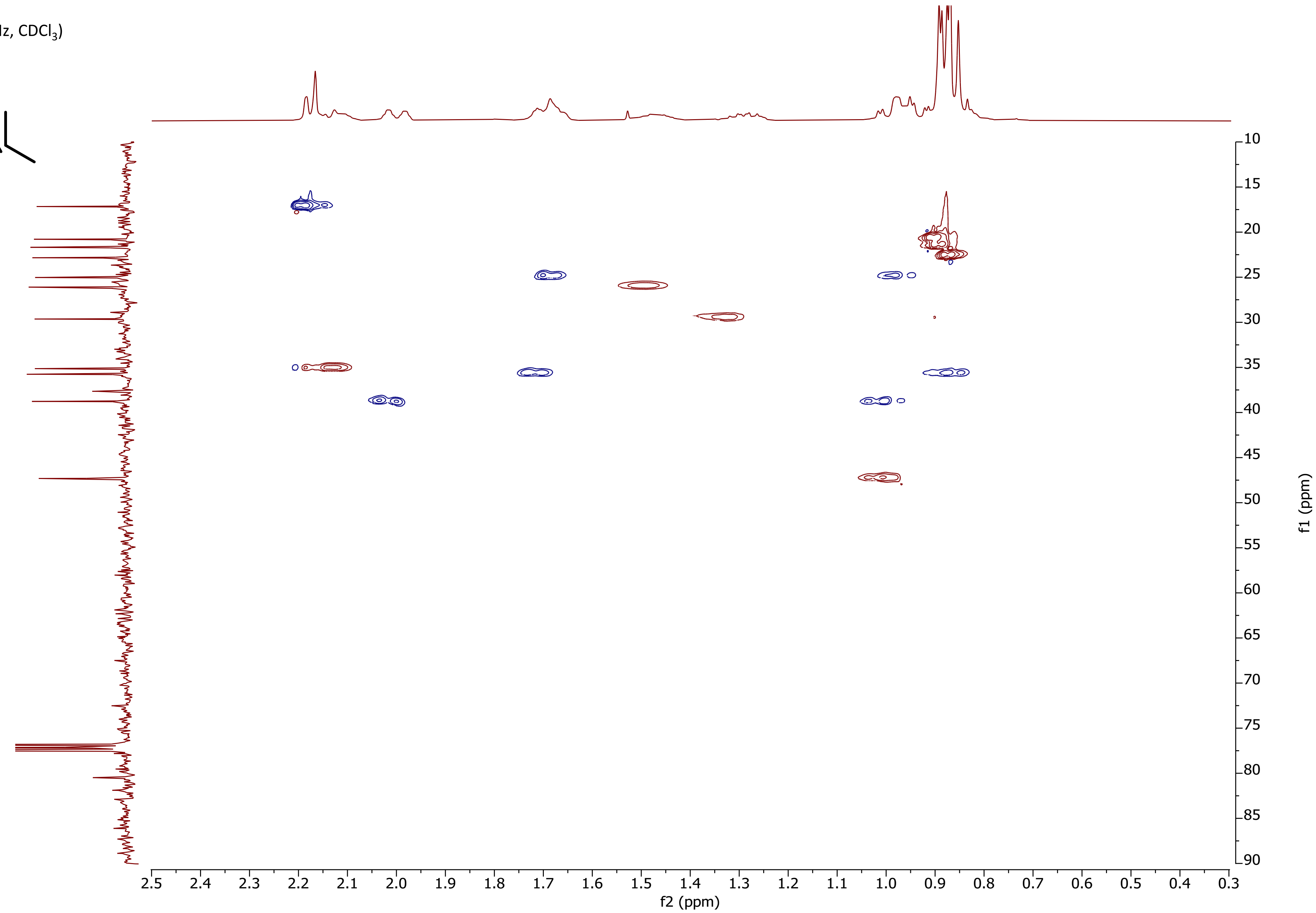

HMBC NMR([400, 101] MHz, $CDCl_3$)

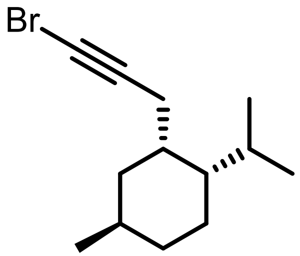

2b

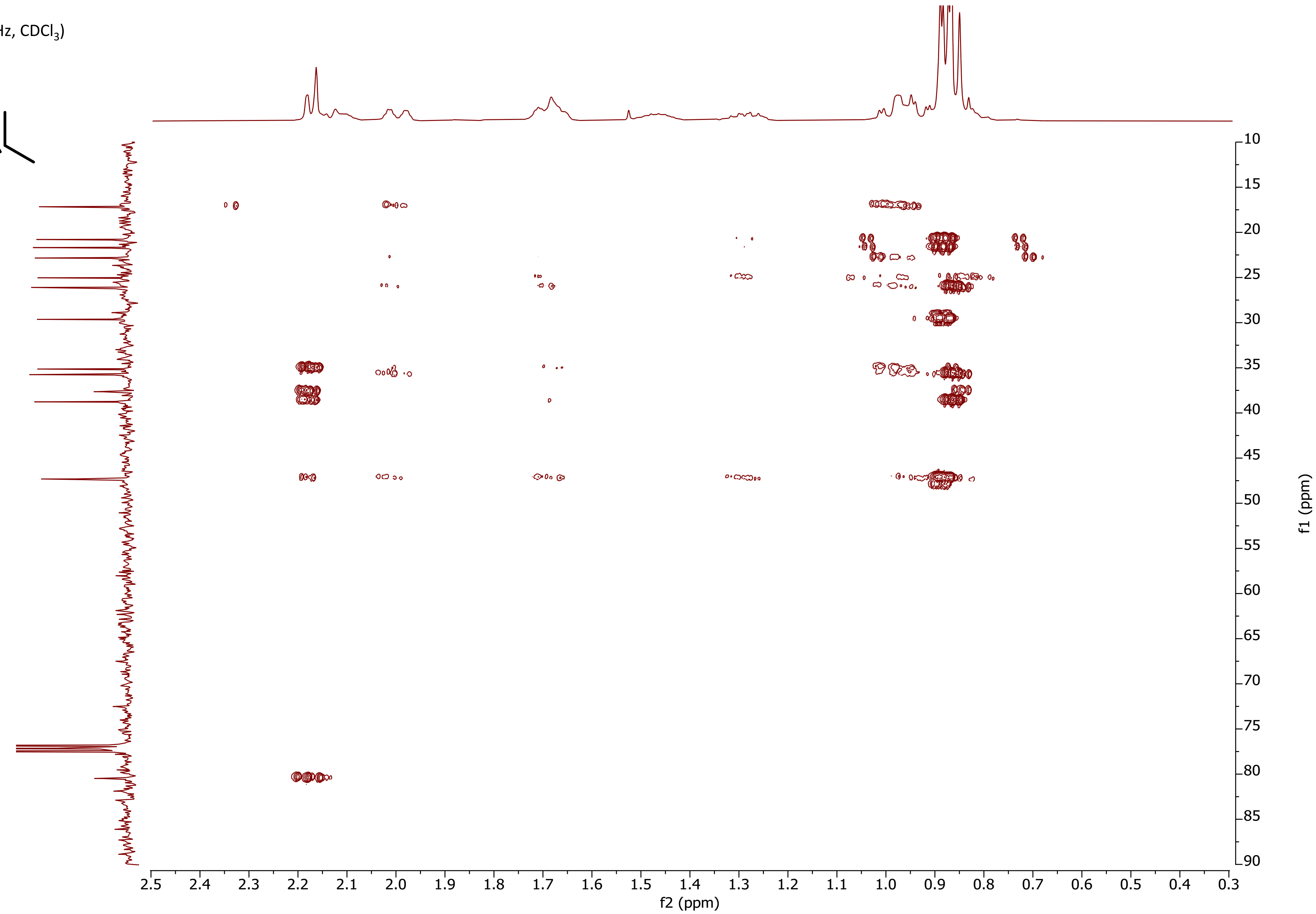

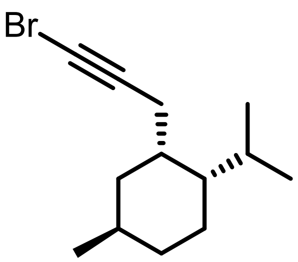

2b

NOESY NMR([400, 400] MHz, $CDCl_3$)

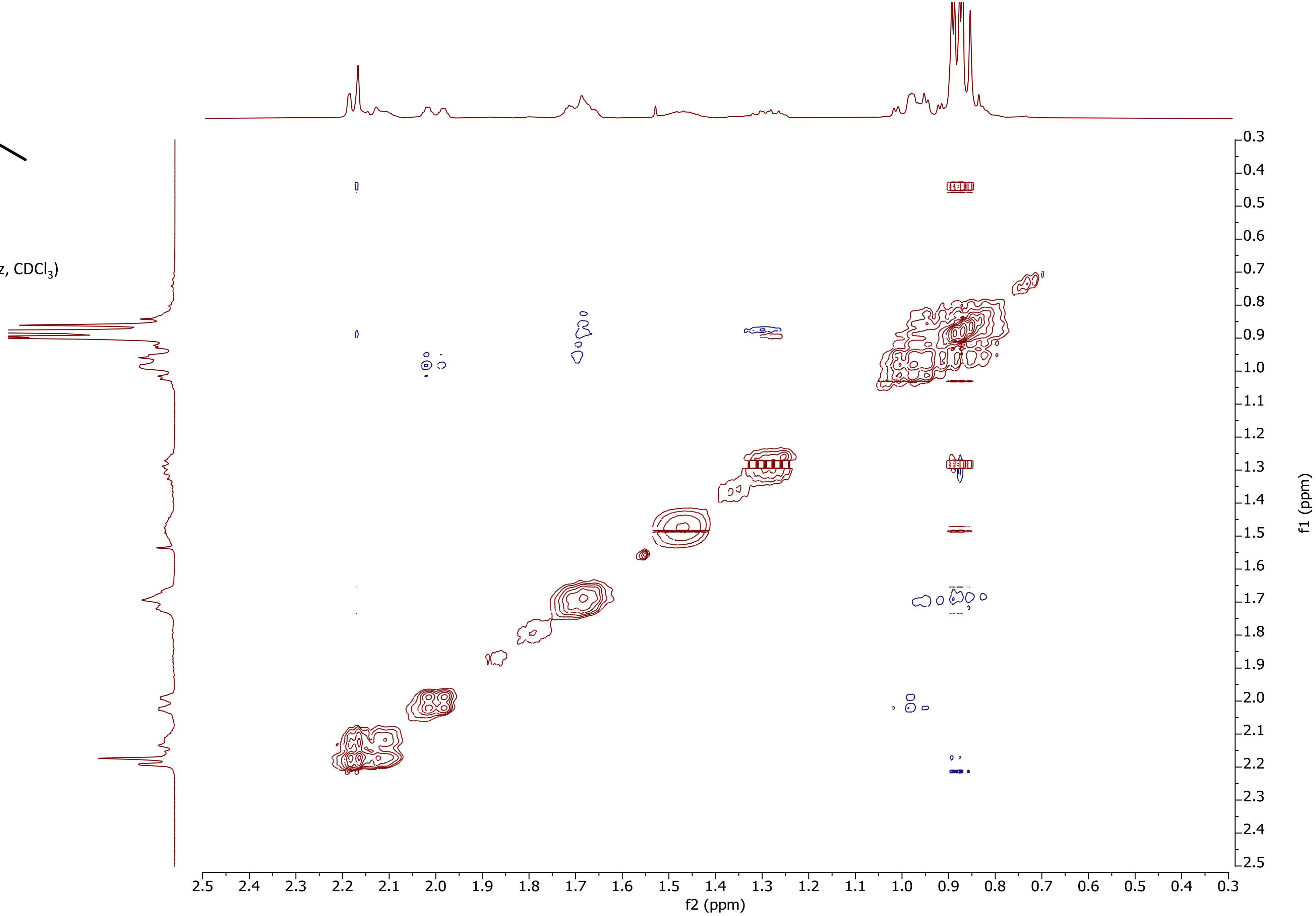

2c,d

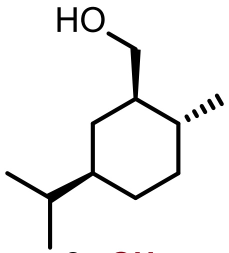

2c-OH
-crude-

^{1}H NMR(300 MHz, $CDCl_3$)

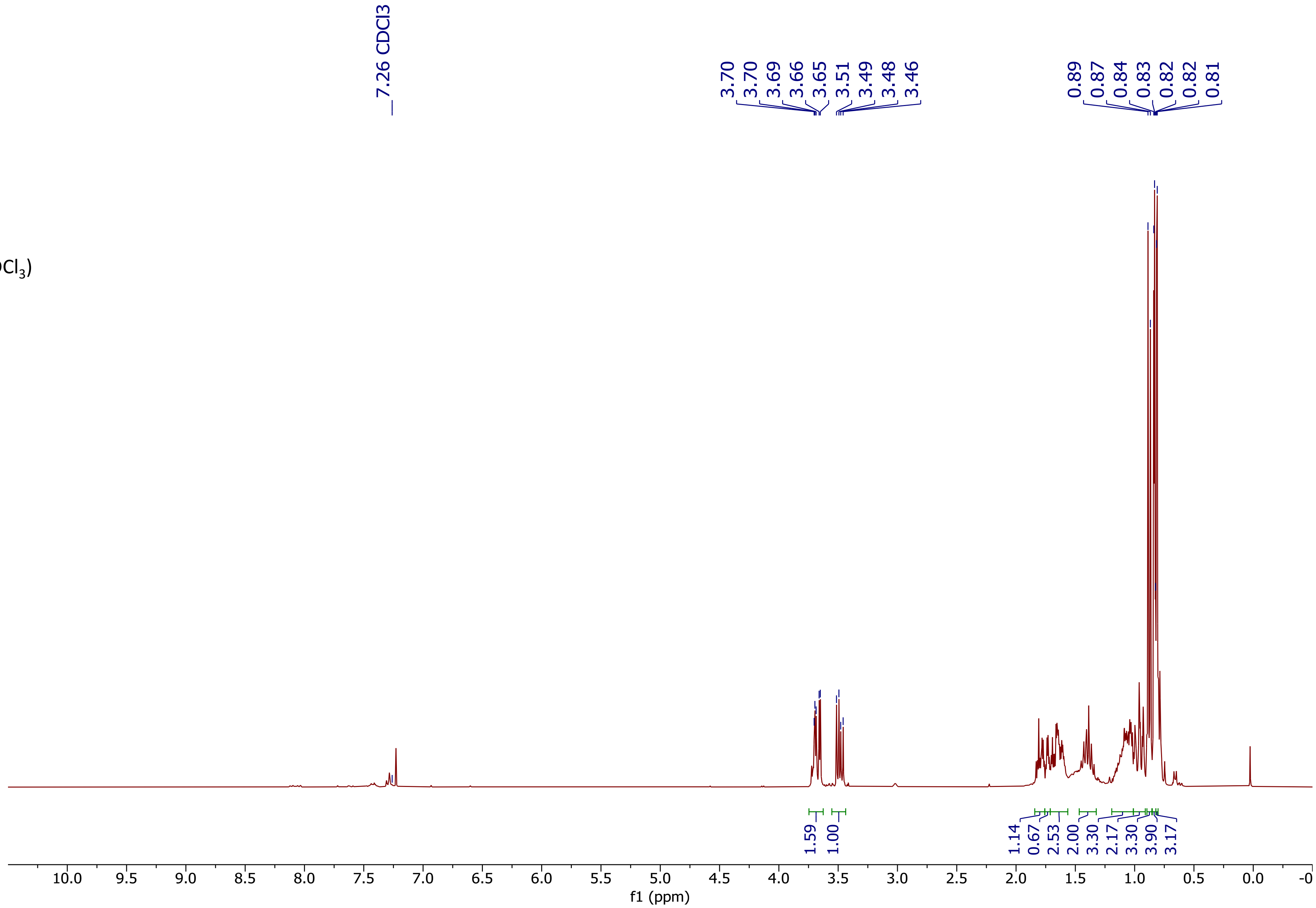

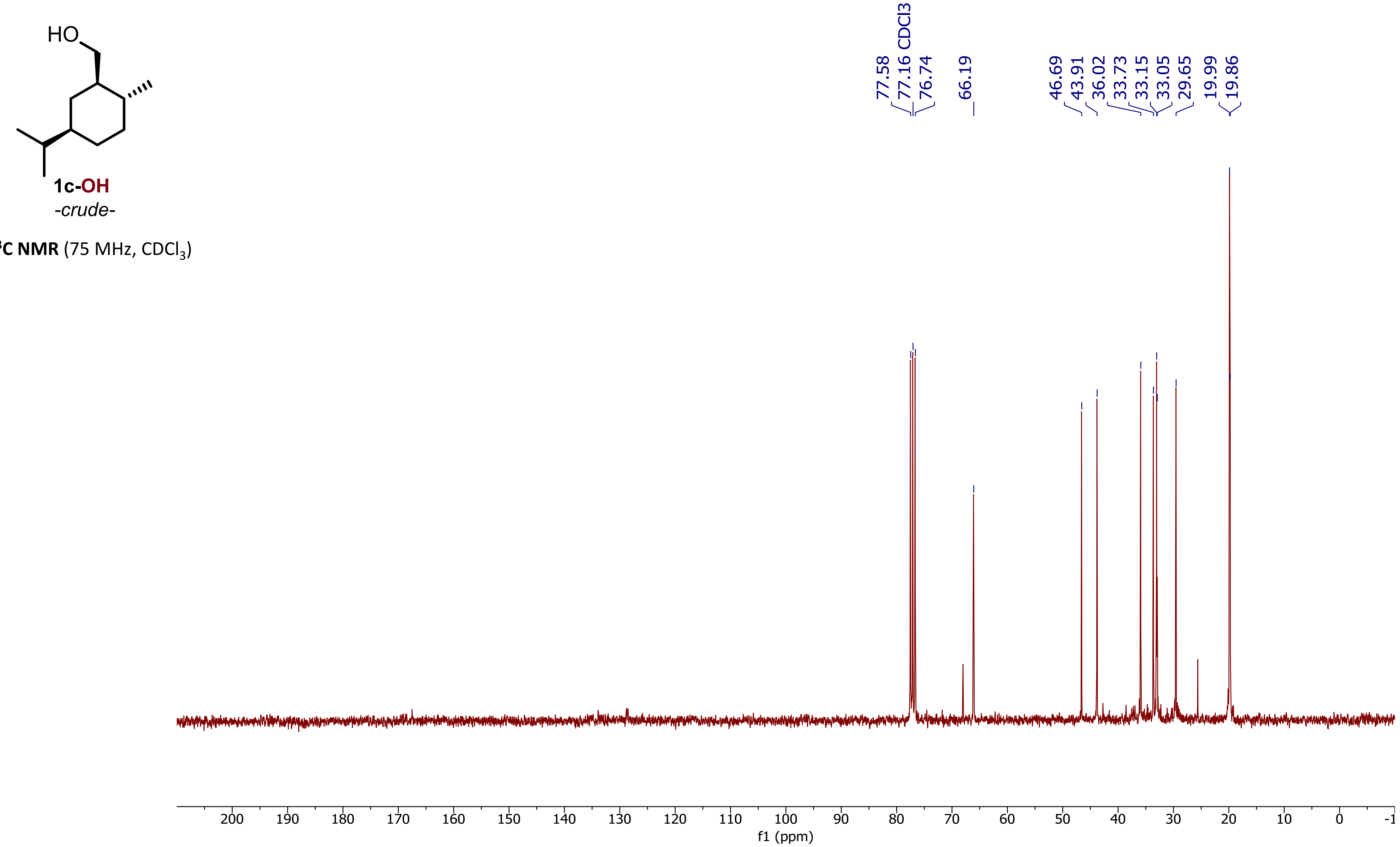

1c-OH

-crude-

^{13}C NMR (75 MHz, $CDCl_3$)

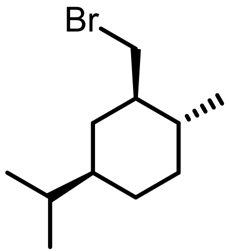

2c-Br

¹H NMR(300 MHz, $CDCl_3$)

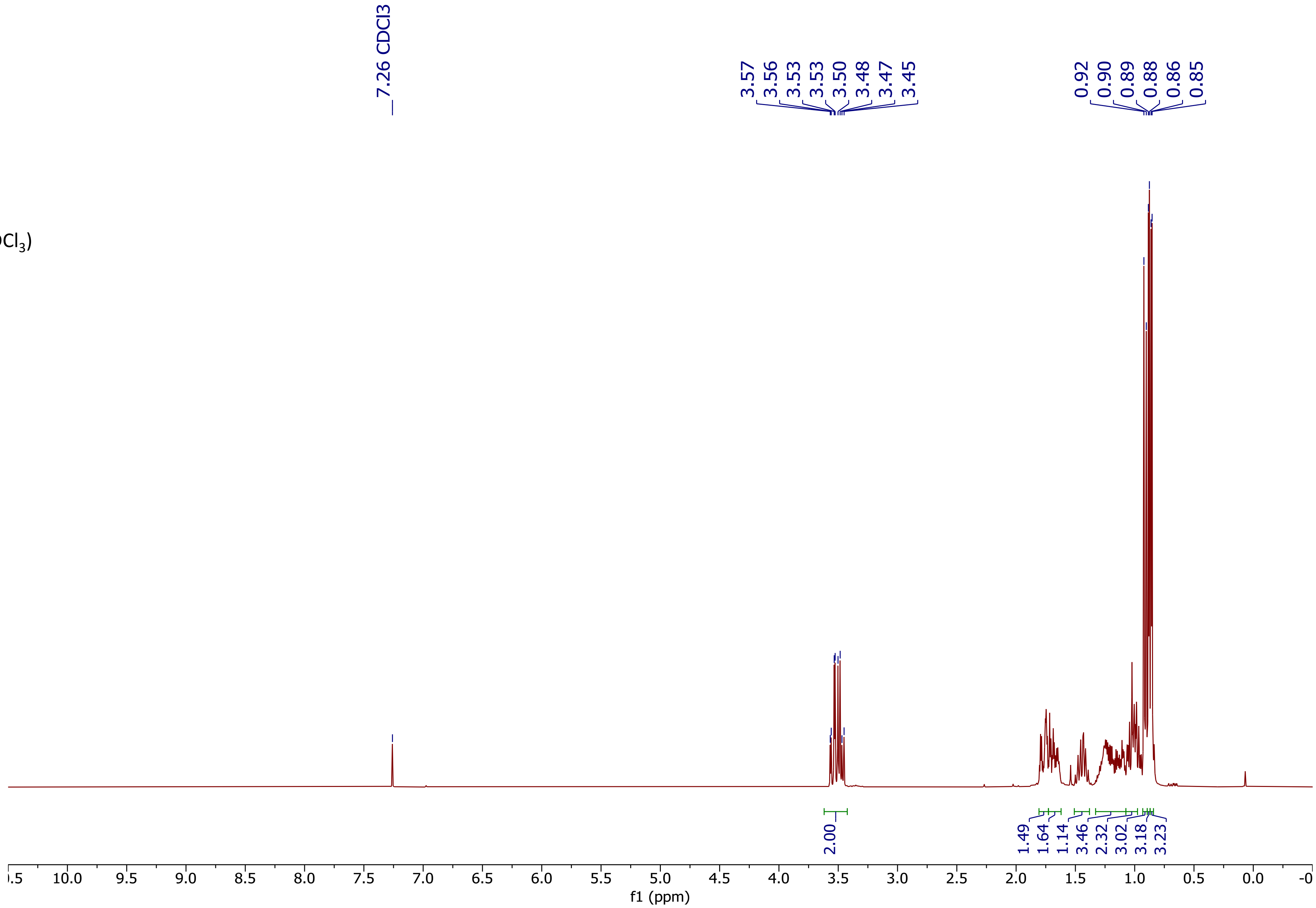

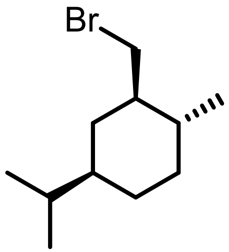

2c-Br

^{13}C NMR (75 MHz, $CDCl_3$)

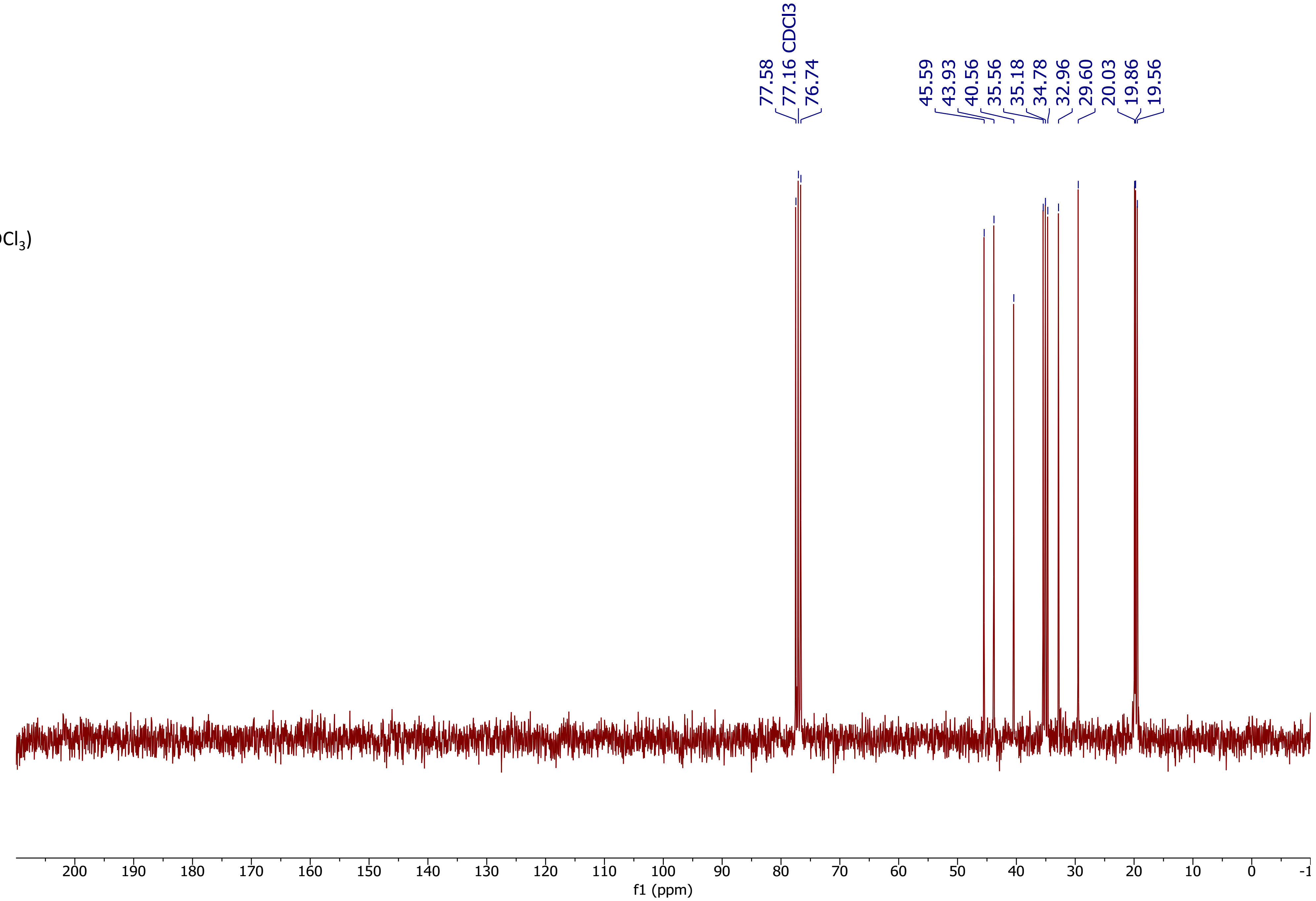

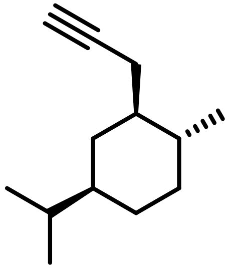

2c-CCH

^{1}H NMR(300 MHz, $CDCl_3$)

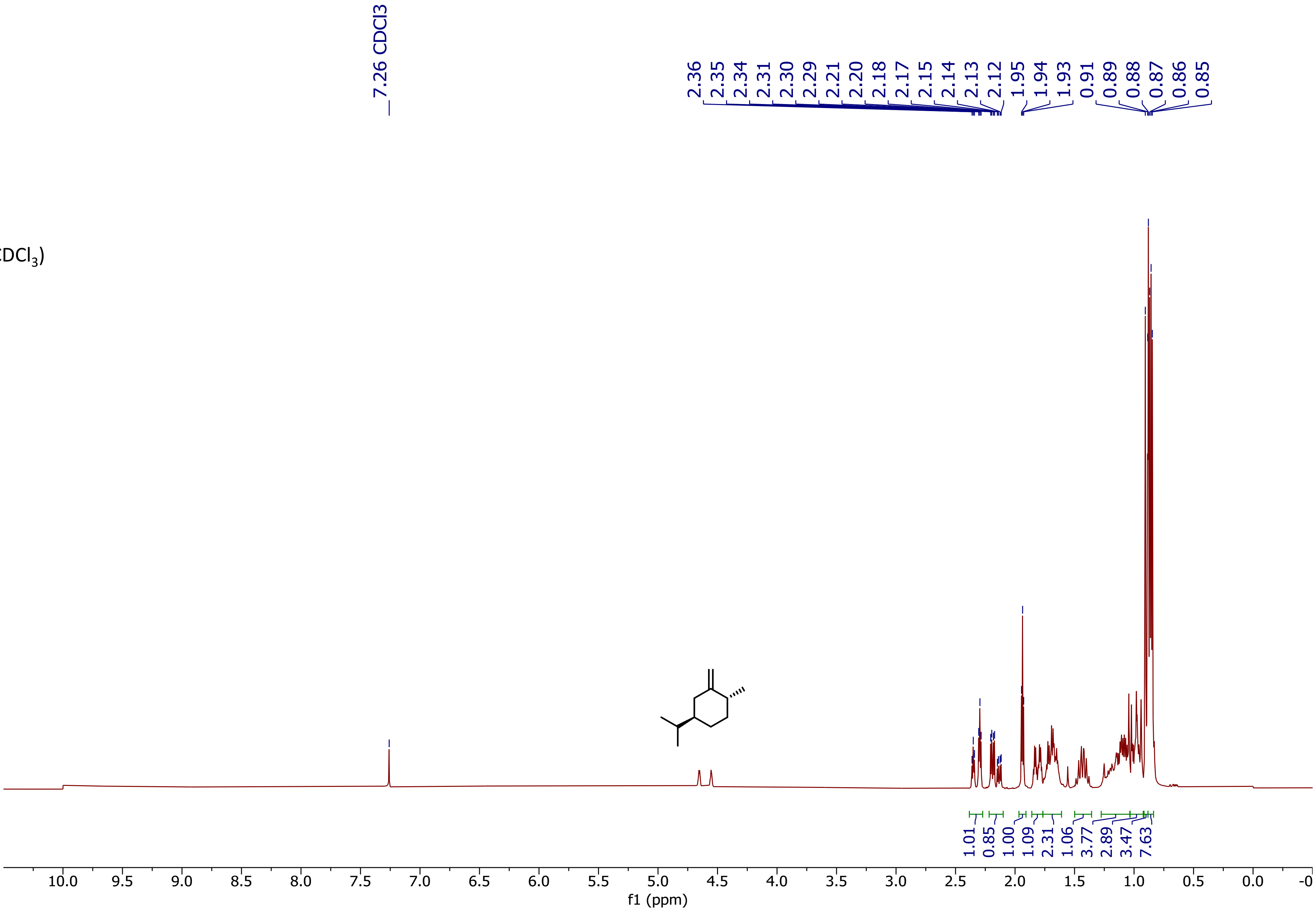

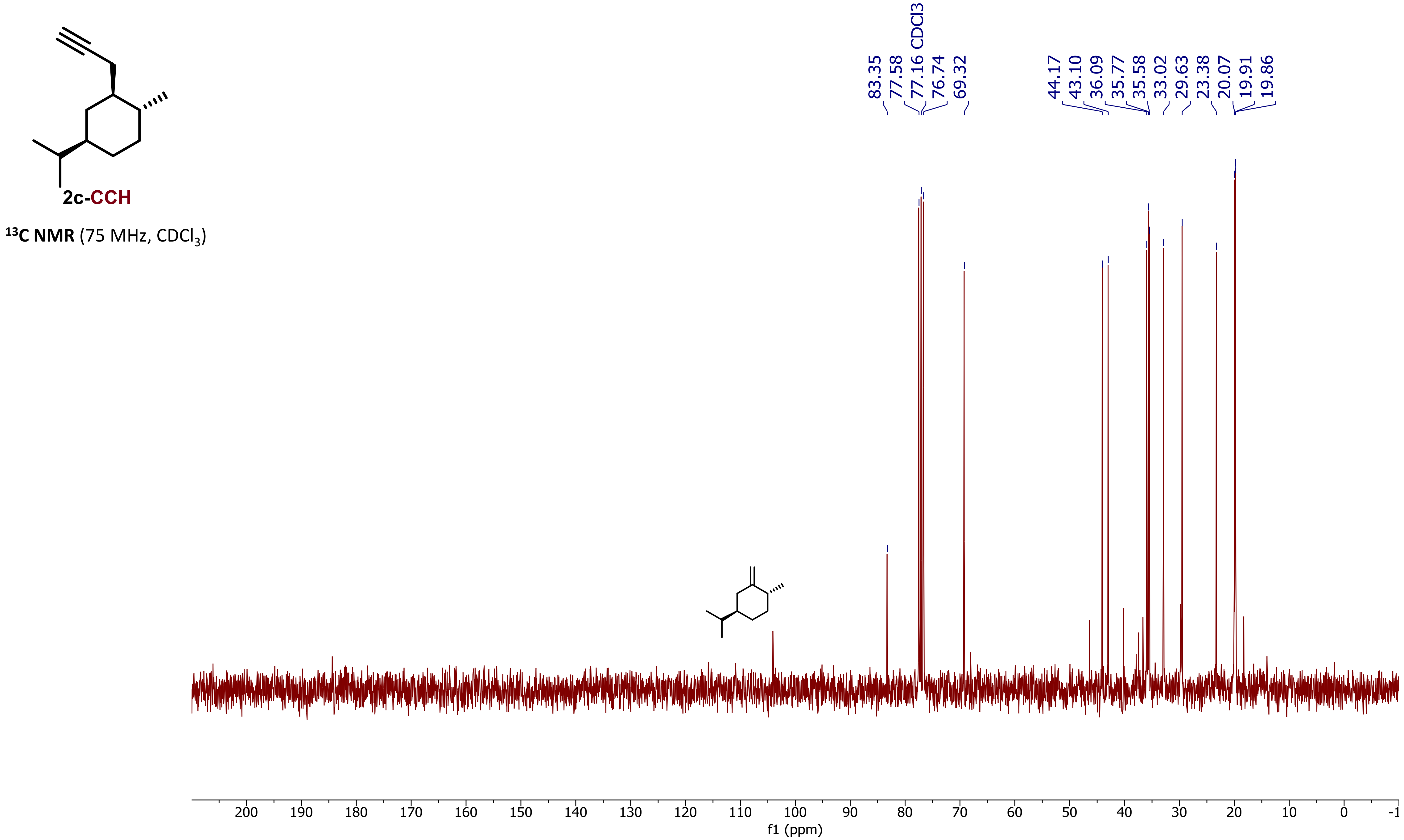
2c-CCH
13C NMR (75 MHz, CDCl3)
83.35
77.58
77.16 CDCl3
76.74
69.32
44.17
43.10
36.09
35.77
35.58
33.02
29.63
23.38
20.07
19.91
19.86
f1 (ppm)

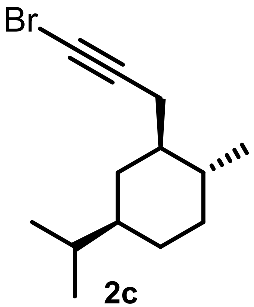

2c

^{1}H NMR(300 MHz, $CDCl_3$)

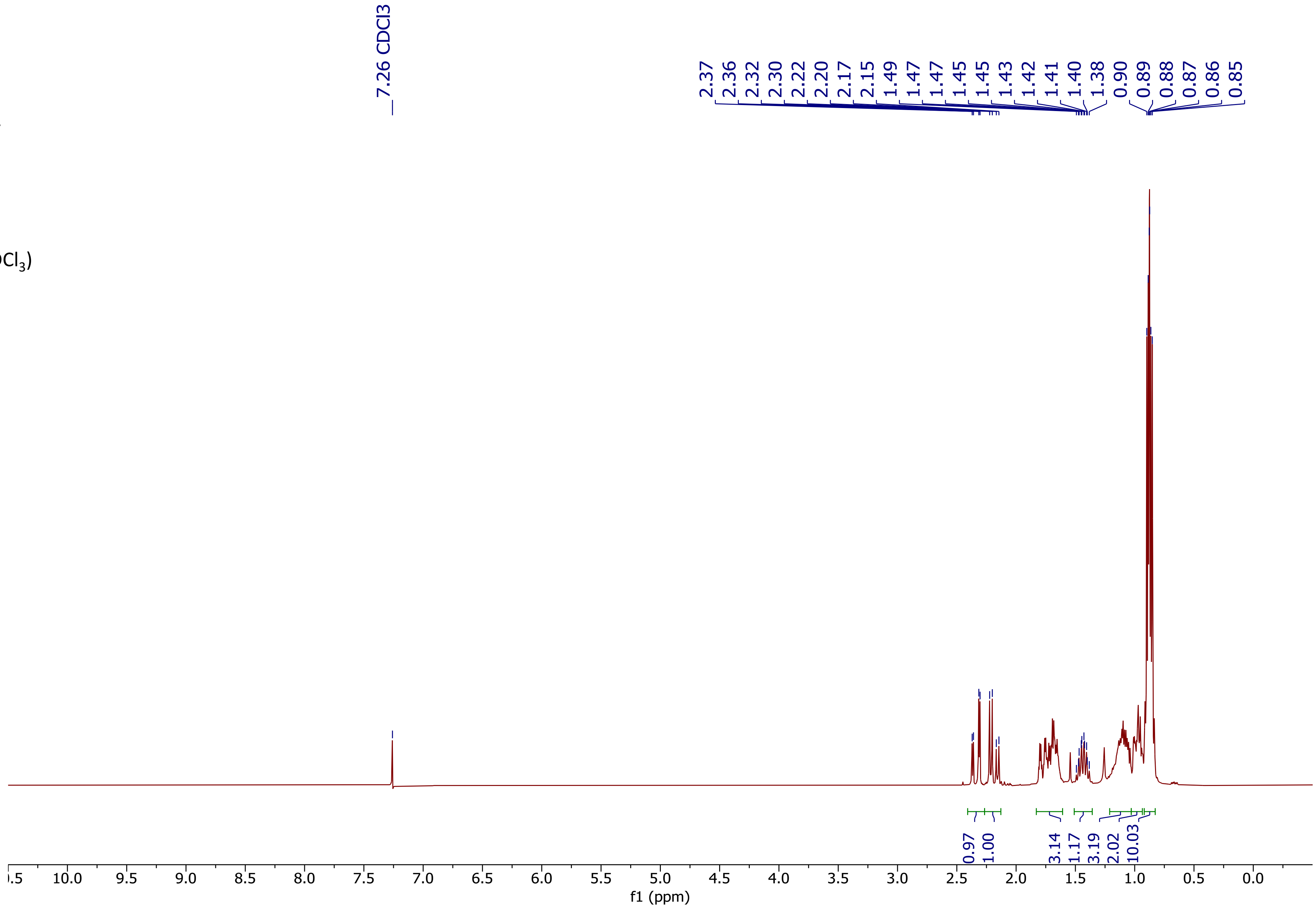

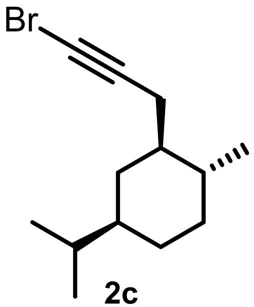

2c

^{13}C NMR (75 MHz, $CDCl_3$)

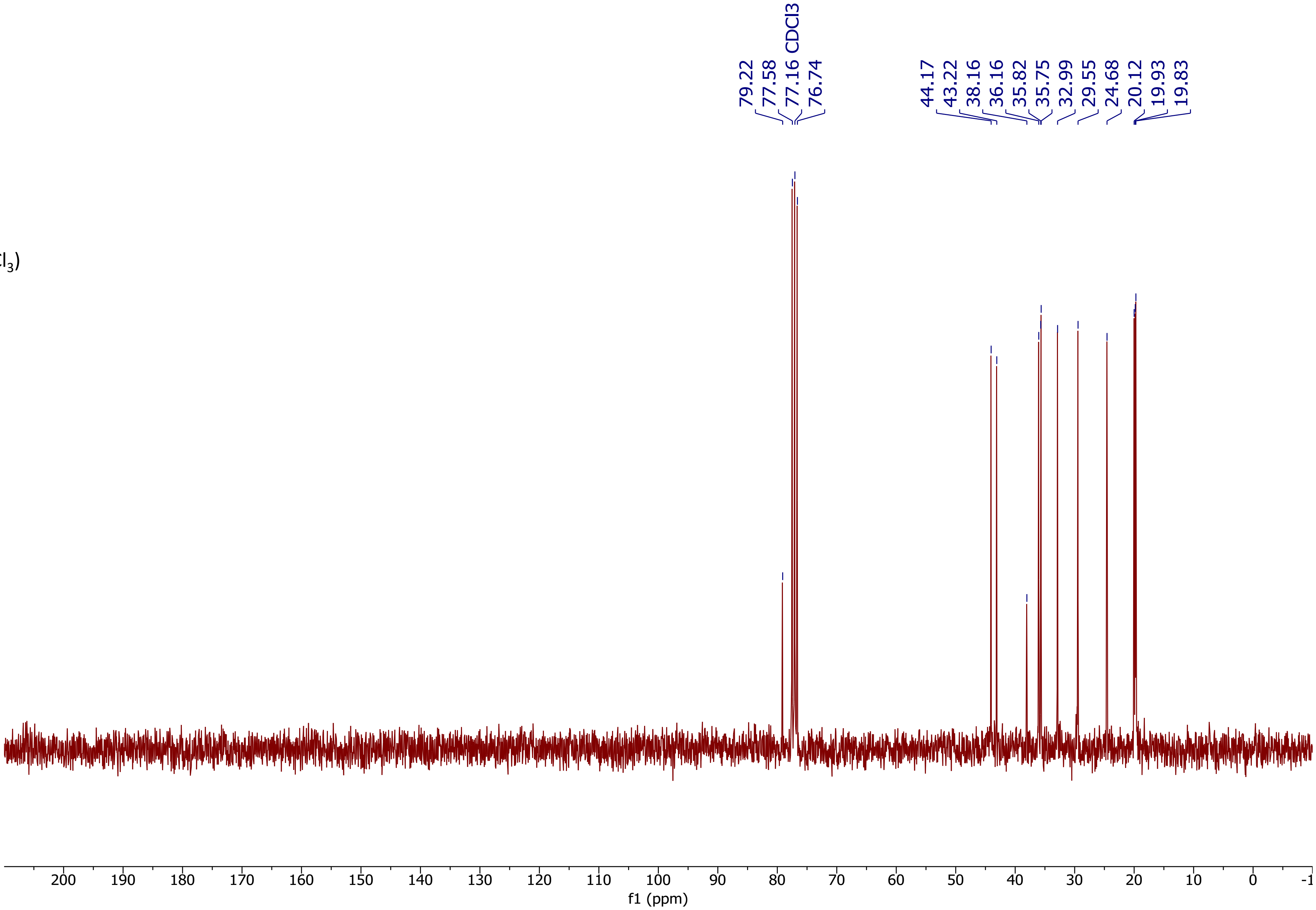

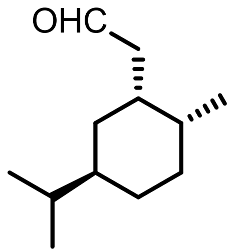

2d-CHO

^{1}H NMR(300 MHz, $CDCl_3$)

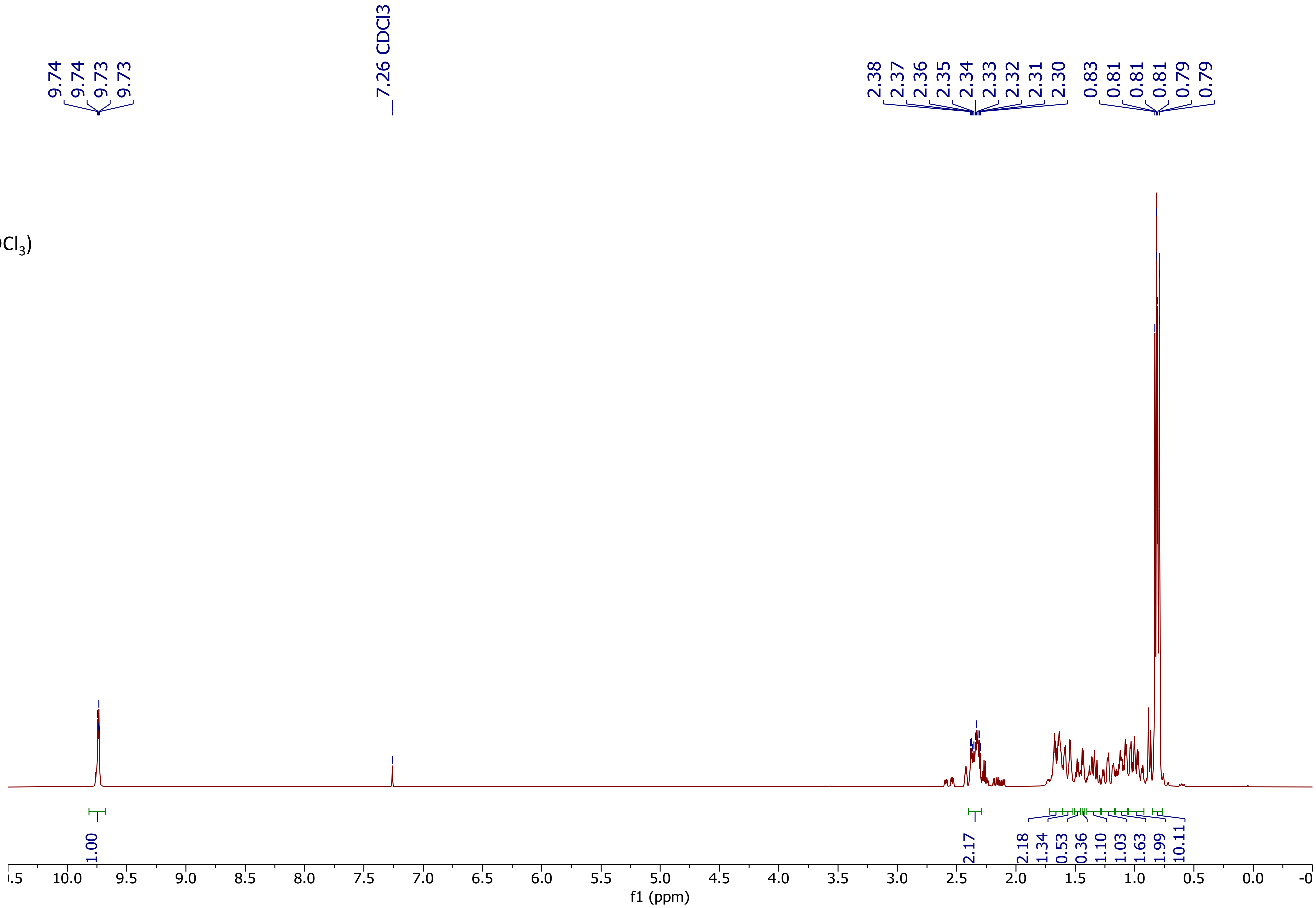

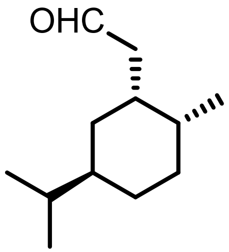

2d-CHO

^{13}C NMR (75 MHz, $CDCl_3$)

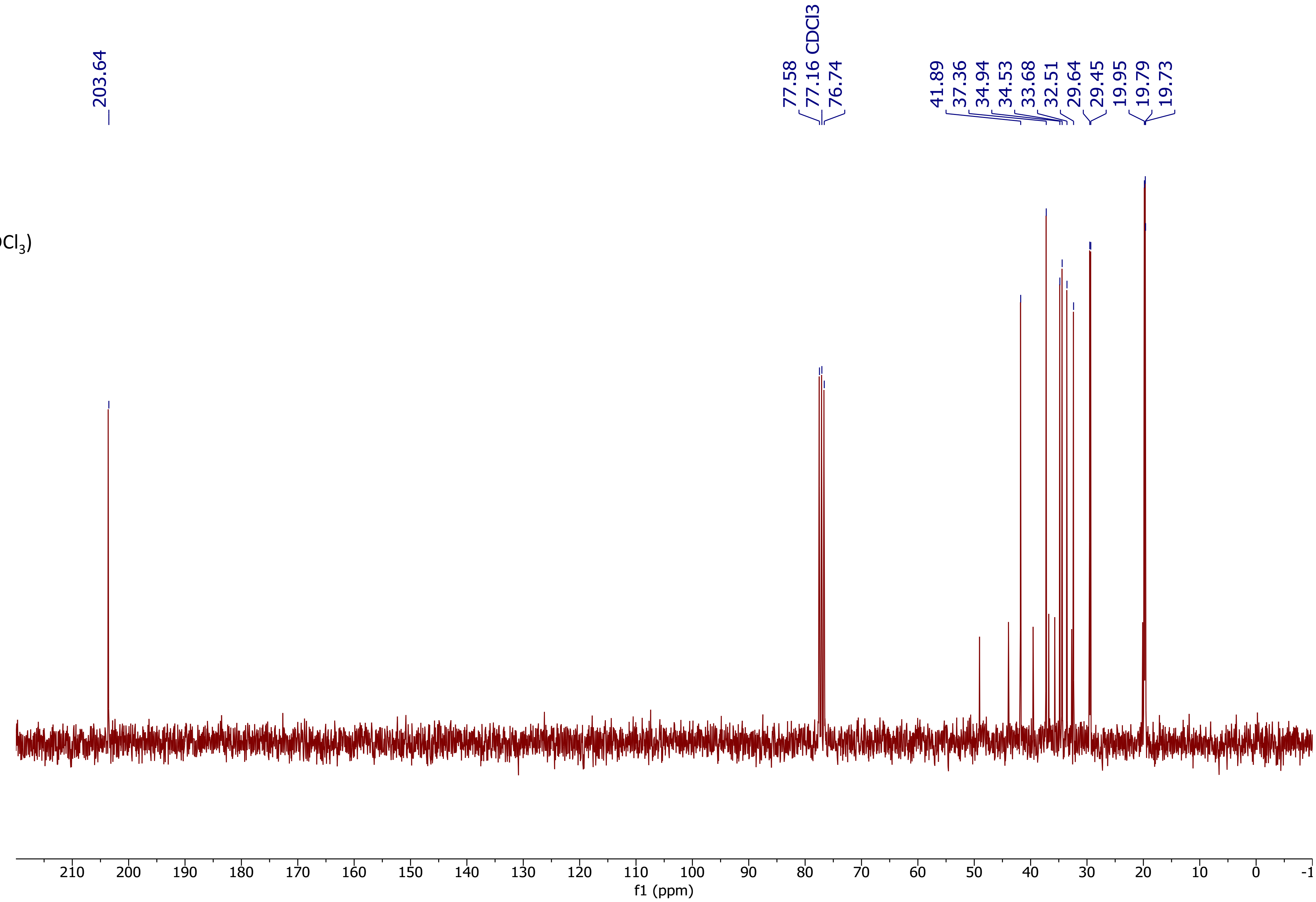

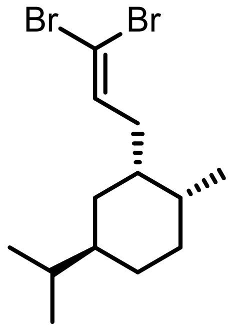

2d-CBr$_2$

^{1}H NMR(300 MHz, $CDCl_3$)

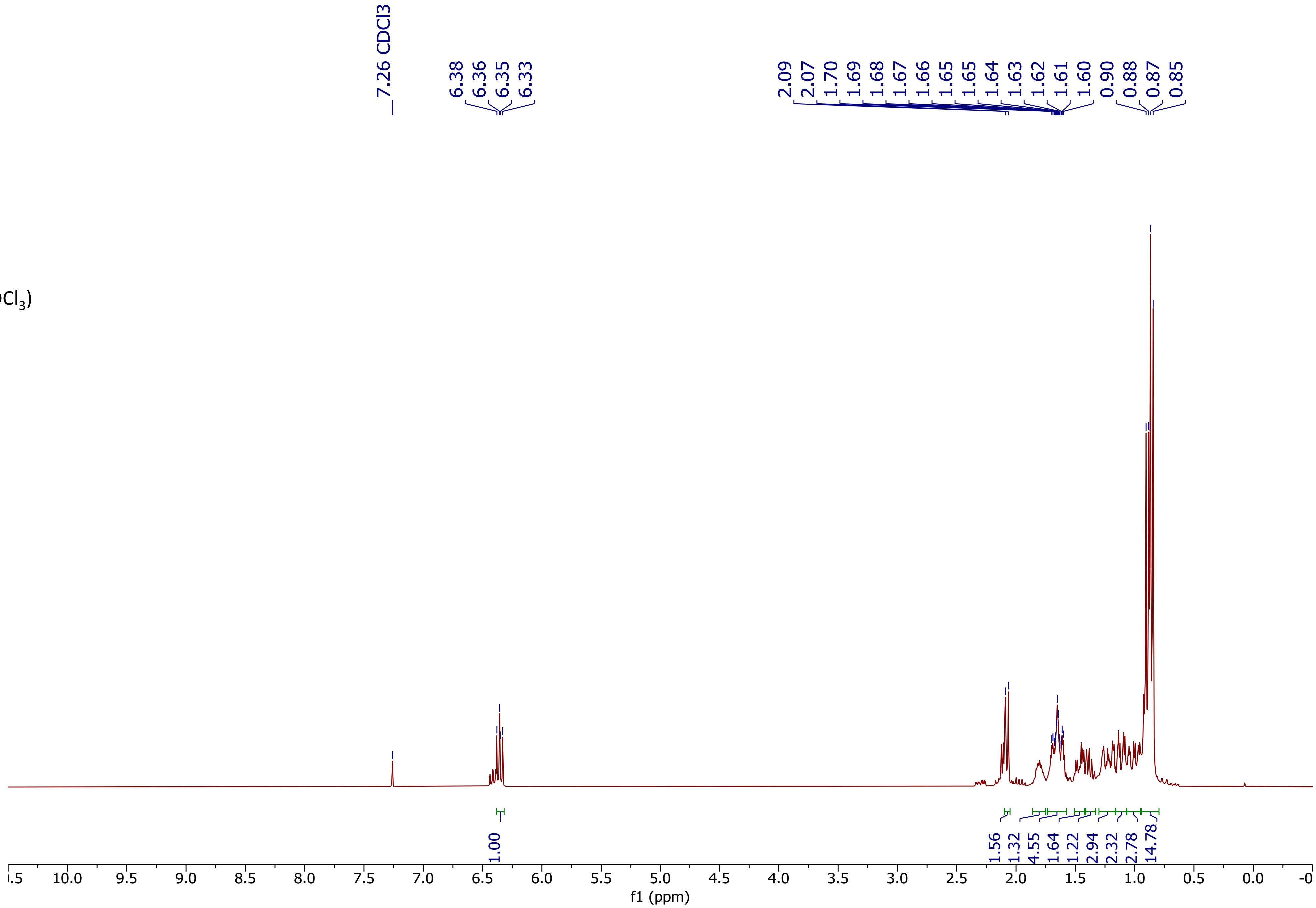

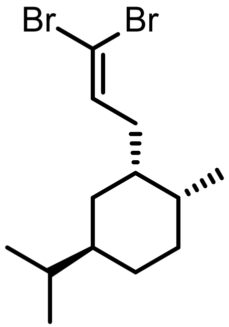

2d-CBr$_2$

^{13}C NMR (75 MHz, $CDCl_3$)

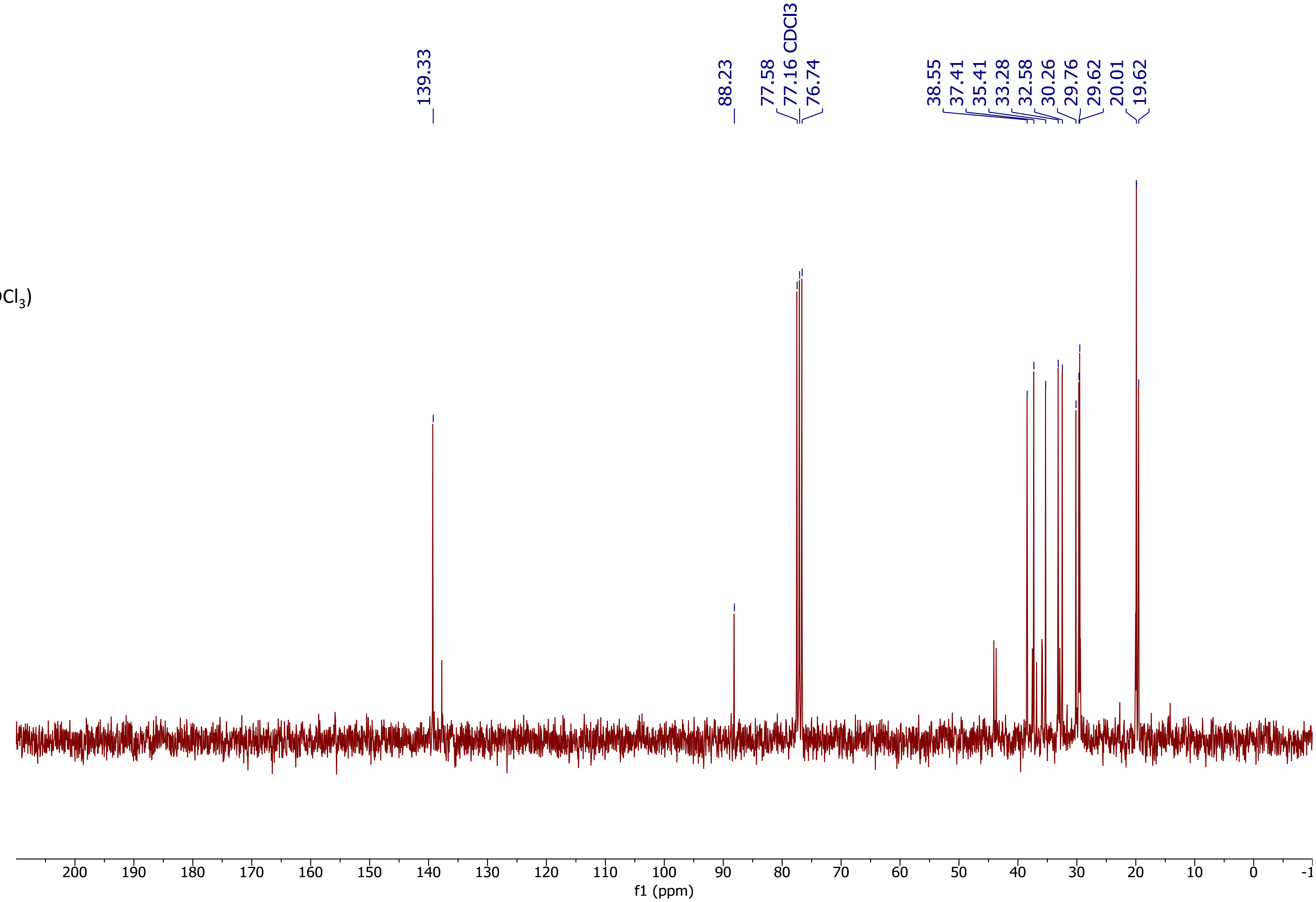

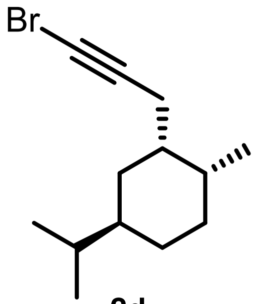

2d

¹H NMR(300 MHz, $CDCl_3$)

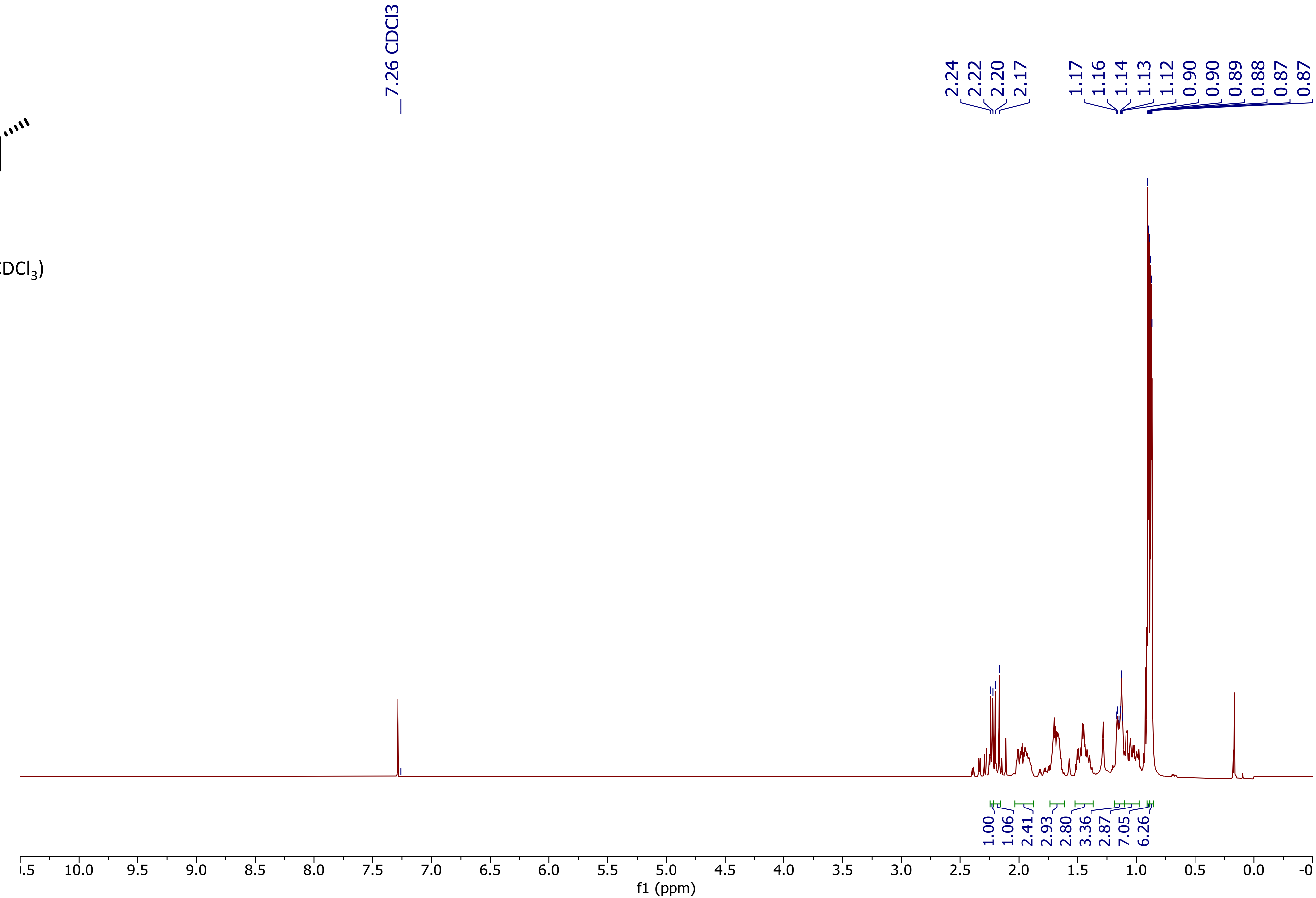

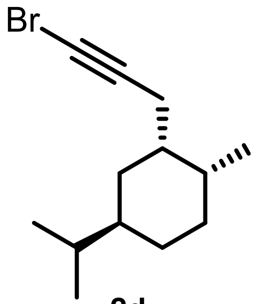

2d

^{13}C NMR (75 MHz, $CDCl_3$)

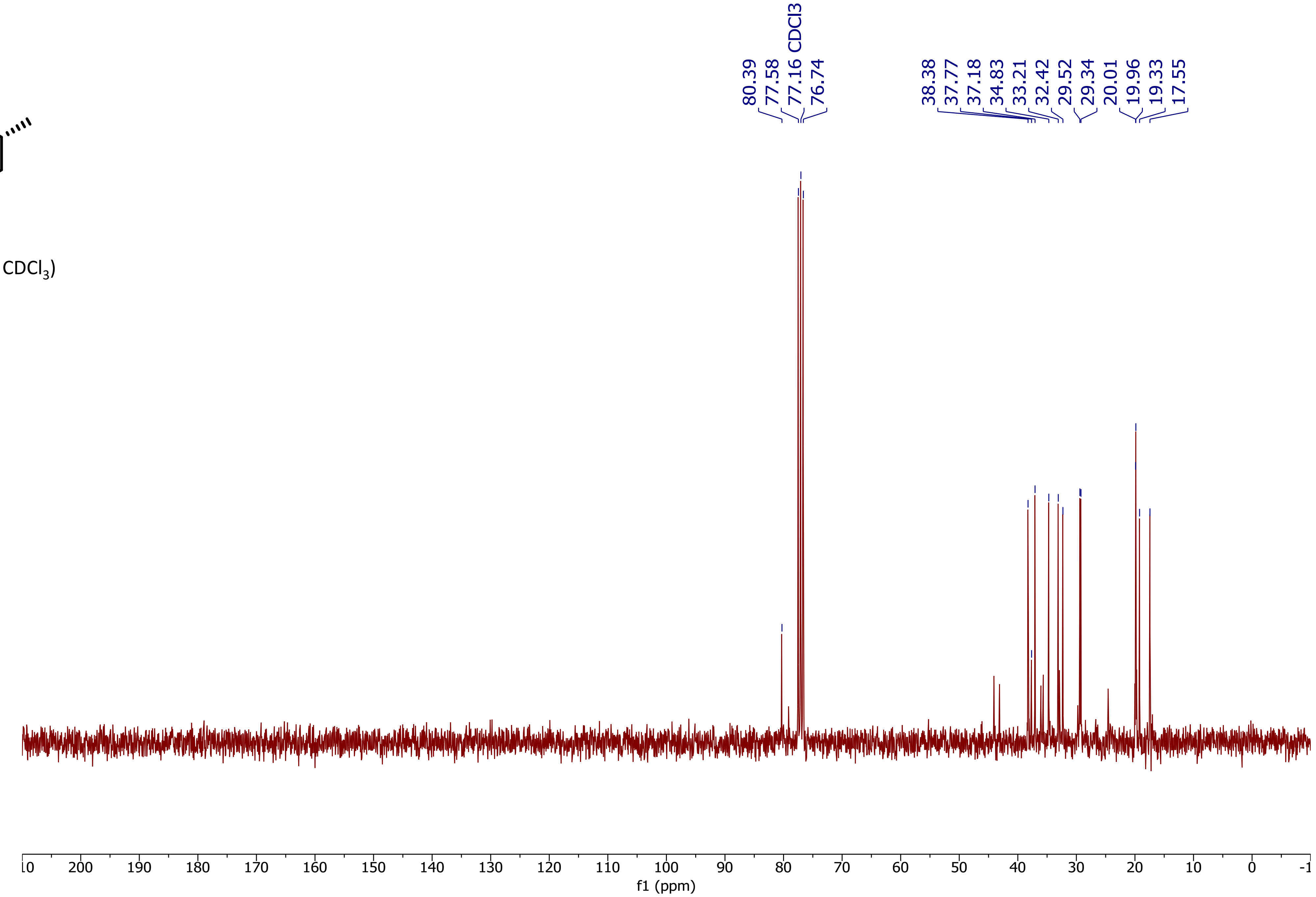

SUBSTRATES FOR THE EXO FAMILY: 2e-g

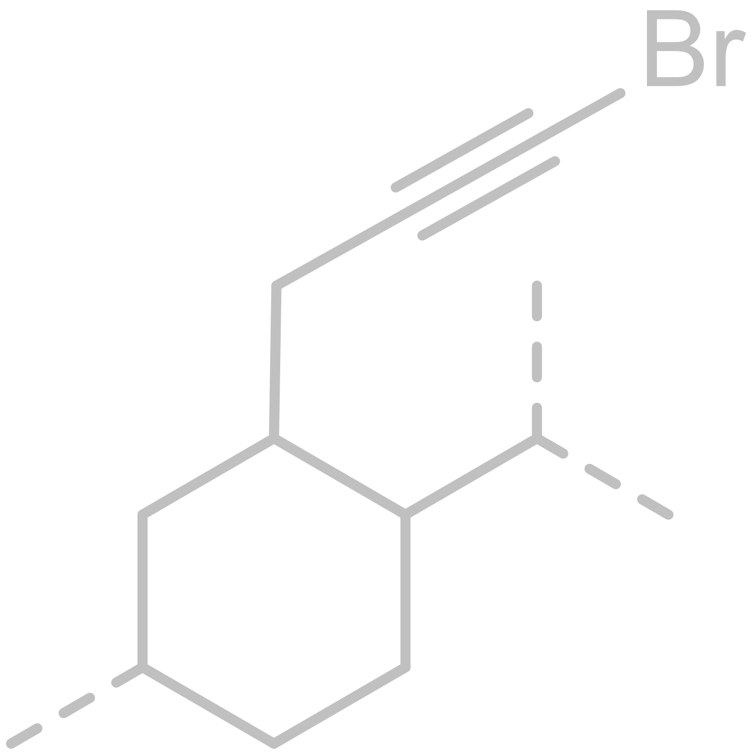

2e-g

2e

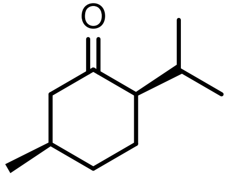

2e-CO

^{1}H NMR(300 MHz, $CDCl_3$)

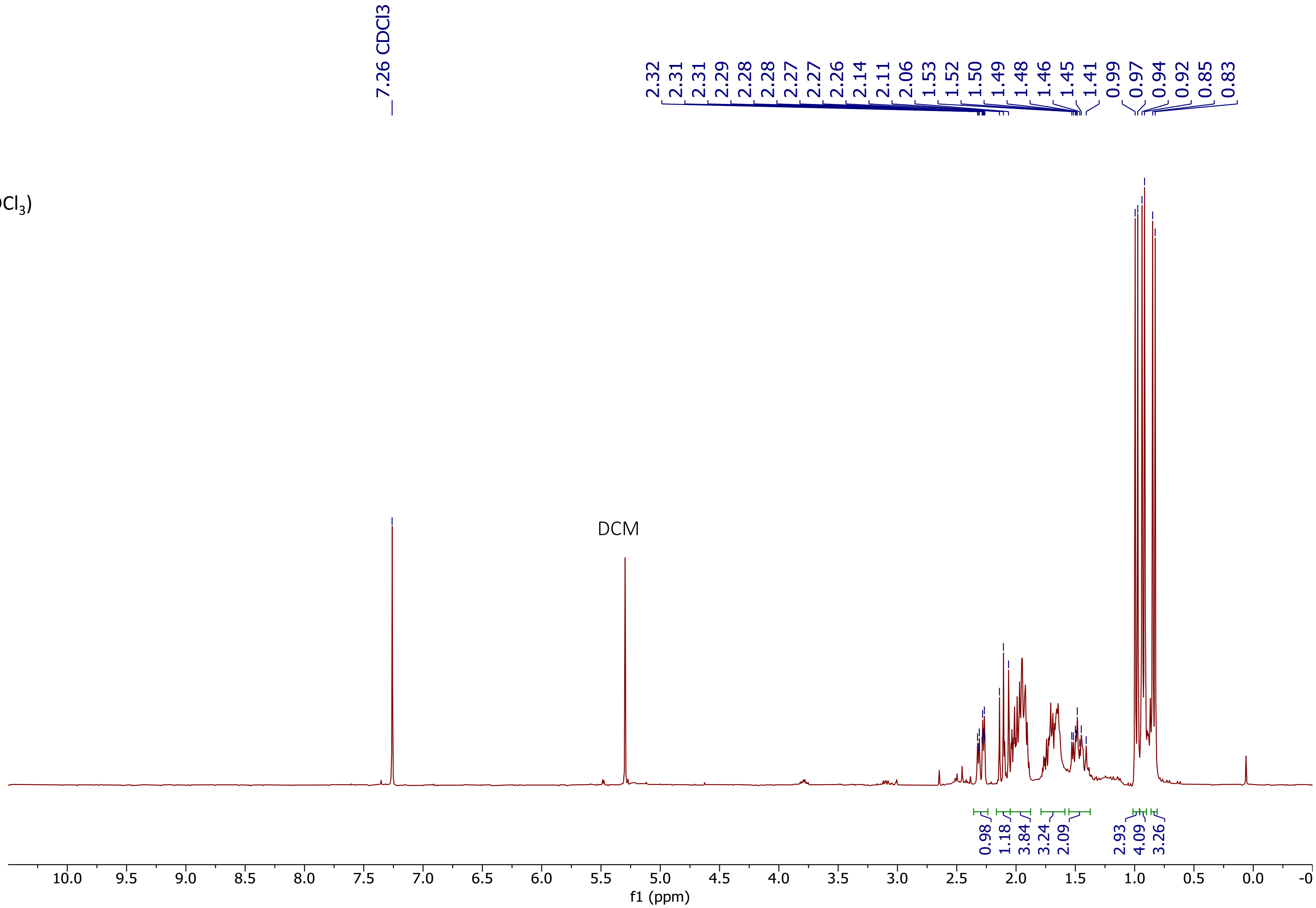

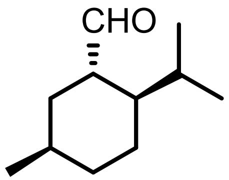

2e-CHO

^{1}H NMR(300 MHz, $CDCl_3$)

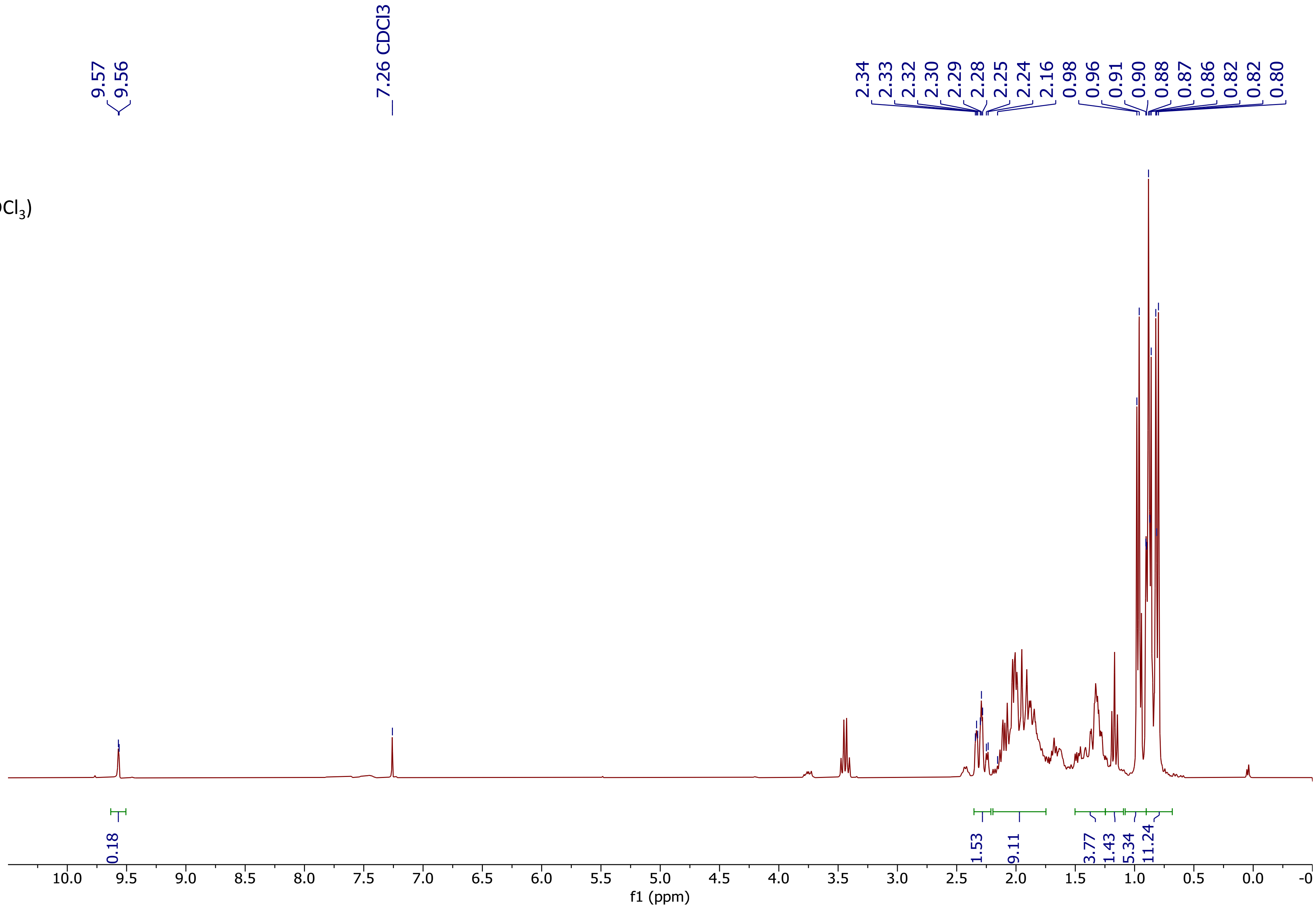

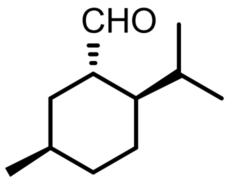

2e-CHO

^{13}C NMR (75 MHz, $CDCl_3$)

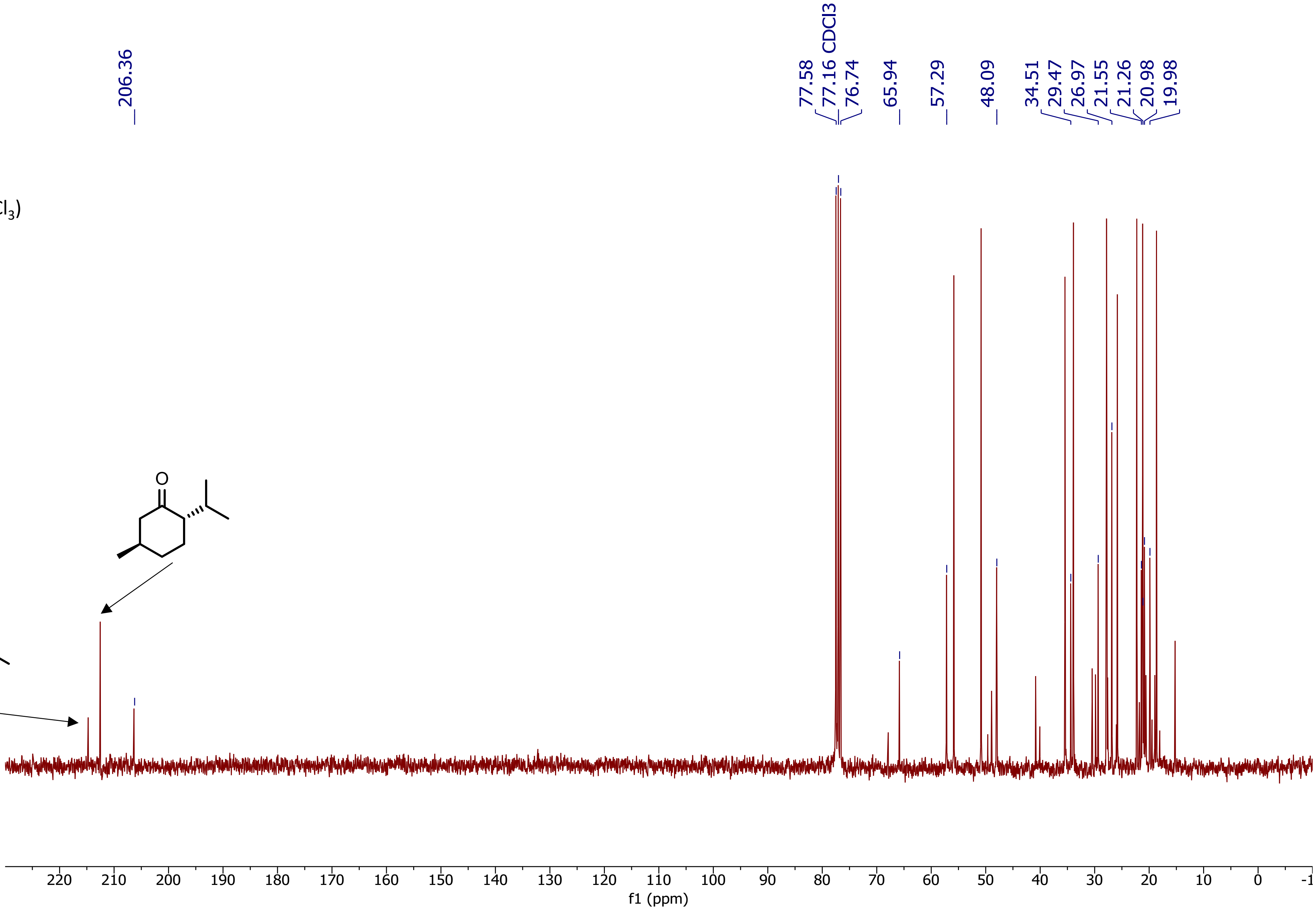

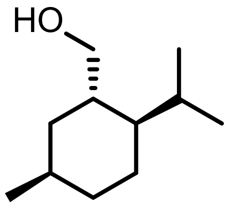

2e-OH

-crude-

^{1}H NMR(300 MHz, $CDCl_3$)

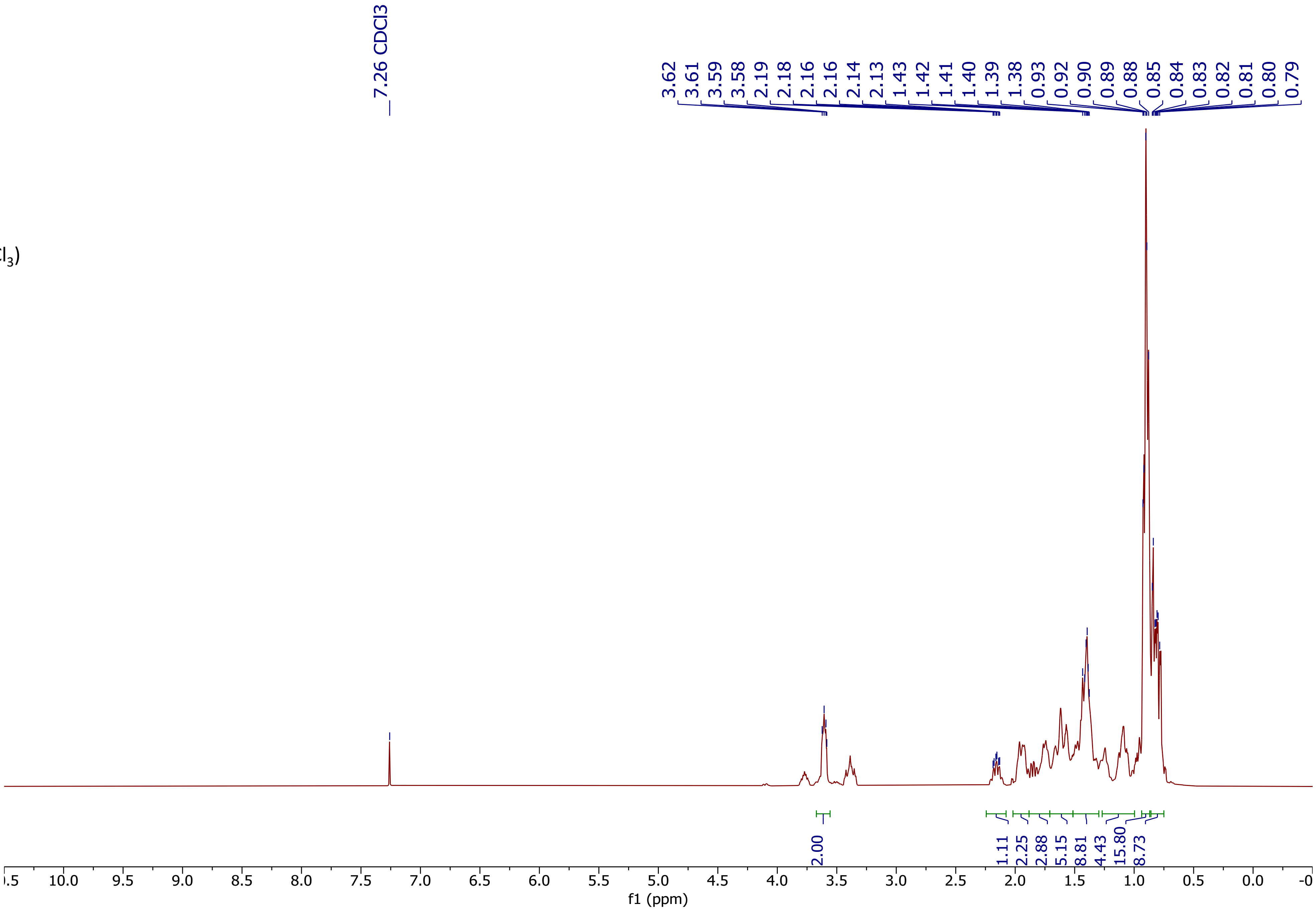

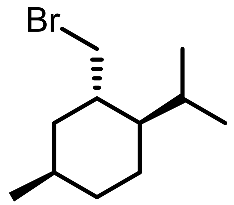

2e-Br

^{1}H NMR(300 MHz, $CDCl_3$)

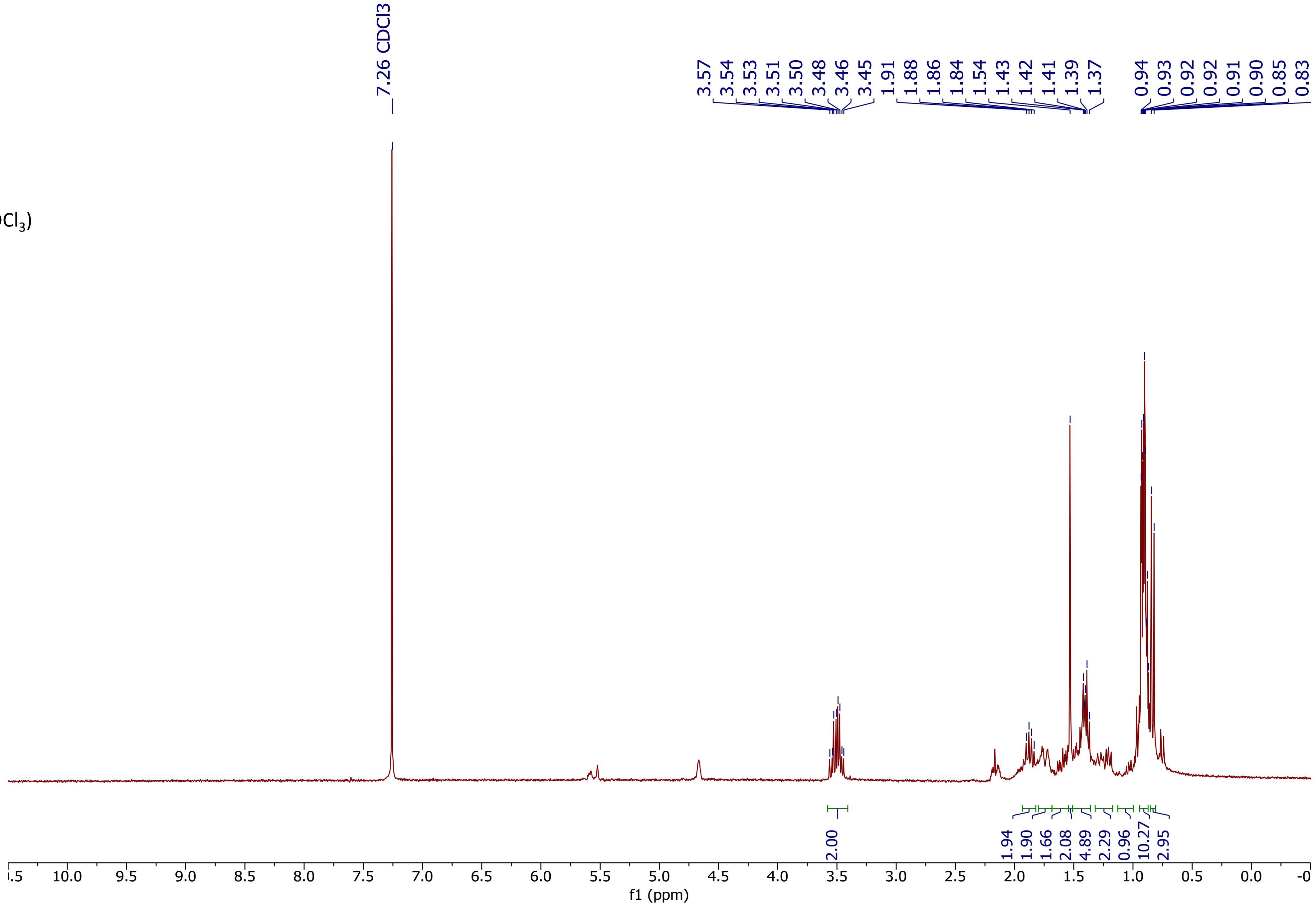

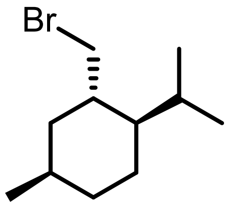

2e-Br

^{13}C NMR (75 MHz, $CDCl_3$)

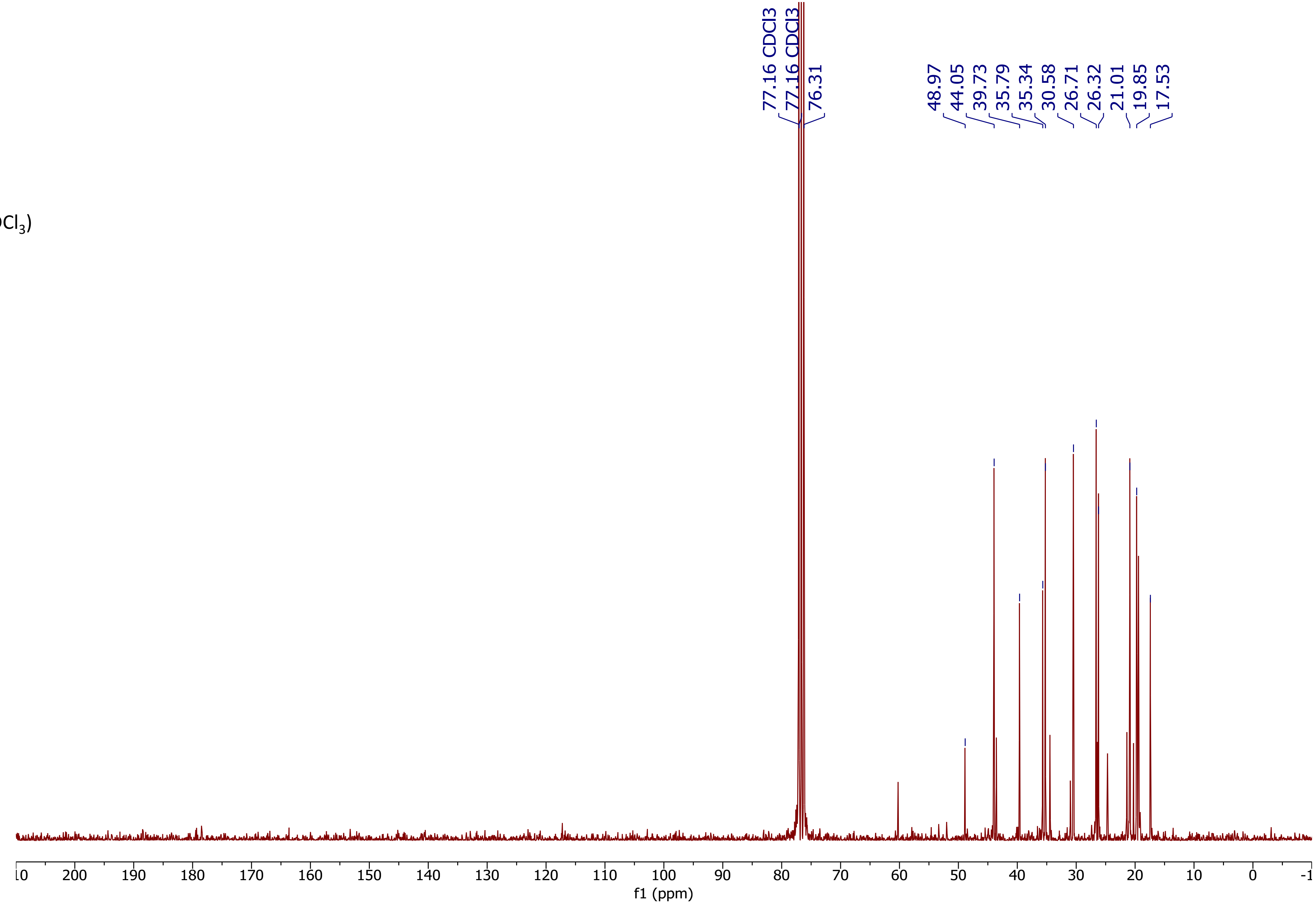

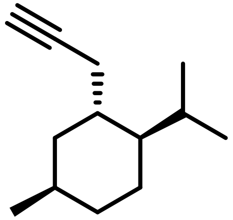

2e-CCH

^{1}H NMR(300 MHz, $CDCl_3$)

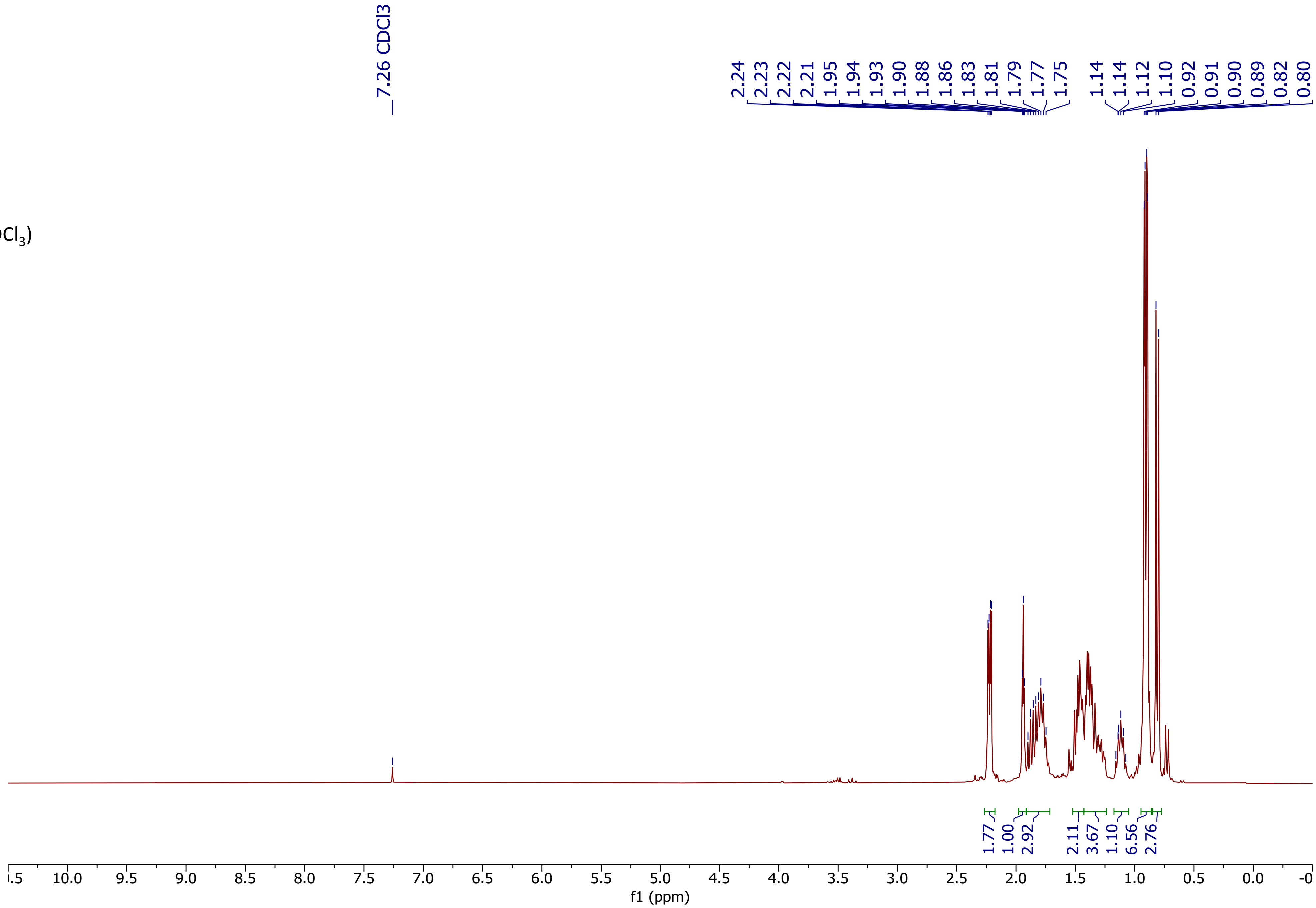

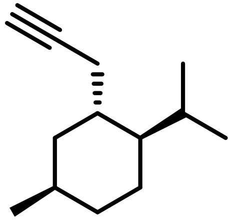

2e-CCH

^{13}C NMR (75 MHz, $CDCl_3$)

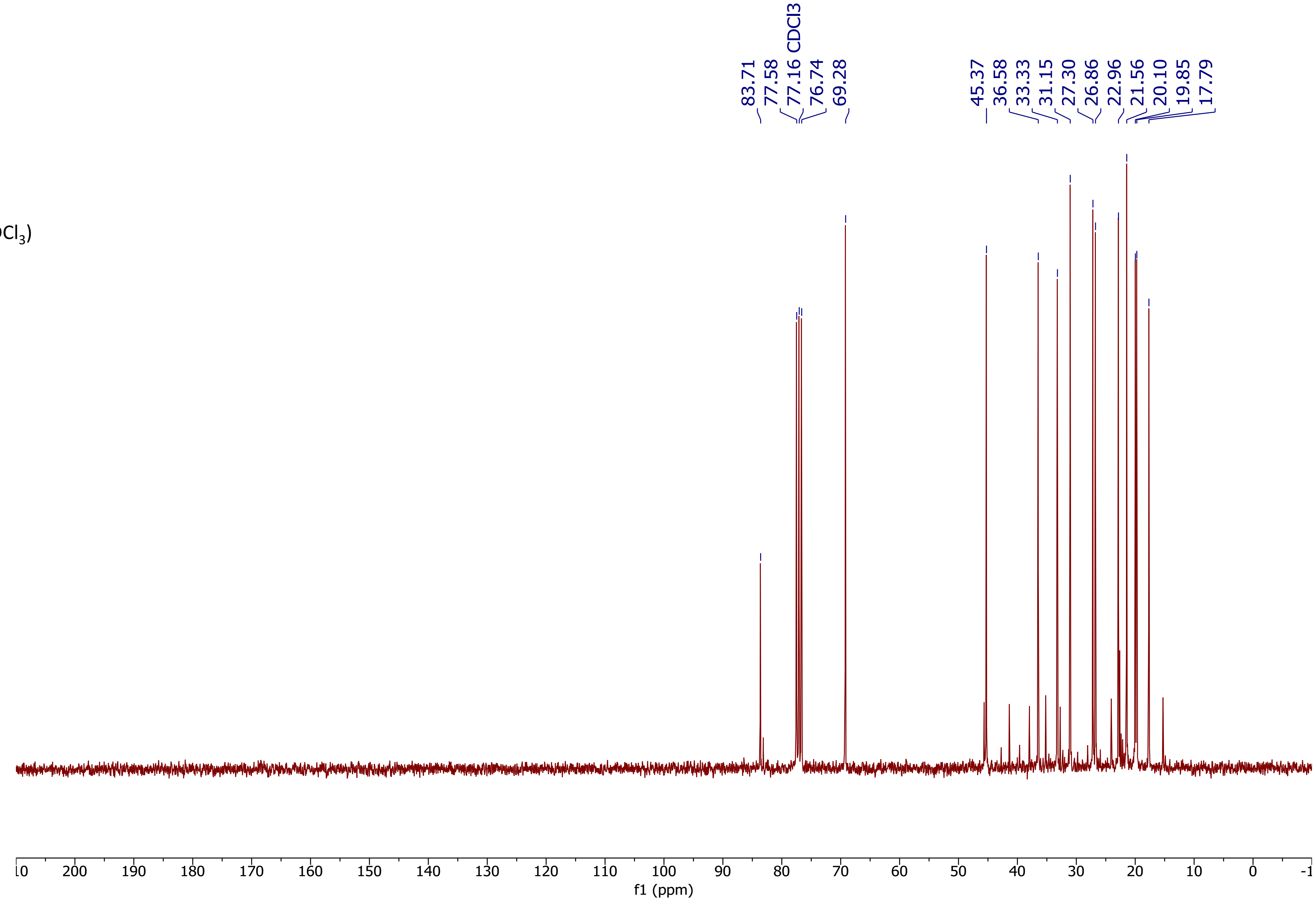

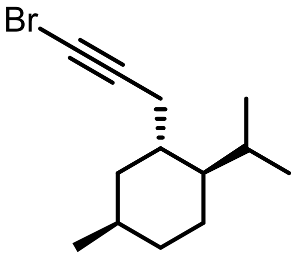

2e

^{1}H NMR(300 MHz, $CDCl_3$)

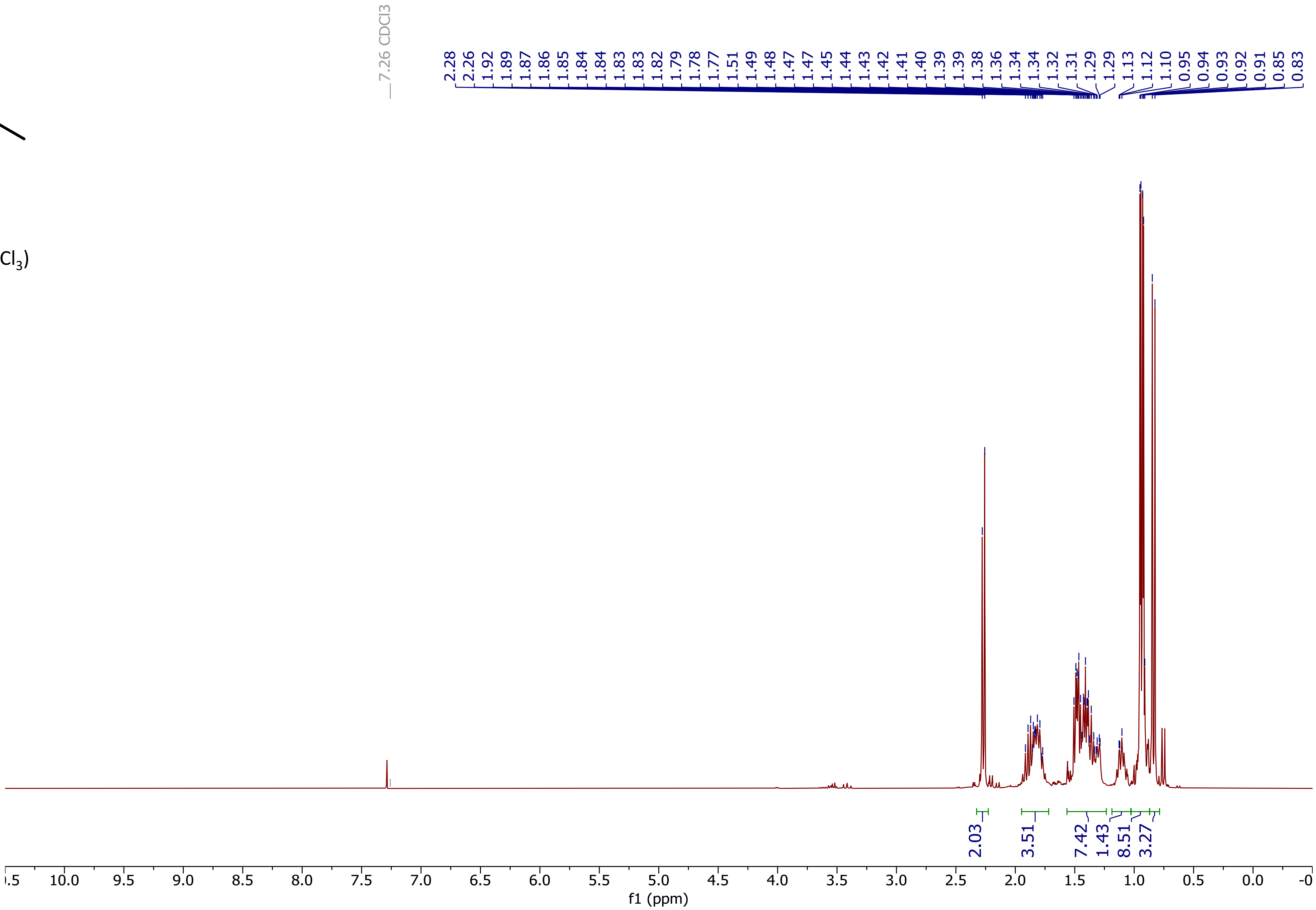

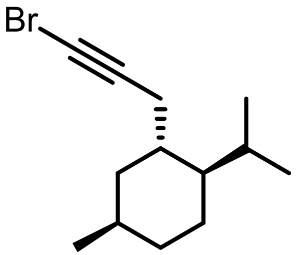

2e

^{13}C NMR (75 MHz, $CDCl_3$)

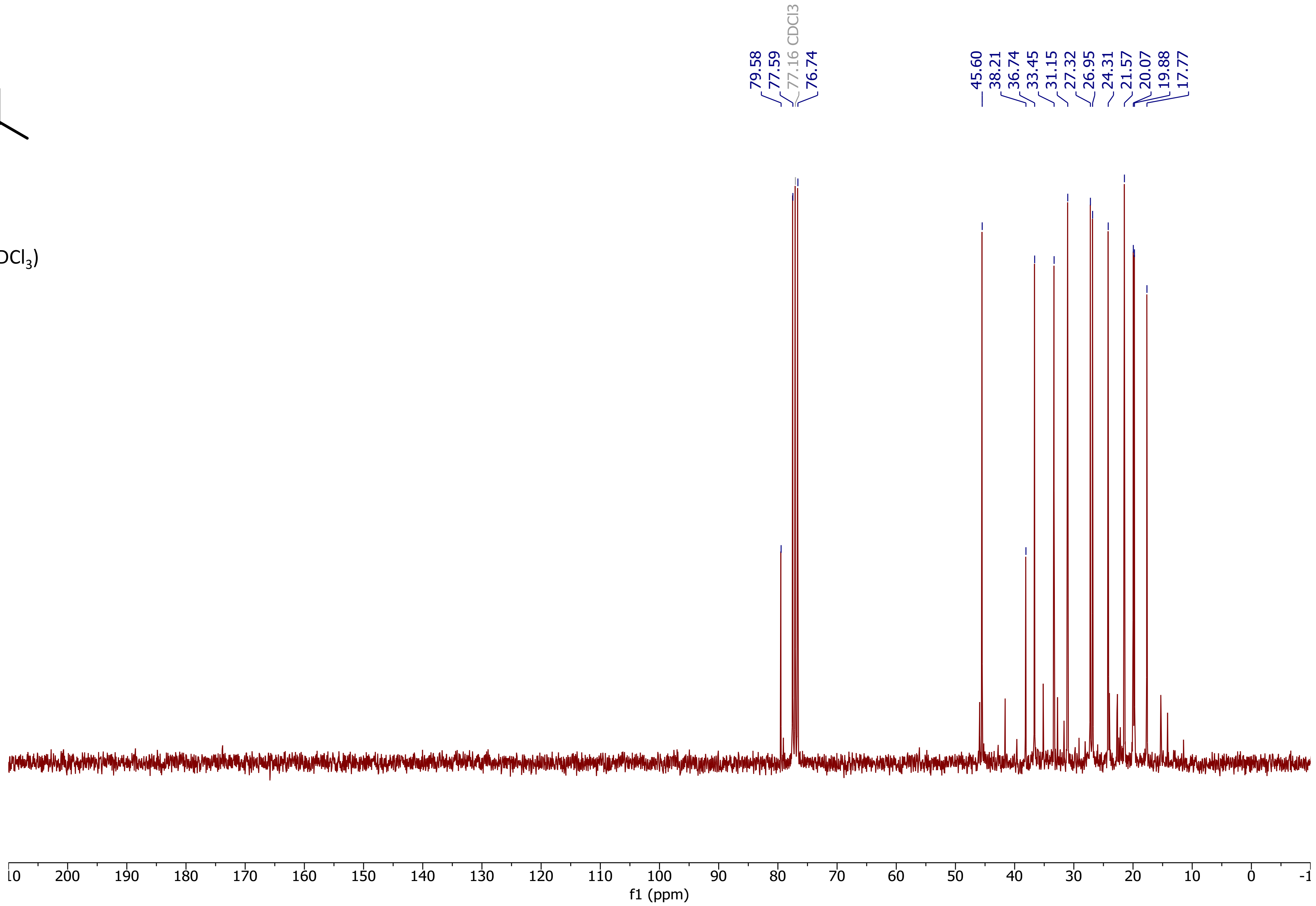

2f

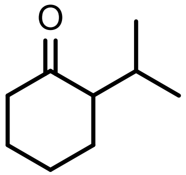

2f-CO

^{1}H NMR(300 MHz, $CDCl_3$)

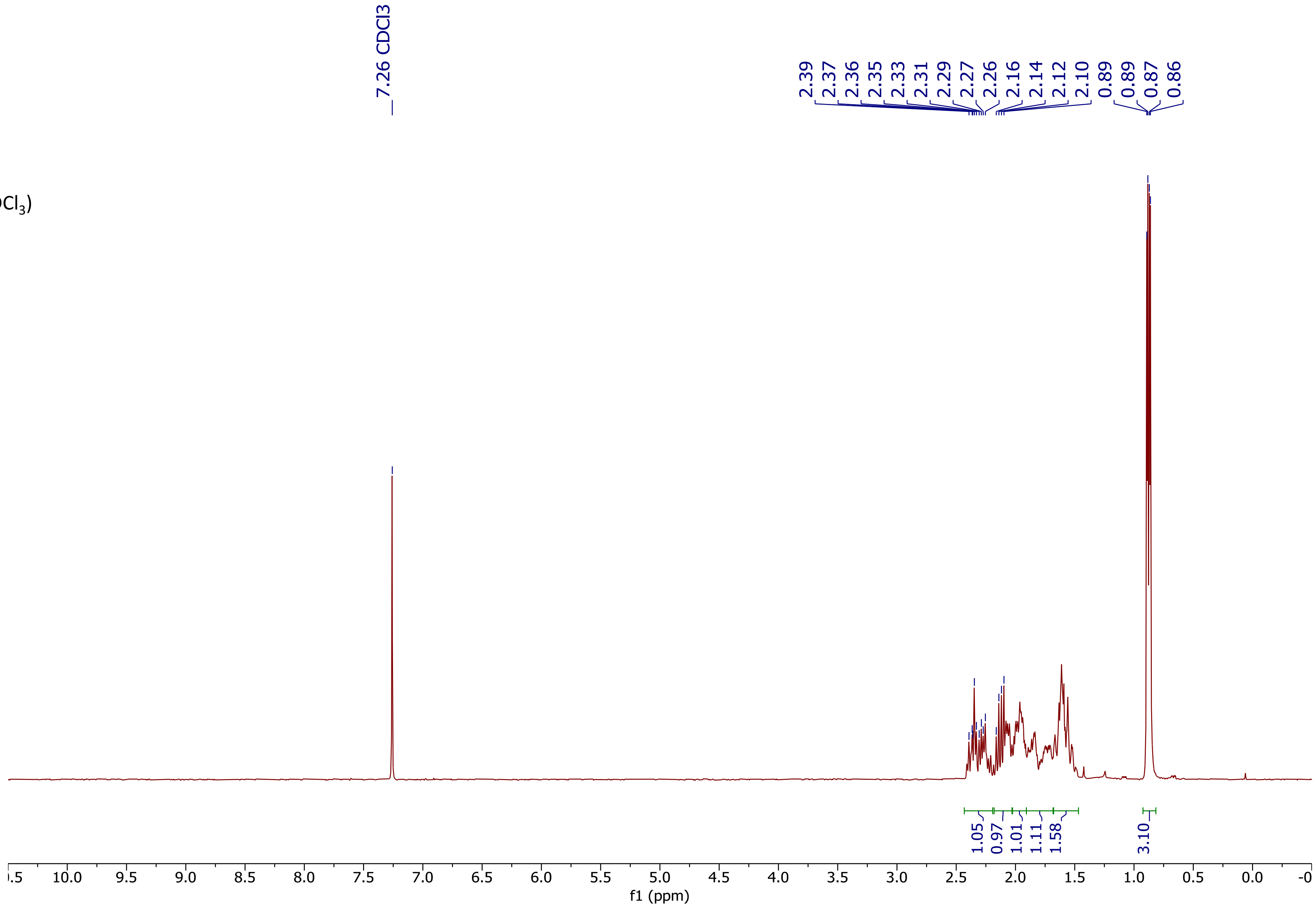

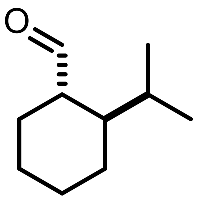

2f-CHO

-crude-

^{1}H NMR(300 MHz, $CDCl_3$)

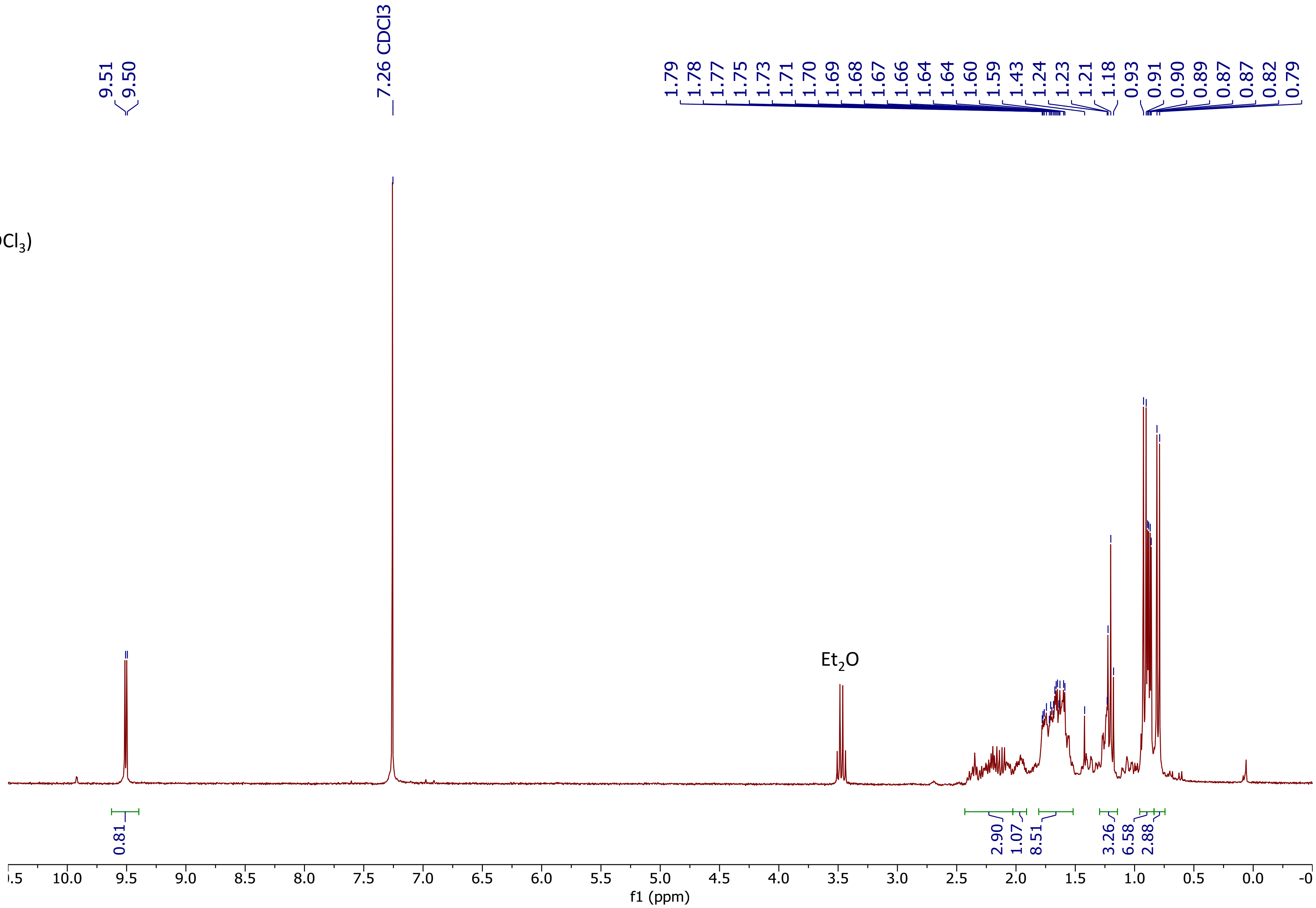

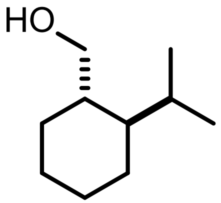

2f-OH

-crude-

^{1}H NMR(300 MHz, $CDCl_3$)

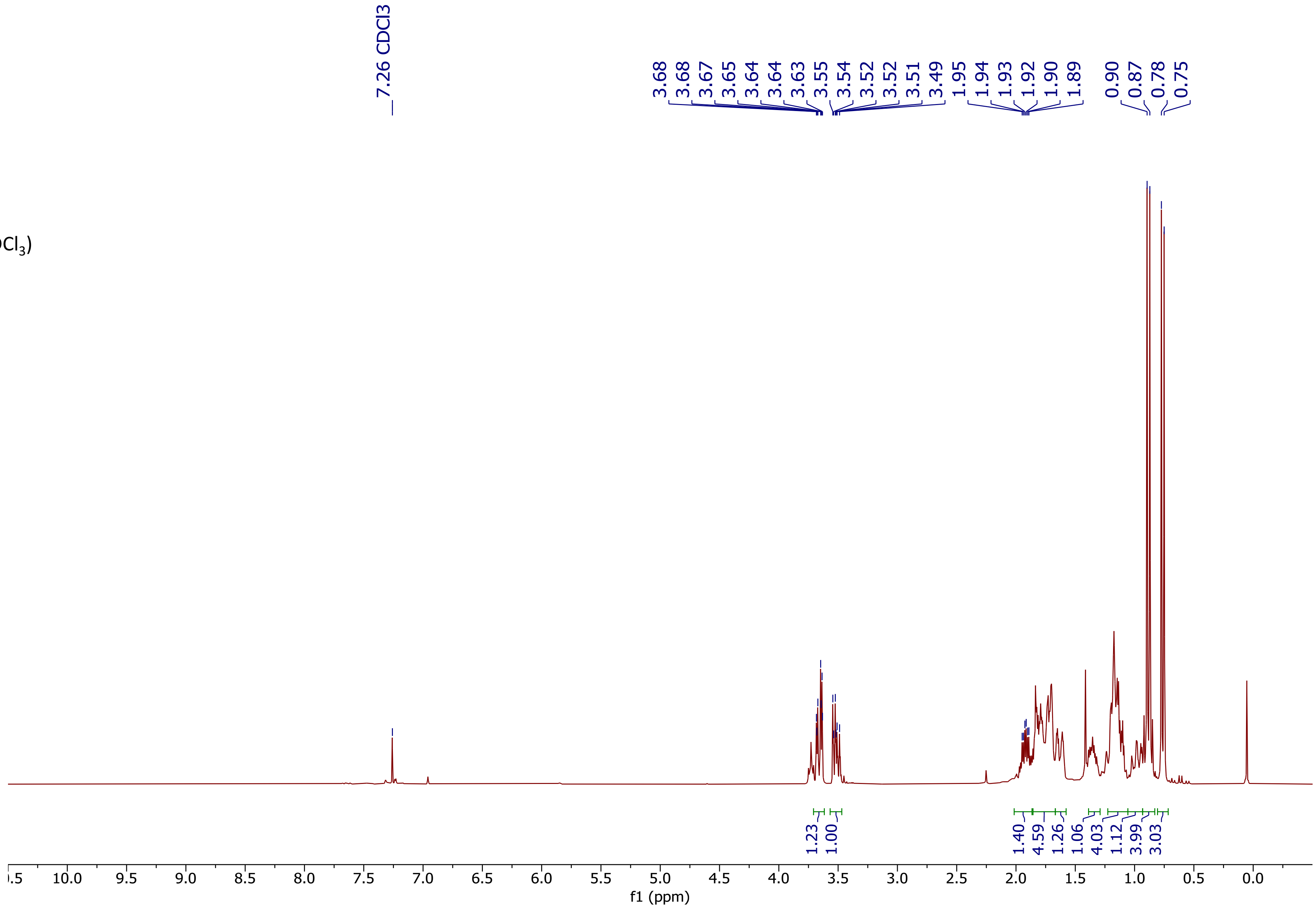

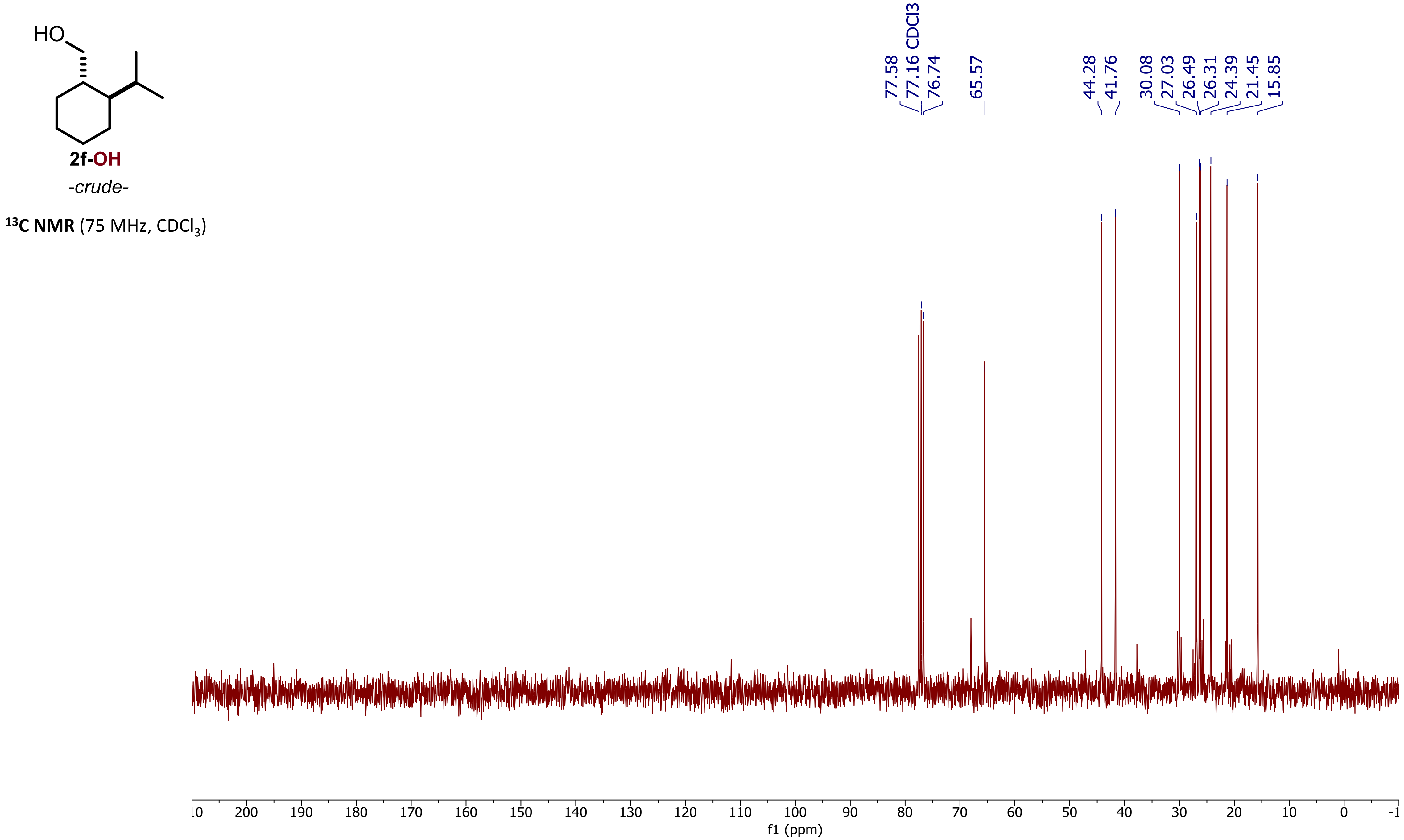
HO
2f-OH
-crude-
13C NMR (75 MHz, CDCl3)
77.58
77.16 CDCl3
76.74
65.57
44.28
41.76
30.08
27.03
26.49
26.31
24.39
21.45
15.85
f1 (ppm)

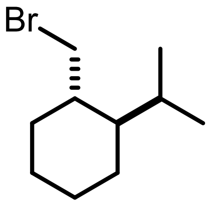

2f-Br

-crude-

^{1}H NMR(300 MHz, $CDCl_3$)

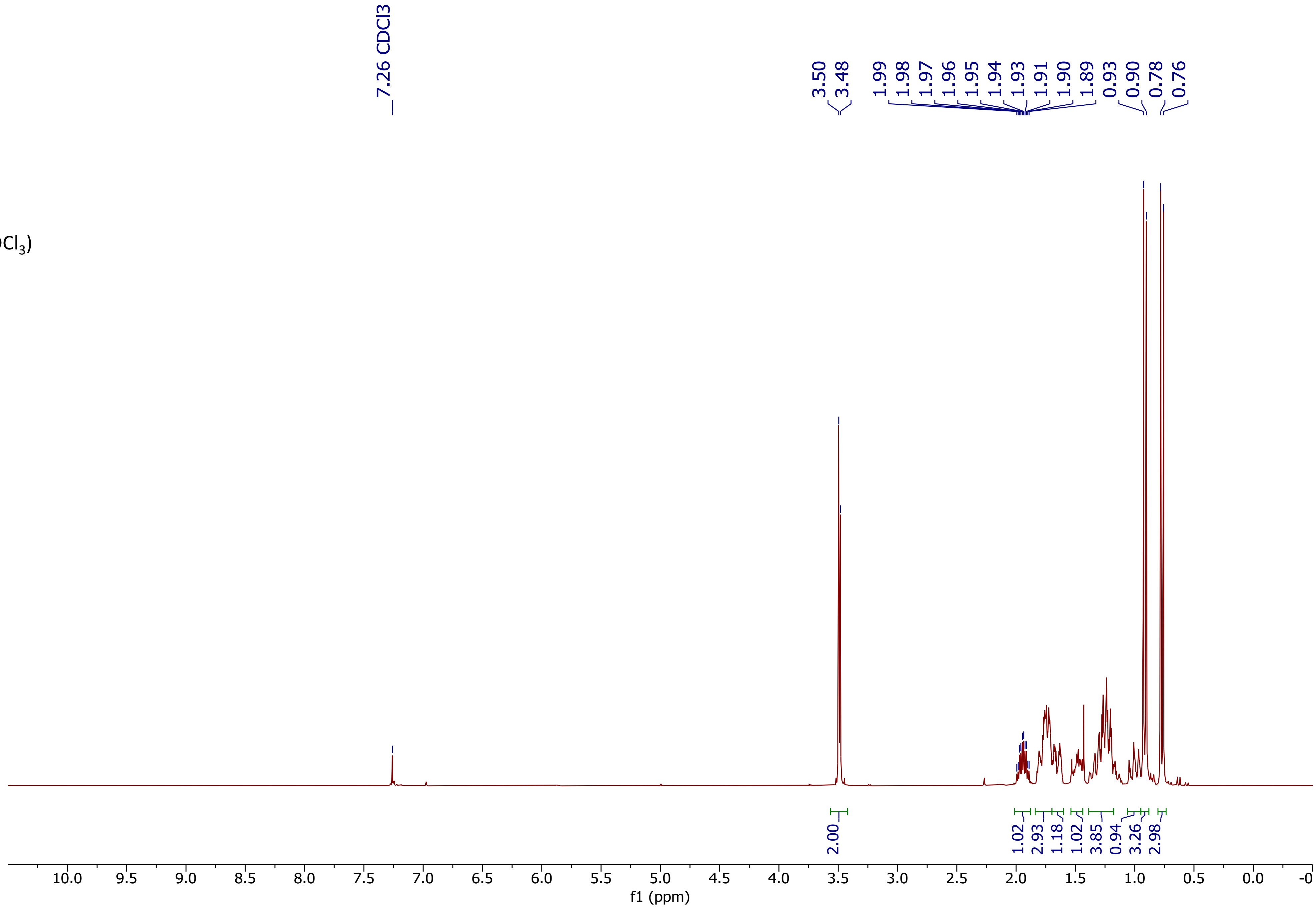

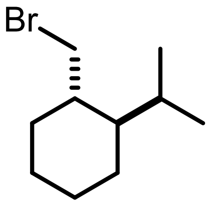

2f-Br

-crude-

^{13}C NMR (75 MHz, $CDCl_3$)

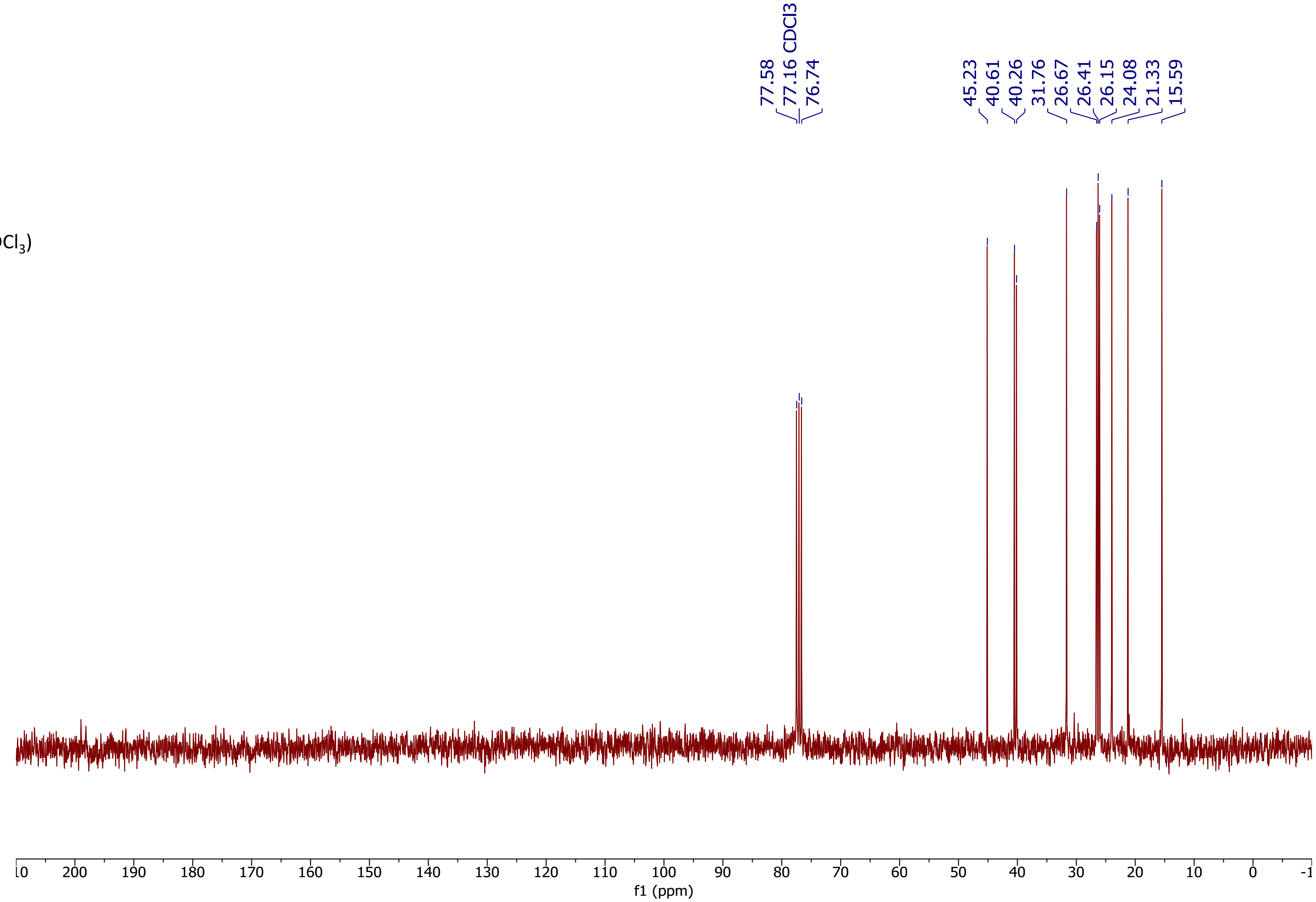

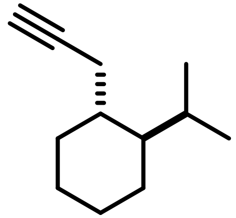

2f-CCH

^{1}H NMR(300 MHz, $CDCl_3$)

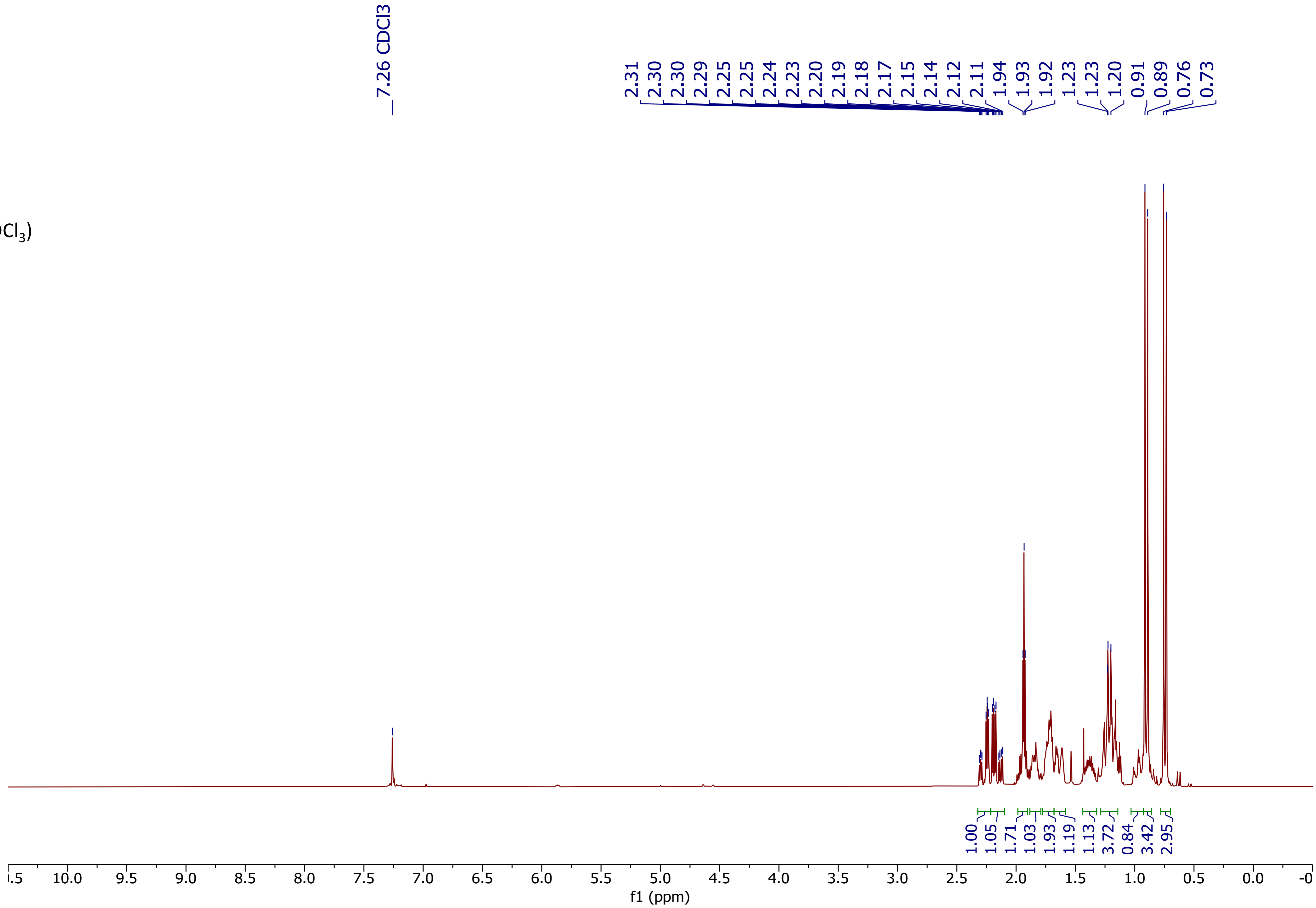

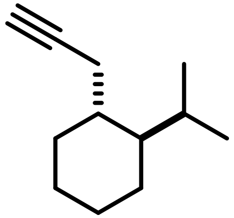

2f-CCH

^{13}C NMR (75 MHz, $CDCl_3$)

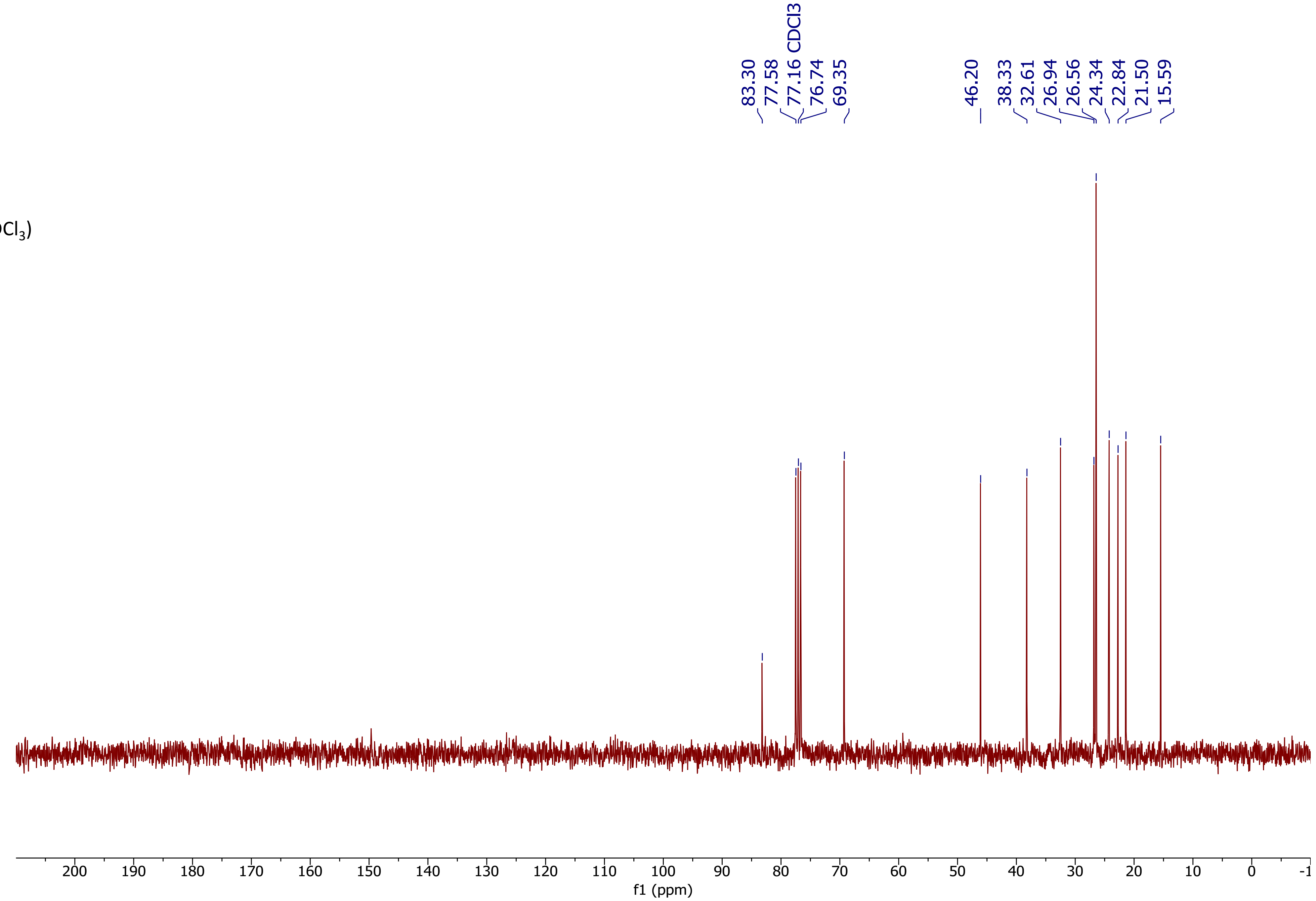

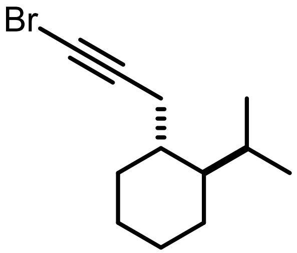

2f

^{1}H NMR(300 MHz, $CDCl_3$)

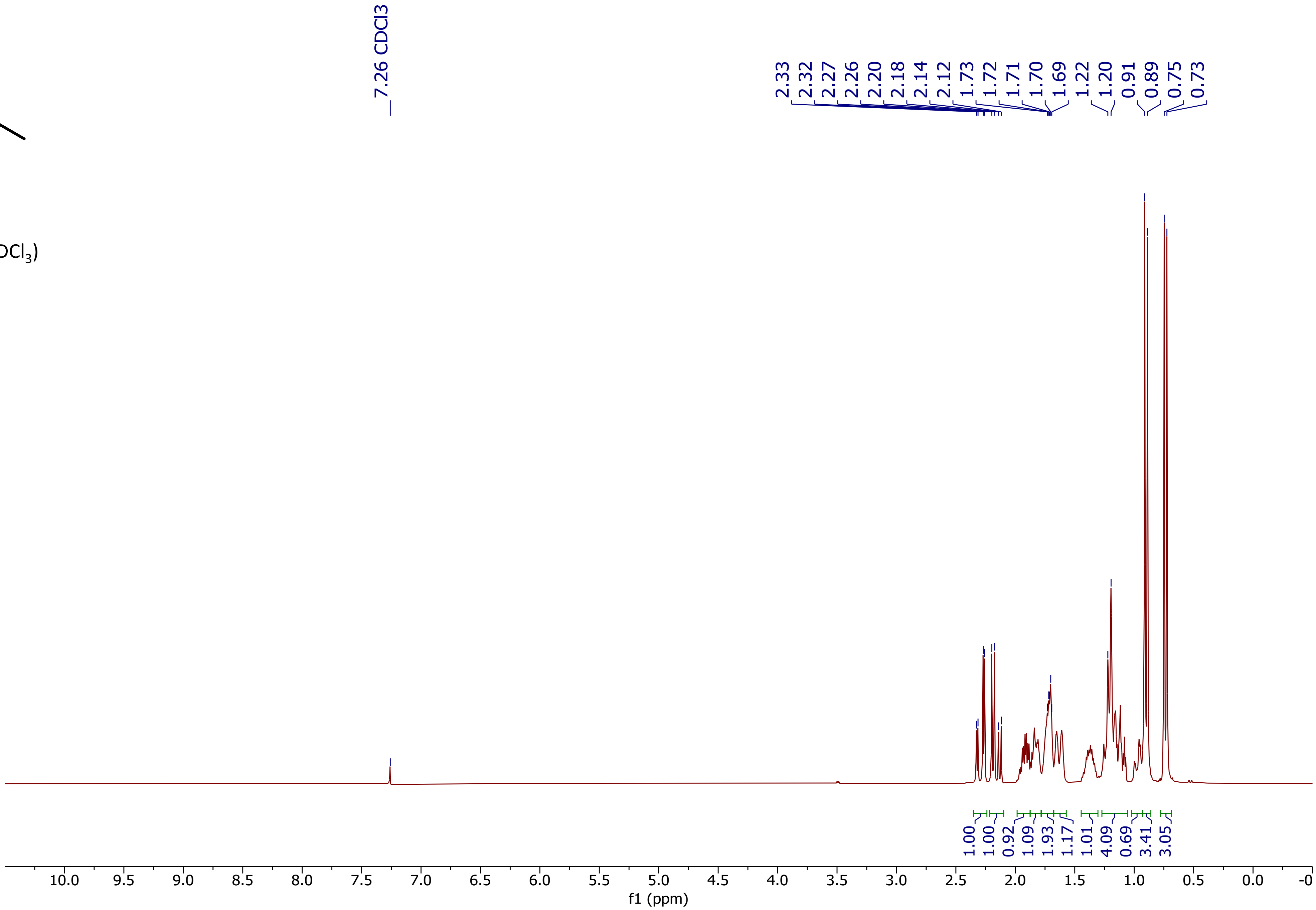

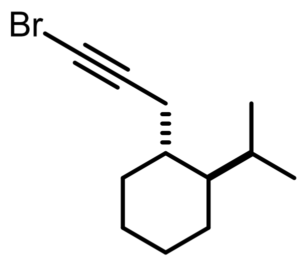

2f

^{13}C NMR (75 MHz, $CDCl_3$)

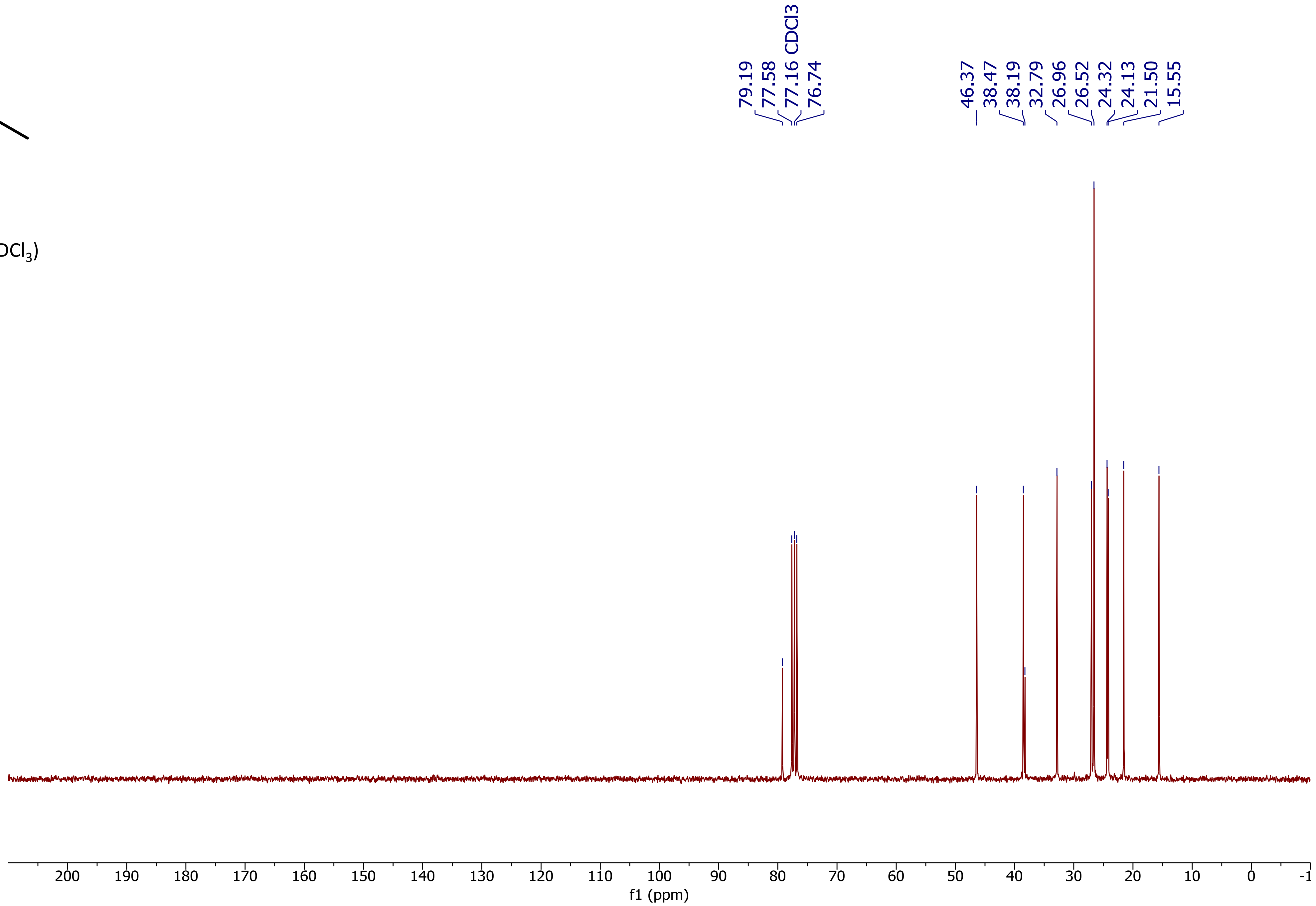

2g

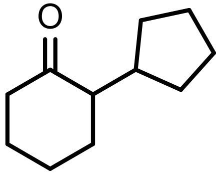

2g-CO

^{1}H NMR(300 MHz, $CDCl_3$)

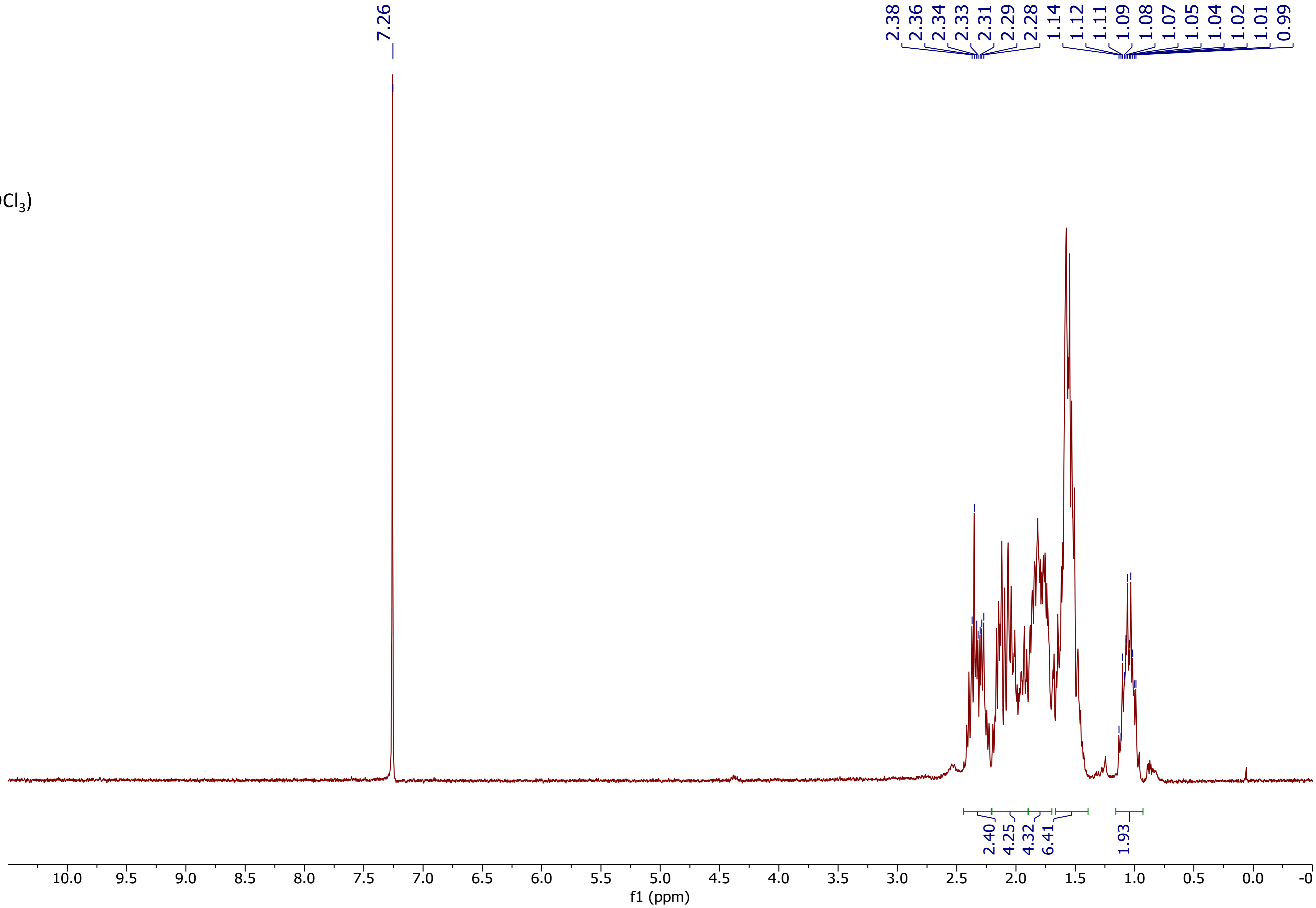

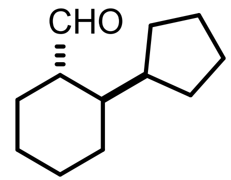

2g-CHO
-crude-

^{1}H NMR(300 MHz, $CDCl_3$)

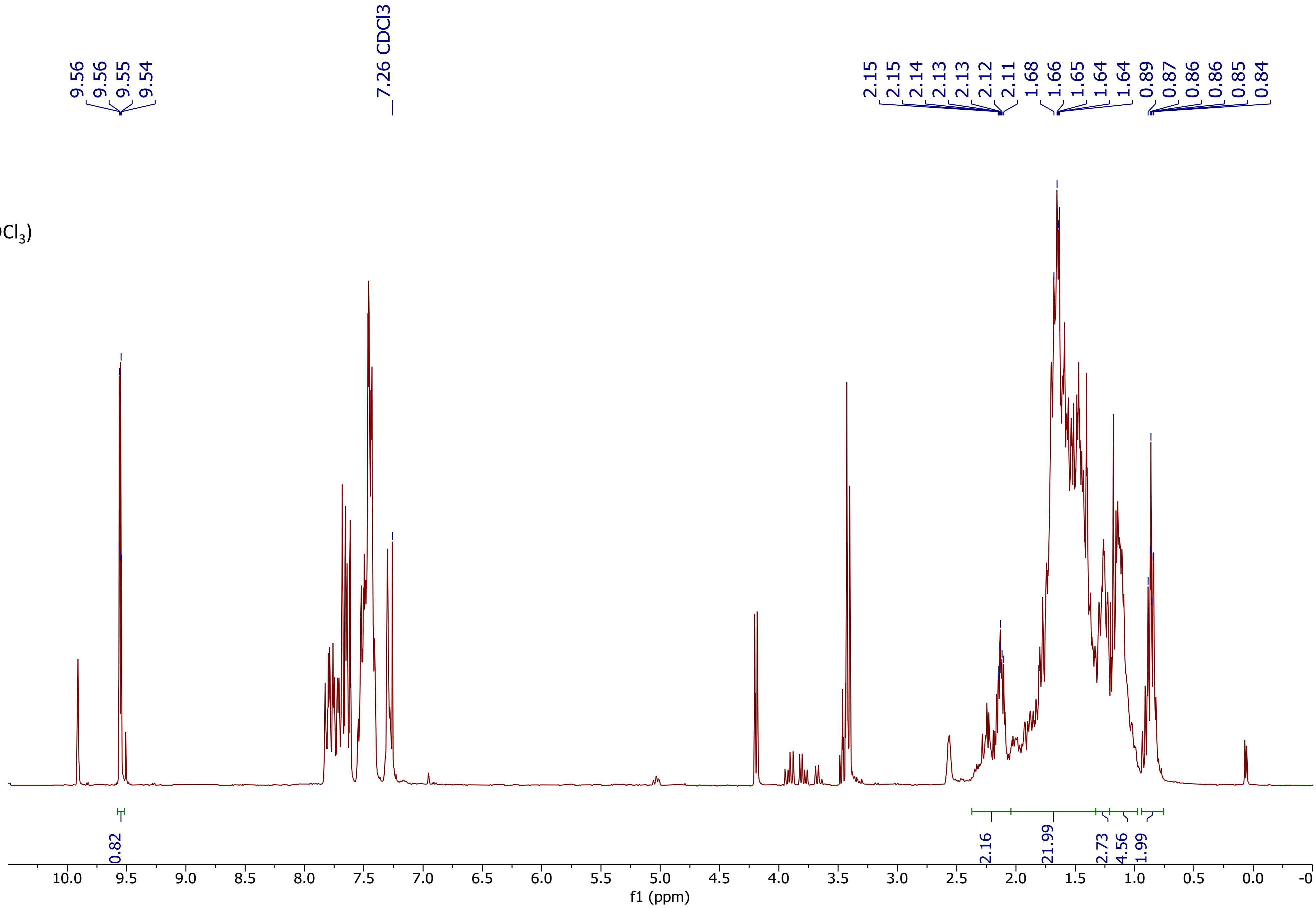

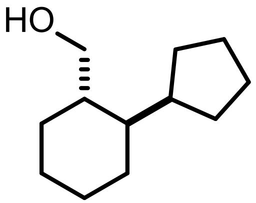

2g-OH
-crude-

^{1}H NMR(300 MHz, $CDCl_3$)

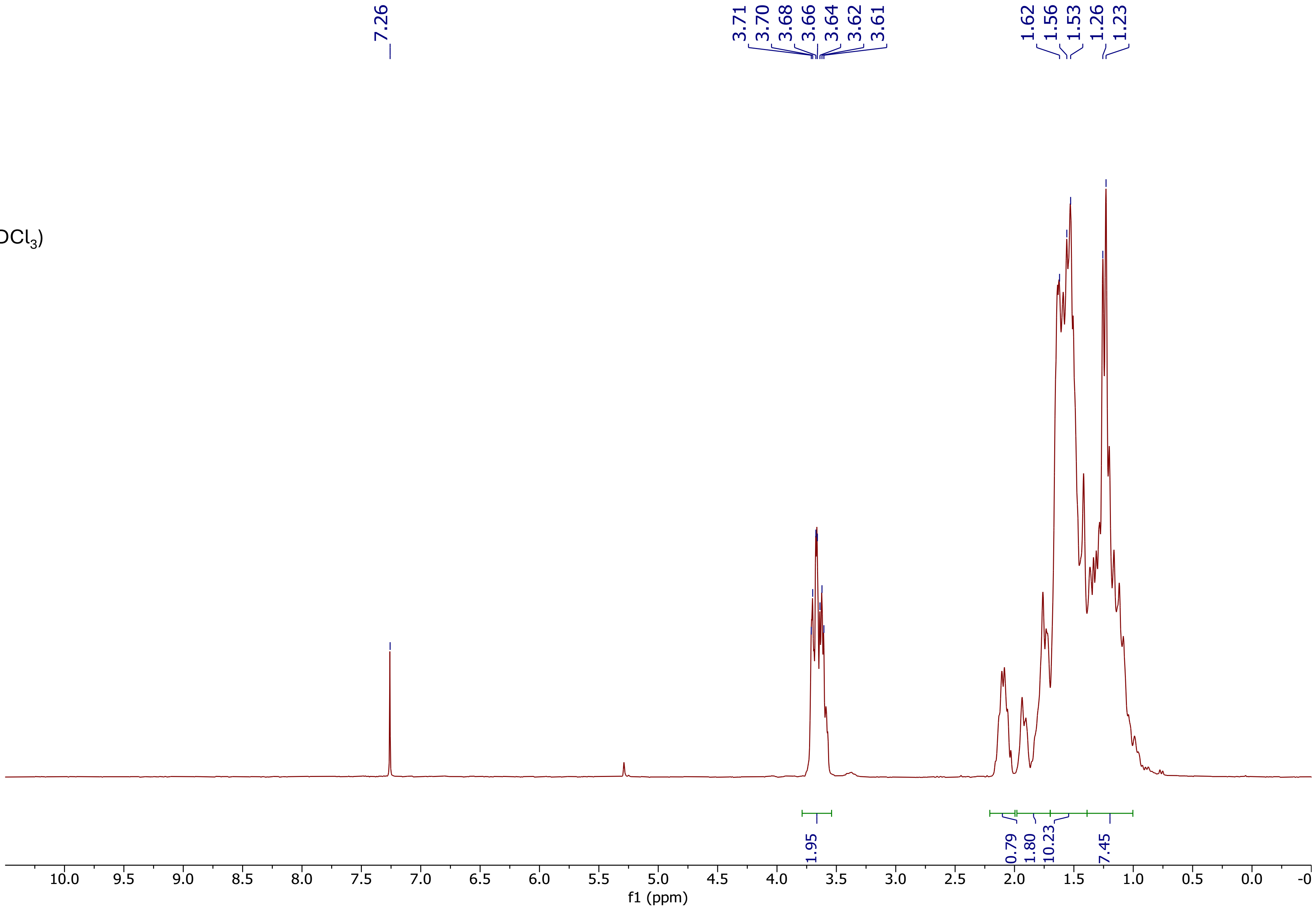

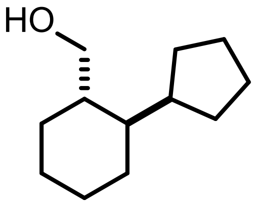

2g-OH
-crude-

^{13}C NMR (75 MHz, $CDCl_3$)

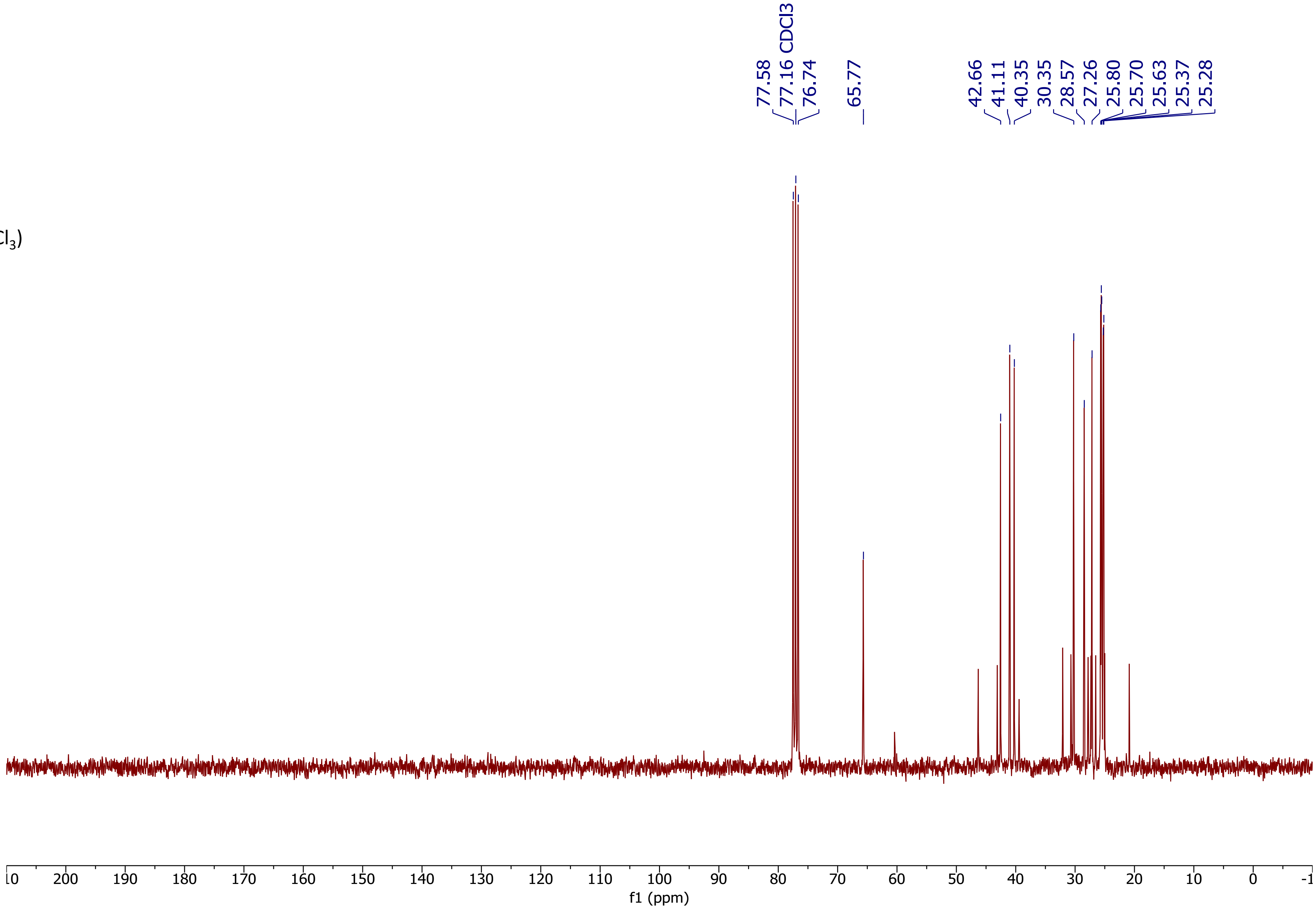

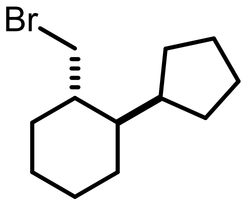

2g-Br

^{1}H NMR(300 MHz, $CDCl_3$)

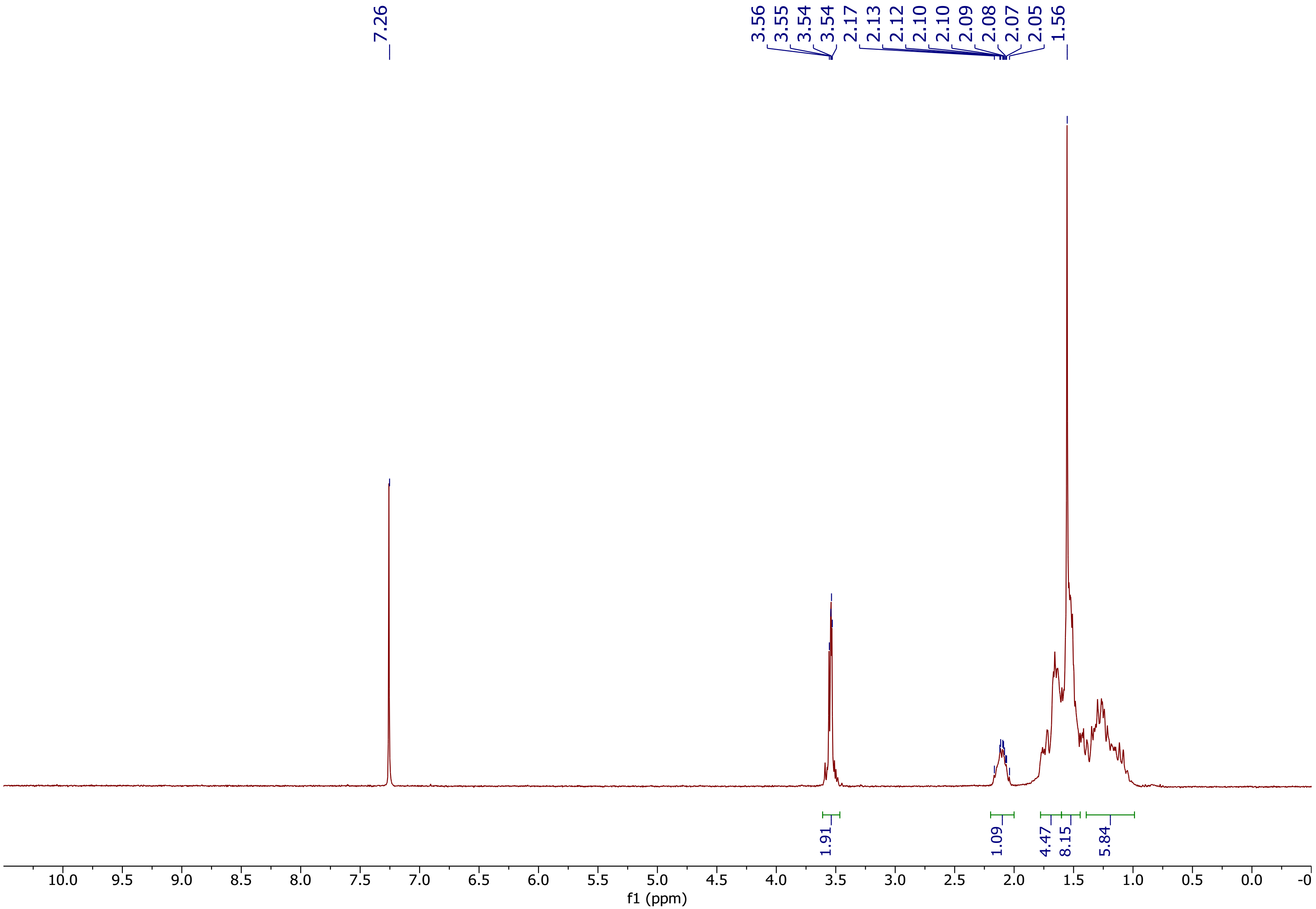

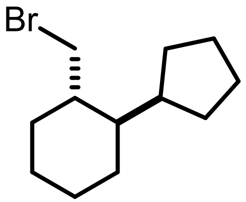

2g-Br

^{13}C NMR (75 MHz, $CDCl_3$)

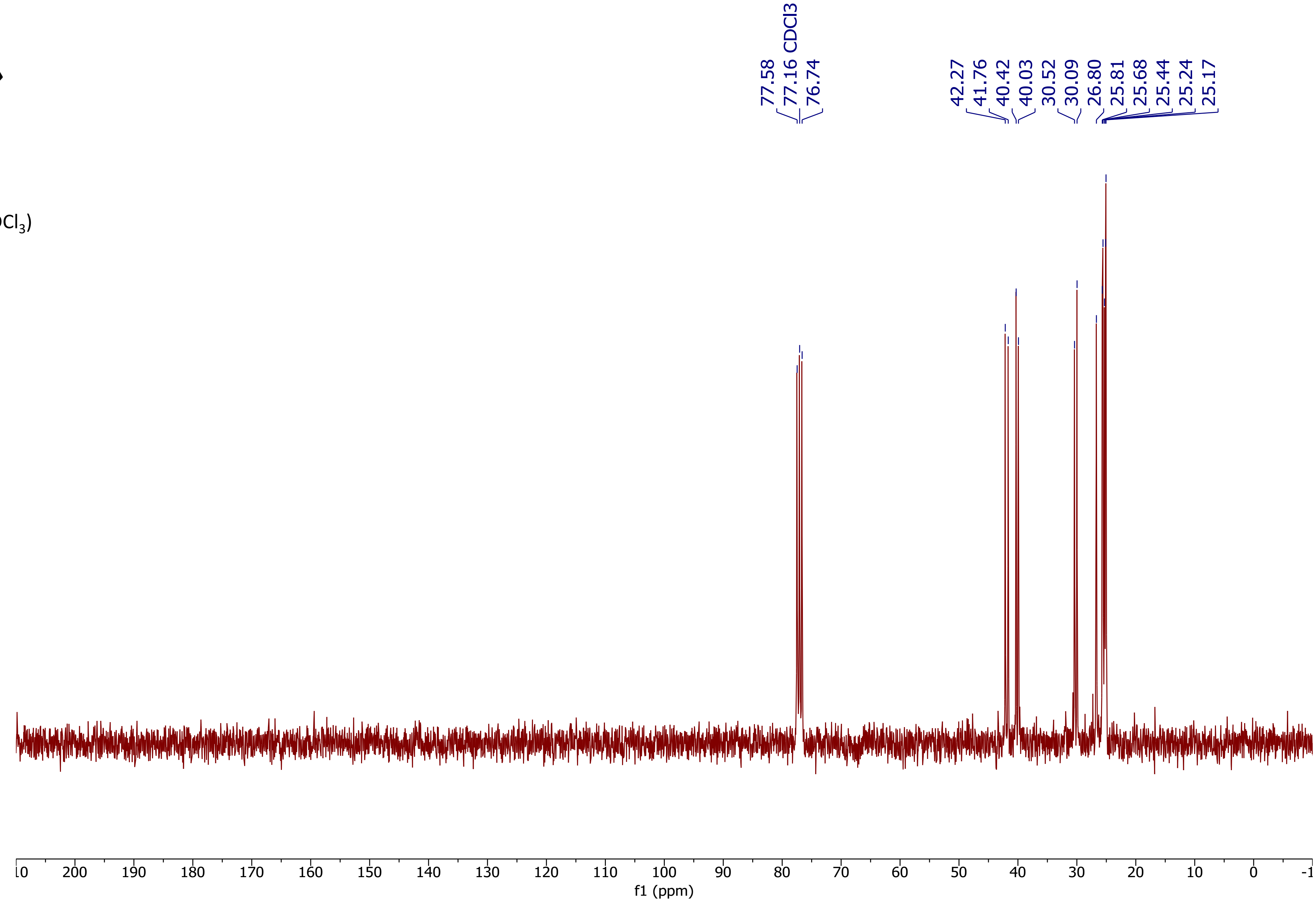

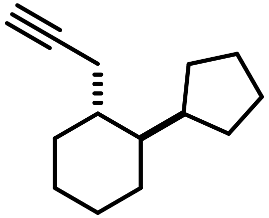

2g-CCH

^{1}H NMR(300 MHz, $CDCl_3$)

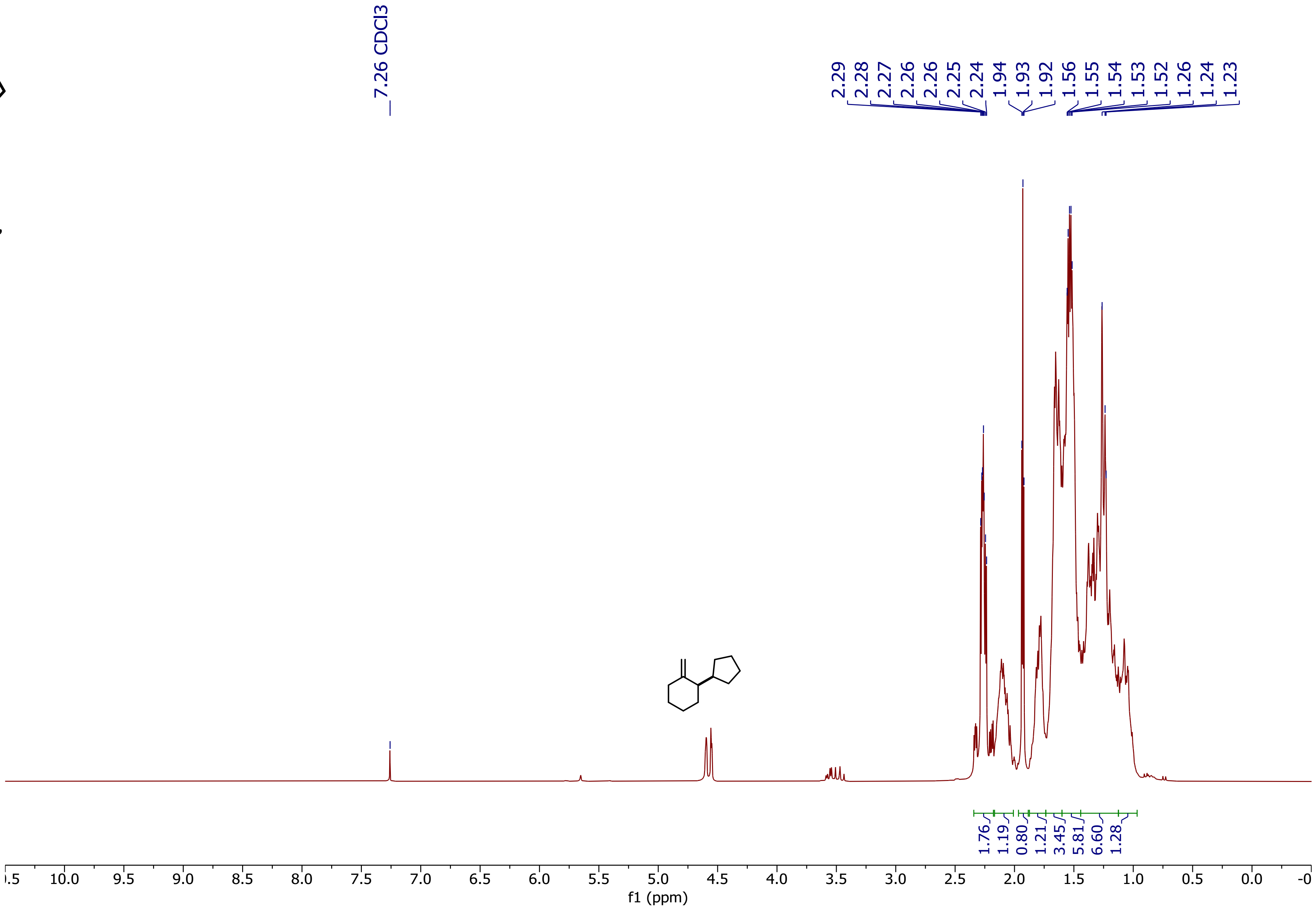

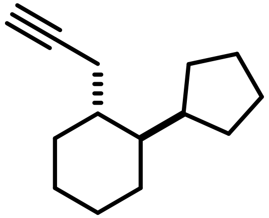

2g-CCH

^{13}C NMR (75 MHz, $CDCl_3$)

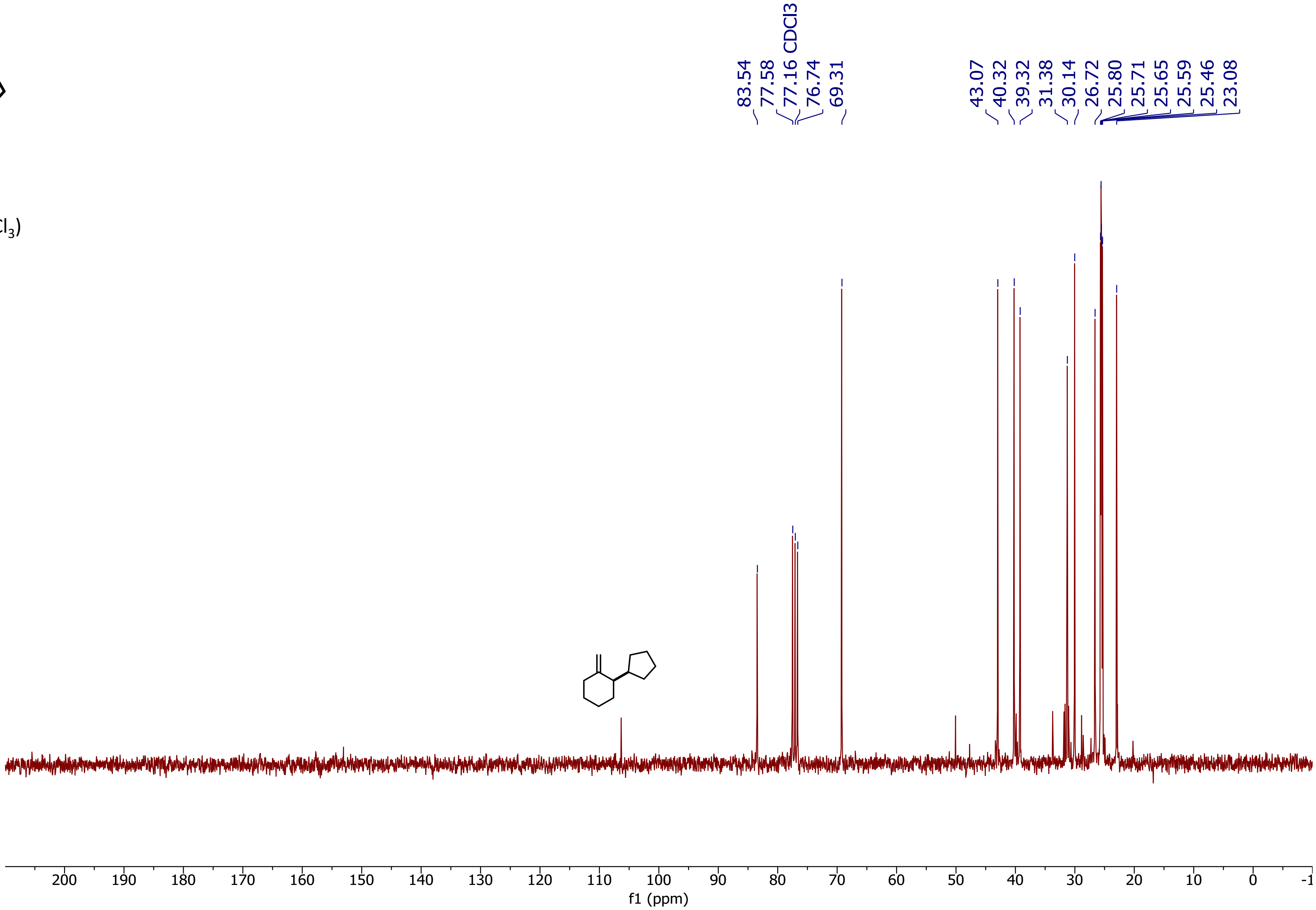

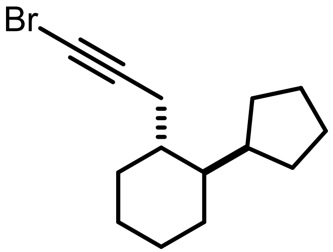

2g

^{1}H NMR(300 MHz, $CDCl_3$)

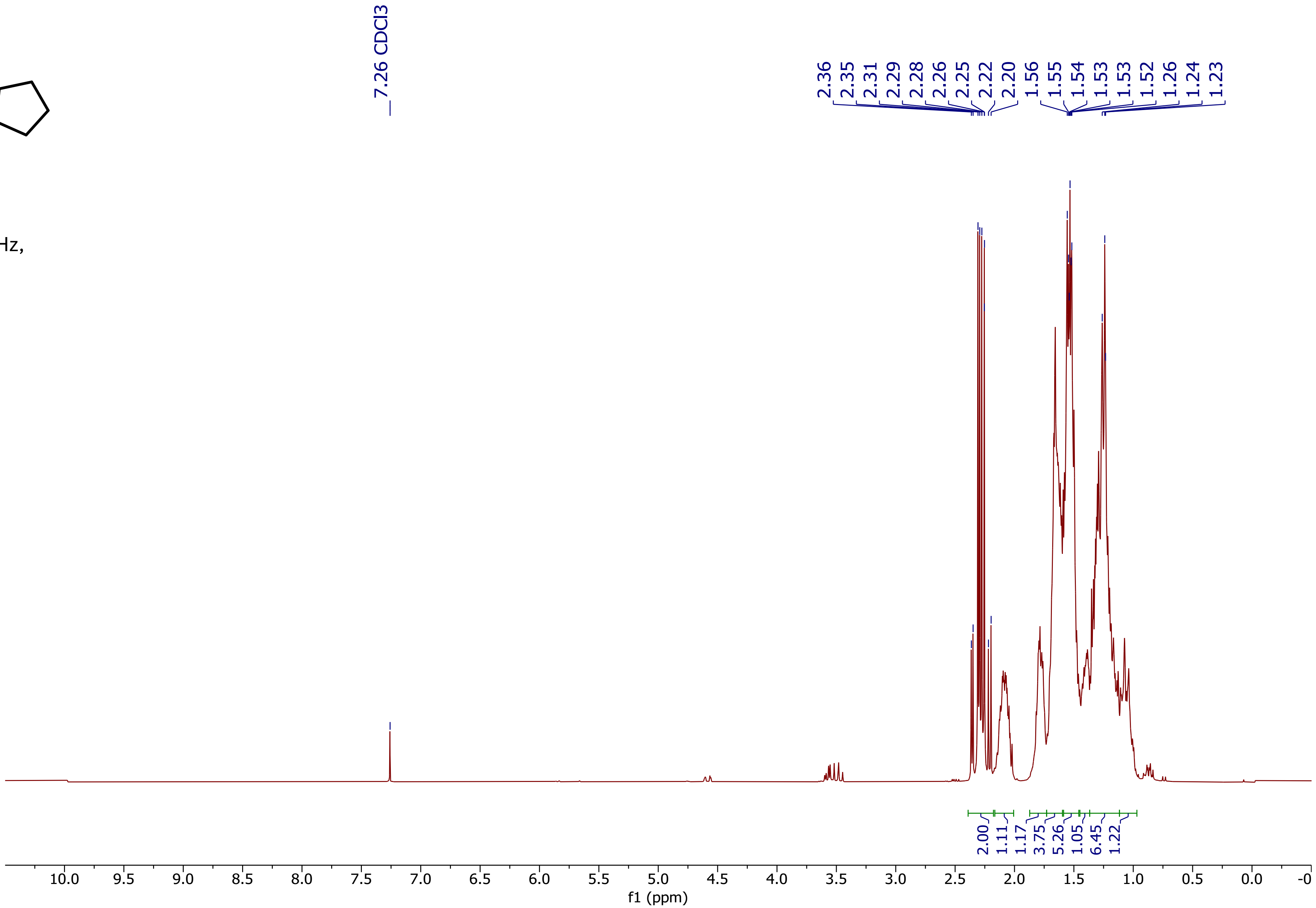

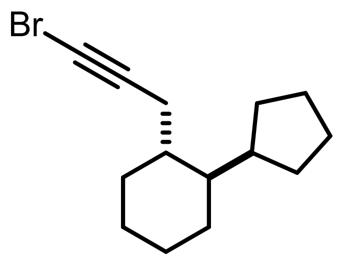

2g

^{13}C NMR (75 MHz, $CDCl_3$)

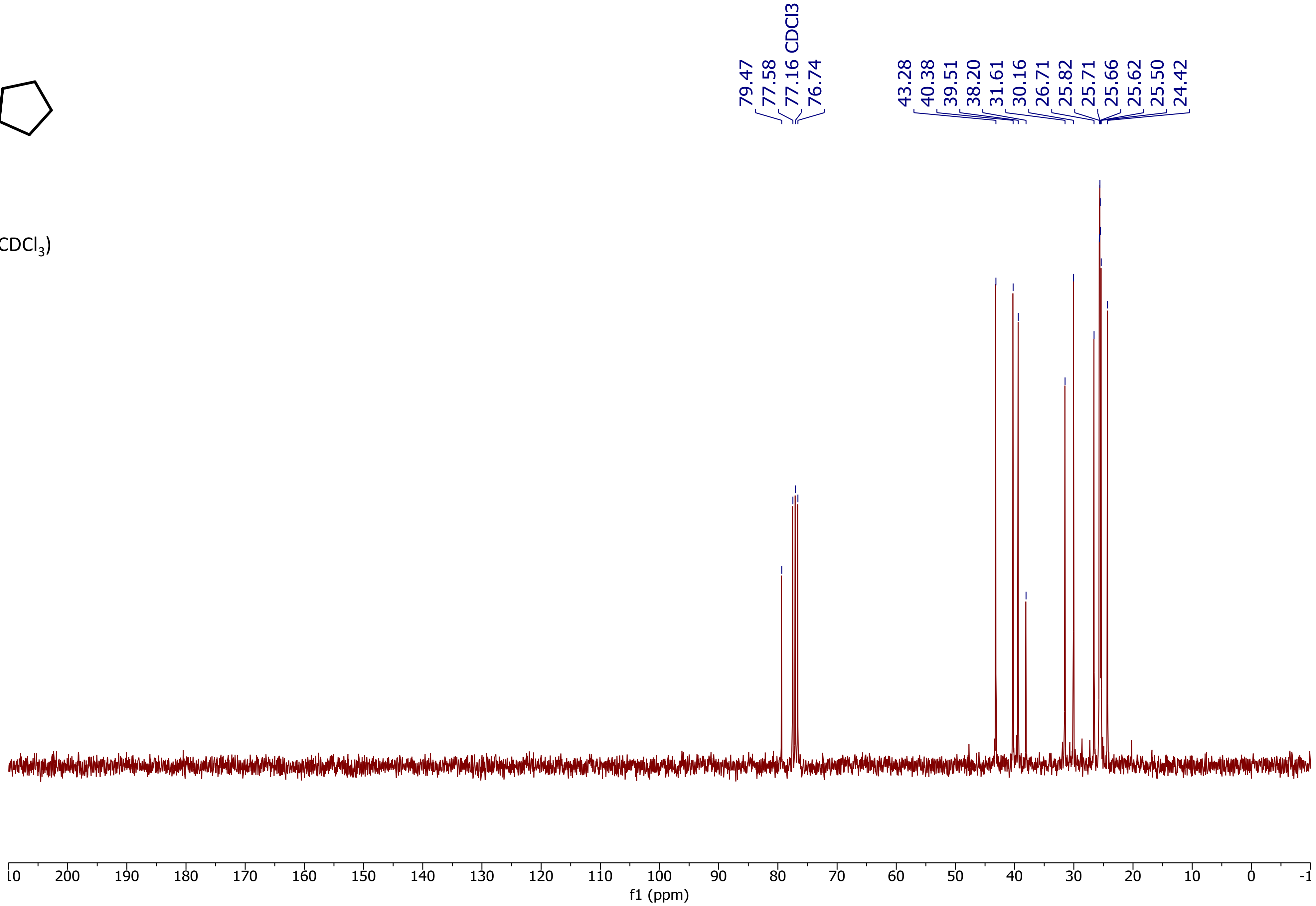

CRUDE REACTION MIXTURES AND PURIFIED PRODUCTS

BRIDGED FAMILY

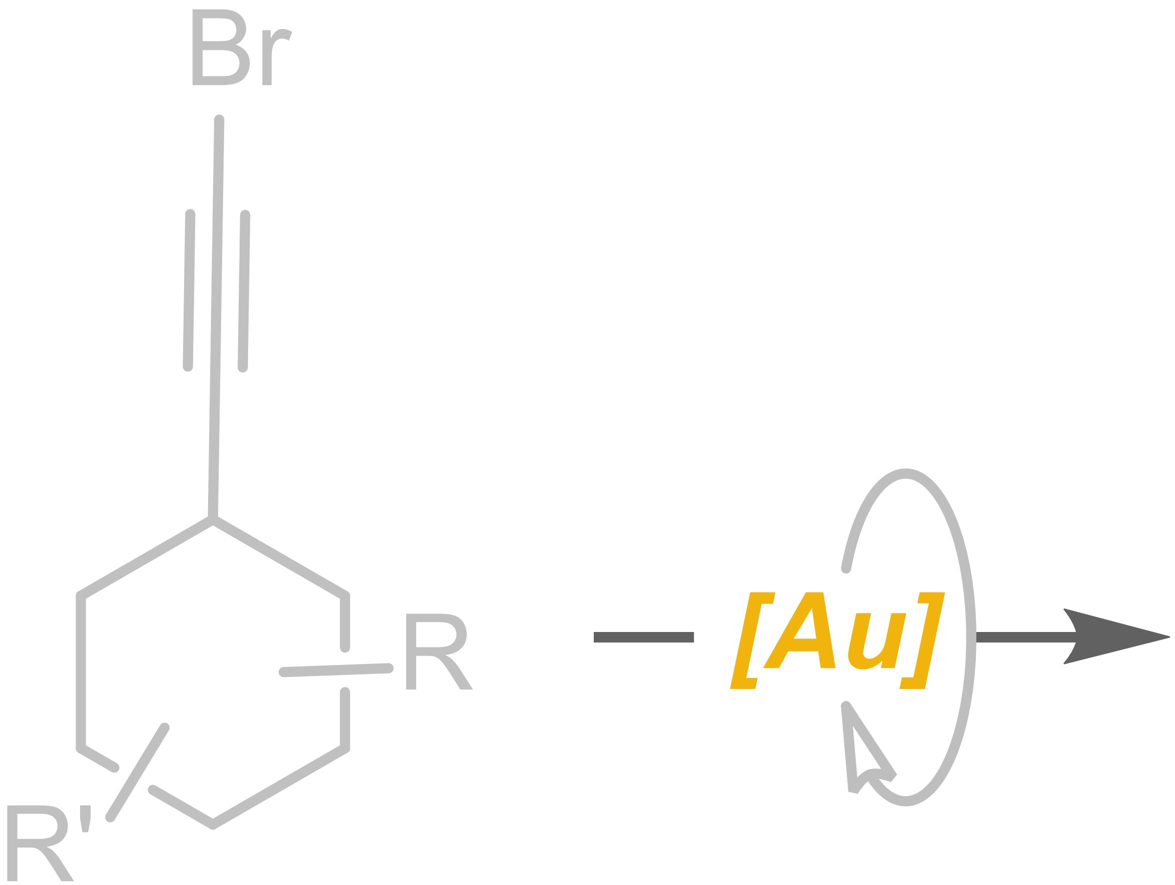

1a-d

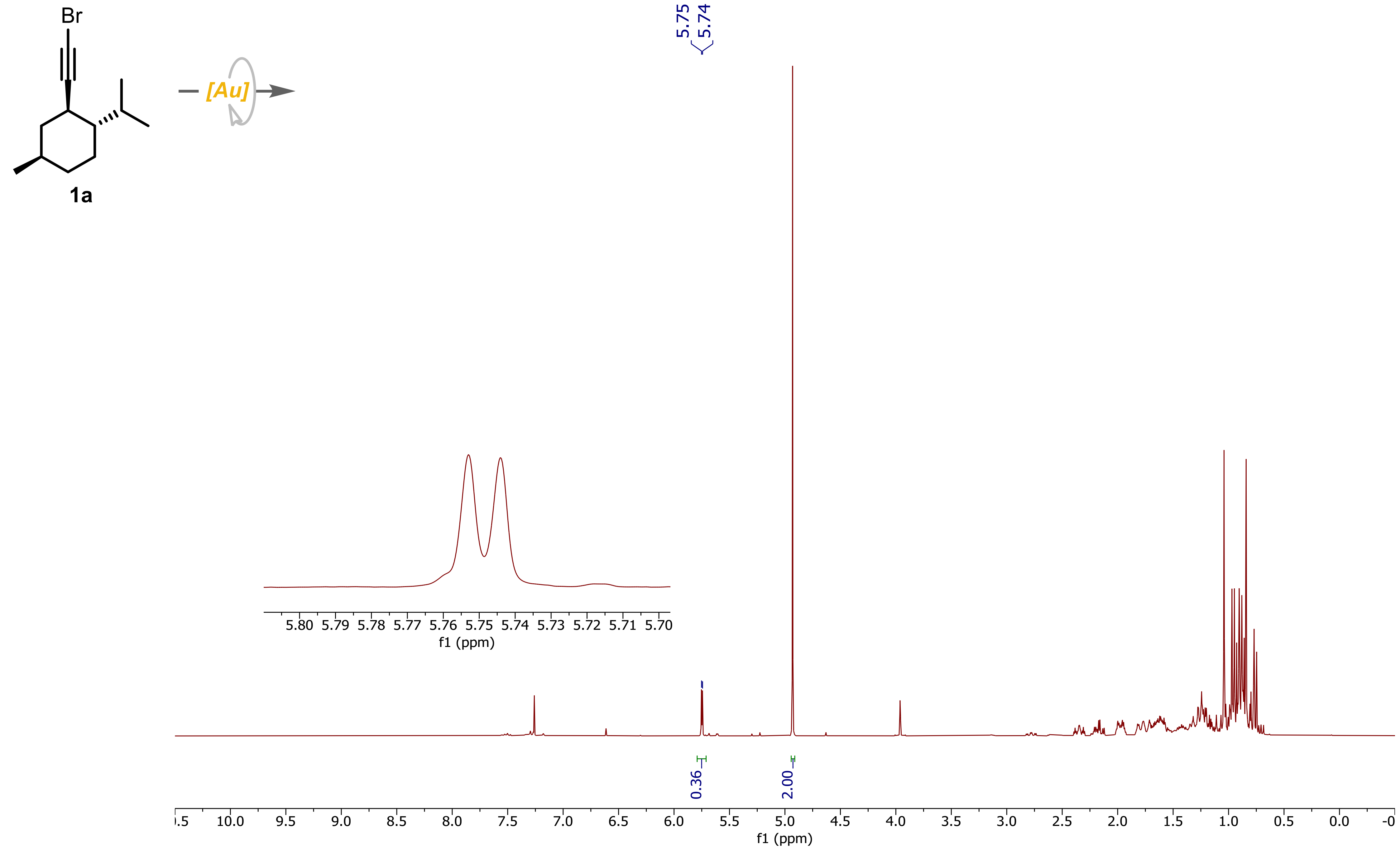
Br
[Au]
1a
5.75
5.74
5.80 5.79 5.78 5.77 5.76 5.75 5.74 5.73 5.72 5.71 5.70
f1 (ppm)
0.36
2.00
10.0 9.5 9.0 8.5 8.0 7.5 7.0 6.5 6.0 5.5 5.0 4.5 4.0 3.5 3.0 2.5 2.0 1.5 1.0 0.5 0.0
f1 (ppm)

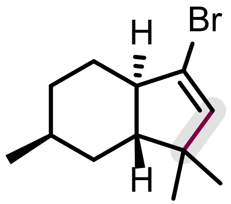

3a

^{1}H NMR(300 MHz, $CDCl_3$)

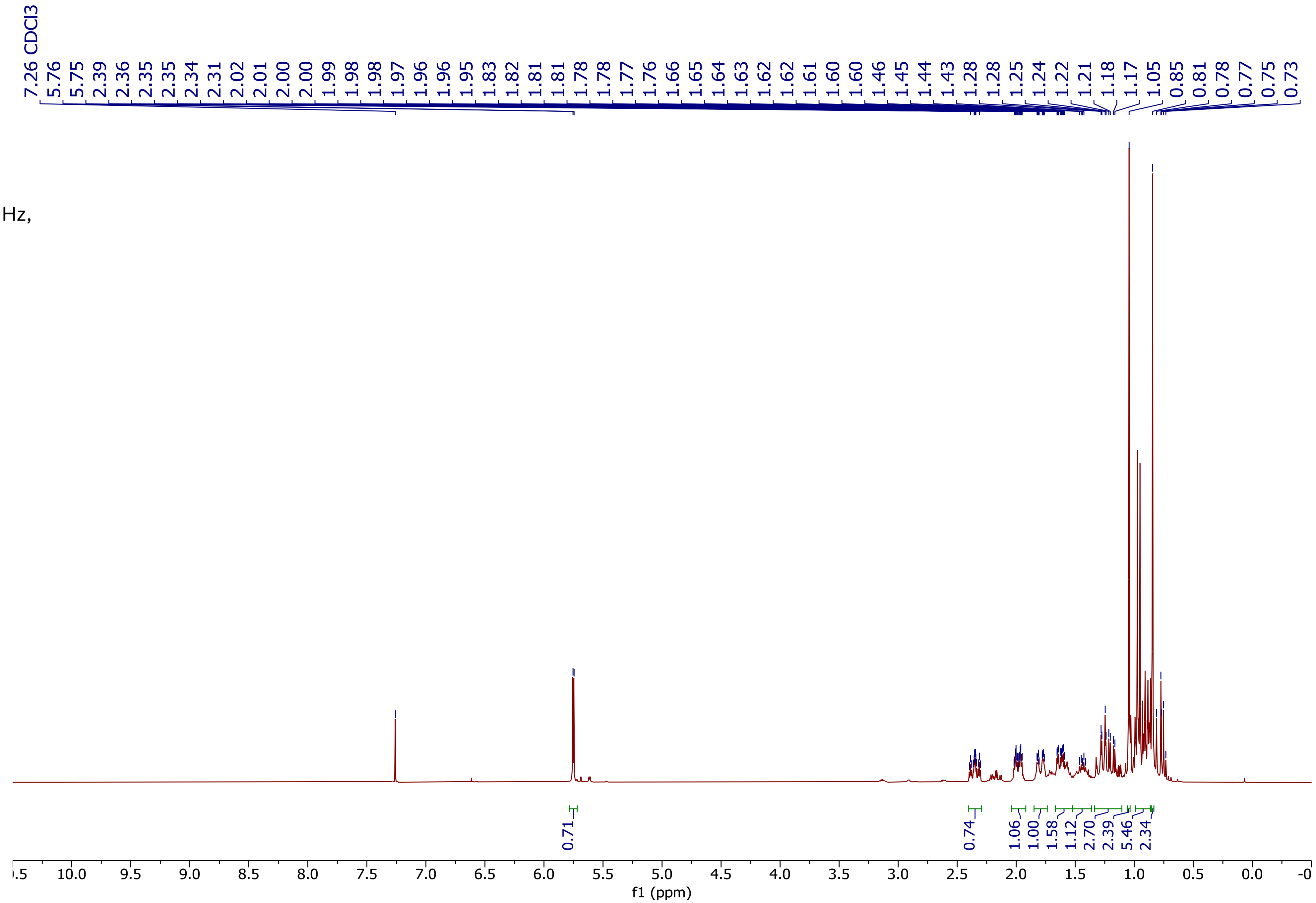

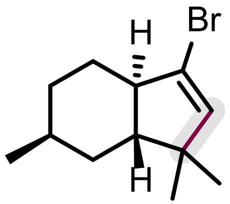

3a

^{13}C NMR (75 MHz, $CDCl_3$)

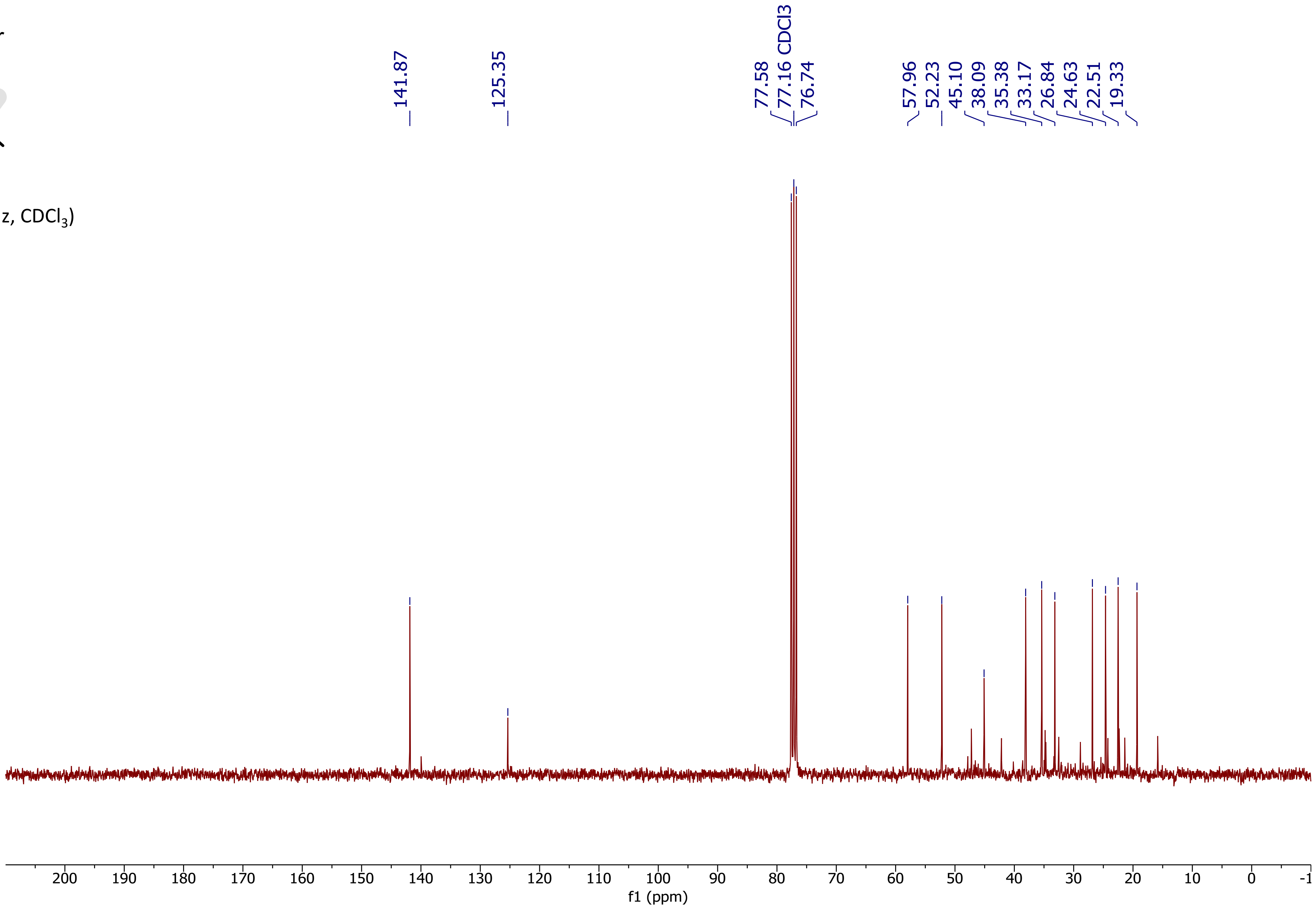

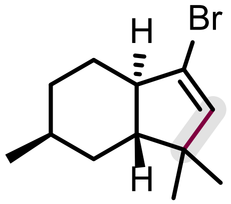

3a

COSY NMR([300, 300] MHz, $CDCl_3$)

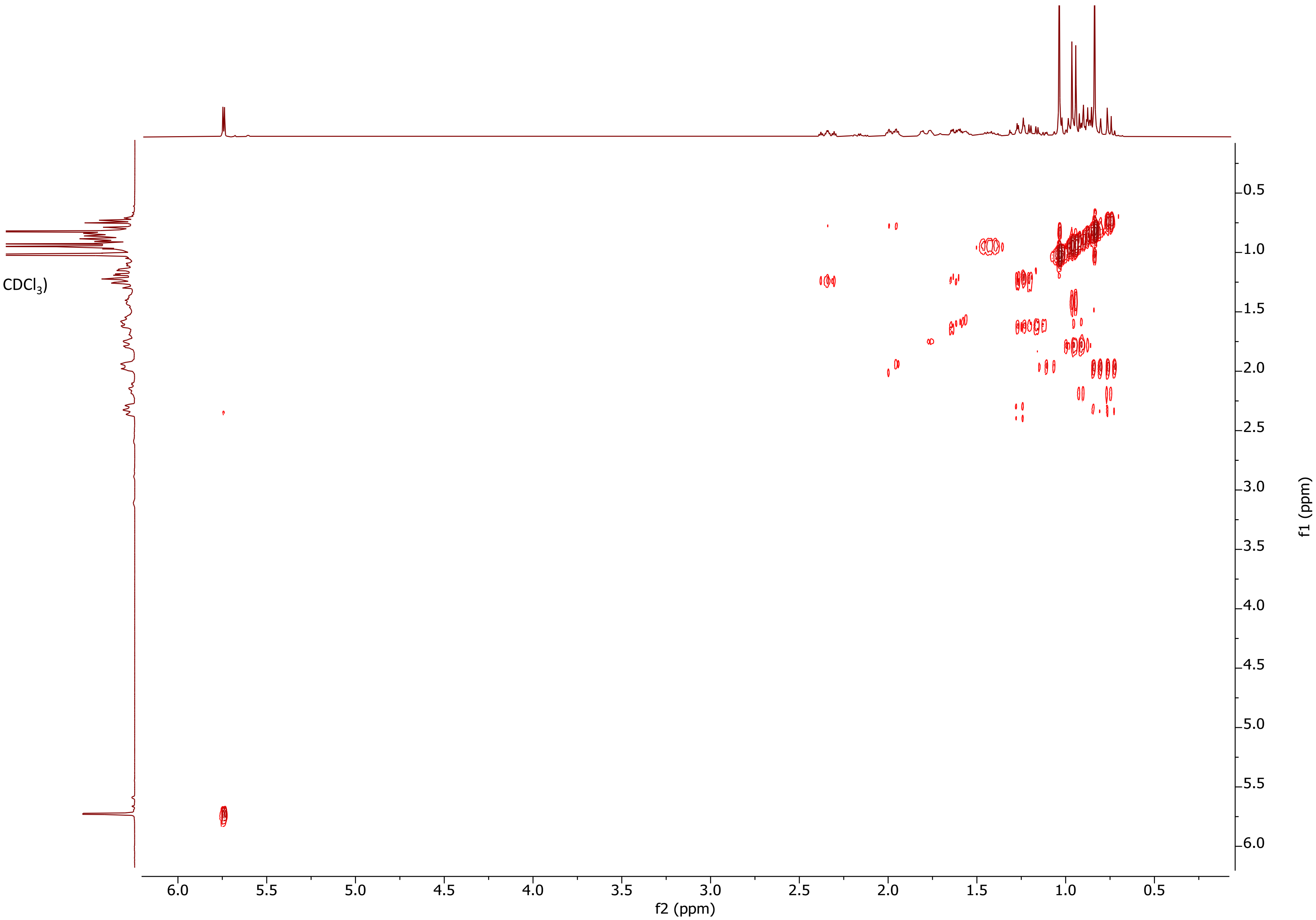

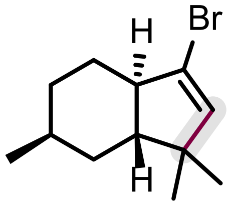

3a

HSQC NMR([300, 75] MHz, $CDCl_3$)

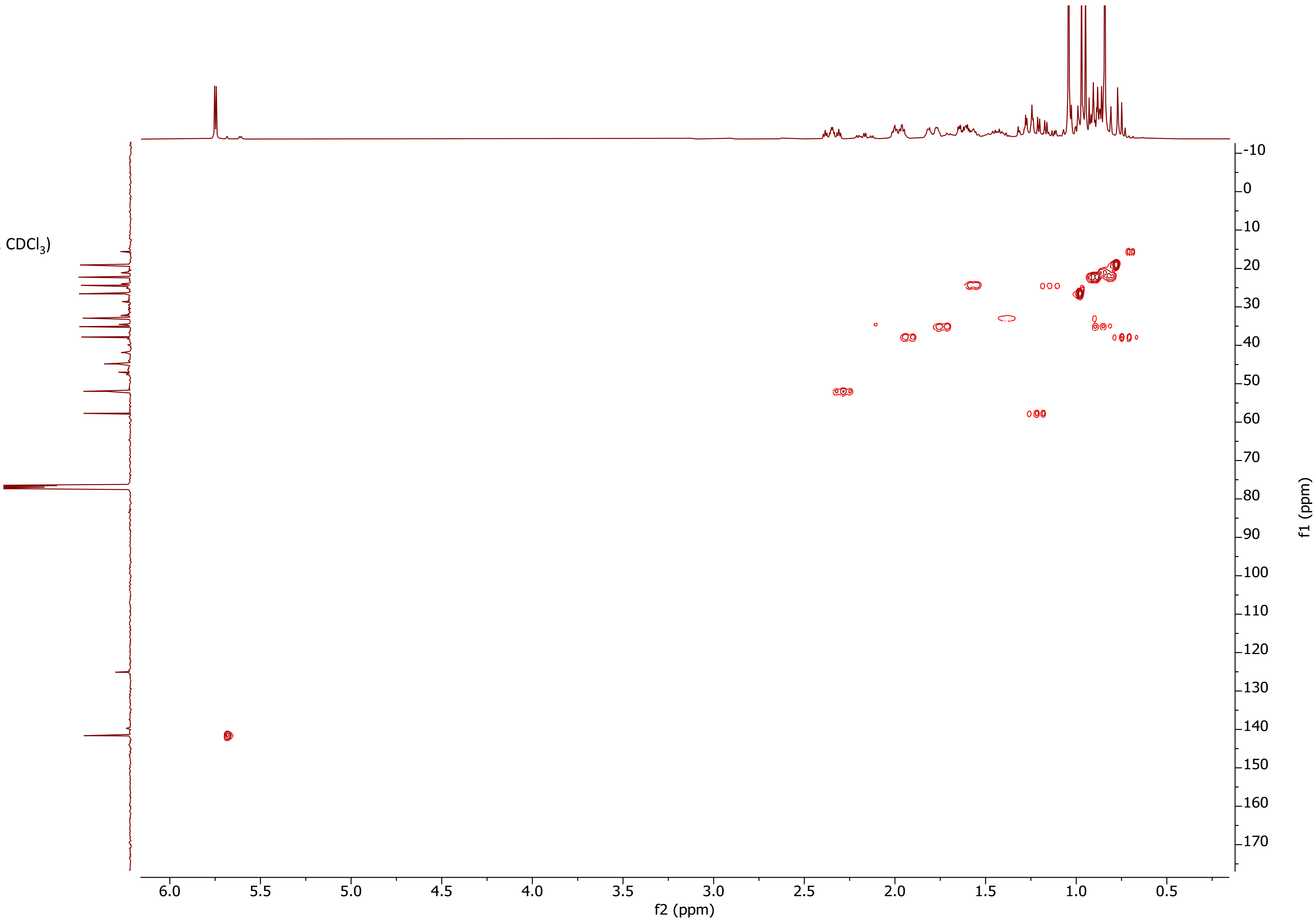

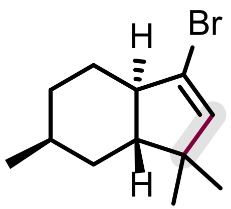

3a

HMBC NMR([300, 75] MHz, $CDCl_3$)

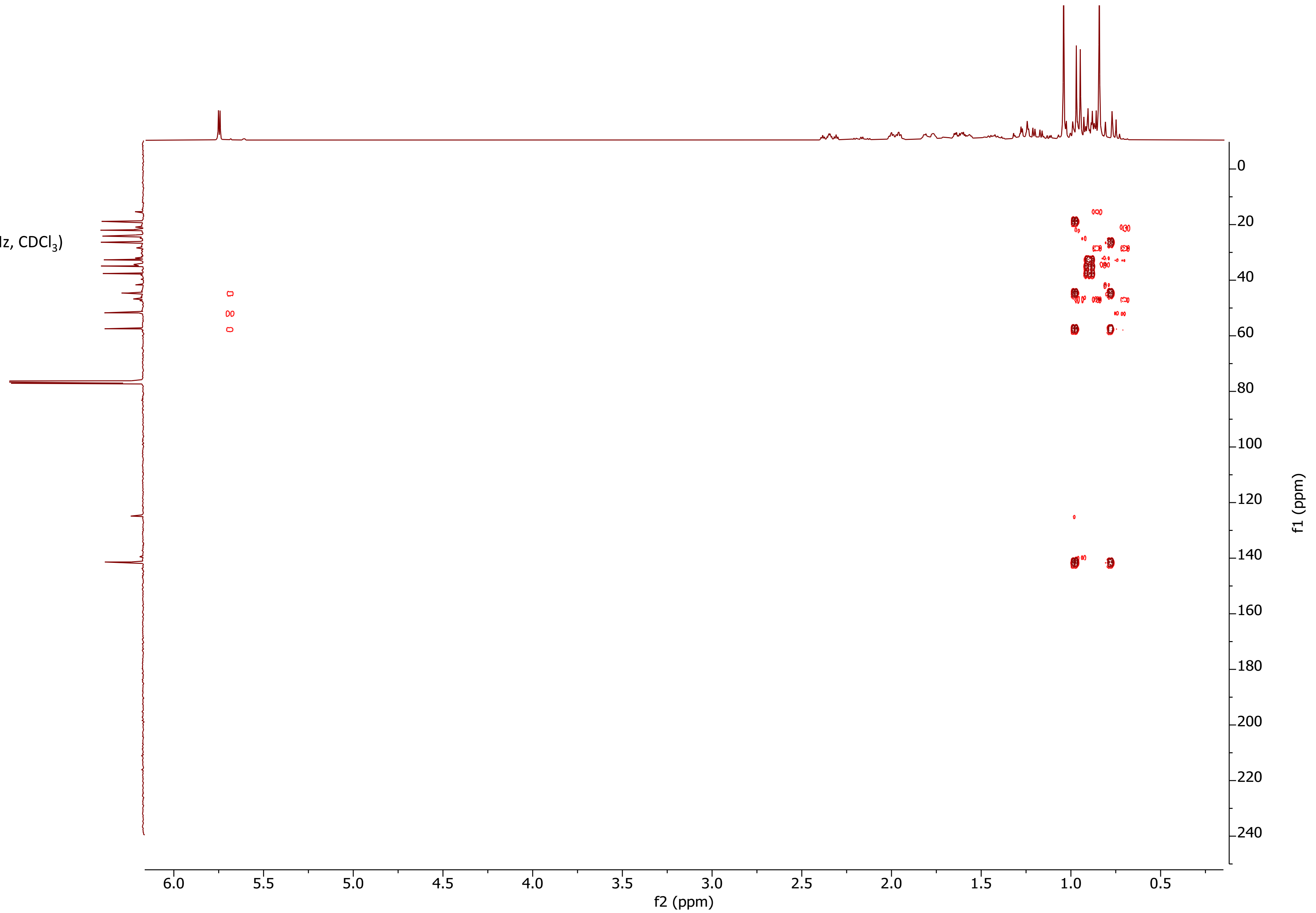

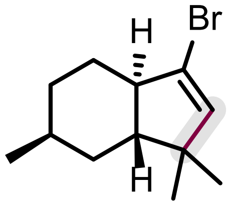

3a

NOESY NMR([300, 300] MHz, $CDCl_3$)

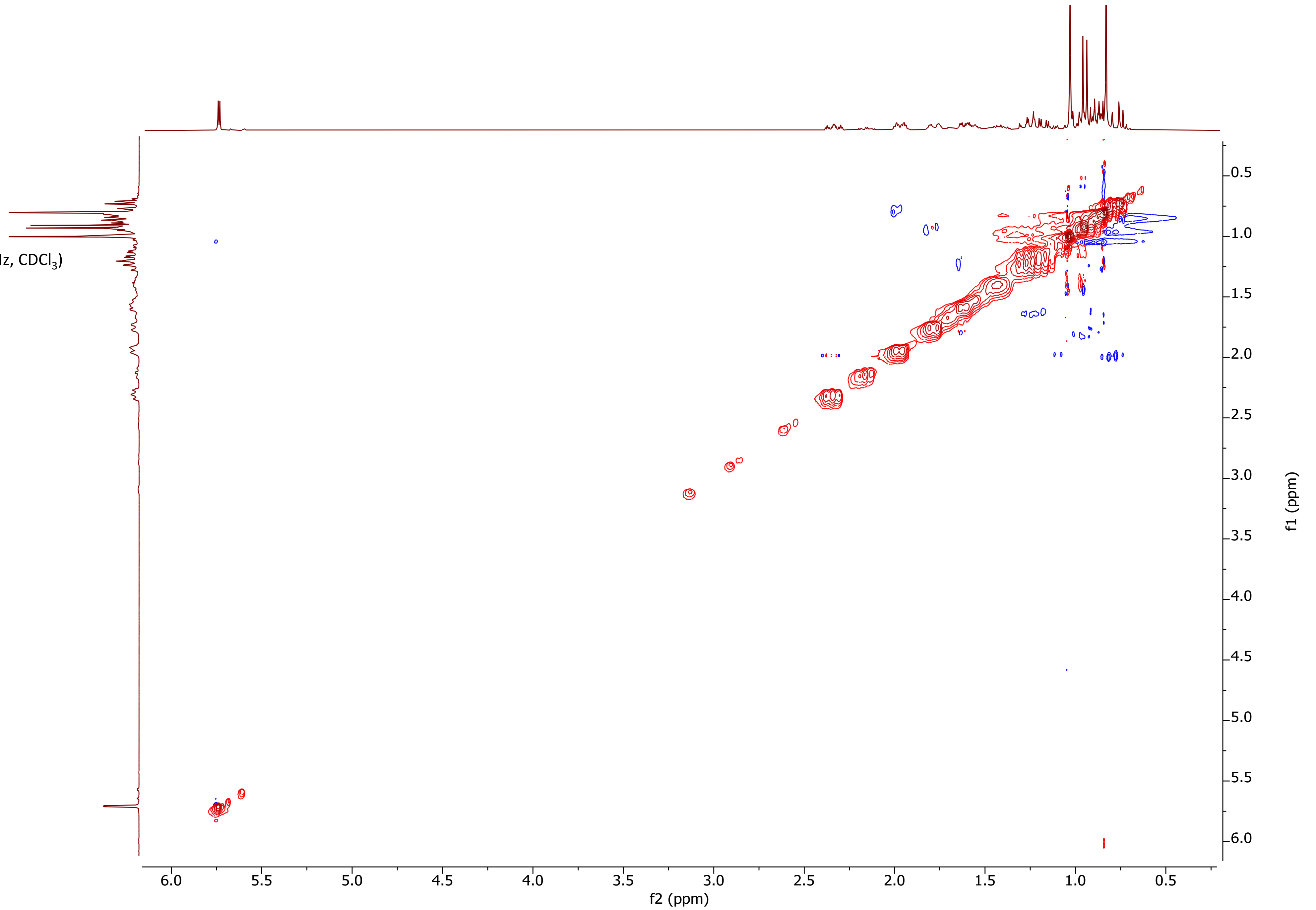

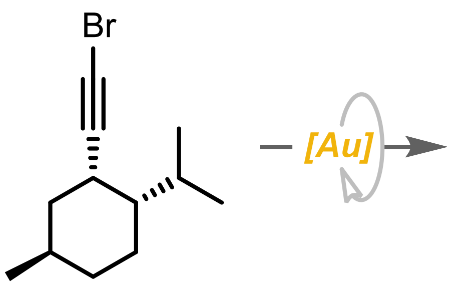
Br
[Au]

1b

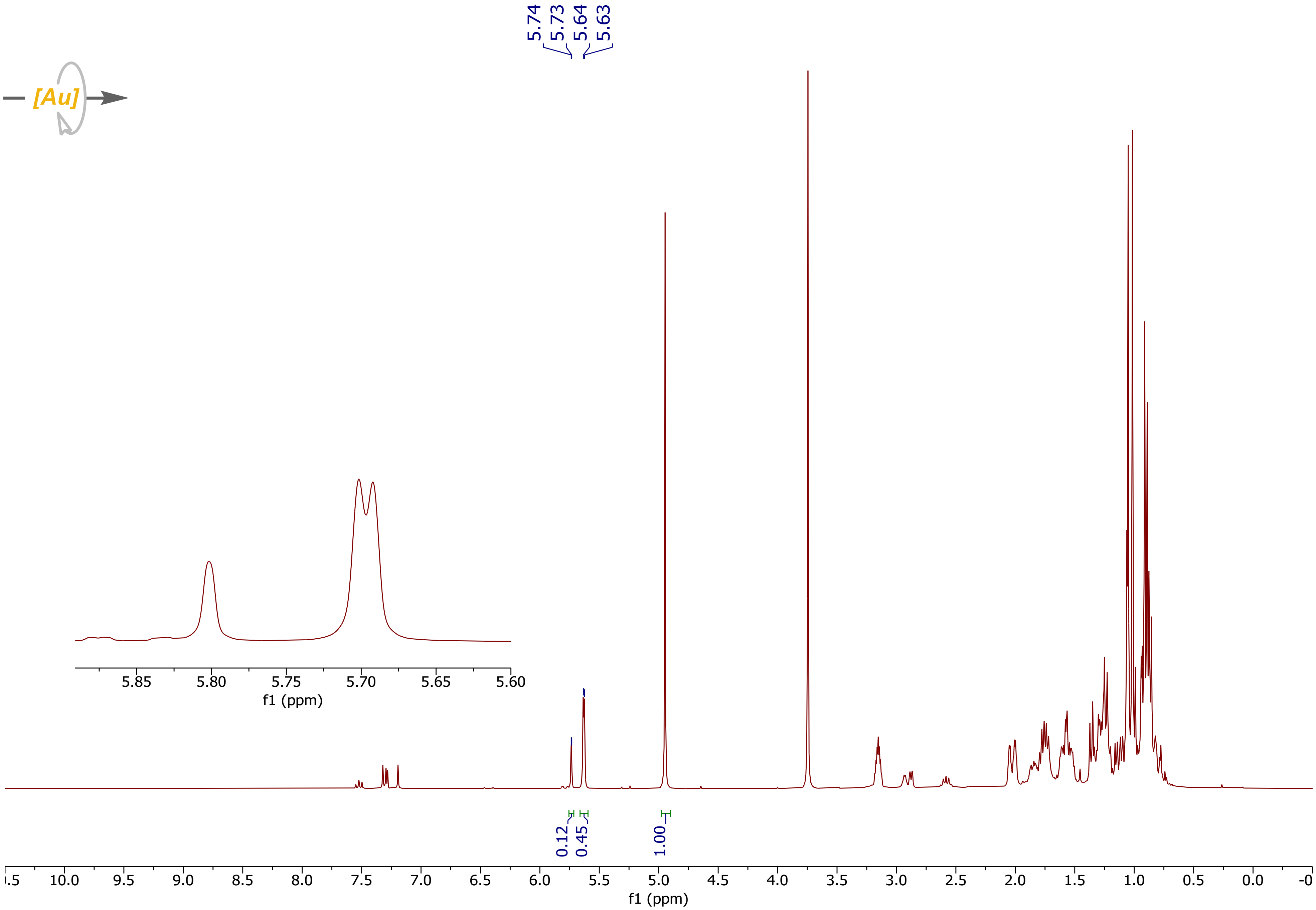
5.74
5.73
5.64
5.63
5.85
5.80
5.75
5.70
5.65
5.60
f1 (ppm)
0.12
0.45
1.00
10.0
9.5
9.0
8.5
8.0
7.5
7.0
6.5
6.0
5.5
5.0
4.5
4.0
3.5
3.0
2.5
2.0
1.5
1.0
0.5
0.0
f1 (ppm)

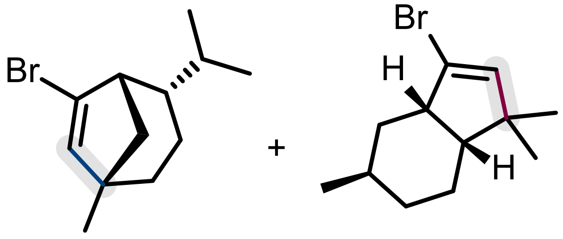

4b **3b**

^{1}H NMR(400 MHz, $CDCl_3$)

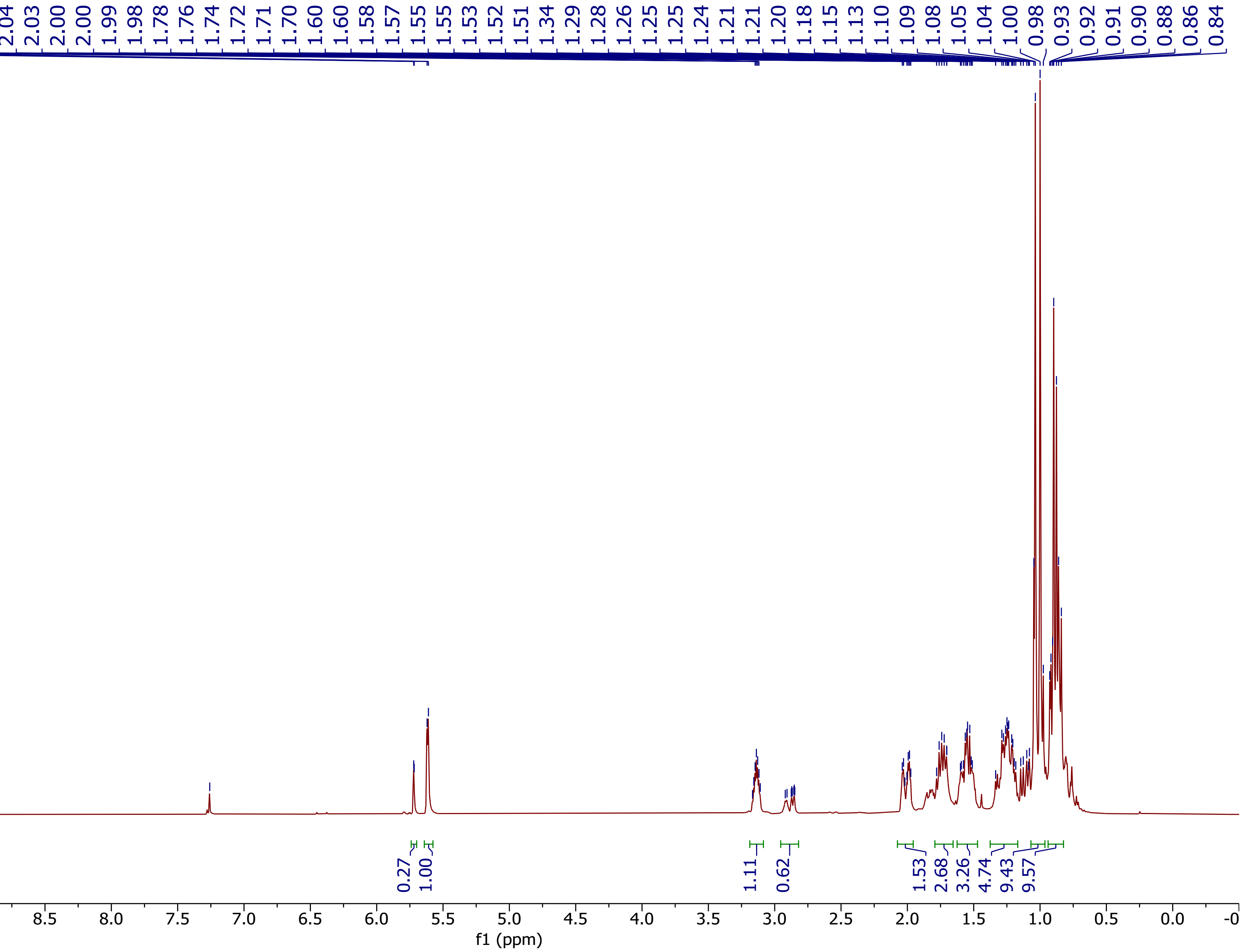

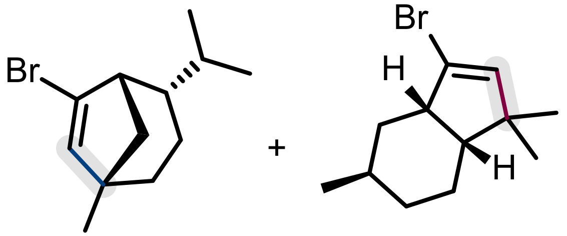

4b **3b**

^{13}**C NMR** (101 MHz, $CDCl_3$)

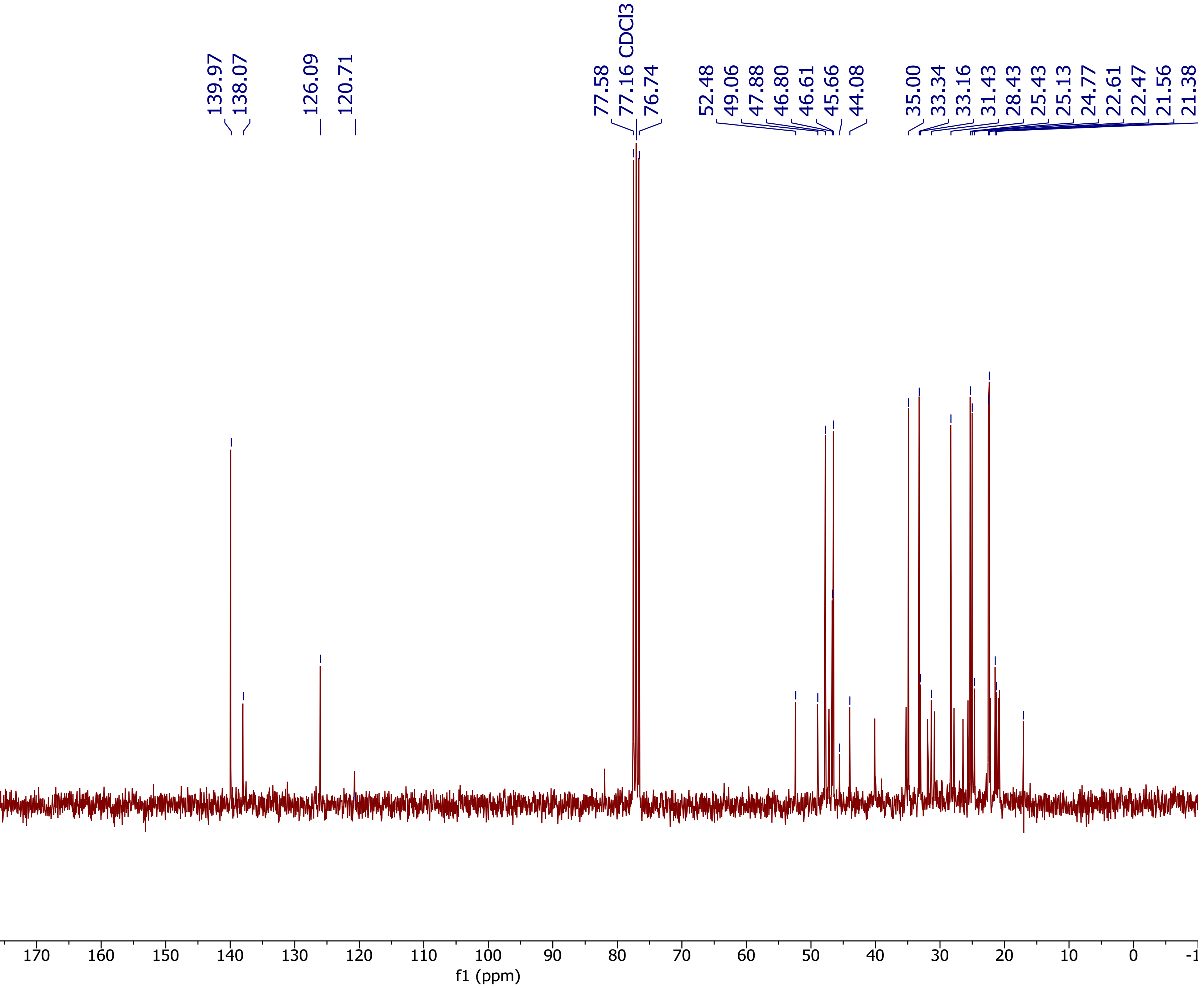

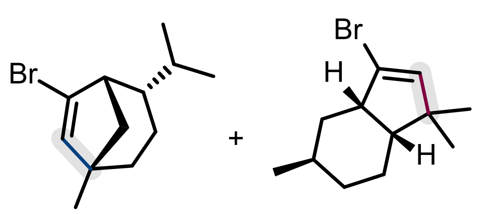

4b **3b**

COSY NMR([400, 400] MHz, $CDCl_3$)

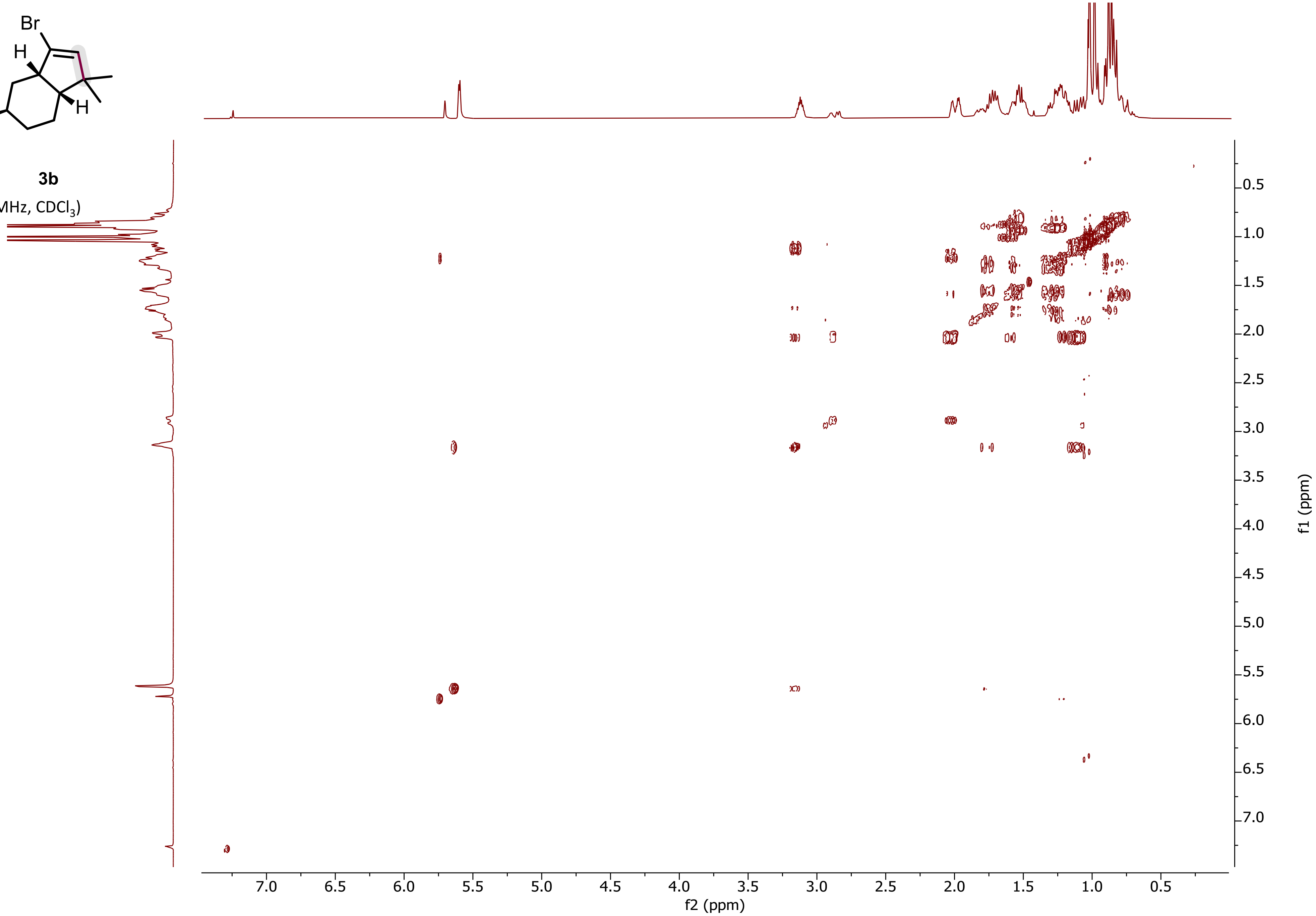

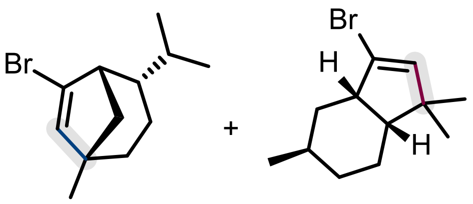

4b **3b**

HSQC NMR([400, 101] MHz, $CDCl_3$)

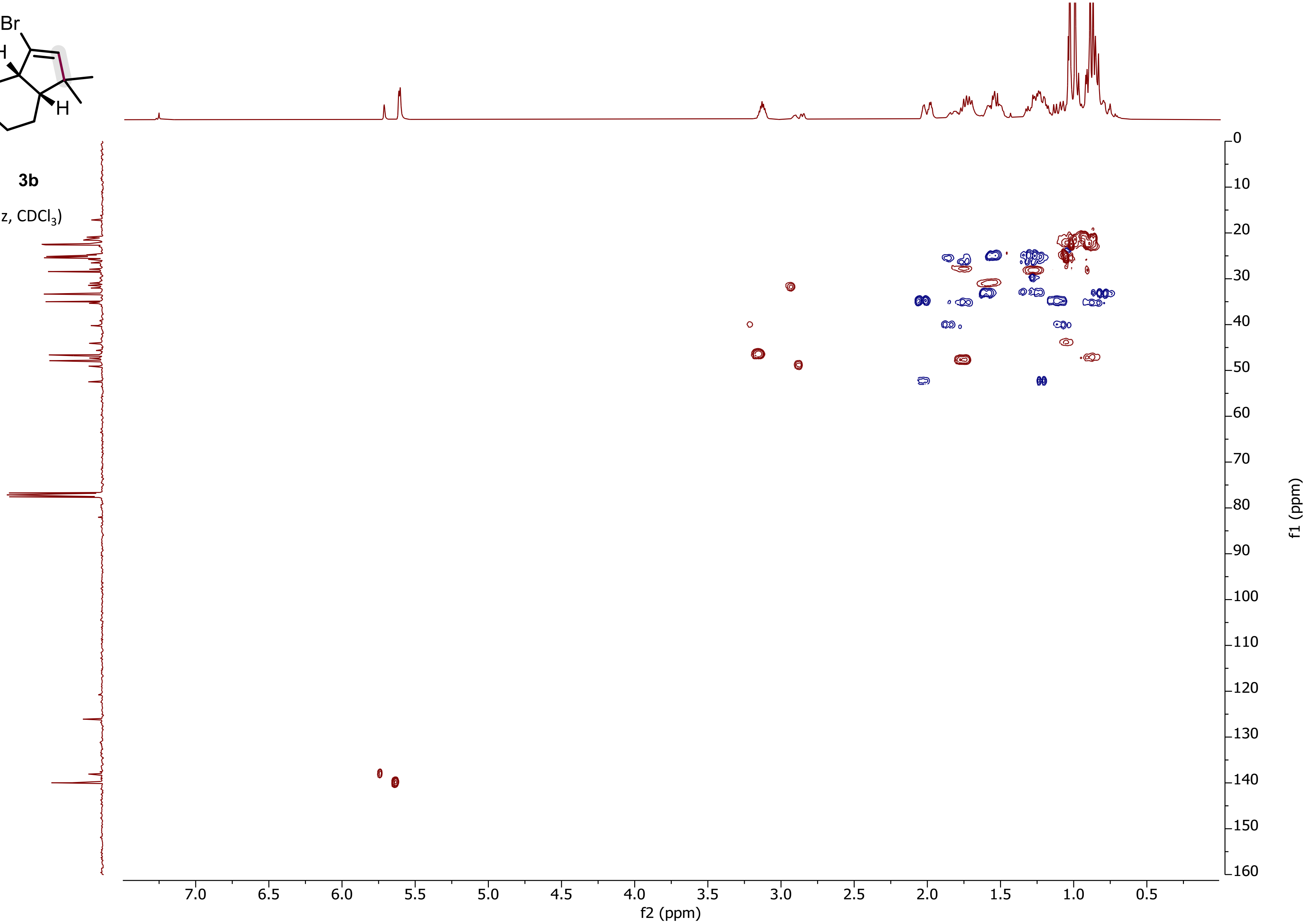

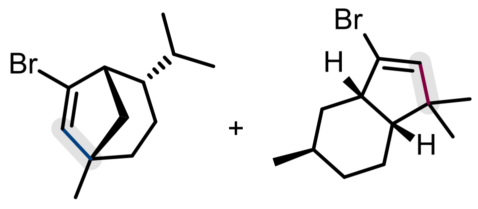

4b + **3b**

HMBC NMR([400, 101] MHz, $CDCl_3$)

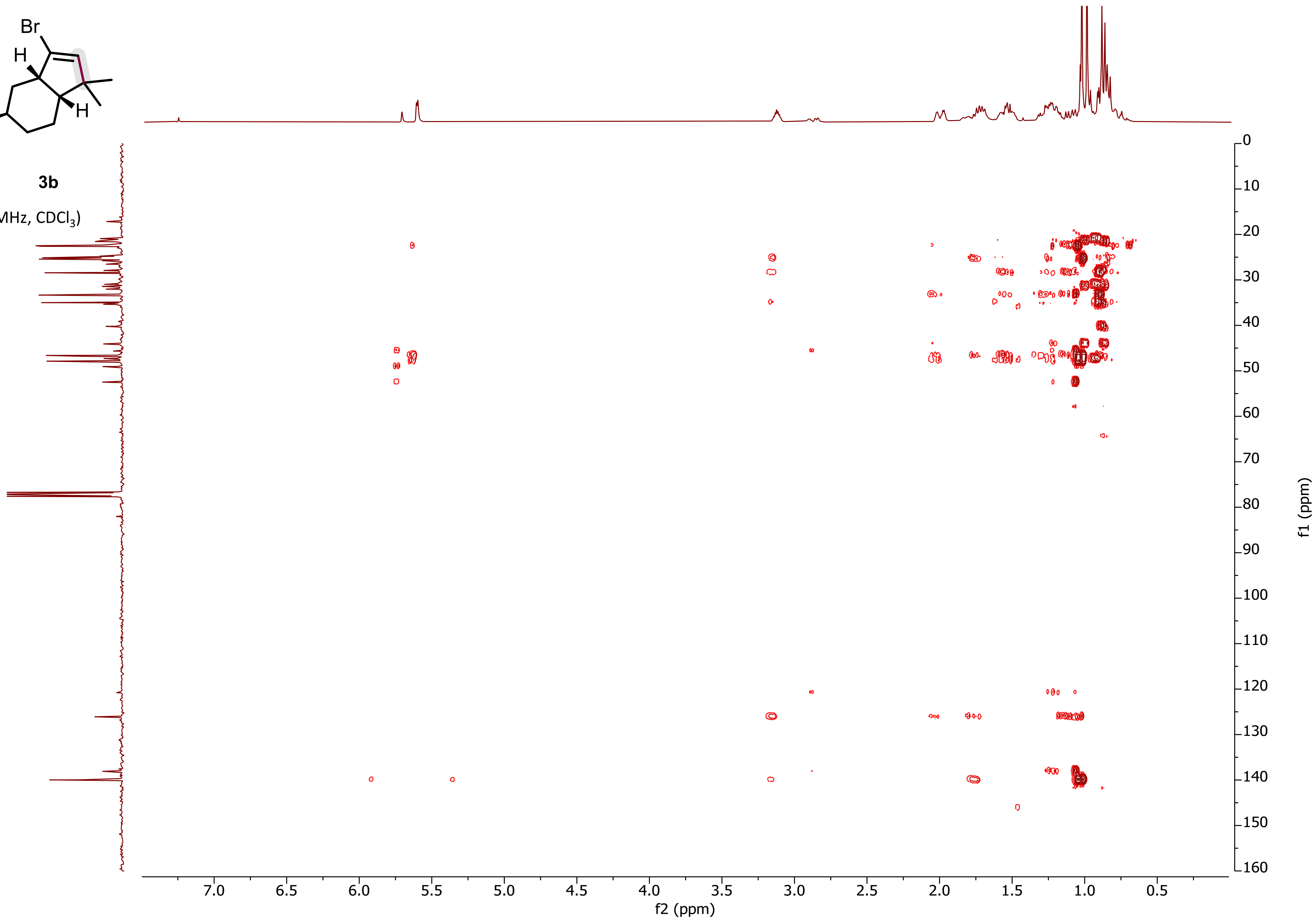

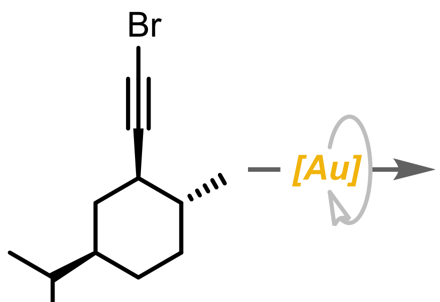
Br
[Au]

1c

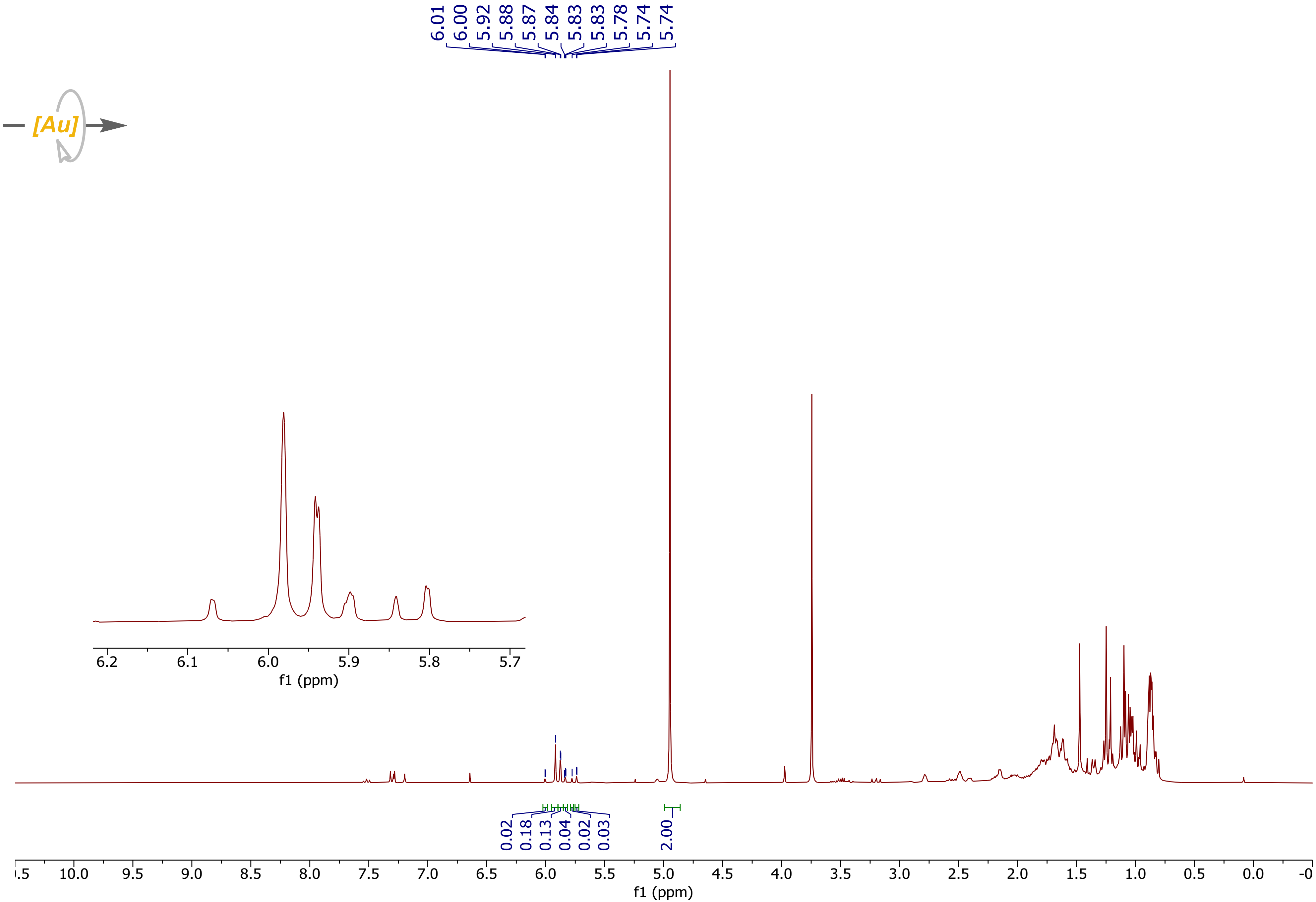
6.01
6.00
5.92
5.88
5.87
5.84
5.83
5.83
5.78
5.74
5.74
0.02
0.18
0.13
0.04
0.02
0.03
2.00
f1 (ppm)
f1 (ppm)

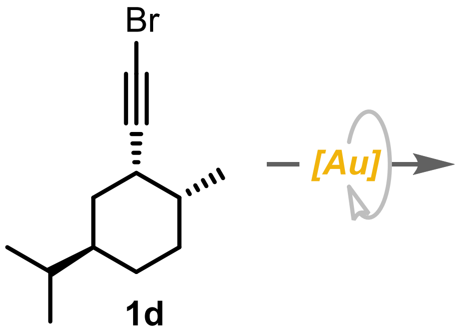
Br
[Au]
1d

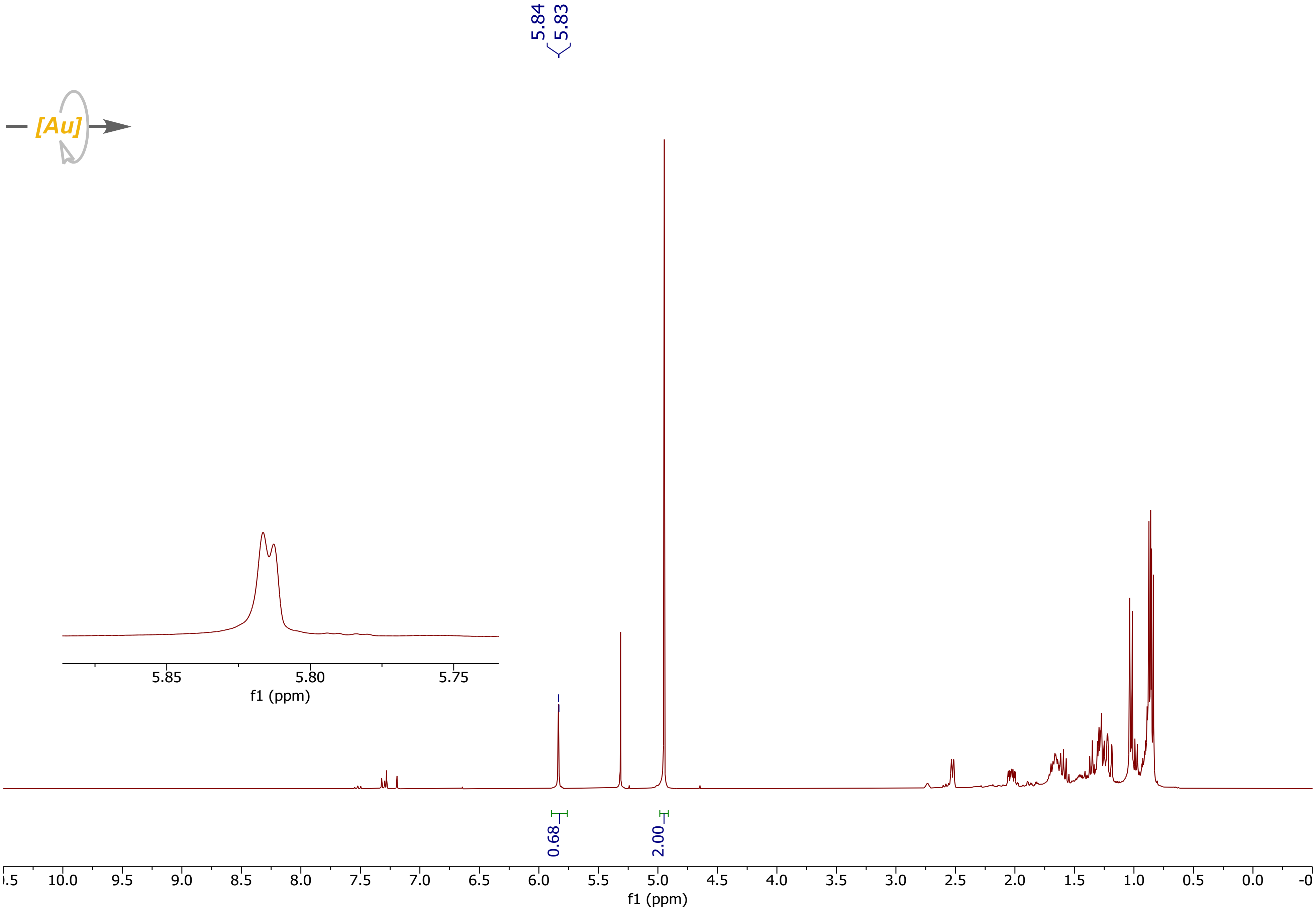
5.84
5.83
5.85
5.80
5.75
f1 (ppm)
0.68
2.00
10.0
9.5
9.0
8.5
8.0
7.5
7.0
6.5
6.0
5.5
5.0
4.5
4.0
3.5
3.0
2.5
2.0
1.5
1.0
0.5
0.0
f1 (ppm)

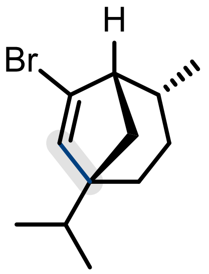

4d

^{1}H NMR(400 MHz, $CDCl_3$)

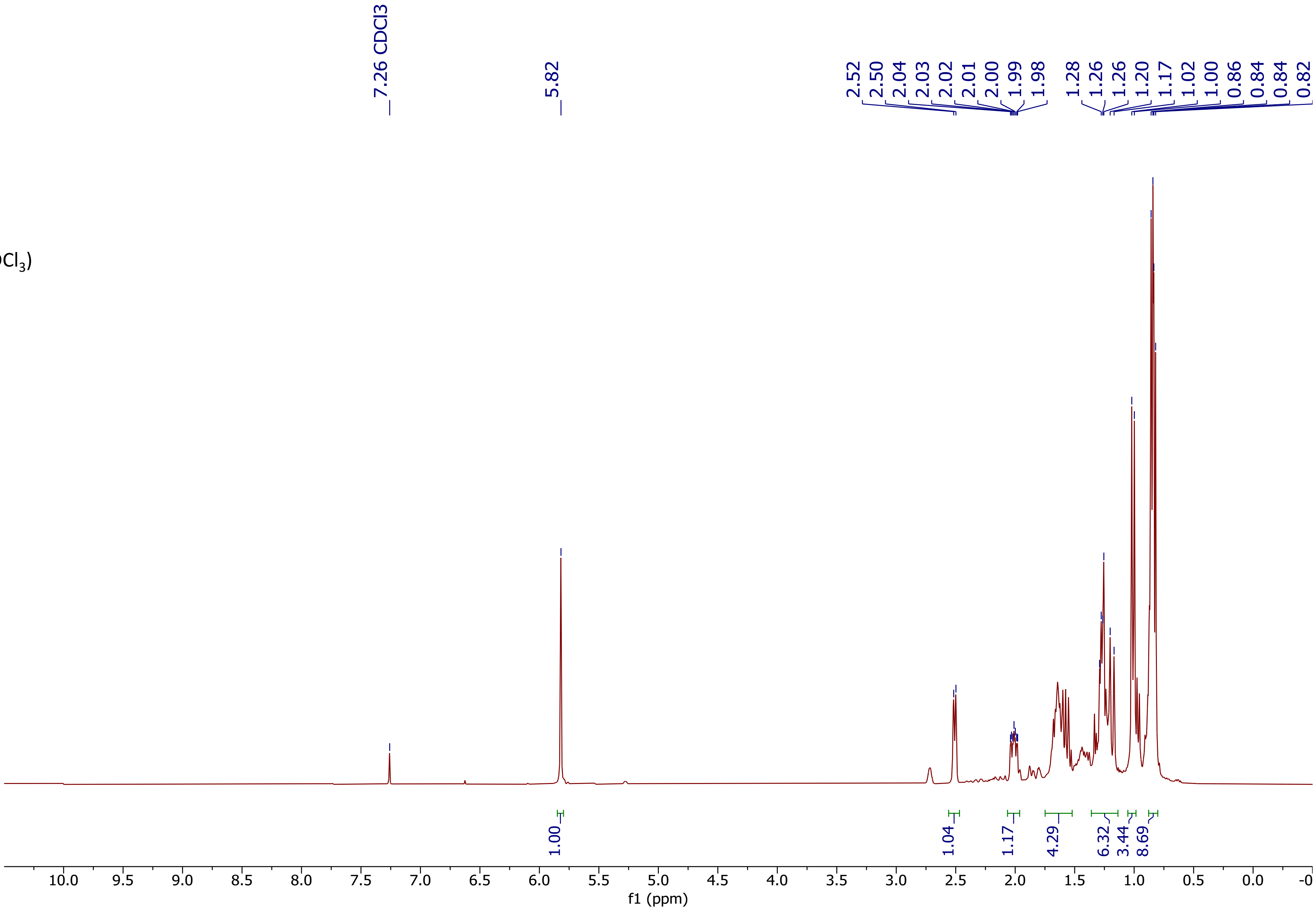

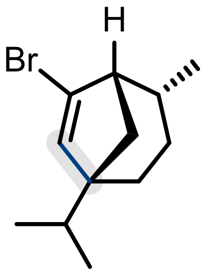

4d

^{13}C NMR (101 MHz, $CDCl_3$)

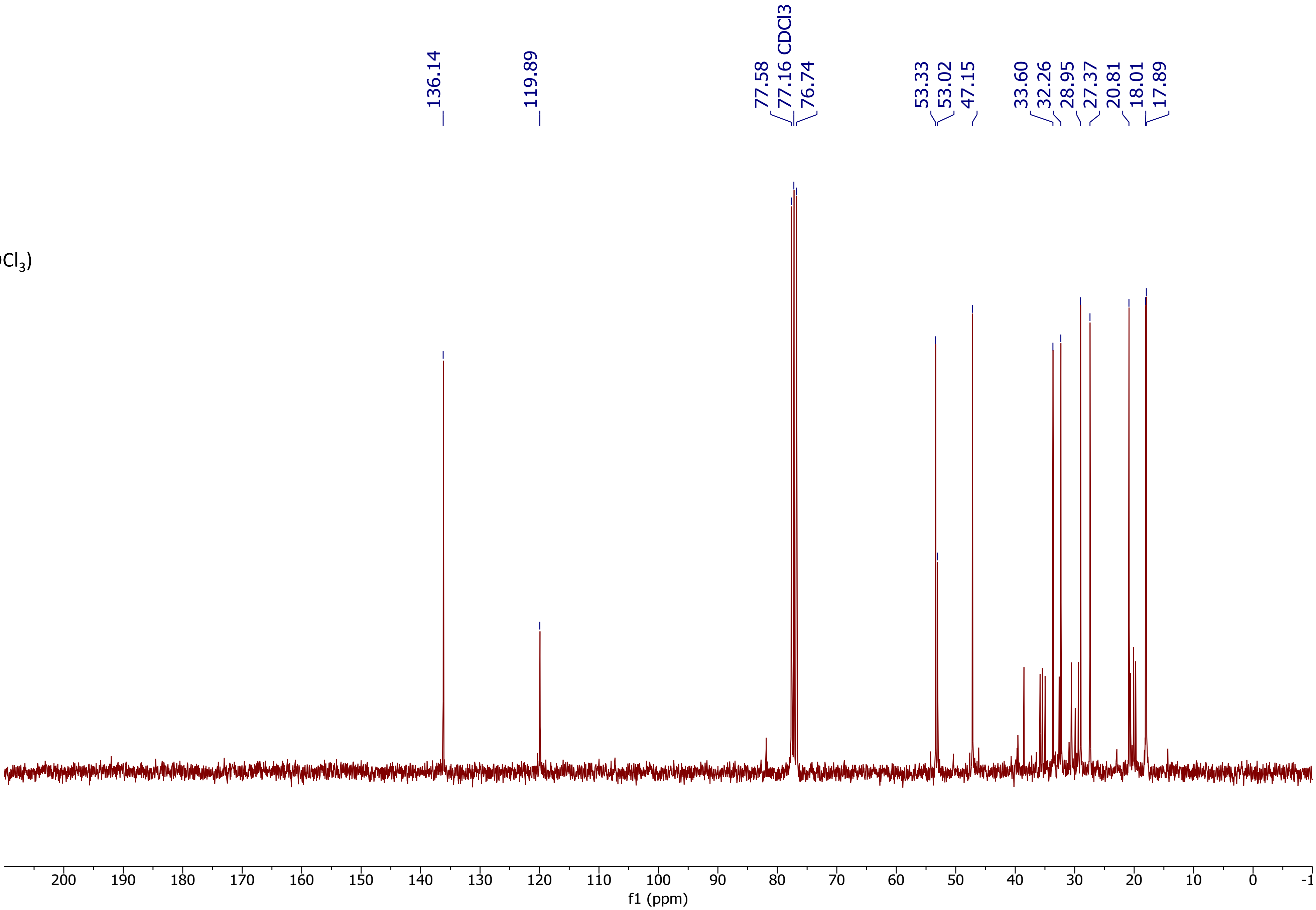

FUSED FAMILY

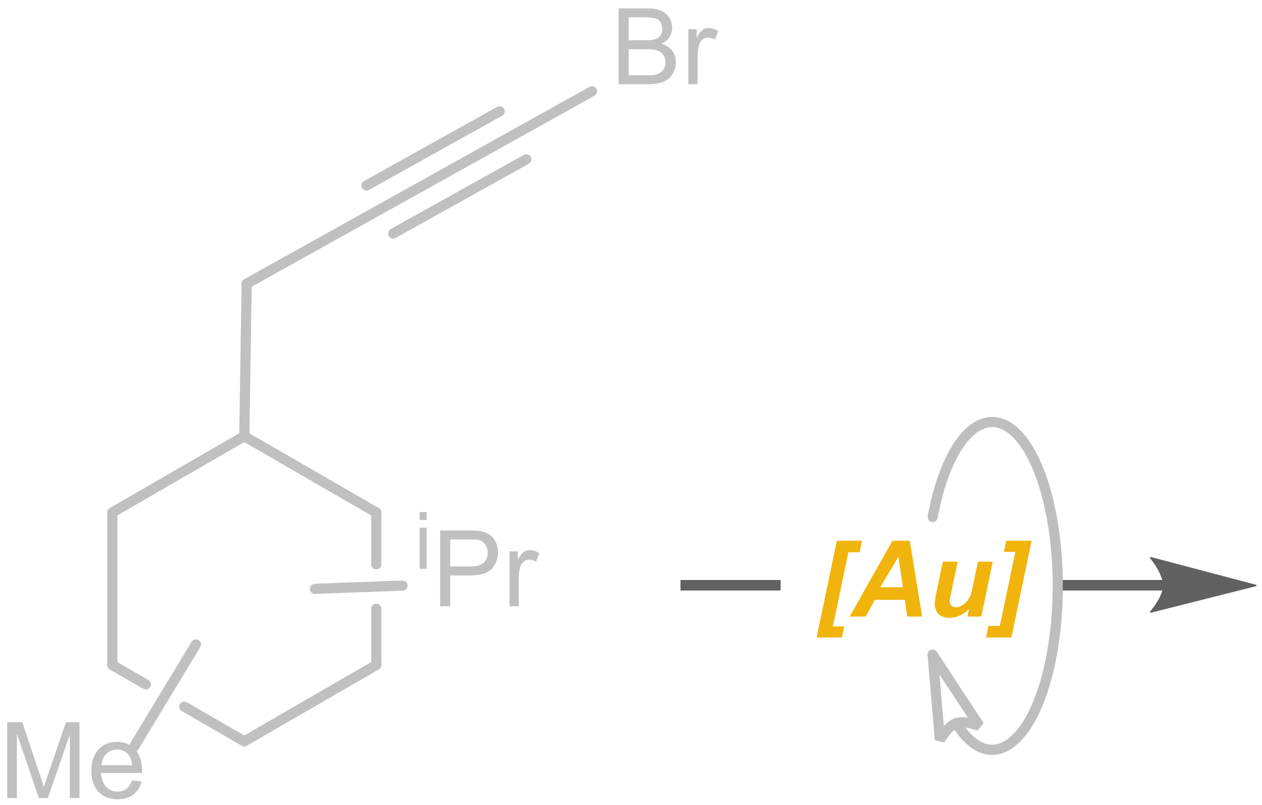

2a-d

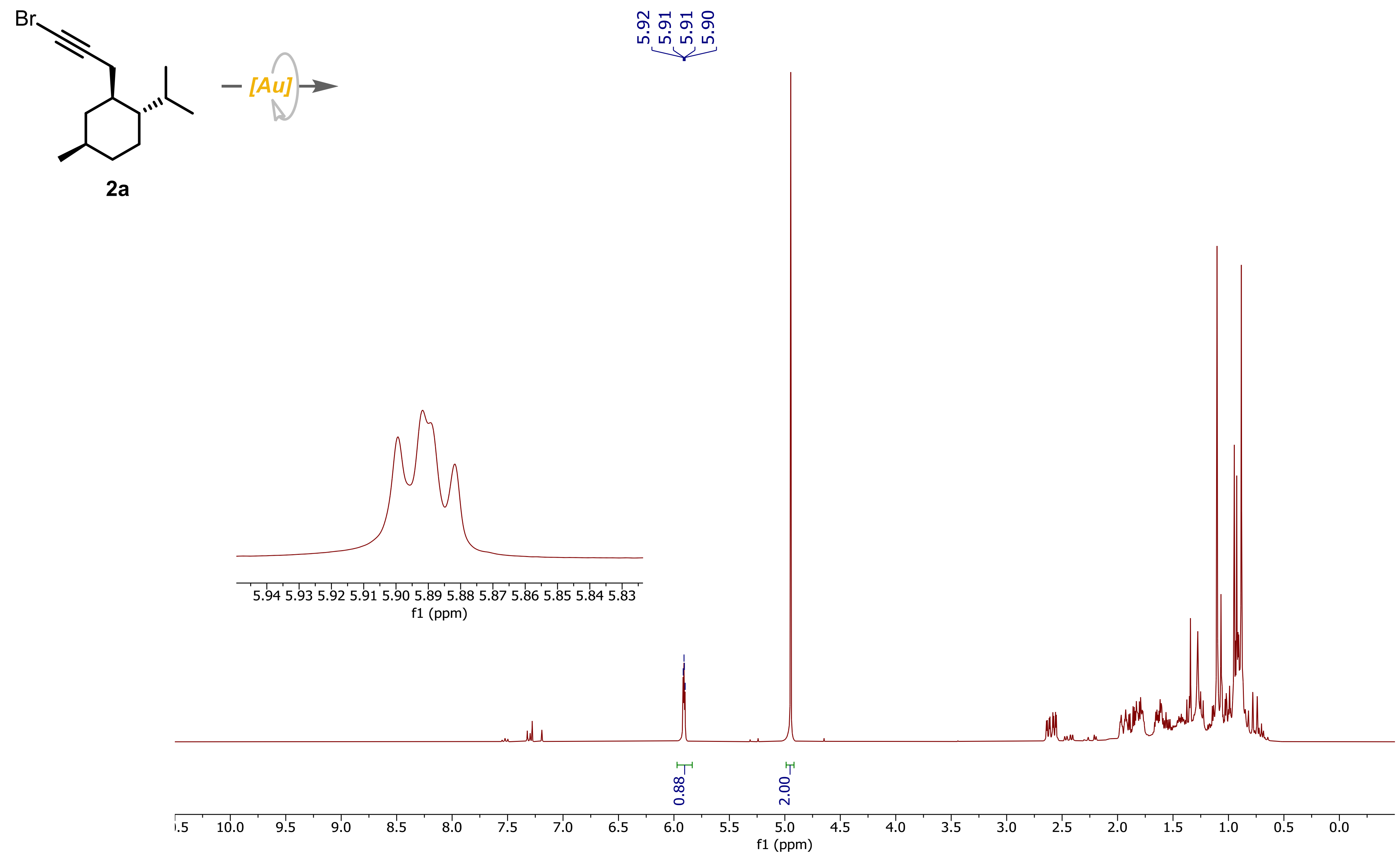
Br
[Au]
2a
5.92
5.91
5.91
5.90
5.94 5.93 5.92 5.91 5.90 5.89 5.88 5.87 5.86 5.85 5.84 5.83
f1 (ppm)
0.88
2.00
.5 10.0 9.5 9.0 8.5 8.0 7.5 7.0 6.5 6.0 5.5 5.0 4.5 4.0 3.5 3.0 2.5 2.0 1.5 1.0 0.5 0.0
f1 (ppm)

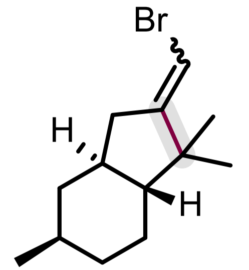

5a

^{1}H NMR(300 MHz, $CDCl_3$)

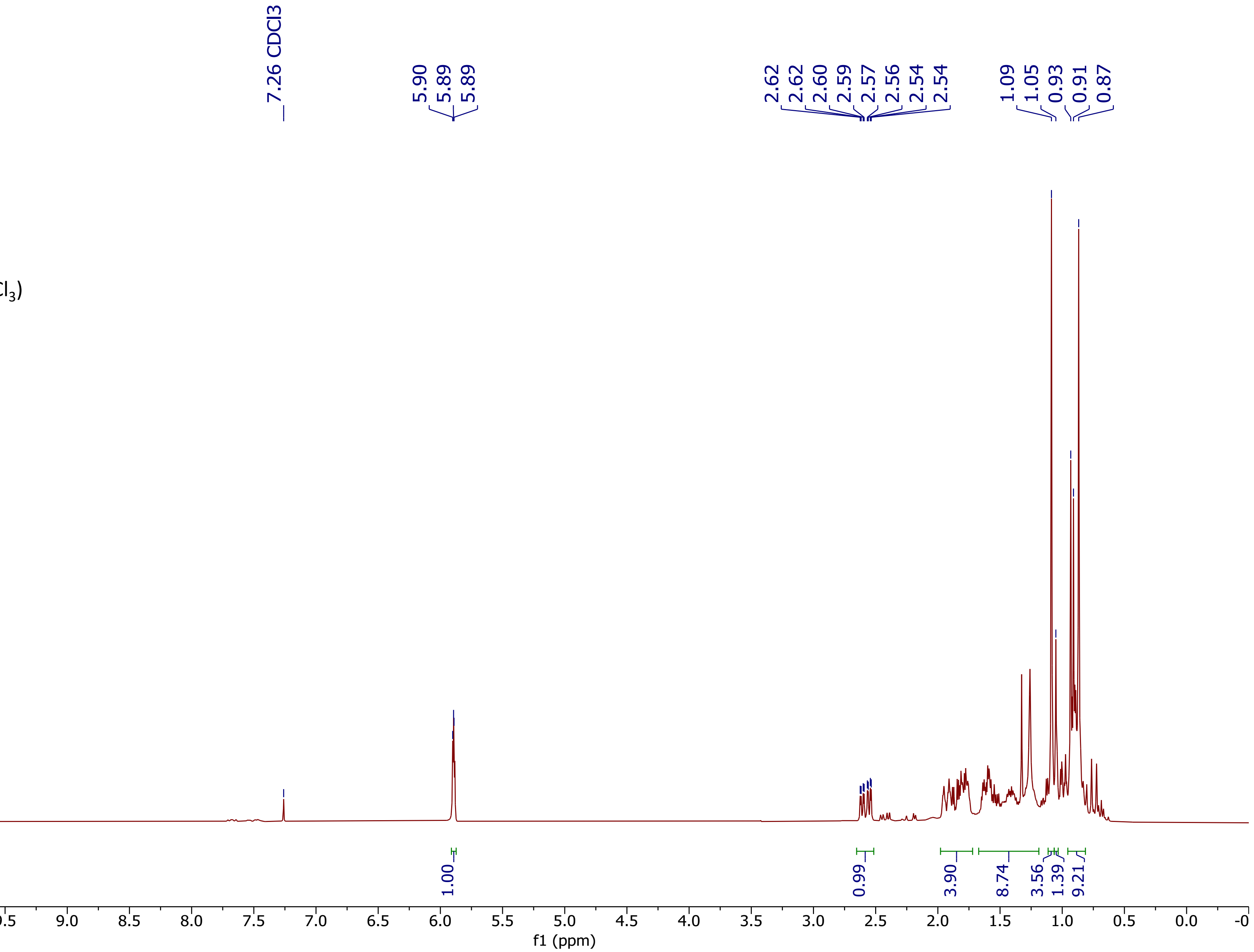

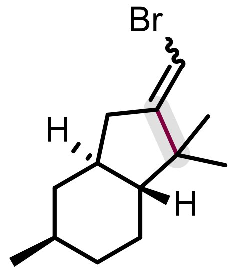

5a

^{13}C NMR (75 MHz, $CDCl_3$)

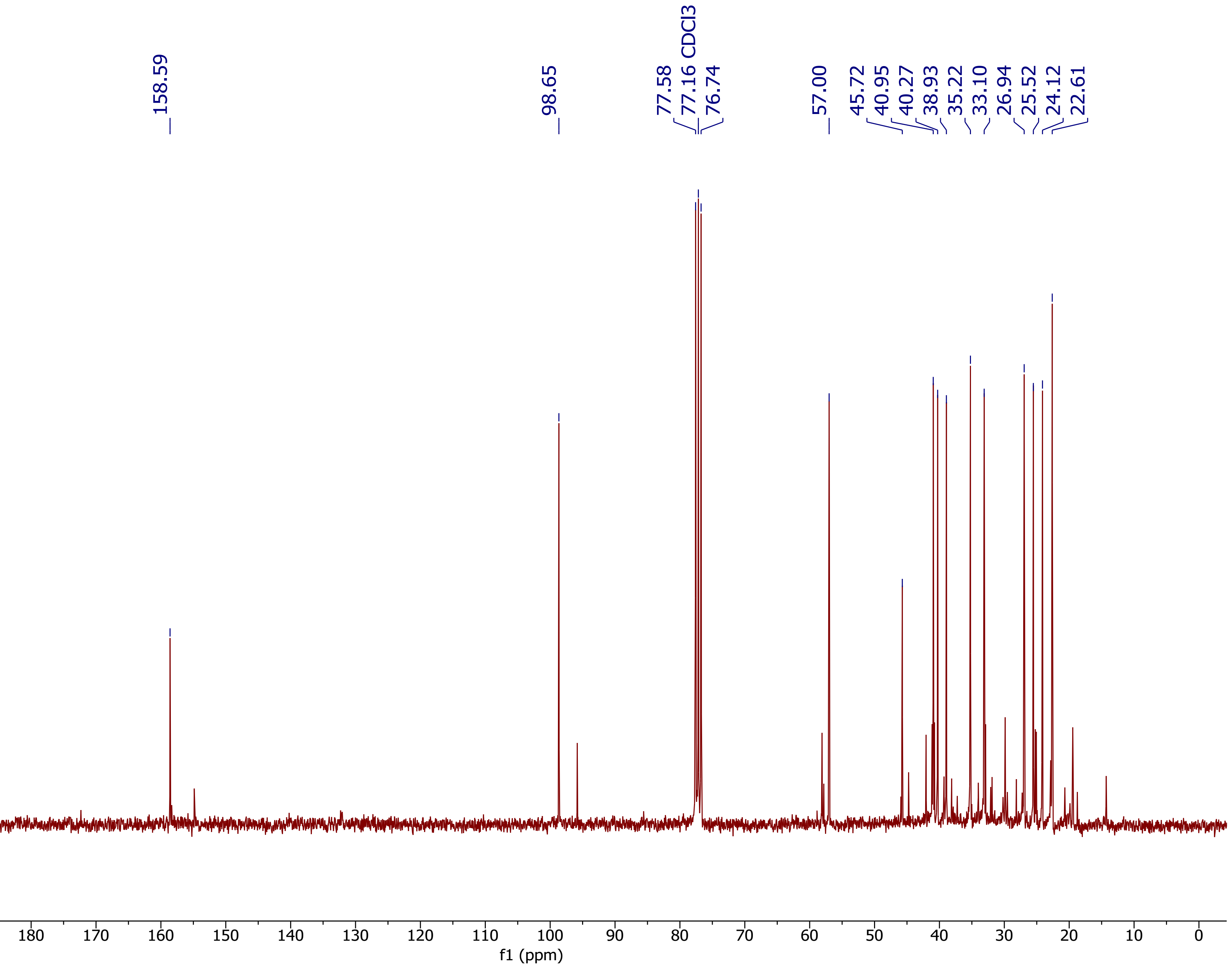

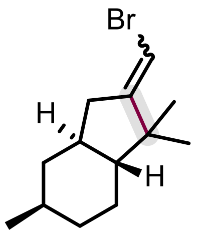

5a

COSY NMR([300, 300] MHz, $CDCl_3$)

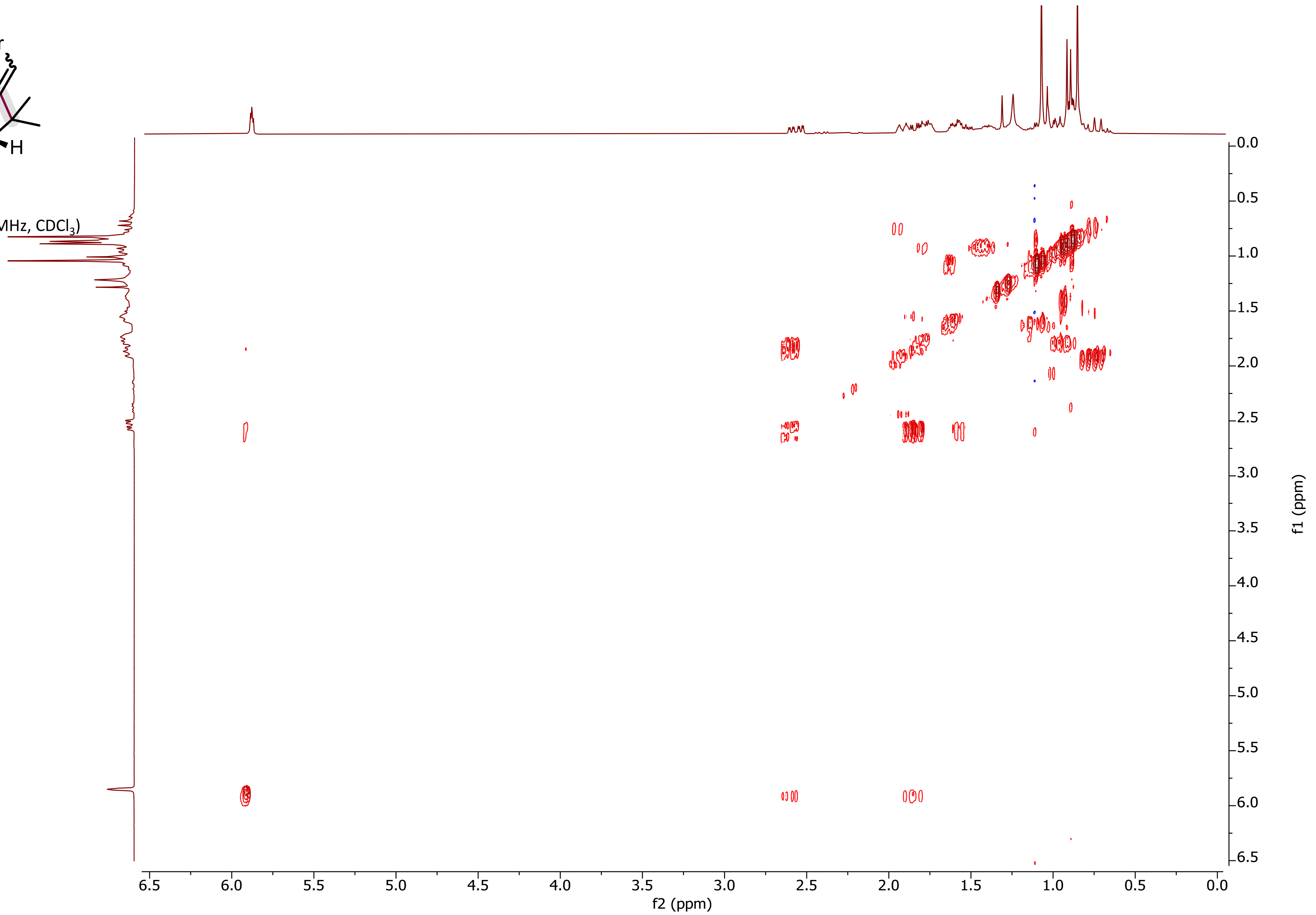

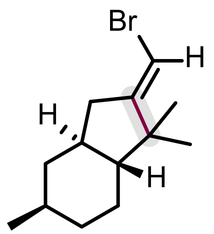

5a

HSQC NMR([300, 75] MHz, $CDCl_3$)

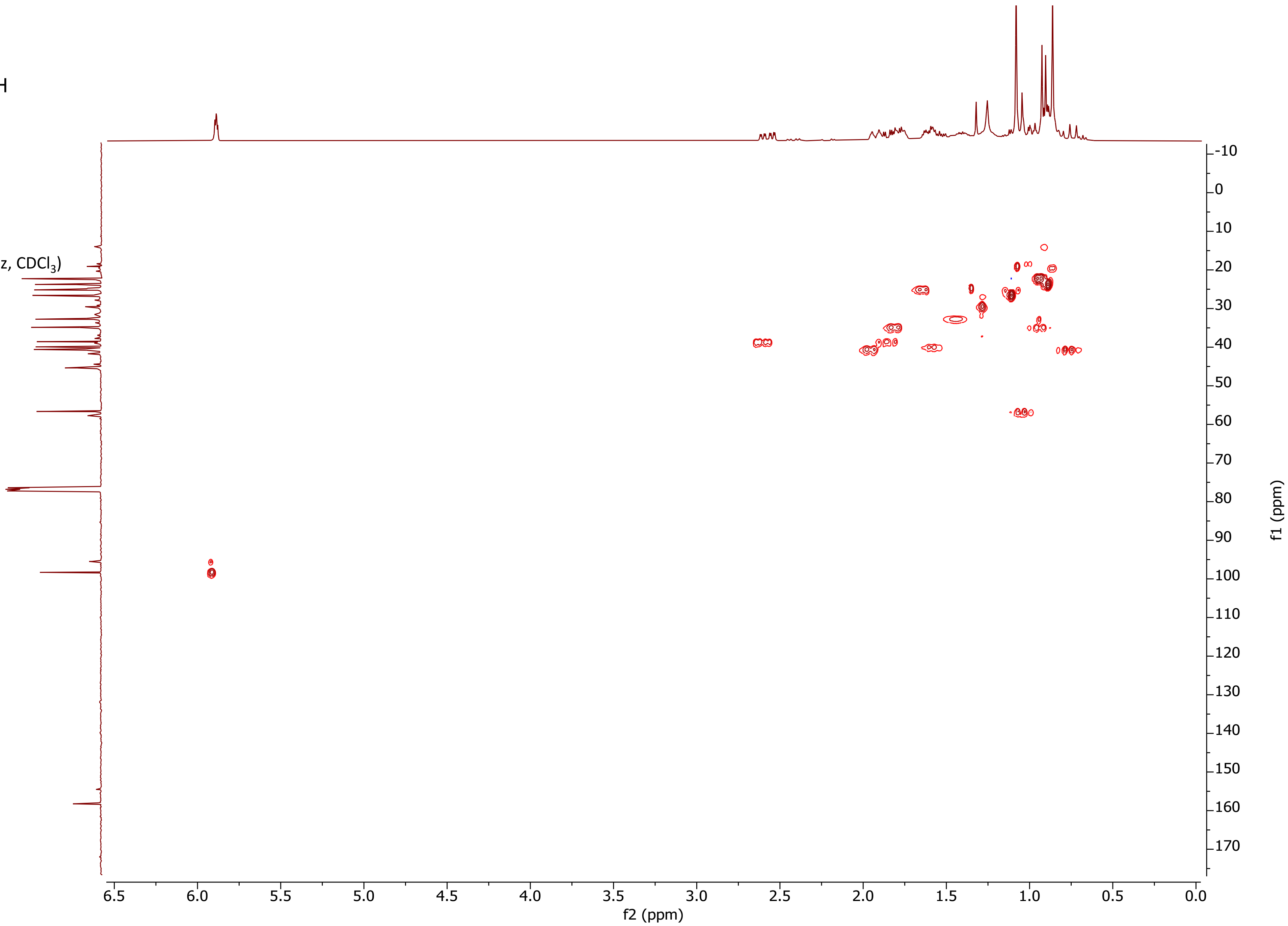

HMBC NMR([300, 75] MHz, $CDCl_3$)

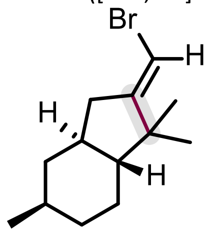

5a

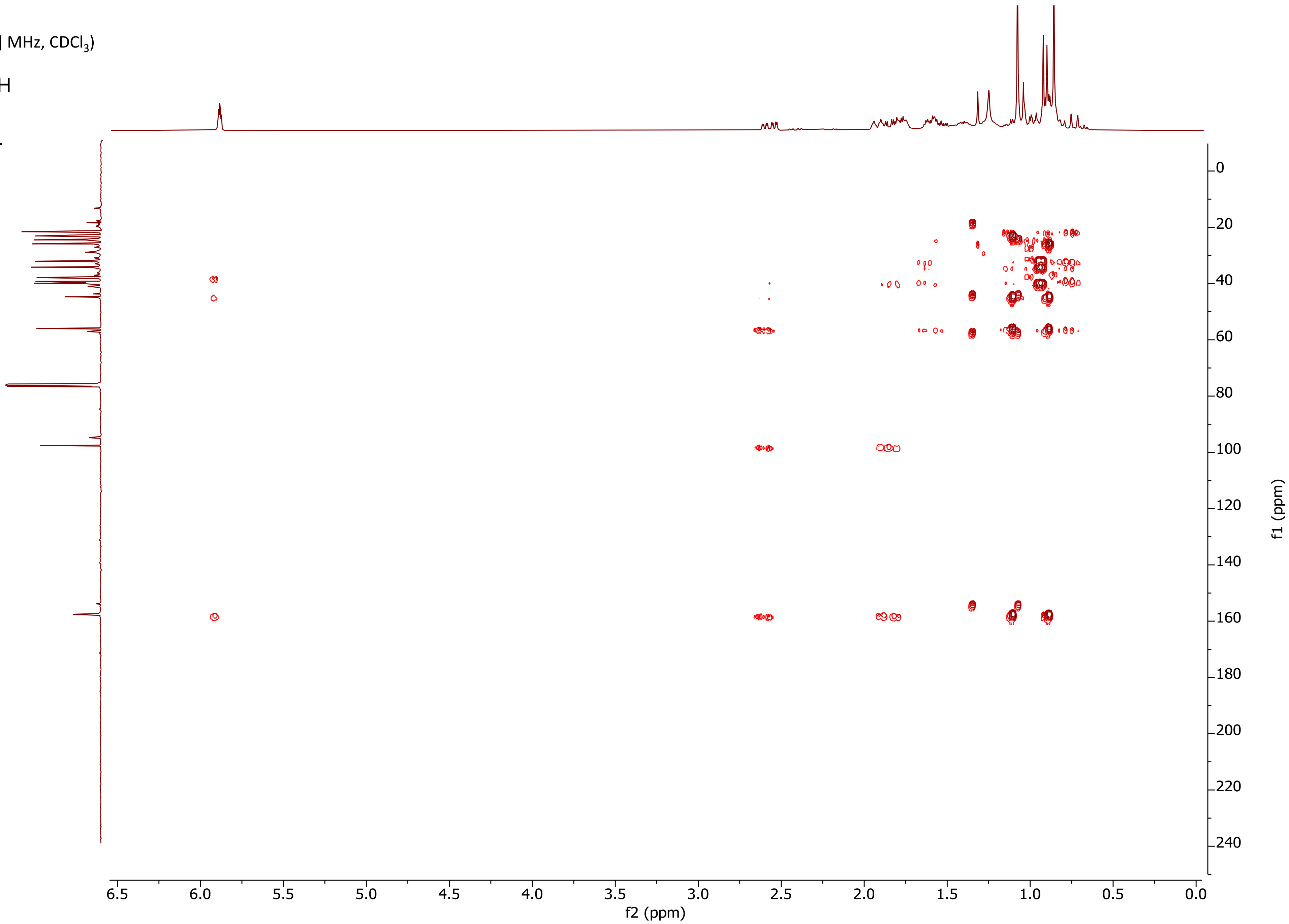

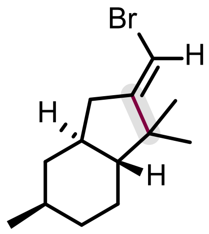

5a

NOESY NMR([300, 300] MHz, $CDCl_3$)

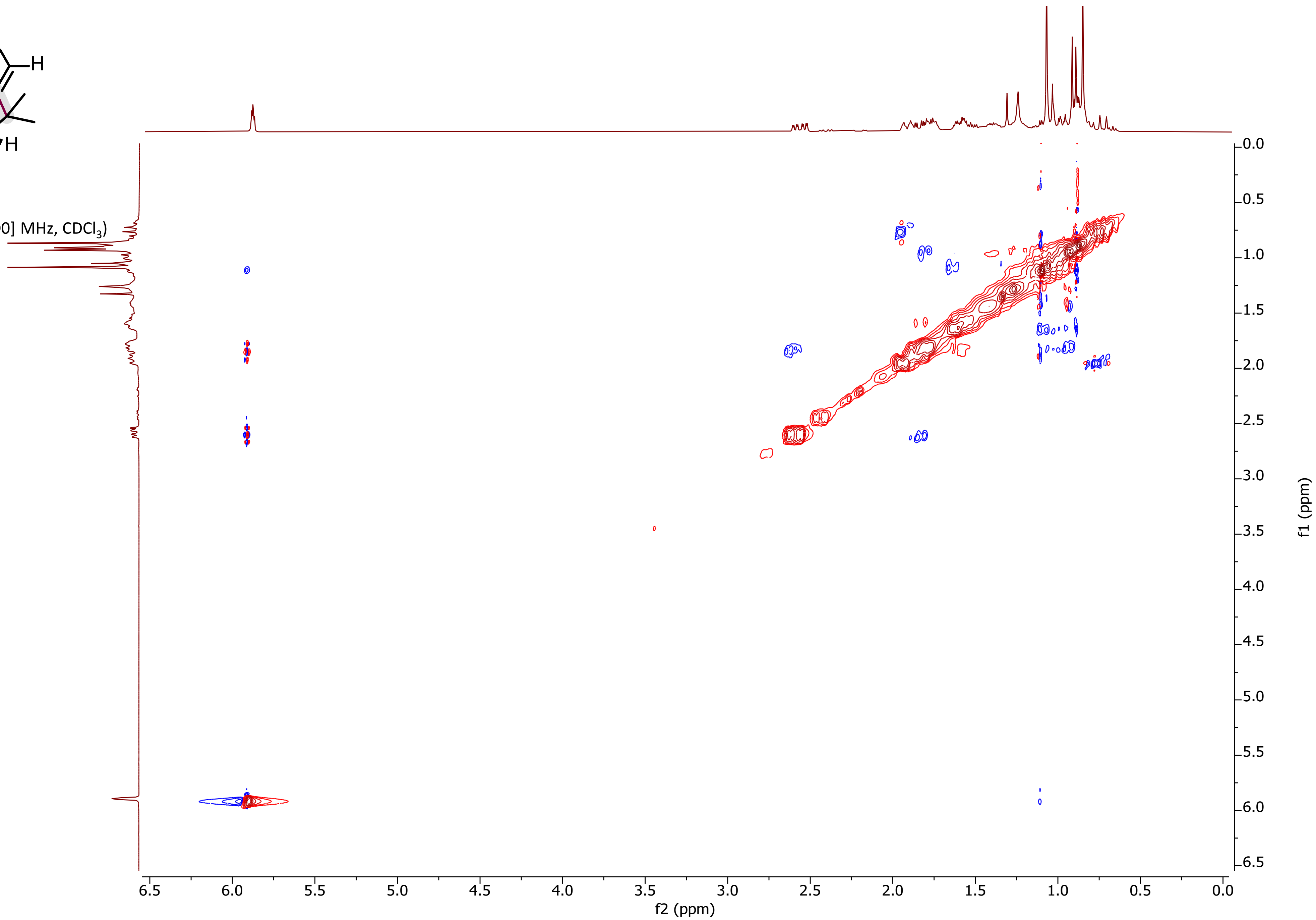

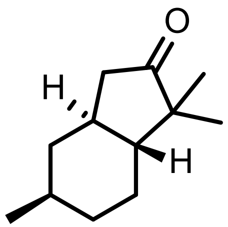

5a-CO

^{1}H NMR(300 MHz, $CDCl_3$)

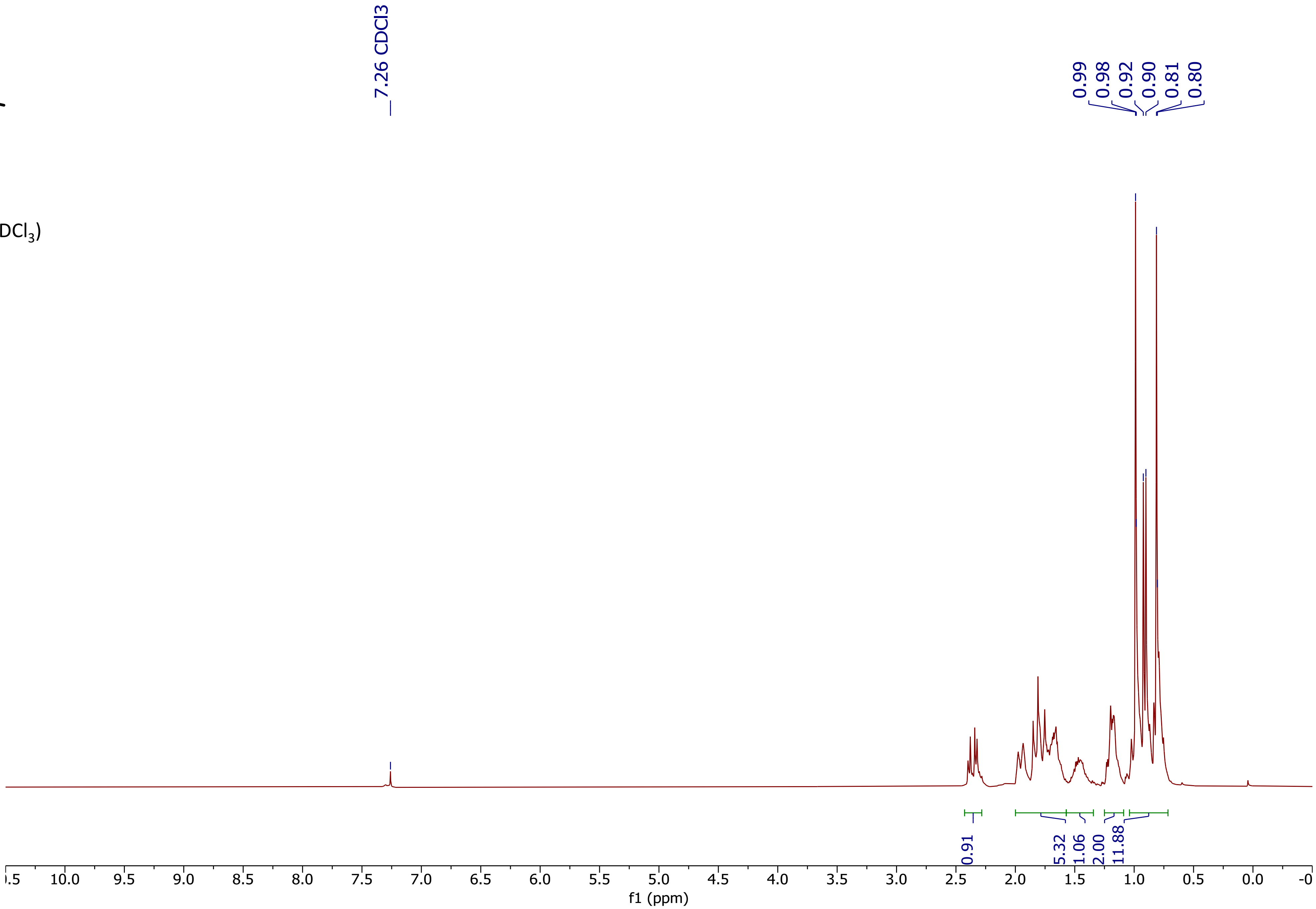

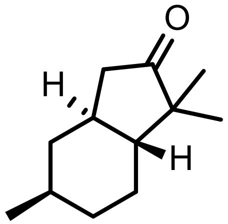

5a-CO

^{13}C NMR (75 MHz, $CDCl_3$)

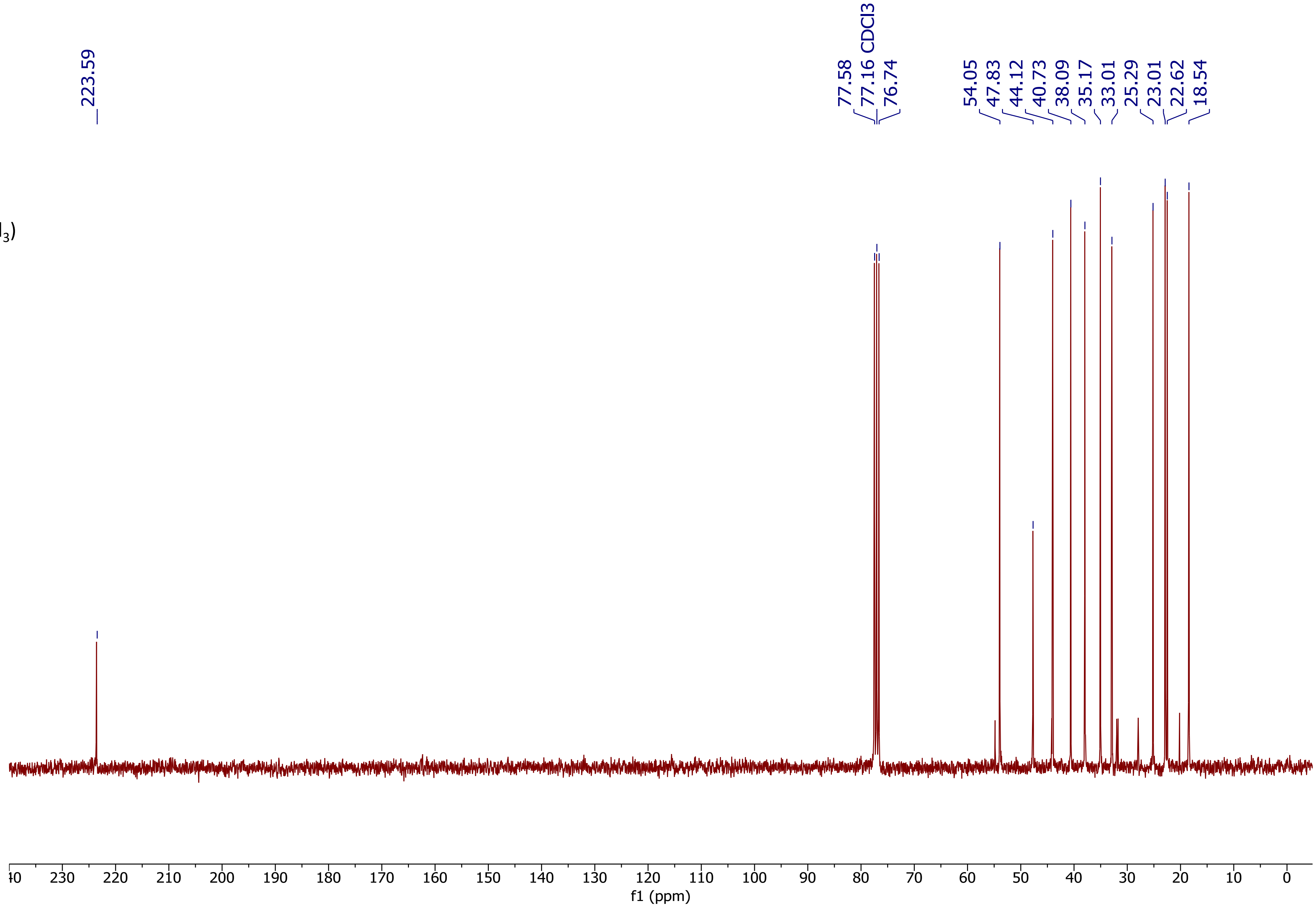

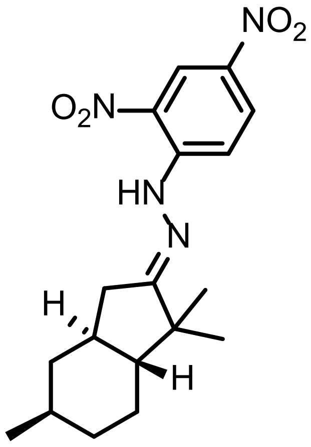

9

¹H NMR(300 MHz, $CDCl_3$)

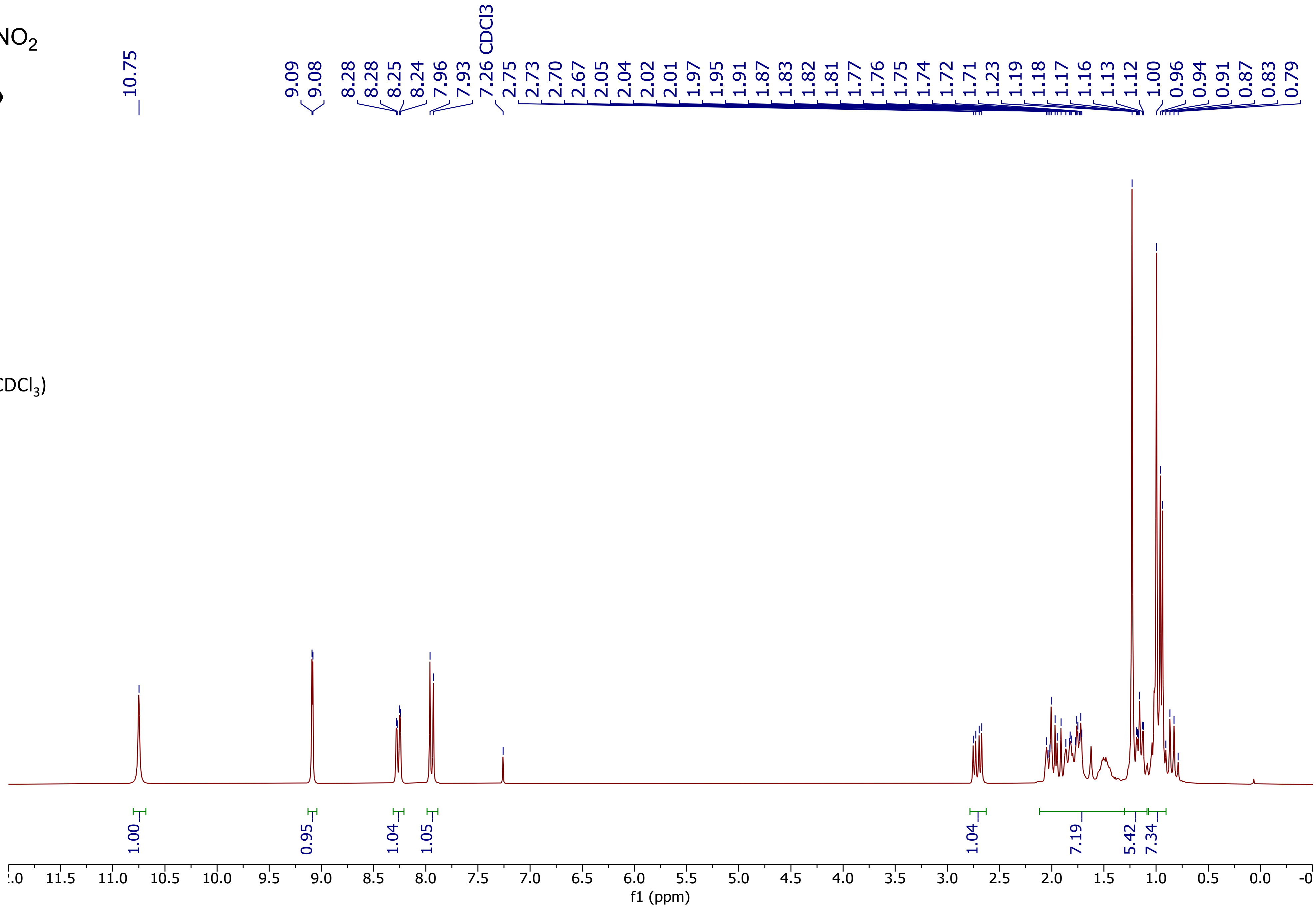

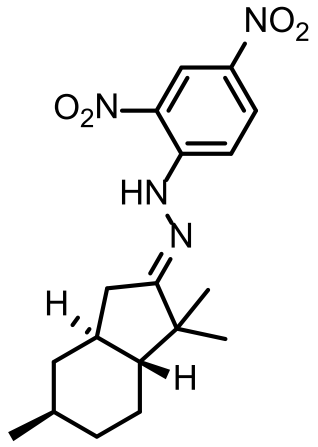

9

^{13}C NMR (75 MHz, $CDCl_3$)

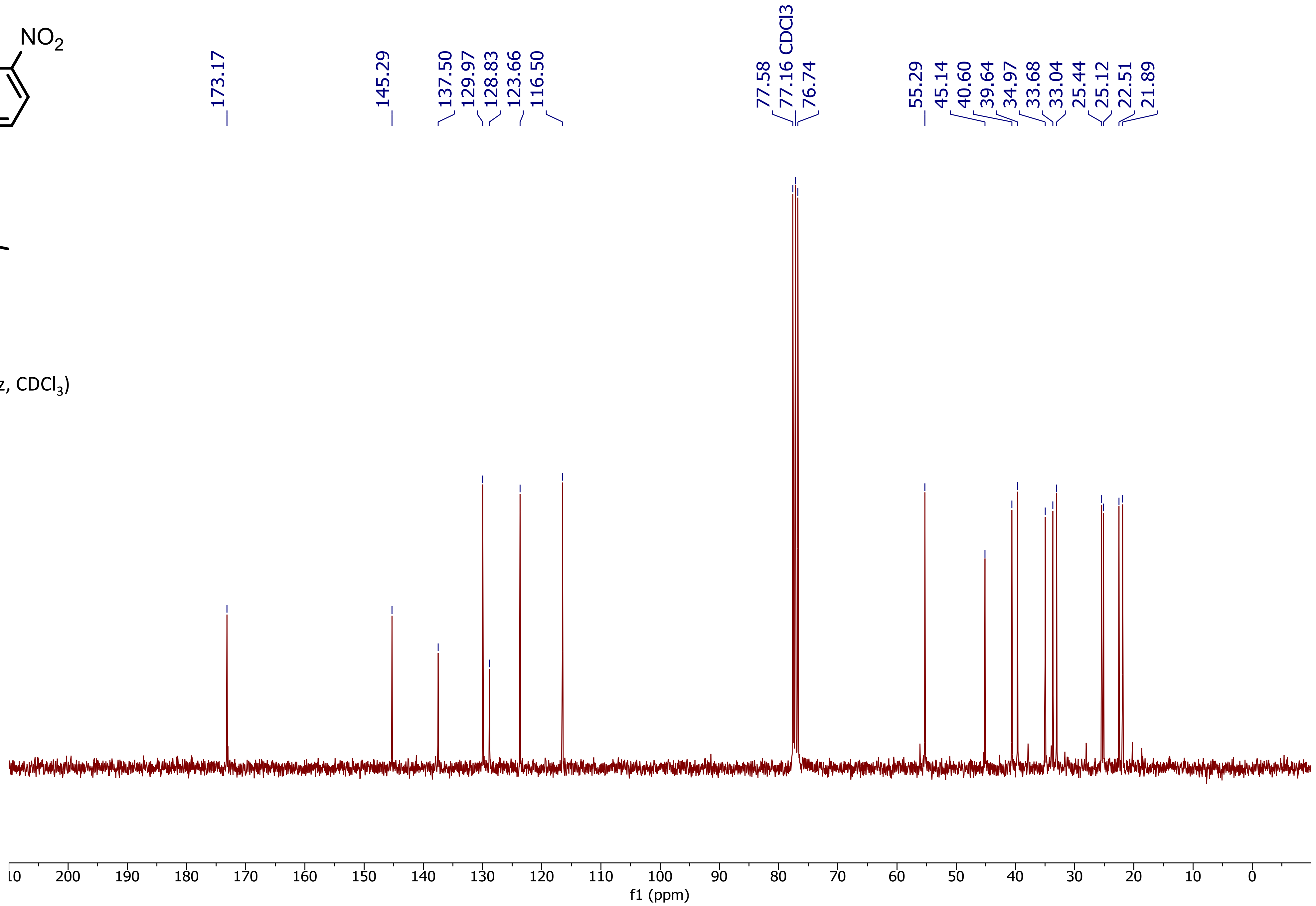

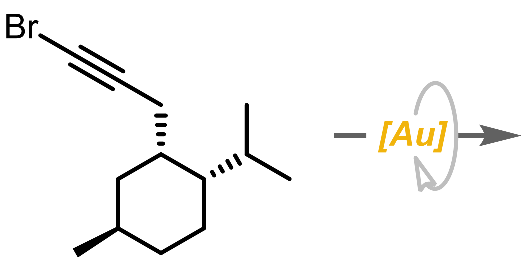
Br
[Au]

2b

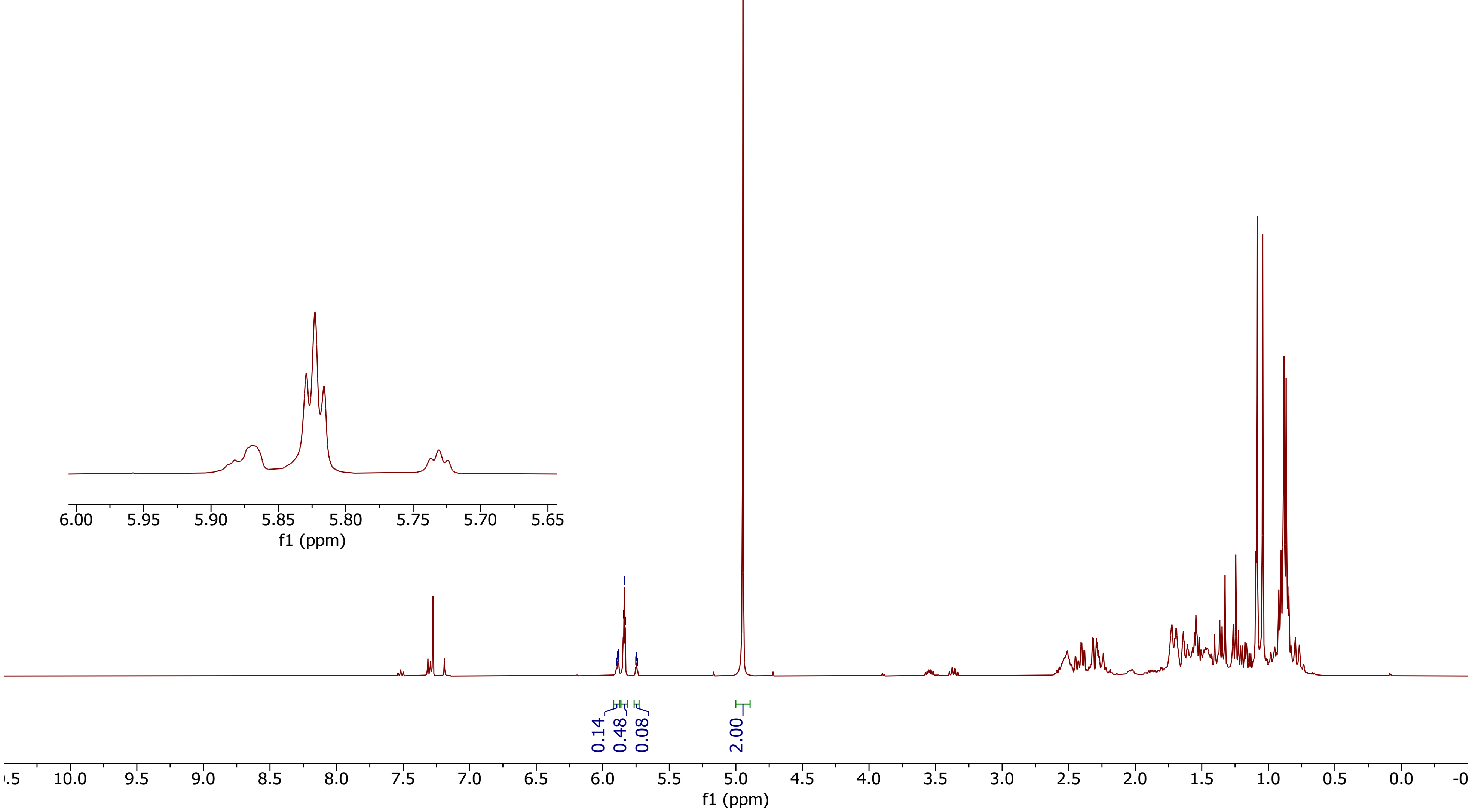
5.90
5.89
5.88
5.88
5.85
5.84
5.83
5.75
5.75
5.74
6.00
5.95
5.90
5.85
5.80
5.75
5.70
5.65
f1 (ppm)
0.14
0.48
0.08
2.00
.5
10.0
9.5
9.0
8.5
8.0
7.5
7.0
6.5
6.0
5.5
5.0
4.5
4.0
3.5
3.0
2.5
2.0
1.5
1.0
0.5
0.0
-0
f1 (ppm)

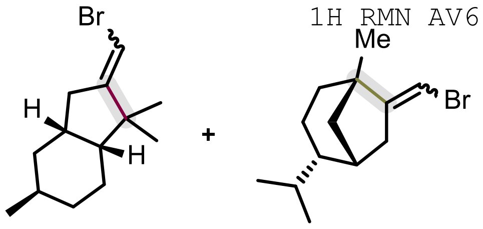

5b **6b**

1H RMN AV600

^{1}**H NMR**(600 MHz, $CDCl_3$)

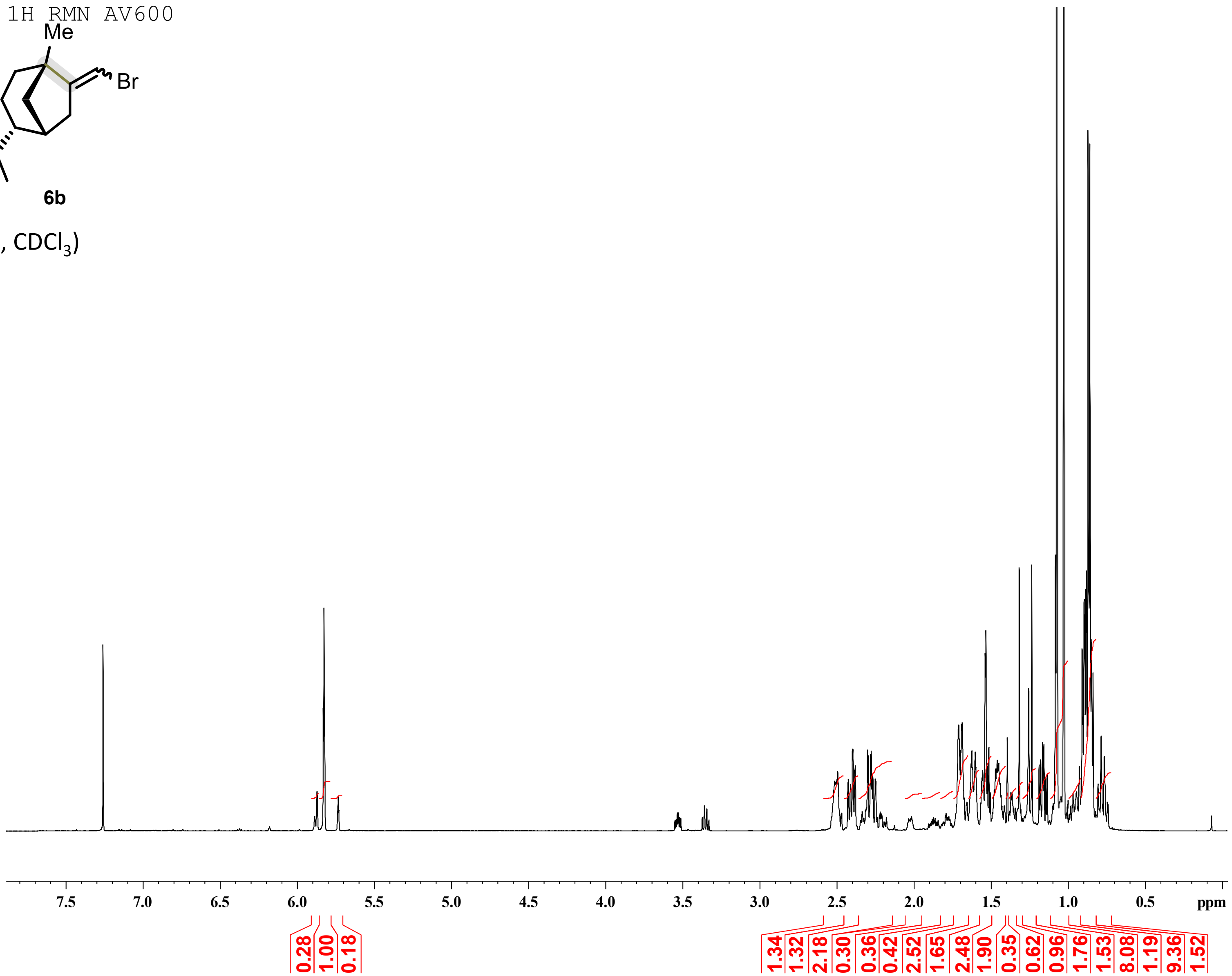

BRUKER

```
NAME              OAS-702F
EXPNO                    5
PROCNO                   1
Date_             2024120
Time              4
INSTRUM               2.18
PROBHD               spect
PULPROG    5 mm PATXI
TD                    1H/
SOLVENT               zg30
NS                   32768
DS                   CDCl3
SWH                   1 6
FIDRES                   0
AQ             6613.757 Hz
RG             0.201836 Hz
DW            2.4773865
DE                     sec
TE                    71.8
D1              75.600 usec
TD0               6.00 usec
                   298.2 K
======== CHANNEL f1 ========
NUC1                  1H sec
P1                 8.60 usec
PL1                2.00 dB
PL1W         15.84893227 W
SFO1         600.1528884
SI                     MHz
SF                   32768
WDW          600.1500165 MHz
SSB                      no
LB                        0
GB                     0.00
PC                       Hz
                          0
                       1.00
```

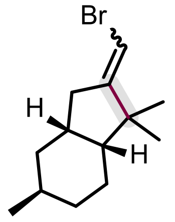

5b

+

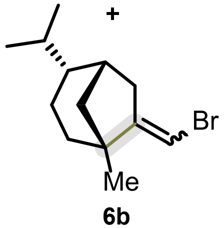

6b

^{1}H NMR(600 MHz, $CDCl_3$)

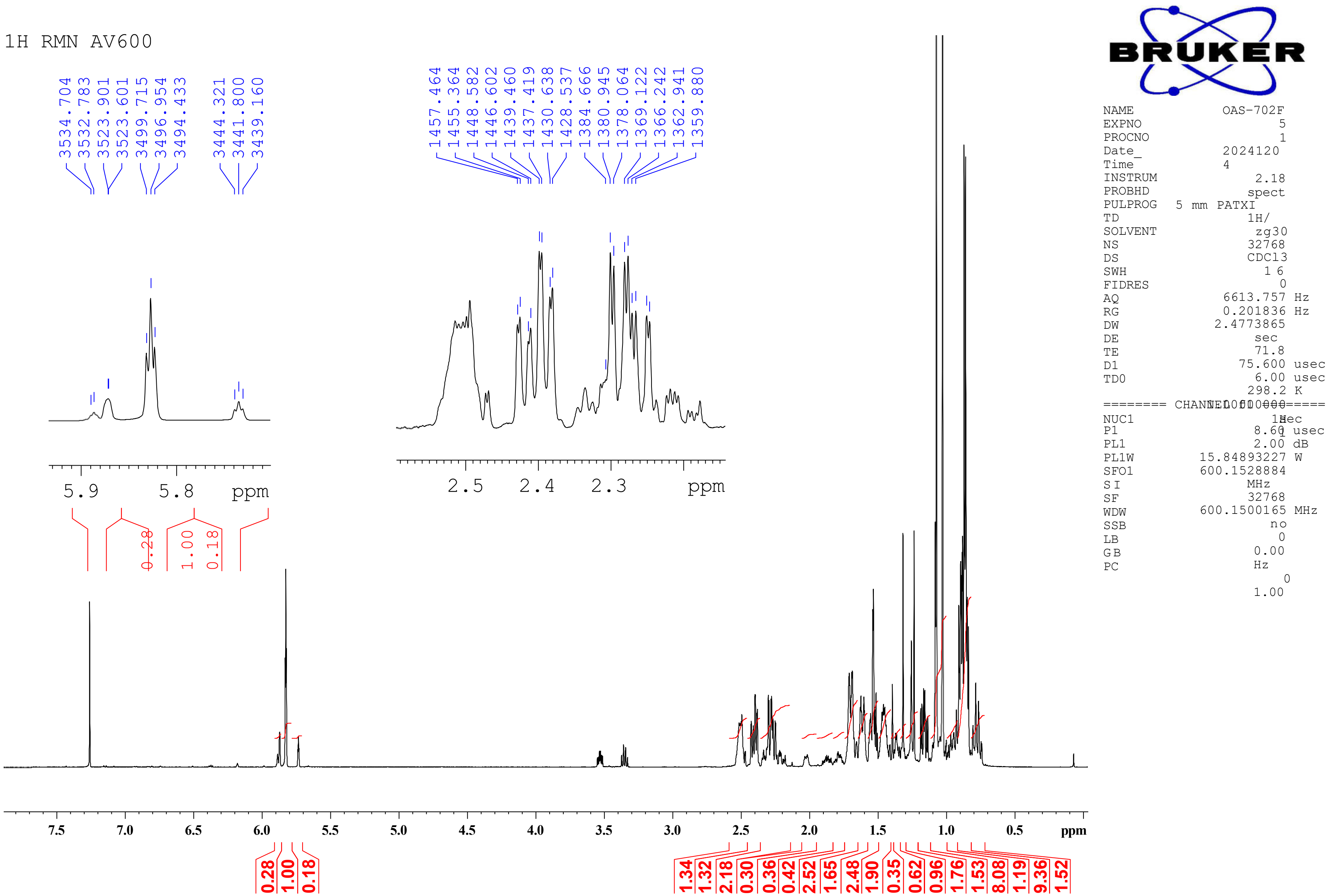

1H RMN AV600

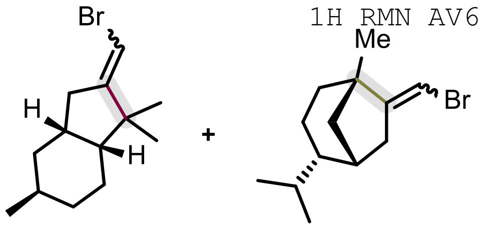

5b **6b**

^{1}H NMR(600 MHz, $CDCl_3$)

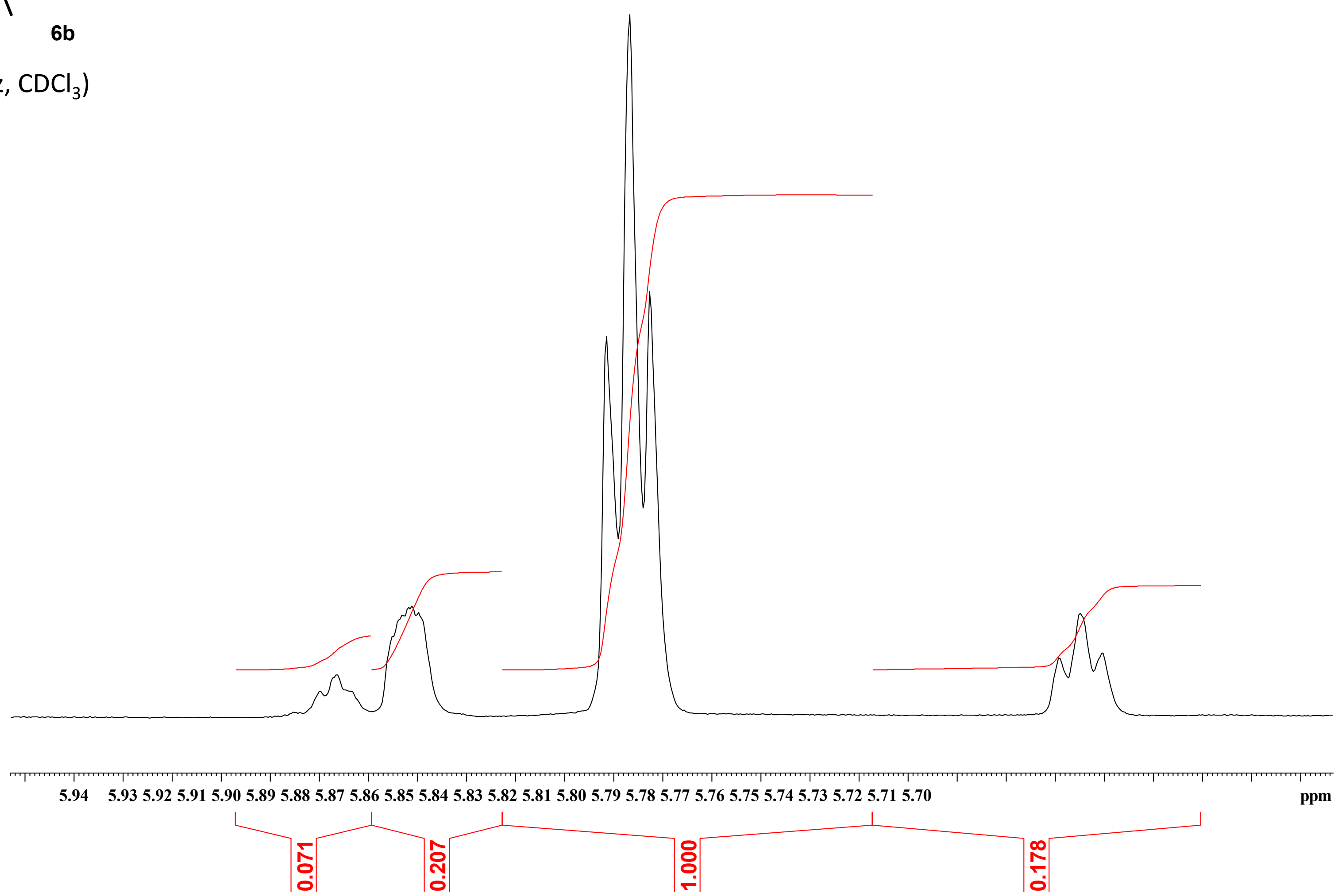


```
NAME                OAS-702F
EXPNO                      5
PROCNO                     2
Date_               2024120
Time                4
INSTRUM                 2.18
PROBHD                 spect
PULPROG   5 mm PATXI
TD                       1H/
SOLVENT                 zg30
NS                     32768
DS                     CDCl3
SWH                      1 6
FIDRES                     0
AQ                  6613.757 Hz
RG                  0.201836 Hz
DW                 2.4773865
DE                       sec
TE                      71.8
D1                    75.600 usec
TD0                     6.00 usec
                       298.2 K
======== CHANNEL f1 ========
NUC1                     1H
P1                      8.60 usec
PL1                     2.00 dB
PL1W             15.84893227 W
SFO1             600.1528884
SI                       MHz
SF                     32768
WDW              600.1500165 MHz
SSB                       GM
LB                         0
GB                     -0.50 Hz
PC                       0.5
                        1.00
```

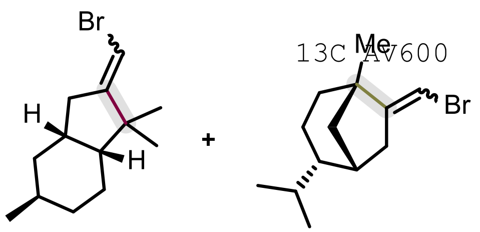

5b **6b**

^{13}C NMR (150 MHz, $CDCl_3$)

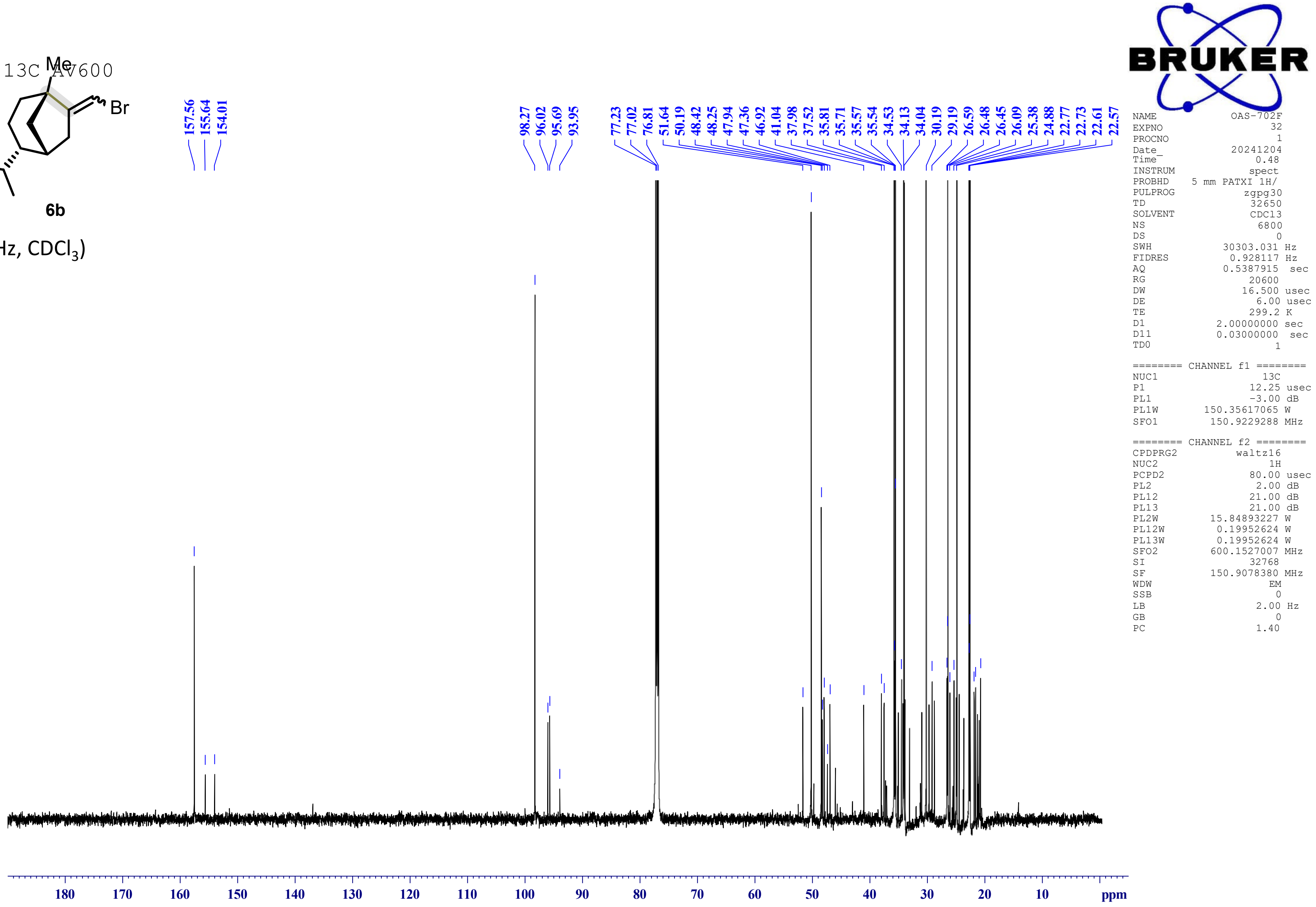

DEPT3 AV600

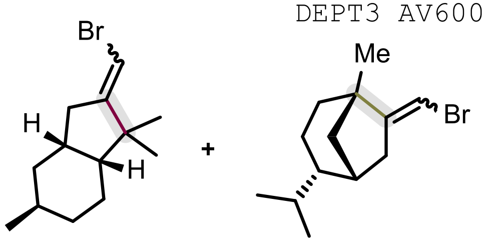

5b **6b**

DEPT-135 NMR (150 MHz, $CDCl_3$)

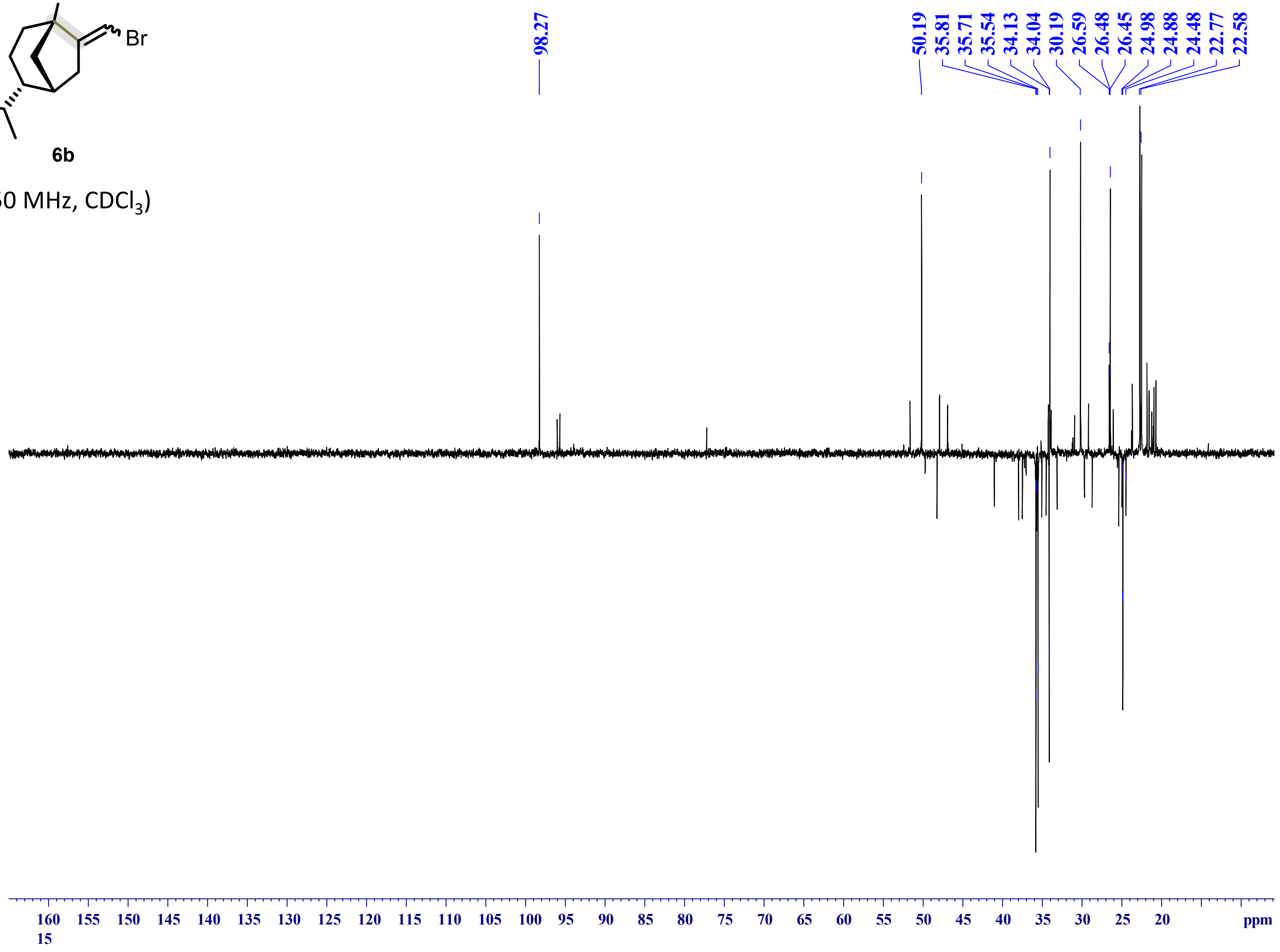


```
NAME          OAS-702F
EXPNO                2
PROCNO               1
Date_         20241203
Time             19.53
INSTRUM          spect
PROBHD   5 mm PATXI 1H/
PULPROG        dept135
TD               32768
SOLVENT          CDCl3
NS                2000
DS                   0
SWH          26041.666 Hz
FIDRES        0.794729 Hz
AQ           0.6292148 sec
RG               20600
DW              19.200 usec
DE                6.00 usec
TE               299.2 K
CNST2      145.0000000
D1          2.00000000 sec
D2          0.00344828 sec
D12         0.00002000 sec
TD0                  1

======== CHANNEL f1 ========
NUC1               13C
P1               12.25 usec
P2               24.50 usec
PL1              -3.00 dB
PL1W      150.35617065 W
SFO1       150.9206652 MHz

======== CHANNEL f2 ========
CPDPRG2        waltz16
NUC2                1H
P3                9.00 usec
P4               18.00 usec
PCPD2            80.00 usec
PL2               2.00 dB
PL12             21.00 dB
PL2W       15.84893227 W
PL12W       0.19952624 W
SFO2       600.1524010 MHz
SI               32768
SF         150.9078380 MHz
WDW                 EM
SSB                  0
LB                2.00 Hz
GB                   0
PC                1.40
```

13C/DEPT-135 NMR ([150, 150] MHz, $CDCl_3$)

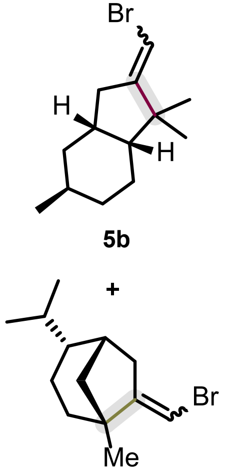

5b

+

6b

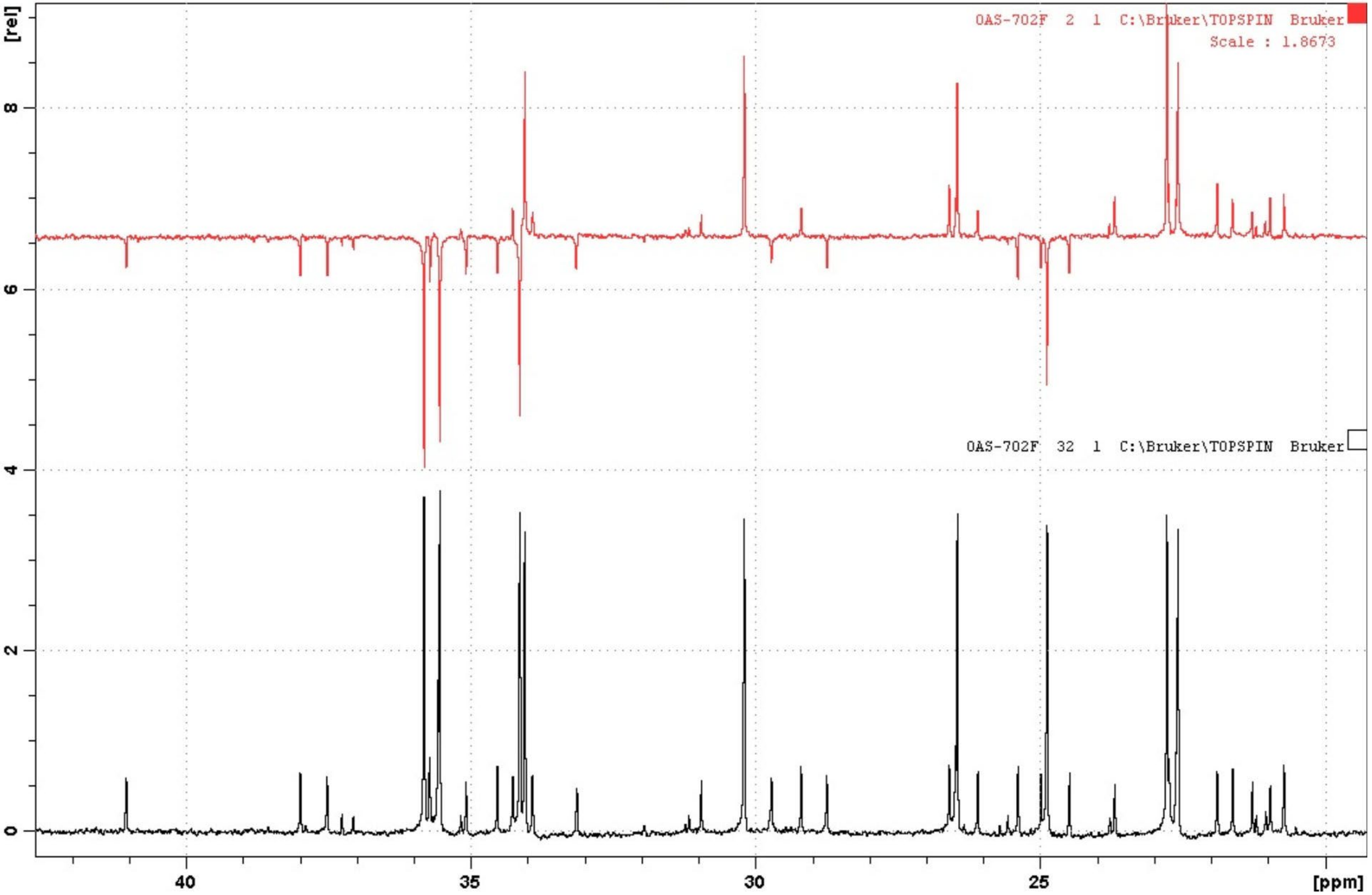

COSY NMR([600, 600] MHz, $CDCl_3$)

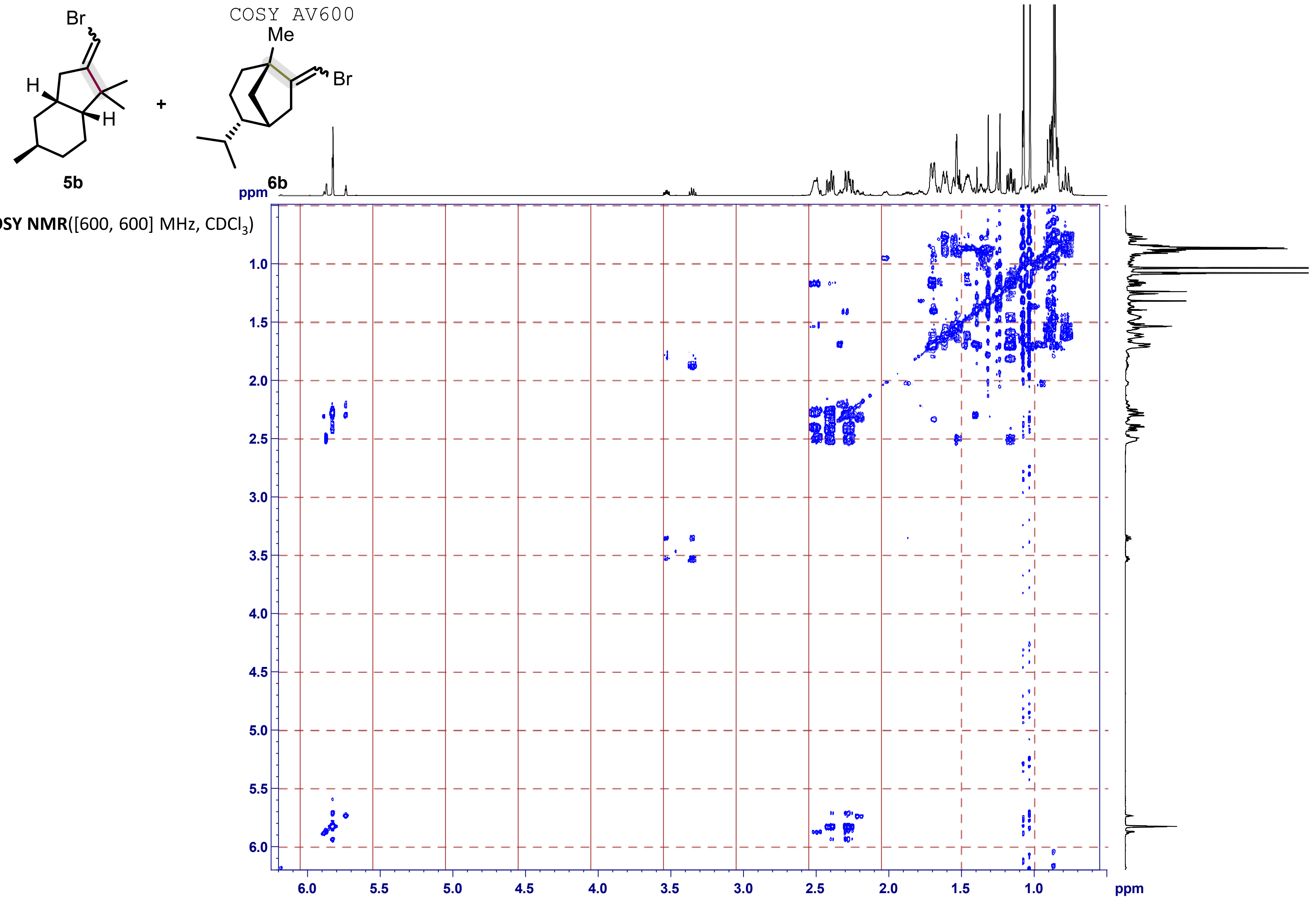


```
NAME           OAS-702F
EXPNO                 7
PROCNO                1
Date_          20241204
Time              11.17
INSTRUM           spect
PROBHD   5 mm PATXI 1H/
PULPROG        cosygpqf
TD                 2048
SOLVENT           CDCl3
NS                    2
DS                    8
SWH            3591.954 Hz
FIDRES         1.753884 Hz
AQ            0.2852708
sec RG              128
DW              139.200 usec
DE                 6.00 usec
TE                299.2 K
D0           0.00000300 sec
D1           1.00000000 sec
D13          0.00000400 sec
D16          0.00015000 sec
IN0          0.00027840 sec

======== CHANNEL f1 ========
NUC1                 1H
P0                 8.60 usec
P1                 8.60 usec
PL1                2.00 dB
PL1W        15.84893227 W
SFO1        600.1520032 MHz

====== GRADIENT CHANNEL =====
GPNAM1         SINE.100
GPZ1              10.00 %
P16             1000.00
usec ND0                  1
TD                  256
SFO1            600.152
MHz FIDRES    14.031132
Hz SW             5.985
ppm FnMODE           QF
SI                 2048
SF          600.1500165 MHz
WDW                SINE
SSB                   0
LB                 0.00 Hz
GB                    0
PC                 4.00
SI                 2048
MC2                  QF
SF          600.1500165 MHz
WDW                SINE
SSB                   0
LB                 0.00 Hz
GB                    0
```

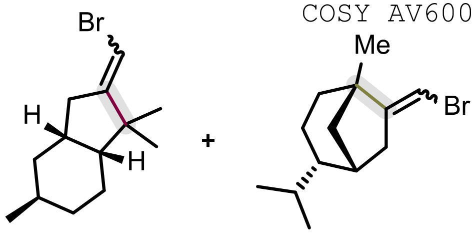

5b **6b**

COSY NMR([600, 600] MHz, $CDCl_3$)

COSY AV600

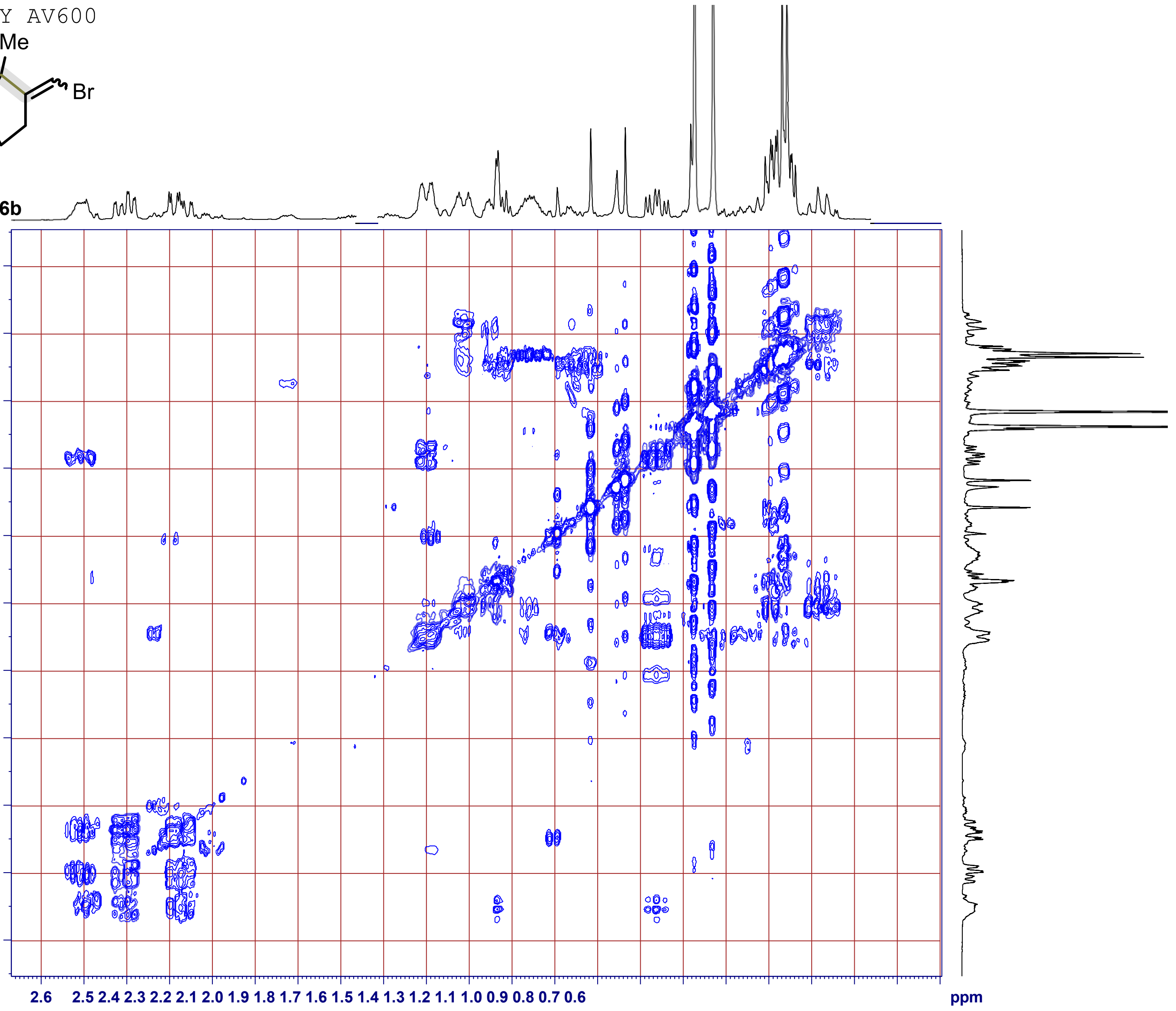


```
NAME            OAS-702F
EXPNO                  7
PROCNO                 1
Date_           20241204
Time               11.17
INSTRUM            spect
PROBHD   5 mm PATXI 1H/
PULPROG         cosygpqf
TD                  2048
SOLVENT            CDCl3
NS                     2
DS                     8
SWH            3591.954 Hz
FIDRES         1.753884 Hz
AQ            0.2852708
sec RG                   128
DW              139.200 usec
DE                 6.00 usec
TE                299.2 K
D0           0.00000300 sec
D1           1.00000000 sec
D13          0.00000400 sec
D16          0.00015000 sec
IN0          0.00027840 sec

======== CHANNEL f1 ========
NUC1                  1H
P0                  8.60 usec
P1                  8.60 usec
PL1                 2.00 dB
PL1W         15.84893227 W
SFO1         600.1520032 MHz

====== GRADIENT CHANNEL =====
GPNAM1          SINE.100
GPZ1               10.00 %
P16              1000.00
usec ND0                   1
TD                   256
SFO1             600.152
MHz FIDRES         14.031132
Hz SW                5.985
ppm FnMODE                QF
SI                  2048
SF           600.1500165 MHz
WDW                 SINE
SSB                    0
LB                  0.00 Hz
GB                     0
PC                  4.00
SI                  2048
MC2                   QF
SF           600.1500165 MHz
WDW                 SINE
SSB                    0
LB                  0.00 Hz
GB                     0
```

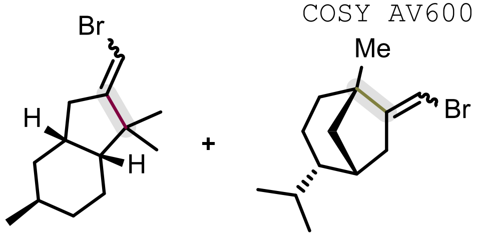

5b + **6b**

COSY NMR([600, 600] MHz, $CDCl_3$)

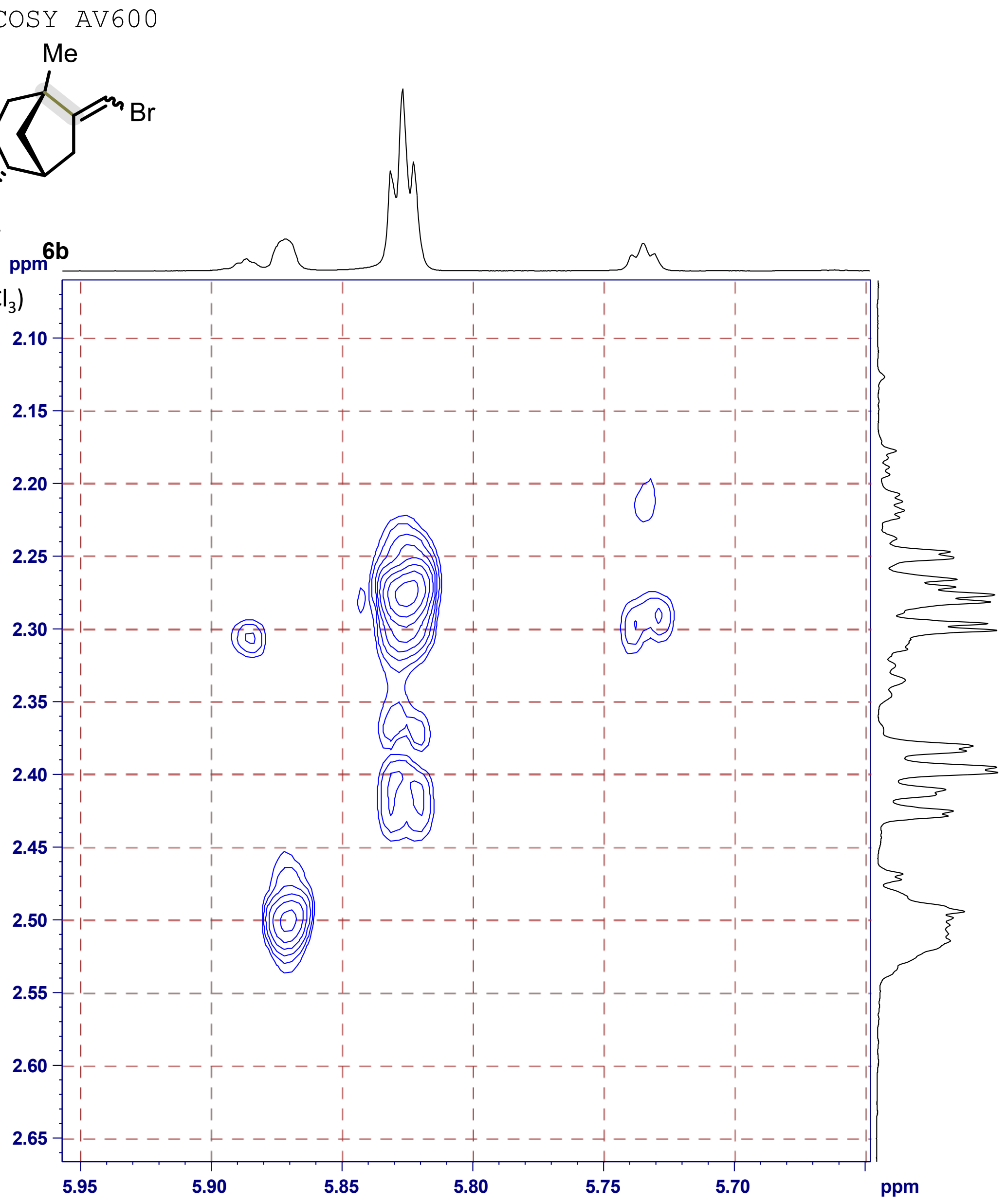


```
NAME          OAS-702F
EXPNO                7
PROCNO               1
Date_         20241204
Time             11.17
INSTRUM          spect
PROBHD   5 mm PATXI 1H/
PULPROG       cosygpqf
TD                2048
SOLVENT          CDC13
NS                   2
DS                   8
SWH           3591.954 Hz
FIDRES        1.753884 Hz
AQ           0.2852708
sec RG                 128
DW             139.200 usec
DE                6.00 usec
TE               299.2 K
D0          0.00000300 sec
D1          1.00000000 sec
D13         0.00000400 sec
D16         0.00015000 sec
IN0         0.00027840 sec

======== CHANNEL f1 ========
NUC1                1H
P0                8.60 usec
P1                8.60 usec
PL1               2.00 dB
PL1W       15.84893227 W
SFO1       600.1520032 MHz

====== GRADIENT CHANNEL =====
GPNAM1        SINE.100
GPZ1             10.00 %
P16            1000.00
usec ND0                 1
TD                 256
SFO1           600.152
MHz FIDRES   14.031132
Hz SW            5.985
ppm FnMODE          QF
SI                2048
SF         600.1500165 MHz
WDW               SINE
SSB                  0
LB                0.00 Hz
GB                   0
PC                4.00
SI                2048
MC2                 QF
SF         600.1500165 MHz
WDW               SINE
SSB                  0
LB                0.00 Hz
GB                   0
```

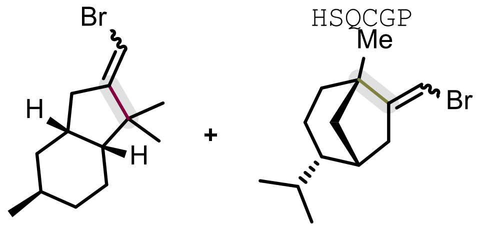

5b + **6b**

HSQC NMR([600, 150] MHz, $CDCl_3$)

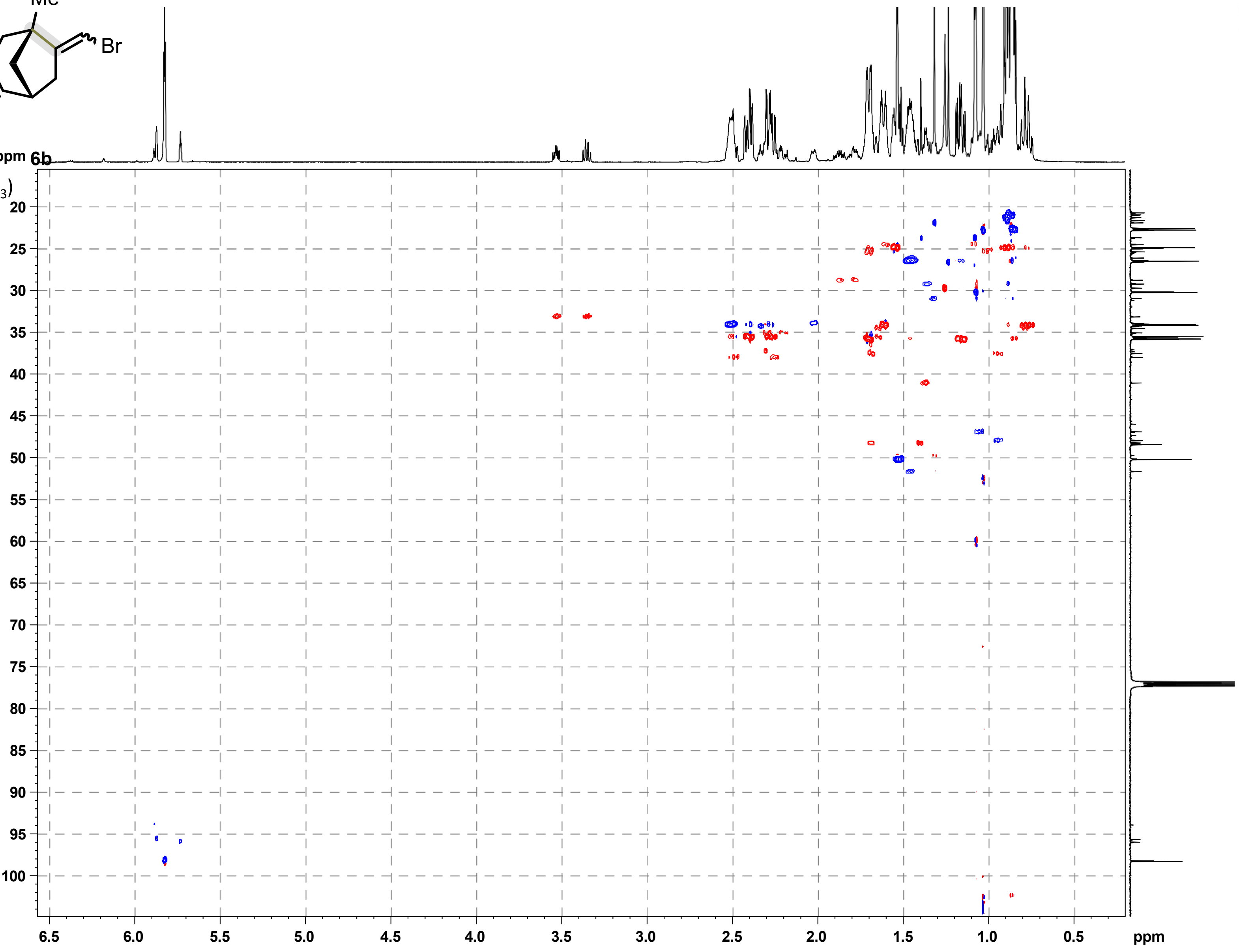

NAME OAS-702F
EXPNO 4
PROCNO 2
Date_ 20241204
Time 0.49
INSTRUM spect
PROBHD 5 mm PATXI 1H/
PULPROG hsqcedetgpsisp2
TD 2048
SOLVENT CDCl3
NS 16
DS 8
SWH 3822.630 Hz
FIDRES 1.866518 Hz
AQ 0.2680592 sec
RG 14600
DW 130.800 usec
DE 6.00 usec
TE 300.2 K
CNST2 145.0000000
D0 0.00000300 sec
D1 1.00000000 sec
D4 0.00172414 sec
D11 0.03000000 sec
D16 0.00015000 sec
D21 0.00350000 sec
D24 0.00086200 sec
IN0 0.00003010 sec
ZGOPTNS
======== CHANNEL f1 ========
NUC1 1H
P1 8.60 usec
P2 17.20 usec
P28 250.00 usec
PL1 2.00 dB
PL1W 15.84893227 W
SFO1 600.1520480 MHz
======== CHANNEL f2 ========
CPDPRG2 garp
NUC2 13C
P3 12.50 usec
P4 25.00 usec
P14 500.00 usec
PCPD2 77.00 usec
PL0 120.00 dB
PL2 -3.00 dB
PL12 12.75 dB
PL0W 0.00000000 W
PL2W 150.35617065 W
PL12W 4.00056410 W
SFO2 150.9153834 MHz
SP3 3.22 dB
SPNAM3 Crp60,0.5,20.1
SPOAL3 0.500
SPOFFS3 0.00 Hz
GPNAM2 GRADIENT CHANNEL =====
GPNAM1 SINE.100
GPNAM4 SINE.10
GPZ1 0
GPZ2 80.00
GPZ3 %
GPZ4 20.10
P16 %
P19 11.00 %
ND0 -5.00 %
TD 1000.00
SFO1 usec
FIDRES 600.00 usec
SW 2
FnMODE 256
SI 150.9154 MHz
SF 64.846451 Hz
WDW 110.000 ppm
SSB Echo-Antiecho
LB 1024
GB 600.1500165 MHz
PC QSINE
SI 2
MC2 0.00 Hz
SF 0
WDW 4.0
SSB 0
LB 832
GB echo-antiecho
150.9078380 MHz
QSIN
E
2
0.00
Hz
0

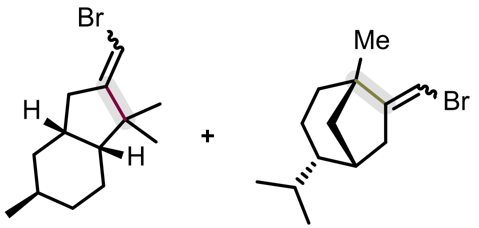

5b + **6b**

HSQC NMR([600, 150] MHz, $CDCl_3$)

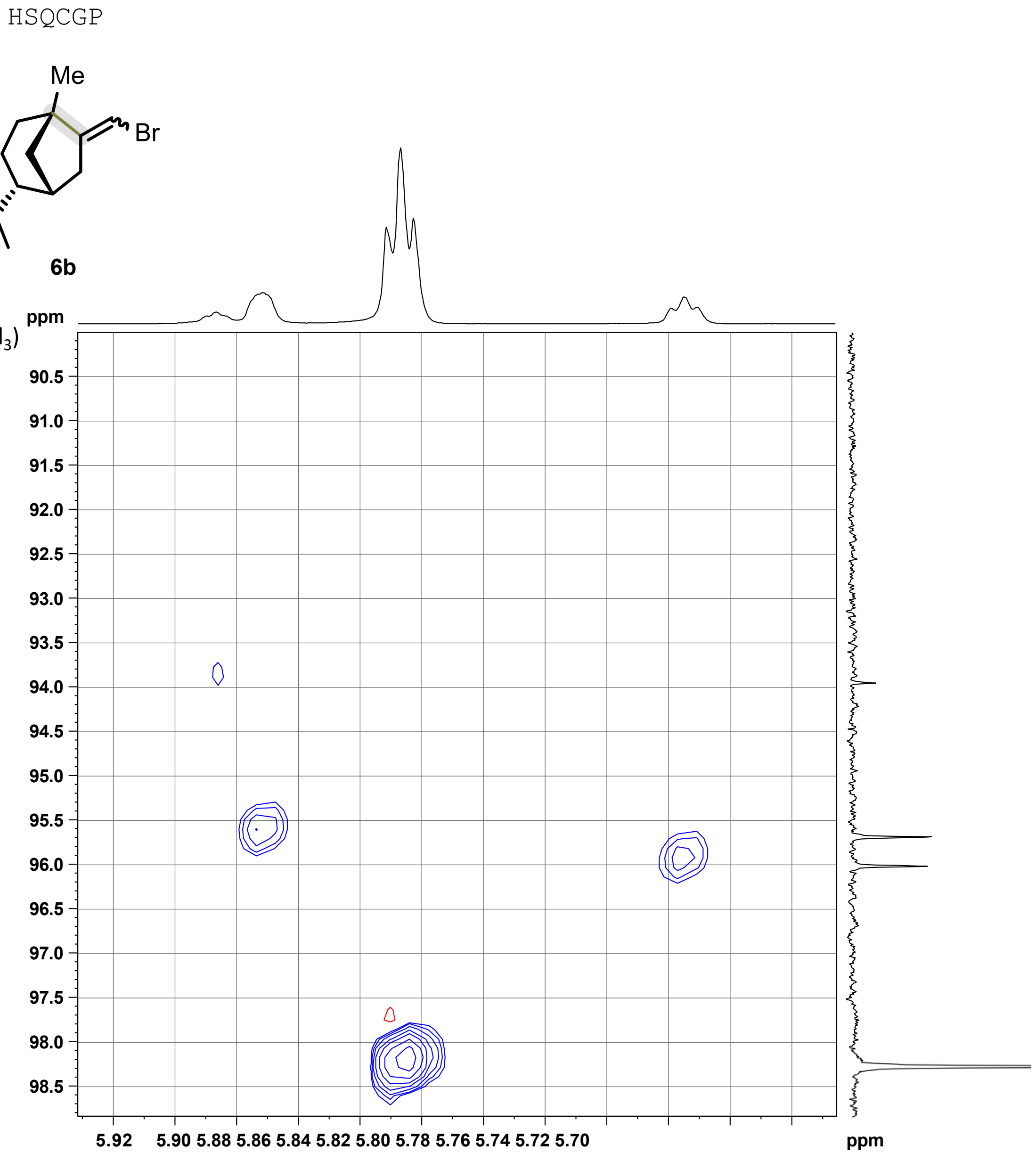


```
NAME              OAS-702F
EXPNO
4
PROCNO                   3
Date_             20241204
Time                  0.49
INSTRUM              spect
PROBHD   5 mm PATXI 1H/
PULPROG
hsqcedetgpsisp2 TD
2048
SOLVENT              CDCl3
NS                      16
DS                       8
SWH               3822.630 Hz
FIDRES            1.866518 Hz
AQ               0.2680592 sec
RG                   14600
DW                 130.800 usec
DE                    6.00 usec
TE                   300.2 K
CNST2         145.0000000
D0              0.00000300 sec
D1              1.00000000 sec
D4              0.00172414 sec
D11             0.03000000 sec
D16             0.00015000 sec
D21             0.00350000 sec
D24             0.00086200 sec
IN0             0.00003010 sec

ZGOPTNS

======== CHANNEL f1 ========
NUC1                    1H
P1                    8.60 usec
P2                   17.20 usec
P28                 250.00 usec
PL1                   2.00 dB
PL1W           15.84893227 W
SFO1           600.1520480 MHz

======== CHANNEL f2 ========
CPDPRG2               garp
NUC2                   13C
P3                   12.50 usec
P4                   25.00 usec
P14                 500.00 usec
PCPD2                77.00 usec
PL0                 120.00 dB
PL2                  -3.00 dB
PL12                 12.75 dB
PL0W            0.00000000 W
PL2W          150.35617065 W
PL12W           4.00056410 W
SFO2           150.9153834 MHz
SP3                   3.22 dB
SPNAM3   Crp60,0.5,20.1
SPOAL3               0.500
SPOFFS3               0.00 Hz

====== GRADIENT CHANNEL =====
GPNAM1            SINE.100
GPNAM2            SINE.100
GPNAM3            SINE.100
GPNAM4            SINE.100
GPZ1                 80.00 %
GPZ2                 20.10 %
GPZ3                 11.00 %
GPZ4                 -5.00 %
P16                1000.00
usec P19
600.00 usec ND0
2
TD                     256
SFO1              150.9154
MHz FIDRES
64.846451 Hz SW
110.000 ppm FnMODE
Echo-Antiecho
SI                    1024
SF             600.1500144
MHz WDW                  QSINE
SSB                      2
LB                    0.00 Hz
GB                       0
PC                    4.00
SI                     832
MC2          echo-antiecho
SF             150.9078380
MHz WDW                  QSINE
SSB                      2
LB                    0.00 Hz
GB                       0
```

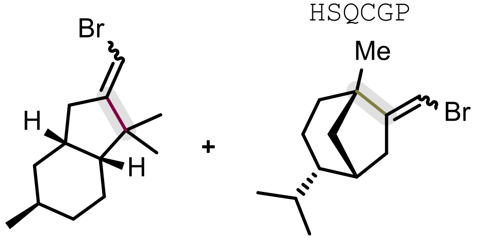

5b + 6b

HSQC NMR([600, 150] MHz, $CDCl_3$)

HSQCGP

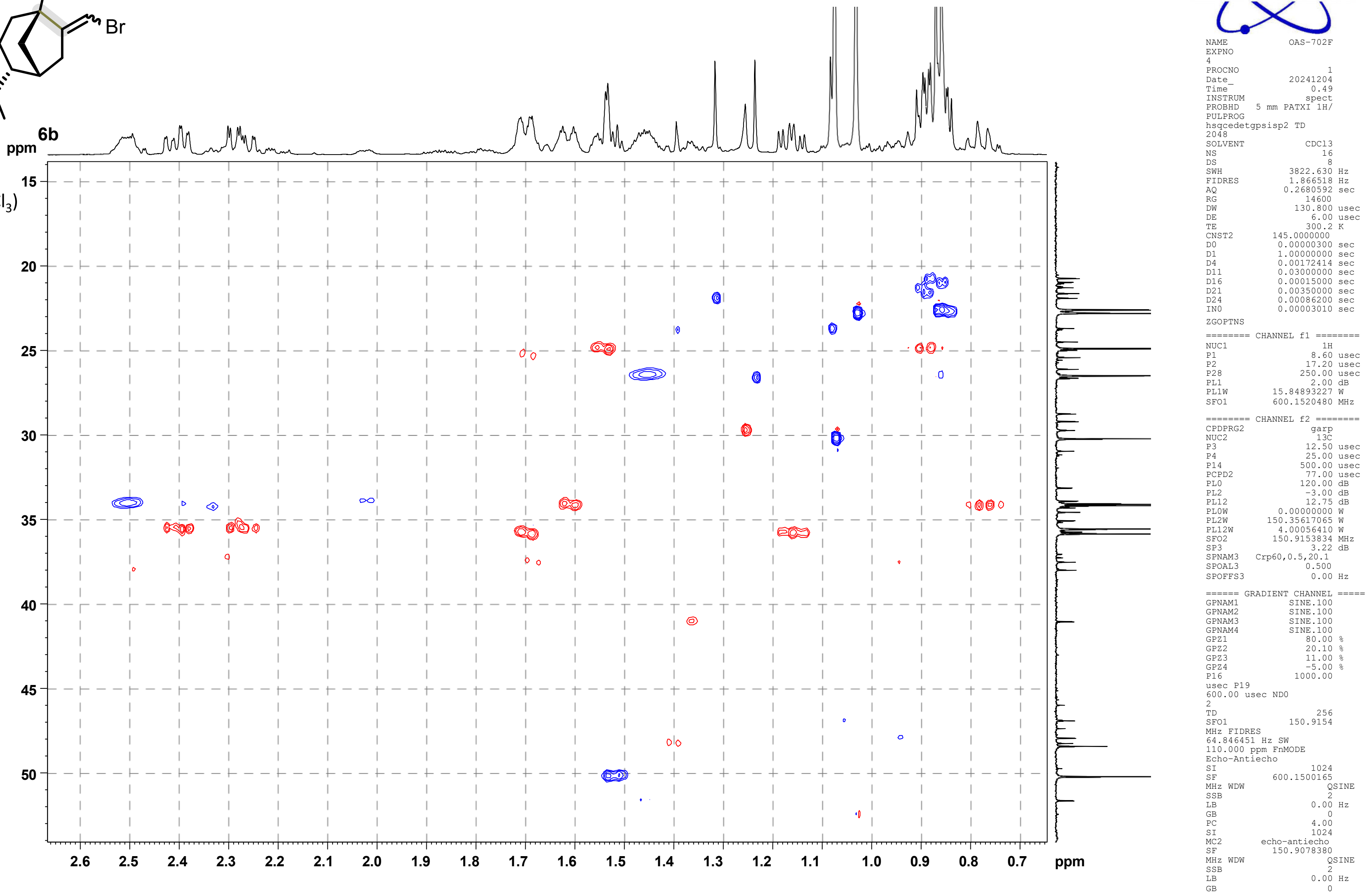

HSQCGP

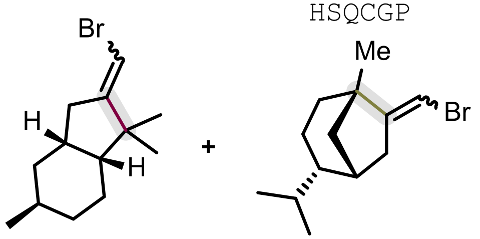

5b **6b**

HSQC NMR([600, 150] MHz, $CDCl_3$)

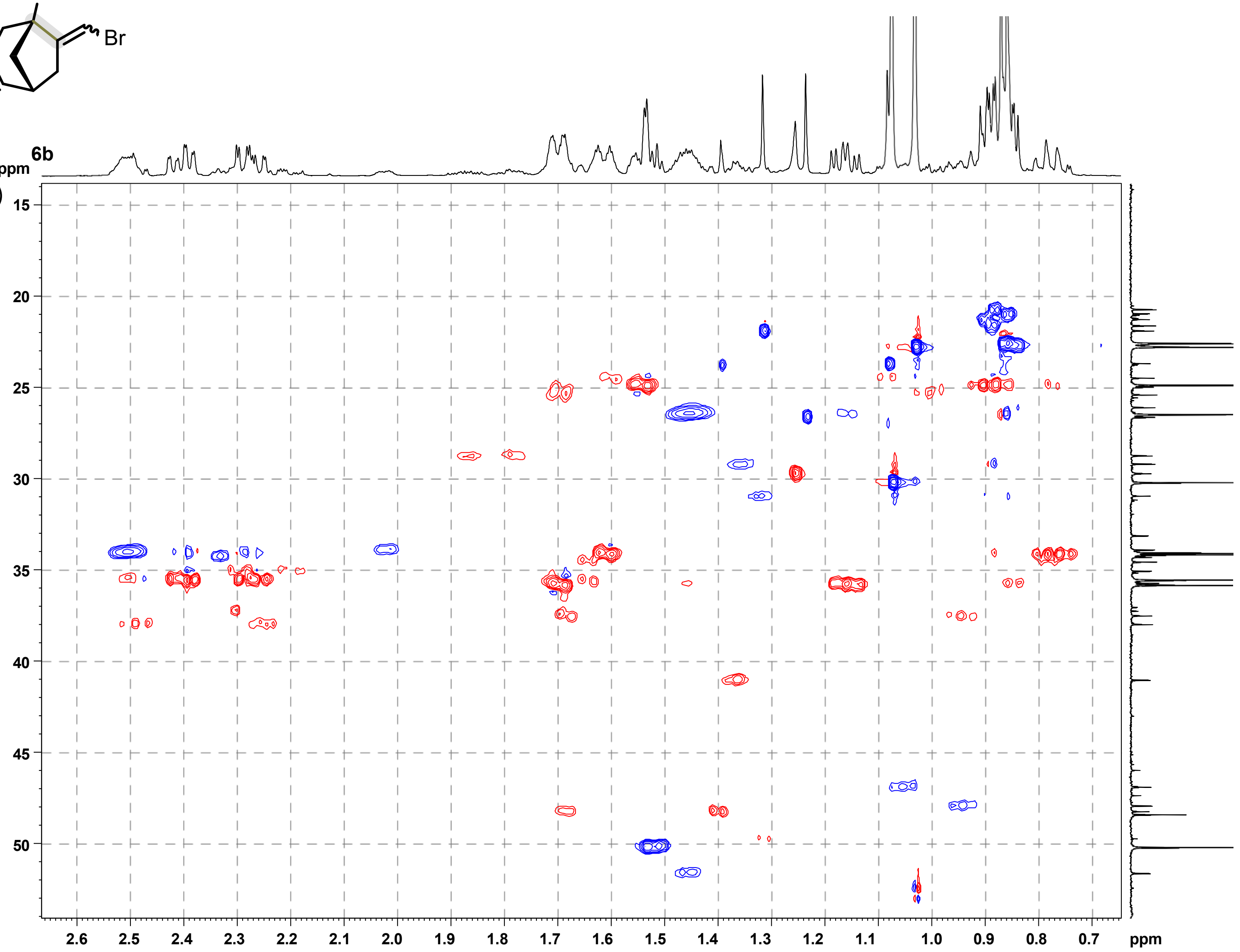


```
NAME              OAS-702F
EXPNO
4
PROCNO                   1
Date_             20241204
Time                  0.49
INSTRUM              spect
PROBHD   5 mm PATXI 1H/
PULPROG
hsqcedetgpsisp2 TD
2048
SOLVENT              CDCl3
NS                      16
DS                       8
SWH             3822.630 Hz
FIDRES          1.866518 Hz
AQ             0.2680592 sec
RG                   14600
DW               130.800 usec
DE                  6.00 usec
TE                 300.2 K
CNST2        145.0000000
D0            0.00000300 sec
D1            1.00000000 sec
D4            0.00172414 sec
D11           0.03000000 sec
D16           0.00015000 sec
D21           0.00350000 sec
D24           0.00086200 sec
IN0           0.00003010 sec
ZGOPTNS

======== CHANNEL f1 ========
NUC1                    1H
P1                    8.60 usec
P2                   17.20 usec
P28                 250.00 usec
PL1                   2.00 dB
PL1W           15.84893227 W
SFO1           600.1520480 MHz

======== CHANNEL f2 ========
CPDPRG2               garp
NUC2                   13C
P3                   12.50 usec
P4                   25.00 usec
P14                 500.00 usec
PCPD2                77.00 usec
PL0                 120.00 dB
PL2                  -3.00 dB
PL12                 12.75 dB
PL0W            0.00000000 W
PL2W          150.35617065 W
PL12W           4.00056410 W
SFO2           150.9153834 MHz
SP3                   3.22 dB
SPNAM3    Crp60,0.5,20.1
SPOAL3               0.500
SPOFFS3               0.00 Hz

====== GRADIENT CHANNEL =====
GPNAM1            SINE.100
GPNAM2            SINE.100
GPNAM3            SINE.100
GPNAM4            SINE.100
GPZ1                 80.00 %
GPZ2                 20.10 %
GPZ3                 11.00 %
GPZ4                 -5.00 %
P16                1000.00
usec P19
600.00 usec ND0
2
TD                     256
SFO1              150.9154
MHz FIDRES
64.846451 Hz SW
110.000 ppm FnMODE
Echo-Antiecho
SI                    1024
SF             600.1500165
MHz WDW              QSINE
SSB                      2
LB                    0.00 Hz
GB                       0
PC                    4.00
SI                    1024
MC2           echo-antiecho
SF             150.9078380
MHz WDW              QSINE
SSB                      2
LB                    0.00 Hz
GB                       0
```

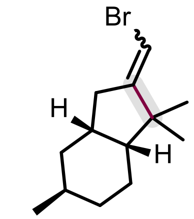

5b

+

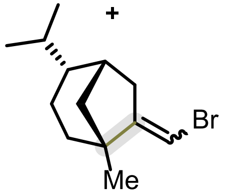

6b

HMBC NMR([600, 150] MHz, $CDCl_3$)

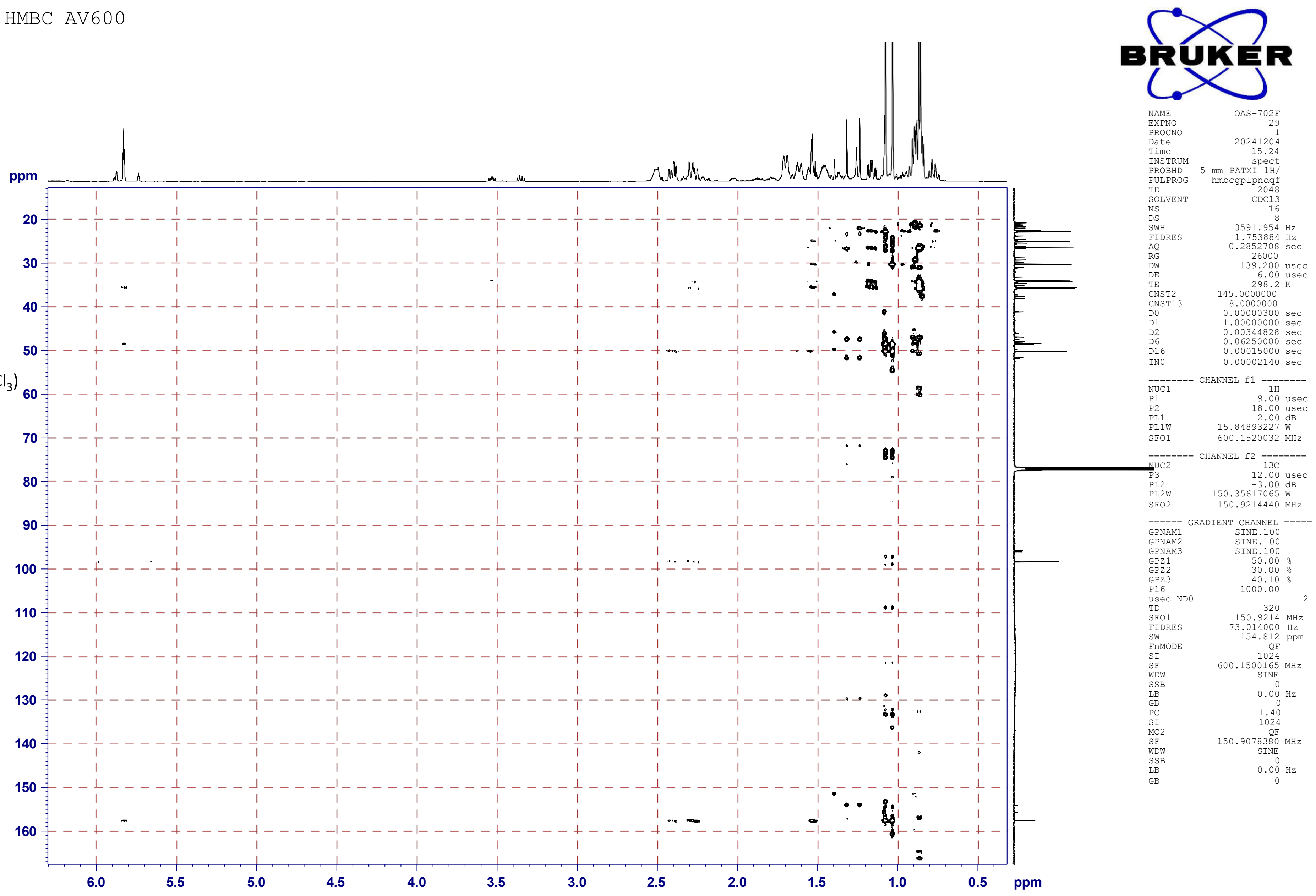

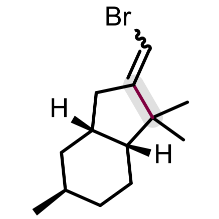

5b

+

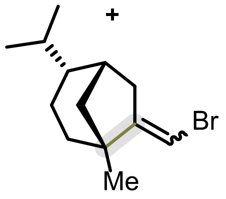

6b

HMBC NMR([600, 150] MHz, $CDCl_3$)

HMBC AV600

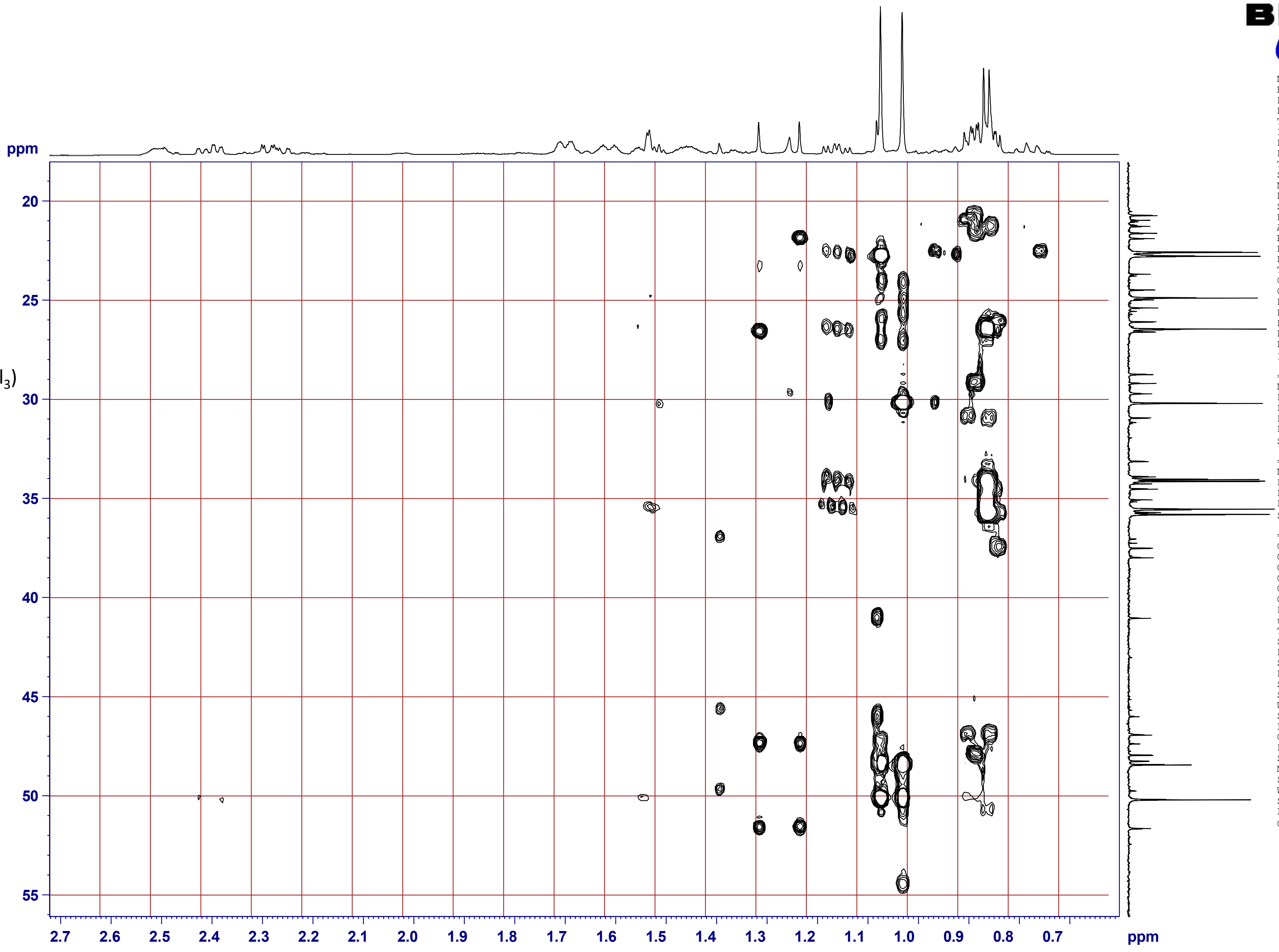

NAME	OAS-702F
EXPNO	29
PROCNO	1
Date_	20241204
Time	15.24
INSTRUM	spect
PROBHD	5 mm PATXI 1H/
PULPROG	hmbcgplpndqf
TD	2048
SOLVENT	CDCl3
NS	16
DS	8
SWH	3591.954 Hz
FIDRES	1.753884 Hz
AQ	0.2852708 sec
RG	26000
DW	139.200 usec
DE	6.00 usec
TE	298.2 K
CNST2	145.0000000
CNST13	8.0000000
D0	0.00000300 sec
D1	1.00000000 sec
D2	0.00344828 sec
D6	0.06250000 sec
D16	0.00015000 sec
IN0	0.00002140 sec
======== CHANNEL f1 ========	
NUC1	1H
P1	9.00 usec
P2	18.00 usec
PL1	2.00 dB
PL1W	15.84893227 W
SFO1	600.1520032 MHz
======== CHANNEL f2 ========	
NUC2	13C
P3	12.00 usec
PL2	-3.00 dB
PL2W	150.35617065 W
SFO2	150.9214440 MHz
====== GRADIENT CHANNEL =====	
GPNAM1	SINE.100
GPNAM2	SINE.100
GPNAM3	SINE.100
GPZ1	50.00 %
GPZ2	30.00 %
GPZ3	40.10 %
P16	1000.00
usec ND0	2
TD	320
SFO1	150.9214 MHz
FIDRES	73.014000 Hz
SW	154.812 ppm
FnMODE	QF
SI	1024
SF	600.1500165 MHz
WDW	SINE
SSB	0
LB	0.00 Hz
GB	0
PC	1.40
SI	1024
MC2	QF
SF	150.9078380 MHz
WDW	SINE
SSB	0
LB	0.00 Hz
GB	0

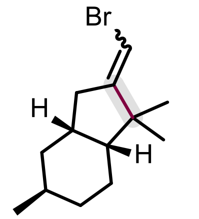

5b

+

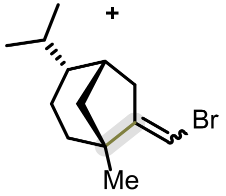

6b

HMBC NMR([600, 150] MHz, $CDCl_3$)

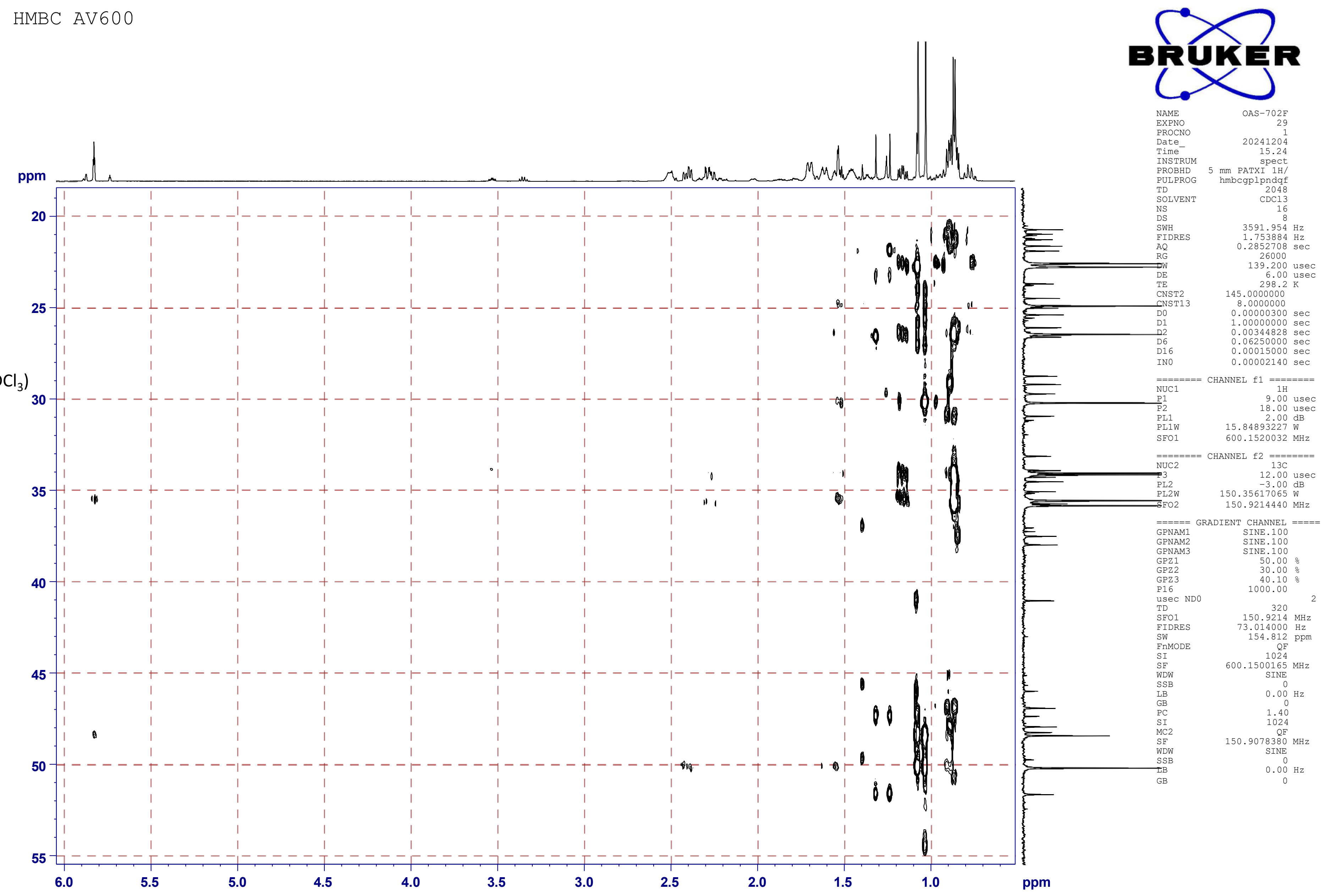

HMBC AV600

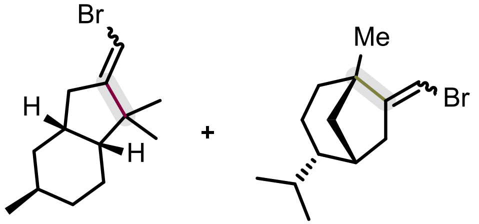

5b **6b**

HMBC NMR([600, 150] MHz, $CDCl_3$)

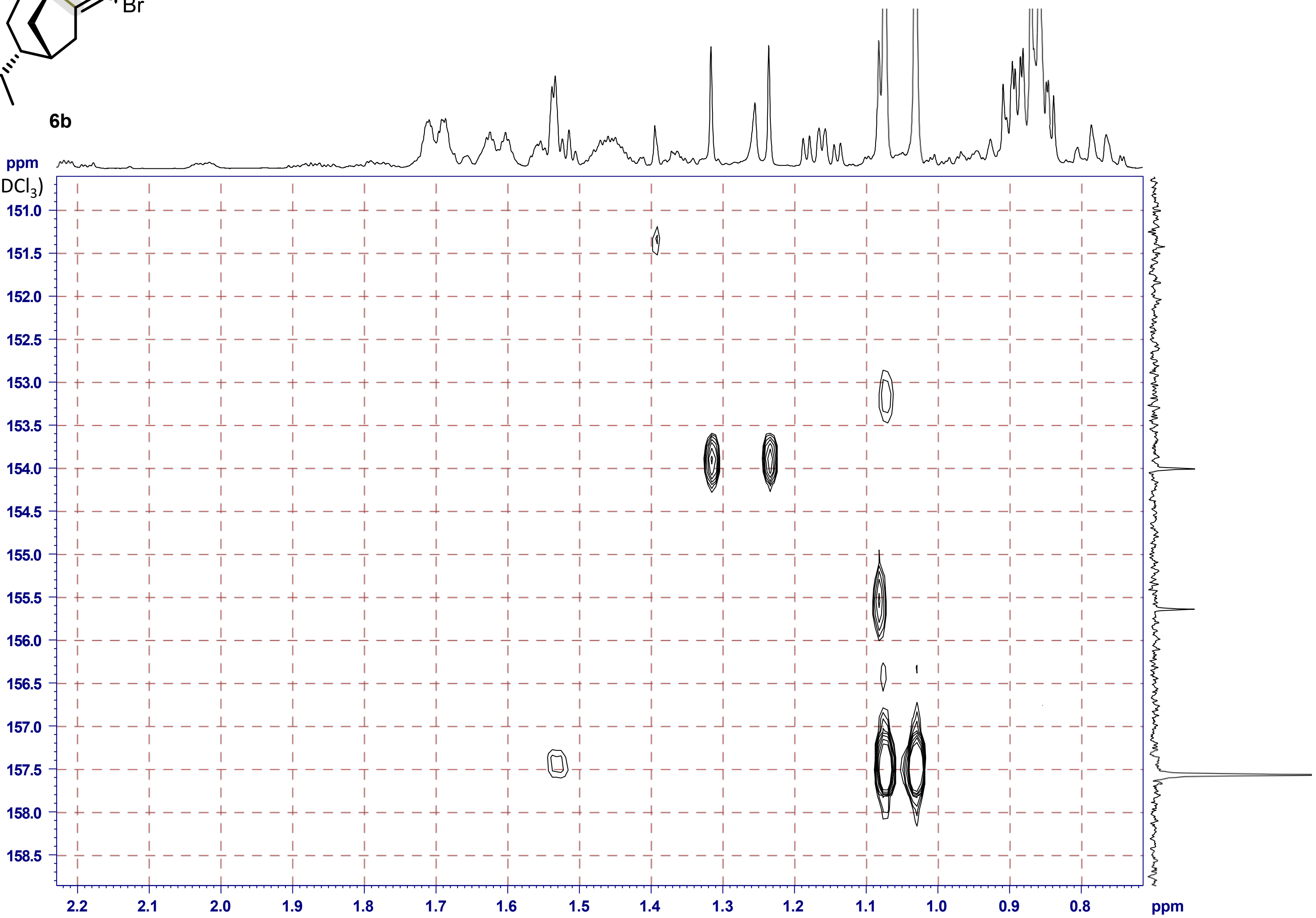

NAME OAS-702F
EXPNO 29
PROCNO 1
Date_ 2024120
Time 4
INSTRUM 15.24
PROBHD spect
PULPROG 5 mm PATXI 1H/
TD hmbcgplpndq
SOLVENT f
NS 2048
DS CDCl
SWH 3
FIDRES 16
AQ 8
RG 3591.954 Hz
DW 1.753884 Hz
DE 0.2852708
TE sec
CNST2 26000
CNST13 139.200 usec
D0 6.00 usec
D1 298.2 K
D2 145.000000
D6 0
D16 8.0000000
IN0 0.00000300 sec
1.00000000 sec
======== CHANNEL f1 ========
NUC1 0.06250000 sec
P1 0.00015000 sec
P2 0.00002140 usec
PL1 2.00 dB
PL1W 15.84893227 W
SFO1 600.1520032 MHz
======== CHANNEL f2 ========
NUC2 13C
P3 12.00 usec
PL2 -3.00 dB
PL2W 150.35617065 W
SFO2 150.9214440 MHz
====== GRADIENT CHANNEL =====
GPNAM1 SINE.100
GPNAM2 SINE.100
GPNAM3 SINE.10
GPZ1 0
GPZ2 50.00
GPZ3 %
P16 30.00
ND0 %
TD 40.10 %
SFO1 1000.00 usec
FIDRES 2
SW 320
FnMODE 150.9214 MHz
SI 73.014000 Hz
SF 154.812
WDW ppm
SSB QF
LB 1024
GB 600.1500165 MHz
PC SINE
SI 0
MC2 0.00 Hz
SF 0
WDW 1.4
SSB 0
LB 1024
GB QF
150.9078380 MHz
SIN
E
0
0.00
Hz
0

HMBC AV600

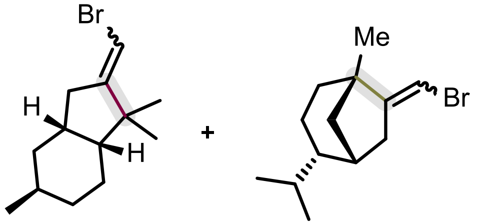

5b + **6b**

HMBC NMR([600, 150] MHz, $CDCl_3$)

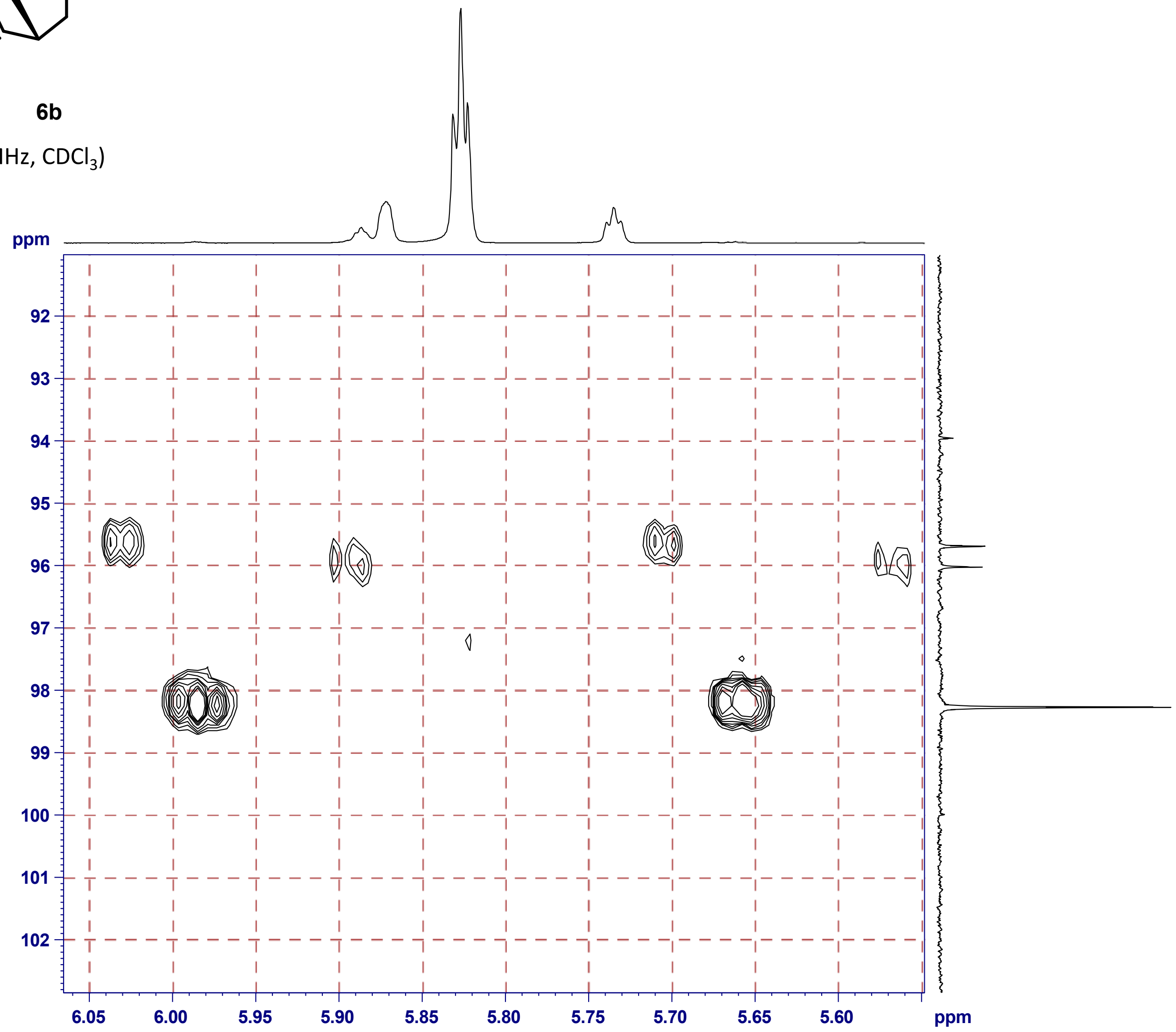


```
NAME              OAS-702F
EXPNO                   29
PROCNO                   1
Date_             20241204
Time                 15.24
INSTRUM              spect
PROBHD   5 mm PATXI 1H/
PULPROG     hmbcgplpndqf
TD                    2048
SOLVENT              CDCl3
NS                      16
DS                       8
SWH               3591.954 Hz
FIDRES            1.753884 Hz
AQ               0.2852708 sec
RG                   26000
DW                 139.200 usec
DE                    6.00 usec
TE                   298.2 K
CNST2          145.0000000
CNST13           8.0000000
D0              0.00000300 sec
D1              1.00000000 sec
D2              0.00344828 sec
D6              0.06250000 sec
D16             0.00015000 sec
IN0             0.00002140 sec

======== CHANNEL f1 ========
NUC1                    1H
P1                    9.00 usec
P2                   18.00 usec
PL1                   2.00 dB
PL1W           15.84893227 W
SFO1           600.1520032 MHz

======== CHANNEL f2 ========
NUC2                   13C
P3                   12.00 usec
PL2                  -3.00 dB
PL2W          150.35617065 W
SFO2           150.9214440 MHz

====== GRADIENT CHANNEL =====
GPNAM1           SINE.100
GPNAM2           SINE.100
GPNAM3           SINE.100
GPZ1                50.00 %
GPZ2                30.00 %
GPZ3                40.10 %
P16               1000.00
usec ND0                     2
TD                        320
SFO1             150.9214 MHz
FIDRES          73.014000 Hz
SW                154.812 ppm
FnMODE                  QF
SI                    1024
SF             600.1500165 MHz
WDW                  SINE
SSB                      0
LB                    0.00 Hz
GB                       0
PC                    1.40
SI                    1024
MC2                     QF
SF             150.9078380 MHz
WDW                  SINE
SSB                      0
LB                    0.00 Hz
GB                       0
```

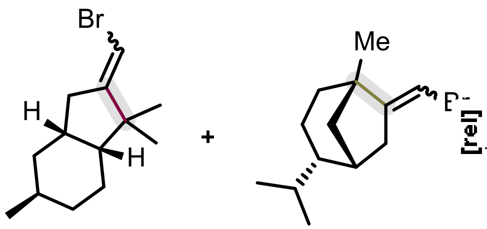

5b + 6b

SEL-NOE NMR(600 MHz, $CDCl_3$)

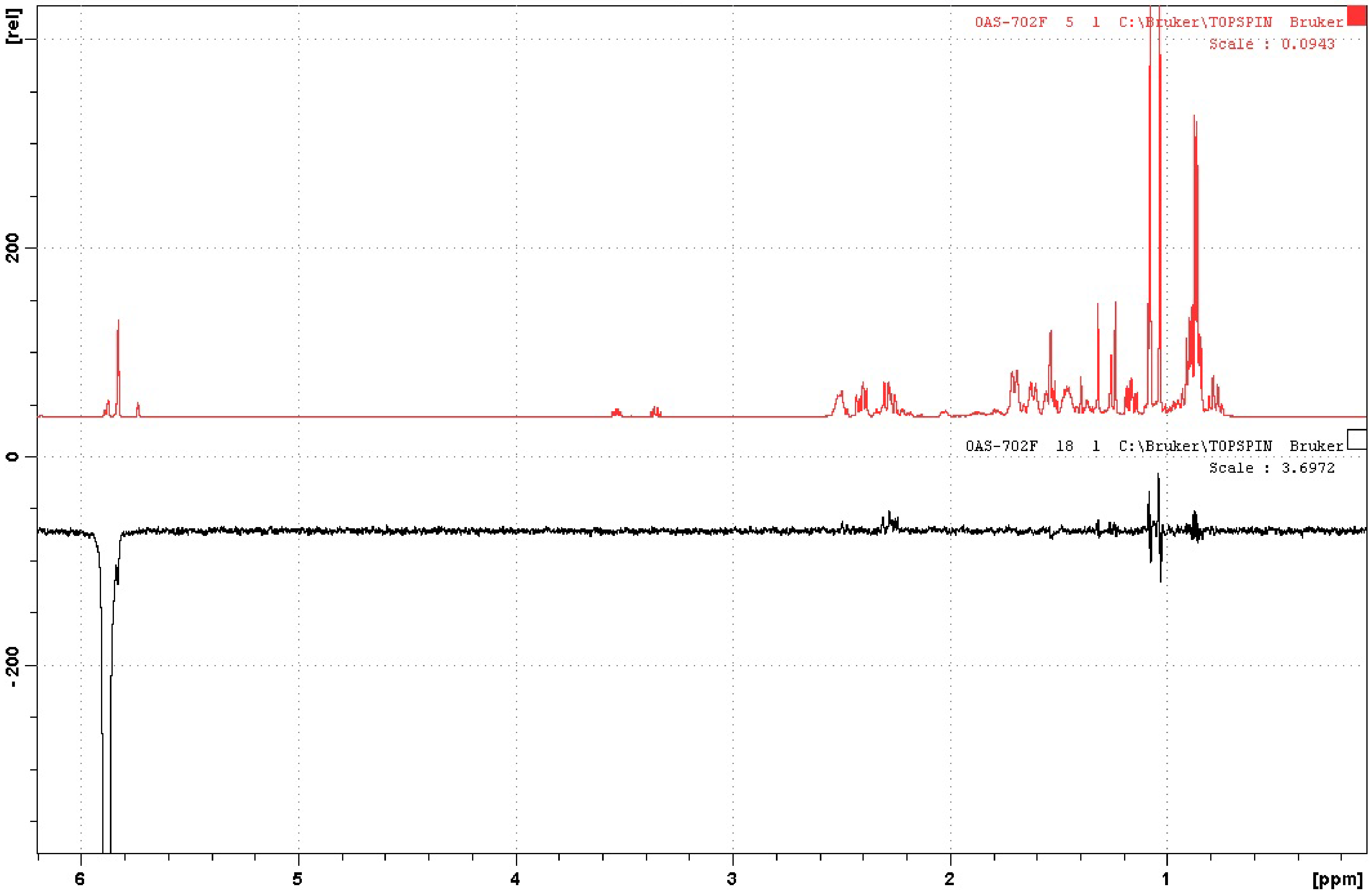

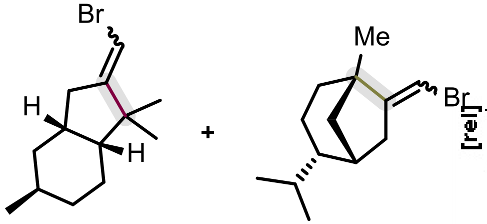

5b **6b**

SEL-NOE NMR(600 MHz, $CDCl_3$)

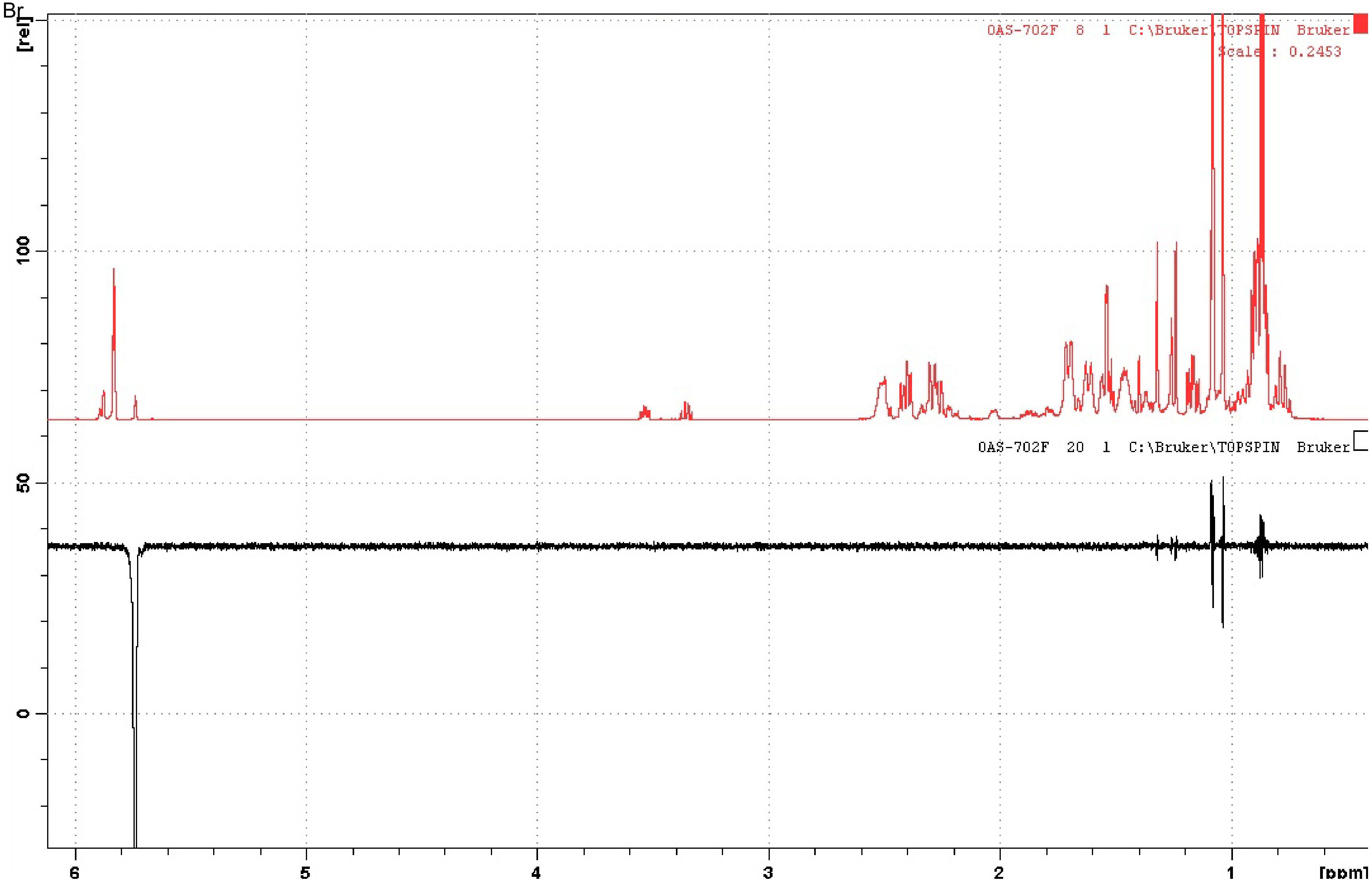

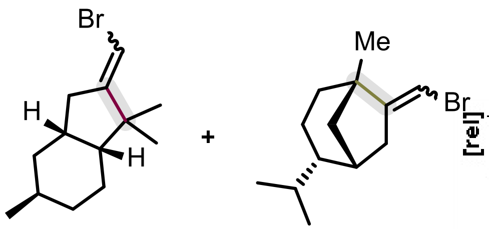

5b **6b**

SEL-NOE NMR(600 MHz, $CDCl_3$)

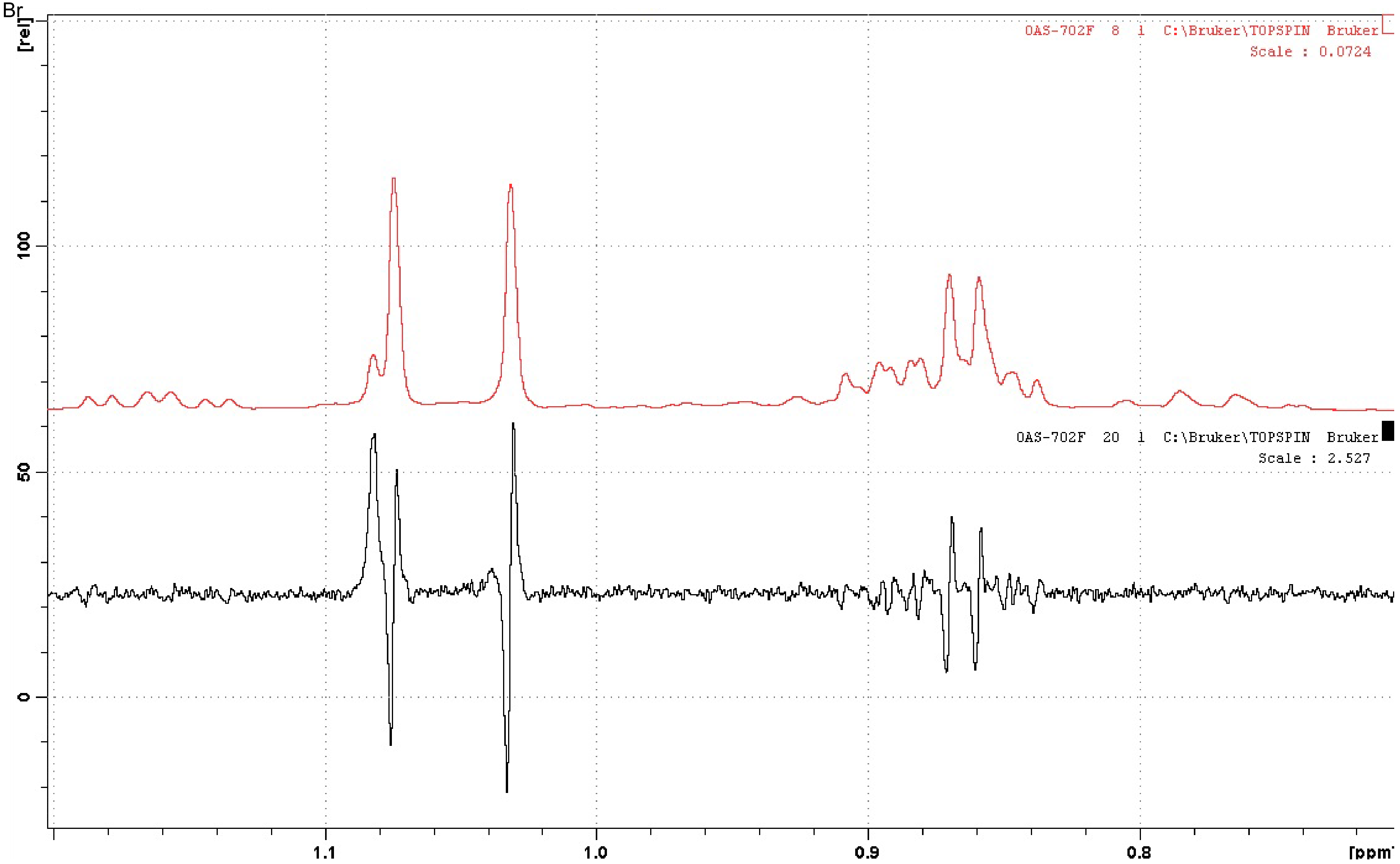

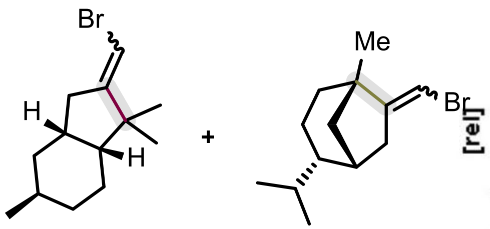

5b **6b**

SEL-NOE NMR(600 MHz, $CDCl_3$)

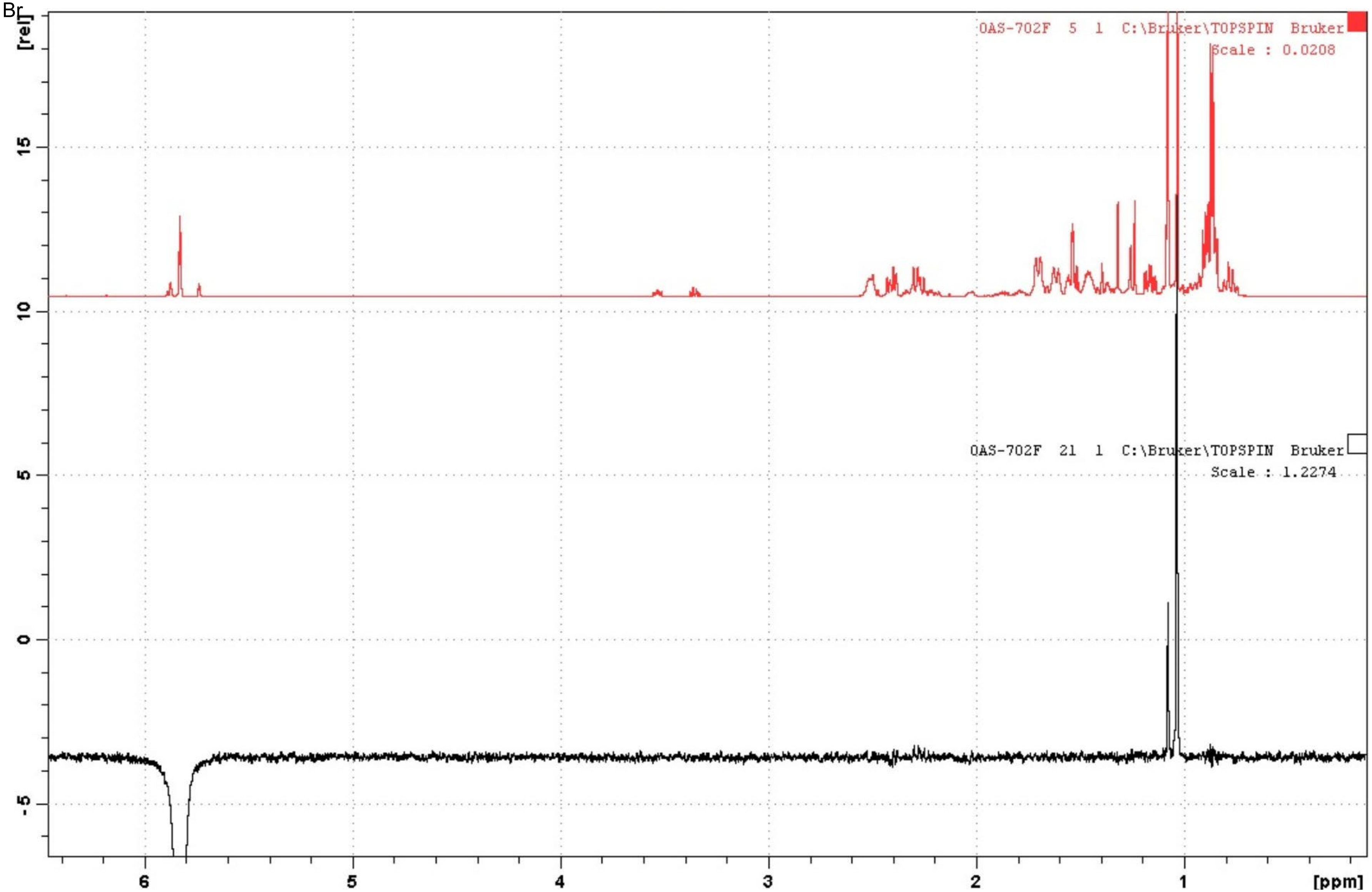

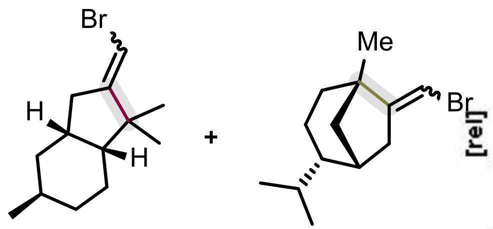

5b + **6b**

SEL-NOE/ SEL-TOCSY NMR(600 MHz, $CDCl_3$)

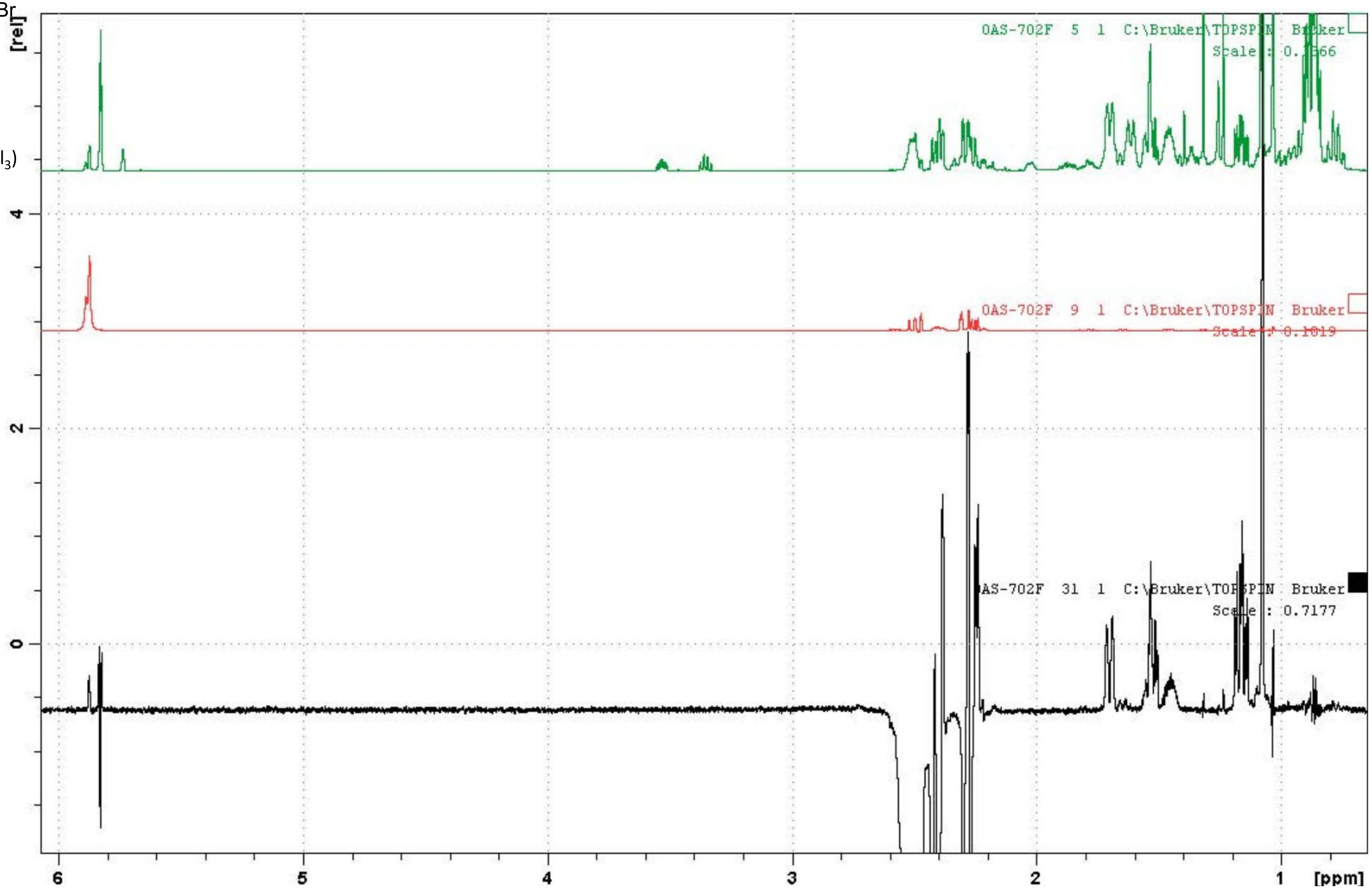

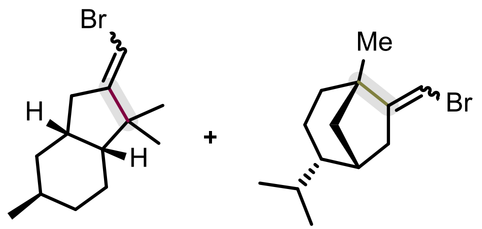

5b **6b**

HSQC/ SEL-TOCSY NMR(600 MHz, $CDCl_3$)

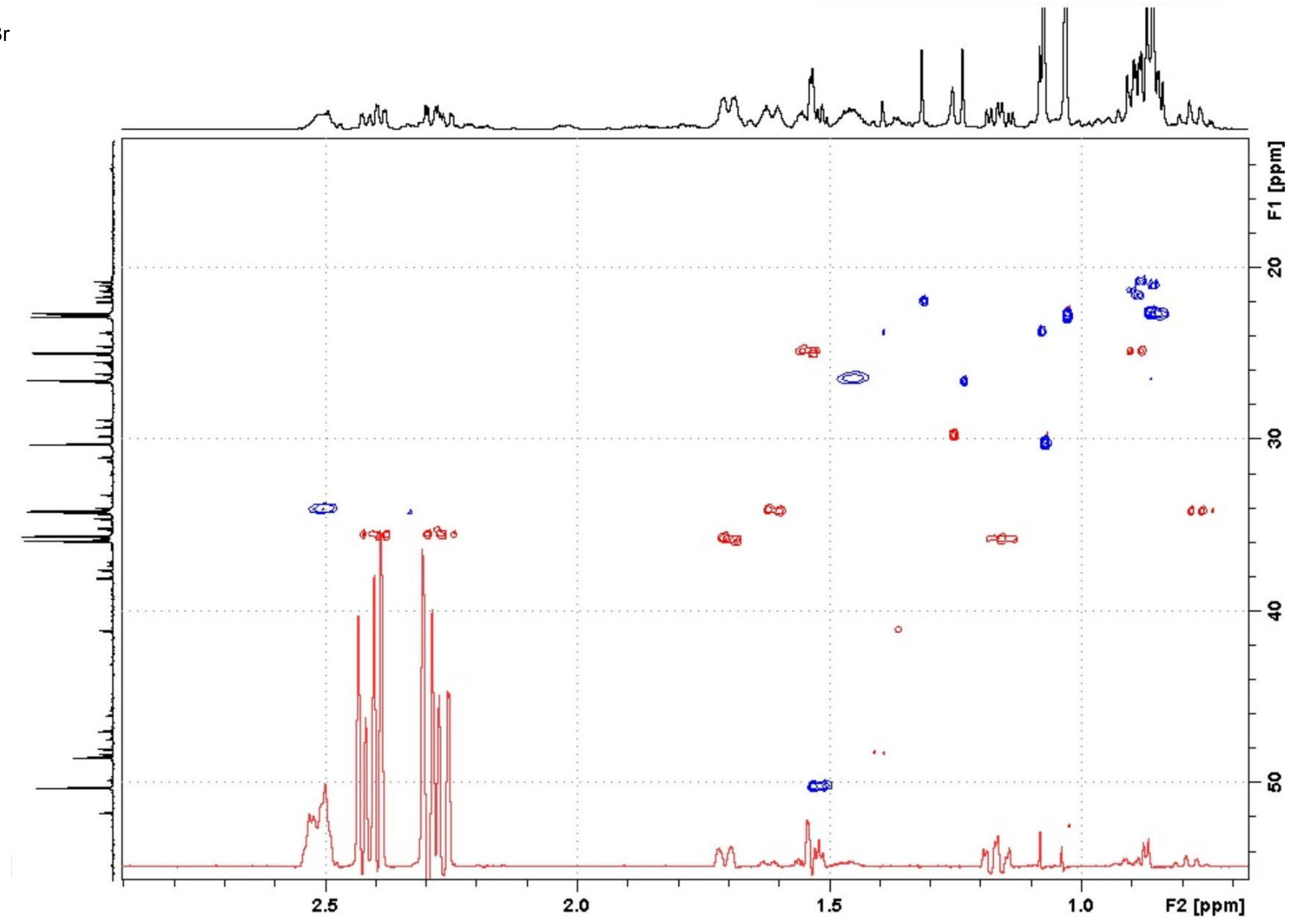

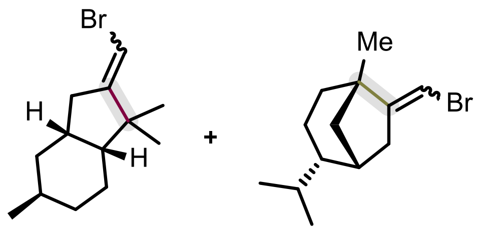

5b **6b**

HSQC/ SEL-TOCSY NMR(600 MHz, $CDCl_3$)

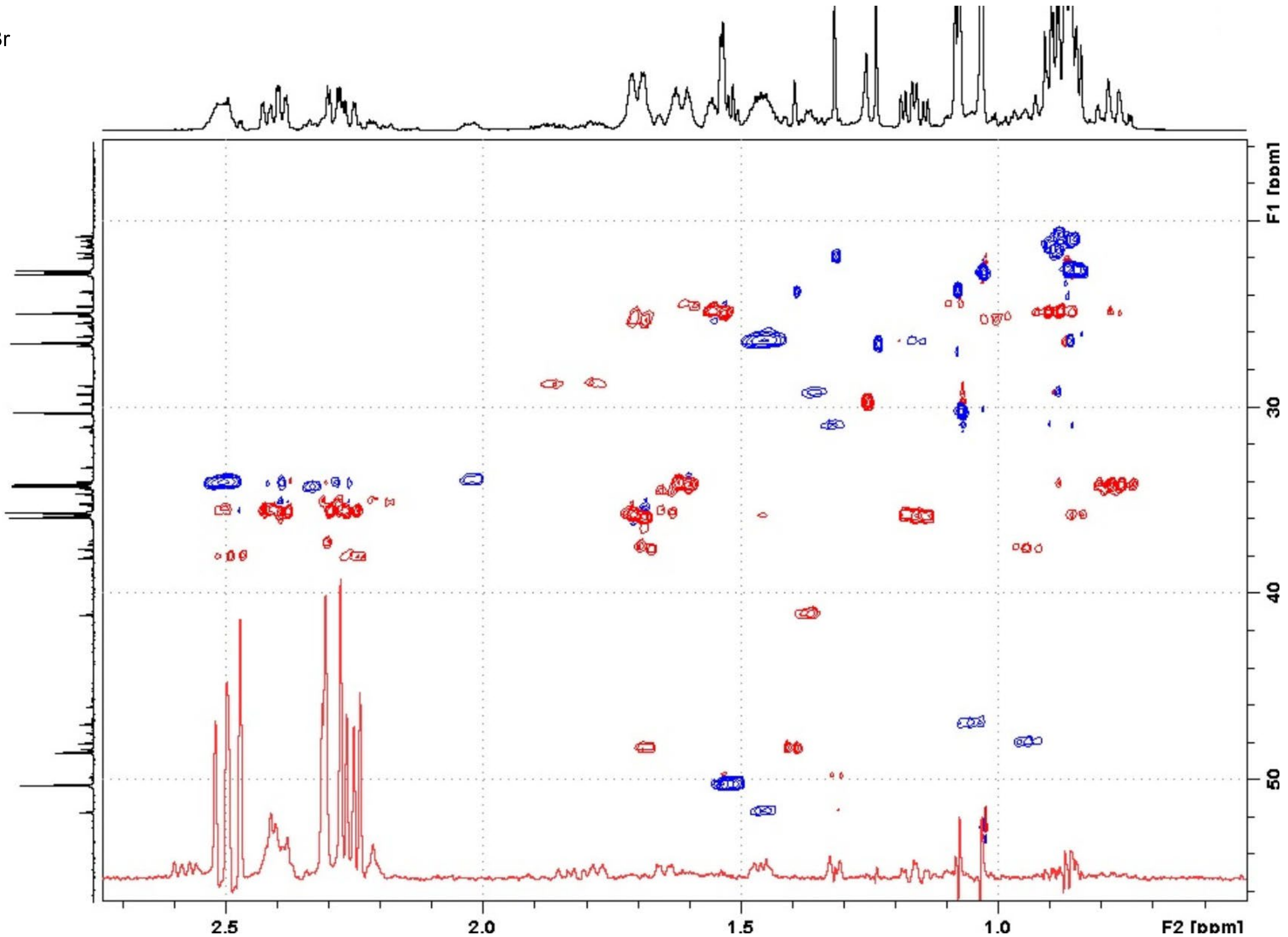

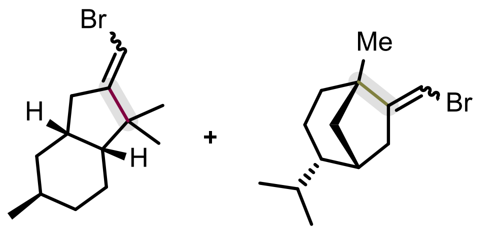

5b **6b**

HSQC/ SEL-TOCSY NMR(600 MHz, $CDCl_3$)

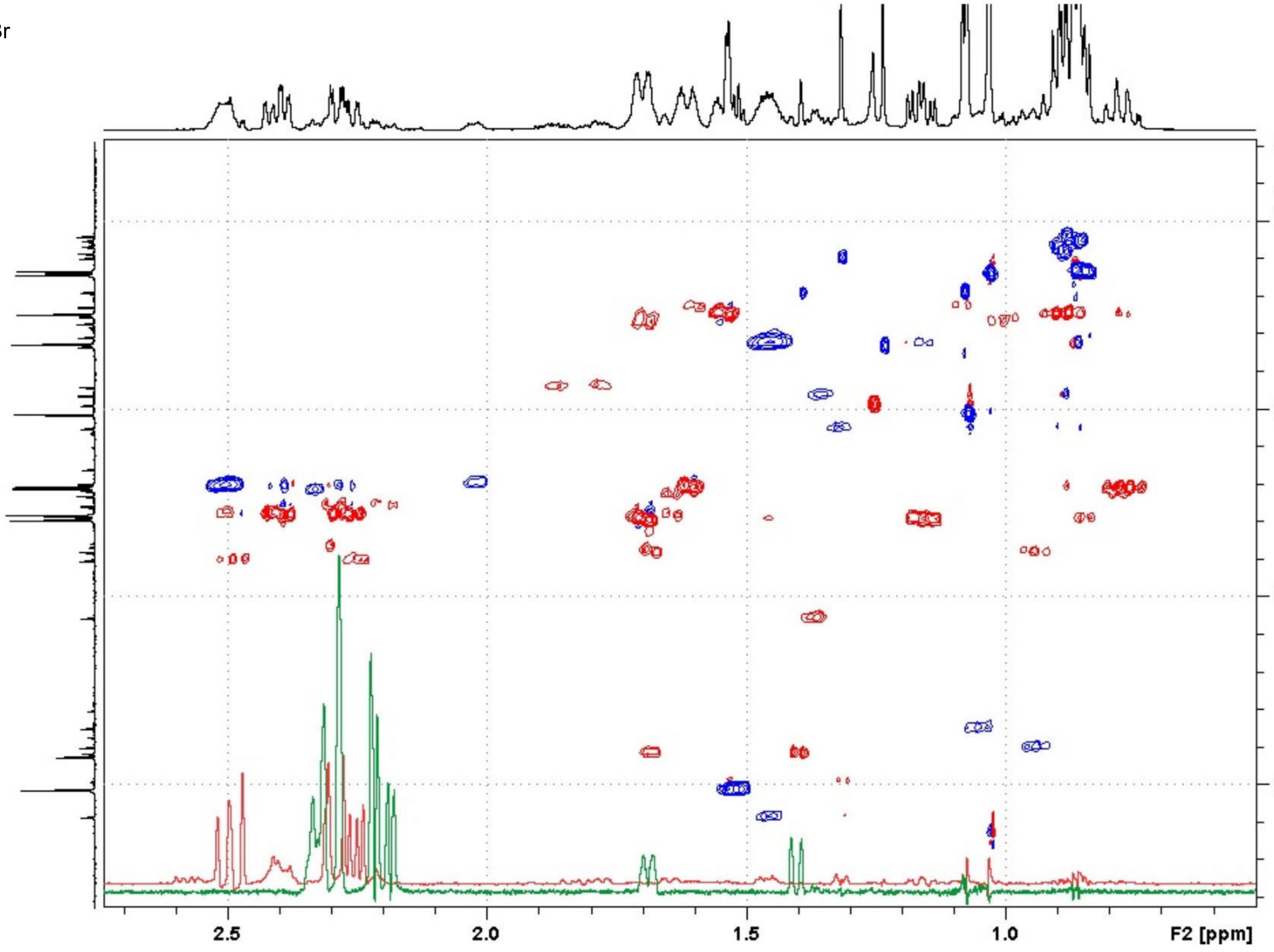

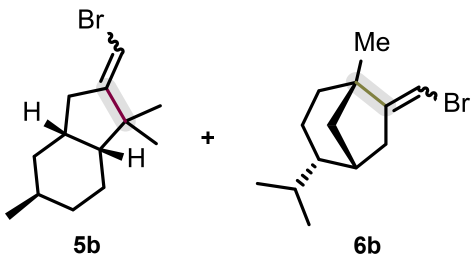

SEL-TOCSYs NMR(600 MHz, $CDCl_3$)

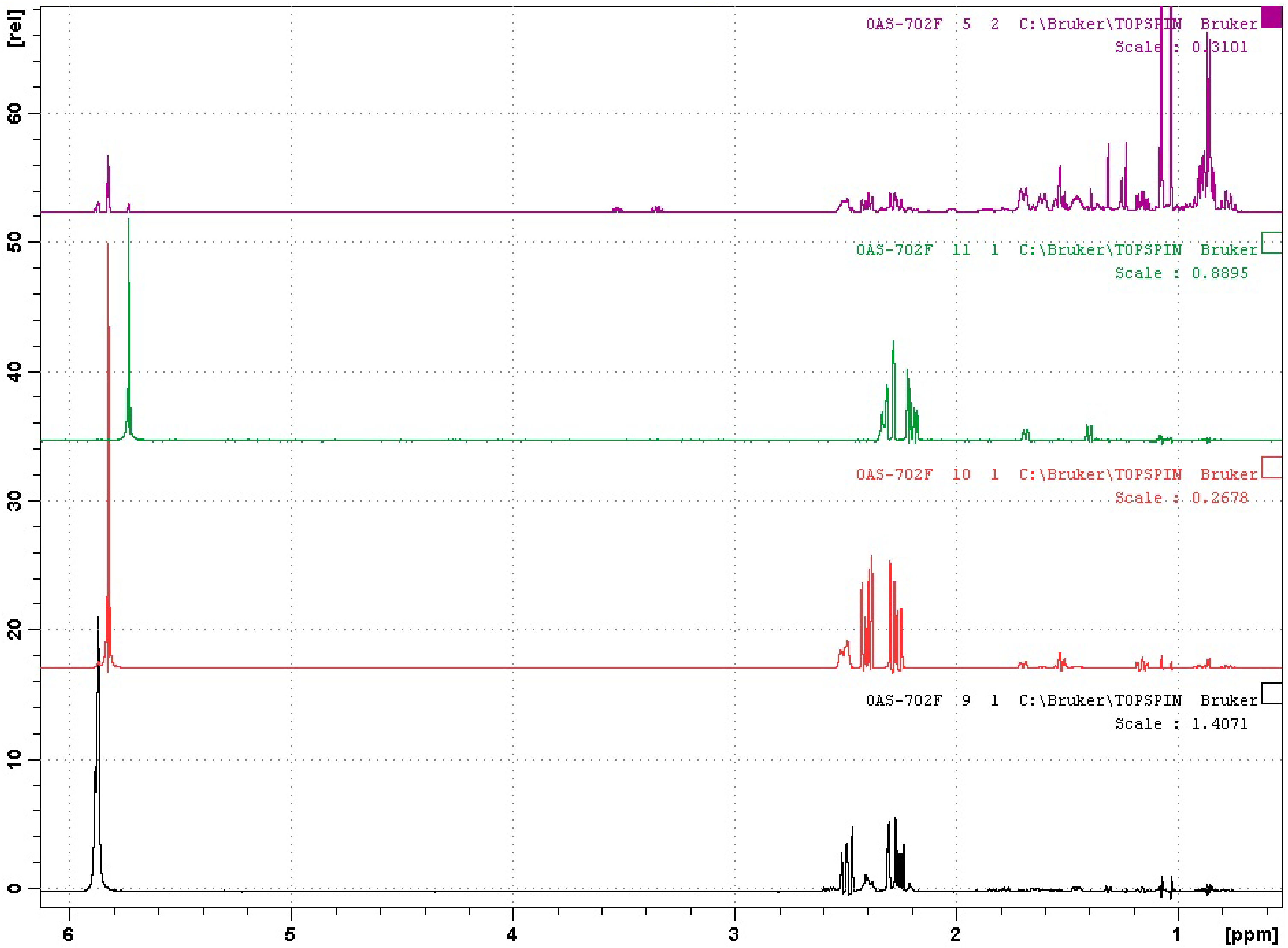

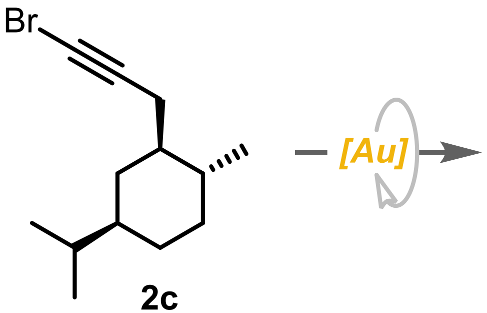
Br
[Au]

2c

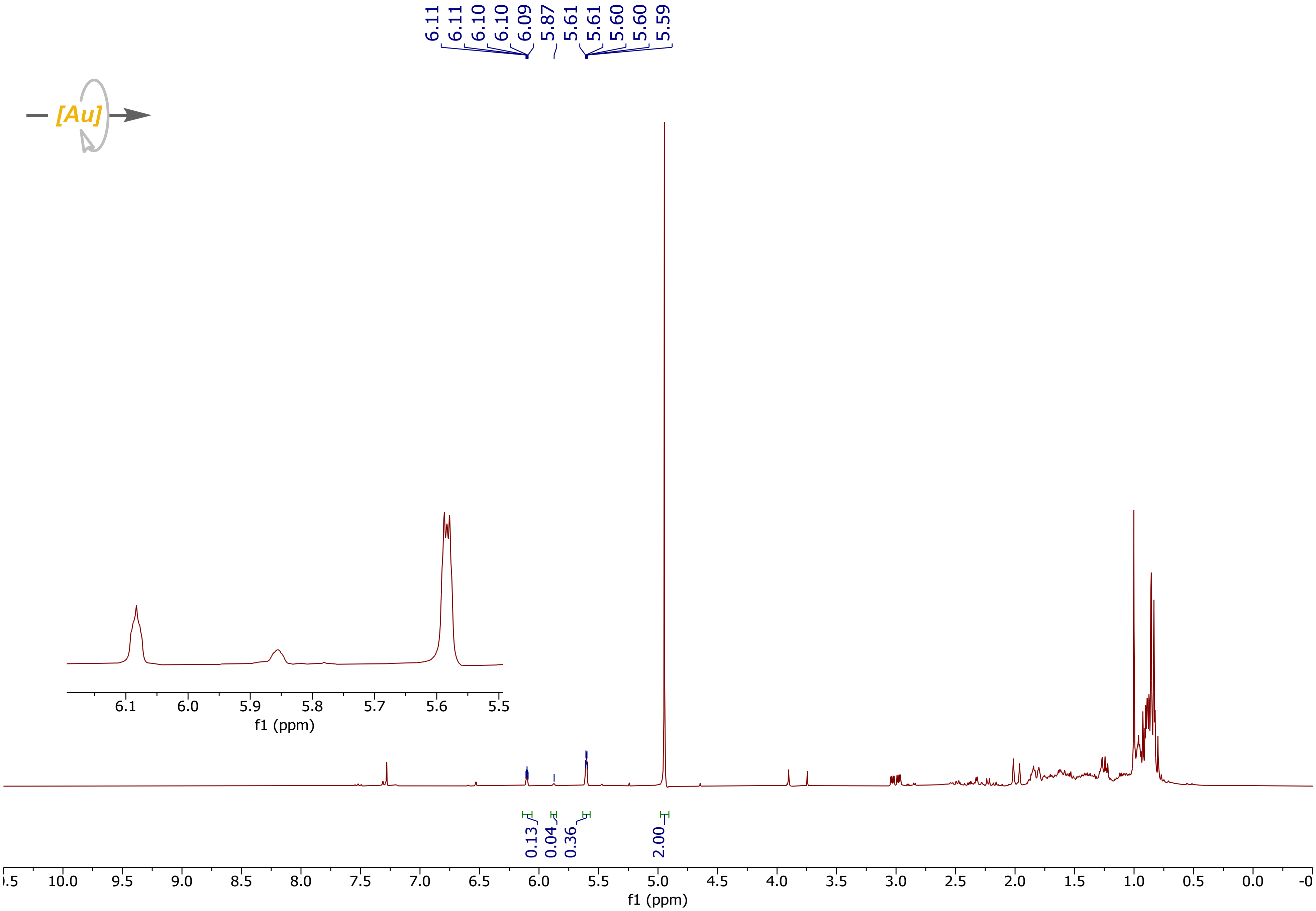
6.11
6.11
6.10
6.10
6.09
5.87
5.61
5.61
5.60
5.60
5.59
6.1
6.0
5.9
5.8
5.7
5.6
5.5
f1 (ppm)
0.13
0.04
0.36
2.00
10.0
9.5
9.0
8.5
8.0
7.5
7.0
6.5
6.0
5.5
5.0
4.5
4.0
3.5
3.0
2.5
2.0
1.5
1.0
0.5
0.0
f1 (ppm)

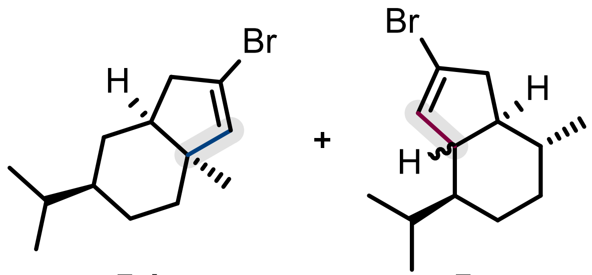

7c' 7c

^{1}H NMR(300 MHz, $CDCl_3$)

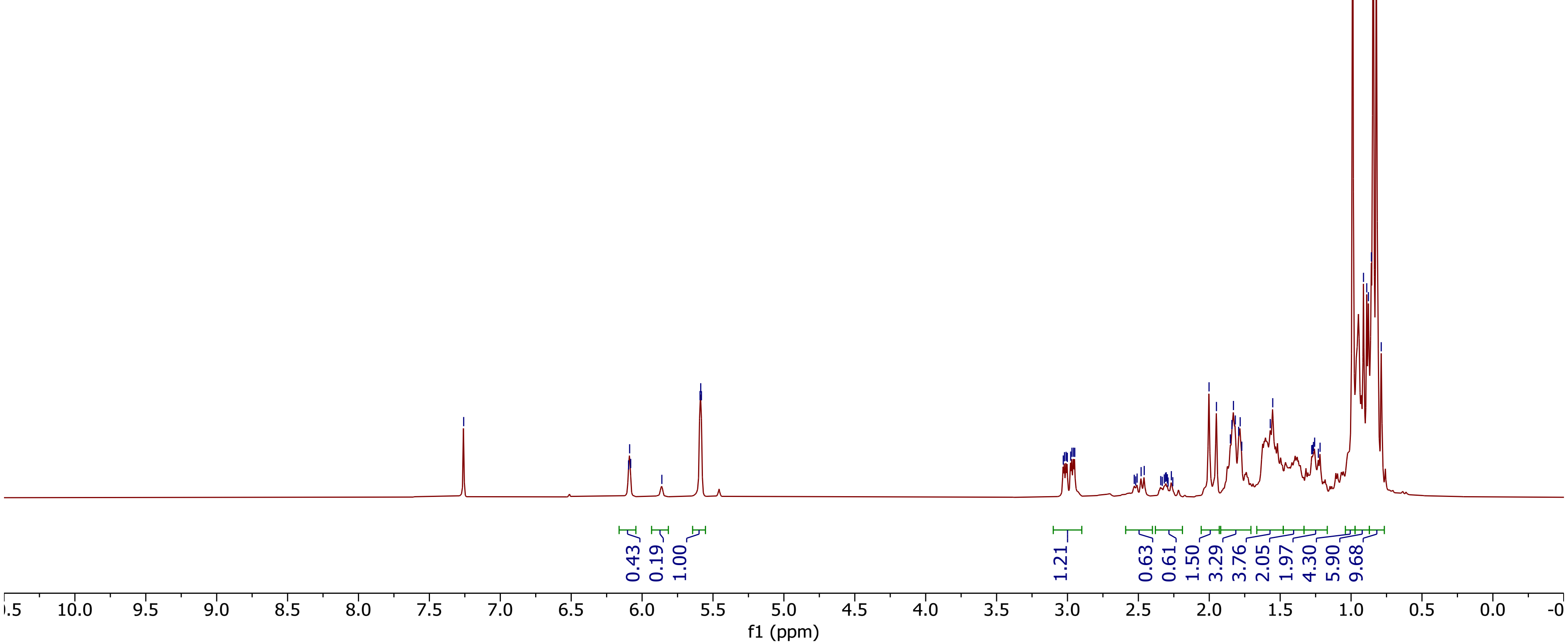

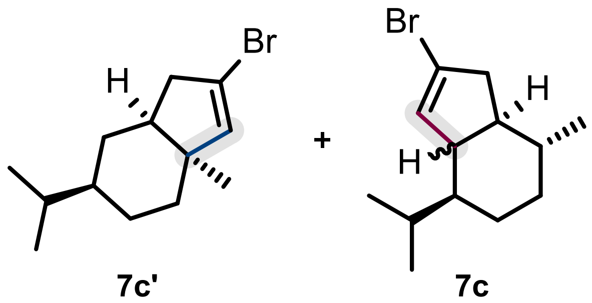

^{13}**C NMR** (75 MHz, $CDCl_3$)

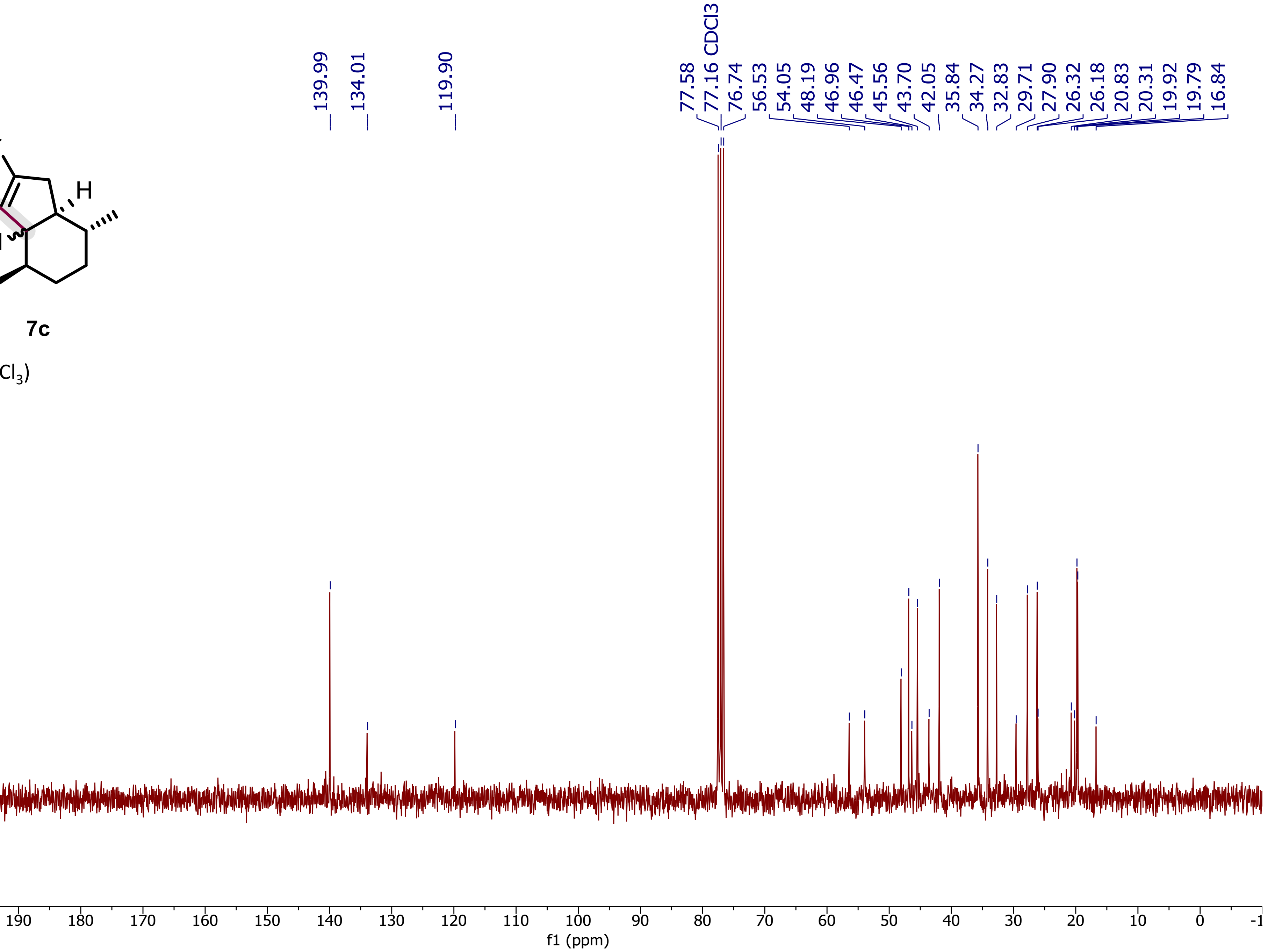

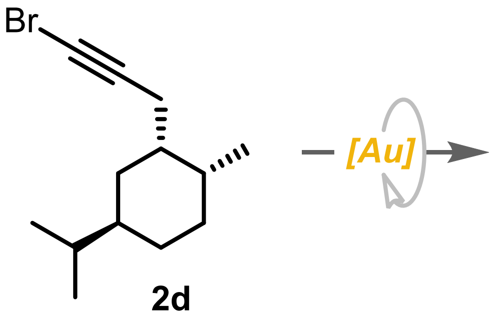
Br
[Au]

2d

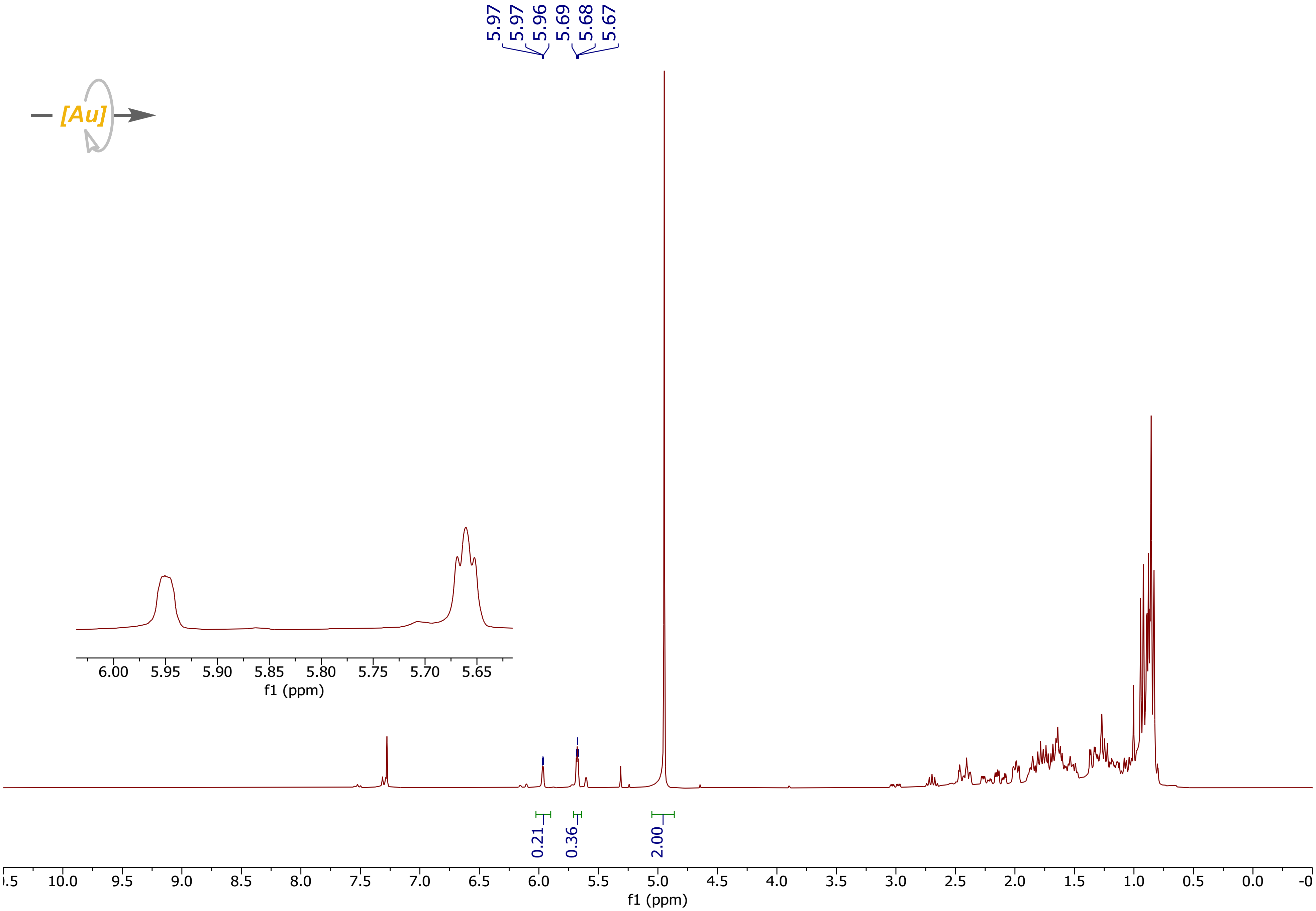
5.97
5.97
5.96
5.69
5.68
5.67
6.00
5.95
5.90
5.85
5.80
5.75
5.70
5.65
f1 (ppm)
0.21
0.36
2.00
10.0
9.5
9.0
8.5
8.0
7.5
7.0
6.5
6.0
5.5
5.0
4.5
4.0
3.5
3.0
2.5
2.0
1.5
1.0
0.5
0.0
f1 (ppm)

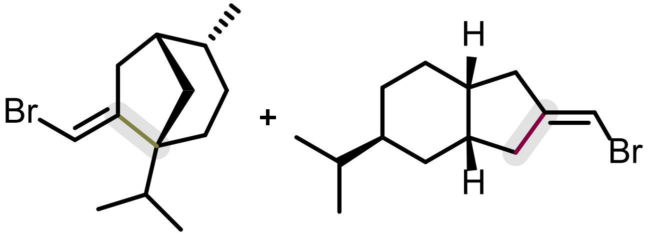

6d **8d**

^{1}H NMR(400 MHz, $CDCl_3$)

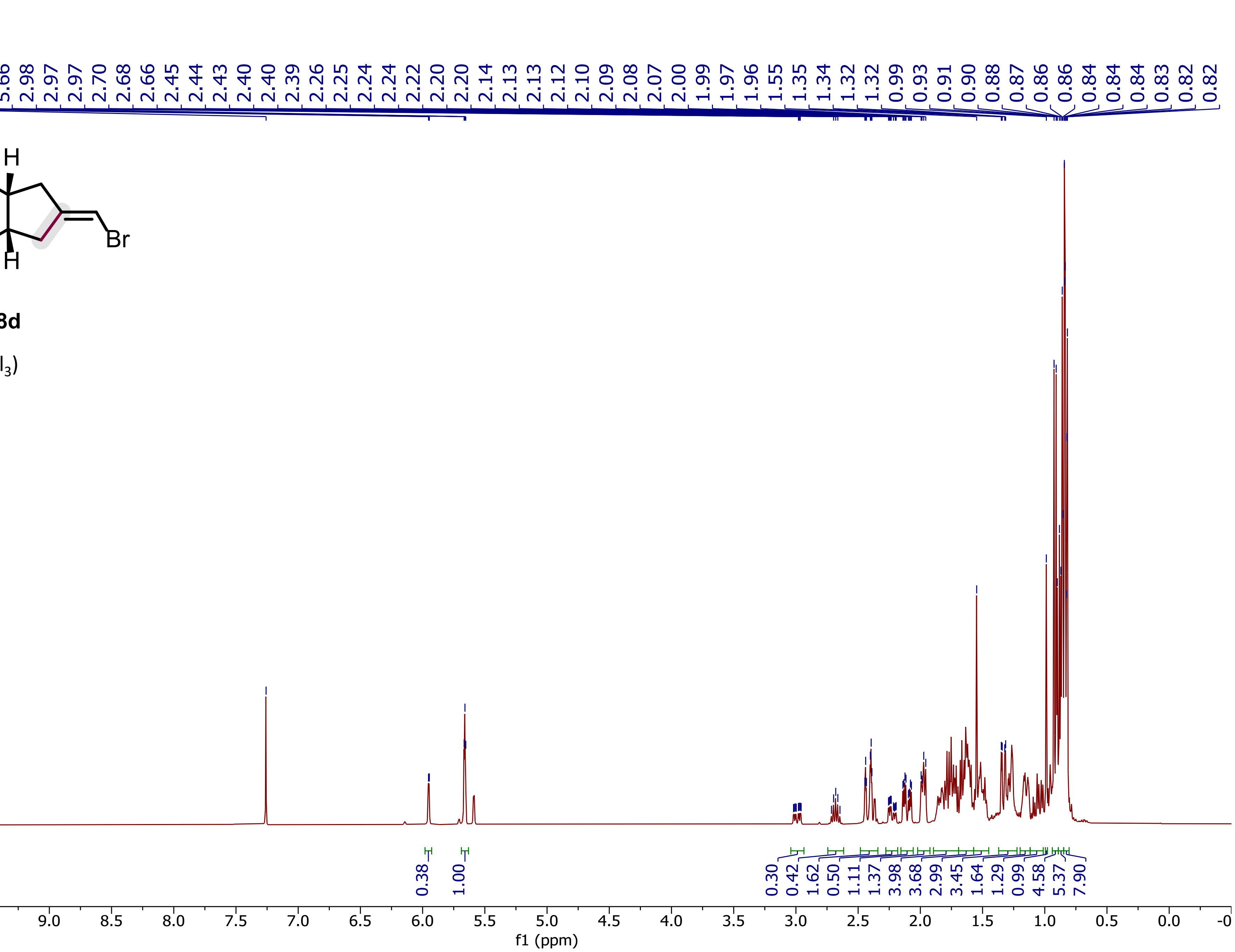

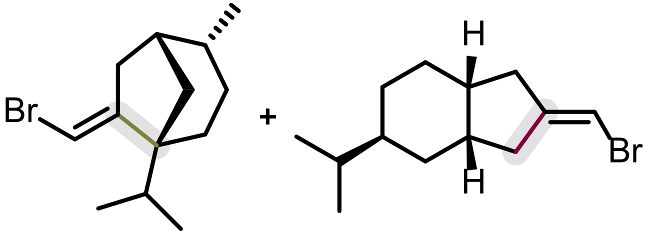

6d **8d**

^{13}C NMR (101 MHz, $CDCl_3$)

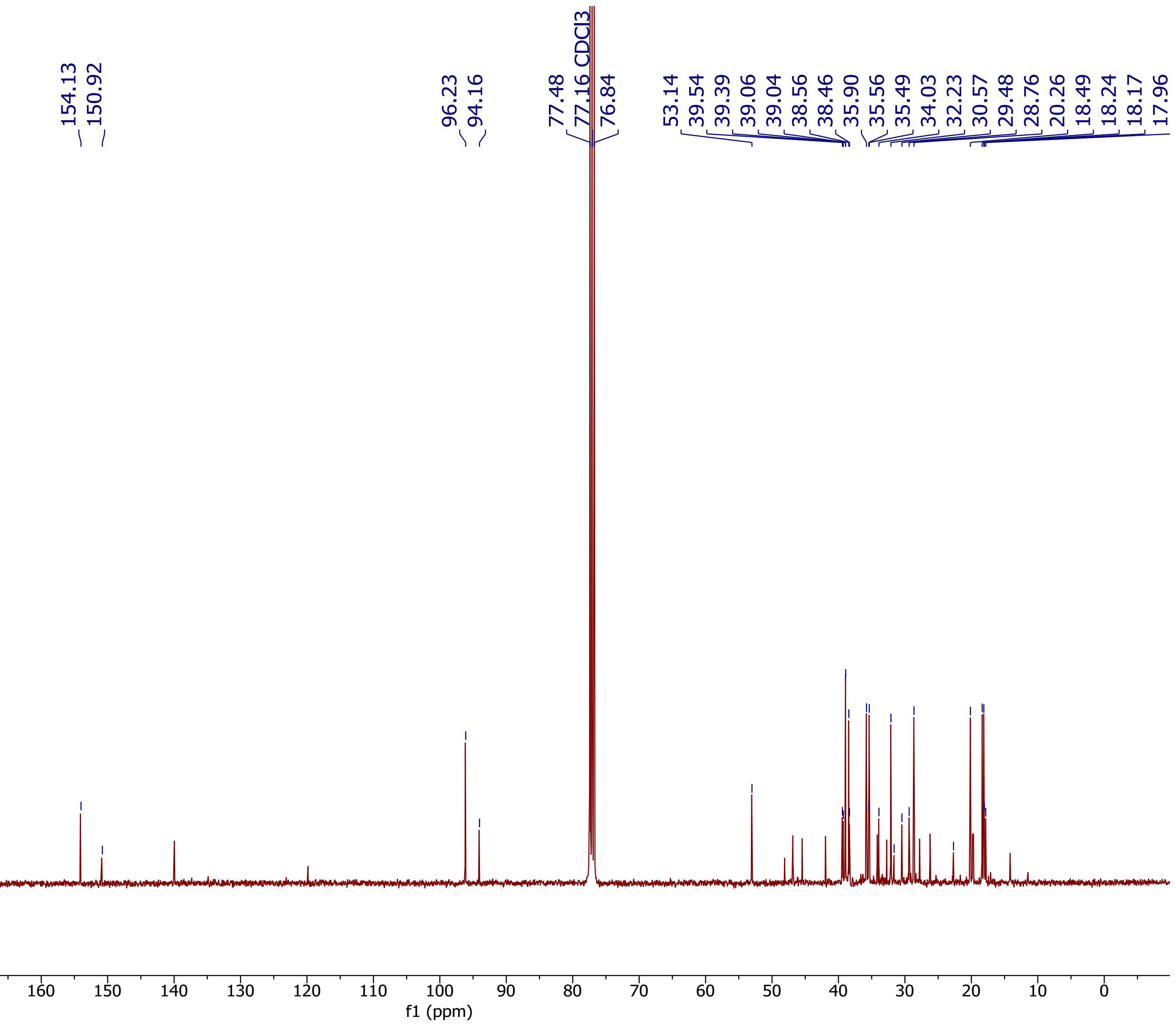

EXO FAMILY

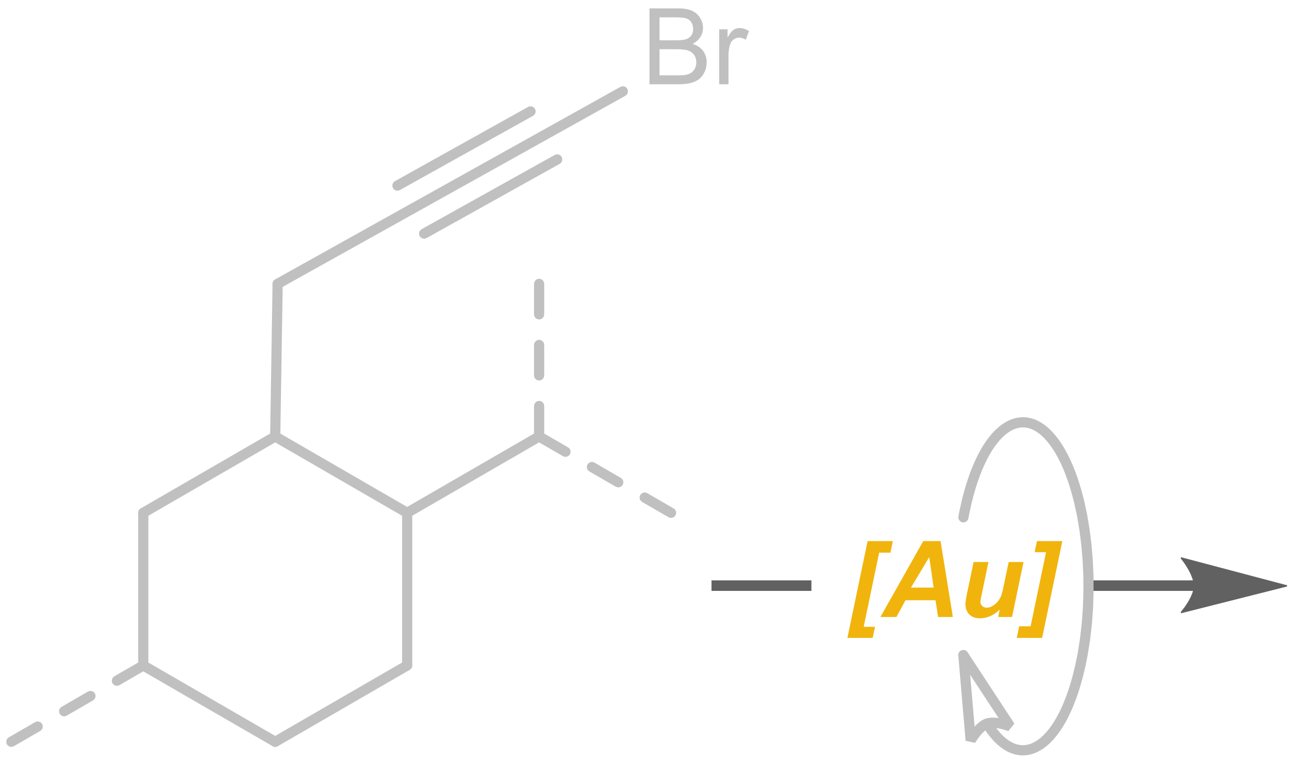

2e-g

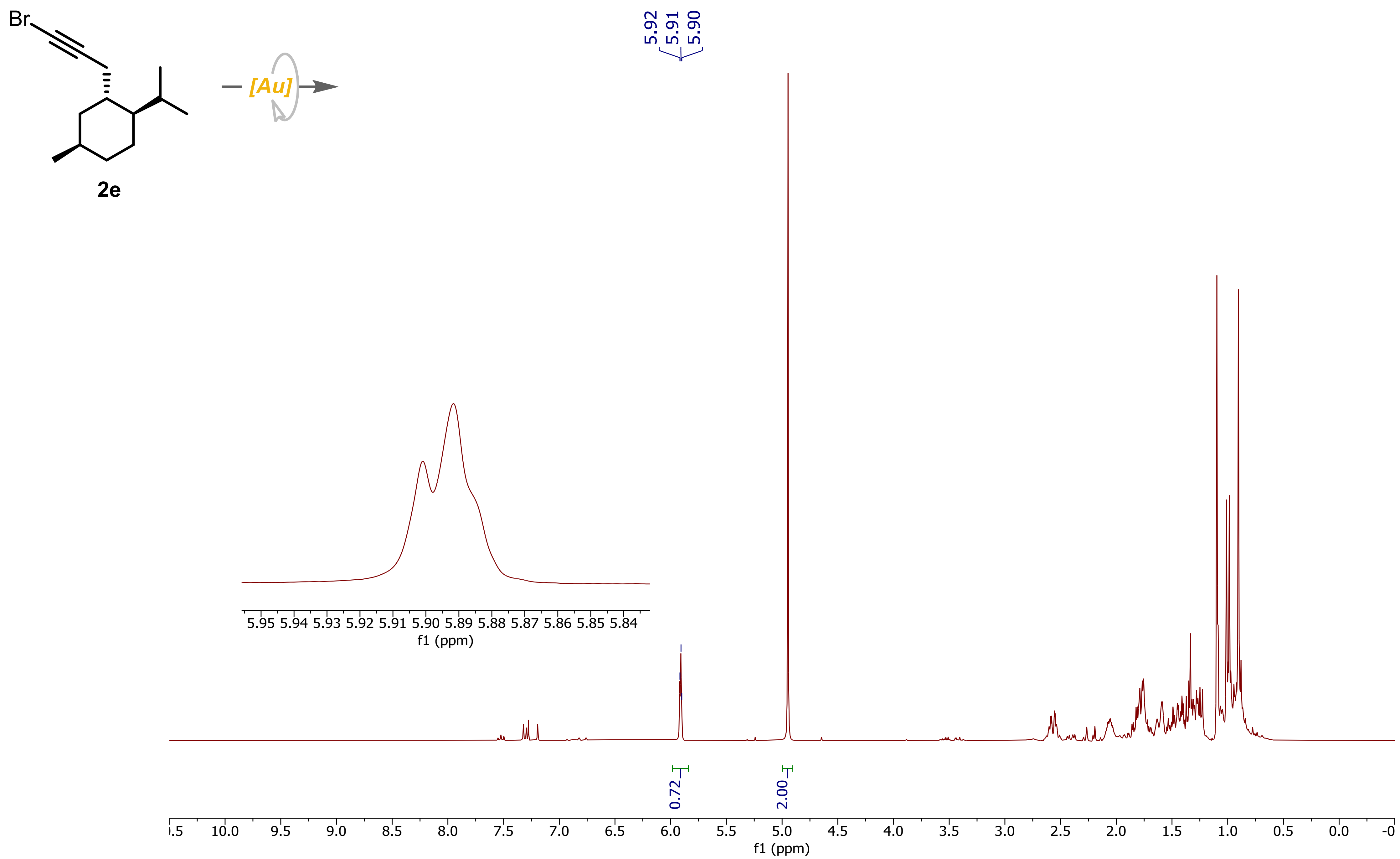
Br
[Au]
2e
5.92
5.91
5.90
5.95 5.94 5.93 5.92 5.91 5.90 5.89 5.88 5.87 5.86 5.85 5.84
f1 (ppm)
0.72
2.00
10.0 9.5 9.0 8.5 8.0 7.5 7.0 6.5 6.0 5.5 5.0 4.5 4.0 3.5 3.0 2.5 2.0 1.5 1.0 0.5 0.0
f1 (ppm)

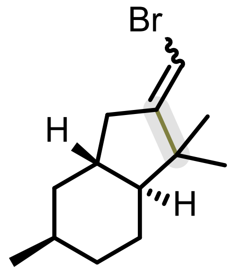

5e

[1]**H NMR**(300 MHz, $CDCl_3$)

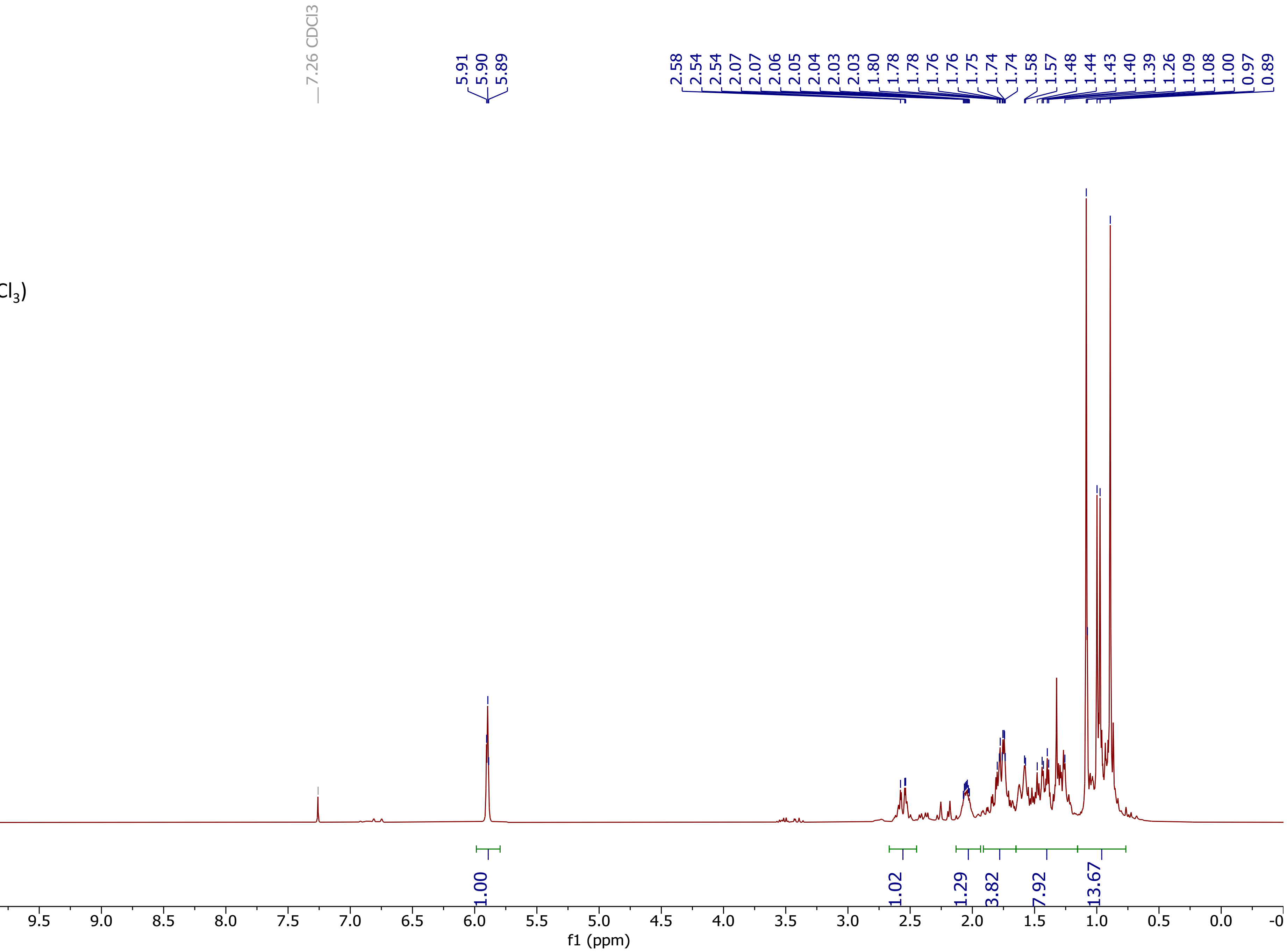

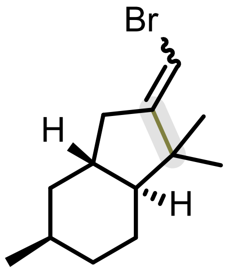

5e

^{13}C NMR (75 MHz, $CDCl_3$)

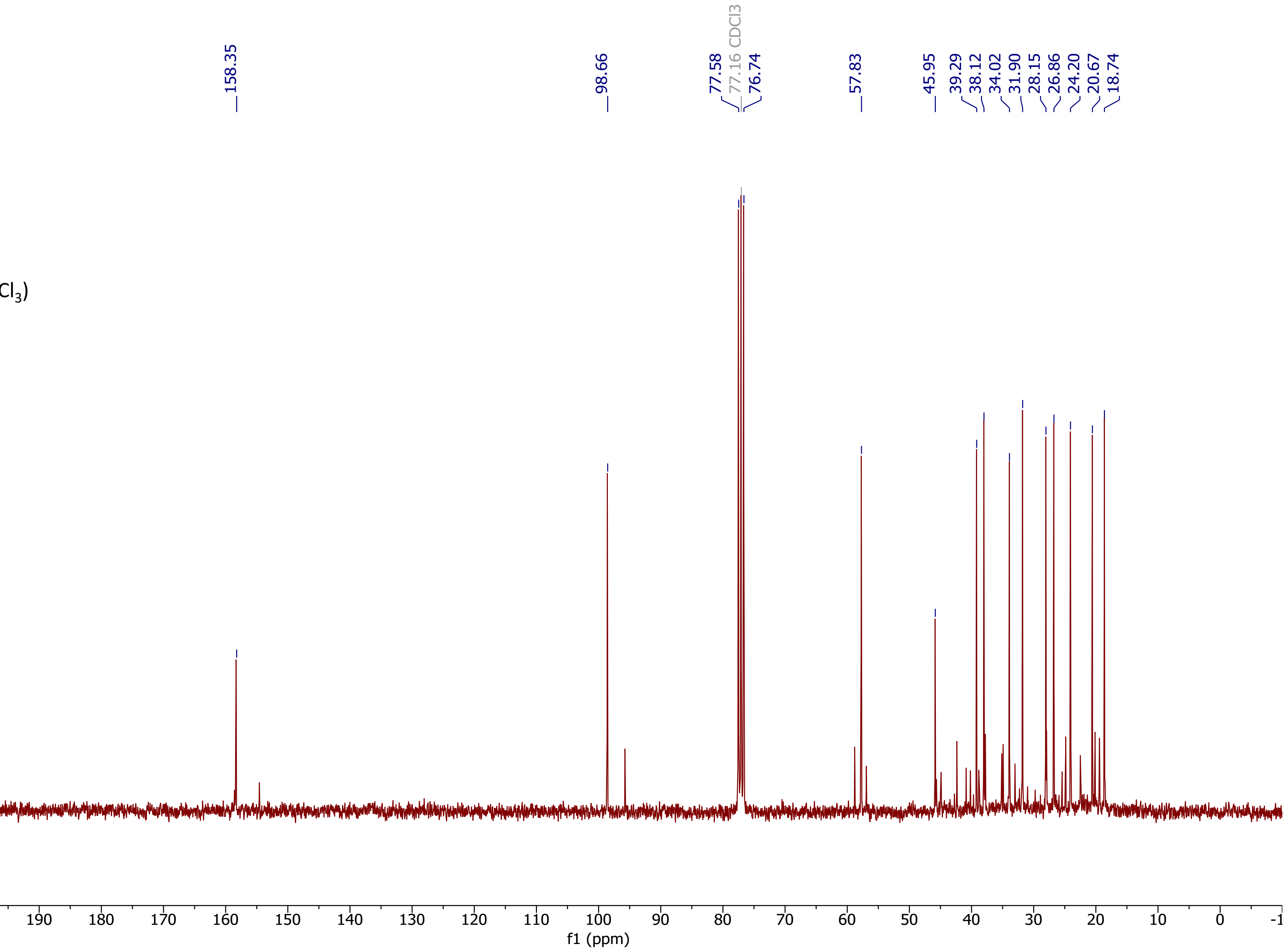

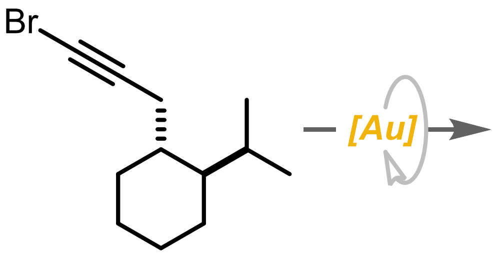
Br
[Au]

2f

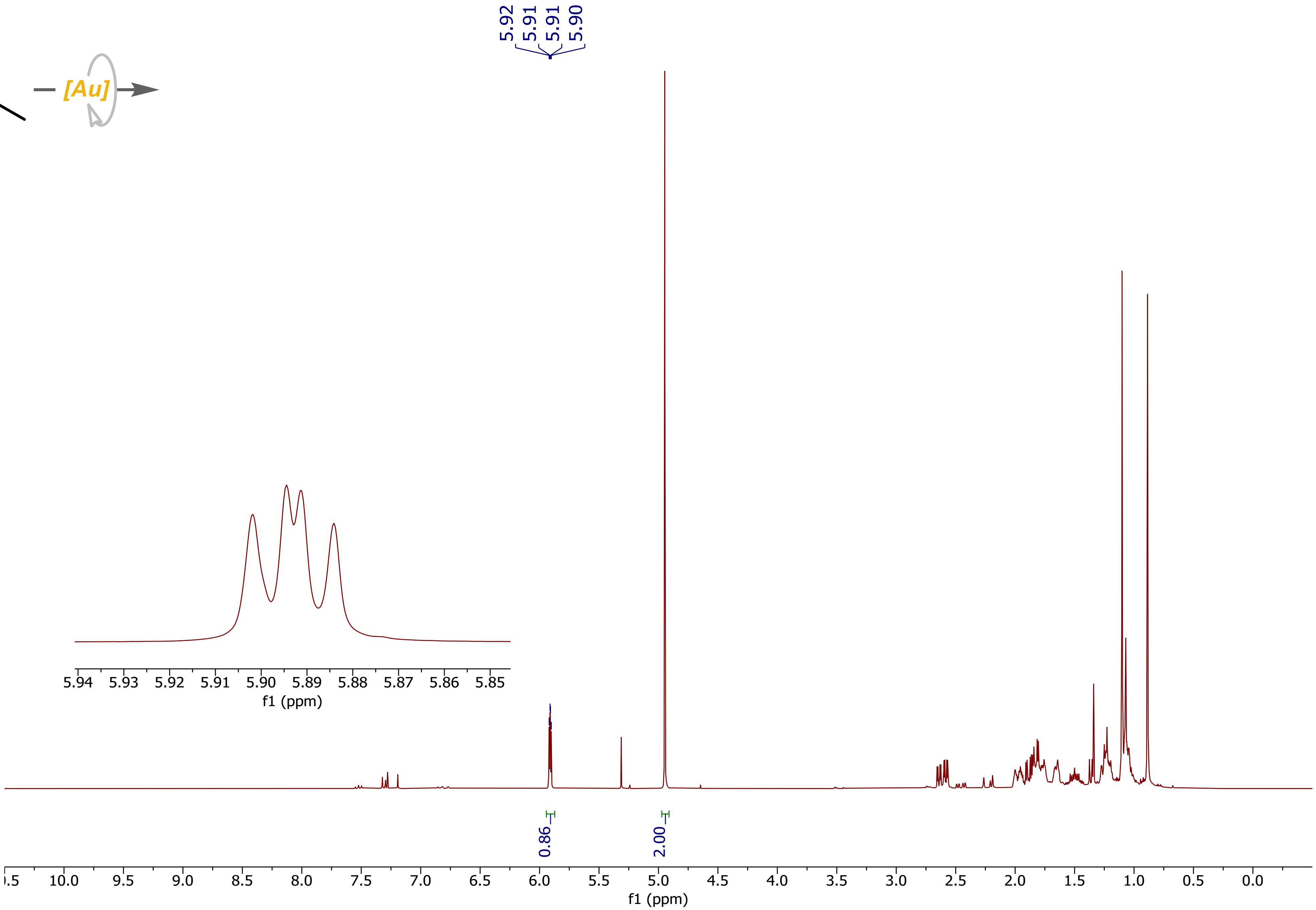
5.92
5.91
5.91
5.90
5.94 5.93 5.92 5.91 5.90 5.89 5.88 5.87 5.86 5.85
f1 (ppm)
0.86
2.00
10.0 9.5 9.0 8.5 8.0 7.5 7.0 6.5 6.0 5.5 5.0 4.5 4.0 3.5 3.0 2.5 2.0 1.5 1.0 0.5 0.0
f1 (ppm)

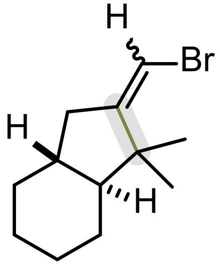

5f

^{1}H NMR(300 MHz, $CDCl_3$)

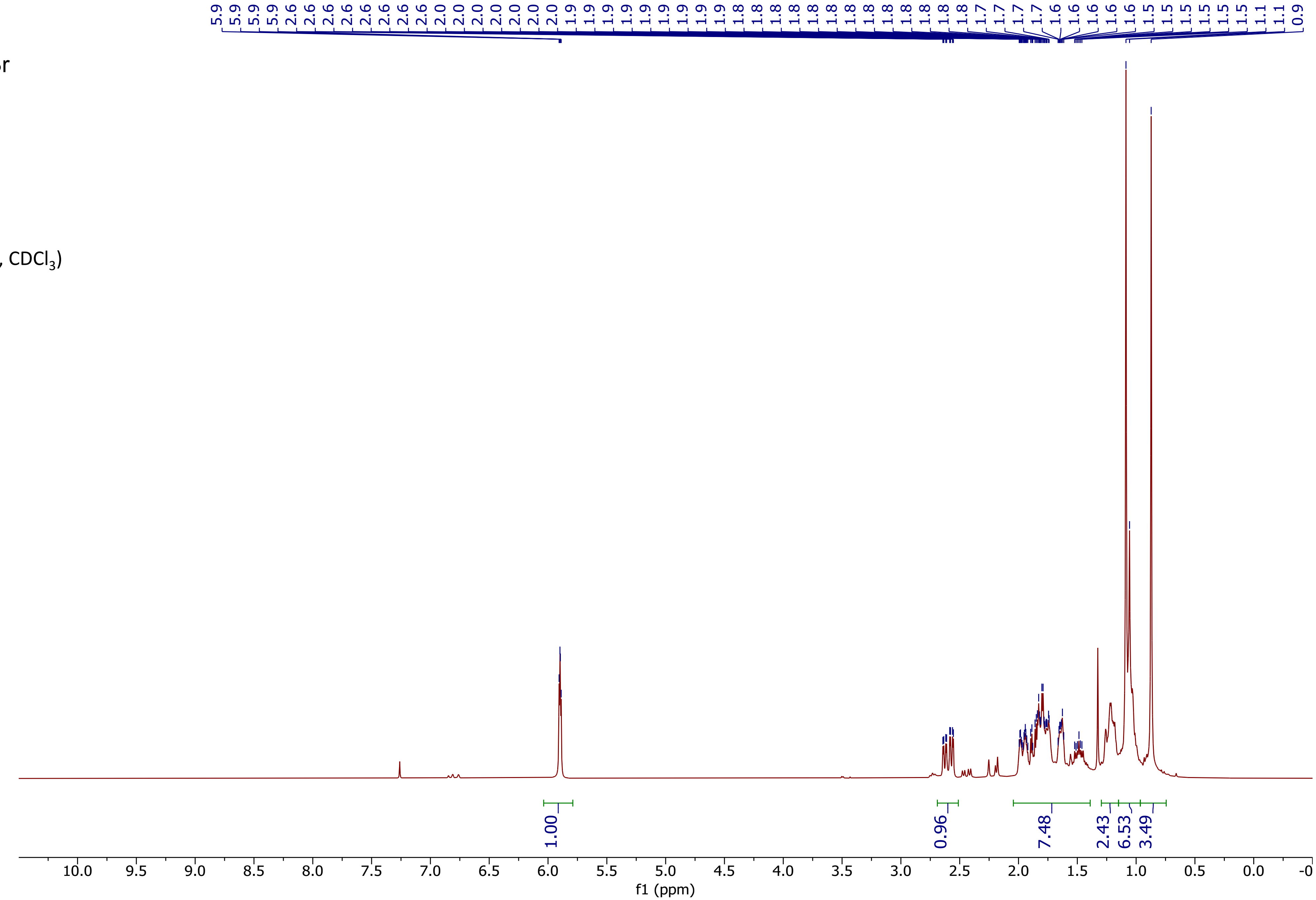

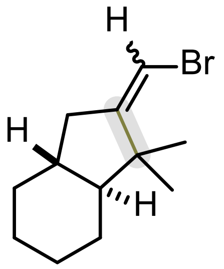

5f

^{13}C NMR (75 MHz, $CDCl_3$)

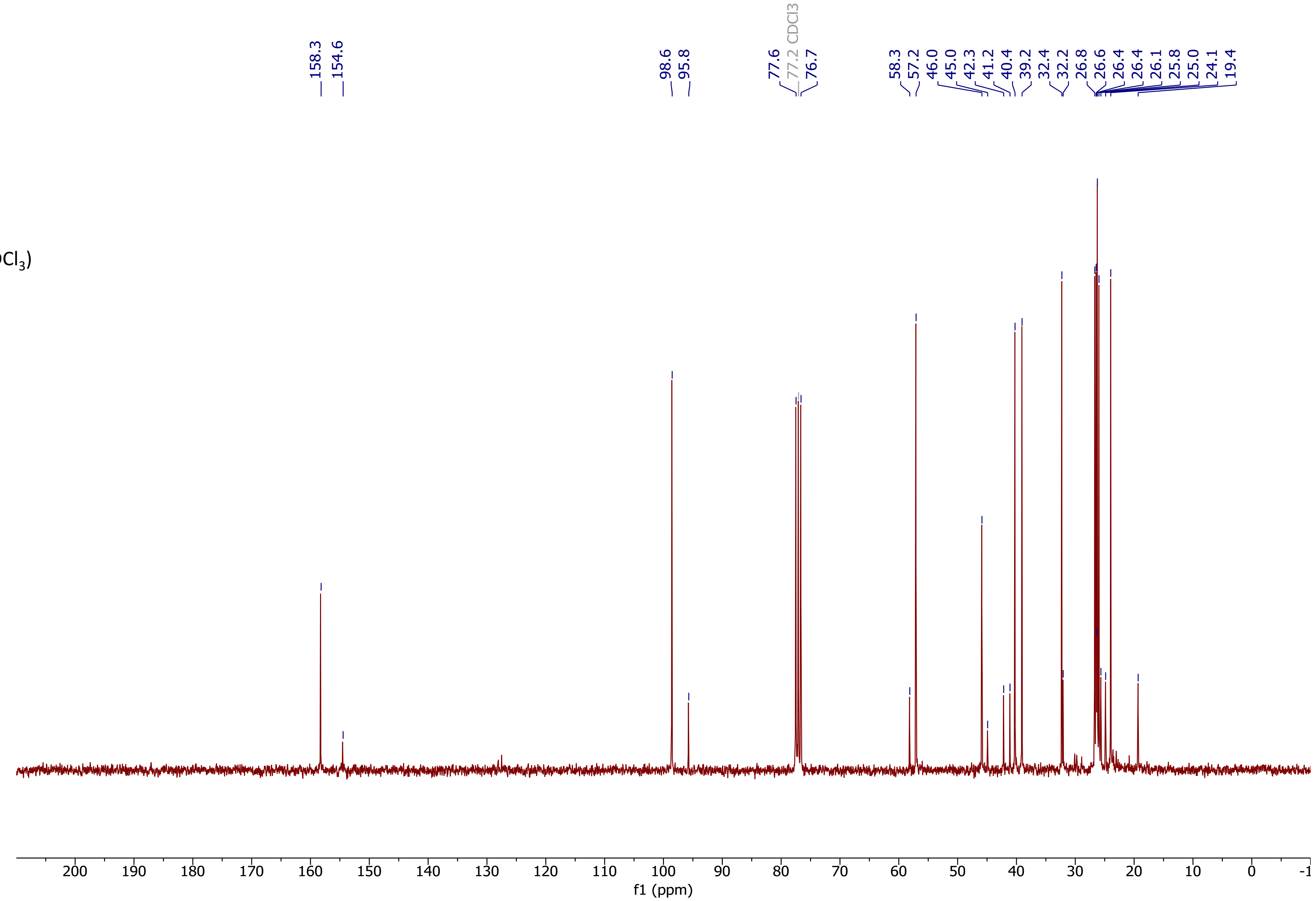

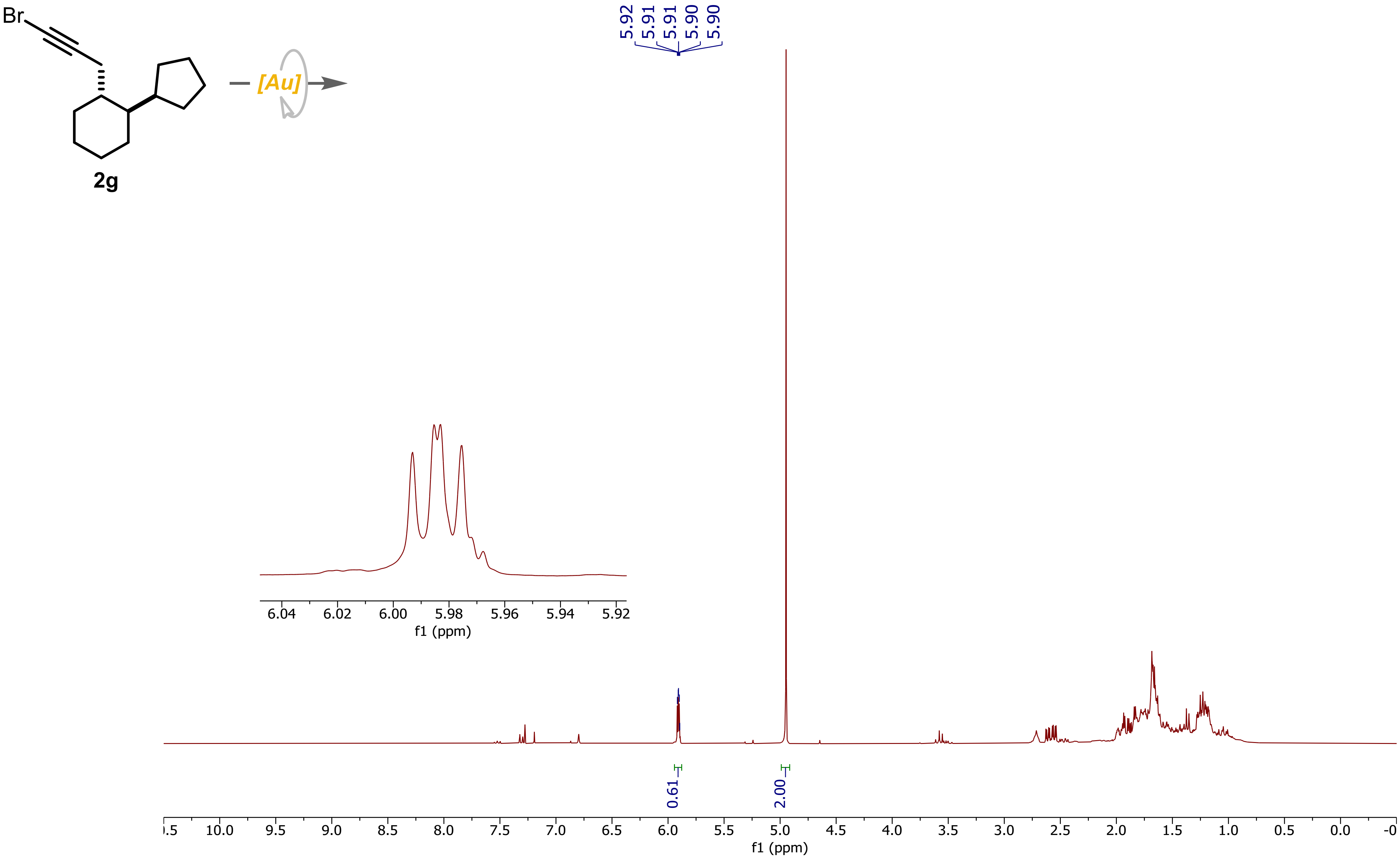
Br
2g
[Au]
5.92
5.91
5.91
5.90
5.90
6.04
6.02
6.00
5.98
5.96
5.94
5.92
f1 (ppm)
0.61
2.00
10.0
9.5
9.0
8.5
8.0
7.5
7.0
6.5
6.0
5.5
5.0
4.5
4.0
3.5
3.0
2.5
2.0
1.5
1.0
0.5
0.0
f1 (ppm)

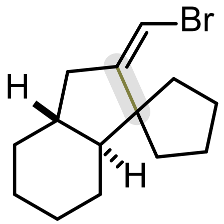

5g

^{1}H NMR(300 MHz, $CDCl_3$)

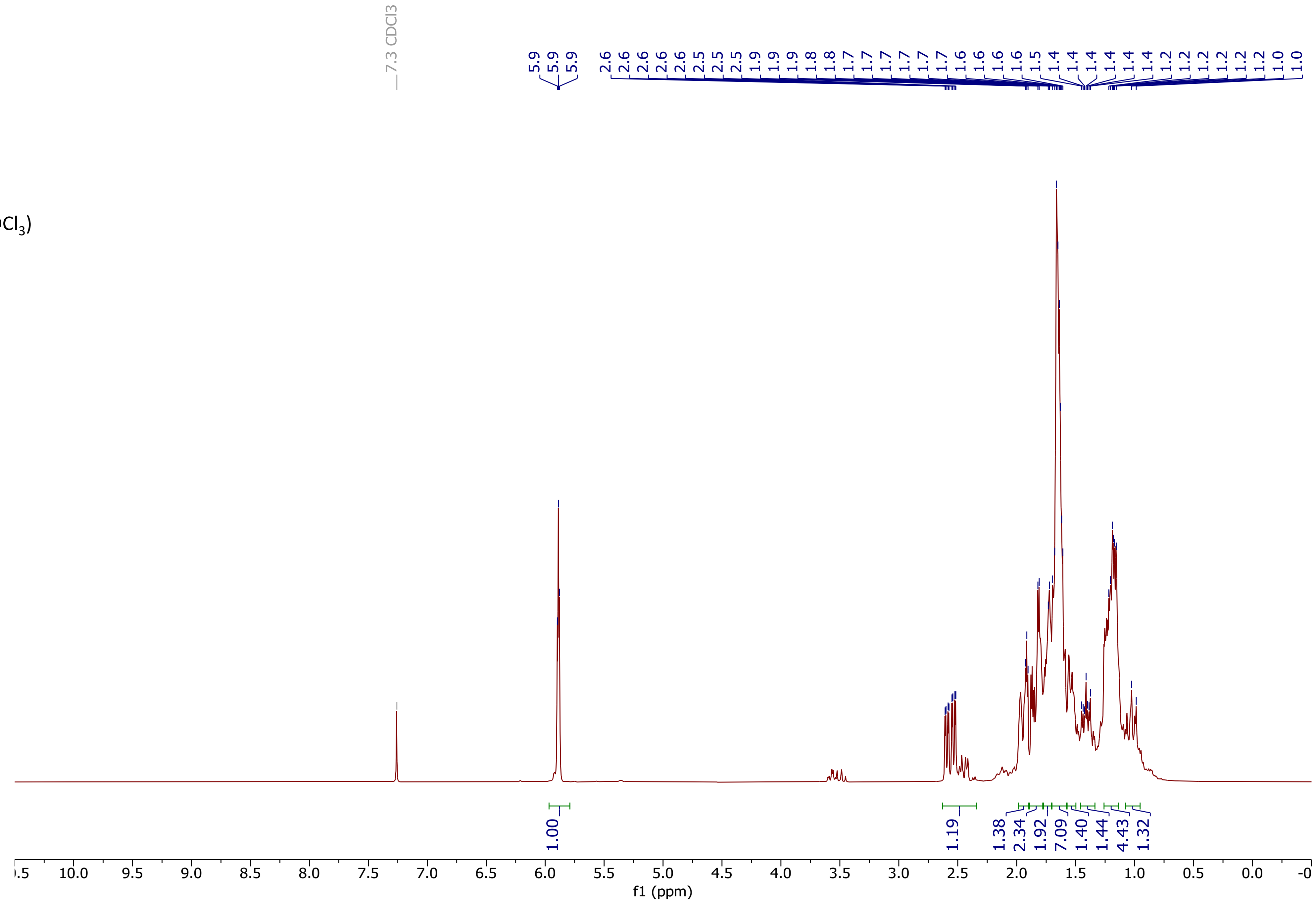

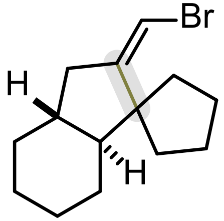

5g

^{13}C NMR (75 MHz, $CDCl_3$)

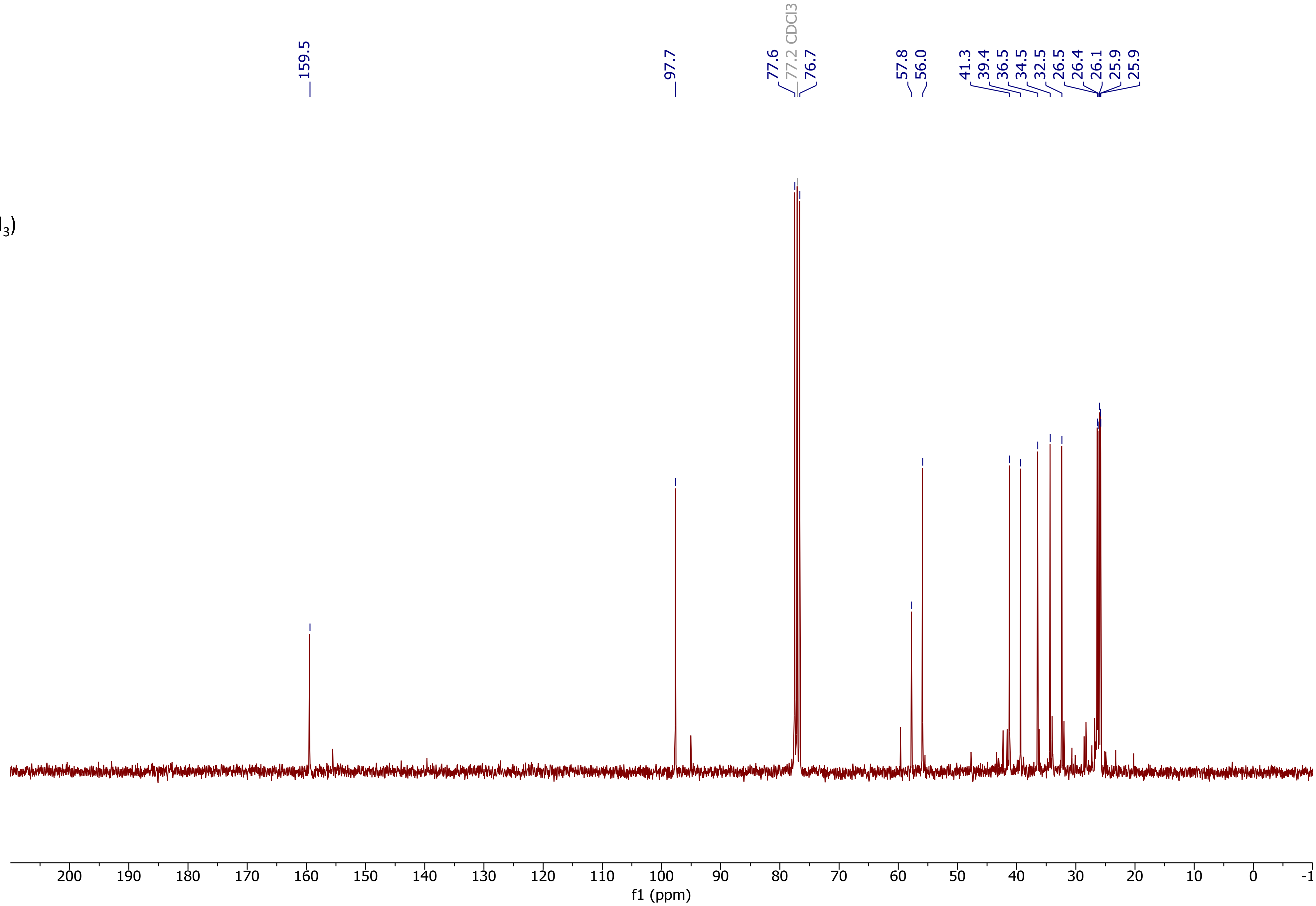

Supplement: Supplementary file 3 [file ol5c03431_si_003.pdf]
